# Using Combined Methods of Genetic Mapping and Nanopore-Based Sequencing Technology to Analyze the Insertion Positions of *G10evo*-EPSPS and *Cry1Ab/Cry2Aj* Transgenes in Maize

**DOI:** 10.3389/fpls.2021.690951

**Published:** 2021-07-29

**Authors:** Cheng Peng, Yingting Mei, Lin Ding, Xiaofu Wang, Xiaoyun Chen, Junmin Wang, Junfeng Xu

**Affiliations:** ^1^State Key Laboratory Breeding Base for Zhejiang Sustainable Pest and Disease Control, Institute of Agro-Product Safety and Nutrition, Zhejiang Academy of Agricultural Sciences, Hangzhou, China; ^2^Biotechnology Research Institute, Chinese Academy of Agricultural Sciences, Beijing, China; ^3^Institute of Crops and Nuclear Technology Utilization, Zhejiang Academy of Agricultural Sciences, Hangzhou, China

**Keywords:** nanopore-sequencing, next-generation sequencing, genetically modified organism, insertion position, genetic mapping

## Abstract

The insertion position of the exogenous fragment sequence in a genetically modified organism (GMO) is important for the safety assessment and labeling of GMOs. SK12-5 is a newly developed transgenic maize line transformed with two trait genes [i.e., *G10evo*-5-enolpyrul-shikimate-3-phosphate synthase (EPSPS) and *Cry1Ab/Cry2Aj*] that was recently approved for commercial use in China. In this study, we tried to determine the insertion position of the exogenous fragment for SK12-5. The transgene–host left border and right border integration junctions were obtained from SK12-5 genomic DNA by using the thermal asymmetric interlaced polymerase chain reaction (TAIL-PCR) and next-generation Illumina sequencing technology. However, a Basic Local Alignment Search Tool (BLAST) analysis revealed that the flanking sequences in the maize genome are unspecific and that the insertion position is located in a repetitive sequence area in the maize genome. To locate the fine-scale insertion position in SK12-5, we combined the methods of genetic mapping and nanopore-based sequencing technology. From a classical bulked-segregant analysis (BSA), the insertion position in SK12-5 was mapped onto Bin9.03 of chromosome 9 between the simple sequence repeat (SSR) markers *umc2337* and *umc1743* (26,822,048–100,724,531 bp). The nanopore sequencing results uncovered 10 reads for which one end was mapped onto the vector and the other end was mapped onto the maize genome. These observations indicated that the exogenous T-DNA fragments were putatively integrated at the position from 82,329,568 to 82,379,296 bp of chromosome 9 in the transgenic maize SK12-5. This study is helpful for the safety assessment of the novel transgenic maize SK12-5 and shows that the combined method of genetic mapping and the nanopore-based sequencing technology will be a useful approach for identifying the insertion positions of transgenic sequences in other GM plants with relatively large and complex genomes.

## Introduction

Globally, the total cultivated land area dedicated to genetically modified (GM) crops has been increasing for several years (James, 2017). During this time, the safety assessment system for the registration of transgenic plants has evolved to require additional key information, such as the detailed molecular characterization of the DNA sequence and the integrity of the transgene locus (Codex Alimentarius Commission., 2003 and European Food Safety Authority., 2010). The current international consensus is that GM crops should be commercialized only after a thorough safety assessment with no findings of unintended effects. The molecular characterization of the insertion positions of the transgenes, including the insertion sequence, its localization, and the number of insertions, as well as its flanking sequences, is considered essential for a sound safety assessment (Kok et al., 2014). The insertion positions of the transgenes may affect the function of the surrounding genes, and it is frequently associated with both intended and unintended changes at the genomic, transcriptomic, proteomic, and metabolomic levels, which could affect the derived food/feed quality and safety (Azpiroz-Leehan and Feldmann, 1997; Sparrow, 2010). Therefore, obtaining the data of robust molecular characterization on the insertion positions of the transgenes and their flanking sequences is valuable for the developers and regulators of GM crops and the risk assessment of the latter. Such data are also useful for the development and validation of the specific detection methods for the monitoring of commercial genetically modified organisms (GMOs) (Miraglia et al., 2004, and Fraiture et al., 2015).

The current legally required and commonly applied approach to determining the insertion position of the transgenic sequence (Codex Alimentarius Commission., 2003 and European Food Safety Authority., 2010) is based on the polymerase chain reaction (PCR) analyses, including thermal asymmetric interlaced polymerase chain reaction (TAIL-PCR) (Liu et al., 1995, and Liu and Chen, 2007), adaptor PCR, which is sometimes referred to as anchored PCR (Singer and Burke, 2003, and Thole et al., 2009), and T-linker PCR, which utilizes a specific T/A ligation (Yuanxin et al., 2003), all of which rely on the sequence information of transgenic elements. The junction sequences of most GM crops such as transgenic rice TT51-1 were obtained *via* those methods (Cao et al., 2011). Yet, these approaches always remain laborious and expensive, and it is especially difficult to achieve high throughput while using them. Instead, the next-generation Illumina sequencing technology has been used successfully to identify the insertion positions of the transgenes in GMOs, given its depth of sequencing capacity and low labor cost (Polko et al., 2012; Lepage et al., 2013; Yang et al., 2013; Guo et al., 2016; and Siddique et al., 2019). However, the next-generation Illumina sequencing-based method produces short reads; these pose a challenge for analyzing the data and resolving the insertion positions of the transgenes in many plant species having complex genomes primarily because of the issues related to genome rearrangements and copy-number variations, which may lead to inaccurately mapped locations (Li et al., 2019). Therefore, long-read sequencers are needed, especially for crops such as maize which have large genomes rich in repetitive sequences (Rabinowicz and Bennetzen, 2006).

SK12-5 is a GM maize plant that was developed in China (Chang et al., 2013), which was transformed with the modified pCAMBIA1300 carrying two trait genes [*G10evo*-5-enolpyrul-shikimate-3-phosphate synthase (EPSPS) and *Cry1Ab/Cry2Aj*]. The *G10evo* encodes an EPSPS, conferring to resistance of plants against the herbicide glyphosate (Li et al., 2013). The *Cry1Ab/Cry2Aj* encodes a fused *Bacillus thuringiensis* (Bt), thus providing SK12-5 with a great potential for protection against Lepidopteran pests, especially against the Asian corn borer (*Ostrinia furnacalis*), a major pest of maize fields in China (Chang et al., 2013, and Zhang et al., 2017) which is resistant to two Bt maize events, namely Mon810 and Bt11 (Xue et al., 2008). Furthermore, some studies have shown that the consumption of transgenic maize SK12-5 pollen has no negative effects on non-target insects, such as green lacewings (Rawat et al., 2011) or *Folsomia candida* (Zhang et al., 2017). Transgenic maize SK12-5 has recently obtained GM safety certificates in China, where it is likely to get approval soon for cultivation. A complete molecular characterization of its transgene inserts will expedite this process.

We initially tried to analyze the insertion position of the exogenous gene in GM maize, namely SK12-5. Based on the results of the TAIL-PCR and next-generation Illumina sequencing, the extension of the T-DNA flanking DNA sequence in SK12-5 was obtained; this was located in the long terminal repeat (LTP) region of the maize genome. Despite conducting a bioinformatics analysis of that flanking sequence, it was still difficult to determine the insert position of SK12-5 by using the conventional methods. To overcome this problem, in this study, the combined methods of genetic mapping (Zhang et al., 2015, and Michelmore et al., 1991) and nanopore-based sequencing technology (Giraldo et al., 2020) were applied to further analyze the insertion position of SK12-5. The findings of this study are therefore very helpful for understanding the molecular characterization of SK12-5.

## Materials and Methods

### Plant Materials

Transgenic maize SK12-5 was originally produced through the transformation of an inbred line Zheng58 (Z58) by Prof. Zhicheng Shen (Zhejiang University, Hangzhou, China). The plasmid used for transforming Z58 was a modified binary vector pCAMBIA1300. The *Cry1Ab/Cry2Aj* was cloned into the binary vector pCAMBIA1300 under the control of the maize *ubiquitin* promoter, and the selective marker *Hpt* gene was replaced by *G10evo*-EPSPS.

The F_2_ mapping populations segregating for the exogenous fragment were developed by crossing two parental inbred lines, namely, GM SK12-5 and non-GM Chang7-2 (C7). All plants were planted with 0.25-m spacing between individuals, under normal field management practices, in the experimental field of Zhejiang University during spring and summer crop seasons of 2018.

### Extraction and Preparation of DNA

For all tests, DNA was prepared from leaf samples by using a DNA Extraction Kit for GMO Detection, v3.0 (Takara, Shiga, Japan). According to the instructions of the manufacturer, the DNA was quantified using the PicoGreen reagent (Qubit dsDNA BR Assay Kit, Invitrogen, Shanghai, China). The purity of the extracted DNA was assessed by the ratio of absorbance at 260 and 280 nm, measured by a spectrophotometer (Ultrospec 1100 pro, GE Healthcare, USA), which was found to be in the range of 1.8–1.9. The integrity of DNA was further characterized by using agarose gel electrophoresis.

### Next-Generation Illumina Sequencing

The qualified DNA samples were randomly sheared into fragments, whose average length was 400 bp, and the truncated DNA fragments were end-repaired, polyA tail-added, sequencing adapter-added, purified, and PCR-amplified; the ensuing products were used to construct libraries with the Nextera XT DNA Sample Preparation Kit, according to the instructions of the manufacturer (Illumina, San Diego, USA). These libraries were then subjected to sequencing on the Illumina NovaSeq 6000 platform (Illumina, Inc., USA), and 150-bp paired-end reads were generated.

The data obtained from the sequencer were processed for quality control. Paired-end sequencing of DNA and simple modifications to the raw data were performed at Biozeron Biotechnology Co., Ltd. (Shanghai, China) to ensure the sequencing quality. Filtering was applied to remove any reads of insufficient length or reads having a low read quality and to trim away the adapter sequences before conducting the data analysis.

### Nanopore-Based Sequencing

The SK12-5 genome DNA was end-repaired with an Ultra II End-prep enzyme mix (E7546L, NEB), followed by purification with AMPure XP beads (Beckman Coulter, USA). After completing the end-repair process, DNA was ligated to the sequencing adapter, using the Blunt/TA Ligase Master Mix (M0367L, NEB). Then, the barcoded DNA was purified again with AMPure XP beads. To measure the concentration of the DNA library, a Qubit dsDNA HS Assay Kit and a Qubit 3.0 Fluorometer (Life Technologies, Carlsbad, CA, USA) were used according to the instructions of the manufacturer. Then, the adapted DNA libraries were sequenced in a one-flow cell (PromethION, Nanopore, Oxford, UK); after about 72 h, the sequencing was stopped.

**Figure 1:**
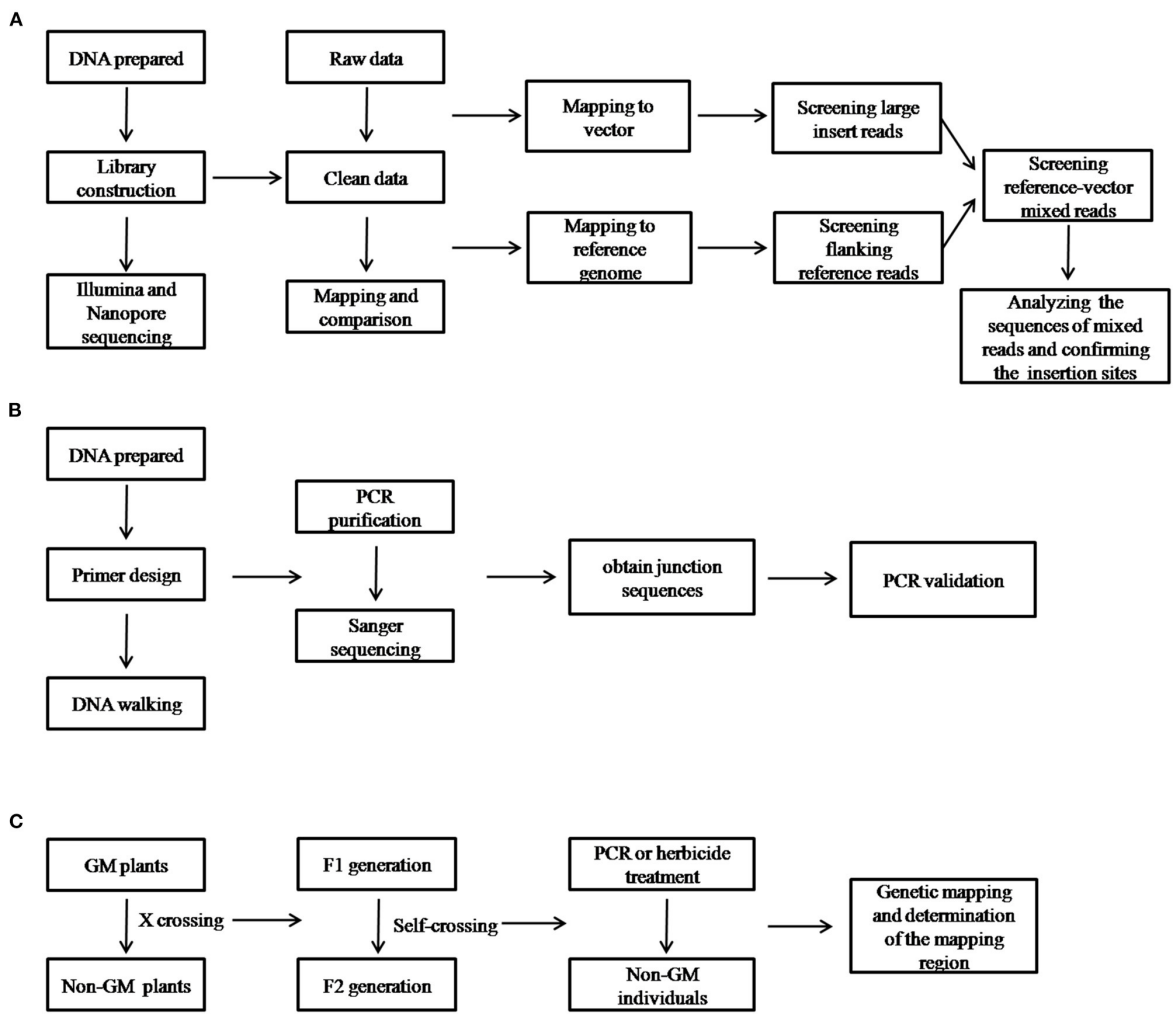
Schematic diagram of the workflow for the identification of the insertion site and its validation using the different techniques implemented in this study. **(A)** Illumina and nanopore sequencing; **(B)** thermal asymmetric interlaced polymerase chain reaction (TAIL-PCR); and **(C)** genetic mapping.

To analyze the data of nanopore sequencing, the Guppy software (v2.2, Oxford Nanopore Technologies, Oxford, UK) was used to transform raw nanopore data into base calls and quality scores. The adapter and low-quality reads (<Q7) were removed. The pipeline for the data analysis and validation is briefly described in Figure 1. After mapping and screening all of the reads to the maize genome and vector, only the reference-vector-mixed reads were retained and selected for use in the formal analysis.

### Cloning and Analysis of PCR Products

The PCR products were purified with a universal DNA purification kit (Tiangen, China) and then cloned into a pMD18-T vector (Takara, Japan) for sequencing. The obtained insert sequence was further analyzed by blasting against the nucleotide (nt) database of NCB, using its default search parameters (http://www.ncbi.nlm.nih.gov/BLAST/Blast.cgi).

### Mapping of the Transgenes

To identify those markers linked to the transgenes, we used both parents and F_2_ plants with homozygous non-transgenic individuals. Based on the maize database (MaizeGDB; http://www.maizegdb.org), a total set of 283 simple sequence repeat (SSR) markers with a uniform distribution on the maize genome were used to screen among parents and non-transgenic individuals of the F_2_ population. Among the 93 polymorphic SSRs across the population, the exogenous fragments were linked with *umc1191* and *umc1691*. The linkage was considered viable if the genotypes of all the non-transgenic individuals from F_2_ population were consistent with the genotype of the non-transgenic parent.

The PCR was carried out using the protocol developed by Menkir et al. (2005). The amplified products were resolved on a 3% super-fine-resolution agarose gel (Amresco, USA) and were visualized with ethidium bromide.

## Results

### Identification of Genetically Modified Maize SK12-5

**Figure 2:**
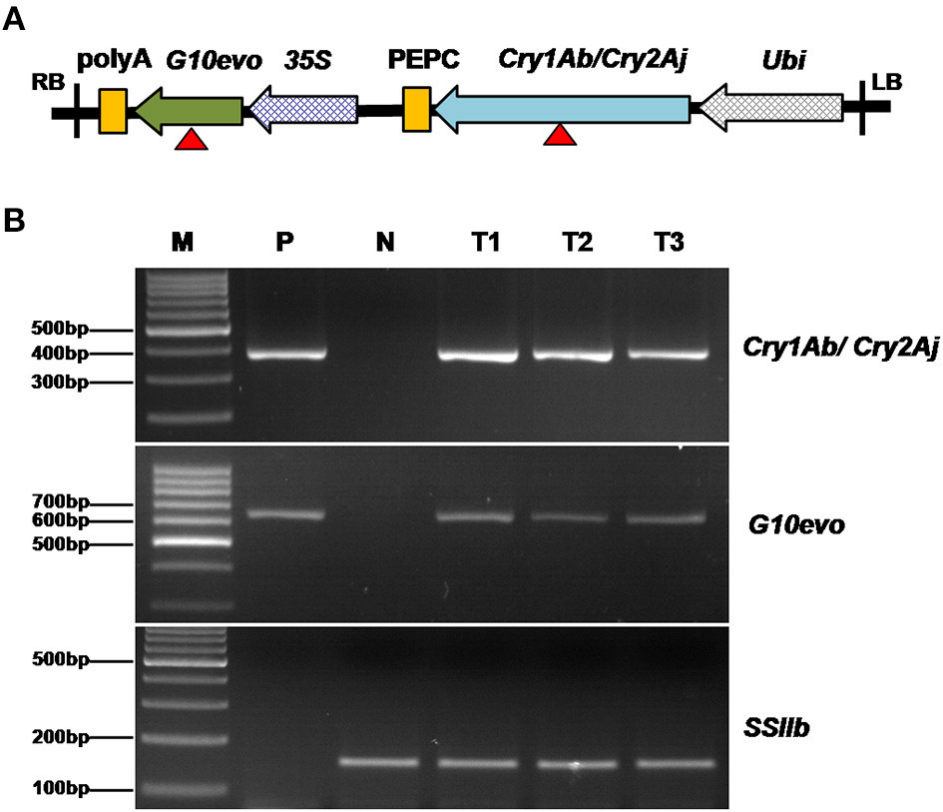
Detection of genetically modified maize SK12-5. **(A)** Organization of transgenic elements used in SK12-5; the red triangle indicates the location of the primes for the detection of *G10evo* and *Cry1Ab/Cry2Aj*. **(B)** Notably, 3% agarose gel electrophoresis of polymerase chain reaction (PCR) products amplified with the *Cry1Ab/Cry2Aj, G10evo* event-specific primers and the maize *SSIIb* gene prime. M, the 1,000-bp DNA ladder; P, transgenic plasmid as the positive control; N, non-transgenic maize line Zheng58 as the negative control; and T1–T3, the three samples of SK12-5.

The GM maize SK12-5 has recently obtained GM safety certificates in China (http://www.moa.gov.cn/ztzl/zjyqwgz/spxx/202104/t20210407_6365331.htm). We began by identifying the molecular characteristics of SK12-5, including information of its insertion site and flanking sequence. SK12-5 was originally transformed with a modified pCAMBIA1300, carrying genes for two traits (i.e., *G10evo*-EPSPS and *Cry1Ab/Cry2Aj*; Figure 2A). Based on the sequences of *G10evo* and *Cry1Ab/Cry2Aj*, we designed two pairs of primers to detect whether the exogenous gene was successfully transferred to the non-transgenic maize line Z58. The transgenic plasmid and DNA of Z58 served as positive and negative controls, respectively, while the *SSIIb* gene was used as an internal control for maize genomic DNA. The two primers did not appear as band in Z58, while SK12-5 displayed the same bands as the positive control in three different individuals (Figure 2B). These results confirmed that the line SK12-5 is positive for being a transgenic maize containing the *G10evo* and *Cry1Ab/Cry2Aj* genes.

### Characterization of T-DNA/Maize DNA Junction by TAIL-PCR

**Figure 3:**
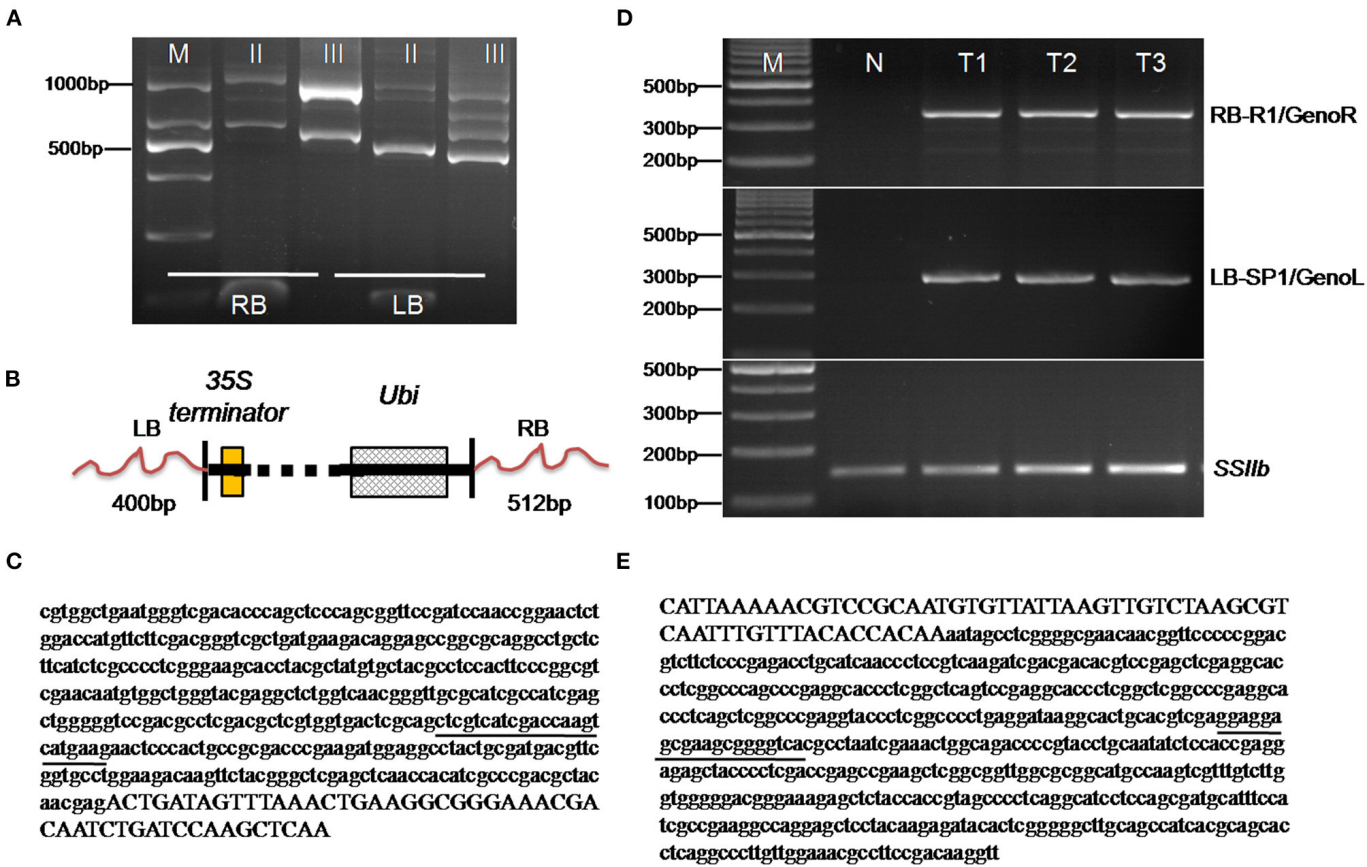
Isolation and identification of the flanking sequences of SK 12-5. **(A)** The analysis of thermal asymmetric interlaced polymerase chain reaction (TAIL-PCR) products from the SK12-5 insertion line. M denotes the 1,000-bp DNA ladder, while lanes II and III indicate the products of the secondary and tertiary reactions, respectively; RB and LB indicate the products of right border and left border, respectively. **(B)** Schematic diagrams of the integrated heterologous DNAs in SK12-5. **(C,E)** Sanger sequencing of the amplified thermal asymmetric interlaced polymerase chain reaction (TAIL-PCR) products in the LB and the RB of SK12-5, respectively. The sequence of the maize genome at both LB and RB is denoted by lowercase letters and that of T-DNA by capital letters; underlined are the primers of GenoL and GenoR on the maize genome. **(D)** Validation of the LB and RB flanking sequences from SK12-5. M, the 1,000-bp DNA ladder; N, non-transgenic maize line Zheng58 as the negative control; and T1–T3, the three samples of SK12-5.

The extension of the T-DNA flanking DNA sequence in the transgenic maize SK12-5 was done by using the TAIL-PCR method (Liu and Chen, 2007). The left border (LB) flanking DNA sequence was used with three nested specific primers (i.e., LB-1, LB-2, and LB-3) combined with a degenerate primer (AP; Table 1). The amplification products from the secondary to tertiary PCR were analyzed simultaneously by using agarose gel electrophoresis. Accordingly, the amplicon size was reduced in successive PCRs (Figure 3A). The amplification products were isolated from the gel and were ligated into the pMD18-T vector for sequencing. A Basic Local Alignment Search Tool (BLAST) sequence alignment (http://www.ncbi.nlm.nih.gov) was used to analyze the sequence; this revealed that one 450-bp fragment encompassing the LB junction region was obtained, of which 400 bp originated from the maize genomic DNA and 50 bp originated from the T-DNA vector. Also, among three nested specific primers (i.e., right border RB-1, RB-2, and RB-3) and a degenerate primer (AP, Table 1), one 573-bp fragment encompassing the RB junction region was obtained, of which 512 and 61 bp originated from the maize genomic DNA and the T-DNA vector, respectively (Figures 3B–E).

**Table 1:** List of the primers used in this study.

**Primer name**	**Primer sequence (5′-3′)**
*zSSIIb*-F	5′-CTCCCAATCCTTTGACATCTGC-3′
*zSSIIb*-R	5′-TCGATTTCTCTCTTGGTGACAGG-3′
*G10evo*-F	5′-TCACCTCTGGCACCACTTTCG-3′
*G10evo*-R	5′-CGGCGTCGGTGAAGGAAT-3′
*Cry1Ab/Cry2Aj*-F	5′-TTCACCACCCCCTTCAACTTC-3′
*Cry1Ab/Cry2Aj*-R	5′-TTCCACTCGGTCCACTCCTTC-3′
LB-1	5′-GTCGTTTCCCGCCTTCAGTT-3′
LB-2	5′-CGCAGCCTGAATGGCGAATG-3′'
LB-3	5′-GGAAAACCCTGGCGTTACCC-3′
RB-1	5′-TTTCTCCATAATAATGTGTG-3′
RB-2	5′-TGAGTAGTTCCCAGATAAGG-3′
RB-3	5′-TGTAAAATACTTCTATCAAT-3′
AP1	5′-ACGATGGACTCCAGAGGCGGCCGC(G/C/A)N(G/C/A)NNNGGAA
AP2	5′-ACGATGGACTCCAGAGGCGGCCGC(G/C/T)N(G/C/T)NNNGGTT
AP3	5′-ACGATGGACTCCAGAGGCGGCCGC(G/C/A)(G/C/A)N(G/C/A)NNNCCAA
AP4	5′-ACGATGGACTCCAGAGGCGGCCGC(G/C/T)(G/C/T)N(G/C/T)NNNCGGT
LB-SP1	5′-CGTCGTTTTACAACGTCGTGACTGG-3′
GenoL	5′-CTCGTCATCGACCAAGTCATGAAG-3′
RB-R1	5′-TCAGATTGTCGTTTCCCGCC-3′
GenoR	5′-TGACCCCGCTTCGCTCCTCC-3′

*LB, left border; RB, right border*.

### Identification of Possible Insertion Sites by the Next-Generation Illumina-Based Sequencing Technology

The next-generation Illumina-based sequencing technology is a powerful tool for identifying the insertion positions of GMOs (Polko et al., 2012; Lepage et al., 2013; Yang et al., 2013; Guo et al., 2016; and Siddique et al., 2019). In this study, the technology was utilized to identify the insertion of GM maize SK12-5. The sequencing libraries were constructed, for which sequencing reads of 150 bp in length were generated *via* paired-end sequencing. After the quality control checks and processing, 2,103.6 million clean reads were acquired for SK12-5. Out of these sequencing data, 87.77% had Phred-like quality scores >Q30, indicative of their high quality (Table 2). About 99.58% reads could be mapped onto the reference genome of maize, accounting for approximately 40.81× coverage of it. All clean reads were mapped onto the sequences of the maize genome and vector. Then, the reads for which one end was mapped onto the vector and the other end to the maize genome were selected. Although several sequencing reads were consistent with the TAIL-PCR results, the insertion position could not yet be determined because the next-generation Illumina-based sequencing reads are too short to be specific.

**Table 2:** Summary of sequence data from next-generation sequencing (NGS).

**Parameters**	**Quality**
Clean reads (M)	2,103.6
Q30 (%)	87.77
Average depth	40.81X
Mapped ratio (%)	99.58

To further confirm the junction sequences obtained from the TAIL-PCR and next-generation sequencing (NGS), event-specific primers were designed based on the RB and LB insert-to-plant junction events in SK12-5 (i.e., one primer on the maize genome and another on the vector sequences). Using the RB primers, RB-R1/GenoR and LB primers LB-SP1/GenoL (Table 1), only SK12-5 was amplified to 350- and 290-bp bands, respectively, whereas the non-transgenic Z58 failed to amplify (Figure 3D); this indicated that the sequences from the TAIL-PCR and NGS results are indeed correct and these primer pairs are sufficiently specific for the detection of SK12-5. Although the flanking sequences of LB and RB were successfully assembled, both being in the maize genome, the exact location of the insertion site remained unknown. The BLAST analysis showed that the flanking sequences of LB and RB were unspecific, with 257 high-confidence positions having high similarity (i.e., >90%) being found.

### Bioinformatic Analysis of the Flanking Sequence of Transgenic Maize SK12-5

**Figure 4:**
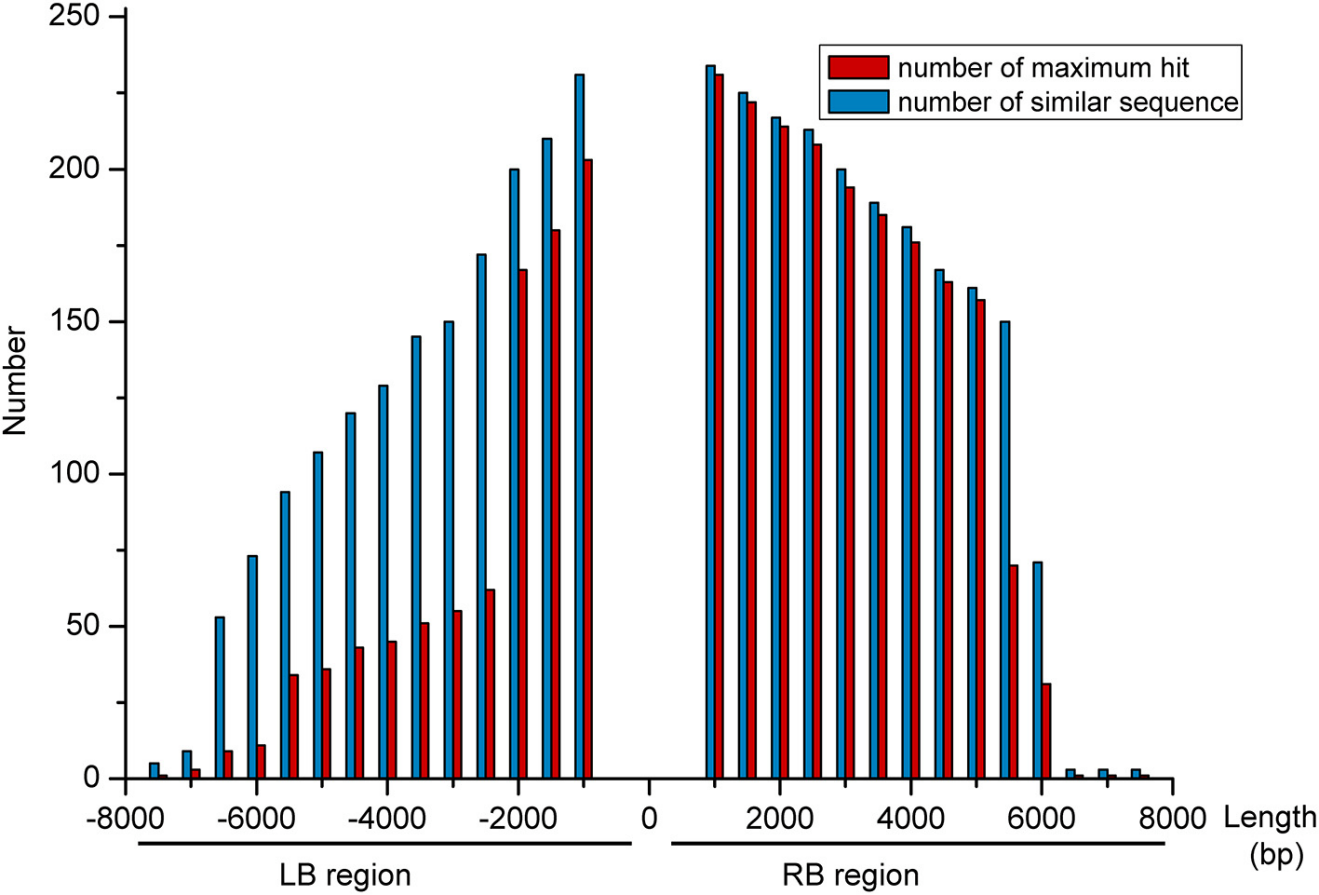
Comparing the extended sequence from similarity of 257 sites at different lengths, separately. The *x*-axis shows the different lengths (0.5–7.5 Kb, step length of 0.5 Kb) extended at the left border (LB) and right border (RB) regions, respectively. The number is denoted on the *y*-axis.

We further analyzed the sequencing results from the TAIL-PCR and NGS by downloading the maize genome sequence (B73, http://plants.ensembl.org/index.html) and then aligning the flanking genomic DNA sequence to the maize genome using BLAST (Liu and Chen, 2007). After filtering out those positions containing a long gap (>100 bp) from the total (320 bp), this left 257 high-confidence positions characterized by high similarity; all of them were located in a repetitive sequence area, according to the comparison with maize repeat annotations performed using the BEDtools (Quinlan, 2014). To determine the specificity of flanking sequences at the insert position, different lengths (0.5–7.5 Kb, step length of 0.5 Kb) were extended at the LB and RB regions, respectively. After extracting the extended sequences from the maize genome, we used BLASTN to compare the similarity of these 257 sequences at different lengths separately. Figure 4 shows that the LB and RB regions had a similar trend as follows: as the length increased, the number of similar sequences decreased. At an LB of 3,000 bp, there were a total of 150 similar sequences, and the maximum hit number of these sequences was 55. When the length increased to 7,500 bp, there were only five similar sequences, and the maximum hit number was one (Figure 4). From these results, we speculated that the LB region extended over 7,000 bp and had a high-specific sequence, while the RB must be at least 6,500 bp. Therefore, it is difficult to determine the exact insert position on the maize genome at a fine scale from the TAIL-PCR and NGS sequencing methods.

### Mapping the Exogenous Fragment of Transgenic Maize SK12-5

To refine the insert position of the exogenous genes on the genome of transgenic maize SK12-5, another method must be used. For this, an F_2_ mapping population segregating for the exogenous fragment was developed, by crossing SK12-5 with non-transgenic Chang7-2 (C7). We then identified the transgenic and non-transgenic individuals from the F_2_ population by using the same primers for detecting exogenous *Cry1Ab/Cry2Aj* (Table 1; Supplementary Figure 1). Out of 353 F_2_ plants from the SK12-5 × C7 cross, 266 were transgenic and 87 were non-transgenic. The segregation ratio in the F_2_ population closely followed a 3:1 ratio (transgenic: non-transgenic), confirmed by a chi-square test ($\chi_{\left[3:1\right]}^{2}$ = 0.008 < $\chi_{0.05}^{2}$ = 3.84), which indicated that the transgenic maize SK12-5 was a single-copy event.

**Figure 5:**
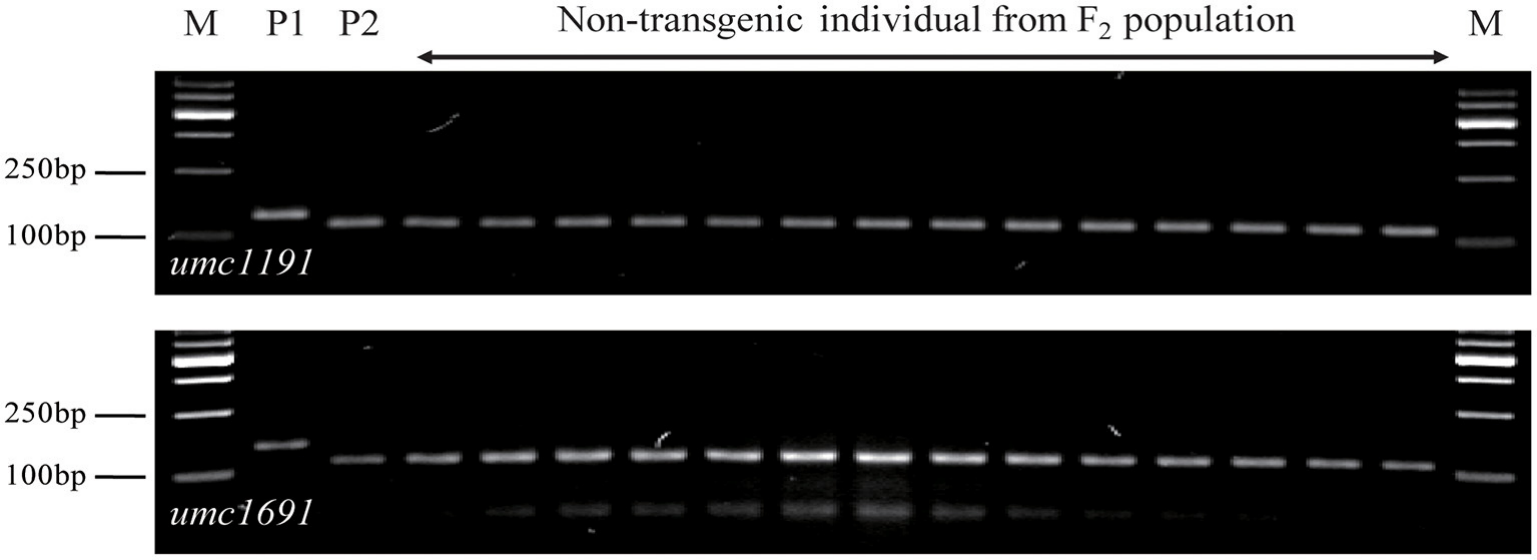
Segregation pattern of *umc1191* and *umc1691* in non-transgenic individuals from the F_2_ population of the SK12-5 × C7 cross; P1 for SK12-5 and P2 for C7. M, 1,000-bp DNA ladder.

**Figure 6:**
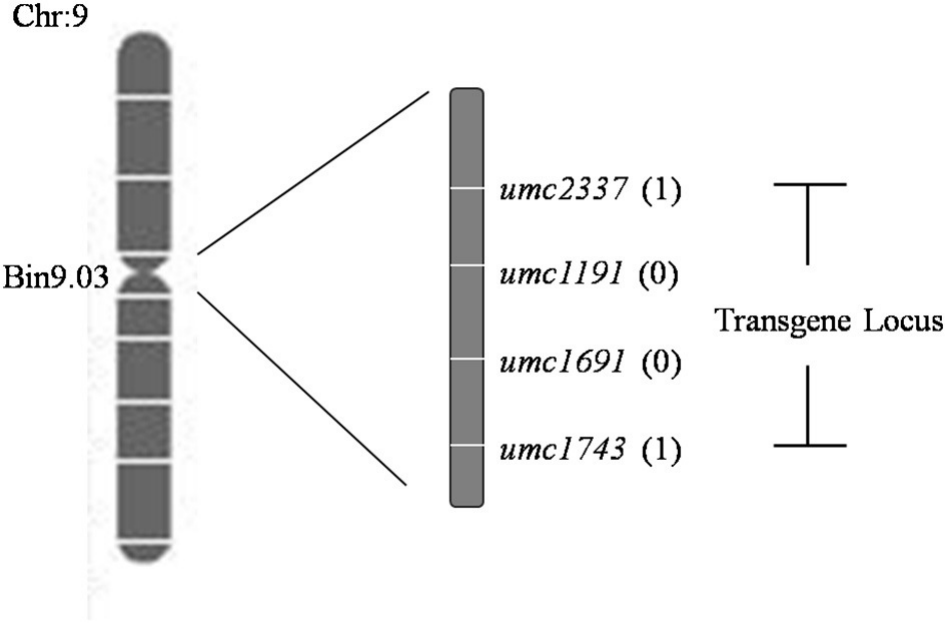
Deduced locations of the simple sequence repeat (SSR) markers and transgenes. The name of each marker is listed on the right side of the map. The number of recombinants is given after each corresponding marker.

A total set of 283 SSR markers with a uniform distribution on the maize genome were used to screen among parents and non-transgenic individuals of the F_2_ population. Among 93 polymorphic SSR markers across the population, the exogenous fragments were linked with *umc1191* and *umc1691* using a whole-genome scan and linkage analysis (Figure 5; Michelmore et al., 1991; Zhang et al., 2015). The positions of these SSR markers and the linkage analysis delineated the region of exogenous fragments as lying between the SSR markers *umc2337* and *umc1743* (Chr9: 26,822,048–100,724,531 bp), and the size of this region 73,902,483 bp (http://www.maizegdb.org/locus_pair_lookup; Figure 6).

### Nanopore-Based Sequencing for Identifying the Insertion Sites of Transgenic Maize SK12-5

**Figure 7:**
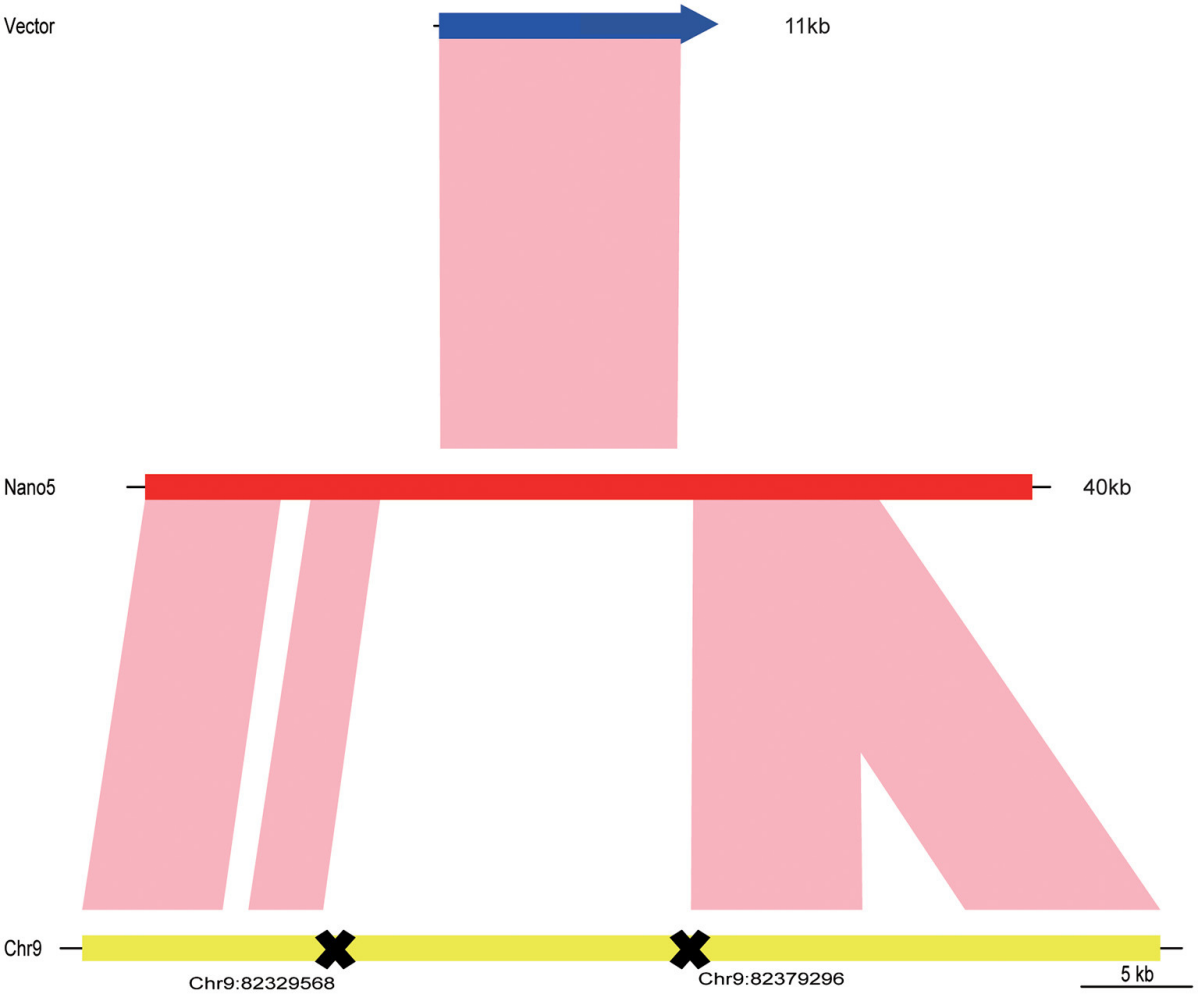
The three sequences for the vector, nano5 read, and maize genome were compared and drawn using the R package “genoPlotR” (Guy et al., 2010).

The long-read nanopore technology was then applied to further analyze the insertion sites of transgenic maize SK12-5. After completing the quality control, a total of 1,062,647 reads were obtained, whose mean reading length was 23,169.6 bp, with an N50 of 42,357 bp. About 99.08% reads could be mapped onto the maize reference genome, accounting for ~11.22× coverage of it (Table 3). After a detailed data analysis, 10 reference-vector-mixed reads (named here as “nano1–10”) were isolated from the sequence data of SK12-5. All 10 junk reads covered the vector, ranging from 23 to 51 kb in length (Supplementary Data 1). The sequence data (i.e., nano5 sequences with the highest BLAST score; Supplementary Data 2) and the BLAST comparison sequences were drawn with the R package “genoPlotR” (Figure 7) (Guy et al., 2010). By combining the genetic mapping and nanopore-based sequencing results, the insertion position of SK12-5 was putatively integrated to the position of chromosome 9 from 82,329,568 to 82,379,296 bp.

**Table 3:** Summary of sequence data from the nanopore sequencing.

**Parameters**	**Quality**
Active channels	2,650
Mean read length	23,169.6
Mean read quality	6.9
Median read length	18,693.0
Median read quality	7.8
Clean reads	1.063
Read length N50	42,357
Average depth	11.22X
Mapped ratio (%)	99.08

## Discussion

The insertion position of the exogenous gene fragment in GMOs at the chromosome level is important for their safety assessment. At present, two methods are mainly used to determine the insertion position of the exogenous fragment. The first method is based on the TAIL-PCR, combined with standard sequencing of the functional (intended) insert(s) and flanking genomic DNA of the host; through the latter analysis, the insertion position can be obtained. For example, the TT51-1 event was the first food crop approved for commercial use in China in 2009 (Tu et al., 2000, and Lu, 2010). Based on the TAIL-PCR and sequencing, Cao et al. (2011) demonstrated that the transgenes in TT51-1 were located on the rice chromosome 10. As the TAIL-PCR is simple and efficient, and uses sensitive reaction products with high specificity, the target fragments can be quickly obtained (Qiu et al., 2008) and the method has been widely adopted. The second method for studying the insertion position is the NGS technology. The benefit of the NGS platform is the depth of its sequencing capacity and low labor costs (Polko et al., 2012; Lepage et al., 2013; Yang et al., 2013; Guo et al., 2016; and Siddique et al., 2019). In this study, we first tried to analyze the insertion position of transgenic maize SK12-5 by applying both the methods, that is, the TAIL-PCR and next-generation Illumina sequencing technology. Although the junctions of transgene–host LB and RB integration were elucidated, we failed to discern the insert position because the flanking sequences in the maize genome are unspecific. Thus, by using either method, it remains difficult to determine the insertion position when the exogenous fragments of interest have been inserted into the LTP region of a large and complex genome. To our best knowledge, an insertion position in the LTP region of GM crops has yet to be reported.

Bulked-segregant analysis (BSA), initially developed by Michelmore et al. (1991), is a genetic mapping technique that is useful for the rapid identification of molecular markers or mutations linked to a specific gene or genomic region (Michelmore et al., 1991). In this study, we used this approach to identify the insertion position of the exogenous fragment sequence in GM maize. We implemented a classical BSA approach to map the region of the exogenous fragment. To do this, first, an F_2_ segregating population between GMOs and non-GMOs should be constructed. Second, it is necessary to select non-transgenic individuals from the population. Both of them were easy to do using the PCR validation of the function of the given exogenous gene; for example, a herbicide treatment can be used to screen out non-transgenic individuals if the GMOs contain herbicide-resistance genes. Additionally, it is necessary to determine the proportions of transgenic and non-transgenic individuals, which provides helpful data for confirming whether the transgenic event is a single or multicopy insertion. Finally, an SSR-based linkage analysis of the insertion position using non-transgenic individuals from the mapped population is performed, and the mapping region is determined. Accordingly, in this study, we used this above approach to analyze the insertion position in SK12-5. While limited by the number of mapping population, the transgenes of SK12-5 were nonetheless mapped onto Bin9.03 of chromosome 9 between the SSR markers *umc2337* and *umc1743* (26,822,048–100,724,531 bp), a region that was still large.

Recently, the nanopore-based sequencing technology has been developed to identify the insertion positions of transgenes (Li et al., 2019, and Suzuki et al., 2020). Unlike the NGS, however, nanopore sequencing is based on long reads (Jain et al., 2018). In our study, the mean read length was about 23 kb (Table 3), with the longest reads reaching 51 kb (Supplementary Data 1), which is sufficient to cover the whole vector sequences and longer flanking sequences. Furthermore, the MinION Nanopore sequencing device is a lightweight (<100 g) portable apparatus, compatible with a PC desktop or a laptop, thus providing flexibility that allows for its use in the field outside laboratory settings (Castro-Wallace et al., 2017). In our study, we relied on the nanopore-based sequencing technology to discover the final insertion position. An earlier study showed that the nanopore reads usually have a broad error distribution and harbor subsequences at a high error rate (Chen et al., 2021). Thus, blasting the genome using error-prone reads will make the genome comparison less effective, especially in the complicated genome regions (Koren et al., 2017, and Jayakumar and Sakakibara, 2019). In consistent with this view, when compared with the maize genome, the nanopore reads did have some unaligned fragments (Figure 7). These may have been caused by the error sequence data produced by the nanopore sequencing. Oxford Nanopore Technologies recently released a R9 flow cell that can generate reads up to 1 M in length and with a read N50 value >100 kb, which may significantly improve the contiguity of assembly (Jain et al., 2018, and Kuderna et al., 2019). Taken together, a long-read sequencer will be a powerful tool for determining the location of the insertion of a transgene on the host genome in most instances. In this study, by exploiting the advantages of the nanopore technology, we were able to use the whole vector sequence for the comparative analysis to investigate the exogenous fragments inserted into the LTP region of the maize genome. Notably, we did not find any multicopy, rearrangements, the presence of backbone sequences, or truncations in the maize genome. Although the nanopore-based sequencing technology help us acquire long sequencing reads, the error rate of nanopore reads is about 5–15% (Chen et al., 2021), which remains a formidable challenge for bioinformatics and for accurately determining the insertion position. However, when coupled to a linkage analysis, this may help us reliably infer the region of a given insertion position, by eliminating interference from other regions of the genome. We concluded that an approach of combined methods can yield more genomic information and strengthen the final results, and so we anticipated that this combined method (as exemplified by this study) will find broad applications.

## Data Availability Statement

The original contributions presented in the study are publicly available. This data can be found here: NCBI [Sequence Read Archive (SRA): SRR14289756 and SRR14289757].

## Author Contributions

CP and JX performed the experiments. LD, YM, and XW analyzed the data. JW and XC wrote the manuscript. CP read the manuscript and provided helpful suggestions to improve it. All authors contributed to the article and approved the submitted version.

## Conflict of Interest

The authors declare that the research was conducted in the absence of any commercial or financial relationships that could be construed as a potential conflict of interest.

## Publisher's Note

All claims expressed in this article are solely those of the authors and do not necessarily represent those of their affiliated organizations, or those of the publisher, the editors and the reviewers. Any product that may be evaluated in this article, or claim that may be made by its manufacturer, is not guaranteed or endorsed by the publisher.

## Supplementary Material

Supplementary Figure 1 Identifying the transgenic and non-transgenic individuals from the F_2_ population *via* polymerase chain reaction (PCR).

Supplementary Data 1 The sequences of 10 reads from the nanopore sequencing, with one end mapped onto the vector and the other end to the maize genome.

Supplementary Data 2 Nucleotide Basic Local Alignment Search Tool (BLAST) results for the transgenic sequences *vis-à-vis* the 10 reads.
