# Supplementary figures and images for: Using Combined Methods of Genetic Mapping and Nanopore-Based Sequencing Technology to Analyze the Insertion Positions of G10evo-EPSPS and Cry1Ab/Cry2Aj Transgenes in Maize
Source: Front Plant Sci. 2021 Jul 29;12:690951. doi: 10.3389/fpls.2021.690951 (PMC8358107; doi:10.3389/fpls.2021.690951)

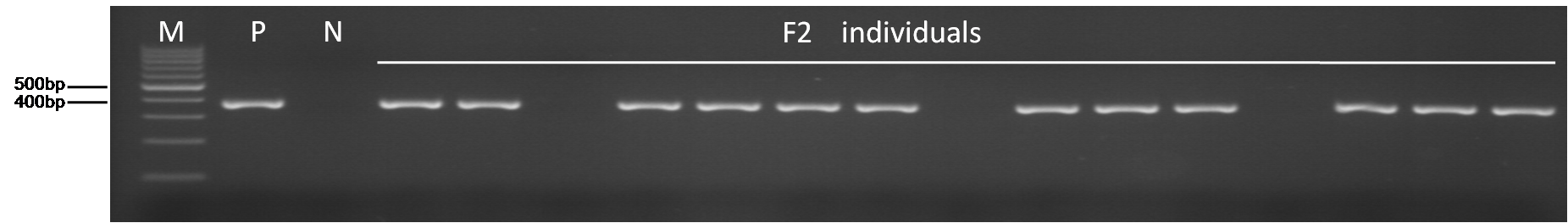

Supplement: Supplementary Figure 1 — Identifying the transgenic and non-transgenic individuals from the F2 population via polymerase chain reaction (PCR). [file Image_1.TIF]
