# Supplementary material for: Using Combined Methods of Genetic Mapping and Nanopore-Based Sequencing Technology to Analyze the Insertion Positions of G10evo-EPSPS and Cry1Ab/Cry2Aj Transgenes in Maize
Source: Front Plant Sci. 2021 Jul 29;12:690951. doi: 10.3389/fpls.2021.690951 (PMC8358107; doi:10.3389/fpls.2021.690951)
Supplement: Supplementary Data 1 — The sequences of 10 reads from the nanopore sequencing, with one end mapped onto the vector and the other end to the maize genome. [file Data_Sheet_1.docx]

>nano1

CAAAGCTGAGGTAACATAGGCTCCCATCAAAATTAACCTATGCAGATCATTAAAATTAATAAGAACATGGCTGGGAAAAGTAAGTGATCAAGGGCACAACTTGTCGGCATGAGAATCCGGTATCAACTTGCTGGGATGACACATGTCCTCGCTAATCGTAGCAATACAAACAAACATGGTATAGATAAAATTAACATCACACCAAACATAAGAATAAGTTTCGTAATAATAATCTACGCATTGCTCTGAGGCTAGCGCTGGCAGGCGGATCAATTGAGTTAAAAGCAGTTAGTTATGATTTTCGAAGTGCAGCAGATGTTTGAGTGGGAATAGCGATAAATTTTAATCTAGTTGTCATGCTAAACGAGTTACCAATTGATAAACATTATTAATATGAATTTTATGCAACTAGAATGGATCCATTTGGGCTATAATGAATTTAATATGATTTATCAAGTTTATGCAGAATTGTTTTTATACTAAAAATCTATTTTATTTATTATTTCTGAATTTATTTATTTGATTTTCATGAGCTGCTTGGGTGGGTCATTTTCAGGAAAGTGAGGTCTTAGCGCAAGATTGCTAAGACTCAGTGCACTCCGCAGTGAACTAGGGTTAAATATTTGATTATCACGAGGCTCATACATAAAAGAGTTGTCAGAAGGTATTGTTGGTATAGACCACCCGGTCGAAGCGTCGCGGTGGATTTTAATGTGCCGAAGGTGTCGCCGCGATCTAATCCTATCCATCAACTACCGATCGGCGGTCGATTAGAAGTTTTCGCCCACGAACCGATATCAACTCGACCATCGATTACGATCTAGCAGCGCGCGATAAAGGGGTGAAAGGAATCGCCAGCTCTGTCTGGGCACGCGTTCACGCATCAAGAGGCCATCCGCTATCTTCCTCCACTCGGCCATGGACGCGGTGAGCTCCGCGACGACCAGCGGCCGCGCCACCGCGAAACAACCATCCTTGCACAAGGGTTCTAACCTTTTGATCTAATAGCACTACTCGTCACGGTGATAATGGTGCTCAATTGGCGGGACTCACCAATGAGCGGCGGGGCGCAGCCGCCACGCTAAGGTGCGGATCCGGCGGCGGCAGTCAGCGTGAGTAATTCAAGAACACCGCCCGGCGCGCGCGGGAAGTTCACGGTGGAGCGTACACCCGGCGGTCGGCGGCGGAGAGGGCGAGAGAGGAGGCGTTAACGATGGCAGAACCCCTCTGGCGCGGCGTGGGGTCGAGCAGTGCGCGCTGACGCGGAACGCGCGTCGGCTGGAGCAGCGAGCATACGTCTGATCAATACACAAGCGCCACGGCTGGGTGGTAGAACTTGTCTGGCGCTGCTTCCCTTCGCCTTGCCCATCTCTGCGATGCGCCGACGAGCAGCGAAGCTGCGAGCGGCAATGGCGTTTGCGGCGGGATCTCGGCGGCGGCGGAATCTGGCGAACTTCACGAGCTCTACCACCGCATCAGTAGTTAGCGGTAGCGAAGGTTCGCGATGACAACGATGCTCGCTACCACACATCTCCAACCTCTTGTTTGAATCTGCACCTCTCCATGTACTTGGTGCAATGGTGGTGGTAGCTGCGCCAGATCTCCCATTTCTCTACCTCTCTCACGGCGGCACAGAAGCAGCGTGGCCGGGGTATCTGAGGCGTGAGGGCCCGCGGCGAGTTTTATGGCTAGAGCCACGCCGCACGCATTCGATTTTTACGGCCGGTGAAATTTCCTCGGTTGACTTCGGGCCCGGGGGAGCGAAGCGGCGTGTTGAGGAGGGCGAAAGCGGTGACAAGGGCTTGCGAGTTTAGGCAGCCGTCTGTGGCGGGCTGAAGTCCCAGGGTCCCGCGGCGGCAAGTGCGCACCCGCCCAATCTAAGCGAGGAGATGAACAGGAGGCTCGCGGCTGCGGTGGGGCGCGAAGAGGGTCACGCTGGCGAGGTGTTGCGCGGGATAAAGTTGGAAATGGGCATGGTGAAAGCAGCCCAAGCGTGGCGGACCTTACTTCTTTCTTTTTTATATATTTTTTCACTTCTCCTTTTCTTCTTTTCCCAATTCAATTTCCACTTCAGATTTAAATTCCCATCTTGTGTCGAGTTGCCCTAAATCAAAGGCATGAATTTGAACATACTCAAAATGATCTGAATTTGTGTATTTATAAATTTTATTTTGGTTTTGTATAATATTATTCTTTCCTTCTACATTTTCAACCCAAATCTAAATTTAGGTTTTTACTTCAATCCTCTTCTCATTATTATTATATTTTAATTCATATTATTGTTATTCTTATTAAATTACACAAACAAACAAAACTCATCATGATGCGTGATTTAATGGCATGTCCTTTATTAAGTATTTTTTTAATGAGTTATTCACATGAAACAATAAATAGGGACAACACACATATATATAAAGGAATATATTTTCTCCTTTTAGACTTTTCTTATAAAGTGGGTATTACATCAATCAACAAGTTATCTCATGTAGTCCATTTCACTTCTTACCGATGCAGTACAAGGATCAAGCGATCTCATTATTTGCGAAAGCGAGCGATTGAATCGAGTTTTAAACCTTGCAAGGTAAACCTAAACTACGGCATGTCGGGGTACTCGACCCGCATGTAACAACCGTCCCCATCGATTCCCGTTAACGTCGGGCCTCACCGCCTTGGCATACAATGCTCCACTGACCCGCTGCCGCCCGTGCGGTGGCCACCTTGTACCCACCATAGCTAGCATGGGAGACCGGTCTCAGTCGCATGAGGATAAAGTCCGTGCGGCTTCACTCAGTACTAGGTTTACCAGTTACCATTTTTCCGACATGTAGTAAATGCGTTCAAAGCTTGACTCGGTATCACACATTAATCCTTAATTCATTTTTCCGTATTATCATGGACAAGGCATCCTCCTGGATCCCAATCCATAGACCAACATAGATCCCGTTATCAAGATGAATACAATCAATTCGACCTCGCGCGAGTGCTAGAAAAATCACTGCGACTTCTCAGAGATCTGATTGGCGGCTACTGCGACCTAGCATACTAGTATTCATCTCAAAGAGATCCTATGTTCATGCAACTCCTACACTTAAGTGCACATTGCAATCCTATAAGCATTAAGTGTAGTAAAGTAGCATATAATAACATGGTTATGCCATAAAACCGGGGCTTCCTTCATTGCTTTGAAATTGGAGATCCTAATAGCGACCCTCGACACCGCTCTGGTCCTCCTCTTGGACAGTCCTTGCTCGGGGATGAGCGCATTACTCTCGTCGGCAAGATTACAATCTAATAGAATGCAATGCGTAAGATATATGCATGATATGATATGTGCTTTTAGAATTACAACTTTTAAGGTGTGTAGGATCTTTTGAGTTTAAAACAAGTTAACTTTACTTATAATAAAACCCTTAGTGGTATACTTGGTAAATTGGGTTAGACTTAGCTAAGGTAAGTGGTAGGTTTATTTTTTGGGGTTCCACTGAATTCCTTTTAAGTCTTAGGGATAAATGATAAGTTCTCAATTAGACTTATTGGGATGAGGTTTATTCCTTCTTCTCTTTTCTTTTATTCTCTTTAATGTTTTGGGTAAGTTTGAACTACAAGTTGCTTATATAAAATTCTAAAAATTCCACAAAAATTACAGTGGCTTGGTATATTTCCCTGTCTCAAAATTTGGGGGTCAGCAAGTGAATAGATTTTCTCTGGACAAAATTATTAACTCTACGGGAGCAGAAGGGTGCTTTGAACTACAACTACTATTTAACAGTGGGTAATTCTCTAAAACTTATTTTGCTGGCTTTTAGGTGTTATAACATGACTTGATACAAATTTCTAATCATTAATACTTCTTAATAAATTTTCTATGGTTTTTCTCAAGTTTCTAGCCAAATGGGTGCTTTCTCTACCACTATATTTGAAAAATATCAAACAACAGTTCTTATTTTCATAGTTAGTATTTTGTGCAAGAGCAATCATTCAAGTTTGGCTTCTTTTTGCTTAAGGGAAGGGTGGTTTGCATTATTTGAATTAAGTGGTCTTTCTCTTTTATTAGCAAAAGGTATGGGTTTACTTCTTCTTCATGGGTTTGCATTTTTCTCTGGTAGTTTACCTCCTCATGAGCTTAGCAATTTTTGGTTGCCTATTATCACATTAAAAGGTTGTTCATGATTTATTTGGAAAATGTCTTATTATCACCTTGTATTTATTTTCTTTACTTAAAAAGTTGGTGAGGAGTGCTCTGTATTTTTTGTAGTGGGGCTCTAGTGGTTATAAGTCTACTAGATTTTTATTAATCATTTTGGTTATAGTTTTGTAACTCTAATAATTGATTTTGTCTATATGAGGCTAAACAAATCATCTTAATTTAAAAACTAGTCCAAATTAATGGTCTCTGCATTTTCCTAGGTTCTGACATAAGTAAACTAGAAAAATATTATTAATCAACATTCATTAATTTCTAAGGCCTTTGATTTTCTCTAAGTTTTGGACAAATTAGCTTTAAATGAAAACTACATCATATTTTTTCAATGCTGGGGTTCCCTACTATTTTAAATAGTGTCTAAATAAGGTTTAAACATCTACAATTTTTCTTAAGTTCACCTCGAGGAAAGAAAACTAATTTCCTTAATTAAACAAGGTTTAGTAGGTTTACATTTTAATTTTAAACTTTAAAATTTTGAGCAAGAAAACATGGAGTTCTATTTTTAAATGATAGATCATAATATTGAGCTAGCAAAATTGGTTTGACAACTTTTATTAAGATTTCATCAAGTTATGGGTTTTCTAAGTTCTCTGGTCATTTAAAATAAAATTGATTAAATAAATCCACTTTGCCGGGGGTCCTGGCGATTTTATAAGTTTCCTCGCTTGAATCAGTCAGCAGGTTACTACTCACATGAGTCAGCGACATTCAGAAAACCCTCGGGTTCTACAATCTAACCGAGGTCCTTCTTCTACCTTAAGCGATGCGTGGCGGAGAAAAGGCGGAGGGCTTCTGTGGCCGAGTTGCTCGGTGGTAGTCGGGGCGAAGGGAGGTCTCGGGATCTAGCGGTGTGCGGGCGCCCGTCAGATGGTCGTGTTCGGTCCACCGTGCGGCGGGATGCTGCGTCGGCGGCGAGAGACCCGGCCTTGTCCGGCGAGATAGTTCAATTAGATGGGTCGGGAGAGTTCACGGGATGCCGAAGAGATATGGCCCGAAAGGGGTGGCGGAGACTCAGCTGGATAGCTCGGTCCTGCGGCGGCGGGAGCCGAAGTCCGGTGAGTCAGTCTCGAACCTCCGTGAAATCCGGCGGTCAGGAAGCGACAAGCTTCACGGGCTCTTTGCGAGCTAAGCGAAGCCTTGGCAGGCTTGAGGTGGGCCCAGGTGGGTTAGCCCGCGATGGCAGCTCTACGCAATGGCGGCGGCGGTCGCTCGCCGGGCTAAGGAAGGTGGTGGCGAGGAGAGGGTGAGTCGCGAGGCGAAGAGAGTGCGCCGGAGGCTATAAGCGGCAGGCGAGCGCGGTAGGCGTGGCGTGTGGCCGGACTTGACCGGATGCGAGGTGGCCAGCGCGCGGTTAGGCCGAGCTGGCGTCGCAGGCAGTCAAACTGCGCGCGTGTGCCGTGCGTTACGCCCAAGTTGGCCAGTGGTGGCTCATCCGAGAGCTGCTCGCCTTAATGGATGCACAAAACCTCTTCTCCTCCTACAAGCTACCATTCTTGTGTGGGGTCGCAGGATTTTGCCCTGCGGTTGCGAGATATGGAGCCGGGAAATCTGGTGCATCTCCACTGCCCAAACAGAGGCAAATCAAGGTTTTATGGTGTCTAGGCTCGCGTCCCAATGCCATCTTCGGCCTGCACGAGAGGGGTTAGTTAGACACAATTTTGTCAATGGGGCCATTAGGATTGAGTTGGGATCAAGGTGAACACCTGATCTTTGGCTCAGGTCTGAATTTAGAATCCTGAAATTCGGAAATCCCAATAGTCCCCACATGAGAAGCTTGATTTGGGGTTTTATTTGAGTTATTTTGGCTAAGCTTTCTCAGCATTTCTTGTTGCCCATTAAATATTCTTTAATTTTCATAATTGGCTCAAGTCATAATTTTAAACTTTTCATTCCCTTTTTCTTATTTTCTTGAATTTTGCTCATGGGGCTCACTTAGGGTTCTTAATTAGGGTTGCACATTTTTATCTTTTCAAAGACTCAATTGTTTTGATCATGTTACTTTTAAGTATTTACTTGGTGAATTCTTTATTACTTAAGTTGTTTTGATGCTATGCTTACTGGTTCACATAAAATGATGGTTCTTAGTTTGGCATTTGAGAAAACCACGAGTGACAGCGGGGGTGTCTGACCTTCCCCCTTAAGGAATCTCGTCCGAGATTCCGGGCGAGTCCTCGAGTGAAGCGAAGGGTGAGGTGATAGGAAAAGGGTGATGATTATTATTATTGGCAGCGCTCTTGAATGTCATTCTCTTTGCAACTTGGAAGGAAGTCCTTTTAAGTTTTACTTTATAGTTTCTTCATTACGAATTGAGATCATGTTTTTGGAAATTAGATTGAAAGGCGAGAAGGAGGAGGGTTGGGGGTGATTACGTACCCGGCTTCCTTAGGTAGATGTCGGGAAAGTTGGATCTCGGGAATTCTCGGTTTCCCAAGTAACCTCCTCACGAGTGATTACTCCACTGGACCTTGAACATCTTGATCGATTTAGCCAGGTTGATCTTTCCTTGCAATCCAAAAAATCTTGGGGTACACTTGATACGATAGATCGCTCTATCTCCAATTCAGTTACCTTGCTATGATCTCGGTCGACAATCGACACACTTCTTGGTGAGATACATGGAATACTTGTGAATGGCGGACATCTTTGAAGGTAACTTCCAGCTTATGCCACAGGTCCACATGCTTGATGATCTCATAGGTCCAATGTAACGAGGGCTAGCTTGCCTTTGATCCCAAACCTCGCACTCCTTGGTGGGTGATACCTTGAGGTATCTGAAACTCCCACCTCAGGCCCGGAGGGTTTCCTTCTTCTATCATGATAGCTCTTTTTCCTGGCCTACAGGCGGCTTCTAGATTCTTCACGATTAAGCTTGACCTTCCTTTCGGCTTTAGTCACCGTCGGGTCAAAAACTCTCTTTCACCGAGTTGAGACCAATTGGTGGTGTCACCTTCTTCCATAGAAGCTTCAAAGGTGCCATCTTTAGGTTTGGATTGATAACTATTGTTATAAGCAAGCTCGCCAAGGAGAGGTGCTTATCCAGTTCTTGCCACAATCGATCGCACAAGCTCTCGGCATATCCTCCGGAATCTGTTTACCCTTTAAATCGACCATCGATTACAGTGGGTGGTAAAAGCTGAACTCTGAGATTTGGTTCCCCAAAGACTCTTGCAATTGTTCCCAAATCTGGCAACAAATTGGGCTCCTGGATCCGAAACAATGGTCCGAGGTAATCCATGCAAACACGATCAGTCAATATACAATTATGCATATTTACAGGCCTTATCGTTGGTGTGCCACGAAGAGAAAATGTGCCACTTCGTCAATCGGTCCACAATAACCCAAATTGAATCATGATGTGAAGGTGTTGGGCAGACCACAATGAAATCCATCTTGATGTCGTCCCACTTCCACGAAGAGTGCAGACGGTGGTTGCAAAGCTCAGCGGACTTCAAGTAGCTTGCCTTTATCCTCGACAGTGTCACACTCTGATACATCTTGGGCTATCTCCCTCTTCATTACGGTCCACACCAGTACAACTTCAAATCATGGTACATCTTGGTGCTTCCGGATGCAAGAATTTGGAGAGATGAGCCTCATCCAAAATTTTCCTCTTGAGCTCTGGTCCTTAGGAATCACTGTCCCTTTTGAACCATAACACACCTTTCTCATCTGGCGAAACAATTATACTTCTCAACCTTAGATTAGATTCTTCTTGATAATCTGCACTCCCTTGTCACTTTGGCTGAGGCATGATAATCTGGTCTTGCAAAGCTAGCTCATGAAATTTGAGACAAAGATCGGAAGAATCACTTCAATTTGCATCTTGCTCAACTCATCACACAAGGTGTTAATCGAGAATCCATCAGAATACAATTGCATTGCAACTTCCGACTCAAGGCATCTGCTACATTAGCTTTCCTGGGTGATAATGTACCTCGAGTCATAATCCTTGATCGGCTCTAGCCATCTTCTACGCCTCATGTTGAGATTAGCCTAAGTAAAAATGTACTTAAGGCTCTTATGATCGTGAAGATGTTGCGATGAGGTTCCCATTAGATAGTGCCTCCATCTTCAATGCATGAACCACGCTGCTAACTCAAGGTCATAGTGGGATAATTTTGCTCATGAGACCTGAGTGCTCTTGAGGCATAAGCAATGTTTCGTTGTCTTGCATCAAGACACAACCTAGTCGGTGCCGGAGAGCATCACAATATACATCAAAGGCTTCTCTGCTTTGTCGGGTTGCGCCAACACTGGTGCTGTGGTCGATCTGCCTTAATGCATGGAAGCATCTTCGCATGACTCCACACAAATTTGACCTCTTTCTTGACAGCTCGGTGATAAACTTGCATGGTCGAGAAGTCGGAATAAATCTTCGGTAATAACATTAATCGAAGAAGCTCGAATCGCGGGAACAGTTGTTGGTGGCCTCGAGTTCATCACCTCTTGCACTTTATCAGGATCAACTTGCTATTCCATCACGAGATGAGTGTGACCCAAAATTTGATTTCCTTGACCAAAAATCACACTTGGATAACTTGGCATAAAGGTGGTGCTCGCATGACGTTGAAGCACTACATGCAAATGCGAACATGTTCTTCTTCGTTCTTTGAGTACACGAATGTCATCGATGAAAACCACCACAAACTTGTCCAATTCCGGCATGAAAGCGGAATTCATCGGATACATGAAATATCTTTGGTGCATTTGTCGGCCAGATGACATCACCAAGAATTCATATAGCCCATATACGGTTGAGAATGCCGTCTTGAATATCACTTGCTCGTATTTTTGATGATCGATGGTAGCGGAGCGGGTCTATCTTGGAAAACACCTTGGCCCGACCAAGCGGTCAAAGAACATCAATACGAGGCAAAGGATACTTGTTCTTGATAGTTACGCATTAAGAGGCGTAATCTATACAGACCTCAAGCTTTCATCCTTCTTCTTCACAAGCAGTCTTTGGACATCCCCAAGGCGAAGTGCTTGGCGAATAAAACCATATCCAGCAATCTTGCAATTGCTTCTTCAACTCTGCCAACTCGGCAGGTGGCATTCGGTAGAGCCTCTTGGAAATTGGGCATTTCGGTTGCAACTTTCGATGGCGAATTCAATATCCGATCCGGTGGCATTCCGGCAATTCATCAGGAAAGACATCTACTCCTCAGACCACTGGGATCTTCTTGGGGAGTAACTCCGTCATAGAAAGCACATGGTGAGAAGAACCTGACTAAGCGAGAATCAAAGTGAAATTCCCAAAGGAAAGAATCAACTGCAGCGGCATTGGCTACATCAAGCACAACTTGGTGCAAGGTCATCAATTTGCCCCTAGAATAATGTCACATTTTCCAATCCCAACACAAGAAGAGTGGTTTTGATAATGTGGCTTCCGATTGAATAGGCACACTTTGGTTTAATTGATTAGTTGCAATTTTACCCCGGTGTGACTATCATGAATGACCCTTTTGAGTGAGAGAATGGCGGTTCGTTTAGCACTGAACTTTTGGCTAATGAAACTATGAGATGCACGAGAATCAAGCAGAATTAAAGCAAGTTGATTATAAAGTGAAAAGATCACAGGTCATGATAGGAGCTCCTTCGGCACTTCCTCTAGAAGCGGTGAAGTTGAGCTTCCCTTGCGACTTGCACCCATACCTTTCTTCCCTTGTTTTGATTTGGTGCGGCATGCCTCTCTTTGGTTCTGGGACAATTCTTGGCATAGTGGCCCACGTTGCCAGAAGTGAAACACTTGTTCCCATTGCCACTGGCGGAGCTACTTTCTGGCACTGCGGGTGATTGTTTCTTAGGCGGTGGATGTGATTGGTGCCCATGCTTCTTCTTCTTCGAGGTAACTTCATCCATCTGCACTGCTGCTGAAAGCCTGCTCTTATTGTGAGAAACAATCCGGAACCTCACGAGCACGGCGGATGGTGCGCCATTGGTGCCTTTCTCTTCTTCTCTGCCCGGTGGCAACAATGCAATCTTCTGGGGAGATGGCCATGTTGACCAACTCATTAAAGCTATCGGCCGGGCCGGTGTTGAGTCGTTCCAGCGACTTAGTATTGAGACCCCTGCGGGCGATCCTCTTCTTTCATGAATCGGCGCGATACCGCATCGGCATAAGTCATTGAAAGGCTTGTGCATCTGCGACGTCCGTCGGTTCCTTGATTAAGAGGCGAAATTCATTCAACTTCCGATCAAGAATGCCGGTGGGATGTGGTGTCCCACGAAGCGATCTTGAATTCCTCCCAAGATACCTCACGATCGTAGGGAGCATAGCACGAAGTGGTCCCACCAAGTCCGGCGGGCGAAGGCTCGTCGCGTGGCGAGCCTTAGTCTCATCGAGCGATCCTCACGTGAGGAGAAACTGGACTCGATGGCGAGCCACAGCGTCGGCGTCCGCGGATCCTACTGCCTTGGTGAACAAGAGGCGGTGCGTGCTCGAAAACTCTGTATGTTGCCATAGCCGGGAGGTCGTGATCTTGCCTCCACCATGATCTTTGTTGGTGGCTGGCGCAGAAGAGCTCGCGAATCTCATTCTGGCGGGCCAGCCTCTGCGCTTTATGGGGTGAGGAGGTGGCGGGAGCTTGCTCCGTTCTGCCCTGACCCGCCTGGTACGCCATCGGCAGAGATTGTGCCATTGTTATCCCAACTCCAGTTTGAGCGACAAGATATCATCTCATATGGAAGGAAAAATGCCATAATCATAATATTAGGTTCGAAATGAAGATAACATGGTGACAAGATCCCGATTTCAAAAGTTTATAGGGTTACATCAATCAGGAAAGTACCTACAAGCCTAGTCCAAAATGTGATACTACTAAGCTGCATAGGTTTCTATCCGCCTAAAATGTCAAGCTGTTGCTTAACCAGAGCGTAGTAGGCGACCGCGGATACGAATGAAGGAGGCATCGCGGAGGTAGTCCCATTGGCACGGTAGTCCTGGCTCTCGGGGCCTCTTCTCCTAAACTTCCTACTTCATTGGCCTCCATCTCCGGTGGTGCATGTCCAAGTGGCCATTGGCTTCCTCGAGTTCCTACGCACATCGTGGTGGTTCTCAAGACATCAATTGTGTTGTCTCGGATCTCCACTTCTTTGCTCGAGGGGTGGTATCTGCTGGCTCGACTTTCCACTGCAGATCCTTCTCCACCAACTCTGTGGATAGGTCGACCACAAAATCTTCATTAGTCGAGAGTGAGCTTGGCACTGCGGTGTTAGCAAGAAGTGTCATAGCATCGCTCTGAAGGGCTGAAGGCGGTCTGTGCACTCATGCAGCGAGCATTGACCCTCCCAACCAAGTCGGGATACATTGCCCACACATCCTTCATGGCTCACAGCGGTTACACCACATGGGATCATCCTTCTTCTCGGCGGGAAGAGTCCCAGGGTGCATCACCATCTCGGGATGGTAGTGTACGAGTTGTCGAGTCTTCATGGCGCATTGCCTCAGCGGTGTCGTCGTCCCAGTCAATCGTCTGGAGAGTCAAGAGCGTAACTTGGTGAAGGATGAGCCTCCAAAGTCGGCCGGACGACAACAAGGTACCCGTGCTCCTCATACAAGCTGCAGGTGTACAAAGGGGCGTAGAATGGCGGCCGAGTTAAGCACTTCCCACAAGATGGAAGGAAAGCCATGCGAGAAAGGAATCGAAGACTGAAACGAGTCTCCTCCTTTGGCGGGGGTGGGTGAATTCATCTATGGAAGGAATCAAAATAAAGATTATGGTGGAAGGAAAAGAAAAAGAGAACCGGATGGTTTTTAAAGAAAGGGGTTAGCTCAAATTTTAATTCCTCTTTGGGGTTTATAATGCATGCATCTTTGGAAAGCGTTGCCTCTCAAAGAAAGATAGGGTGCCTTTTAGGGCATCCTTAAAATATAAATATTACTGCAGCCTTAATTAGCCACCTATTTCTCCCTCTGCCTAAGGCCTTTCGTCCTAGGTCTAGCGGTCTAGTCTGTCGATCCGTGTGATTTCTAGAGCAAGTTTAGATTTTGAAAATTAGTATTCATGGTTTATTATCCTTCTGTGGTGGAATTTGCTCCGATGCAGTGTAAGCGAACCACCCGAATTATTCGGTTTAAGTGCTAAGTCACGCCCAGAAGTATAACACTTAAATCGAATAACCGTCGATCCCTCATATCTAGTCTGATAGAGCCATAACGAGGATCAAATTCCACAATCCCACTCGAAGGTGAGTCACAGAAGAAATACAAAAAACGAAACCTCAAATTAAACCGAGTTATTACATAAATCAGAGTTTTAGTAGCAAATAAAGTTCACAAAATAAAGTCTGACGGATGTCGATGTAAATGCGGTAATGAGGAAATGGGCAGCCTGGCCCACTACTCCTCATCTCTCTGCCGAGCAACATCACTAGTATCCAACCCAGTTATGGCGGAGTGGTAAGCCAATCCATCAGCCAATTCTGTATGGTACACAAAAATTGTGCCACAAGCAAAGGTGAGTATACTAATACTCGGCTAGACTTAACTGGTGTGAGAGTCTACTCCTACCTCTGAACTATGCATTTGTTTGGTGAGGGTTTGGTTGCCAAAGCACTTTGTTTTCTAAAATCAATTTTTAGCTTTTCAATTTCTACCATCATTAACTTAGCTAGATTTGCTCCTTCTAAGCATACATGGTAACAATCAATTAGTTCAATCAACAAGTTATCTCATGTAGTCCATTTCACTTCTTACTCGATGCGATTACAAGAATCAAGCGGTCTCATTAGCTCACGAAAGCGAGCGATCGAATCGAGTTTTAAACCTTGCAGGTAAACCTAAACACACGGCATGTCGGGGTACTCGACCCGCGCATGACAACCGTCCCCATCGATTCCCGTTAAGCGTCCGGGCCTCATCGCCTTGGCATACAATGCTCCACTAACCCATTGCCGCCGTGCGGTGGCTGCACTTGTACCACCATAGCTAGCATGGGAGACCAGTCTCAGTCGCATGAGGATAAAGTCGCGCTTGACTTCACTCGAGTACTAGGTTACGGTTACCATTTTCGGCATGTGCTTGATACGTTCAAAGCTTGACTCGGTATCCACATTAATCCTTAATTCATTTTTCCGTCTCATGGACAAGGCATCCTCCACGGATCCAAATCCATAGACCAACATAGATCCGTTATCAAGATGAATACAATCAATTCACGACCTGTGCAGTGCTAGAAAAATCACTCGACTTCTACCGAGATCTGATTATGGCGACTACTCGACCTAGCATACTAGTATTCATCTCAAAGGAATCCTAAGTTCATGCAACTAGAGGTTTCAAGCAACTCCTACACTAAGTGCATTGCAATCCTACAAGCATTAAGTGTAGTAAAGTAGCATAATAACATGGTTATGCATAAACAGGGCTTGCCTTCAATTGTTGGGGCTGGGAGATCCTCAATACTGACCTCAGGCGCTATCTGGTCCTCCTCTTGGGACGAGTCCTTGCTCGGGATGAGCAAGTATCTCGTCGACAAGATTACAATCTAATGAATGCAATCGTAAGATATATGCATGATATGATATGTGCTTTTAGAAATTACAACTTTTAAGGTGTAGGATCTTTTGAGTTTAAAACAAGTTAACTTTACTTATGTAAAACCCTTAGTGGTATACTTGGTAAATTGGGTTAGACTTAGCTAAGTAAGTGGTAGGTTTATTTTTTGGGTTCCATTGAATTCCTTTTAAGTCTTAGGGATAAATGATAAGTTCTCAAATTAGACTTATTGGGATGAGAGTTTATTCCTTCTTCTCTTTTCTTTATTCTCTTTAATGTTTTGGAGTAGGTTTGAACTACAAGTTGCTTTATAAAATTCTAAAATTTCACAAAAATTACAGTGGCTTGTTACTGGTGTATGTTTCCTGTCTCAAAATTTGGGGTCAGAAAGTGAATAGTTTTCTCTGACAAAATTATTAAACTCAAGGTGAGAAGAGGTGCTGAACTACAACTACTATTTAACAGATGGGTAATTCTCTAAAACTTATTTTGCTGGCTTTTAGGTGTTATAACATGACTTGATACAAATTTCTAATCATTAATACCTCTTAATAAATTTTCTATGGTTTTCTCAAGTTTCTAGCCAAATGGGTGCTCTACCACTATATTTGAAAAATATCAAACAATTCTTATTTTTTCATAGTTAGTATTTTGTGCAAAGAGCAATCATTGAAGTTTGGCTTCTTTTGCTTAAGGGAAGGGGTGGTTGCATTATTTGAATTAAGTGGTCTTTCTCTTTAATATTAGCAAAGGTATGGGTTTACTTCTTCTTCATGGGTTTGCATTTTCTGCTGTAGTTTACCTCCTCATGAGCTTAGCAAATTTTGGTTTGCTATTATCACATTAAAGGTTGTTCATGATTTATTTGGAAAATGCCTTATTATCACCTTGTATTTATTTTCTTTACTTAAAAAGTTGGGTTGAGGTGCTCTGTATTTTTGTAGTGAGGCTCTGGTGGTTATAAGTCCACTAGATTTTTATTAATCATTTTGGTTATAGTTTGTAACTCTAATAATTGATTTTCAGTCTATATGAGGCTAAACAAATCATCTTAATTTAAAGCGGTCCAAATTAATGTCTGCATTTTTCCTAGGTTCTGTTGCATAAGTAAACTAGGAAAAATATTATTAATCCTTGTTCATTAATTTCTAGGCCTTTACGATTTTCTCTAAGTTTTGGACAAATTAGCTTTAAATGAAAAACTACATCATATTTTTTCAATCTTTGGGTTCCTACTATTTTAAATAGTGTCTAAATAAGGTTTAAACATCTACAATTTTTCTTTAAGTTCAGCCTGGAAGAAAAACTAATTTTCCTTAATTAAACAAGGTTTAGTGGGTTTACGTCTTTAATTTTAAACTTTAAAATTTTGAGCAGAAAAGCATGGAGTTCTATTTTTAAATGATAGATCATAATATTGGGAGCTAGCAAAATTGGTTTGACAACTTTTATTAAGATTTCATCATGTTATGGGTTTTCTAAGTTCTTTGGTCATTTTAAAAGAATAAGAATTGATTAAATGGAAATCCACTTTGCACGAGGGTCCTGGCGATTTTCTAAGTTTCCCTCGCGAATCGGTCCTTAGGTTACTATTCATGAGTCGCGACATTAGAGAAACCCTCGGGTTCTACCAATCCTAACGAGGTCCTTCTTCTACCTTAAGCGATGTCGCGGCGGAGAAAAGAGCGAAGAGGCTTACTGCGGCGAGGTTGCTCGGTGGTGAGTCGAGAGAAGGGGAGGTCTCGAGGATCAGCGGTGTGCGAACACCGTCGGAGAATGGCGGAGTCGGTCGATCCGCAGCGCGAGGGGATGCTCGTCGTGGCGAGAGACCGACCTTGTCACGCGAGATAGTTCAATTAGATGGGTCGGGAGGTTCACGGATGCGGAGAAGATATGGCGAAAGGAATTGGCGGAGACTCAGCTGGATAGCTCGGTCCACAGCGTGAAGACCAAGTCCGGTGAGGTCGATCTCGGGCCTCCGGTGAAATCGGCGGTCAGGGCAGCAGCTTCAGCTACTAGCGGGCTAGCCAGAAACCTTTGGGTGGGCTTGAGGGTGGTGCGAGTGGGTGGCCATGGTGGAAGCTCGTGGCAATGGCGCGAATTAAGCTCGCGAGCTAAGGAAAGGTGGTGGCGGGAGAGAGGTGAGTCTTGAGCGAGAGAATGGCAGGAGGCGCTATCGCGCGGCGGTACAGTAGAGGCGTGGGCGGCGGACAATAGAGACGCCGGGGTGGCGAGGTTAGGCGAGCTCGGCGTCCGACCGAAGTCAGGCTGCGATGTGCCGTGCGTTACGCCCAGTACGCGCGTGTGGTCGCTCATCAGAGCTGCTCTCGCCTGGTGGTGCACAAAACCTCTTCTCCTCCCTACAAGCTACCATTCTTGTGTAGGGGTCTTAGGTTTTGCCTCGGTTGCGAGAGATATGGAGCGAGAAATCTGTCTGTCTCCCTGCCCAAACCCGAGGCAAATCAAGGTTTTGTCGTGTCTAGGGCTCAGCGTCCCAATGCCATCTTACGGCACCTGACGGAGGGTTAGTTAGACACAATTTTGTCAATGGGCCATTAGGATTCAAGTTAGGGATCAAGGTGAACATCCCGATCTTTGGCTCAAGTCGAATTTGAGAATCACAGAATTCAGAAATCTAATGAGTCCCACATGGGAGCTTGATTTGGGGTTTATTTTTATTTTGGCTAAGCTTTCTCAACATTTCTTGTTGCCCATTAAATATTCTTTAATTTTCATAATTGGCTCAAGTCATAATTTTAAACTTTTCATTCCCTTTTCTTATTTTCTTGAATTTTGCTCATGGGGCTCACTTAGGGTTCTTAATTAGTTGCACATTTTATCTTTTCAAAGACTCAATTGTTTTGATCATGTTACTTTTAAGTATTTACTTGGTGAATTCTTTATTACTTAAGTTGTTTGATGCTCATGCTTACTTTGGTTCACATAAAATGATGGTTCTTGGTTTGGCATTTGAGAAAACCTGAGTGACCTTTAGGGTGTCCTGATCTGAGCCACAGGGTCAGGCGACGGAGTTAAATCGTCTTGGTGAGCCGAGTCAGAGCCCAGGTCGAGTGCGTGAGGGTTCGCCATCTTCCGGGTGAGCAAGTCAGGCTCCGGGTTAGGCGGGCGGAGTTGCGCTCTTCGGAGAGCCGTCCAGAACCCTAGGTCGGCGGAGCGGAGTTCGCCGTCTTCGGAGTTGAGCGAGTCCGAGCCCTAGGTCGAGGCGAGCGGAGTTCGCCATCTTAGGTGAGCCGAGTCCGAGCCCTAGGTCGGGCGGCGGAGTTTCCTATGGTGCACGAGGCGAACCCCGGCCACTGTCAGCCTCACTCTGTCGAGTGCACAGCCCGATCGGAAGCGCGCGGCGGCGCTATCCTTCTGTCAGCGGTCGGTGGGCGGCGAAGTAGCGCGGTCGCCGGCTCTGTCGGTGGAGGCGTCGTCGGATAAAGGTGTGAGCCACCTCGCATTAAATGCCCTGCGATTTGGTCGGTTAGCCGGCGATTTGGCGGAGTTGCTTCTTAGCGAGGCCGGGCCTCGGCGAGCCGGAGTATGTTCGTCGGCCGGAGGGCCTCGGCGAGCGGAGATCCTCCAGTGGTGCGCTTGCCGAGCTAGGCGCTGCGGGCGGCGTGTCGAGTCCCTCGAATGGACCGATCCACGACTTAGTCGCACCCATCGGCCTTTGCGGCTTTGTCGCGATGGGTTACAGTGAGAATTAGAGTCTTGAGGTACCCTAATTATGGTACCGGTTAGCCCGAGCCTCGAAGAGTGTTAACACTCGCGGAGGCTTTATCACACTTTTGCAAGGGACCAGGCCTTTCTCGGTTGCGTTTTGTTCGGTGGAGTGCGCGCGGCGCCCCGGCGATCTCTTTCAAGGCTCGGAGGAGTGGTTTGACTCGAGGTCTTAATACCTCACGCAATGCTTGGCCGGTCTGAGTCGTTCCCTCATCGGTGGCCGTAGCCGGGTGCGGTCGGGTCCCAAGTTCTTGAGCGTATGTTGGCGTTGTCAGGTTTGGCAGGCGGGTTGCGAAGCGACCGAACCTCACGCCGCAGGCGAAAGGGCAGTCAAGGGCAGTTCGACTTTTTAACATCGCCCCTAAATGCCGCTTCAGGAGGAGAGGAAAGCGCCATGTTGCCCTCGGAGGCGCCAAACATGGTGTCTCGGTCAGTTTCTTGGCGGGTAATCAGTGGGCGCTTCGTGCCCCATTTGTTAGAAGTCGGCTAGAGGCCCGGGCGCGCTCAAAAGTACCTCTGGTGATCTGGGGACCGGTCCTGTCGACGGGTGAGGCTCGATGCCTCCTCTGATGGGATTAGTTACAAGATCATTTCTGCTGGTCTCGGAATGTCTTAGGGTACCTCGGGCGTGGCGAGCCTTGGTTATGTATCGAGCGTACCCATGGTCATCCCTCGCTCAGTATCTGGCGGTTGTGAACCCTTCGAGGGCGACCTTGAACCCCCGATCGATGGTGGGCGCGGGCCAGGTGGCTGGTGGCCGTTGAACCCTCAGGGGCCGACCTTGAACCTCTTGATGGTGGGTGTGGAGCCCACGCTCTGGCGGTGTTGAACCCCTCAGGGCGACCTTGAACCTCTTATCGGTGGGAGGCTGGAGCCTGTTTCCTTCAGAGAAGGATCCTTTTGGGGTATCCCCTTTCCCGGTCCACAGTATAAGAGAGAAAGAGGAAAAGAAAGGATGCAAAATCGAATGGCGTGGCGTACCTCTTTTGGCCGCGGTCATTGTGGCGAAAGCGGCGTCGCTCGCTTCCCTGCCGGGCTGCGCCTTCCCGCCGCGGAGTTAACGGCAGGCGGCGGTCATGGGCGGCCGTTGCGGCGTCGCGTGGCGTTCGAGGAGCGGAACGCAGGCGCGCCGTCTTCACCCGCGTGAGAGGTTCTCTCAACGCCCGAGATGGGGCGTAAGCTTAGGTGGCGGCCATTCTCCCGCCACTTTGCCACCGCCATTCTGCGGCCCATTTTGGCCGTATTGACCATCGCGCTGGGCGCCTTCTTGCAGGTCGTGCAGCGAGTCGCCTCAGTCGCGATGCCGGTTCCATGATCGAGGCGTGGCGGTGGTCGTGCGATGCGATTGCCTGCGTAGCGGCCGTGCGCCCGGTTGCGTGACGCGTGGCGTGCCCCATCTTTGCGTCGCGTTGGAAGTCGGAGCGCCGCCAGCGCGTGATTGCATACCGCTGCATAGCTGCCCGCCCTTCGCCCACGTGATGCAGGCGAAAGTGGAAGAATCTTTCGTAACGGCCCAGGCGGTTGCATGCCTATGAGCGGTTTAGCTAATACGCTGCAGGCGGCTTATGCGTAGCGTGGGTCGGCCCGTCGTGGGGAGGACCTTGGGCGTGTTGGAGAAGACTCGGCCCACGGTGGGGCGCAAGTAGGAAGTCGCCTTTAAAAGGTGGGTGACCCCTTGAAAGGCGACCATGTCTTCGCAGCTCCCTTATGTCGTGTCTTTCCACCTTGAGCCCGGATGGGACACCGATCCCTCGCCTTGTGCGTTGGAACGCAAACTCCGTGGAGTTGGTACCTTTAACCATCGTTGGCTTCAAGTTTTCATCAGCGGCGTGCACCCCTCTGCCGGCGGTCACCAAGATGGTGACCTCGGTTGATGGCGGGAAAGCAAACAGGCTGCGGCCTCTGCCCTCCCTCGGCCTCAAGGATTTTCATCGCCGGTCTTTGGGAGGGAGTGTCTCGGTGGGGTCGGCCCGATGTGCGGCGGCGGCCCGCTCCTTCCTCGGTGATCGAGGGAAGGGCGTTCGCCGTTCACGGTGGCGGTACGCGTGCAGCTCTCGGCCGAGTGGCCGGGCGCTCCGGCGCCGCCTTCCGCCATGGCGGTGGAAGGGTTCTTCGCCGACAAGATAGCGGGGGCCATACGACCAAACCTCCAACTCTACGCCACGCCGATCCTTGCCTCACGAGTTTGGGCATGGCGAGAGCCTCATGGCGGCGACATCCACCACGAGTCATCGGCTGCTTGTTAGGCCGGAGAGCGAGTCGTCATCATCGTCCGGCCGTTGGGCGGCGGCGTGCAGCCGCTCGTTGATCTTGTTGCTCATGAGCCCCAATCGTGTGGGGTTGTTCATTACTGCGGAGGTGGAACGAGTTCAGTTTGTAATGGCACCTTGAATGTCGGTCTTTTTCATTGTTGTAATTGGGGCACGAGCGTGTATATTTTTGGCGCGAGCCGTGTTTTTCCTTATTTTGAGCACTAAGACTACTTTGTTGGTTATGCGAACCGCTTCACCAAAGTGAGTCGCCCGTACAAGGTGACGAGTGAGGTATCCGTATCCGGGGCGTAAAGTCCCTCGGCTCGATCGGCCTTCTTGTGAGCTTCTCTTCTTAGTTAAAGAACCCCTGCGCGCTCTTGATGGCGGCGGGGTAGCGGTGTCGCCTGCAGGCAGGCGAGTTGGCTCGAAAAGAACGGTGGCGGGCTGCCGGGTGCGTCCAATAGCGGGGCCAAGCGCGAGTTGATCTGCAAGGCGGGCGGTGATGTCTTGAGACGGTGGCGAGGCCTCGGGGTGACGGCCGAGCCGCCTACTCGGCCGGATTCTTGGAAGACCTGGCGGCGATGGCGAGCGTGATGATGATGTCGTCCTTAGTGGAGATCCTCGGACCGCGTCATGCGTCAGGGCTAGGTCGGACCTCGGCGAAGGTGTAGTCGGCGCCGAGGTCTTCTTTGCTCCCTTCATTCATCAAGATCAAGCTGCGGGATCGGATTATCTTGTAGTGTGCGTTTACTGCGGCCGCCGGGCTAAACACACCATCGTGTTGTAGCTGCGCTTCTTTTCCTCTTGTTTTGAGTATCTGGACTTTTTCGTAGAACGGAGTTATGCTGCATGCGGGAGTTGCTTTTCACGGAAGGTGGCGAGTGAGGTATCCGTATCCCGCGTAGAATCCTCGGCTCGGTCGGCCTTGCCGCCGTGCACTCTTGCCCGTCCATGGGGTTACGCCGTAGCGATCGAAGGGCGAAGAATCGCCGGAGAGGCTTCGAGTGTGAAGACTTGTTCGATCGCGAATCGCTTATCGAGCGCGAGTTACTTATCACAGAAGGTGATGGTGAGGTATCCGTATCCGGGCCGTAGGAGTCCTCGGCTCGATCGACCTTGGTTGCTTCGCGTGTACTCCGTCGTTTTGGATCCACTTTGAGTAGTCGAAAAGCACGAAAGACATTCGGAAAGGATCTTTTTGAGGAAAATTTCGGTAGGAGGGTTCCCCCTTTTAGCCCAGAGGAGGTCGGGCTTTGCGAGGCAAGGTGACCCTTCCTTGATGACTAAACTGCGTGGAGGCGGTATATGAACAACGAAAGCATCTTAAGGGTAGAGCGGCGTGGTGTTGGATGTTCCAAGCGTTCTGTGGACCTCGCCTTGTATTGGCGGCTTATGCGTTCAGACGAACTTTGGCGATAATGACGGCCCTTCGAGGCGCGTGAGCTTGTAGCCCTCGGGTCTATGCGGCCAAGCACGGTGTCGCCCGTGAGGAGTCTGGGACGGACCCTCGGGTGTGGTAGCGTCGCGGGCTTCTTTGTCGTGCGAATGTAGTAAGGCCATGTCCAGGCCTCTTTCCGTGATAGCGAGTCTTCTCGACTAACTTGGTTGCTTTGGTGGCGTAGGCCCTGGTCCTTGGGGGCCGTATTCTAAGTGCGTGGGCAAGATGGCCTCGGCCCCATAGACTAGAAGAGCGGCGTGAAGCCGTGGCTCGGCTCGGCGTTGTCCTCGGACTCTAGACCAGGGAGTTCCTTCATCCATCGCGCGAACACTGTTGAGGTCGTTGTAGATCCGAGGCTTGAGGTCCTTGTAGAATCATGCAGTTGGCGCGCTCTGCCCATTCGATCGGGTGAGCCACGGCCCAGTCCACCGGATGTGGTGATCCTCATGAAGTCGGGAACTTTGCGGTGAGGCTGGTGCGTTGTGGTGATGATGGGTTGGGACTGAAAGGAAATGTGCTTTGGGCCATTTCTAAGTATTTTAGTGATTGAGTGCAAACACAAGTGCTTAAATGTGAAAATATGCAGGATGAACAAAGTGCAAATCACAGTTAAGGTATGTTTCTAAGCCTTAGTACATTGGTTTTGTGTACTAATATATTTGTCTAAGTGTTAAAAGCAGATAGAAAGAAGAAAGAAGACTTGGTGTGTACTGCCAAAGAAACGCTTGATGCAGAACACCGGTGTCGGTGGTGCCGGACGGTGTCGGTGGTGCCGGATGATGTCCGTGCGCTTGACTACCTCGGCCGAAGAGGCGCTCGGGTTTTCTCGGCGACCGGCTAAAATTCACCGTTGTCCGGTGTGCCGCGTCCGTTGAGCCAGCGATCGGCCGGCCAGCGGTCGGCCCGCGATCGGTGCGACACGTGGCGAGCCAACGGTCGGAAAAAGCCGGGTATCGGTGTGCCGGGCCCGTCGGTCGCGGATCTACCTTGATCGGCAGCGGTCGGCCGCCATTTAAGAAACAAATCGGCGCGGACGGTGTCCGTGTGCACGACTATCCGTGCGCCCGCGAGAACGAAAGGCAAAGATGGCCTTCAGTTTATTCCCAATGTCCTAGCTGCCTTGGGGCTATAAAGGGACTGGCGCATGGAGGAGTACACAAGCATTCCTACAACTCTTCTAAGCACCAAGACATCAATCTCACGATTCGTTTCATTGTGATAGCATATAGAGCTCTTGTGGAGTTGTGAACTCTTTGTGTTGCGTTAGAGCTCTTGTTGCGACTTGTGCGTGTTGTTGCTCGATTTTCGAGTCTTGTGTCGTTGCTCATTCCCACCTTACTCAGTATTTCTTTGTGAACTCAATTGTAAGGCGAGACTCCAAGTTGTGGAGATTCCTCTTAAGCGGGAAAAGATCAAAGGAAAGAAAAACACCGTGGTATTCAAGTTGATCATTGGATCACTTGAGAGGAGTTGAGTGCAACTCGTCGGATTGGGCGCCACAGCGTGGAGTAGGCAAGTTTGTACTTGGCGAACGGGATAACCACGTGTCAACTCTGATTGCTTTCTTGTGGTTATTGTGTTTTGAGTTCTCTCTAGCCACTTGGCCATACTTGTACTAACCCTTAACAAGTTTTGTGGCTTAAGTTTAAGTTTTCTGGGATCACCTATTCACCCCCTCTAGGTGCTCTCAATTGGTATCGGGCCGTTCTCTTCAGAAAGGGACTAACCTTGAAGAGATGGATCCTAAGGGAAGGAATTGTGATCAGCGACAAGGAAGGTCGCCGTCAGGAGCCAAGGGATGACAAGTCCAATGACTCGGGCTCGGGCTGGCGAAGAGATGGGAAGAAGAAGACAAGGCGCATCAGGAGATCGTCTACCTCTACGGCAATGATAGTCCTCTTCTTCCCAAAGGGCGGCGACCACGACAAACAAAGGAAAGCGGTTAATTCTAACTTTTCTTTTGACTACTCTCGTATTCATAAGTTCAAATTCACATTTGCTTTCTATTCCACTCGGCAAGCCCCACACTTTGATGGGGAGGACTACGGATTTTGGAGCCACAAAATGCGTAGTCACCTATTCTCTCTCCATCCTAGCATATGGGAGATTGTAGATAGTGGAATGCACTTTAATAGTTGGATAGTCCTATATTCATTAATGAGCAAATCCATAGAATGCACAAGCTACTCTACATTCTTCTAGCCTCATTGTGCGGGGATGAATATAATAAAGTGAGTGGCTTGGATGCCTTAACAAATGCAGGATACCTCAAGATCTCTCATGAAAATGATGCTACCTTACTCACCAAAATGGAGTTGGTAGAAGGCGAGCTTGGGCGGTTCGCGATGATAAGGCGAGGAACTCAAACATACAAGCCGACTCAAGACCCTTGTCAACAAGATAAGGAGCTATGGAAGCGCGATGGGCGGACCACGACGTCGTCGCCTCATGCTAAGGTCGCTACATTCTTGATCCTCATTTGGTGAATAATATTCGTGAGAATCCCGTACACCAAATGTCGCCCGAAGAAATTCTTGGAAAATTTGTAGCGGGCGGATGATGATCAGGAGGCAAGGTGCGTCGATGAGCGTTGAGCGGTCAATCCACGAGCCTCAACCCATTGCTCTCAAGGCATCGAGGCAAGGAGGCACTACCAAGCAAGGTGGTGCAAATTGAGTAGCCGGGCTTAATGATGAAGAAATGGCTCTCATCATTAAGCGCTTCAAAACAGTGCTAAAGGTGCAAATGGACACCGAGGCAAGACTAAGACCAAGGGAAGCGATCATGCTTCAAATGCGGTAAGCTTGGTCATTTATTGCTAAGCTGTCCGATAATGAAAGTGACCGGAAAGGAAACAAGAGGAAAGAAGAAGCATTATAAGAAGCCAAAGGGTAGGCGCATCTAGGCAAGGAGTGGGACTCGGATTGCTCTCGTCCGAGGCTCAGAGCAATGAAGGACTGCCGCCACCTTCAACAAATCAACCCTCTTCCCAAGCGGCGTCACACATGCCTTATGGCAAGGAGAAGAAGGTATGTACTCATAACTCTACCTATGCTTCTTCAAGTGAGGACGAATCTAGTGATGAGGATGAAGTAGATTATTCATGTTTGTTCAAGGGCTTAGATAGATCTAAGATAGACAAAATTAATGAATTAATTGATGCCTTGAATGAAAGAATATACTTTTAAAGCAAGAGGATTTGTTGTATGAGAGCATGATAAATTTGTTGAGGCACAAAAATCCTATGCTTTAGAAGTTAAAAGAAATGAAATGCTTTCTTTTGAACTATCTACTTGTCATGAAACCATTTCTACTTTGAAAGGTGTCAACAATGATTTAAATGCTAAATTAGAAGTAGCAAATAAATCCAATTCTTGTGTAGAACATGTTGAAATTTGTACTAGGTGTAAAGATTTTGGCGTTGATGCTTGTAGTGAACACCTAGTTTCAATTTCCAAGCTTAATGATGAGTGGCTAGTCTTAATGCTCAACTTAAGACTAGCAAGAATATTCGATAAGCTAAAATTTGCAGGGATGCCTACACATTGGTAGACACCCCTCAATTAAGGATGGACTTGGCTTCAAGAAGCTAAGAACTTAACAAGCCATAAGGCTCCCATTCCATAAGGAGAAAGGAAGGTCCCTATGGCTAGTAATGTGCAAAAGAACCATGCCTTTTGTATCATGATAGAAGACAAACTAGAAATGTAAGTGATGCTTTTGATTCATATGTTTATGATTCTCATGCCATGTTTGCTCCTAGTTCCTCTTATGTGTATGATAGAAATGTTACTAGGAGAAATGTTGTTCCTAAAAGAAATGCTATTCATCATGTGCCTAGAAAGAATGTTATTCATGCTCCTAGGAAAGTAGTGAATGAACCTTCCACAATTTATTATGCTTTAAATGCTTCCTTTGCTATTTGTAGAAAGGATAAGAAAATTGTTGCTAGGAAGTTAGGGGCAAAATGCAAGGAGACAAAACTTGCATTTGGGTCCCTAAGGATATTGTGCTAACCTAGGACCCAACATGAGTTGGGTACCTAAAACAAACCTAAATTTGCCTTGCGAGTTTATGCATCCAGAGTTCAAATTGGATTATCGGCAGCGGATACAAACCATATGAGAGAAGATGTTCCTCCTACGTCAAAAATAAGGATTCCCAAGATTCAATTATATTGGTGATGGGAATCAAAGCAAGGTAAAGGGTTAGGTAAAATTGCGATTTCTAATGAGCATTCTATCTCAATGTATTTTTAGTAGAGAGTCTTGGATATAATTTGCTATACATTAGTCAATTATGTCATATAGTATAGCTGTCTATTTACAAATGTAGATGTATCATCTTTAGAAGAAGTGATGGTTCACTAGCTTTTAAGGGTGTATTAGACGGCAAACTTTATTTAGTTGATTTTGCAAAAGAAGAGGCGGTCTAGATGCATGTAATGGCTAAGACTTACGTAATTTGTTGTGGCATCGCAGCTTAGCACATGTGGGGATGAAGAACCTTCACAAGCTTCTAAAGAGAACACGTGATAGGATTGACTAACGTGCATTTCGAAAAAGATAGACCTTGTGCGGCTTGTCAAGCGGGTAAACAAGTGGGAGGAGCACATCACACAGAGCGTGATGACCACTTCAAGACCCTGAGGCTGGCATATGGATCTCTTGGACTGTCGCCTATCGAGCATAGGAGGGAGTAAGTATGGTTTAGTTATTGTTGATGACTTTTCCCTTCACTTGGGTGTTCTTTTTGCGATAAGTAAAACCCAAGGGACCCTCAAGCGCTTTCTCGGAGAGCTCAAAATGAGTTTGAGCTCAAGGTGAAGATGAGGCGACAATGGGTCGAGTTCAAGAACCTTCAAGTGGAGGTTCCTTGAAGATGAAGGATCAAGCACGAGTTCTCCGCTCCTACACCCACAAAATGGTGTGGTAGAAAGGAAAAAACAGGGCGCTCATCGACATGGCGAGGGCGATGCTAGGGAGTTCAAGACCCAGAGTGCTTTGGGCAGGCGGTGCTGCCGCTGCCACGCCATCAACGGGGTCTACCTTCATCGCCTCCTCAAGAAGGCGTCGTATGAGCTACTAACCGGTAACAAACCCAATGTATCGTGCTTTCGTGTATTTGGGAGTAAATGCTACATTCTAGTGAAGAAGGGTAGAAATTCTAAGTTTGCTCCCAAAGGCGTAGAAGGTTTTTATTAGGTTATGACTCAAATACAAAAGCGTATAGAGTCTTCAACAAATCATCGGGTTTGGTTGAAGTCTCTAGCGACGTTGTATTTGATGAGACTAATGGCTCTCCAAGAGAGCAAGTTGTTGATTGTGATGATGTAGATGAAGAAGATGTTAGGCGGTGCTATACGAACCATGGCGATTGGAGAGTGCGCCCGGGAACAAGATAACGAGATCAATCATCTTCCTCAACAATGGTAGCCCCAACTCAAGTGGAGCGCTTGATCAAGGAAGCGCAAGATGATCAAGTGATGGAGGAAGAAGCGCAGCGGCACCTCCAACCCAGGTTCGGCGATGATTCAAAGGGATCATCCCGTCGACCAAATTCAGGTGACATTAGCAAGGGAGTAACTCGATCTCGATTAGTTAATTTTGTGAGCATTACTCTTTTGTCTCTTCTATTGAGCCTTTCGGGGTAGAAGAGGCCTTGCTAGATCCGGACTAGGTGTTGGCCATGCAAGAGGTTAAACAACTTCAAGCGCAATGAAGTTTGGACAGTGCCTCGTCAGAAGCAAAATGTTGTAGGAACCAAGTGGGTGTTCCATGAACAAGCAGGGCAAACACGGGGTGGTGACGAGGAACAAGGCTCGACTTGTGGCAAGGTTATGCCCAAGTCGCGAGTTTGGACTTTGAGGAGACTTTTGCTCTGTGGCTAGGCTAGAGTCCATTGTATTTTGCTAGCATATGTCGCTCACCATTCTTTGAGTTGTTCCAAATGGATGTGAAGAGCGCTTTCCTCAGCAGGCAATCAAGGAGGAGGTGTCGTGAGCAACCCAGCCGAGGATGAGCAGTACCCCGACCATGTGTGTAGCTCTCTAAGGCTCTCTATGGACTTAAGCAAGCCCAAGAGCATGGTATGAATGCCTTAGAGACTTTTACTTGCTAATGCTTTCAAGGTTGGGAAGGCGATCAACTCTTTTACAAAGACATGTGATGGTGATTTGTTTGTGTGCCAAATTTATGTCGATGACATAATATTTGGTTCTACTAACAAAAGTCTTGTGAAGAGTTTAGCGGGGTGATAGCGAAGTCGAGATGTCAATGATGGGCGAGTTGAACTACTTCCTTGGGTTCCAAGTGAAGCAACTCAGGACGGCACCTTCATCTCCCAAGCGAAGTACACCAAGATCTGCTAAGCGGTTTGGGATGAAGGGCGCCAAGCCGCAAAGACTCCGATGGGGGCCAGACGGACACAGAGCCTCAACAAAGGAGGTAAGTCCGTTGATCAAAAAACATCCGGTCAATGATAGGTTCTTTGCTTTACTTATGTGCTAGTAGGCCAGGATATTATGCTTGCGTATGCATGTGTGCTAGATTTCAATCCGATCCTAAGAATGTCACTTGAGTAGCGGTGAAGCGAATTCTTAGATATTTAGTTGCTCACGTGCTTGGAGCTCTGGTATCCAAAGGGTCTACCTTTGACTTAGTTGGATACTCGACTCAACTATCTTTGGATGTAAGTCGATAGGAAGAGCACATCGGGGCGTGCAATTCTTAGGAAGGTCCTGGTGTCATGGAACTGAAACAAACTTCCGTTGCCCTATCCACCACCGCGAGTAGGTATGTTGCCGCGGAGACAGTGTTGCAGCGCAACTACTTTGGATGAGGCAAACCCTCGAGGACTTTGGCTAATCGAGCAAAGTCCCACTCTATGTGACAATGAGAGTGCTATCATGGCGGAGAATCACATTGAACACATGCTCGCACATAGACATCGGCATCACTTTTGAGAGACCACCGCAAAGGAGATATCGAGTGTTTCATGTGCTACAGAGAACCACTAGCCGATATCTTCACTAAGCCTCTAGATGAGAAGACCTTTTTTTGAGTTGCGTAGTGAGCTAAATGTCTTAGATTCGCGGAACTTGGATTGAATTGTAGCATACATGTAGTTATGCTTTTGATCATGTTCCTTTTTGCATTATGTTGCTTATTATGGTGCTCAAGTTGTACAAACACTCCTGGACCTCCAAGTCCGTTGCAAAGTGATGCACACGTTTGGGGAGATGTGTTACAACTTGACCCTTTGAACTAACCATTTGCTTGAGTTTGCTTGATTTAGTCTCGAAGGAGGATTGAAAGGAAAAGGTGGACTTGGACCATGAAAGACTTCAGCACTCAGATGAGAGGGTAACTAATTCCAAGTTCATCTCATGAAATCTTATTGCCATTTGCTCTTAATTGAAGACTTTGGTGAGGCAATGGGGTTAAAAGGCCAAATTAATCCGTTTTGGTGCTTGATGCCAAGGGGAGAAAATAAAGGCAAAGTGATAAATGGATCCTACCACTTGAGAGATTTTGAAAATAGTAGAATAGAGTTTTTGTTTTGTCAAAAGCTTTTATGTCTCTTATTGTCTCTATTGTCAAAAGTTGGCTTCTTGTGGGAAGTGTTGATTATGGGAAATAGGGGAGTTTTGAAATCTTTGATCAATCTCTTTTGGAATGACTCTCTTATACTTCATCATGTGTGTTTGACTTAGAGATAGAGATTTGAGTTTGATTTGCAAAAACAAACCAAGTGGTAACAAAGATGATCCATATGCCAAAATTGAATCAAAACCAATTTGAGTTTTTATTTGGTGATTTTGCACTTGTGCTATCTACTTTATGTTGTGTTGGCATAAATCACCAAAAAGAGATTGAAAGGAAATGTGCCTTTGGGCCATTTCTAAGTATTTTAGTGATTGAGTGCAAACACAAGTGCTTAAATGTGAAAATATGCCCAAGGATGAACAAAGTGCAAATCACAAGTTAAGGTATGTTTCTAAGCCTTAGTACATTGGTTTTGTGTACTAATATATTTGTCTAAGTGTTAGAGCAGATAGAAGAAGAGAAGAAGACTTGGTGTGTGCGACCAATTTGCTCGATGCAGAACGCCGGTGTCGGTGGTGCCGGACGATGTCGTCTTGCCAGCTACCTCGTAAAAGGCGCTCGGAGTTTTCTCGGCGACTTGGCTAAAATTCACGGGTGTCAGGTGTGCCGGCTGTCCGGTGAGCCATGGTCGGCCAAGCCATGATCGGCGATCGGCGCGCGACACGTGGCCAGAAATAGCGGTCGGAAAAAAACGCGGGTATCCGGTGTGCCGGGCTTGTCGGTCGCGGTCTGCGATCGACAGCGGTCAGCCGCCATTTAAAAACAAATCGAGACACCGGGCGGTGTCGGTGTGCCGGTGTCCGGTCTTACGAGGCGAAGGCAAGATGGCCTTCGGTTTGTTATGGCTCCTAGCGCCAGGGGCTATAAAAGGACCCTAGGCGCATGGAGAGTACACCAAGCATTCCTACAACTCTTCTAAGCACCAAGACATCAATCTCGCGCATTCGTTTCATTGTGATAGCATATAGAGCTCTTGTGGAGTTGTGAACTCTTTGTGTTGCGTTGCGAGCTCTTGTTGCGACATGTGTGCGTGTTGTTGCTCGATTTTGAGTCTTGTGTGCGTTGCTCATTCCCACCTTACTCAGTATTTCTTTGTGAACTCAATTGTAAGGGCGAGAGACTCCAAGTTGTGGAGATTCACCATGAGGAAAAGATCAAAGGAAAGAAAACACCGTGGTATTCAAGTTGATCATTGGATCACTTGAGAGGTTGAGTGCAACTCTCGTCAGTTGGGGCCTTGGCGTGGAGTAGCAAGTTTGTACTTGGTAGAACCACGGGATAACCACCGTGTCAACTCTGTGATTGCTTTCTTGTGGTTGTGTGTTTGAGTTCTCTCTAGCCACTTGGCCATACTTGTACTAACCCTAACAAGTTTTTGTGGCTTAAGTTTAAGTTTTCGAGGATCACTATTCACCCCCTCTGGTGCTCGGGACCACGAAGCGATGGATGATGTTGGTGAAGAACGCCACCGCTTGTTCGGACTGATCTTTGTTTAGGGTCGGACCTCGATCCACTTGGAGAATTTGCGATGGTGACCAGCAGTGCGTGTAGCCGGGGTGCCATACGCAAGGGGCGGCGAGATCCGGACCACGACAAAGGCGGGTGATGGGTATCGTCTGCGGGGCTGGCGAGCGGTGGTCTGCCTTGCGTAGGCCCGACACCCTTCGCGAGTGCGGACAATTCTAGTGGCGTCGGCCACCGTTGCTGATGAAACCTTGTCGGGCGTTTCCAACAAGGGCTGAGGTCTTCGCGTGATGGCCACAAGCCCAGTGTATCTCTTGGAGCTCGGCCTTGGCGATGGAAATGCATCGGCTGGAGGATGCTGAGGGCTACGGTGGTAGAGCTCTTTCGTCCCCACCAAGACAAGCGACTTGGCATGCGGCATCAACGTAGAGCTTGGCTCGGTCGAGGGTAGCTCTCCTCGGTGGAGATATTGCAGTCGGGTCTGCCGTTTCGATTAGGCGTGACCCAGCCGCTCCTCCTCGGCGTGCGGTGCCTTATCCTCGAGGCGGCGAGGTACCTCGGGCGGTGAGTGCCTGGGCCGAGCGAGTGCCTCGGGCGAGCCGAGGGTGCCTCGGGCTGGCGGTGCCTCGGCTTCGGGACGTGTCGTCGATCTTGGCGGAGGGTTGATGCAGGTCTCGGGAGAAGGCGTCGGAACCGTTGTTCGCCGAGGCTATTTTGTGGTGTAAACAAATTGGCGCTTAGACAACTTAATAACATTGCGGACGTTTTAATGTACTGGAATTGAATTAATTCGGGGGATCTGGATTTTAGTACTGGATTTTGGTTTTAGGAATTAGAAATTTTATTGATAGAAGTATTTTACAAATACAAATACATACTAAGGGTTTCTTATATGCTCAACACATGAGCGAAACCTATAGGAACCTAATTCCCTTATCTGGGAACTACTCACACATTATTATGGAGAAACTCGAGCTCCTATTAGGCGGTGGCCTCAGCGTACTCGGCGGCGCCGAGGGCTTGGTGGCAGCGAAGAGTGAGGACTTGCGGATGTGGTGTGCGCGGTGATGCGGAGTGGAGCGTCTGCAGGCCGAGGTGAGCATGATCATGCGGTGGTCGCCGTGGCGTCGGCGGTGATGCCTGCGAGTGAGCGAGCCGATCCGCGAGAGAGTCAGCGGTCTCGGCGTGCGCGCGGAGGCCGAGGCGCTCAAGCTCAGCGCGGTGTCGGTGCGGTCGCACTCCTTGAGGGGAGAGTGGCCACGTTCTCCCAGGTGGTGTCGCCCTGGCCGAAGGCAGCAGCGGCGGTGAGGGCTTGCGGCGTCGGTGAAGGAATCGCCGTCGCGAGTCCGCGGCGTGGAGAGGCGTACCGCGCCGCGGTGGTATCGCCCTCGCGCCGCGATGTCAGCGCCCATCTCGCGGAGCACGTTCGGCCTCCTTCTCGCCCTGGAGGTCGTGCTCGCGAGGTTAGAGAGCGCACCTCGCCTGGGAGGAGGGCAGCGGCGGTGAGGATGGCAGCGAGGCCGGGTAGTCGCCCGGCACGAGCACGCGGCCTGGGCGGTACTTCTGGCCACCGGGATGGAGATCGGCGGAGGTCGTCGGAGGCAGTGGCGCGCGCCGAAGTCAGAGGGTGTCGAGTGTCTGGCGGAGCGGAGCGTGGGACTTGATGTCGCGGTGAGGCGGAGTTCGAGTCCGTGGGAGGAGAGGGCCGAGGAACATGAGGCGGAGGCGTACTGGGAGGCGCTCGGCGGACACCTCCACGGTGCCACCGCGCACTGGGCCGGACACGGGATCGGGAGGCGACCGTCGTTGGAGGACACCCAGGCACCGAGGCGTTCGAGGGCCTCAAGGAGGTCGCCCTGAGGGCGCTTGCCGAGGAGTCCGGGTAGTCGGTCACGAAAGTGGTGCCAGAGGTGAGCGGCCACGCCCATGAGGAGGCGGGCCACCGCGGCAGCGTTGCCTGGAGTTGAGGTCACACCGGCCTGTGGGCGAGCGCCGAAACCGCGGATCACGGCGTCATCGCCCACAAGCTCCACGCCAGCGCCCCAGTCGCGGAGGCAGCGGAGCATGGCCTCGGCGTCCTCAGAGTAGCCACGCCCACCACGCGGGTCTCGCCCTCAGCGAGGGCAGCGGCGAGGCAGCGAGTGGTGTAGTTCTTGGATGGCTGAGCGCGAAGCTCGCCGCGGAGTTCGCGAGCTGGATGCACGATCACGTCGAAGGTGGCGGGCAGGGCGTCGGATCCTTGCGGGGATCGGTGGGGCCCCACGGCCGGGCAGGGTGGCCGGGGAGCCATCGGCATGGCGGGTGACGCCGCTGAGGCACCTGATGGGCGCGGCGAGGCGCGGCGGGTGGCGGAGGTGCGCCCGCGCCTCGCCTTGGGCGCAGCGGTAGTGGCGCCAGTGAGCGCGGTAGACGCGGCGGCGGCGGTGGCCATGGTAGATCCTCTAGAGTCGACCTGCAGTAACGCAAACAACAGGGTGAGCATCGACAAAAGAAACAGTACCAAGCAAATAAATAGCGTATGAAGGCAGGGCTAAAAAATCCACATATAGCTGCTGCATAATGCCATCATCCAAGTATATCAAGATCAAAATAATTATAAAACATACTTGTTATTATAATAGATAGGTACTCAAGGTTAGAGCATATGAATAGATGCTGCATATGCCATCATGTATATGCATCAGTAAAACCCACATCAACATGTATACCTATCCTAGATCGATATTTCCATCCATCTTAAACTCGTAACTATGAAGATGTATGACACACACATACAGTTCCAAAATTATAAATACACCAGGTAGTTTGAAACAGTATTCTCGATCTAGAACGAATGAACGACCGCCCAACCACACCACATCATCACAACCAAGCGAACAAAAGCATCTCTGTATATGCATCAGTAAAACCCGCATCAACATGTATACCTATCCTAGATCGATATTTCCATCCATCATCTTCAATTCGTAACTATGAATATGTATGGCACACACATACAGATCCAAAATTAATAAATCCACCAGGTAGTTTGAAACAGAATTCTACTCCGATCTAGAACGACCGCCCAACCAGACCACATCATCACAACCAAGACAAAAAGCATGAAAAGATGACCCGACAAACAAATTGCACGGCATATATTGAAATAAAGGAAAGGGCAAACCAAACCCTATGCAACGAAACAAAAAAATCATGAAATCGATCCCGTCTGCGGAACGGCTAGAGCCATCCCAGGATTCCCCAAAGAGAAACACTGGCAAGTTAGCAATCAGAACGTGTCTGACGTACAGGTCGCATCGTGTACGAACGCTAGCACTGGATCTAACACAAACACGGATCTAACACAAACATGAACAGAAGTAGAACTACCGGGCCTAACCATGGACCGGAACGCCGATCTAGAGAAGGTAGAGGGGGAGGGACGAGCGGCGTACCTTGAAGCGGAGGTGCCGACGGGTGGATTTGGGGAGATCTGGTTGTGTGTGTGTGCGCTCGAACAACACGAGGTTGGGGAAGAGGGTGTGGAGGGGTGTCTATTTATTACGGCGGGCGAGGAAGGAAAGCGAAGGAGCGGTGGAAAAGGAATCCCCGTAGCTGCCGGTGCCGTGAGAGGAGGAGGCCGCCTGCCGTGCCGGCTCACGTCTGCCGCTCCGCCACGCAATTTCTGGATGCCGACAGCGGAGCAAGTCAACAGTGAGCGGAACTCTCGAGAGGGTCAGAGGCAGCGACAGAGATGCCGTGCCGTCTGCTTCGCTTGGCCCGACGCGACGCTGCTGGTTCGCTGGTTGGTGTCCGTTAGACTCGTCGACGGCGTTTAACAGGCTGGCATTATCTACTCGAAACAAGAAAAATGTTTCCTTAGTTTTTAATTTCTTAAAGGGTATTTGTTTAATTTTTAGTCACTTTATTTTATTCTATTTTATATCTAAATTATTAAATAAAAAACTAAAATAGAGTTTTAGTTTTCTTAATTTAGAGGCTAAAATAGAATAAAATAGATGTACTAAAAATTAGTCTATAAAACCATTAACCTAAACCCTAAATGGATGTACTAATAAAATGGATGAAGTATTATATAGGTGAAGCTATTTGCAAAAAAAAGGAGAACACACACACTAAAAGATAAAACTGAGAGTCCTGTTGTCAAAATACTCAATTGTCCTTTAGACCATGTCTAACTGTTCATTTATATGATTCTCTAAAACACTGATATTATTGTAGTACTATAGATTATATTATTCGTAGAGTAAAGTTTAAATATATGTATAAAGATAGATAAACTGCACTTCAAACAAGTGTGACAAAAAATATGTGGTAATTTTTATAACTTAGACATGCAATGCTCATTATCTCTAGAGAGGCACGACCGGGTCACGCTGCACTGTAGGCATGCGTCGAGAGATAGATTTGTAGAGAGACTGGTGATTTCAGCGTGTCCTCTCCAAATGAAATGAACTTCCTATATAGAGGAAGGGTCTTGCGAAGGATAGTGGGATTGTGCGTCATCCTTACGTCAGTGGAGATATCACATCAATCCACTTGCTTTGAAGACGTGGTTGGAACGTCTTCTTTCCACGATGCTCCTCGTGGGTGGGGTCCATCTTTGGGACCACTGTCGGCAGAGGCATCTTGAACGATAGCCTTTCCTTTATCTGATGGCATTTGTAGGTGCCACCTTCCTTTCTACTGTCCTTTTGATGAAGTGACAGATAGCTGGGCAATGGAATCGAGGAGGTTTCCCGATATTACCCTTTGTTGAAAAGTCTCAATAGCCCTTTGGTCTTCTGAGACTGTATCTTTGATATTCTTGGGTAGACGAGTGTCGTGCTCCACCATGTTCACATCAATCCACTTGCTTTGAAGACGTGGTTGGAACGTCTTCTTTTCCACGATGCTCCTCGTGGGTGGGGTCCATCTTTGGGACCACTGTCGGCAGAGGCATCTTGAACGATAGCCTTTCCTTTATCGCAATGATGGCATTTGTAGTGCCACCTTCCTTTTCTACTGTCCTTTTGATGAAGTGACAGATAGCTGGGCAATGGAATCGAGGAGGTTTCCCGATATTACCCTTTGTTGAAAAGTCTCAATAGCCTTTGGTCTTCTGAGACTGTATCTTTGATATTCTTGGAGTAGACGAGTGTCGTGCTCCACCATGTTGGCAAGCTGCTCTAGCCAATACGCAAACCGCCTCTCCCGCGCGTTGGCCGATTCATTAATGCAGCTGGCACGACAGGTTTCCCGACTGGAAAGCGGGCAGTGAGCGCAACGCAATTAATGTGAGGTTAGCTCACTCATTAGGCACCCAGGCTTTACACTTTATGCTTCCGGCTCGTATGTTGTGTGGAATTGTGAGCGGATAACAATTTCACACAGAAACAGCTATGACATGATTACAATTCGAGCTCAGTACCCTGGATTTTGGTTTTAGGAATTAGAAATTTTATTGATAGAAGTATTTTACAAATACAAATACATACTAAGTTGTACAAAACCAGCAACTCACTGCACTGCACTTCACTACTTCACTGTATGAATAAAAGTCTGGTGTCTGGTTCCTGATCGATGACTGACTTCTCCACTTTGTGCAGAACAGATCTAGAGCTCTTAGTAGAGGCGGGAGATGTTGGTCGGCACGAGCATGATGTTCATGAGGTCGAACTGTGCCAGAGTTGAGGTCACGTTGATGTCGAGCGGCACGTCGGAGTTGGAGGAGGCCACCACGTTGCCGATGTTGATGTCGGAGAAGCGAGCACCGTTGTCGTTCACGCCGTCGTTGTTTGTGGTGGTGTTCACGTTTGTGGCGGTGTACACGGCCGTTGATGGTCACGCGGATGGTGGAGTTGCCGATGGAAGACACGCGGAGGTAGAGGTTGTAGGAGTTGCCGTTACCGCGGAGGGTGTAACGAGCGGTGTTGTTCTGCTCGAAGCGGAGAGAGTCGCCCTGGTTGCCGAACTTCTCGGAGATGAAGGTGCGGGTCTGGTTGTTCACCTGGGTAGCGTGGATCGGGAGATGGTGAAGCCGGTGTAGTCGTTTGGGCGAGGTGGGTCATGGAGCCGTTCTCGTGCACAGCGTGGATGTTGTTCTTGCGGTTGTGCACGGACACCATGTAAGCGCGATGCACCTCAGGGTGCCGGACGGGGAGGCGATGTTGCGGATCTCGTTGTAGTGGAGTGGGCGACGGAGGTCCTCGTTGCGCACCACGAGCGGCACGCCGGAGATGTTGCGGATGAAGTAGTCCGGGAAGTAGTTGGAGTTGCCGCGTGCGGTGAAGGCACCAGGAGCGGAGGCCGAGGTGGTCTCGAAGGACTCGGTCTGCCAGTTGGTCACGGTAGCCACGCCCTCGCGGTCGGAGCCGGAGTCGAGCCAGGAGCGCACGAACGGTGTGAGGAGCGGAGGGAGGAAGGTAGAGCAGTTGAAGTTCTGGTTGAACGGGAAGCGCCGATGTCGCCGGAAGAGATGCCACCGGGTAGTTCACGCGAGCGGCGAGGAGGGCGTGGGTAGTTGTGGAGCCAGGAGGCCCACGATGTTCGGGAAGGTGTTGGGAGGCGGGCGCCGGAGAAGCCGTTGAGCACGTAGTTGGAGTTCACCTGGAAGGGGAGTAGAGGAACAGCCAGTCCTGGGAGGTGAAGGACTGGGTCTGCTGCGGGCGGAGCCGGAGGCGTAGAGGTTGGCGCCGGAGGACACGAGGAGGACTGGTACTTGAAGAGGGACCAGATGGACACGTACTCGAACACGTTGAGGAACATGTAGGTGCGGAACTCAAGCATCTCGTGGAGAGGGTGTTGAGCTTCTTGAAGGCTGTCTGGTAGGTGTTGATGCAGTAGTTGGAGTACTCCTTGGTGTAGTCCTTGAGGTAGCCACGGTAGGTGCGGAGAGTGGCAGCGGAGATCCCACTCCTCTGCGTTGAGGATCACATCGCGGATGAAGGAGAGGTGGAGGTTGGCGGCCTGAGCGAAGAGTGGAAGGAGAAGGAGTTCGTAGCCGCGGAGCTGGAACTGGGTGAGGCGGTTGAGAAGAGCTGCTGCATGGTGTTCACCGGGGATGTGATGGAAAGGAGAGTCGGGTTACGGTTCGGGTTGAGGAAGTTGTCCACCTGACGGTTGAACTCGGCAACGTTCTTCTGGAGGCCCTCAAGTTCGGCGTTCACGCGGGAGAGGGTGTCAGCGTTGAGGCGCTGGTTGAGGAACTTCTCGTCTCACGGAGGATGTCCTGCATGAGCTTGGTGTTGTCGTTTGGGAACACGAGGTCCCAGAGGCCATTGAGGATGCGCTTGGCAGCAGAAGCCTCCGAGCTTCTTGAGGAGGAAGATGGCCACAGTACCCACAGGAGCCACGTAGAGTGAGTGGTCGTCGCGCTTCCTCGGTCCACTCCTTCTGGATGGTGTCGAGGACTTGTACTGGAAGAACGGGTCCTGGGCCATCACGTTGTGCCGTCGCAGATGGTGGTGCGTCCGGAGTTGAGCACGGAAGCTCCTCCACCCTTCCGGGCCTCGCACCTGATCGATGTGGTAGTCGGTCACGTCGGTCTTCAGGCCGATCTGGTTGCTGCTGGTGAACAGCTCGTTCACGGCCTTCTGAGCCCTCTCCAGGTCGTACTCGGCCTCGAAGGTCACCTCGGCGGGCACGAACTCGATGCGGTCGATGTACACCTCGTTGCCGCTGTTGAACACGTGGGCGCTCAGGGTGAACACGCTGCTGCCGTTGCTGAAGTTGAAGGGTGGTGAAGCCACGGTGCGGAAGCTGCCGCTCTGCAGGTTGCTGCCGCTGCTCATGGTGGCGCTGAAGTTGCCCTGGTTGATGGGGCGGCCGTCGATGCTGGTGTGGAACTGCAGGTTGGTGGTGCTGGCGTAGCGGATGCGGACGCGGTAGCGCTGGCTCAGGGCGGTGATGTTCACGCGCAGGGTGCTGATCTGGCCGGGGCTGGTGCGGCGCAGGATGTCGCCGCCGGTGAAGCCGGGGCCCTTCACCACGCTGGTGCCGCTGCCCAGGTTGGTGCTCTTGGTCAGGGATCTGGGTGATCTGGCTGCTGGGGATGATGTTGTTGAACTCGGCACTGCGGTGATCCAGCTGAACATAGGTGCACGGATGATGCTCACGCTGCTGTTGCTGAAGCCACTGCGGAACATGCTCACGTGGCTCAGACGGTGGCTGAAGCCCTGTCGAGGTGGCACGTTGTTGTTCTGAGGATCTCGTCCAGGCTGTCCACGGTGCCGCTCTTGCGGTACACGGCGCTGGGCAGGTTGCTGCTGGTGCCGTAGGCGAACTCGGTGCCGTCCAGCACGCTCAGCTGCTGGTTGTTGATGCCGATGTTGAAAGGTCGACGGTACAGGGTGCTGCTCAGGGTGCGGTACACTCCCTGGCCCAGCTGTGCCACGATGCGCTGCTGAGGTGCAGCGTTGCCCATGGTGCCGTACAGGGAAGGTGAACTCGGGGCCGCTGAAGCCGACGGGGCTGGCCGCGATCTGATGGCCGCTCCAGTAGTACTCGCCGCGGTGGGCGTCGGTGTAGATGGTGATGCTGTTCAGGATGTCCATCAGGTGGGGCTGCGGATGCTGCCCTCGATGCCCTAGGCGCTGCCGCGGAAGCTGCCGTCAGAAGTTCTCCAGCACGGGGTTGGTGTAAATCTCGCGGGTCAGCTGGCTCACGGTGCGGATGGGGTAGGTGCGGCTGTCGTAGTTGGGGAACAGGCTCACGATGTCCAGCACGGTCAGGGTCAGCTCGCGGCGGAACTGGTTGTACCTGATCCAGTCGCGGCTGTCCGGACCCCACACGCGCTCCAGGCCGGTGTTGTACCAGCGCACGGCGTGGTCGGTGTAGTTGCCGATCAGGCGGGTCAGGTCGTTGTAGCGGCTGTTGATGGTGGCGGCGTCGAAGCCCCAGCGCTGGCCGAACACGCTGACGTCGCGCAGCACGCTCAGGTGCGGTTGGCGGCCTGCACGTACACGCTCAGCAGGGGCACCTGGTAGTTCTGCACGGCGAACAGGGGATGGCGGTGGTCAGGGCGCTGTTCATGTCGTTGAACTGGATGCGCATCTCCTCGCGCAGGGCGGGGTTGGTGGGGTCGGCCTCCCACTCGCGGAAGCTCTCGGCGTAGATTTGGTACAGGTTGCTCCAGGCCCTCCAGGCGGCTGATGGCCTGGTTGCGGGCGAACTCCTCGATGCGCTGGTTGATCAGCTGCTCGATCTGCACCAGGAAGGCGTCCCACTGGCTGGGGCCGAAGATGCCCCAGATGATGTCCACCAGCCCAGCACGAAGCCGGCGCCGGGCACGAACTCGCTCAGCAGGAACTGGGTCAGGCTCAGGCTGATGTCGATGGGGGTGCCGGTCTCGATGCGCTCGCCGCCCAGCACCTCCACCTCGGGGTTGCTCAGGCAGTTGTAGGATGCACTCGTTGATGTTGGGGTTGTTGTCCATTGTTGGATCCTCTAGAGTCGACCTGCAGAAGTAACACAAACAACAGGGTGAGCATCGACAAAAGAAACAGTACCAAGCAAATAAATAGCGTATGAAGGGGCTAAAAATCCACATATAGCTGCTGCATGTGCCATCATCCAAGTATATCAAGATCAAAATAATTATAAAACATACTTGTTTATTATAATAGATAGGTACTCAAGGTTAGAGCATATGAATAGATGCTGCATATGCCATCATGTATATGCATCAGTAAAACCCACATCAACATGTATACCTATCCTAGATCGATATTTCCATCCATCTTAAACTCGTAACTATGAAGATGTATGACACACATACAGTTCCAAAATTAATAAATACACCAGGTAGTTTGAAACAGTATTCTACTCCGATCTAGAACGAATGAACGACCGCCCAACCACACATCATCACAACCAAGCGAACAAAAGCATCTCTGTATATGCATCAGTAAAACCCGCATCAACATGTATACCTATCCTAGATCGATATTTCCATCCATCATCTTCAATTCGTAACTATGAATATGTATGGCACACATACAGATCCAAAATTAATAAATCCACCAGGTAGTTTGAAACAGAATTCTACTCCGATCTAGAACGACCGCCCAACCAGACCACATCATCACAACCAAGACAAAAAAGCATGAAAAAGATGACCCGACAAACAAGTGCACGGCATATATTGAAATAAAGGAAAAGGGCAAACCAAACCCTATGCAACGAAACAAAAAAATCATGAAATCGATCCCGTCTGCGGAACAGCTAGAAGCCATCCGGATTCCCCAAAAGAAACACTGGCAAGTTAGCAATCAGAACGTGTCTGACGTACAGGTCGCATCCGTGTACGAACGCTAGCAGCACGGATCTAACACAAACACGGATCTAACACAAACATGAACAGAAGTAGAACTACGGGCCTAACCATGGACCGGAACGCCGATCTAGAGAAGGTAGAGAGGGGAGGACGAGCGGCGTACCTTGAAGCGGAGGTGCCGACGGGTGGATTTGGGGAGATCTGGTTGTGTGTGTGTGCGCTCGAACAACACGAGGTTGGGGAAAGAGGGTGTGGAGGTGTCTATTTATTACGGCGGGCGAGGAAGGAAAGCGAAGGAGCGGTGGGAAGGGTCCCCGTAGCTGCCGGTGCCGTGAGGAGGAGGAGGAGGCCTGCCGTGCCGGCTCACGTCTGCCGCTCGCCACGCAATTTCTGGATGCCGACAGCGGAGCAAGTCCAACGGTGGAGCGGAACTCTCGAGAGGGTCCAGAGGCAGCGACAGAGATGCCGTGCCGTCTGCTTCGCTTGGCCCGACGCGACGCTGCTGGTTCGCTGGTTGGTGTCCGTTAGACTCGTCGACGGCGTTTAACAGGCTGGCATTATCTACTCGAAACAAGAAAAATGTTTCCTTAGTTTTTAATTTCTTAGGGTATTTGTTTAATTTTTAGTCACTTTATTTTATTCTATTTTATATCTAAATTATTAAATAAAAAACTAAAATAGAGTTTTAGTTTTCTTAATTTAGAGGCTAAAATAGAATAAAATAGATATACTAAAAAATTAGTCTATAAAACCATTAACCCTAAACCCTAAATGGATGTACTAATAAAATGGATGAAGTATTATATAGGTGAAGCTATTTGCAAAAAAAAAGGAGAACACATGCACACTAAAAGATAAAACTGTAGAGTCCTGTTGTCAAAATACTCAATTGTCCTTTAGACCATGTCTATTTGTTCATTTATATGATTCTACAAAACTTTGATATTATTGTAGTCTATAGATTATATTATTCGTAGAGTAAAGTTTAAATATATGTATAAAGATAGATAAACTGCACTTCAAACAAGTGTGACAAAAAAATATGTGGTAATTTTTATAACTTAGACATGCAATGCTCATTATCTCTAGAGAGGGGCACGACCGGGTCACGCTGCACTGCAGGCATGCAAACTTGCACTGGCCGTCGTTTACAACGTCGTGACTGGGAAAACCTGGCGTTACCCAACAATCGCCTTGCAGCACATCCCCTTTCGCCAGCTGGCGTAATGGCGAAGAGGCCCGCACCGATCGCCCTTCCCAACAGTTGCGCAGCCTGAATGGCGAATGCTAGAGCAGCGAGCTTAGATCAGATTGCCGTTTCCCGCCTTCAGTTTAAACTACTCGGTCGTTGTAGCGTCGAGGCGTGGTTGGCTTCGAGCCGTGAGCATCTGAGCAAAGCGTCGTCGCGATGAGCCTCCATCTTGGTCGCGGCGGTGGGAGTTCTTCATGACTTGCCGATGGCGAGTGCCCGAGTCACCGGCGTCGGCGTCGGACCCCGGCTCGATGGCGATCGCGGCCGATTGACGAGCCGTGCCGACCACATTGTTCGGCGGCAAAGTGGGCGTAGCACATAGCGTAGGTGCTAGGCGAGATGAAGAAGCGACTGCAGGCGGCTCTGTCTTCATCGACCCGTCGAAGAACATGGTCGAGTTAGGTTGGATCGAACGGCCCGGGAGGCCGGTGTCGACCCATTCACCTTGAAGTCCGCGAACACAGGACTTGATGGCCCAGAAGCGAGCGAGATCGCCGCCCATGATTTCCACCGCCCGCTGCGATTCTACAGGGCCTCGGCGCTAGATGATCTCGGGGAAGGATGACACGATTCGGATGAGACTCAAGTAGTGTGCGCAACTTCCGCGCGTCGGGATCACCGCGTCTGCAGACTCCACGAATTTGTGGGTAGCGGATCTTGGTGCATTGATGCACCGATGAAGTAGACTTGCCTCGGGCCGGGCAATGCATGCCCCTCTTCTCGTCTCAACCACGATCGCGGCGCTAAGCCTTTGAGTGGTCGCGGCTTGTAGATCAATAAGGCTTCTCCAGTGAGGGCACCAAGATGGGCGCGTTCGTGAGGCGCCTTGAGTTCGAGGCTTCCTCGGCCTCGGGGTCCAAGTGAAGCACTCGGCCTTCCTTAAGAGGTATAGAGCGAGCCTCTTTCGCAGCGTGAGATGAACGGCTCGAGCCGCAAGACATCCGTGACTCTGTCGCCCTTCAAGTCCTTGATGGGCCCCATCTTGTGATGGCGCGATCTTCTCGGATTGGCCTCGATGCCCCACTCGGAGACAATGAACGAGCATGCCTCGAGGAACCCCAAGACACACTTCTGGATTAAGCTTCCGTCTTTCGCCTTGAGACATCGGAATGTCACTTCAAGGTCGGAAAGGAGGTCGAGGCTTTCCTCGTCTTGACTCGATGTCATCGGCGTAGGCCTCGGCCGTCCGACCAATGTGTTCGCCGAACACATGGTTCATCACACCGGTGATGCGTCGCACCCGCATTCCTCAAGCGACGGTATGGTGACATAGCGGTACATGCAGAAGGGTGTGATGAAAGAAGTCGCGGTGTCGGATTCTTTCATCCCGATTTAGTGGTACAGTAGGCATCGAGGAAAGACAAGGTTTCGCACCCAGCGGTGAATCCACAATTTGATCGATGCGAGGCGGAGGTAGGGAACGCCGGACATGCTTTGTTTAGACAGTGTAGTCTACACACATCCGCCTTCCCTCCTTTCTTTCTCAAGAACGAGGTTGGCAAGCCATTCGGGATGGAATACCTCTTTGATGAACCTGCTTTGCCATTAGCTTGTGGATCTCTCGCCTATGGCTCTGCGCTTTTCTTCATCGAATGGGCGAGAGGCAGCTTGAGTCGGGCTCCAACTCGGATATCGGCGGTGCTCGGCGACATCCCTCGGTATACGGGCATGTCAGGGACTCCGGCAGCGTCGGCGTTCTTGCGGAGAAATCGGCAACCTTTGCACTTCCTATTTGGGATCGAGCTCGGGCCGATCCGGATCTGCTTGGAGCGTCGGCTGGCTGGGTCGAGGGGCGGACTTAACCGTCTCGGCTCGAGTTGTCGGCGTGGCGCTTCGTCTGCACCTCCTTAGAGAGCTTTCAGTCGGCGATGAGGGCCTTGGATTCGGCGAGGGCCTCGGCGTACTCCACGCACTCCGCGTCGCGATTGACGCGTGTTTGTGCGTGGGGCGTGATGACGACGTTGGGGCGGCATCTTGAGCTTGAGGTAGGTGTAGTTGGGGACGGCCATGAACCCGCAGCATGGCCTCCCGATCTTGCGTGGTAGGTTCCGGGCAGGCCACCTCGACGTGAGGGTCTCCCGAAGTTGGAGGGTGTTCAGGCGACGGAAGGTCGAGTTGTCCGAATTTGGGCCGCTTCGGGATGATCCGTGGAATGGCGCGGCGCTGCGGACCGAGGATAGATCAACAGCGAGGCAGGGTCTCGGCATAGATGATGTTGAGGTGCTTGCCTCGCGTCCATGAGGACCTTGGTGAGCTGGCGTCACCGGCGAGGGTCGACAGCGGCGGGTATTTCCCTGCTCGGCACGTGGTCGGGGTGGTCGCTTAGAAGGTGATGGGCTTGTCGGACCAGTCTAGGTAGTAAAGTAGCTAGAGGGGGTGAATAGGCTAATCTGAAAATTTTCACAACAAACTTGAAGATTATTATATACAGTTCATTGGTGCAAGTAGGTTCAGAGCTACGTCATAAGTTGAACCACTCGAACCAATCCAATCTTTTATAAACTTTAGACTAAAATCTACTAGAAAGTATTTCTTGAAGTTCTTGAGAGAAGTAAGATATAGCTAAGTGAGGATGAAAAGATGAATATGTAAAGGCTATTTTACTTCTAGATAAACTCTACGGAGAAATCTTTATGTCAATCTTGCAAGTAAAAATATCTCAAGTTGAAACAAGTAAGAACACAAGACACAAGATTTAATCAGGTTTTGGCCACACCACAAGGTGTCCTACTCCCGTTGAGAGGCCACAGGGCGGGTCTTTTTCAGCCTAATCCTCCAGCAGCCCACAAAGGTCAAGGCAATCTCTTCTTATCTTAGCTCAGAAGCGGGTGATACCAACTTCTTAGGTCGTCCACAAATTTGGAGACTCCCAAGTAACCTCGAAGATCTTGAAACCTAGGGTTTCAAGAACACCAAGCACGCTAAGAGGGGTTTGCACAAGCTCAAGTCTTTGAAAAAGAGATGGGAGAGGAAACCAAATCGTGAGCACAAGCACAAACCTCACACCCAGAGCTCCTCCAACAAGGTTGAATCTTGAGGAAGATTTAAGTGTGAGAGATGGAGAGATGAGTGCTTTGTCTCAAGTTAGGTGAGCAATAAATGAGTGAGTGTTGGTGTGTGAAGAAGAGAGAGGGGGTCTATTTATAGTCACGGCTCAAAACTAGCCGTTTGACCAAAAACCGCTGAAAACGTTGAACAGCCCCCTAAGGCTTGGTGCTCCCAACAATGTCGCGGTATCTGCAGTCGGTGCGGCGGCGGTGTGACCGACCTGCGAGGCGAATTGACGAGCGGATCGGTTGAGCAGCCCCCTAGGCGGTTGAGCCGCCCCGACCTACGGCACATTGCCCGGTCCGGTGCAGGCTGCGATCGGAGGTCGAGAGGGTGAGAGGCGTTGAGCTACCCCAGGACGGTTGGTGGTCTAGACCGAAAGTTTTGAGAGAAACCCTAGCTCGGTTGCCACAGGGCAAGTTCAATTTGGTATGGTCGAGGAGGTCCACAATGAAGTTGAAGTTCATTTGGGTTGAAAGTTCAACTCATTGAAGTCGGCTCATTTGAAAAGCTCAGAAATTTAAGTGAAGCTCATCTAACTGTTTAGTTCAGTGGAAAGCTCAAGTACTTGATTGAAGTTATTGTTTTTCAGAAGAACACTCTAGGTTTCTCAACCCAAACCATGAGTGACCAAATAATGTTAAAGAGATTTTTGGTTTTCAAAAATAGCTTTTGAATTAGAGGACTTGAGCTATAGCAAACACTGTACAATGCAGGAAAAGAACAAGGAAGAATTACATCATGCAACACAAGATTTTACATAAATTTTATCGTCTTTGCATGAAGTCCTTGGTGCTTCCTTAAGTTCTGTTTCCTTCTAATCAAACACTGAGAACAAAATTGTTAGTACTCTTATTTGTTTGTCATTAAATCACCAAAACCCTCACTTGGGGTTGATTGCACTTACAATCTCCCGGTGATTGATGCCAAACAATTAAATCAAATATATATTTGCAATAGAAAATTTCTTTTGAATGATTGTATGTAGATGGCTCCTAAATGTGTGCGATTGTGAATCCAACGCTTGACATAATATGTCATGTAGCAACACATTTAGAGATAGTGACAAATAACCATACTAAATACAATTATCCGAGGGTGCAAGAGTGTCATGACAGGTTGTGATGTACATGACTATCTCTAAAAACCATTATTTTTACTTCATAGCGAGTAGACATTACAAAGCGATAGGACAAAGATGATGGCCAGAAGACCATCATATTACTTTGAATAACAATCCAACAGTTTTATTTCATACAATGGTAAGGGTACAAGCCACAACGGTGGCCAAAAACAAAAATAATCCAAAAGAAAAGCGAAGAACCACAAACTAGAAGTTGGATTTTCTCCCCTTTGGCAACAAGTACCAAAGCGGAGAGAGAGACAAGGATACAGAATCAATATCCTCCAGGAGGAGGATATGACGGAGGATATGAAGTGTAGTCGGAAAAAGTCCTCATCGCGTCCTCCTCAAAGTGGAGTCGAGGTGGCTCATCGTGCTGAAAAAGCAGATCACCGAGATGTAACGATGATCGGGAGGAGGTGGAGGTGGTGGTGGTAACGAGCCGGATGGCCTAGGTCACCAGAATGGGTCCGGACCCGGTAAGGAGGCGGAGGTGGGAGGATGATTGACCGGATCCCCATGGTAGGGAGGCGGGGCAAACTCCTCGCGATCATCAACATAGACTTCTTCATCTTCATCGTCTCCTGAGACATAAAGGCACCCGTAGGCTGCGGTGCCACTCATTGATCTCCGGGAGAGGATGGAGAGGCACATCAGGCGAACGAGGACAAAAGGCATGCCCATGGAGGAAGCTTGACGGCGGGTGTCGTCGATCTCGGCGGCGTCGTGCCACCTCATGGACATCGGCAAAATGTTGCGTCATGGAGAAAATGCACGCAAACCCATGGACCAAGCGAGCACCCATCCCACGGCCCGACCTCGACCATGACCACGAGCACGTGGCATGGGAGATCCATGGCGGTGAGAGGAGGCGCAGGCGGCGATGGGGAGGATGGATGATGGGGTGGCCGGGTGGCTCAGTGGAGGAGGCGGGCCTCGGGCCGTGAAAGAGGCCCACGTGCGGCGGCTGGGTTGGAGGAATCCCCGGTAGAGTGCAATAGCGGGAGTGTTTGGCGGGTTTTTGAAGTGGGTCAGGTGACCACCCGATCATCTTCATGATAAAGGGTGCATGAAGACACCCTCCGAGAGGAGACCAGAAACGCAGACGATCTCTTCCCAGATCATGTCAAACACATTGATGCGGGCGAAATGTTTGGAGTAAGGAGGAGGAGGAAGACTCGACTCTCCCAAGGGCGTTATTTGATTTCAGCCTACTGGAGAATGGTCATCGGGACAAAGAATTAAGGTACCGTAGTACCTATATGCATGTTACATTGTCGAACTCTACTCAAGAGACATGAATGAATCTGTCTCTTCGCATAGGCGTGAATGTCATGCACTGCATGTTCTTTGCCACGAATATCTCATCGAGAAGCCCGAAATATGGCAAAACAGCGATAAGCTGACTTTGTGCGATTACCCGGATGAAGAAATACATGGTGGGTAATCATACTCATCGGCTTCTCACATCTACTTTCTTTATCGGTGGCATAGGTAGCTACCACCTCCACGTTAGGGTATTATGGTCATAATGTTCCATGACACTCTCTCTAAGTTTACAGGCGGCGACATGTCGACATCTCCTATTCTCACAACCCTCCAGTCAATAAATCTATGTTCGGACGGCTGGGTGTTTCTTGGAAAGAATGCGAGGTGTAGAAGTCGGCTGAAAAGATTCCAGAACCGGTCTGGACACCCGAGATGACGAGGAGTGAGTAGGTTGGTGAACCTCAGGCGCTGAAAAGTATCTATTCCTTGAGTGAAGTCGGCGGGAGATTCATGCGGACCCGGCGGCGTTCACCGGATGCATGCGATCCGGTGACGTAGAGCATCGGCGCGGAAGGTCAGCGAGTCGTCGAGCGCCCCCATCCTCCTCCCACGGAGCATACCGAAGGAAGGCGTGCTACAGCGAGGATGAGGCGCCAACCTTTTGGCGTCTCGCGGAAGTCGGCGCGAGGCATGGAAGAGCCTCACCATCCATCCGACGAGGCCGTCCTCTCTTAGAGGGAGGCGACGCGGAGGAGAGAGGGATGGGAGATTCTCTGAATCTCGCCGAGTCGCTGCTAAACTCAATCACGAGGGGTTGCGTGACGCACCATGATTGAAAAAGGTGGTGAAAGCGTGAACGGTTGAGGTGTGTGAGTGGCTCTAAAAGGTTGTGAAGCGACTAAGGCAAGAGTGAATGAAGTGGGAGAAGAAGGGAGAGTCAAGTATATAGCGATTGGTGATCCTCGGACCCGTTCAACCCTTATGGGCGATTCATTTGGTTCTAACCGGTGTAGTCGAAGAGCAGCAGGAGGAAGATGCCAAAAATCAAATTTCGCAGATTACAAAAGTATCTTGCGGAGATTCTATGAAAAAAAATCAAAACCAAGTGTTTGCAGATGAGGCCGACGACAAAAGAAAAAAACTGCGCAAAAGAAAGGATTCTGCGGCGCCTGTATCCCTCACCGCTGCGGAAGTGACAAAAATATTTTTGCCGCCTTGGGTGACAGTGGTCACAAGAGCAAAAGCTGCGCCTGGCGGTAGTGTGGGGCTAAAAACTGCCTTGGCAATTCTGGAAGCGGCCAGACGAGGAAAGAAAAATTAGGCCCACGACACGCATGGTGTGAGCAGGAGAGAAAGAATCGCAGCATGGTCGCGGTGCGACGAAGCGTCCAAGAAAAATGCTGCGGCGCCTTGATGAAGGAGACCGGACAGAACAAATAAAAAATTCCACGCATCAGTGTGGCTGAGCTCTAGGACACCACAAAAATGCGTAACGCGTGGCGCTAGAAACACAGCCGGATACACGCCGCCTACATTTGCATGCAGCTAAGACCGAACAGCCCTAAAAAAAGTTCAGCTGCGACAAGCGGCCGGACGAGAAAAGAAAAGTAGCGTGATTCCTTGGCGCGCAGCGAAAAAGAAAAAAATAAATCTGCGCGAGCGGAAGTAAGTAGGACGGGGAAGAGAAAAAATCCACTTGAGCCACCAAAGAAGGAGCGGCCGGTAAAAATATTGTTGCGGCCCGAAAAAAGGTCGGTAGACTGCGGCCGGTAAAAAAAAAAGTAAAAAATTTCACGCCCGATTTGTCGGCTATTGACAAAGTGGAAAGCGACCAAGTCAGCGAAGAAAAAATCTTGCTGGCGACATGCTGGCGATTCATGAAAATCACCATGCGACCTTCTTTGCGGTCGACCTTCCGTGACAGGCTCGCGTTAAATTAGTGACCGGTCAGCGGTTACATCGCAGTGCAGTTCTTCAGATGTTCCTCTAAGTGCGCTGATGTTGCTAACCAGCTGAGTTCAGCACTAGTTTCAGTATTTCTCTTTGTTTCATTCACTATTTTACGTCAATCAATGTTTTCAGAAGTTAAGATTCACGAAATCCAAGATATTTAATTCACTCTTAGAAAACAAAACCTAGTACGATCAGGGGTTTTGTGAAGATATCGACTGAATTGATTTTGGTGCTCACATGAAACAATTCGATATCTCCTTTGGCGTGGTCTCTCAGAAATGGTGTGCGATGTCAATATGTTTAGTTCTAGAGTGTTGCAGGTTGTTTGCAAGTTTTATGGCACTCTCATTGTCACACAAAGTGGAATTTTGTTAAACTCACAACCAAAATCTCTAAGGGTTTACTTCATCCATAACAAGCTGTACAACATGCCCCTACTATATGCCCTCGCTTCTGGAAAAGTGCAAACAATTTTGTTTCTTGGAACTCCATAATACTAAGGATCGCCAAGGTGTTGAGTCAGATGGTTTTCGATCTACTTTGCACCGCATAATCTAGTCGAGAATAGCCAAGTAAATCGAAAGGAGCCTTTGGGATACCATAATCCTAGGTTTTAGGTGTGAACTAAGTATCTTAGAATCTTCTTGCTACAAGATGGCAATCTTTAGGATTTGCTTGAAAACGTGCACACATGCAAACACTCAACATAATATCGTGTCTAGATGCACATAAGTAAACTAGTGATCCTATCATTGATCTATATAATGTTTGATCTACTGTTTACCTTCCTCATTTAGTAGAGATGTCCATTTGATGACATTGAGTCTTGGCGTGTTTGCCTTTTCCATGCCAAATTTCTTGAGCATATCTTGTGTATATTTGGTTTGGCATAGAAAAGTACCTTCCTTATTTGACTTGAAATCAGGAAGTATTTAAGCAGCCCATCATAGACATCTCAAACACATTCAGTATTACTTTGCTAAACTCTTCACAAAATTTTTCATTAGTACTACCAAATATAATGTCATCAACATATACTTGGCACACAAATAATTCATTGTCAACTTTTCTAGTAAATAAGGTAGAGTCGGCTTTTCCTATTGTAAACCCATTCTTAATTAAAAATTCTTTAAAACAGTCATACCAAGCTCTAGGGGCTTGTTTAAGCCCGTAGAGTGCCTTGTGAAGTAGATAAACATGATTTGGCTTCTTTGGATCTTCAAAACGGAGGTTGCTCCACATATCTCTCTCTTGTAGTGGTCCATTTAGAAATGTGACATCCATTTGGTATAGCTTGAAATCATGGTTAGTAGCATATGCAATTAATATTCTAATTGATTCTAACCTTGCTCTAGGCGCATATGTTTAGCCAAATCAAGTCCTTCCACTTGAGTATAGCCTTGGGCAATAATCGTGCCTTGTTTCTTGTTAACGCTCCATGTTCATCTTGTTTGTTCCTAAAGACCCATTTAGTCCCAATCACATTTTGTTTGGGTCTTTGGACTAAGGACAGACTTCATTAGGGTGAAGTTGTTTAACTCTCTTGCATGGCAATTATCCAATCCGGATCACCAATGCTTCTTCAACCTTAAGTGGCTCAAGAGAGGAAACAAGCAGTAAAATTCACAAAATTAGCTAGCGAGATCAGTCGTGCCCCTCTGATGCTACCCGGGATGTTGTCACTTAGGATGATCCCTTTGAATTGTATGATGGACTCTTGGATGAGGCAGCGATGGTTGTCTTTGTATTTCTCCTCCTCATCATTCACTTGATCAAGAGGTACTTCACCATCAATGCTTTCGTGTTCATCTTCTACCATTGTGGCATCCTTTGATGATTTTCTTCATGGCTTGGATGACTTGAGGTTGATGGGTTTGCTTGGGTGGAGACTTTGTCCTCACCACCTTTGCACCACATCAACAACCTCATTGGTCATCAGAAAGTTCCTTCCTCATCATCCTTTTCTTGAGGTCTCACTTCACCTGTACAGGATTTCTTTGTAAGCTACAAAGGTAGTTCTTCATTTCCTGCTGGTGACATTAGAAACATGCCCTATGACATTGAGACTCATCAAATGTCACGTCTATCGCTATTTCAACAAGACCGTGGTATTGTTTGAAAACACGATATCCATGCGCATTTGATGCATAACCAAGCAAGAAGCCCTCGTCCACTCTAGGAGCAAACTTTGAGCTCTTGACTTTCTTGTTAAGAATAAAATTTACAACCAAATACTCTAAAATAATCAACTTTAGGTTTGTTGGTAAAAAGCTCATAAATGTGATCTT

>nano2

CATTTCCCACTCATATCTTTGTGATTATATGTTGACTTGGATTTAGGAAAATTGGTTGTGAAATATGTGAGTGCCTACACTAAGCGAAAAGTCATGAAAAGAAGTGTGTGGGTACCCAAGGCTCTTAACTCTTGTAGGACCCAATTCAATTTGGGTACCTAAAAGCATAGCCTAAACTTGTTTTGCGAGTCTACTCCTACAGTGGGTCAAGTTGGGTGTTGCGGTGGATGTACAAATCACATGAAGCCGGGAAAAGACATAATTTCGCCTCATACAACTAACTCAAGAAGCACAAGAAATTGTGTTTGGAGATAGTGGCAAGAGTAAGGTGATTGGTATTGGTAAAATTCCTATCTCGACCAACAATCACTTTCAAATGTTTTATTGGTAGATTCTTTAAGCTATAATTTGTTGTCCGTTTCACAACTTTGTGGAATGGGTTATAATTGTTTATTTTGGATGTGGATGTGAAGATCCTTAGAAGGGAGGACTCTCGATTGCCTTTACAGGTCGCTTGAAGGGCAGCTTTATCTTGTTGATTTCACAACAAGTAAAGTAGCGCTGAGACTTATTTAGTGGCAAAAGTCGACAAGGGTGGCTATGGCATCACGGCTAGCCCATGTCGGTATGAGGTTTGGCCAAACTTCAAAAGGATAATCATATCATTGGACTAACAAATGTTGTATTTGAGAAAGATAGGGTTTGTAGGCGCATGCCAAGCGGAAAACAACATGAGTCCCACATCAATCAAAGAATGGTCACAACAAAGAGGCCATTGGAGCTTCTTCACATGGACCTCTTTGGACTGGGCCTACATTAGCATTAATTGGTAGTAAGTATGGTTTAGTCATTGTTGATGATTTTTCTCGATTCACACAGGTTTTCTTTTTAGTGATAAAGGTGAAACTCAAGAAATATTGAAGAAATTCATGAGAGCTCAAAATGAATTTGAGCTCAAAATCAAGAAAGTGAGAAGTGATAATGGGGCGGAATTCAAGAACACAGTGTCGAAGAATTCTTAGGAGAAAAGAGGAATCAAGCATGAGTTCTCGGTGCCTTACACTCCACAACAAAATGGTGTTGTGGAAAGAAAGAACGAACTCTAATTGAAAGCTGCAAGAACCATGTTGGATGAGTACAAGACACTGACAACTTTTTAGGCGGAGGCGGTCAACACCGCCTATCATGCAATCAACCGTCTCTCTATCTTCATAAGATCTACAAAAATGCTTATGAGCTTCTCACCGTAACAAACCTAAAGTTGATTATTTTAGAGTATTTGGTTGTAAATGTTTTATTCTTAACAAGAAAGTCAAGAGCTCAAAGTTTGCTCCTAGAGTGGACGAGGGCTTCTTGCTTGGTTATGCATCAAATGCGCATGGATATCGTGTTTTCAACAATACCACCGGTCTTGTTGAAATAGCGATAAACGTGACATTTGATGAGTCTAATATTTCATAAGGGCATGTTTCTAATGTCGCTATGAAATGAAGAACTACCTTGTGAAGCCATAAAGAAACTTCTGCAATAGGTGAAGTGAGACCTCAAGAAAAGGATGAGGAAGGAACTTTCTGGATGACCAATGAGGTTGTTGATGTGGGTGCAAAGGTGGTGAGTACAAAGTCTCCACCCAAGCAAACCCATCAACCTCAAGTCATCAAGCCATGAAGAAAATCATCAAAGGATGCCAACATGGTAGAAGATGAACACGAAAGCATTGATGGTGAAGTACCTCTTGATCAAGTGAATGATGAGGAGGAGCAAATACAAAGACAACCATCATTACTCATCCAAGAGTCCATCATACAATTCAAAGGATCATCCTTGGACAACATCCCGGGTAGCATCAGAGAGGGGTAACGACTCGATCTCGTTTAGCTAATTTTGTGAATTTTACTCGTTTGTTTCCTCTCTTGAGCCACTTAAGGTTGAAGAAGCATTGGGTGATCCGGATTGGATAATTGCCATGCAAGAGGAGTTAAACAACTTCACGGAAAATGAAGGTCCTTAGTCCAAAGACCAAACAAAATGTGATTGGGACTAAATGGGTCTTTAGGAACAAACAAGATGAACATGGTGCGATTACAAGAAACAAGGCACTATTGGTTGCCCAAGGCTATACTCAAGTGGAAGGACTTGATTTTGGCTAAACATATCGCTGCAGAGCAAGGTTAGAATCAATTAGAATATTAATTGCATATACTACAACCATGATTTCAAGCTATACCAAATGGATGTCAGAGCACATTTCTAAATGGACCACTACAAGAGAGAGTATATGTGGAGCAACCTCAGTTTGAAGATCCAAAGAAGCCAAATCATGTTTATCTACTTCACAAGGCACTCACAGGCTTAAACAAGCCCCTAGAAACTGGTATAGTGTCTTAAAAGAATTTTTAATTAAGAATGGGTTTACAATAGGAAAAGCGACTCTACCTTATTTACTAGAAAAGTTGACAATGAATTATTTGTGTGCCAAGTATATGTTGATGACATTATATTTGGTAGTACTAATGAAAAATTTTGTGAAGAGTTTAGCAAAGTAATGAGCGAGTGTTTGAGATGTCTATGATGGGCGAGCTTAAATACTTCTGGGATTTCAAGTCAAACAGCTCAAGGAAGGTACTTTTCTATGCCAAACCAAATATACACAAGATATGCTCAAGAAATTTGGCATGGAAAAGGCAAACACCAAGACTCCAATGTCATCAAATGGACATCTCTACTAAATGAGGAAGGTAAACAGTAGATCAAACATTATATAGATCAATGATAGGATCACTAGTTTACTTATGTGCATCTAGACACGATATTATGTTGAGTGTTTGCATGTGTCGCGTTTTCAAGCAAATCCTAAAGATTGCCATCTTGTGCAGATTAAGATTCTAAGATACTTAGTTCACACTAAAACCTAGGATTATGGTATCCCAAAGGCTCCTTTCGATTTACTTGGCTATTCTCACAGTTATGCGGTTGCAAAGTAGATCGAAAAGCACTCTGGGACTTACCAATTCCTTGAGGCGATCCTTAGTATTATGGAGTTCCAGAAACAAAATTGTGTTGCACTTTCCACTGCGAAGTAGTACATGGTAGCATATTGTACGATTGTTATGGATGAAGTAAACCCTTAGAGATTTTGGTTGTGAGTTTAACAAAATTCCACTTTGTGTGACAATGAGAGTGCCATAAAACTTGCAAACAACCACCGTGCAACACTCTAGAACTAAACATATTGACATCGGACACCATTTCTTGAGAGCGAAGCCAAAGGAGATATCGAATTGTTTCATGTGAGCAAGAAAATCAACTGGATATCTTCACAAAACCCCTCGTCGCAGTAGGTTTTGTTTTCTTAAGAGTGAATTAAATATCTTGGATTCTCGTAACTTAACTTAGCGGTCACAAAATTGTTTGATTCACTATTTTTGATTGATAGTTTTAAAGCTATGTGATGATCTTTCAAAAAACTTGGTGAAATGTTAAATTCGAATGCATTTTAGTGCTAAGATTCTTTTTGGGCTCAATGGCTTGACCGGTGAACTTCTAGTTCAATTTGGAGAACAGTGAACTTAGAGCCGAACTTCAGCTGTTTGTTTTAACCGGTTTTTGGGACCATCCTAGTTAAACCGGTTGAGCAGGCATGGTGGACCCGGTTGAACCGTCTCTTGGTCGACGCGCAGGCGCTGTTCACGAGTCGCCACCGGCAATGATGTCGAGCGTCGTGAGATTTTTTTAACCGGTCACATCTGGCGTACGCGATCTTTTCTCTATTGACCGGTCGCCTCCTTTTGGCCGCGACTGGCGTGAAATTTTTACTGCGGTCTGTATCGGTGCACTACCCACCGATCGCGCAACAATATTTTTGCGGTCGCTTCTCTTTGGTGGCTGGCGTCAGATTTTTCTCTCTTTCTGTCCGGTCCTACTTCCCTCTGTCTGGCGCTTGATTTATTTTTCTTTTCTTCTTTGCCAGCTCGTGAAGTCTGCCTACGCCCTTTTTTCGTCTGTCATTCTTGTGTTGGCGCCAGAACTTTTTTTAGGTCTGTCTAGCTTTGCATGCAAATGTCGAGGCGCCATCTGATATCCGTTGTGTTCTAGCGTCAATCGCGTATGCATTTTTGTGGTGTCCAGAGTAAGCAGTGCTGCATGCGAGATTTTTTATTTGTTCTGTCAGGTCCTTCTCCTCTCTTGGCGCGCAGCATTTTCTTGACGCGTTTACCTCATCACCGCGCACCATGCGCAGATTCTTTCTCTCCTGCTCCACACCATGCATGCGTGTCGAGGCGTAATTTTCTTTCATCCGATCGCTTCCAGTGAATGCGCGCAGTTTTTAGCCCCACACTATGCTGTAGCCTTTTTACTCTTGTGACCCGATCATTTGTCACCCGCGAAGACAAAAATATTTTTGTCACTTCCGTGCAGCGGTGTGAGGATACAGCGCCGTGAGAACTTCTTTCTCGCGCAGTTCTTTTCTTTTGCCGTCGGTCTCATCTGCAAACACTTGGTTTTGATTTTTTTTCATAGAATCTCCATGATACTTTTTTTTGAATCTGCGTCAGTTTGATTTTTGGCATCTTCCTCTGCTTTGTTTACGGTTCTCTGACCACCTTTGGTTAGAACCAGTGAAGCCCAGCCCATGGGCGGTGGCTGGTCTGAGCCGGCTCAGCTGCCTATATATGACTCTCCCTTCTCTTCTCCCACTTCATTCACTCTTGCCTTGGTCGCTTCACAACCTTTGAGCCACTCTTTCCACACCTCAACGGTTCACGGTTCACCACCTTTTTCAATCATGGTGCGTCGTCGCAACCCCTCGTGATTGAGTTTAGCAGCGACTCAGGTGAGATTCGGAGGAATCTCCCATCCTCTCTCGCTCGTCGCTCCCTCTCTAAGAGAGGGCGTGGCTCACGTGGATGGATGGTGAGGCTCTTCCATGCCCTCGCGGCCGACTTCGCTCGAGACGCGCAAAGGTTGGCGCCTCTCATCCTCGTCGTAACGTCTTCCTTCGGTATGCTCCTGTCGGAGGAGGATGGGCGTTCGGCGACTTCGATGACTTTCCGGCGCTGATGCTCTCGTCCGCCGGATCGCTGTGCATCCGTGACGTCGGTAGTGGCCCGCATGAATCTCCCGTCGACTTCACTCACGAAGGAATAGACACCTTGGCGCTGAGGTTCACCAACCCTACTCTCCTCCTCGTCATCACGGTGTCGGGACCCTCGATTCTGGAATCTTTTCGGTGACTTCTACAGTCTGTCATTCTTTCCAAGAAACACCCAGTCGTCCAACATAGATTTATTGTGGGAGGGTTGTGAGAATATGGAGATCTTGACATGTGCGCCGGCTCTCGTAAACAGAAAGGGGTTCTAAACATTATGACCATGGAATACCTAGAGCGATGAGGTGGTAGCTCGATTCTATGCCACCCTACGGATAAAGAAAGTAGATGAGGAAGCCGATGAGTATGATTACCCGTCATGTATTTCTTCATCCGGGTAACGCACAAAGTCATTATCGTCGTTTTGCCATATTCAGGCTTCTCACGAGATATTGAAGCGGCAACATGCAGTGCATGACATTCGGCCCATGCGGAAAGACAGATTCATTCATGTCTCTCTGTGAGGAGTTGGACAACGGCTAACATGCAGGTACTCGGTACCTTAATTCTTTGTCGGATGACCGTTCTCCGAAGAGCGGAAATCAAATAAACGCCCTTGGGAGAGTCGAGTCTTCCTCCTCCTCCTTACTCCAAACCGCGCATCAATGTGTTTGACATGATGCAGGAAGAGATCGTCAGCTTCTCCGGTCTCCTCTCGGAGGTGTCTTCATGCACCCTTTATCATGAATGATCAGTGGTGCAGACCCACTTGAAAATCAAACACTCCTTCGTGCCCTGCGGGTTGATTCCTCCAACCCAGTGCCGTCGGCGGGCCCCCTCATGATGCCCAGGCCTGCCTCCTCCCTTTGAGCCACCCGCCCGGCCTCCCCATCATCCATCCTCCTCTCCCCCATTTGCGCCTCGCGTCCTCTCTCTCGCTGGATCTCCCATGCCACGTGCTCGTGGTCATGGTCGAGTGGCCCGTGGGATGGTGCTCGCTTGTCCATGGGTTTGCGGCATTTTCTCCATGTGGCGCAACATTTTGCTGATGTCCATGAGGTGTACAGCGTCGACAGGCGGCGACAATCGACATCGTCAAGCTTCTCCATGGGCATGCCTTTTGTCCTCGTTCGCCGATGTGCCTCTCCATCCTCCTCCGGAGATCAATGAGTGGCACCGGCCAGTCTCTGAGTGCCTTTATGTCGTAGACGATGAAGATGAAGAAGTCTATGTTGATGATCGAGAGTTTGCCCCCGCCTCCCTACCATGGGGATCCGGGTCAATCATCCTCCCACCTCCGCCTCCTTACCGGGTCCGGACCCATTCTGGTGACCCTAGGCCATCCGGCTCGTTACCACCACCCCTCCACCTCCTCCCGATCATCATTGCATCTTCGATCTGCTTTTTTAGGTGATAGCCACCTCGGACTCCACTTTTGGGAGGACGCGATGAGACTTTTCCCGACTACACTTCATCCTCCGTCATATCCTCCTCCTGAGGATATTGATTCTGTATCCTTGTCTCTCTCTCGTTTTGGTACTTGTTGCCAAGGGGAGAAATCCAACTTCTAGTTTGTGGTTCTTCGTTTTCTTTTGGATTATTTTTGTTTTGGCCCGTTGTGGCTTGTACCCTTTACCATTGTATGAAATAAAACTGTTGGTTGTTATTCAAAGTAATATGATGGTCTTCGCATCATCTTTTGTCCTATCGATTTGTAATGTCTACTCGCTATGAAGTAAAAATAATGGTTTTTAGAGATAGTCATTGCATCACAACTCTGTCATGACACTCTTGCACCCTCGGATAATTGTATTTAGTATGGTTATTGTCACTATCTCTAAATGTGTTGCTCACATGACATATTATGTCAAGGCGTTGGATTCACAATCGTGCACACATTTAGAATATCTACATACAATCATTCAAAAAAATTTTCTATTGCAAATATATATCTTTTGCCTTAATTGTTTTGCATCAATCACCAAAGGGAGATTGTAAGTGCAATCAACAAGTAGGAGTTTTGGTGATTTAATGACAAAACAAATAAGAGTACTAACAATTTTGTTCTCAGTGTTTGATTAGAAGGAAAGCAGAACTTAAGGAAGCACCAAGGACTTCATGCAACATGACGATATAAAATTTATGTAAAATCTTGTGTTGCATGATGTAATTCTTCCTTGTTCTTTTCCTGCAATGCACCAGTGTTTGCTATAGCTCAAGTCCTCTAATTCAAAAGCTATTTTTGAAAACCAAAAATCTCTTTAACATTATTTGGTGACCATGGTTTGGGTTGAGAACCTAGAGTGTTCTTCTTTGAAAGCAATGAACTTCTTCAGCTACTTGGTGAGCTTTCCCGGTGAACTTAGGAGTTGTGATGAGCTTCTTCAATTGCGGTGAGCTTCAAATGGTGGACTTCATGAGTTGAACTTTCAACTCCCAACTTCAACTAACTCATTGTGGACCTCGACCATACCGGCCGAACTTGCCCTGGCGGTGAGCTAGGGTTTCTCTCAAAACTCTGGTCTAGACCACCGACCGGTCTGAGGTAGCTCAAGCCGTCTCGACCCTCGACCTCCGATCTGATCTGCCGGTCGGGGCGATCAGTGCGCCGCGGGAGGTGTCGGCGGTTCAACTTAGGGGCGGTTCAACCGATCCGGCTCGTCTGACACGCGCAAGTCGATGCACTGGATCCCGATGCCGCAGTCAGGTGGCGGACGATCTATGACTTTGTCGGCGGTTCAAAGCCCTAGGGGCGTTCAACGGTTTTCAGGGGTTTTTGGTCAAGCGGCTAGTTTTTGAGCCGTGACTATAAATAGACCCCTCTCTCTTCTTCAAGGCAATAACACTCACTCATTTATTGCTCACCTAACTTGAGACAAAGCACTCATCTCTCCATCTCTCTCACTTAAATCTTCCTCAAGATTCAACCTTGTTGGAGGAGCTCTTGGGTGTGAGGTTTGTGCTTGTGCTCACGATTTGGTTTCCTCTCCCATCTCTTTTTCAAAGACTTGAGCTTGTGCAAACCCCCTCTTGTATGCGTTCTTGGTGTTCTTGAAACCCTAGGTTTCAAGATCTTCGAGGTTGGGAGTCTCCAAATTTGTGGGCGACCCTAAGAAGTTGGTATCACCGCTCTTTGAGCTAAGATAAGAAGAGATTGCCTTGACCTTTGTGGTCGGCTTTGGAGGATTAGGGTTGAAAAAGACCCGCCCTTTGTGGGCTCCTCAGGGAGTAGGACACCTTTGTGGTGTGGCCAACCTCGGATTAAATCTTGTGTCTTGTGTTCTTACTTGTTTCAACTTGAGATATTTTGCAAGATTGACATAAAGATTTCTCCGTAGAGTTTATCTAGAAGTAAAATAGCCTTTACATATTCATCTTTTCATCCTCACTTAGCTATATCTTACTTCTCTCAAGAACTTGCGAAATACTTTCTGAGTAGATTTTAGTCTAAAGTTTATAAACGATTGGATTGGTTCGAGTGGTTCAACTTGCGCTGCGTAGGCATTGAGCCGAACTGCACCACCCCATGTATATAATAATCTTCAAGTTTGTTGTGAAAATTTTCAGATTAGCCTATTCACCCCCTCTGGGCTACTTTCACTACTAGACTGTCCGACAAGCCCATCACCTTCGACCAAGCGACCACCCGACCACGTCGAGCAGGGAAATACCGCTTCGTTGTCGACCCGTCATCGGTGACGTCCAGGCTCACCAAGGTCCTCATGGACGGAGGCGGCAGACCTCAACATCATCTATCTTGAGACCCTGCCTCCGTGTTGATCTATCCTCGATTCGGGCGAGGCGCTGCGCCATTCCACGGGATCATCCCGGAGCGTCGGCCCCTTCGGACAACTCGACCTTCTGTGCGGCGGAACACCCTCCAACCGAGGGGGACCCTCACGTTGGTGTGGTGGGGTTCGAGGGCCTACCACGCGTCTTGGGAGGCCATGCTACGCGGTTCGCGGCCGTCCCCAACTACACCTACCTCAAGCTCAAGATGCGAAGATTTGCAGTCGTCACAGCGTTGCGACGTAAACACGCGTTGCTGCCGACGTCGCGGAGTGGTGCCGGAAGAATGCATCAGAACCTCGGCGTCCGCTCGCCTCTATGAGAGGTGCCGGACGTGAAGCGCCACGCCGACAACGAGCGCGAGCGGTTAAGTCCGTCCCCTCGACCCAGCAGGCCGACGCCTCCAAGCGGATCCGGATCGGCTCGAGCTCGATCCCAAATAGGAAGCGATGCTGCGTGGAAACAAGCGCCGCGCGACGCTGCGCGTGAGTCGGACATGCCCGTATACCGAGGATGTCGCCGAGCACTCGCTGATATCCGAGTTGGAGCCCGCAGTGAAGCGGCCTCTGCCTTCGATGAAGAAAAGCGCAGAGCCATGGCGAGAGAGATCCACACAGCTAATGGCAGCAGGTTCATCAAAGAGGTATTCCATCCCGAATGGCTTGCCAACCACGTTCTTGTGAGAAAGGAGGAAGGGGCGGATGTGTGTAGACTACTTTGGTCTAAACAAAGCATGTCCGAAGTTCCCTACCCTACCTCGCATCGATCAAATTGTGGATTCCACCGCGGGTGCGAAACCTTGTCTTTCCTCGATGCCTACTCGGTACCACCAAATCGGGATGAAAGAATCCGACACCGCGACTTCTTTCATCACACCCTTCGGCATATACCGCTATGTCACCATACTGGTTCGACAGGAATGCGGGTCGACGTGCAGTGATTGCATAGAACCATGTGTTCGGCGAACACATTGGCGACGGTCGAGGCCTACGTCGATGACATCGTGGTCAAGACGAGGAAAGCCTCGGACCTCCTTTCCGACCAGTGACATTCAGATGTCTCAAGGCGAAAGACGTGAAGCTTAATCCGAGAAGTGTCTTTGGGGTTCCTCGAGGCATGCTCTTGGGGTTCATTGTCTCAGTGGGGCATCGAGGCTAATCCGAGAAGATCGCGGCCATCACGGCATGGGGCCCATCAAGGACTTGAAGGCGCTGAGAGTCACGGATGTCTTGCGGCTCAGCCGCTTCATCTCACGCCGCCGAAACTGCCTCTATCAGCCTCTTAAGGAAGGGCGTGCTTCACTTGGACCCCGAGGCCGAGAAGCCTCGGAACCTCAAGGCGCTCTCACGAACGCGCCCATCTTGGTGCCCCTGCTGGAGAAGCCTATTGATCTACGTCGCGCGACCACTCGGGTGCTTAAGCGCGCGATCGTGGTTGAGAGACGAGAGGGGCATGCATTGCGGCCGAGAGGCAAGTCTACTTCATCGGTATCTTGTCGAGACCAAGATCCGCTACCACAAATTGAGGAAGGCTGCACGCGGTGATCACGCGCGGAAGTTGCGACACTACTTCGAGTCTCATCCGATGCCTGTGGTGTCATCCTTCCGGGAGAGATCATCTAGTCGCCGAGGCCTCGGTAGAATCGCAGCGTGGCGGTGGAAATCATGGGCGGGCGATCTCGCTCGCCCTCTGGAAGGCCATCAAGTCCTGTGTTCGCGGACTTGTGGTGAATGGGTCGACACCGGCTCCGGGCCCGATCCAACCGGACTCGACCATGTTCTTCGACGGGCCGATGAAGACGGGCCGCGCGAGCCTCTTTCTCATCTCGCCTCGAAAGCACCTCGCTATGTGCTACGCCTCCCTTCGTCGAACAATGTGGTAGTCGGGCTCAGTCGCGGAAGCATCGCCATCGGTGAAGTCCGGGCCGACGGCCGCCGTGGTGACTCGCGACTCGTCATCGACCAAGTCATGAAGAACTCCCATGCCGCGACCAGAAGATGGAGGCTCGCTGCTGACGTTCGGTGCTCGGAAGACAAGTTCTCGGGGCTCGAAGCTCAAGCCACATCGCCGGCCTTCTAGCGGTGATAGGTTTAAACTGAAGGCGGAAACGAGCAATCTGATCAAGCTCAAGCTGCTCTAGCATTCGCCATTCAGGCTGCGCAACTGTTGGGAAGGGCGATCGGTGCGGGCCTCTTCGCTATTACGCCAGCTGGCGAAAGGGATGTGCTGCAAGGCGATTAAGTTGGGTAACGCCAGGGTTTTCCCAGTCGACGTTGTAAAACGACGGCCAGTGCCAAGCTTGCATGCCTGCAGTGCAGCGTGACCCGGTCGTGCCCCTCTCTAGAGATAATGAGCATTGCATGTCTAAGTTATAAAAATTACCACATATTTTTTTGTCACACTTGTTTGAAGTGCAGTTTATCTATCTTTATACATATATTTAAACTTTACTCTACGAATAATATAATCTATAGTACTACAATAATATCAGTGTTTTAGAGAATCATATAAATGAACAGTTAGACATGGTCTAAAGGACAATTGAGTATTTTGACAACAGGACTCTACAGCTTTATCTTTTTAGTGTGCATGTGTTCTCCTTTTTTTTTGCAAATAGCTTCACCTATATAATACTTCATCCATTTTATTAGTACATCCATTTAGGGTTTAGGGTTAATGGTTTTATAGACTAATTTTTTATTATCTATTTTATTCTATTTTAGCCTCTAAATTAAGAAAACTAAAACTCTATTTTAGTTTTTATTTAATAATTTAGATATAAAATAGAATAAAATAAAGTGACTAAAAATTAAACAAATACCCTTTAAGAAATTAAAAACTAAGGAAACATTTTTCTTGTTTCAGTAGATAATGCCAGCCTGTTAAACGCCGTCGACGAGTCTAACGGACACCAACCAGCGAACCAGCAGCGTCGCGTCGGGCCAAGCGAAGCAGACGGCACGGCATCTCTGTCGCTGCCTCTGGACCCCTCTCGAGAGTTCCGCTCCACCGTTGGACTTGCTCCGCTGTCGGCATCCAGAAATTGCGTGGCGGGCGGCAGACGTGAGCCGGCACGGCAGGCGGCCTCCTCCTCCTCTCACGGCACCGGCAGCTACGGGGTTCCTTTCCACCGCTCCTTCGCTTTCCTTCCTCGCCCGCCGTAATAAATAGACACCTCCACCTCTTTCCCCAACCTCGTGTTGTTCGGGCGCACACACACAGCAGATCTCCCCAATCCACCCGTCGGCACCTCCGCTTCAAGGTACGCCGCTCGTCCTCCCCTCTCTACCTTCTCTAGATCGGCGTTCCGGTCCATGGTTAGGCCCGTAGTTCTTCTGTTCATGTTTGTGTTAGATCCGTGTTTGTGTTAGATCCGTGCTGCTAGCGTTCGTACACGGATGCGACCTGTACGTCAGACACGTTCTGATTGCTAACTTGCCAGTGTTTCTCTTTGGGGAATCCTGGGATGGCTCTAGCTGTTCCGCAGACGGGATCGATTTCATGATTTTTTTGTTTCGTTGCATAGGGTTTGGTTTGCCCTTTCCTTTATTTCAATATATGCCGTGCACTTGTTTGTCAGGTCATCTTTTCATGCTTTTTTTCTTGGTTGTGATGATGTGGTCTGGTTGGGCGGTCGTTCTAGATCGGAGTAAATTCTGTTTCAAACTACCTGGTGGATTTATTAATTTTGGATCTGTATGTGTGTGCCATACATATTCATAGTTACGAATTGAAGATGATGGATGGAAATATCGATCTAGGATAGGTATACATGTTGATGCGGGTTTTACTGATGCATATACAGAGATGCTTTTGTTCGCTTGGTTGTGATGATGTGGTGTGGTTGGGCGGTCGTTCATTCGTTCTAGATCAGGTAGAATACTGTTTCAAACTACCTGGTGTATTTATTAATTTTGGAACTGTATGTGTGTGTCATACATCTTCATAGTTACGAGTTTAAGATGGATGGAAATATCGATCTAGGATAGGTATACATGTTGATGTGGGTTTTACTGATGCATATACATGATGGCATATGCAGCATCTATTCATATGCTCTAACCTTGAGTACCTATCTATTATAATAAACAAGTATGTTTTATAATTATTTTGATCTTGATATACTTGGATGATGGCATATGCAGCAGCTATATGGATTTTTAGCCCTGCCTTCATACGCTATTTATTTGCTTGGTACTGTTTCTTTTGTCGATGCTCACCCTGTTGTTGGGTGTTACTTCTGCAGGTCGACTCTAGAGATCCAACAATGGACAACAACCCCAACATCAACGAGTGCATCCTACAACTGCCTGAGCAACCCGAGGTGGAGGTGCTGGGCGGCGAGCGCATCGAGACCGGCTACACCCCCATCGACATCAGCCTGAGCCTGACCCAGTTCCTGCTGAGCGAGTTCGTGCCCGGCGCCGGCTTCGTGCTGGGCCTGGTGGACATCATCTGGGGCATCTTCGGCCCCAGCCAGTGGGACGCCTTCCTGGTGCAGATCGAGCAGCTGATCAACCAGCGCATCGAGGAGTTCGCCCGCAACCAGGCCATCAGCCGCCTGGAGGGCCTGAGCAACCTGTACCAAATCTACGCCGAGCTTCCGCGAGTGGGAGGCCGACCCCACCAACCCTGCCCTGCGCGAGGAGATGCGCATCCAGTTCAACGACATGAACAGCGCCTGACCACCGCCATCCCCTGTTCGCCGTGCAGAACTGGGTGCCCCTGCTGAGCGTGTACGTGCAGGTGCCAACCCACCTGGGCGTGCTGCGCGACGTCAGCGTGTTCGGCCAGCGCTGGGGCTTCGACGCCGCCACCATCAACAGCCGCTACAACGACCTGACCCGCCTGATCGGCAGCTACACCGACCACGCCGTGCGCTGGTACAACACCGGCCTGGAGCGCGTGTGGGGTCCCGACAGCCGCGACTGGATCAGGTACAACCAGTTCCGCCGCGAGCTGACCCTGACCGTGCTGGACATCGTGAGCCTGTTCCCCAACTACGACAGCCGCACCTACCCCATCCGCACCGTGAGCCAGCTGACCCGCGAGTTTACACCAACCCCGTGCTGGAGAACCCGACGGCAGCTTCCGCGGCAGCGCCGGGGCATCGAGGCAGCATCCGCAGCCCCACCTGATGGACATCCTGAACAGCATCACCATCTACACCGACGCCCACCGCGGCGAGTACTACTGGAGCGGCCGCAGATCGCGGCCAGCCCCGTCGGCTTCAGCGGCCCCGAGTTCACCTTCCCCTGTACGGCACCATGGGCAACGCTGCACCTCAGCAGCGCATCGTGGCACAGCTGGGCCAGGGAGTGTACCGCACCTGAGCAGCACCCTGTACCGTCGACCTTTCAACATCGGCATCAACAACCAGCAGCTGAGCGTGCTGGACGGCACCGAGTTCGCCTACGGCACCAGCAGCAACCTGCCCAGCGCCGTGTACCGCAAGAGCGGCACCGTGGACAGCCTGGACGAGATCCCTCAGAACAACAACGTGCCACCTCGACAGGGCTTCAGCCACCGTCTGAGCCACGTGAGCATGTTCCGCAGTGGCTTCAGCAACAGCAGCGTGAGCATCATCCGTGCACCTATGTTCAGCTGGATTCACCGCAGTGCCGAGTTCAACAACATCATCCCCAGCAGCCAGATCACCCAGATCCCTGACCAAGAGCACCAACCTGGGCAGCGGCACCAGCGTGGTGAAGGGCCCCGCTTCACCGGCGGCGACATCCTGCGCCGCACCAGCCCCGGCCAGATCAGCACCCTGCGCGTGAACATCACCGCCCCCTGAGCCAGCGCTACCGCGTCCGCATCCGCTACGCCAGCACCACCAACCTGCAGTTCCACACCAGCATCGACGGCCGCCCCATCAACCAGGGCAACTTCGCCACCATGAGCAGCGGCAGCAACCTGCAGAGCGGCAGCTTCCGCACCGTGGCTTCCACCCCCTTCAACTTCAGCAACGGCAGCAGCGTGTTCACCCTGAGCGCCCGTGTTCCAACAGCGGCAACGAGGTGTACATCGACCGCATCGAGTTCGTGCCCGCCGAGGTGACCTTCGAGGCCGAGTACGACCTGGAGAGGGCTCAGAAGGCCGTGAACGAGCTGTTCACCAGCAGCAACCAGATCGGCCTGAAGACCGACGTGACCGACTACCACATCGATCAGGTGCGAGGCCCAGGAAGGGTGGAGGAGCTTCCGTGCTCAACTCCGGACGCACCACCATCTGCGACGGCCACAACGTGATGGCCCAGGACCCGTTCTCCTTCAGTACAAGTCCTCGACACCATCCAGAAGGAGTGGACCGAGTGGAGAGCGCGACGACCACTCACTCTACGTGGCTCCTGTGGTGGGTACTGTGGCCATCTTCCTCCTCAAGAAGCTCGGAGGCTTCGCTGCCAAGCGCATCCTCAATGGCCTCTGGGACCTCGTGTTCCCAAACGACAACACCAAGCTCATGCAGGACATCCTCCGTGAGACCGAAGTTCCTCAACCAGCGCCTCAACGCTGACACCCTCTCCCGCGTGAACGCCGAACTTGAGGGCCTCCAGAAGAACGTTGCCGAGTTCAACCGTCAGGTGGACAACTTCCTCCCCGAACCGTAACCCGACTCTCCTCCATCATCCCGGTGAACACCATGCAGCAGCTCTTCCTCAAGCCTCACCCAGTTCCAGCTCGCGGCTACAGAACTCCTTCTCCTTCCACTCTTCGCTCAGGCCGCCAACCTCCACCTCTCCTTCATCCGCGATGTGATCCTCAACGCAGAGGAGGGTATCTCCGCTGCCACTCTCGCACCTACCGTGGCTACCTCAAGGACTACACCAAGGAGTACTCCAACTACTGCATCAACACCTACCAGACAGCCTTCAAGAAGCTCAACACCCTCTCCACGAGATGCTTGAGTTCCGCACCTACATGTTCCTCAACGTGTTCAGTACGTGTCCATCTGGTCCCTCTTCAAGTACCAGTCCTCCTCGTGTCCTCCGGCGCCAACCTCTACGCCTCCGGCTCCGGCCCGCAGCAGACCCAGTCCTTCACCTCCCAGGACTGGCGTTCCTCTACTCCTTCCAGGTGAACTCCAACTACGTGCTCAACGGCTTCTCCGGCGCCCGCCTCTCCAACACCTTCCCGAACATCGTGGGCCTCCCTGGCTCACAACTACCCACGCCCTCCTCGCCGCTCGCGTGAACTACTCGGTGGCATCTCTTCCGGCGACATCGGCGCTTCCCGTTCAGCCAGAACTTCAACTGCTCTACCTTCCTCCTCCGCTCCTCACACCGTTCGTGCGCTCCTGGCTCGACTCCGGCTCCGACCGCGAGGGCGTGGCTACCGTGACCAACTGGCAGACCGAGTCCTTCGAGACCACCTCGGCCTCCGCTCCGGTGCCTTCACCGCACGCGGCAACTCCAACTACTTCCCGGACTACTTCATCCGCAACATCTCCGGCGTGCCGCTCGTGGTGCGCAACGAGGACCTCCGTCGCCCTCCTACAACAGATCCGCAACATCGCCTCCCCGTCCGGCACCCTGAGGTGCACGCGCTTACATGGTGTCCGTGCACAACCGCAAGAACAACATCCACGCTGTGCACGAGAACGGCTCCATGACCCACCTCGCTCAAACGACTACACCGGCTTCACCATCTCCCGATCCACGCTACCCAGGTGAACAACCAGACCCCGCACCTTCATCTCCGAGAAGTTCGGCAACCAGGGCGACTCTCTCCGCTTCGAGCAGAACAACACCACCGCTCGTTACACCCTCCGCGGTAACGGCAACTCCTACAACCTCTACCTCCGCGTGTCTTCCATCGGCAACTCCACCATCCGCGTGACCATCAACGGCCGTGTACACCGCCACAAACGTGAACACCACCACAAACAACGACGGCGTGAACGACAACGGTGCTCGCTTCTCCGACATCAACATCGGCAACGTGGTGGCCTCCTCCAACTCCGACGTGCCGCTCGACATCAACGTGACCTCAACTCTGGCACACAGTTCGACCTCATGAACATCATGCTCGTGCCGACCAACATCTCCCCGCTCTACTAAGAGCTCTAGATCTGTTCTGCACAAAGTGGGTGAGTCAGTCATCGATCAGGAACCAGACACCAGACTTTTATTCATACAGTGAAGTGAAGTGAAGTGCAGTGCAGTGAGTTGCTGGTTTTGTACAACTTAGTATGTATTTGTATTTGTAAAATACTTCTATCAATAAAATTTCTAATTCCTAAAACCAAAATCCAGGTACTGAGGCTCGAATTCGTAATCATGTCATAGCTGTTTCCTGTGTGAAATTGTTATCCGCCACAATTCCACACAACATACGAGCCGGAAGCATAAAGTGTAAAGCCTGGGTGCCTAATGAGTGAGCTAACTCACATTAATTGCGTTGCGCTCACTGCCCGCTTTCCAGTCAGGAAACCTGTCGTGCCAGCTGCATTAATGAATCGGCCAACGCGCGGGAGAGGCGGTTTGCGTATTGGCTAGAGCAGCTTGCCAACATGGTGGAGCACGACACTCGTCTACTCAAGAATATCAAAGATACAGTCTCAGAAGACCAAGGGCTATTGAGACTTTTCAACAAAGGGTAATATCGAAACCTCCTCAGATTCCATTGCCCAGCTATCTGTCACTTCATCAAAAGGACAGTAGAAAAGGAAGGTGGCACCTACAAATGCCATCACATTGCGATAAAGGAAAGGCTATCGTTCAAGATGCCTCTGCCGACAGTGGTCCCAAAGATGGACCCCCACCCACGAGGAGCATCGTGGAAAAAGAAGACGTTCCAACCACGTCTTCAAAGCAAGTGGATTGATGTGAACATGGTGGAGCGACACACTCTCTCCAAGAATATCAAAGATACAGTCTCAGAAGACCAAAGGGCTATTGAGACTTTTCAACAAAGGGTAATATCGGGAAACCTCCTCGATTCCATTGCCCAGCTATCTGTCACTTCATCAAAAGGACAGTAGAAAGGAAGGTGGCACCTACAAATGCCATCATTGCGATAAAGGAAAGGCTATCGTTCAAGATGCCTCTCTGCCGACAGTGGTCCCAAAGATGGACCCCACCCACGAGGAGCATCGTGGAAAGAAGACGTTCCAACCACGTCTTCAAAGCAAGTGGATTGATGTGATATCTCCACTGACGTAAGGGATGACGCACAATCCCACTATCCTTCATAAGACCCTTCCTCTATATAAGGAAGTTCATTTCATTTGGAGAGGACACGCTGAAATCACCAGTCTCTCTACTAAATCTATCTCTCGACGCATGCCTACAGTGCAGCGTGACCCGGTCGTGCCCTCTAGAGATAATGAGGCATTGCATGTCTAAGTTATAAAAATTACCACATATTTTTTGTCACACTTGTTTGAAGTGCAGTTTATCTATCTTTATACATATATTTAAACTTTACTTCTACGAATAATATAATCTATAGTACTACAATAATATCAGTGTTTTAGAGAATCATATAAATGAACAGTTAGACATGGTCTAAAGGACAATTGAGTATTTTGACAACAGGACTCTGTTTTATCTTTTAGTGTGCATGTGTTCTCCTTTTTTTTGCAAATAGCTTCACCTATATAATACTTCATCCATTTTATTAGTACATCCATTTAGGGTTTAGGGTTAATGGTTTTATAGACTAATTTTTAGTACATCTATTTTATTCTATTTTAGCCTCTAAATTAAGAAAACTAAAACTCTATTTTAGTTTTTTATTTAATAATTTAGATATAAAATAGAATAAAATAAAGTGACTAAAAATTAAACAAATACCCTTTAAGAAATTAAAAACTAAGGAAACATTTTTCTTGTTTCGAGTAGATAATGCCAGCCTGTTAAACGCCGTCGACGAGTCTAACGGACACCAACCAGCGAACCAGCAGCGTCGCGTCGGGCCAAGCGAAGCAGACGGCACGGCATCTCTGTCGCTGCCTCTGGACCCTCTCGAGAGTTCCGCTCCACTGTTGGACTTGCTCCGCTGTCGGCATCCAGAAATTGCGTGGCGGAGCGGCAGACGTGAGCCGGCACGGCAGGCGGCCTCCTCCTCTCACGGCACCGGCAGCTACGGGGATTCCCACCGCTCCTTCGCTTTCCTTCCTCGCCCGCCGTAATAAATAGACACCCCTCCACACCTCTTTCCCCAACCTCGTGTTGTTCGGAGCGCACACACACAACCAGATCTCCCCAAATCCACCCGTCGGCACCTCCGCTTCAAGGTACGCCGCTCGTCCTCCCCCTCTCTACCTTCTCTAGATCGGCGTTCCGGTCCATGGTTAGGCCCGTAGTTCTACTTCTGTTCATGTTTGTGTTAGATCCGTGTTTGTGTTAGATCCGTGCTGCTAGCGTTCGTACAGATGCGACCTGTACGTCGAACACGTTCTGATTGCTAACTTGCCAGTGTTTCTCGGGAATCCTGGGATGGCTCGCCGTTCCGCAGACGGATCGATTTCATGATTTTTTTGTTTCGTTGCATAGGGTTTGGTTTGCCCTTTTTCCTTATTTCAATATATGCCGTGCACTTGTTTGTCGGGTCATCTTTTCATGCTTTTTGTCTTGGTTGTGATGATGTGGTCTGGTTGGGCGGTCGTTCTAGATCGGAGTAGAATTCTGTTTCAAACTACCTGGTGGATTTATTAATTTTGGATCTGTGTGTGTGCCATACATATTCATAGTTACGAATTGAAGATGATGGATGGAAATATCGATCTAGGATAGGTATACATGTTGATGCGGGTTTACTGATGCATATACAGAGATGCTTTTGTTCGCTTGGTTGTGATGATGTGGTGTGGTTGGGCGGTCGTTCATTCGTTCTAGATCGAGGTAAATACTGTTTCAAACTACCTGGTGTATTTATTAATTTTGGAACTGTATGTGTGTCATACATCTTCATAGTTACGAGTTTAAGATGGATGGAAATATCGATCTAGGATGGTATACATGTTGATGTGGGTTTTACTGATGCATATACATGATGGCATATACAGCATCTATTCATATGCTCTAACCTTGAGTACTATCTATTATAATAAACAAGTATGTTTTATAATTATTTTGATCTTGATATACTTGGATGATGGCATATGCAGCAGCTATGTGGTTTTTTAGCCCTGCCTTCATACGCTATTTATTTGCTTGGTACTGTTTCTTTGTCGATGCTCACCCTGTTGTTTGCGTTACTTGCAGGTCGACTCTAGAGGATCTACCATGGCCACCGCCGCCGCCGCGTCTACCGCGCTCACTGGCGCCACTACCGCTGCGCCCAAGGCGAGGCGCCGGGCGCACCTCCTGCCACCCGCCGCGCCCTCGCCGCGCCCATCAGGTGCTCAGCGGCGTCACCCGCCATGCCGATGGCTCCCCGCCACCCTGCTCCGGCCGTGGGGCCCACCGATCCCCGCAAGGATCCGACGCCCTGCCCGCCACCTTCGACGTGATCGTGCATCCAGCTCGCGAACTCCGCGGCGAGCTTCGCGCTCAGCCATCCAAGAACTACACCACTCGCTGCACTCCTCGCCGCTGCCCCTCGCTGAGGGCGAGACCCGCGTGGTGGGCGTGGCTACCTCTGAGGACGCCGAGGTGCTCCGCTGCCTCCGCGACTGGGGCGCTGGCGTGGAGCTTGTGGGCGATGACGCCGTGATCCGCGGTTTCGGCGCTCGCCCACAGGCCGGTGTGACCTCAACAGGCATGCCGCGGTGGCCCGCCTCCTCATGGGCGTGGCCGCTCTCACCTCTGGCACCACTTTCGTGACCGACTACCCGGACTCCTCGGCAAGCGCCCTCAGGGCGACCTCCTTGAGGCCCTCGAACGCCTCGGTGCCTGGGTGTCCTCAACGACGGTCGCCTCGATCTCCGTGTCCGGCCCAGTGCGCGGTGGCACCGTGGAGGTGTCCGCCGAGCGCTCCTCCCAGTACGCCTCCGCCTCATGTTCCTCGGCCTCTCTCCCGGACGGACTCGAACTCCGCCTCACCGGCGACATCAAGTCACGCTCCGCTCCGCCAGACACTCTGACACCCTCTCTGACTTCGGCGTGCGCGCCACTGCCTCCGACGACCTCGCCGCATCTCCATCCCGGGTGGCCAGAAGTACCGCCCAGGCCGCGTGCTGCGTGCCGGCGACTACCCGGCTCCGCTGCCATCCTCACCGCCGCTGCCCTCCTCCCAGGCGAGGTGCGCCTCTCTAACCTCCGCGAGCACGACCTCCAGGGCGAGAAGGAGGCCGTGAACGTGCTCGCGAGATGGGCGCTGACATCGCGCGCGAGGGCGATACCCTCACCGCGCGTGTGGCCGCCCTCTCCACGCGGACTCTCGACGGCGATTCCTTCACCGACGCGTGCAAGCCCTCACCGCCGCTGCTGCCTTCGGCGGCGACACCACTGGGAGAACGTGGCCACTCTCCGCCTCAAGGAGTGCGACCGCATCCTGACACCCGCGGGCTTGAGCGCCTCGGCCTCCGCGCCGCAGCGAGACCGCCGACTCTCCGTGACCGGCTCTCGCTCCTCACTGGGTGGCATCACCGCCGCAACCACGGCGACCGCATGATCATGCTCTCACCCTCCTCGGCCTCAGCGCAGGCGCTCCACTCCGCATCACCGGCGCACACCACATCCGCAAGTCCTCCCTCGTTCTTGGCTCACCTCGAAGCCTCGGCGCCTCGCCAGTACGCTGAGCCACCGCCTAATAGGAGCTCGAGTTTCTCCATAATAATGTGTGAGTAGTTCCCGGATAAGGGAATTAGGTTCCTATAGGTTTCGCTCATGTGTTGAGCATATAAGAAACCCTTAGTATGTATTTGTATTTGTAAAATACTTCTATCAATAAAATTTCTAATTCCTAAAACCAAAATCCAGTACTAAAATCCAGATCCCGAATTAATTCGGCGTTAATTCAGTACATTAAAACGTCCGCAATGTGTTATTAAGTTGTCTAAGCGTCAATTTGTTTACACCACAAAATAGCCTCGGGGCGAACAACGGTTCCGGGCGCCTTCTCCCGAGACACCATCAACCCTCCGTCAAGATCGACGACACGTCGAGAGCCGAGGCACCTCGGCCACGCCGGCACCCTCGGCTCGATCACTGAGCCGCCGGCTCGGCAGGCACCTCGGCTAGCGCGGTACCTCGCCCCGAGGATAAGGCACCGCCGCCGAGGAGGAGCGGCTGGGTCACGCCTAATGAACCGGGACCCGTGCACGCACATCTCCACCGAGGAGCTACCTCGAGAGCCAAGCTCGTGTTATGCGCATGCCAAGTCGCTTGTCTTGGTGGGGGCGAAAGAGTTCTACCGTAGCCCTCGGCATCCTCCAGTGATGCATTTCCATCGCCAAGGCAGAGCTCCTACAGAGATACACTCGGGCTTGCACGGCCATCACGCGACACCTCAGCCCTTGTTGGAAACGCTCGCAAGGTTTCATCAAGCAGCGGTGGCCGACGCCACTAGAATTGTCCGCTGCGAAGGGTGTGTTCTCACATGACGCAGACCACACTGCCGCTCAGCCCTGCAGACGATACCCATCACACGCCTTTTCGTGGGTCTCGGATCTCGTCGCCCTTGCGTAAGGCACCAGGCTACACGCGTTGGTCACCATCGATAAATTCTCCAAGTGGATCGAGGTCCGACCCTAAACAAAGATCAGTCCGAACAAGCGGTGGCGTTCTTCACCAACATCATCCATCGCTTCAGGTCCCAGAGCCCAGAGGGGGTGAATAGGTGATCTGTAAAACTAAACTTAAGCCACAAAAACTTGTTAAGGTTAGTACAAGTATGGCCAAGTGGCTAGAGAGAACTCAAAACACACAATAACCACAAGCAATCACAGAGTTGACACGGTGGTTATCCCGTGGTTCGCCAGTACAAAACTTGCTCACGTTGTAAGCGTCCCAACTGACGAGAGTTGCACTCAACTCCTCTCAAGTGATCCAATGATCAACTTGAATACCACGGTGTCTTTCCTTTGATCTTTTCAGTTTGAGGAATCTCCACAACTTGGAGTCTCTCGCCTTATTGAGTTCACAAAGAAATACTGAGTAAGGTGGGAATGAGCAGCGCACACAAGACTCAAAATGAGCAACAACACGCACATATCGCAACAAGAGCTCGCAGCACAACACAAAGAGTTCACAACTCCACAAGAGCTCTATATGCTATCACAATGAAACGAATGCGTGATTGATGTCTTGGTGCTTAGAAGAGTTGTAGGAATGTGGTGTACTCCTCCATGCGCCTAGGGTCCCTTTTAGCGGCCGCTAGGGCCGTTAGAACAAATCTGACCATGCGCATACATGCGTGGCGGCGGGCCCGGTCCGGTCTGCTGACACCGCTGTCGTGCGATTGTTTCCTTAAATGGCGAAGCCGGCTGTTCTTAGGCCAGATTGCGGTCACAGCGCGCCGGACGGTCGGTGCACGGACGGCGTTCTTCTTCCGACCGTTGGCTCGGCCGTGTCGCGCCGTCATGCGATCGTTAACCGGCCGGCCAGTTGGCTCACGGACGATCCGGTGCTGCCAAGACGATCCGGTGAATTTTAGCCGAAATCGCGGAGAAAAACGACGGCCTCTTGGTTCGGCCAGATCACAGCGCCGGACCTTTGTCGGTGCACCACCGACATCCGGTGCCCGAGCATCGCGACCCTTGGTGTACACACCAAGTCTTCTTCTCTTCTTCTATCTTTCTAACACTTAGACAAATATATTAGTACACAAAACCAATGTACTAAGGCTTAGAAACATACCTTAACTTGTGATTTGCACTTTGTTCATCCTTGGGCATATTTTCACATTTAAGCACTTGTGTTTGCACTCAATCACCAAAATTTAGAAATGGCCCAAAGGCACATTTCCTTTCAATCTCCCTTTTTGGTGATTTATGCCAACACAACATAAAGTAGATAGCACAAGTGCAAAATCACTTCAAATAAAAACTCAAATTGGTTTTGATTCAATTTTGCATATATGGATCATCCTTTGCCACCACTTGGTTTGTTTTGCAAATCAAACTCAAATCTCTATCTCTAAGTCAAACACACATGATGAAGTATAAAGAGTCATTCCAAAAGATTGATCAAAGATTTCAAAACTCCCCTATTTCCATAATCAACACTTCTCCCACAAAAGCCAACTTTTGACAATAGAGACAATAAGAGACAATAAAAGCTTTTGACAAAACAAAACTCTATTCTACTATTTTCAAAATCTCTCAAGTGGTAGTGATCCATTTATCACTTTGGCCTATTTTCTCCCCTTTGGCATCAAGCACCAAACAGGATTAATCTTGGCCTTTAACCCCATTGCCTCACCAAAGTCTTCAATTAAGAGCAAATGGCAATAAGATTTCATGAGATGAACTTGGAATTAGTTACCTCTCATCCAGAGTCGGTGGAAGTCTTTCATGGTCCAAGTCCACCTTTTCCCTTTCAATCCTCCTTCGAGACTAAATCAAGCAAACTCAAGCAAATGGTTTCTCAAAGGTCAAGTTGTAACATCTCCCCTAAACGTGTGCATCACTTTGCAACGGACTTGTGAGGTCGAGGAGTGTTTGTACAACAGGCACCATAATAAGCAACATAATGCAAAAAGAACATGATCAAAAGCATAACTACATGTATGCTACAATTCAATCCGAGTTCCATGAATCTAAGACATTTAGCTCACTCATACTTTGCAAAGGTCTTCTCATCTAGAGCTTAGTGAAGATATCGGCTAGTGGTTTCGGTCTGCAACATGAAACACTCGATATCTCCTTTTCTGGTGGTCTCTCAAAGTGATGCTTGGATGTCTATGTGCTTTGTCTTGGTGTGTTCAGCGGGATTCTCCGCCATGCGGATAGCACTCTCATTGTCACATAGGTGGGACTTTGCTCGGATTGTAGCCAAAGTCCGAGGGTTTGCCTCATCCAAAGTAGTTGCAGCGCAACACCTCCTGCTGACAACATACTGGCCTCGGGTGGATGGGAACAGCGGAAGTTTGTTTCTTAGAGTTCCATGACACGAGGACCTTCCTAAGAATTGCACGTCCCGATGTGCTCTTCCTATCGACCTTACATCCGGATAGTCAGTCCGAGTATCCAACTAAGTCAAAGGTAGACCCTTTGGATACGAGCGAAGCAAGCGTAACAACTAAATATCTAGAATCGCTTCAGCCACTAAGTGACATTCCTTAGGATCGGATTGAAATCTAGCACACATGCATACGCTAAGCATAATATCCGTCTACTAGCACATAAGTAAAGCAAAGAACCTATCATTGACCGTATGCTTTTTGATCAACGGACTTACCTCCTTTGTTGAGGTCGGTGTGTCCGTCGGTCCCCATCAGTCTTTGCGGGCTTGGCGTCCTTCATCCCAAACCGCTTTACGGATCTTGCGTGTGCCGTTTGGGATGAAGGTGCCGTCCTGAGTTGCTTCACTTGGAACCCAAGGAAGTAGTTCAACTCGCCCATCATTGACATCTCGAATTCTGCGTCATCACCACTGCTAAACTCTTCACAAGACTTTGTTAGTAGAACCAAATATTATGTCATCGACATAAATTTAGCACACAAACAAATCACCATCACATGTCTTTGTAAAAGAGTTGGATCGGCTCTCCCAACCTTGAAAGCATTAGCAAGTAAAAGTCTCTAAGGCATTCATACCATGCTCTTGGGGCTTGCTTAAGTCCATAGGCGCCTTAGAGAGCTTACACACATGGTCGAGGTGCCGTTCATCCTCGAAGCAGGGGTTGCTCACGTACACCTCCTCCTTGATTGGCCGATTGAGGAAGCGCTCTTCACATCCATTTGGAACAACTGAAAAGAATGGTGAGCGGCATATGCTAGCAAAATCTGAATGGACTCTAGCCTAGCCAGGGAGCAGTCTCCTCAAGTCCAAACCTGCGACTTGGGCATAACCTTTGCCACAAGTCGGCCTTGTTCTCGTCACCACCAGTGCTCGTCTGTTTGTTCTTGGAACACCCACTTGGTTCCCACAACATTTTGCGGACGAGGCACTCAATCCAAACTTCATTGCGGCTTGAAGTTGTTTAACCTCTGCATGGCCAACACCTAGTCCGGATCTAGCGGCCTCTTCTACTGAAAGGCTCAATAGAAGAGACAAAAGTAATGCTCACAAAATTAACTAATCGAGATCAGTAGTTTCCCGCTAATGTCACCCGAATTTGGTCGACGGGATGATCCCTTTGAATCATCGCCGAACTTGGGTTGGAGGTGCAGTTGCGCTTCTTCCTCCATCACTTGATCATCTTGCGCTCCCCTTGATCAAGCGCCTCCACTTGAGTTGGGGTTTGCCTGCATTGTTGAGGAAGGTTGATCTCGTTCATCTTGTTCTGGGCGCACTTCTCCAATCGCCATGGTTCGTATATGTGGCCGTCAGACATCTTCTTCATCTACATCATCACAATCAACAACTTGCTCTCTTGGAGAGCCATTAGTCTCATCAAATACAACGTCGCTAGAGACTTCAACCAAACCCGATGATTTGTTGAAGACTCTATCGCCTTTATTTGAGTCATAACTAATAAAACCCTTCTACAGCTTTGGGAGCAAACTTAGAATTTCTACCCTTCTTCACTAGAATGTAGCATTTACTCCCAAATACACGTACGATACATTGGGTTTGTTACGGTTAGTAGCTCATACGCGTCTTCTTGAGGCGATGAAGGTAGACCACTGATGATGGCATGTGGCGGTGTTAGCGGCCCGGCAGCGCAAGGTCTTGAACTCCCTAGCATCGTCCTCGCATGTCGATGAGCGTCCTGTTCTTTCCTTTCTACCACACCATTTCTTTGTGGTGTGTAGGGGCGGAGAACTCGTGTGATCCTTCATCTTCAAGGAACTCCTCCACTTGAAGGTTCTTGAACTCGGACCCATTTGGCTCCTTATCTTCTTCACCTTGAGCTCAAACTCATTTTGAGCTCTCACGAAAAGCGCAGGGTCCCTTGGGTTTGAACTTATCCGCAAAAAGAACACCCAAGTGAAGCGGAAAAGTCATCAACAATAACTAAACCATACTTACTCCCTCTATGCTGAGATAGGCGATGGTCGAAAGAGATCCATATGCACGGCTCGGGGTCTTGAAGTGGTCATCACGTTCTGTGTGATGTGCTCCTCCCTTGTTTACCTTGACAAAAATTTGCCCACAAGGTCTATCTTTTTGAAATGCACGTTAGTCAATCTATCACGTGTTCTCCCTTTAGAAGCTTGTGAAGGTTCTTCATCCCCACATGTGCTAAGCACGATGCCACATGACCATGCAAGTCTTAGCTATTAAGCATGCATCTAGACCAAGCCTCTTCTTTTGCAAAATCAACTAAATAAAGTTTGCCGTCTAATACACCCTTAAAAGCTAGTGAACCATCACTTCTTCTAAAGACACATCTACATTTGTAAATAGACGATTATACCCATATGACATAATTGACTAACAGATAGCAAATTATATCCAAGACTCTACTAAAAATACATTAGAGATAGAATGCTCATTAGAAATCGCAATTTTACCTAACCCTTTTACCTTGCCTTGATTCCCATCACGAATATAATTGAATCTTGGAATCCTTATTTTTGGCGTAGGAGGTGAACATCTTCTTCTCCCCGTCATATGGTTTGTGCATCCGTATCGATAATCCGGCTTGAACCCTGGATGCATAAACTCAAAGGCAAATTTAGGCTTGGGTTTAGGTACCCAACTCATGTTGGGTCCTACAAGGTTAGCAAATATCCTTAGGGACCCAAATGCAAGTTTTGTCTCCTTGCATTTTGCCCTAACTTCCTAGCAACAATTTTCTTATCCTTTCTACAAATAGCAAAGGAAGCATTTAAAGCATAATAAATTGTGGAAGGTTCATTCACTACTTTCCTAGGAGCATGAATAACATTCTTTCTAGGCACATGATGAATAGCATTTCTTTGGAACAACATTTCTCTAGTAACATTTCTATCATACACATAAGAGGAACTAGGAGCAAACATGGCATGAGAATCATAAACATATGAATCAAAGCATCATGACTTACATTTCTAGTTTGTCTTCTATCATGATACAAAAGGCATGGTTCTTTTGCACATTACTAGCCATAGGGACCTTCCCTTTCTCCTTGGCGGAATGGGAGCCTTATGGCTTGTTAAGTTCTTAGCTTCTCTCTTGAAGCCAAGTCCATCCTTAATTGAGGGTGTCTACCAATTGTGTAGGCATCCCTTGCAAATTTAGCTTATCGAATCATTCTTGCTAGTCTTAAGTTGAGCATTAAGACTAGCGGTTCATCATTAAGCTTGGAAATTGAAACTAGGTGTTCACTACAAGCATCAACGTCAAAATCTTTACACCTAGTACAAATTTCAACATGTTCTACACAAAATTGGATTTATTGCTACTTCTAATTTAGCATTTAAATCATTGTTGACACCTTTCAAAGTAGAAATGGTTTCATGACAAGTAGATAGTTCAAAAGCATTTCATTTCTTTTAACTTCTAAAGCATAGGATTTTTGTGCCTCAACAAATTTATCATGCTCTTCATACAACAAATCCTCTTGCTTTTCTAAAGTATATTCTTTTCATTCAAGGCATCAATTAATTCATTAATTTTGTCTATCTTAGATCTATCTAAGCCCTTGAACAAACATGAATAATCTACTTCATCCTCATCACTAGATTCGTCCTCACTTGAAGAAGCATAGGTAGAGTTGCAGTATACCTTCTTCTCCCTTGCCATAAGGCATGTGTGGCCTTCGTTTGGGAAGAGGGTTGATTGTTGAAGCGGTGGCGGCGGTCCTTCATTGCCGAGTCGGACGAGGCAATCAAAGTCCCACTCCTTGCCTAATCGCCTCACCCTTTGCCTTCTTATAATGCTTCTTCTTTTCCCTCTTGTTTCCCTTTTCTGGTCACTTTCATTATCGGGACGGCTTAGCAATAAAATGACCAAGCTTACGCATTTGAAGCATGATCGCTTCCCTTGGTCTTAGTCTTGCTCGGTATCCATTCGACCTTTAGCAGCGTCTTGAAGCGCTTAATGATGAGCCATTTCTTCATCATTGCAAGTAGCCTCAATTTGCACCACCTTGGTAGCCTCCTTGCTCCCGATGCCTTGAGGCAATGGGTTGAGGCTCGTGGATTGGACGTTCAGCGCGTCATCGCGTACCTTGCCTCCTTGATCATCATCCGCCCACAAATTTTCCAAGAATTTCTTGAGGCGACATTTTGGTGTACAGGATTCTCACGAATATTATTCACCAAATGAGGATCAAAGCGATGAGACCTTAGCATGAGGCGAGACGGCGTGGTGGTCGATCCATCGCGTCTTCCATAGCTCCTTATCTTGTTGACAAGGGTCTTGAGCCGGTTGTATGTTTGAGTTGGCTCTCGCCCCTTATCATGTGAACCGTCCAAGCTCGCCCTCCAACTCCATTTTGGTGAGCAAGGTAGCATCATTTCCCTCATGAGAGATCTTGAGGGTATCCAGTTGTGGCGTTATCCAAGCCACTCACTTTATTATATTCATCCACGCACAATGAGGCTAGAAACAGTAGTAGCTTGTGCATTCTTATGGATTTGCTCATTAATGAATATAGGACTATCAGAACTATTAAAGTGCATTCCACTATCTACAATCTCCCATATGCTAGGATGGAGAGAGAATAGGTGACTCGCATTTTGTGGCTCCAAAATCCGTAGTCCTCCCCATCAAAGTGTGGGGCTTGCAGTGGAATAGAAAGCAAATGTGAATTTGAACTTTGCGAATACGAGTAGTCAAAAGAAAGTTAGAATTAACGGTTTCCTTTGTTTGTCGTGGTCGTCGTCCTTTTGGGAAGAGGACTCATCACTTGTCGTAGTGAGGCGATCTCCTTGATCGTCTTGTCTTCTTCTTCCCATCTCTTCGCTTGGCCCGAGCAGTCATTGGACTTGTCATCCCTTGGCTCGTTGACGAAGGACTCCTTCTCCTGTCGTTGATCACAATTCCCTTCCCTTAGGATCCATCTCTTGGGCGGTTAGTCCCTTTCTTGAAGAGAGCGGCTCGATACCAATTGAGAGCACCTAGAGGGTGAATAGGTGATCTGTAAAAACAAACTTAAGCCACAAAACTTGTTAAGGGTTAGTACAAGTATGGCCAAGTGGCTAGAGAGAACTCAAACACAATAACCACAAGAAAGCAATCACGCAGTTGACACGGTGGTTATCCGTGGTTCGACCAAGTACAAAACTTGCCTACTCCACGTTGTGGCGTCCCAGCGGACGAGTTGCACTCAACTCCTCAAGTGATCCAATGATCAACTTGAATACCACGGTGTTTTCTTTCCTTTGATCTTTTCCCGTTTGCGAGAATCTCCACAACTTGGTCTCTCTGCCCAATTGAGTTCACAAAATACGGAGTAAGGTGGGAATGAGCAACGCACACAAGACTCGAAAATCGAGCAACAACACGCACACAAGTCGCAATGAGCTCTAGCGCAACACAAAGAGTTCACAACTCCACAAGAGCTCTATATGCTATCACAGTGAAACAATCGTGAGATTGATGTCTTGGTGCTAGAAGAGTTGTAGGAATGCTTGGTGTACTCCTCCATGCGCCTGGGGTCCTTTTATAGCCCCAAGGCAGCTAGAGCCGTTGAGGAACAAATCTGGGGCCATCTTTGCCTTACATCCGTGGCGCACGGACGATCGGTGCACCGGTTTCTTGTCGGTGCCGATTTATTTCCTTAAATGGCGGCCGACGATTGCCGGTGCGATTCAGCGCCGGGCGATCGTGCACGGGCGATCGGTGCTCTTCCTCCGACCGTTGGCCGGCCACGTGTCGCGCGCCGATCAGCCGGCCGTTGCTTGCGGCCGGCCGTTGGCTCACGGACGATCGGTGCACCGAACGATCCGGTGAATTTATAGCTCGCCGGAGAAAAAACCGAGCGGCCTCTTGGCAAAGCGATCAGCGCCGGACATTGTCCGGTACCACCGTTGTCGGCCACCGCAGACGATCGGTGCCCCGCATCAAGCGACCCTTGGCAGTACTACAAAACCTTCTCTTCTCTTCTTTATCTGCTTTCACACACTTAGACAAATATATTAGTACACAAAACCAATGTACTAAGGCTTAGAAACATACCTTAACTTGTGATTTGCACTTTGTTCATCCTTGGGCATATTTTCACATTTAAGCACTTGTGTTTGCACTCAATCACCAAAATACTTAGAAATGGCCCAAAGGCACATTTCCCTTCAGTCGAACTCCATCATCAAACAACGGCACCGGGTCACCGCAAAAGTTCTGCGGCCTGGATCACCATCCGGTGGATGGCGCCGTGTCACCATAATGAATGGGCAGTAGAGCGTCTGCATGGCACATTTACAAGGACCAAGCCCGGATCTACAACGACTTCACAGTTCGCCAGATGGATGAAGGAACTCCTGCAGTGGTCTAGAGTCGAGGACAACGGCGAGCCCACGGGGCTTCATAGCTCTTTCTAGTCTATGGGGCGAGGCCATCTTGCCCACTTAGAATGTCGGCTCCCCAGGGCCAGGGGGCCTCGCCACCAAAGCAACCAAGTTATCGAGAAGACTACGTGAGACGGTGGAAGAGGCTCGGACATGGCCTTACTACATTCCGCAGACGGCCCAGTCCGCGCGGCGCTACCACACCCAGGGAGTGCGGTCAGAGACCTCCACGTGGGCGACACGGTGCTTCGCCATGCAGGGCGCCAGCGCGGCTGGCTCACGCTCCTCGGAAGGCCGTCGTCATCGCCAAAGTTCGAGCCGGCATTACAAGCATGCCTTCATCAAAGCGAGGTCCGAACGCCTTGGAACATCCAACAGCTCATCCGCCGCCCCCTTAAGATGCTTTCAAGTTGTTCATATACCTCGCTCCCGCAAAGTTTAGTCATCAAGGAAGGGTCATTGCCTCTGTAAGCCCACTCCCTCGGGGCTAAAGGGGGCCCCTCCAGTCAAATTTTCCTCAAAAAGATCCTTTCTGCAGGAATGTCTTTGTGCTTTTCGACTACGCCGAAAGTGGATCCACAACGACGGAGTTGCGTGGCGGCCAAGGCGGCGGCGGGGACTCCTCCGGCCCGGGATACGGATACCTCACTCATCACCTTCGTGATAAGTAACTCGGCGTTGGATAAGCGATTCGCGGATCCGAACAAGTCTTCACACTCGAGCTCCTCTCGGCGATTCTTGGCCCTTCTCGCTGCGTCGGTGGCGAACCCATGGGCGGGCAAGAGTGCATGGCGGCAAAGCCGGCCGTGAGGATTCCTCGCCTGGGATACGGATACCTCACTAAGTCACCTTAGTGAAAAGCAACTCCCGCCGCGCGGACAATTCCGTTCTAAGCGAAAAGTCGGATACTCAAAACAAGAGGAAAAGAAGCGCACTTTACAACACTGCGATGGTGTGTTTAGCCTCAGCGGCGCGAAACGCACACTACAAGATAATCCGATCCTGCAGGCTTGGATCTTGCCAGCTGGTTGGGAGCGGCGGCACCTCTGGCGTCGACTACACCTTCGGCGAGTCCGACCTAGCCTCTGGCGGCGGCGGTCCGAGGATCTCCACTGAAGGACGACATCATCATCACGCTCGCCATCGCCGCCGGGTCTTCTCAAGAATCCGGCTCGGTAGGCGGCTCGGCCGTCACCCGAGGCCTCGGCCAAATCCCCAGAAGACATCACCGGCCGAGCCTCGCAGATCAACTCTGGCGTCGGCCCCGCTAATGGGCGGCCGGCGGAGCTCGGCCACCAAGTCTTCTTTCGAGCCAACTCGCCTGCTCGCATCGCGACACCGCTACCCCTGCCGGCTCATCGAAGAGCGCGGTAGGGTTCCTTTAACTAAGCAAGAAGCTCGACAAAGAAGGCCGATAGGCGGGGACTCCTTCTCCGGGATACGGATACTCACTCGTCACCTTGTATGCGGCGACTCACTCTTAGTGGTTCGCACAACCAACAGGCGTAGTCTTAGTGCTCAAAAAAGGAAAAACAAACTCTGCGCAAAAAATACATACGCGTTCCCGACAACCACAATGAACAAAAGGCCGACATTCAAGGTGCCATTACAAACGGAACTCCGTTCTCACACCTCCGCAGTCGAACAACCCCTACGATTGGGGGCTGGCGAACAGCAGAGAAAGACCAACGAGCGGCTCGCCGCCGCCCGCTCCAACGGCGCGGCGGCGATGACGACTCGCCTTTCAGGGCCTAACGGCGGCGTGATGACCTCGGGGTGGATGCTGCCGCCATGAGGCCTCGCCCATGCCCCTTCGTGAGGCAGGGAGGCGGCCGTAGAGTTGAGGTCGATCCGTCGTGGCCGGCCTATCTTGTCGGTGAAAGAACCTCTTCCACCGCCATGGCGGAAGGCGGCGCGGGGCGGCTCGGCCACTCCGAAGAGAGCGCACGCGGCAAGCCGCCAGCAGTGAGCGGGACGCCCTTCCCTCGATCAGGGGAAGGAGCCGGCCGCCGCGCACTGAGCGACCCAACTCGACACACTCCCCCGGCCGACCGATGAAAATCCTTGAGGGAGGCGAGGTATGACCCGGTTGCTTTCCCGCCATCCAGAGGTCACCATCTTGGTGACCGCCGTGTCGTGGGCAGCGTGATGAAAACTTGAAGCCGAACGATGCGGTACCAACTCACGGAGTTGCGTTCCTCAACGACAAGGCGAGGAGGGTGCGGTGTCCCCATCCGGGCTCTAAGGTGGAAGACACGATACATAAGGGAGCTGCGAAGACATGGTCGCCTTTCAGGGGTCACCCACCTTTAAAGGCGACTCTCCTACCGTCCCGGTAGTGTTGGTGCGAGTCTTCTCCAACACGCTCCGGTCCTCCCCCTCGCGACGCGGGGGGACCCGCCGCCGATACAAGCTAACCTGGCGGAAGAAGCCAAACACGCGCGCCCGTGCGCCAATGTTCTGAGCATTATTCCTCCACTTTCGTGATTCCGGTAGGCGAAGGCGAAACATGTCGGCGGCATAGCCGCGCCAAGTGGCGCGCCCTCCGACTTCGCACCCGGCGCCATGGCGCCTTAGCGTCACGCGCCAGCCGCGTCGCTACGGCAAGCAAGCTGCATCGCCACTCGCCTGCTTGCCACGCCTCCTCATGCGAAACATTCCGCGACTGAGGCGACCTGCGTCGACCAGCCGTGCAGCGGCCCGCGATGGTCAATCGGCCAAAAAATGGGCGCGGTAATGGCGGTGGCGAGCGGCGGGAGGCCGTGGTCGTCGTGGTAGCTTACGCCCCATCTGGGCGTTGAGAGAACCTCTCTCACGGCGTGAAGGACGGCGCCCGTGTTCCGTTCGACGGCCACGGCGCGACCAGCAGAGCGGCCGCCATGACGCTTCCGTAGCATTACTCCACGGCGGGAAAGCGCCTCCGGCGGAAGCGGCGGCAGCGCCGCCTTCGCCACAATGACCGCTGGTCAAAGAGGTGCGCCGCCATTCGATTTTGCATCCTTTTCCTTTTCCTCTTTCTCTCTCTTATACGGGGACCGGGAGAGGATACCCCAAGGATCCTTCTCGTGAAGGAAACAGGCTCGAGCCTCCCTTGATAAGAGGTTCAAGGTCGCCCTCGGAGGGGTTCAAGCAGCCGCCTCGAGCTGCGTGGGCTCCACACCCACCTCTTGGTTAGAGGTTCAAGGTCGGCCTCGGAAGGGTTCAACAGCGCCTCAGCCACGGGCTGGCGCCGCGCTGCGTCCATCGGGGTTCAAGGTCGGCCCTCGAAGGTTCCACGGCCGCCTCGGACACGAGGCGAGGGATGACCATGGTCACGGTCGATACATACCAAGGCTCGGGCTCGCTCGAGGCCTCGGACATTTAGAGACCAGCGAGAAATGATCTTGCTAATCCCATCGAGGGAGGCACCAGCTCTCGGACCCGTCGACAGGACCGGTCCGCGATCACCGTAGGTACTTTTGGTAGCCCGGACCTCTAGCGACCTAACAAATGGGGCACGGCGTCCACTCGGATTACCCGCCGGCTCCACGGAGACACCATGTTTGGCGCCCTCAGAGGGCAACATGGCGCTTTCCCCCTCCTTGCGGAAAAGCGTGCAGCGTGTATTAAAGAAGTCAGCAGCCCTTGGCCAGTCCCTCGCCCTGTCTGCGAGGCTCGGGGCTGCTCTCGCAAACCGGCTCCGGCCACCGTTGACAACGCCAACATTGACAAGAACTTGGGACCCGGCCAGTGCACGGACTGCGGCCACTTCGCATGAGAGCGACGGACCGTAGGCATTGCGTGAGGCATTAAGACCTCGAGAGTCAAACCACTCCTCGGCCGAGGAGCTACCGTGAGTCGGCTCGCGCGCACCACCGAACAAGCCAAGCAGAAAGTGGTCCCTTGCAAGGTCGCGACAAAAGCCTCCAAGCGAGTGTTAACTCCCTTCGAGGCTCGGGGGCTCTTGTCCGGTACCATAATTAGGGTACCCTCAAGACTCCTAATTCTCAGTGGTAACCCCCATCGGCCAAGCCGCAAAGGCCGATGGTGCGACTAAGTCGAGGATCGGTCCATTCGAGGGACTCGATCACGCCTCGCCGACCTAGCCTCGGGCAGGCCGGCCGTACTGGAGGATCTCCGTCCGCAGGCCCCCCTCGGTGCGACGCATGCCGGCTCGCCGAGGCCCGATCTTCGCCAGAAGCAGTACAGCCAAATCGCGCGTAACAGGCCAAATCGCGAGGCATTTAATGCGGTGGCTCGACACCTTATCCATCACGACGCCGCGCCTCCAGCAGCCGGCGAAGTGACGCATTCGCCGCCCGCTCAACAGCCGGCTGACAGGAAGGAAGCGCCGCTGCGCCGCTCGGCTGGCGTGCCACTCGACGAGTGAGGCGGACAGTGATGCAGGGCTGGCTCCGAGCACCATAGGAAACTCCGCCGCCCGACCTAGGGCTCGGACTGCAGGCACCGGCCGGAAGATGGCGAACTCTGCTCGCGACCTAGGGCTCGGACTTCGAGCTCAACCCCGGAGACGGCGAACTCCGCTCCGCCCGACCTGGGTTCCGGACTCGGCTCGGTGACGGCGACTCCGCTCCCATAACCTTGAGCCTGACTCGGGCTCAGGCCCGGAAGATGGCGAACTCCGCTCCCTGCCAGGCCAGGGCTGGGACTGGCTCGGCCCAGAGCCAGTGAACTCCGCCGTGAGCCGTGGCTCGGACTCGTGACACCCGATTCATCACTCAGGAGTTTCTCAAATGCCAAACCAAGAACCATCATTTTATGTGAACCAAAGTAAGCATGAGCATCAAAACAACTTAAGTAATAAAGAATTCACCAAGTAAATACTTAAAAGTAACATGATCAAAACAATTGAGTCTTTGAAAAGATAAAATGTGCAACCTAATTAAGAACCCTAAGTGAGCCCCATGAGCAAAATTCAAGAAAATAAGAAAAGGGAATGAAAGTTTAAAATTATGACTTGAGCCAATTATGAAAATTAAAGAATATTTAATGGGCAACAAGAAATGTTGAGAAAGCTTAGCCAAAATAACTCAAATAAAACCCCAAATCAAGCTCCTCATGTGGGACTCATTAGGATTGAATTTCAAGATTCTGAAATTCGACACTTGAACCAAAGATCGGGATGTTCACCTTGATCCCTAACTTGAATCCTAATGGCCCCATTGACAAAATTGTGTCTAACTAACCCCTCGTCGTGTGCCGGAAGATGGCATTGGGGCTGAGCCCTAGACACGACAAAACCTTGGATTTGCCTCGGGTTTGGGCGGGGAGACAGACCAGATTTCCTGCTCCATATCGCAACGATGGCAAAACCTATGACCCCACACAAGAATGGTAGCTTGTAGGGAGGAGAAGAGGTTTTGTGCACTGACCAAGGCGAGAGCGGGCTCGGATGGCGACCACAGCGCCCAGGCAGGCGAGCCTTGCGAGGCACACGTGTTCGACCACGTCGGCCGCGCCGAGCTTCGCCAACCCGCGCGCCACCCGGCGTCGGTCAAGTCCGCGCAGCGCCACGCCCTCGGCCCGTGCCGCCCGCGCCTTCAAAGCCTCCCGGGCGCACCTCTCGCCGCCCGCACTCACCCTCCCGGCCACCACCTTTAAGCTCGGCGAGCTTAATTCCGCTGCCATTGCGCGAGCCCGGCCACCATGGCACCCCTCCTGGCCACCCTCAAGCCCACCCGATTGCCAGCTCCGCTAGTAGCTGTGAAGCTTGCTAAGCCTCGGACCGGCCGGACTTCACCGGGGCAGGATCGACCTCCAGGACTTGGTCTTCCGCCACGCAGGTGGACCGAGCTATCGGTGAGTCTCCGCCCAATTCCTTTCGCTCATATCTTCTCATCCGTGAACCTCCTCGCCCATCTAATTGAACTATCTCGCCGTGACAAGGCCGGTCTCCTCGCCGTAAGCGAGCATCCCGCCGCGCGTGGACCGACCGACTCCGGCCATCTCCGTGGTGTTCGCACACGTTGTGATCCCAGACCTCCCCTTCGTCCTCGACCACTTCCGGAGCAACCTCGCGCAGAAGCCCTCTCGCCCTTTTCTCGCGCTGACTCTCGTTTAAGGTAGAAGAAGACCTGGGTTAGGATTGGTAGAACCGAGGTTTTTAAGAATGTCGATGACTCATGTGAATAGTAACCTAGGGACGATTAAGGGAAACTTAGAAAATCGCGAGGACCTCGTGCAAAGTGGATTTCCATTTAATCAATTCTTATTCTTTTAAAATGACCAAGAACTTAGAAAACCCATAACATGATGAAATCTTAATAAAAGTTGTCAAACCAATTTTGCTAGCTCCCGAATATTATGATCTATCATTTAAAAATAGTAAACTCCATGCTTTCTGTTCAAAATTTTAAAGTTTAAAATTAAAGAATGAAACTAAACCTTGTTTAATTAAGGAAAATTAGTTTTTCTTAATGCTGAACTTAAGAAAAATTGTAGATGTTTAAACCTTATTTAGACACTATTTAAAAATAGTAGGAACGACATTGAAAAATATGATGTAGTTTTTCATTTAAAGCTAATTTGTCCAAAACTTAGAAAAATCGAGAAAGGCCTTAGAAATTAATGAACAGTGATTAATAATATTTTTCCTAGTTTACTTATGCAACAGAGAACCTAGGAAAATGAGAGACCATTAATTTGGACCGTTTTAAATTAAGATGATTTGTTTAGCCTCATATAGACTGAAAATCAATTATTAGAGTTACAAAACTATAACCAAAATGATTAATAAAAATCTAGTGGACTTATAACCACCAGAGCCTCACTACAAAAATACAGAGCACCTCAACCCAACTTTTTAAGTAAAGAAAATAAATACAAGGTGATAATAAGGCATTTTCCAAATAAATCATGAACAACCTTTTAATGTGATAATAGGCAACCAAAATTTGCTAAGCTCATGAGAGGTAAACTACAGAGAAAATGCAAACCCATGAAGAAGAAGTAAACCCATACCTTTTGCTAATAATTAAGAGAAAGACCACTTAATTCAAATAATGCAACCACCCCTTCCCTTAAGCAAAAGAAGCCAAACTAGAATGATTGCTCTTGCACAAAATACTAACTATGAAAAATAAGAACTGTTGTTTGATATTTTTCAAATATAGTGGTAGTAGAAAGCACCCATTTGGCTAGAAACTTGAGAAACCATAGAAAATTTATTAAGAGGTATTAATGATTAGAAATTTGTATCAAGTCATGTTATAACACCTAAAAGCCAGCAAAAATAAGTTTTAGAGAATTACCCATGTTAAATAGTAGTTGTAGTTCAGCACATACCTTTGAGTTTAATAATTTTGTCCAGAGAAAACTATTCACTTTCTGACCCCCAAATTTTGAGACAGGAAACATACACCAGTAACAAGCCACTGTAATTTTGTGGAATTTTTAGAATTTTATAAAGCAACTTGTAGTTCAAACCTACTCCAAAACATTAAAGAGAATAAAGAAAAGAGAAGAAGGAATAAACCTCATCCCAATAAGTCTAATTTGAGAACTTATCATTTATCCCTAAGACTTAAAAGGAATTCAGTGGAACCCCAAAAATAAACCTACCACTTACCTTAGCTAAGTCTAACCCAATTTACCAAGTATACCACTAAGGGTTTTACATAAGTAAAGTTAACTTGTTAAACTCAAAAGATCCTACACCAAAAGTTGTAATTTCTAAAAGCACATATCATATCATGCATATATCTTACGATTGCATTCATTAGATTGTAATCTTGCCGACGAGATGCTCATCCCCAGAGCAAGGACTCGTCCCAAGAGGAGGACCAGAAAGCAAGGCGAGGTCGCCTTGAGGATCTCCCCACCACACAATTGAAGGCAAGCCCGGTTTATGCATAACCATGTTATTATATGCTACTTTACTACACTTAATGCTTGTAGGATTGCAATGCACTTAAGTGTAGGAGTTGCTTGAAACCTCTAGTTGCATGAACTTAGGATTCCTTTTGAGATGAATACTAGTATGCTAGGTGAGTAGTCGCTTGCTAATCGGATCTCGGTAGAAGTCGAGTGATTTTCTAGCACTCGTGAGGTCGAGAATTGATTGTATCATCTTGAATATGGGATCTATGTTGGTCTATGGTTTGGATCCGGGAGGATGCCTTGTCCATGAGGCGGAAAAATGAATTAAGGATTAATGTGTGGATACAGAGTCAAGCTTTGAACGTATTAGCACATGCTTGGAAAATGGTAACCCAGAAACCTAGTACTGAGTGAAGGAAGGACGACTATCCTCATGCGACCCAGACTGGTCTCCCATGCTAGCTATGGTGGGTACAAGTGCGGTCTTTGCAGCGGCCGGGTTAGTGGAGCATTGTATGCCAAGGCGATGAGGCTGGGCCTTGAGCGGGAATCGATGGGGCGGTTGTCATATGCGGGGTCAGTACCACGACATGCGTGTGTTTAGGTTTACCTTGCAAGGTTTAAAACTCGATTCAGATAAATGCGCTTCTCGCGACTAATGAGACCGCTGATTCCTTATCTTGCATCGAGTAAGAAGTGAAATGTGGACTACATGAGATAACTTGTTGATTGAACTAATTGATTGTTACCATGTATGCTTAGAAGGAGCAAATCTAGCTAAGTTAATGATGGTAGAAATTGAAAAGCTAAAAATTGATTTTAGAAACAGCTAGTGCTTTGGCAACCAAACCCTCGATAACAAACATAGTCTAGAGGTAGAGGAGTAGACTCTCACACCGGTTAAGTCTAGCCGAGTATTAGTATACCTTTGTTGCAGTGGCACAATTTTACGGTACCATGAATTGGTTGATGGTGTGACTTGGCCTGCCATGCGCTGGGTTGGGCCCGTCGAGTGGGATGTTGCTCGTGAGGGAGCATGAGAGAGTAGTGGGCTGAGCCTTGCCCATTTCCTCATTACCGGCGACATCGATTATCCGTACTTTATTTTGTGAACTTTATTTGCTACTCAAAAACTCGATTTATGTAATAACTCCGGTTAATTTGAGGTTTCTGTTTTTGTATTTCTTCGTGACTCACCTTCGAGTGGGATTGTGGAATTTGATCCTTAAGTGGCTCTATCAGACTAGATATGAGGGATCGACGGGGTTATTCGATTTAAGTGTTGACCACACGGGCGTGACTTAGACATAAATCTGAATAATTGGGTGGTTACACCTGGTATATCGGAGCAAATTCCACCACAGAGAAGGATAATAAACCATGAATACCAATTTTCAAAATCTAAAACTTGCCTAGAAATCACACGGATCGACAGGACTAGACCGCTAGACCTGGGACGAAGGCCTTAGGCATAGAGGAAATAGGTGGCTAATTAATTAGGCCCATAGGCAATATATATTTTAAGGATGCCTAAAAAGGCACCCTATCTTTCTTTGAGAGAAACGCTTTCTTCCAGCATGCATGCATTATAAAACCCCAAAGAGGAATTAAAATTTGAGCTAACCTTTCTTTAAAACCATCCGGTTCTCTTTTTCTTTTCCTTCCACCATAATCTTTATTTTGATTCCCTTCCGCAGATGAATTCACACCACCCGCGGAGGAGACTCGTTTGTTTCGACTTCCTTTCTCGATGGCTTTCCTTCCATCTTGTGGGAAGTGCTTAACTCACCGTTACCCTACGCCCCTTTGTACTGCATGCGGTTGTATGAGGAGCACGGGTACCTTGTTGTCGTGCAGGTGGACTTTGGAGGCTCATCCCACCGGTGGGTTGGCGTTCTCTTGACTCCGAGGCGATTGGACTCGGGACGGACGACACCGTTGAGCGTGCCATGAAGACTCGACAACTTTCGTGGCTACCATCCTCGGAGATGGTGATGCACCTGGGACTCTTCCTGCGAGAAGGATGATCCCATGTGGTGTAACCGCGTGAGCCATGTGAAGGATGTGTGGGCAATGTATCTGACTTGGTTGGGAGGGTCCTTGTTCGATGCATGAGTCGCATTGTACCGCCTTCAGCCCTTGAGCGATGCTATGACACTTCTTGCTAACAGCCTTTGCCAAGCTCACTCTCGACGATCAGGAAGATTTTGTGGTCCACTATATCACAGAGTTGGTGGAGAAGGATCTGCAGTGGAAAGGTGAGCCTGTAGATACCACTGGAGCGACAAGTGGAGATCCGAGACAACACAATTGATGTCTTGAGAACCAGCTTCACGATGTGCGAGAGGAACTCGAGGTAATGGCCACTTGGACATGCACCACGGAGATGGAGGCCAATGAAGTAGGAAGCGAGGAGAAGAGGCTCGAGGCTAGGACCCCTACAGTGCCAATGGGACTACCTCCGCGATGCCTCCTTCACGTATCGATCGTCGCTTCTACCGCTCTGAGTTAGCGGTCGCTTTGACATTTTAGGCGGATAGAAACCTATACGAGCTTAGTAGTATCACATTTTGGACTAGGCTTGGGTACTTTCCCTGATTGATGTAACCTATAAACTTTTGAAATCTGGGATCTTTGTCACCATGTTATCTTCATTTCGAACCTAATATTATGATTATGGCATTTTTCCTTCCATATGAGATGATATCTTGTCGTTCAAAGCGTGAGTTGGGATAACAATGGCAACAATCCTCTGTTTGTATCAGCGGCAAGCGTCGCTTGAAGCGAAACGAGCAAGCTCCCGCCACCTCCCAGCTCCCGGTCGCGGTCGATGGCAGCGAATGAGATTCTGCGCAGCTCTTCTGGCCAGACCACCATGACATCATGGTGGAGGCCATCAGCGACCTCCGGCTATGGCAACATACCAGAGTTTCGAGCCGCAACCGCCCTTGTTCACCAAGGCGAGAGGATCCGTTGGGCCGACGTGTGGCTCGCGTCATCGAGTCCAGTTTCCCCTCCTCTGGAGACTGATGAGACCAAGGCTCGCCGCGCCACAGCCTTGGCGGCCCTGCCGGACTTGGTGGGACCACTTCATGCTATGCTCCCTAGTCGTGAGGTATCTTGGGAGGAATTCAAGGTATCTTGAGGACACCACATCCCGGTGGCATTCTTGATCGGAAGTTGAATGAATTTCGGCCCTTAATCAAGGAACCGCCGCGACGCAATGGTATGCACAAGCCTTCAATGACTTATGCGATCGGGTATCATGCGGTCTGATGAAAGAAGAGGATCGCCCCAGCAGTCTCAATACCAAGCTGGGAACGACTCAACACTTATCGGTGATAGCTTCAATGAGTTGGTCAACATGGCCATCTCTCCCAGAAGATTGCATTGTTGCTCACCGGCGAGAGAAGAAGAAAAAGGCACCAATGGCGCACCATCCGCTCGGGCTCGTGAGGTTCCGTTGTTTCTCACAATAAGAGCAGGTTTTTCAGCGTGAAAGGGCAGATAAGTGATCAGGCCTCAACGCGGCGGCAAGCACCAGCTATCCGCTCTACGCCTAAGAAACAATCGGCCTCGCGGCCAGAAGGCAGTTCCCCGAGCAATGGGAACAAGTGTTTCACAGTGGCGTGACCACTATGCCAAGAATCGAGAACCGCAGAGGCGGTGCGGCACCAAATCAAAACAAGGGAAGAAAGGTACAAGTCGAGGCAAGGAAGCTCAACTTCACCGCTCTAGAGGAAGTGCCGAAGGAGCTCCTATCATGACCGGTATCTTTTGATTTATAATCAACTTGCTAATTACGTTTGATTGGTGCATCTCATAGTTTCATTAGCCAAAAGTTCGATGCAAACGCAAAGCCGCCATTCTCTCACTCAAGGGTCATTCATGATAGTCACACACAGGGTAAAATTGCAACTAATCAATTAAACCAAAGTGTGCCTATTCAAGTAGGAAGCCACATTATCAAAACCACTCTTCTTGTGTTGGGATTGGAAAATGTGGACATTATTCTAGGGCAAATTGGATGACCTACCAAGTTGTGCTTGATGTAGCCATCGTGCCGTGGAAGTTGATTCTCCTTTGCGAATTTCACTTTGATTCTGCCTAGTCGGGTTCTTCTCGATCATGTGCTTTCTCTGAGCGGAGTACCCGAAAGAAGATCGATGATGCAGTGAGTATCGATGTCTTTCGATGAATTGCAGAATGCCACCGGGTCGGATATTGAATTCGCCATCGAGTTGCAACGAGGAACCCCAATTTCAAGAGGCTCTACGAATGCCACCGTGAGTTGGCGAGTTGAAGAAGCAATTGCAAGATTCTTTGGATAAGGTTTTATTCGCCCAAGCACTTCGCCTTGGGATGTCCAGCATCTGTTTGTGAAGAAGAAGGATGAAAGCTTGAGGTGTGTATAGATTACCGCCTCTTAATGCGGTAACTATCAAGAACAAGTATCCTTTGCCTCGTATTGATGTTCTCTTTGACGGTTGGTTCGGGCCAAGGTGTTTTCCAAGATAGACCGCTCGGCTACCATCGGATCAAAATACGAGCAAGTGATATTCAAGACGGCATTCTCAACGGATATGGGCTATATGAATTCTTGGTGATGTCATTGGTACGACAAATGCACCAAAGATATTTCATGTATCGATGAATTCCTTTTCATGAATTGGACAAGTTTGTGGTGGTTTCATCGATGACATTCGGTGTACTCAAAGAACGAAGAAGAACATGTCGGCATTTGCATGTAGTGCTTCAACGTCTGTGACACCACCTTTATGCCAAGTTATCCAAGTGTGATTTTTGGTCAAGGAAATCAAATTCTTGGGTCACACTATCTCTCGGGATGGAATAGCGTTGATCGATAAAGTGCAAGAGGTGATGAATGAGGCCACCAACAACATTCGCGGATTCGGAGTTTTCGGGATTAGTAGTTATTAGAAGATTTATTCGGACTTCTCTCGACCGCGAAGCCTATCAACGATTCTTCGAAGAAAGAGGTCAAATTTGTGTGGAGTCGGCGCGAAGATGCCTTCCATGCATTAAGGCGAAGCTCGACCCGCACCAGGTGTTAGCGCAACAAGCAGCGACAAGCCTTTGATGTATATTGTGATGCCTCGGCACGGACTAGGTGTGTCTTGATGCAAGACAACCGAGTCATTGCTTATGCCTCAAGAGCACTCGGGTCTCATGAGCAAAATTATCCCACTCATGACCTTGAGTTAGCAGTGATGGTTCATGCATTGAAGATGTGGGGCACTATCTAATGGGACCTTTGCAACATCTTCACGATCATAAGAGCCTTAAGTACATTTTTACTTAGGCTAATCTCAACATGAGGCAGAGAAGATGGCTAGAGTGATCAAGGATTATGACTGAGGTACATTATCACCCGAGGAAAGCTAATGTGGTAGCAGATGCCTTGAGTCGGAAGTTGCAATGCAATTGTATTACGATGGATTCGCATTAACACCTTGTGTGATGAGTTGAGCAAGATGCAAATTGAAGTGATTCCTCGGATCTTTGTCTCAAATTTGTTGAGCTAGCTTTGCAACCAGTTATCATGGCTCATTCAGTGACAAGGGAGTCAGGATTATCAGAAGAATCTCCATCGAAGGTTGAGAAGTATAATTGTTTCCGCCAGGATGAGAAAGGTGTGTTATGGTTCAAGCGTGGTGATTCCTAGGACCGGGAGCTCAAGAGGAAAATTTTGGATGAGGCTCATCTCTCCAAATTCTCTATGCATCCAGGAAGCACCAAGATGTACCATGATTTGAAGCTTTGTCTTTGTGGACCGAATGAAAGGGGAGATAGCCCAGTATGTGTAGAGTGTGACACTGTCGGAGGATAAAGGCAAGCTACTTGAAGTCCGCTGGAGCTTTGCAACCAGTCCGTACCTTCGTGGGAAGGGCGACATCAGCATGGATTTCATTGTGGAGTGCCTTGCCACCTCTCCGTCATCATGATTCAATTTGGGTTATTGTGGACCGATTGAGAAGTGGCACATTTTCTTCTGTGCACACCACCGATAAGGCTCGAAATATGCATAATTGTATATTGGCCGGATCGTGTGTTTGCATGGATTACCTGGACCATTGTTTCGGCCAGAGCCCAATTTGTTGCCAGATTTTGGGAACAATTGCAAGAGTCTTTGGGAACCAAGCTAATCAGAGTTCAGCTTACCACCTGTTGATGAGTCGATTTCGGAAAGGTAAACCAGATTCACGGAGGATATCTTTGAGAGCTTGTGATCGATTGTGTGGCAAACTGGAATAAGCCTCTCCTTGAGCGAGTTTGCTTATAACAATAGTTATCAATCCGACCTAAAGATGGCACCTTTTGGCTCTCTATGGAAGAAGGTGCGGACACCTCAATTGGTCTCAACTCGGTGAAAGAGTTTTTGGACCCAGTTTGGTGACTAAAGCCGAAAGGAAGGTCAAGCTAATCGGGAAGAATCTAGAAGCCGCTCGGGCCGGAAAAAGAGCTATCATGATAGAAGAAGGAAACCTCTCCAGTTCGAGGTGGGAAGTTTCAGATACCTCAAGGTATCACCCACCAAGGAGTGCGAGGTTTGGGATCAAAGGCAAGCTAGCCCTCGTTACATTGGACCTTATGAGATCATAGACATGTGGACCCGTGGCATACAAGAGTGGGTTGCCTTCAAAGATATGCGCCATTCACGAGTGTATTCCATGTATCTCACCAAGAAGTGTGTTCGATTGTAAGATCATAGTGAGCTGACTGGAGATAGAGCGGATCTATCGTACAAGTGTACCTCCAAGATTTTGGATTGCGGGAAAGATCAACTCGGGCTAATCGATCAAGATGTTCAAGGTCCATGGAGTAATCACTCGAGAGGAAGTTACTTGGAAACAGAATTCCCTGAGATCCAACTTCCCGATTGCCTACCTAAGGAAGGCAGTGCGTAATCACCCCCCCTCCTCCGCCTTTCAATCTAATTTTCAAAATATGATCTCAATTGAATGAAGAAACTATAAAGTAAAACTTAAAAGGACTTCCTTCAAGTTGCAAGAAATGACATTGACGATTGCCCTAATAATAACAATCATCACCCTTTTCCTATCAGTCTCACCCTTCGCTTCACCACGGAGGACTCCGCCCGAATCTCGGACGAGATTCCTTAAGGGGAAAGGCTACACACCCATGTCACTCAGGTTTTTCTCAAATGCCAAACCAAGAACCATCATTTTATGTGAACCAAAGTAAGCATGAGCATCAAACAACTTAAGTAATAAAAATTCACCAAGTAAATACTTAAAAGTAACATGATCAAAACAATTGAGTCTTTGAAAAGATAAAAATGTACAACCCTAATTAAGAACCCTAAGTGAGCCCCATGAGCAAAATTCAAGAAAATAAGAAAAAGGGAATGAAAAGTTTAAAATTATGACTTGAGCCAATTATGAAAATTAAAGAATATTTAATGGGCAACAAGAAATGTTGAGAAAGCTTAGCCAAAATAACTCAAATAAAACCCCAAATCAAGCTTCTCATGTGGGGACTATTGGGATTTGAATTTCAGGATTAAAATTCAGACCTTGAGCCAAAGATCAGGGTGTTCACCTGATCCCTAACTCGAATCCTAATGGCCCCATTGACAAAATTGTGTCTAACTAACCCCTCATGCAGTGCGGAAGATGGCATTGGGACCTTGAGCCTAGACGACAAAACCTTGGATTTGCCTCGGGTTTGGGGCAGGGAGGCGGACCAGATTTCCAGCTCCATATCTCTCGCAACAGTGGCAAAATCCTATGACCCCACACAAGAATGGTAGCTTGTAGGGAGGAGAGGTTTGTGCAGCCAAGGCGGCGAGGCTCGGATGAGCGACCACCGCAGCGCGAGAACTTGGGCGAACGCCGCGGGCTGCGTGTTTGACCACGAGTCGACCGCGCGAGCTCGCCCAACCGCGGCGCGCTCACCACTCGACATCCGGTCTGTCAGCGGCCACGCCCTCGGCCGTGCCCTTGCGCCACTTATAGCCTCCGGGCGCACCTCTTCGCCCGCCTCACCCTCTCCTGGCCACCACACCTTTCCTTGCTCGTGAGCCAATTCCGCCCGCCATTGCCGCTAGAGCCGGCCACCGTGGCCAACCCACTCCACCACCTCAAGCCGCCATGCTTCGGCTAGCTCGCCAAGATGGCCGTGAAGCTTGAGCCACCGGACGGCGGACTTCACGGAGGCGAGATCGCCTCACCGGACGGTCTTCGCCGCTTGGCGTGGACCGAGCTATCCGTGAGTCCGCCCAGATTCCTCCGCTCCTTATCTTCTCATCCGTGAACCTCCACCCATCTAATTGAACTATCGCGTGACAAGGCTGCGGTCTCTCTCGCCGAAGAGCATCCCGCTGCTGCGCGTGGACCCGACTCGCCATCTCCGACGGCGTTCGCACACCGTTAGGATCCCGAGACCTCCCTTCGTCCTCGACCACTTCACCGAGCAACCTCTTGCGGAAGCCCTCCTCAGCCCTTTTCTCCTTGACTCTTGTTAAGGTAGAAGAAGGACCTCGGTTAGGATTGGTAGAACCAGGGTTTTCTGTAATGTCGCTGACTCATGTGAGTAGTAACCTAGGCGATTCGAAAACTTATAAAATCGCCAGGACCCCCGTGCAAAGTGGATTTCCATTTAATCAATTCATTATTCTTTTTAAAATGACAGAGAACTTAGAAAACCCATAACTTGATGAAATCTTAATAAAAGTTGTCAAACCAATTTTGCTAGCTCTGAATATTATGATCTATCATTTAAAAATAGTAACTCCATGCTTTCTGTTCAAAATTTTAAAGTTTAAAATTAAAAAGTAAACCCACTAAACCTTGTTTAATTAAGGAAAATTAGTTTTCTTGAGGTGAACTTAAGAAAATTGTAGATGTTTAAACCTTATTTAGACACTATTTAAAAATAGTAAAGGAACAGCATTGAAAAAATATGATGTAGTTTTTCATTTAAAGCTAATTTGTCCAAAACTTAGAGAAAATCGAAAAGGCCTTAGAAATTAATGAACAGATTAATAATATTTTTCCTAGTTTACTTATGCAGTAGAACCTAGGAAAATACGAGAGACCATTAATTTGGACCAGTTTTAAATTAAGATGATTTGTTTAGCCTCATATAGTAGAAATCAATTATTAGAGTTACAAAACTATAACCAAAATGATTAATAAAAATCTAGTAGACTTATAACCACTAGAGCCCCACTACAAAAATACAGAGCACCTCACCAACTTTTAAGTAAGAAAATAAATACAAGGTGATAATAGACATTTTTCAAATAAATCATGAACAACCCCTTTTAATGTGATAATAGGCAACCAAAAATTTGCTAAGCTCATGAGGAGGTAAACTACAGAGAAAAATGCAAACCCATGAAGAAAGTAAACCCATACCTTTTGCTAATAATTAAAGAAAGACCACTTAATTCAAATAATGCAAACCACCCTTTATAAGCAAAAAGAAGCCAAACTTAGAATGATTGCTCTTGCACAAAAAATACTAACTATGAAAATAAGAACTCGTTGTTTGATATTTTTCAAATATAGTGGTAGTAGAAAGCACCCATTTGGCTAGAAACTTAGAAAAACATAGAAAATTTATTAAGAAGTATTAATGATTAGAAATTTGTATCAAGTCATGTTATAACACCTAAAAGCCAGCAAAATAAGTTTTAGAGAATTACCCACTGTTAAATAGTAGTTGTAGTTCAAAGCACCCTTCTGCCCCTAGAGTTTAATAATTTTGTCCAGAGAAACTATTCACTTTCTGACCCCCAAATTTTGAGACAGGAAACATACACAGTAACAAGCCCTGTAATTTTTGCAGAATTTTTAGAATTTTATATAAGCAACTTGTAGTTCAAACTTCTCCCAAAACATTAAGAGAATAAAAGAAAAGAGAAGAAGGAATAAACCTCATCCCAATAAGTCTAATTTGAGAACTTATCATTTATCCCTAAGACTTAAAAGGAATTCAGTGGAACCCCAAAATAAACCTACCACTTACCTTAGCTAAGTCTAACAATTTACCAAGTATACCACTAAGGGTTTTATAAGTAAAGTTAACTTGTTTTAAACTCAAAAGATCATACACCTTTAAAGTTGTAATTTAAAGCACATATCATATCATGCATATATCTTACGACATTGCATTCATTAGATTGTAATCTTGCCGACGAGTCGTGCTCATCCCCGAGCAAGGACACATCCAAGAGGAGGACCAGGCGAGCTTTGCTAATAGATCATATTTGTAATTGAAGGCAAGCCGGTTTTATGCATAACCATGTTATTATATGCTACTACTACACTTAATGCTTATAGGATTGCAATGTGCACTTAAGTGTAGGAGTTGCATGAACATAGGATTCCTTTGAGATGAATACTAGTATGCTAGTCGAGTAGCCGCTGCTAATCGGGATCTCGGTAGAAGTCAGCAGTGATTTTTCTAGCACTCGCGAGTCGAATTGATTGTATTCATCTTGATACGGGATCTATGTTGGTCTATGGATTTGGATCGGGAGGATGCCTTGTCCATGATACGGGAAAAATGAATTAAGGATTAATGTGTGGATACGAGTCAAGCTTTTGAACGCGTACTAAGCATGTCATGCGGAAAAATGGTAGCTGTAAACCTAGTTACGAGTGAAGCAGACACGGACTTTATCCTCATGCGACTGAGACTGGGTCTCCCATGCTAGCTATGGTGGGTACAAGTCACCAGTCCATGCCTGGCCCACTGGCCGAAGTCGTGGAGCATTATGCCAAGGTGAGGCACGGGTAGACGGGTCGATGGGGACGGTTGTCGTAATAAGTCGAGTACCGACATGCCGTGTGTTTAGGTTTACCTTGCAAGGTTTAAAACTCGATTCAGATCGTCTTGCTTCCAGCCTAATGAGGCGCGTTCATATCTTTGCATCGAGTAAGTGAAATGTGACTACATGAGATAACTTGTTGATTGATGTAATACCCACTTTATAAGAAAAGTCTAAAAGGAGAAATATATTCCTTTATATATATGTGTGTTGTCTATTTATTGTTTCATGAACACCTCATTAAAAAAATACTTAATAAAGGACATGCCATTAAATTCACGCATCATGATGAGTTTTGTTTGTTTATTACACTTAATAAGAATAACAATAATATGAATTAAAATATAATAATAATGAAGAGGATTGAAGTAAAACCTAAATTTAGATTTGGGTTGAAAATGTAGAAAGGAAAAAAGAATATTATACAAAACCAAAATAAAATTTATAAATACACAAATTCAGATCATTTGAGTATGTTCAAATTCATGCCTTTGATTTGGGGCAAACTACACACAAGATGGAATTTAAATCATGAGAAATTTTGAATTGGGAAAAGAGAGAAAAGGAGAAGTGAAAATATATAAAGAAAAAGTAAGGTTCCCACGCTGCCGGCTGTTTCACTCATGACCCATTTCCAACTTATCAGCGCGACCCACCCGGCGTGTGCGACTTCGCGGCCCCACCGTGCAGCCGCTAGAGCCTGTTCATCTCTCCGCGGATTAAGAGTGCGCGGCCGTCGCCCGCGGAACCTTTCGGGGACCCGGGATTCCTACCATTCGGCTTGGCCCAAACTCGCAGTCCCGGTCACCGCTTGTGTCCTCCAACTCTGCCGCTTCGCTCCCGGTGCCGAAGTCAATCGGAGGAAATTCTCACGGCCGCTAAAAATCGAATCGTCGCGGCGTGCCTTCTAGCCATAAAACTTGGGCGGCGCCTCACGCCTCATGTCCCAGCCACGCCCGCCCCACGTGTGCCGGTCGTGGAGATAGAAGAAATGGGAGATCTGCGCCAGCCACCACCACCATTGCCATTAAGTACATGGAAGGTCACGAAGATTCATACTGAAGAGGTTGGTGATGTGTGGTAGCGAGCATCGTTGTCATCGCGAACCTTCGCTACCGCTGTAACTACGGATCGGTGGTAGAGCTCGGTGAAGATTCGCCAGATTCCGCTATCGAGATCC

>nano3

AAAGCACATGGTGAGAAGAACCTGACTAAGCGAGAATCAAAGTGAAATTCCCGAAAGGAGAATCAACTTCCGCGGTCATTGGCTACATCAAGCACAACTTGGTGCAAGGTCATCAATTTGCCCCTAGAATAATGTCCATTTTCCAATCCCAACACAAGAAAGTGGTTTTGATAATGTGGCTTCGATTGAATAGGCACACTTTGGTTTTATTGATTAGTTGCAATTTTACCCGTTGTGACTATCATGAATGACCTTTTGAGTGAGAGAATGGCGGTTCGCGTTTAGCACGAACTTTTGGCTAATGAAACTATGAGATGCACGAGAATCAAGCAGAGTGCAAGTTGATTATAAAGTGAAAAGATCACAGGTCATGATAGGAGCTCCTTGCACTTCCTCTAAGCGGTGAAGTTGAGCTTCCTTGCGACTTGCACCTATACCTTTCTTCCCTTGTTTTGATTTGGTCGGCATGCCTCTCTTTGGTTCTGGGACAATTCTTGGCATAGTGGCCGGTTGCCTAGAAGTGAAACCTTGTTCCCATTGCCACAAGCGGGGCTAACTGGCACCTACGGAGGTGATTGTTTCTTAGGCGAGGCGGATAGCGATTGGTGCCATTCTTTCTTCTTCTTTGAGGTGCTGATCACCCATCTGCACACTGTATTTGAAAACCCCTGCTCTTATTGTGAGAAACAATGGAACCTCACGAGCACGGCGGATGGTGCGCCATTGGTGCCTTTCTCTTCTTCTACGCGGTGAGCAACAATGCAATCTTCTGGGGAGATGGCCATGTTGACCAACTCATTGAAGCTATCGGCCGGGCCGGTGTTGAGTCGTTCGCAGACTTGTATTGAGACCCTGCGGGCGATCCTCTTCTTTCATCAGAATCGACGCGATACCTGCATCGGCATAAGTCATTGAAGGCTTGTGCATCTGCCCGTCCGTCGCGGGTTCCTTGATTAAGGGCGAGAAATTCATTCAACTTCCGATCAAGAATGCCGTGGGATGTGTGTCCACGAAAGCGTCTTGAATTCCTCCCAAGATACCTCACGATCGTGGGGGCATAGCGCAGTGGTCCCAAAGTCCAAGCGAGGCGCCCGAAGGCTGTGTGCGTGGCGAGCCTTGGTCTCATCAGCGATCTCACGTGAGGAGAAACTGGACTCGATGGCGAGCCACACGTCGGCGTCCAACGGATCCTCTCGCCTTGGTGAACAAGGCGGTGCGTGCTCGAAACTCTGGTATGTTGCCATAGCCGGAGGTCGATCTTGGCCTCCACCATGATCTCATTGGTGGTCGTGCACAAGAGTGTCGCGAGAATCTCATTCTGGCGGGCCATCGACTCACACCTTTATGGGAGGCTGAGGAGGTGGCGAGGCTTGCTCGTTCTGCCCATGACATTGCTATTATACATCAGGCAGAGATTGTTGCCATTGTTATCCTAACTCCAGTTTGAGCGACAAGATATCATCTCATATGGAAGGAAAAATGCCATAATCATAATATTAGGTTCGAAATGAAGATAACATGGTGACAAAGATCCCGATTTCAAAAGTTTATAGGGTTACATCAATCGGGAAAGTACCACAAGCCTAGTCCAAAATGTGATACTACTAAGCTCATAGGTTTCTATCCGCCTAAAATGTCAAATGACGCTTAACCACGAGCGGTAGAAGCGACCGGATCTGAATTGAAGGAGGCATCGCGAGGTAGTCCCATTGGCACCGGAGTGGTCTAGCTCCTCGGGGCTCTTCTCCTCGCTTCCTACTTCATTGGCCTCCATCTCCGGGTGGTGCATGTCCAGAGTGGCCATTGGCTCGAGTTCCTACATCGTGAAGTGGTTCTCAAGACATCAATTGTGTTGTCTCGGATCTCCACTTCTTTGCTCCAGGGTGGTATCTGCGGCTCGGCCTTTCCACTGCAGATCCTTCTCCACCAACTCTGTGGATAGGTCGACCACAAAATCTTCATTGTCGAGAGTGAGCTTGGCGACTGGCGGTGTTAGCAAAAGTGTCATAGCATCGCTCTGAAGGGCTGAAGGCGGTCTGTGCCTCATGCAGCGACATTGACCCTCCCAACCAAGTCAGGATACATTGCCCACACATCCTTCACATGGCTCATGCGGTTACACCACATGGGATCATCCTTCTTCTCGTGAGGAAGAGTCCCAGGGGTGCATCACCATCTCGGGGATGGTAGCCACGAAAGTTGTCGAGTCTTCATGGCATTGCCTCAACGGTGTCGTCAGTCCCAGTCAATCGTCTGGAGTCAAAGCATAACCGGTGAAGGATGAGCCTCAAAGTCGGCCGGACGGAGCAACAAGGTACCGTGCTCCTCATACAAGCCTGCAGGTGTACAAAGGCGTAGGGTAACCGAGGGTTAAGCACTTCCCACAAGATGGAAGGAAAGCCATCGCGAGAAAGGAAGTCGAAACTGGCTTGAGTCTCCTCCTTTGCGGGGGTGGGTGAATTCATCCGGAAGGGAATCAAAATAAAGATTATGGTGGAAGGAAAAGAAAAAGAGAACCGGATGGTTTTTAAAGAAAGAGTTAGCTCAAATTTTAATTCCTCTTTGGGGTTTATAATGCATGCATCTTTGGAAAGCGTTGCCTCTCAAAGAAAGATAGGGTGCCTTTTAGGGCATCGTAAATATAATGTACAGGGCCTAATTAATTAGCCACCTATTTCTCCTCTATGCCTAAGGCCTTTCGTCCCTAGGTCTAGCGGTCTAGTCTGTCGATATGTGATTTCTAGGCAAGTTTAGATTTTGAAAATTAGTATTCATGGTTTATTATCCTTCGTGGTGGAATTTTTCCGATACCGGTGTAGCGAGAACCACCCGGGTATTCGAGTTTAAGTGCTAAGTCACGCCCAGAAGTCGTAACACTTAAATCGAATAACCGTCGATCCCTCATATCTAGTCACATAGAGCCACTTAACGAGGATCAAATTCACAATCCCACTCGAAGGTGAGTCACAGGAAGAAATACAAAAACAGAAACCTCAAATTAAATCTTGAGTTATTACATAAATCGGAGTTTTTGAGTAGCAAATAAAGTTCACAAAATAAAGTCGTGGATAATCGATGTCGTCGGTGTAGGGGAAATGGGCAAGGCTAGCCCACTACTCTCATGCTCCTCTCTGCTGGAGCAACATCCCTCAGTACCAACCCGAGTGGCGAGGTGGTAGGCCAAGTCACACCATCACCAATTCTGTATGGTACACAAAAATTGTGCCACAAGCAAGGGTGAGTATACTAATACTCGCTAGACTTAACCGGTGTGAGTCTACTCCTCTACCTCTGAACTATGCATTTGTTATTGAGGGTTTGGTTTGCCAAAGCACTATTTGTTTCTAAAATCAATTTTTAGCTTTTCAATTTCTACCATCATTAACTTAGCTAGATTTGCTCCTTCTAAGCATACATGGTAACAATCAATTAGTTCAATCAACAAGTTATCTCATGTAGTCCATTTCACTTCTTACTCGATGCGAGACAAGGAATCAGCAGGTCTCATTAGCTGCGAAGCGAGCGGTCGAATCGAGTTTTAAACCTTCAGGGTAAACCTAAACACACATGTCGGGGTACTCGACCAGCATGACAACCGTCCCCATCGATTCCCGTTCGCGTCGGGCCTCATCGCCTTGGCATACAATGCTCCACTAACCCATTGCCGGATGGGTGCACTTGTACCCACCACCAGCTAGCATGGGAGACCAGTCTCGGTCGCATGAGGATAAAGTCGCGCTTGACTTCACTCGAGTACTAGGTTTTCTGGGTTACCATTTTCGAAGCATGTGCAAATGCGTTCAAAGCTTGTCGGTATCCACACATTAATCCTTAATTCATTTTTCCGTCTCATGGACAAGGCATCCTCCACGGATCCAAATCCATAGACCAACATAGATCCGTTATCAAGATGAATACAATCAATTCTGACCTCATGTGAGTGCTAGAAAAATCACTCGACTTCTACCGAGATCTGATTATGCGACTACTGACCTAGCATACTAGTATTCATCTCAAAAGGAATCCTAAGTTCATGCAACTAGAGGTTTCAAGCAACTCCTACACTTAAGTGCATTGCAATCCTACAAGCATTAAGTGTAGTAAAGTAGCATATAATAACATGGTTATGCATAAACCAGGGCTTGCCTTCAATTGTTGGTGGGGAGATCCTCAATACGACCTCATTTGCTCTCTGTCCTCCTCTTGGGCGGAGTCCTTGCTCGGGGATGAGCAAGTACTCTCCGTCGACAAGATTACAATCTAATGAATGCAATCGTAAGATATATGCATGATATGATATGTGCTTTTAGAAATTACAACTTTTAAAGGTGTAGGATCTTTTGAGTTTAAAACAAGTTAACTTTACTTATGTAAACCCTTAGTGGTATACTTGGTAAATTGGGTTAGACTTAGCTAAGGTAAGTGGTAGTTTATTTTTTGGGGTTCCACAATTCCTTTTAAGTCTTAGGGATAAATGATAAGTTCTCAAATTAGACTTATTGGGATGAGTTTATTCCTTCTTCTCTTTTCTTTATTCTCTTTAATGTTTTGGAGTAGGTTTGAACTACAAGTTGCTTTTATAAAATTCTAAAATTCACAAAAATTACAGTGGCTTGTTACTGGTGTATGTTTCCTGTCTCAAAATTTGGGGTCAGAAAGTGAATAGTTTTCTCTGGACAAAATTATTAAACTCAAGGGAGAAGGGTGCTTTGAACTACAACTACTATTTAACAGTGGGTAATTCTCTAAAACTTATTTTTGCTGGCTTTTAGGTGTTATAACATGACTTGATACAAATTTCTAATCATTAATACCTCTTAATAAATTTTCTATGGTTTTCTCAAGTTTCTAGCCAAATGGGTGCTTTCTACTACCACTATATTTGAAAAATATCAAACAATGAGTTCTTATTTTTCATAGTTAGTATTTTGTGCAAGAGCAATCATTCGAAGTTTGGCTTCTTTGCTGGGGAAGGGGTGGTTTGCATTATTTGAATTAAGTGGTCTTTCTCTTTAATTATTAGCAAAGGTATGGGTTTACTTCTTCTTCATGGGTTTGCATTTTCTCAGTAGTTTACCTCCTCATGAGCTTAGCAAATTTTGGTTGCCTATTATCACATTAAAGGGGTTGTTCATGATTTATTTGGAAAATGCCTTATTATCACCTTGTATTTATTTTCTTTACTTAAAAGTTGGGTTGAGGTGCTCTGTATTTTTGTAGTGAGGCTCTGGTGGTTATAAGTCCACTAGATTTTTATTAATCATTTTGGTTATAGTTTTGTAACTCTAATAATTGATTTTCAGTCTATATGAGGCTAAACAAATCATCTTAATTTAAACGGTCCAAATTAATGGTCTGCATTTTCCCTAGGTTCTCTGTTGCATAAGTAAACTAGGAAAATATTATTAATCCTTGTTCATTAATTTCTAAGGCCTTTACGATTTTCTCTAAGTTTTGGACAAATTAGCTTTAAATGAAAAACTACATCATATTTTTTCAATCTTTGGGTTCCTACTATTTTTAAATAGTGTCTAAATAAGGTTTAAACATCTACAATTTTTCTTAAGTTCAGCCTGGAAGAAAACTAATTTTCCTTAATTAAACAAGGTTTAGTGGGTTTACGTCTTTAATTTTAAACTTTAAAATTTTGAGCAGAAAAGCATGGAGTTTACTATTTTTAAATGATAGATCATAATATTGGGAGCTAGCAAAATTGGTTTGACAACTTTTATTAAGATTTCATCATGTTATGGGTTTCTAAGTTCTTTGGTCATTTAAAAAGAATAACAAATTAAATGGAAATCCACTTTGCGAGAGTCCCACGATTTTCTAAGTTTCCCTCGCGAATCGGTCCTTAGGTTACTATTCGAGTCAGCGACATTCTGAAAACCCTCGGGTTCTAATCCTAACAGAGGTCCTTCTTCTACCTTAAGCGATGAGCGGCGGAGAAAAGAGGCGAAGAGGCCTTACTGCGCGAGGTTGCTCGGTGAAGTGGTCGAGGACGAAGGGGAGGTCTCGGGATCAGCGGTGTGCGAACACCCGTCGGAGATGGCGGAGTCGGTCGATCCAGCAGCGCGGCGAGGATGCTCGTGGCGGCGGGAGGCCGACCTTGTCCGCAGCGAGATAGTTCAATTAGATGGGTCGGGGAGGTTCACGGGATGCGAGAGAAGATATGCGAAAGGAATTGGCGGAGACTCAGCTGGATAGCTCGGTCCACCTTGAAGACCAAGTCCGGTGGTCGATCTCGAGCCTCCGGTGAAGTCTGGCCGGGTCAGGAGGCAGCAAGCTTCACAGAGCTACTAGCGGAGCTAGCCAGAGCCACAGGTGGGCTTGTGGGTACAGGTGGGTGGCCATGGTGGCCGAGCTCTGTGGCAATGGCAATGGTCCTTCGCGGAGCTAAGAAAAGGTGGTGGCGGGAGAGGGTGAGTCTTGGCGAGAGGTGCGCCCAGGAGGCTTATAAACGCGGTGAGCCTGCAGAGGGCGTGGCGCGGCGGACTTAATGGAGACCGGAGTGGCGGCTAGCGCGGGAGTTAGGCGAGCTCAGCGTCTCGAGACAGTCAGCTCGATGTGCCGTCGTTACGCCCAGGTTACGCGTGTGGTCGCTCATCCGAAGCTGCTCGCCTTGGTCAGTGCACAAAACCTCTTCTCCTCCCTACAAGCTACCATTCTTGTGTAGGGTCATAGGTTTTGCCCTCGGTTCAGAGATATGGAGCGAAATCTGTCTGTCTCCCCTGAAACCAGAGGCAAATCAAGGTTTTGTCGTGTCTAGGGCTCAGCGTCCCAATGCCATCTTACGGCACAGCCAGGAGGGTTAGTTAGACACAATTTTGTCAATGGGCCATTAGGATTCAAGTTAGGGATCAAGGTGAACATCCCTGATCTTTGGCTCAAGGTCTGAATTTGAGAATCACAGAATCAGAAATCCCAATGAGTCCCACATGAGGAGCTTGATTTGGGGTTTTATTTGAGTTATTTTGGCTAAGCTTTCTCAACATTTCTTGTTGCCCATTAAATATTCTTTAATTTTCATAATTGGCTCAAGTCATAATTTTAAACTTTTCATTCCCTTTTCTTATTTTCTTGAATTTTGCTCATGGGGCTCACTTAGGGTTCTTAATTAGGTTGCACATTTTATCTTTTCAAAGACTCAATTGTTTTGATCATGTTACTTTTAAGTATTTACTTGGTGAATTCTTTATTACTTAAGTTGTTTGATGCTCATGCTTACTTTGGTTCACATAAAATGATGGTTCTTGGTTTGGCATTTGAGAAAACCACAGTGACCTTTAGGGTGTCAAGTCTGAGCCACAGGGTCAGGCGAAGCGGAGTTAAATCGTCTTGAAGTGAGCCCAGTCAGAACCCAGGTCAGGCGGGCGGAGTTCGCCATCTTAGGAATTTGAGCAGTCAGAAACCTGGGTTGGCGGGCCAGGTTAGTCTTCGGGTGAGCCGTAGAACCCTAGGTCAGGCGGGGCGGAGTTCGCCGTCTTCGGGGTTGAGCAGAGTCCGAGCCCTAGGTCGGGCGAGCGGTCGCCATCTTAGGAGGCGAGCGTCCGAGCCCTAGGTCGGGCGGCGGAGTTTCCTGGTGCTGAGGCGAACCTGGCGCTGTCACCTCACTCTGTCGAGTGGCACGGCCCGATCGGGCCGCGGCGGCCCGACTGTCCTGTCGGTACGGTCGATTGGGCGCGAAGTATTCTCGCCGGCTCTGTCGGCGGGCGTCGTCGAGATAAGGTGTCGAGCCACCTGCATTAAATGCCCTGCGATTTGGTCGGTTAGCGCGCGCGATGGCGAGTTGCTTCTTTGGCGAAAACCGGGCCTCGGCGGCCGGATATATTCGTCGTTGGAGAGGGCCTCGGCGAGCGGAGATCCTCGGTTGTTGCCTTGCCCGGCTAGGCTCGAGCGGCGTGTCGAGTCCCTCGAATGGACCGATCCACGACTTAGTCGCACCATCGAGCCTTTGCGGCTTTGTCGCGATGGGGTTACGGTGAAATTAGGAGTCTTGAGGGTACCCCTAATTATGGTACCGACGTTAGCCCGAGCCTCGAAGAGTGTTAACACTGCTGGAGGCTTTTGTCGCACACCCAAGGGACCACCTTTCTGTTGCGTTTTGTTCGGTGGAGTGCTGTGGCAACCTGGCGATCTCTTTCAAGGCCTCGGAGTGGTTTTTGACTCCTCGAGGTCTTAATACCTCCGCAATGCTTGGCCCGTCGGTCGTTCCCTCATCGGTGGCCGTGCCAGGGTGCAGTCGGTCCCAAGTTCTTGAATGGTATGTTGGCGTTGTCAGCGGTTTGGCCGAGGCCGGGTTTGCGAAGCAAGAGCTCCATACAGGCGAAGGGCAGTCGAGACGGACGACTTTTTAACATACGCCCCTAGCGTGCGCCTTCGCAAGGAGAAAGCGCCATGTTGCCCTCGGGCGCCAAACATGGTGTCTCCGTGTTTCTTTGTGTCCGAGTGGGCGCTTCGTGCCCCATTTGTTAGAAGTCGGCTAGAGGCGGGCGCGCTCAAAAGTACCTCTGGGTGATCTGGACCGGTCCTGTCGACGGGTCAGGCTCGATACTGCCTCCCTCGATGGGATTAGTTACAAGATCATTCTGCTGGTCGGAAATGTCTTAGGGTACCTCGGGGCGTGCGGAGCCTTGGTTATGTCGAGCGTACCCATGGTCATCCCTCGCTCTGTGTCCGAGCGGTGTTGTGAACCCTTCGAGGGCGACCTTGAACCCACGATCGTAGTGGGCCGAGGCCGTGGCTGAGGCGGCCGTTGAACCCTCAGGGTGACCTTGAACCTCTAACGATGGTGGGTGTGGAGCCCAGCGCGCTGGCAGTGTTGTTGAACCCCTCGGGGCGACCTTGAACCTCTTATCGGTGGGAGGCTGGAGCTGTTTCCTTCGGAGAAGGATCCTTTTGGGGTATCCCTTTCGGTCCTGTTATAAGAGAAGAAGGAAAAAAGGAAAGGATGCAAAATCGAATGGCGTGGCGTACCTCTTTTGTGCGGTCATTGTGGCGGCGGCGTCCTTCGCGCTTCCCTGCGGGCCCGCGCGCCTTTCGCCGCCGGAGTTAATCGCAGCGAAGCGGCGGTGGTGGCCGCTTCTTGGCGTAGCTTGTGGCCGTTGAGCGGAACGCAGGCGCCGTCTTCACCCGTGAGAGGTTCTCTCAACGCCCGGAGATGGGGCGTAACTTGGTGGCAACGTGCCGTACTCCCGCACACGCCACCGCCATTCACTGCGGCCCATTTTGGCCGTGACGACCATCGCGCTTTGGCGCCTTCTTTGCGGTCGTCTGCGAGTCGCCTCAGTCGTGATGCCGGTTCCGCTGATCGAGCGTGGCGATGGTCGGTCGTGCGATTGCTTGCGTGCGGCCGTGCGCCCGGTGCGTGCAGCGTGGCGTGAAACCCCATCTTCGTCGTTAAATCGGAGGCGCCTAAGCCTTGATTGCATACGCGCTGCATGTTGCCCGCCCTTCGCCCAGCGGATGCGGCGAAAGTGGAAGAATCTTCGTGCCATTGGCGGTTGCATGCCATGTGCGCGGTTTGGCTAATACGCTGCAGGCGGCTTAATGCGGCCAGCGTGGGTCCCGGCCGCGTCGTAGGGGAGGACCTTGGAGCGTGTTGGAGAAGACTTCGGCCACGGCTTGGGGCGCAGTAGGAGAGTCGCTAAAGGTGGGTGACCCCTTGAAAGGCGACCATGTCTTCAGCTCCCTTATGTATCGTGTCTTCCACCTTAGAGCCGGATGGGAACACACATGATCCCTCGCCTTGTGCGTTGGAGGAGCGCAACTCCGTGGGAGTTGGTACCTTTAACCATCGTTCGCTTCAAGGTTTTCATCACAGCGGCCGGCTGCACCCCTACTGGCGGTCACCCAAGATGGTGACCTCGGTTCGATGGCGGGAAAGCAAGCGAGGCTGCGGCCTCGCCTCCCTCGGCCTCAGGATTTTCATCGCCGGTCTTTGGGGAGGAGTGTGTCGAGTTAGAGTCGGCCCGCATGGCGGCGGCCCGCTCCTTCCCTCGATGTCGAGGGAAGGGCGTTCGCCGTTCATGCGGCGGTGCCGCGTGCACGCTCTCTCGGCAGTGGCCGGCGCTCCGGCCGCTTCCGCCATGGCGGTGGAAGAGTTCTTCGCCGCAAGATAGCGGGAGCATACGAGATGAACCTCAACTCGACCTTCGCCGTCCTTGCCTCCGAGTTTGGGCATGGGCGAGGGCCTCATGGCGGCAGCATCCACCGAGGTCATCGGCTACTCTGTTAAACCGGAGCGAAAATCGTCATCGTCGCGTGTTAGGCGCGGCGGCAGCCGCTCGTTAGTGTGTTGCTCGCTGTTCCGTCGTGTGAAGTTTGTTCGTACTGCGGAGGTGGAGCCGAGTTCAGTTTGTAATGGCACCTTGAATGTCGGTCTTTTGTTCATTGTGGTTGTCGGGGCACGAGCGTGTAGTATTTTTGCCTTGAGCCGTGTTTTTCCTTATTTTGAGCACTAAGACTACTTTGTTGGTTGTGCGAACCTTCAGAGTGAGTCGCCCGTACAAGGTAGTGAGGTGTCCGTATCCGGGAGCGTAAGGTCCTCGGCTCAGTCGGCCTTCTTTGTCGAGCTTCTCTTGTAGTTAAAGAACCCCTGTGCGCTCTTGATGGCCGGCAGTAGCGGTGTCGCATTGAGCGAGGCGAGTTGGCTCGAAAAAAGACTTGGTCGGCCGGGCTGGCCGGGTGCGTCCGATTAGCGGGGCCAAGCGCGAGTTGATCTGCAGGAGCCTCGGCGGTGATGTCTTCGAGACGGTGGCGAGGCCTCGGGTGACGGTGCCGCCTACTGGCCGGATTCTTGGAAGACCTGGCGGCGATGGCGAGCGTGATGATGTCGTCGTTGAGTGGAGATCCTCGGACCGCGTCGTGCGTCAGAGGCTGGTCGGACCTCGTGAAGGTGTAGTCGGCAGAGGTCTTCTGCTCCCTTCAGCTGTCAAGATCAAGCTGCGAGGATCGGATTATCTTGTAGTGTGCGTTTTACTGTGAGGAATAAACACACCATCGCAGTGTTGTAAAGCTGCGCTTCTTTTCCTCTTGTTTTGAGTATCTGGACTTTTTAGTCATTAGCGGAATTATGCTGCGGCGGGAGTTGCTTTTCACGGAAGGTAGTGAGGTATCAGTATCCCAGTAGGAATCCTCACTTGGCCTTCTTGCTTCGTGCACTCTTGCCCGTCCATGGGTTACGCCGTAGCGATCGAGAAGGGCGAAGAATCGCCGGAGGAGCTTTGAGTGTGAAGACTTGTTCGATCTGCAGATAAGCTTATCCGAGCGAGTTACTTATCAGAAGGTGATGAGTGAGGTATCCGTATCCGGGCCGTAGGAGTCCTCGGCTCGGCCGTTGGTACCTGCTTATTACTCCGTCGTTTTGGATCCACTTTGAGTAGTCGAAAAGCGAAAGACATTACGGAAAGGATCTTTTTGAGGAAAATTTCGGAGAGGGGTTCCCCTTTTTAGCCCAGGGAGGTCGGGCTTTGAGAGCAAGGTGACCCTTCCTTGATGACTAAACTTTGGTAGGGCGAGTATATGAACAACGAAAGCATCTTAAGGGTAGAAGCGTGGTGTTGGATGTTCCAAGCGTTCTTCGTGGACCTCGCGTATTGCGGCTTATGCGTTAAGACCAGAACTTTGGCGATAATGACGGCCCTTCGAGAGGAGCGTGAGCTTGTGCGCCCTCGGGCGTCTGTCGCGGCGAAGCACCGGGCGCCCGCGTGGAGTCTCGGGACCGGACCCCTCGGGTGTGGTAGCGTCGCGGGCTTGTGCCGTCTGGGAATGTAGTAAGGCCATGTCAGAGCCTCTTCCAGGTGGTCAGCGAGTCTTCTCGACTAACTTGGTTGCTTTGGTGGCGTAGGCCCTCGTCCCCAGGGGCCATTACAAGTCACGTGGGCAAGATGGCCTCGGCCCCATAGACTAGAAGAGCGGCGTGAAGCCGTGGCTCGGCTGGCGTTGTCCTCGGACTCTAGAACTGCAGGGGAGTTCCTTCATCCCGCGCGAACTGTTGAGGTCGTTGTAGATCCGAGCTTGGTCCTTGTAGAATCATGCAGTTGCGCGCTCTACTGCCCATTCGTATGGGGTGAGCCACGGCGGCAGTCCACCGGATGTGGTGATCCTCATGAAGTCGGGAACTTTGCGGTGAGCGGGTGCGGTTGTGGTGATGATGGAGTTCGGGACTGAAAGGGAAATGTGCCTTTGGGCCATTTCTAAGTATTTTGGTGATTGAGTGCAAACACAAGTGCTTAAATGTGAAAATATGCCCAGGATGAACAAAGTGCAAATCACAAGTTAAGGTATGTTTCTAAGCCTTAGTACATTGGTTTTGTGTACTAATATATTTGTCTAAGTGTTAAAAGCAGATAGAAGAAGAGAAGAGAAGACTTGGTGTATGCGGCGGCTGCTGATGCGGGCACCGGATCGTCGGTGGTGCCGGACAGTGTCGTGGTGCCGGACGATGTCGTGCGCCAATTCTCGGCGAAGAGGCCGCTCTCGGGTTTTCTCCGGCGACTTGCTAAAATTCACCGGTTTGTCGGTGTGCCGAGCCATCCGTTGAGCCACGATCGGCGGCCAGCGGTCGGCCAGCCTTGATCGGTAGCGACGCATGGCGAGCCAACGGTCGAGGAAACCGGGTATCGGTGTGCCGAATCGTCCGGTCGCGCGGATCTGCGATCGACAGCGGTCGGACCATTTAAGAAACAATCGAGGCGGACGGTGTCGGTGTGCGATCTGTCCGGTCGGCGAGAGGCATGAAGGCAAAGATGGCCTTCAGTTTGTTCCCACGGCTCCTAGCTGCCTTGGGGCTATAAAAGGGACCCCTAGGCATGGAGGAGTACACAAGCATTCCTACAACTCTTCTAAGCACAAGACATCAATCTCACGCGTCGTCATTGTGATAGCATATAGAGCTCTTGTGGGTTGTGAACTCTTTGTGTGTTGCGTTGCGAGCTCTTGTTGCGACTTGTGTGCGTGTTGTTGCTCGATTTTCGAGTCTTGTGTCGTTGCTCATTCCACCTTACTCCGTATTTCTTTGTGAACTCAATTGTAAGGCGAGACTCAAGTTGTGGAGATTCCTCATAAGCGGGAAAGATCAAAGGAAAGAAAAACACCGTGGTATTCAAGTTGATCATTGGATCACTTGAGAGGAGTTGAGTGCAACTCGTCGGATTGGGGCGCCACAACGTGGAGTAGGCAAGTTTTGTACTTGTAGAACCACGGGATAACCAGTGTCAACTCTGTGATTGCTTTCTTGTGGTTATTGTGTTTTGAGTTCTCTCTAGCCACTTGGCCATACTTGTACTAACCCTTAACAAGTTTTGTGGCTTAAGTTTAAGTTTCTACAGGATCACCTATTCACCCCCTCTAGGTGCTCTCAATTGGTATCGGAGCTTCTCTTCAAGAAAGGACTAACGAAGAGATGGATCCTAAGAAGGGAATTGTGATCAGCGACAAGGAAGAAGGAGTCCTTCCGTCCAGGCCAGGGATGACAAGTCCAATGACTCGGGCTCGGGCCTAGCGAAGAGATGGGAAGAAGAAGACAGAGCGCATCAAGGAGATCGTCTCTACAGTGATGAGTCCTCTTCTTCCCAAAGGGCGACGACCACGACAAACAAAGGAAAGCAGGTTAATTCTAACTTTTCTTTTGACTACTCTCGTATTCATAAAGTTCAAATTCACATTTGCTTTATTCCACTCGCAAGCCCCACACTTTGATGGGAGGACTACGGATTTTGGAGCCACAAAATGCGTAGTCACCTATTCTCTCTCCATCCTAGCATATGGGAGATTGTAGATAGTGGAATGCACTTTAATAGTTCGGATAGTCCTATATTCATTAATGAGCAAATCCATAAGAATGCACAAGCTACTCATTGTTCTTCTAGCCTCATTGTGCGAGGATGAATATAATAAAGTGAGTGGCTTGGATAACGCTAAGCAACTGGATACCCTCAAGATCTCTCATGAGAAATGATGCTACCTTGCTCACCAAAATGGAGTTGGTAGAGGCGAGCTTGGGCGGTTCGCGATGATAAGGGCGAGGAGCAACTCAAACATACAAGGCTCAAGACCCTTGTCAACAAGATAAGGAGCTATGGAAGCAGCGCGATGGGCGGACCACGACGTCGTCGCCTCATGCTAAGGTCCTGCATTCTTGATCCTCATTTGGTGAATAATATTCGTGAGAATCCCGGTACACCAAATGTCGAAAGAAATTCTTGGAAAATTTGTAGCGGCGGATGATGATCAAGGAGGCAAGGTGCGTCGATGACGCGTTGAGCGGTCCAATCCACGAGCCTCAACCCATTGCTCTCAAGGCATCGAGGAGCAGGAGGCACTACCAAGCAAGGTGGTGCAAATTGGTAGCCGGGCTTAATGATGAAGAAATGGCTCTCATCATTAAGCGCTTCAAGACAGTGCTAAAGGTCGCAATGGACGGTAGAGCAAGACTAAGACCAAGGGGCGATCATGCTTCAAATGCGGTAAGCTTGGTCATTTATTGCTAAGCTGTCACGATAATGAAAGTGACGGAAAAGGAAACAAGAGGGAAAGAAGAAGCATTATAAGAAGGCAAAGGGTAGGCGCATCTAGGCAAGGAGTGGGACTGGATTGCTCCTCGTCCGACTCAAATGATGGACTCGCGCCACCGCCTTCAACAAATCAACCCTCTTCCCAGCAGTCACACATGCCTTATGGCAAGGGAGAAGAAGGTATGTACTCATAACTCTACCTATGCTTCTTCAAGTGAGGACGAATCTAGTGATGAGGATGAAGTAGATTATTCATGTTTGTTCAAGGGCTTAGATAGATCTAAGATAGACAAAATTAATGAATTAATTGATGCCTTGAATGAAAAGAATATACTTTTAGAAGCAAGAGGATTTGTTGTATGAAGAGCATGATAAATTTGTTGAGGCACAAAAATCCTATGCTTTAGAAGTTAAAAGAAATGAAATGCTTTCTTTTGAACTATCTACTTGTCATGAAACCATTTCTACTTTGAAAGGTGTCAACAATGATTTAAATGCTAAATTAGAAGTAGCAAATAAATCCAATTCTTGTGTAGAACATGTTGAAATTTGTACTAGGTGTAAAGATTTTGACGTTGATGCTTGTAGTGAACACAGTTTCAATTTCAAGCTTAATGATGAACAGCTAGTCTTAATGCTCAACTTAAGACTAGCAAGAATGATTTCGATAAGCTAAATTTGCAAGGATGCCTACAATTGGTAGACACCCCTCAATTAAGGATGGACTTGGCTTCAAGAGAGCTAAGAACTTAACAAGCCATAAGGCTCCCATTCCTAAGGAGAAAGGGAAGGTCCTATGGCTAGTAATGTGCAAAAGAACCATGCCTTTTGTATCATGATAGAGACAAACTAGAAATGTAAGTCATGATGCTTTTGATTCATATGTTTATGATTCTCATGCCATGTTTGCTCTAGTTCCTCTTATGTGTATGATAGAAATGTTACTAGAGAAATGTTGTTCCTAAAAGAAATATTCATCACCTAGAAAGAATGTTATTCATGCTCCTAGGAAAGTAGTGAATGAACCTTCCACAATTTATTATGCTTTAAATGCTTCCTTTGCTATTTGTAGAAAGGATAAGAAAATTGTTGCTAGGAAGTTAGGGCAAAATGCAAGGGACAAAACTTGCATTTGGGTCCCTAAGGATATTTATGCTAACCTTGTAGGACCCAACATGAGTTGGGTACCTAAACCAAGCCTAAATTTGCCTTGCGGTTTATGCATCCGGGGTTCAAGCTGGATTATCGGCAGCGGATGCACAAACCATATGTGGGGAGAAGAAGATGTTCACCTCCTCGTCAAAAATAAGGATTCCCAATTCAATTATATTCGTGATGGGAATCAAGGCAAGGTAAAGGGTTAGGTAAATTGCGATTTCTAATGAGCATTCTATCTCAATGTATTTTTAGTAGAGAGTCTTGGATATAATTTGCTATCTCATTAGTCAATTATGTCATATGGGATATATTGTCTATTTACAAATGTAGATGTGTCATCTTTAGAAGAAGTGATGGTTCACTAGCTTTTAAGGGTGTATTAGACGGCAAACTTTATTTAGTTGATTTTGCAAAAGAAGAGGCGGTCTAGATGCATGCTTAATGGCTAAGACTTACGTAATTGTTTGTGGCATCGCCGCTTAGCACATGTGGGGATGAAGAACCTTCACAAGCTTCAAAGGAGAACACGTGATAGGATGACTAACGTGCATTTCGAAAAAGATAGACCTTGTGCGGCTTGTCAAGGGGTAAACAAGTGGGAGGAGCACATCACAGCAGAAGCGTGATGACCACTTCAAGACCCTGAGCTCTTGCATGGATCTCTTGGACCTGTCGCCTATCTCGAGCATAGGAGAAGTAAGTATGGTTTAGTTATTGTTGATGACTTTTCCGCTTCACTTGGGTGTTCTTTTTGCGGATAAGTCGAACCCAAGGACCCTCAAGCGCTTTCTCGGAGAGCTCAAAATGAGTTTGAGCTCAGGTGAAGAAGATAGGAGCGACAATGGGTCGAGAGTTCAAGAACCTTCAAGTGGAGGAGTTCCTTGAAGATGAAGGATCAAGCACGAGTTCTCCGCTCCTACACACCACAAAGCAAAATGGTGTGGTAGAAAGGAAAAACAGGGCGCTCATCGACATGGCGGGGCGATGCTAGGGAGTTCAAGACCCAGAGTGCTTTGGGCCAGGCGGGTGCCCTTGCCACGCCATCAACAGGTCTACCTTCATCGCCTCCTCAAGAAGGCGTCGTATGAGCTACTAACCGGTAACAAACCCAATGTATCGTGCGTGTATTTGGGAGTAAATGCTACATTCTAGTGAAGAAGGGTAGAAATTCTAAGTTTGCTCCCAAAGGCTGAAGAAGGTTTTATTAGGTTATGACTCAAATACAAAGCGTATAGAGTCTTCAACAAATCATCGGGTTTGGTTGAAGTCTCTAGCGACGTTGTATTTGATGAGACTAATGGCTCTCAAGAGAGCAAGTTGTTGATTGTGATGATGTAGATGAAGAAGATGTTCAGGCGGTGCTATCTGAACCATGGCGATTGGAGAAGTGCGCACAGGAACAAGATGAACGAATCAATCTTCCTCAACAATGGTGCATCCCCAACTCAAGTGGGCGCTTGATCAAGGGGAGCGCAAGATGATCAAGTGATGGAGGAAGAAGCGCCTTAAACACCTCCAACCCAAGTTCGGCGATGATTCAAAGGATCATCCGTCGACCAAATTCAGGTGACATTAGCAAGGGTAACTACTCGATCTCGATTAGTTAATTTTGTAGACATTACTCTTTTGTCTCTTCTATTGAGCCTTTCAGGTGAAGAGGCCTTGCTAGATCCGGACTAGGTGTTGGCCATGCAGAGAGTTAAACAACTTCAAGCGCAATGAAGTTTGGACAGTGCCTCGTCAGAAGCAAAATGTTGTGGGAACCAAGTGGGTGTTCATGACAAGCGGGACGAAACAGGGTGGTGACGAGGAACAAGGCTCGACTTGTGGCAAAGGTTATGCCCAAGTCGCGAGTTTGGACTTTGAGGAGACTTTTGCTCTGTGGCTAGGCTAGAGTCCATTCAATTTTGCTAGCATATGCTCACCATTCTTTGAGTTGTTCCAAATGGATGTGAAGAGCGCTTTCCTCAACAGGCCAATCAAGGAGGAGGTGTCGTGGAGCAACCTGGCCGAGGATGAGCAGTTACCCGACCATGTGTGTAAGCTCTCTAAGGCGCTCTGGACTTAAGCAAGCCCCAAGAGCATGGTATGAATGCCTTAGAGACTTTTTACTTGCTAATGCTTTCAAGGTTGGGAAGGCGATCCAACTCTTTTACAAAGACATGTGATGGTGATTTGTTTGTGTGCCAAATTTATGTCGATGACATAATATTTGGTTCTACTAACAAAAGTCTTGTGAAGAGTTTAGCGAGGGTGATGGCAGTCGAGATGTCAATGATGGGCGAGTTGAACTACTTCCTTGGGTTCCAAGTGAAGCAACTCAGGGCGGCACCTTCATCTCCCAAACGAAGTACATAAGATCTGCTAAGCGGTTTGGGATGAAGGGCCCAAGCCGCAAAGACTTCCGATGGGGGCCAACGGACACACCGCCTCAACAAAGGAGGTAAGTCAGTTGATCAAAAATATCTGCGGTCAATGATAGGTTCTTTGCTTTACTTATGTGCTAGTAGCCGGATATTATGCTTAGCGTATGCATGTGTGCTAGATTTCAATCCGATCCTAAGGAATGTCACTTAGTGGCGGTGAAGCGAATTCTTAGATATTTAGTTGCTCATGCTTCTTGGGCTCTGGTATCCAAGGGTCTACCTTTGACAGTTGGATACTCGGACTCGACTATCTTTGGATGTAAGGTCGATAGGAAGAGCACATCGAGGGCGTGCAATTCTTAGGAAGGTCCTGGTGTCATGGAACTAAGAAACAAACTTCCGTTGCCCTATCCACCCCGTGGTAGGTATGTTCTTGCGGAACGGTGTTCTTGCGAACTACTTTGGATGAGGCAAACCCTCGAGGACTTTGGCTACAATCGAGCAAAGTCCCTCCTATGTGACAATGAGAGTGCTATCCATGTGCGGAATCCAGTGAACACCTGTACGCACAAAGCACATAGACATCCGGCATCACTTTTGAGAGACCACAAAGGGAGATATCGAGTGTTTCATGTTAGCCAGAGAACCATTAGCCGATATCTTCACTAAGCCTCTAGATGAGAGACCTTTGCGAGTTGCGTAGTGAGCTAAATGTCTTAGATTCGCGGAACTGGATTGAATTGTAGCATACATGTAGTTATGCTTTTGATCATGTTCCTTTTGCATTATGTTGCTTATTATGGTGCTCAAGTTGTACAAACACTCCTGGACCTCACAAGTCCGTTGCAAAGTGATGCACACGTTTAGGGGAGATGTGTTACAACTTGACCCTTTGAGACTAACCATTTGCTTGAGTTTGCTTGATTTAGTCTCGAAGGAGGATTGAAAGGAAAAGGTGGACTTGGACCATGAAAGACTTCCACTTGCACTCAGATGAGAGGTAACTAATTCCAAGTTCATCTCATGAAATCTTATTGCCATTTGCTCTTAATTGAAGACTTTGGTGAGGCAATGGGGTTAAAAGGCCAAGATTAATCCGTTTTGGTGCTTGATGCCAAGGGGAGAAAATAAAGCCAAAGTGATAAATGGATCAGCTACCACTTGAGAGATTTTGAAAATAGTAGAATAGAGTTTTGTTTTGTCAAAAGCTTTTATTGTCTCTTATTGTCTCTATTGTCAAAAGTTGGCTTCTTGTGGGGAGAAGTGTTGATTATGGGAAATAGGGGAGTTTTGAAATCTTTGATCAATCTCTTTTGGAATGACTCTCTTTATACTTCATCATGTGTGTTTGACTTAGAGATAGAGATTTGAGTTTGATTTGCAAAAACAAACCAAGTGGTGGCAAAGGATGATCCATATATGCAAAATTGAATCAAAACCAATTTGAGTTTTTATTTGAAGTGATTTTGCACTTGTTTATCTACTTTATGTTGTGTTGGCATAAATCACCAAAGGGGAGATTGAAAGGAAATGTGCCTTTGGGCCATTTCTAAGTATTTGGTGATTGAGTGCAAACACAAGTGCTTAAATGTGAAAATATGCCCAAGGATGAACAAAGTGCAAATCACAAGTTAAGGTATGTTTCTAAGCCTTAGTACATTGGTTTTGTGTACTAATATATTTGTCTAAGTGTTAGAAGCAGATAGAAGAAGAGAAGAAGACTTGGTGTGTGCGGCCAGGGCTCGATGCAGAGACGCGGTGTCGGTGGTGCCGGACGATGTCGTCTCGCCAGGCCGCCTCGTAGCGCGCTCTCGGATTTTTCTCCGCGACTTGGCTAAAATTCCGGATGTCATCAGTGTGCCGGCTGTCGGTGAGCCAGTCTGGCCAAGACCAGCGATCGGCCGCGATCGCGCGCGACACGTGGCCGAGCCAACGGTCGGAAAAGCCGGTGTCCGGTGTGCCGATTTGTCCGGTGCGCGGATCTGCGATCGACAGCGGTCGACCGCCATTTAAGGAAACAAATCGAGACACCGGGCGGTGTCGGTGTGCGGATGTCCGTCGCCACGAACGAAGGCAAAGATGGCCTTCAGTTTGTTCAAGGCTCCTAGCGCCTTGGGGCTATAAAGGGACCCTAGGCGCATGGAGGGTACACCAAGCATTCCTACAACTCTTCTAAGCACCAGACATCAATCTCACGCATTCGTTTCATTGTGATAGCATATAGAGCTCTTGTGGAGTTGTGAACTCTTTGTGTTGCGTTGCGAGCTCTTGTTGCGACATGTGTGCGTGTTGTTGCTCGATTTTGAGTCTTGTGTGCGTTGCTCATTCCCACCTTACTCAGTATTTCTTTGAACTCAATTGTAAGGGCGAGAGACTCCAAGTTGTGGAGATTCTACATAGCGAAAAAGATCAAAGGAAAGAAAAACAGTGGTATTCAAGTTGATCATTGGATCACTTGAGAGGAGTTGAGTGCAACTCTCGTCAGTTGGGGCGCCTGGCGTGGAGTAGGCAAGTTTTGTGGCGAGACCAGGATAACCACCGTCAACTCTGTGATTGCTTTCTTGTGGTTATTGTGTTTTGAGTTCTCTCTAGCCACTTGGCCATACTTGTACTAACCCTTAACAAGTTTTTGTGGCTTAAGTTTAAGTTTTTCAGAGATCACCTATTCACCCCCTCTAGGTGCTCGGGACACTGAAGCGATGGATGATGTTGGTGAAGAACGCCACCGCTTGTTCGGACGATCTTTGTTTAGGTCGGACCTCGATCCACTGGAGTATCGATGGTGACCAGGTCGTGTAGCCGGGGTGCTTTACGCAAGGGCGGCGAGATCGGACCCACGGCAAAAGGCGGGTGATGGGTATCGTCTGCGGGGGCTGGCGGCGAGTGGAGTCGCCTTAAATGGCCCGACACCCTTCGCGAGTGCGGACAATTCAGTGGCGTCGACCCCACCGCCGTTGCGATGAAACCTTGTCGGAAGCGTTTCCAACAAGGGCTGAGGTCTTCGCGTGATGGGCACCAAGCCCAGTGTATCTCTTGTAGGAGCTCGGCCTTGGCGATGGAAATGCATCGCTGGAGGATGCTGAGGGCTACGGTGGTAAACTCTTTCGAGTCCCCACCAAGACAAGCGACTTGCATAGCCATGGAAACCGGCTCGGTCGAGTGTCTCCTCGGTGGAGATATGCGGTCGGAGTGCGCGGTTCGATTAAGCGTGACCAGCCGCTCCTCTCGGCGTCGGTGCCTTATCCTCGGGGCGGCGGGTACCTCGGCGGTGAGAGTGCCTGGCGAGAGAGTGCCTCGGGTTGAGCGAGTGCTCGGCTGGTAGAGGTGCCTCGGCTTCGGACGTGTCGTCGATCTTGGCGGAGGGTTGATGCAGTCTCGGGAGAAGGCGTCGGGAGGCGTTGTTCGCCCCGAGGCTATTTTGTGGTGTAAACAAATTGGCGCTTAGACAACTTAATAACACATTGCGGACGTTTTTATGTACTGATGCTTGAATTAATTCGGGGATCTGGATTTAGTACTGGATTTTGGTTTTAGGAATTAGAAATTTTATTGATAGAAGTATTTTACAAATACAAATACATACTAAGGGTTTCTTATATGCTCAACACATGAGCGAAACCTATAGAACCTAATTCCCTTATCGGGAACTACTCACACATTATTATGGAGAACTCGAGCTCCTATTAGGCGGTGGCCTCAGCGTACTCGAGCGGCCCGAGGGCTTGGTGGCGGCCGAAAGTGAGACTTGCGGATGTGTGTGCCGGTGATGGAGTGGAGCGTGCGAGGCCGAGGAGGGTGAGCATGATCATGCGGTGAGTCGCCGTGGCGCGTCGTGTGATGCCACCAGGCGAGTGGCGAGCCCGATCACGGAGAGAGTCGGCGGTCTCGGCGTCGCGGAGGCCGAGGCGCTCAAGCTCAGCGCGGTGTCAGAATGCGGTCGCCTCCTTGAGGCGGAGAGTGGCCGTTCTCCCAGGTGGTGTCGCCTCGGCGAAGGCAGCAGCGGCGGTGAGGCCTGGCGTCGGTGAAGGAATCGCCGTCGCGAGTCCGCGGCGTGGAGAGGCGGCCACCGCGCGCCGGTGAGGTATCGCCCTCGCGCCGATGTCAGCGCCCATCTCGCGAACACGTTCGGCCTCCTTCTCGCCCTGGAGGTCGTGCTCGCGGAGGTTAGGCGCACTCGCCTGGGAGGAGGGCAGCGGCGGTGAGGATGGCAGCGAGCCGGGTAGTCGCCCGGCACAGCACGCGGCCTGGGCGGTACTTCTGGCCACCCGGATGGAGATGCGGCGGAGGTCGTCGGAGGCAGTGGCGCGCACGCCAAGTCAGAGAGGGTGTCGAGTGTCTGGCGGAGCGGAGCGGGGACTTGATGTCGCCGGTGAGGCGGTTCGAGTCCGTCCGGGAGAGAGGGCCGAGGAACATGAGGGCGGAGGCGTACTGGGAGGAGCGCTCGGCGGACACCTCCACGGTGCCACCGCGCACTGGGCCGGACACGGAGATCGGAGGCGACCGTCGTTGGACACCCAGGCACCGAGGCGTTCGAGGGCCTCAGGAGGTCGCCCTGAGGGCGCTTGCCGAGGAGTCCGGGTAGTCGGTCACGAAAGTGGTGCCAGAGGTGAGAGCAGCCACGCCCATGAGGAGGCGGGCCACCGCGGCAGCGTTGCCTGGGTTGAGGTCACACCGGCCTGTGGGCGAGCGCCGAAACCGCGGATCACGGCGTCATCGCCCACAAGCTCCACGCCAGCGCCCCAGTCGCGGAGGCAGCGGAGCATGGCCTCGGCGTCCTCAGAGTATACGCCCACCACGCGGGTCTCGCCCTCAGCGAGGGCAGCGCGGCGAGTGCGAGTGGTGTAGTTCTTGGATGGCTGAGCGCGAAGCTCGCCGCGGAGTTCGCGAGCTGGATGCACGATCACGTCGAAGGTGGCGGGCAGGGCGTCGGATCCTTGCGGGGATCGGTGGGGCCCCACGGCCGGGCAGGGTGGCCGGGGAGCCATCGGCATGGCGGGTGACGCCGCTGAGCACCTGATGGGCGCGGCGAGGGCGCGGCGGGTGGCAGGAGGTGCGCCCGGCGCCTCGCCTTGGGCGCAGCGGTAGTGGCGCCAGTGAGCGCGGTAGACGCGGCGGCGGCGGTGGCCATGGTAGATCCTCTAGAGTCGACCTGCAGTAACGCAAACAACAGGGTGAGCATCGACAAAAGAAACAGTACCAAGCAAATAAATAGCGTATGAAGGCAGGGCTAAAAAATCCACATATAGCTGCTGCATATGCCATCATCCAAGTATATCAAGATCAAAATAATTATAAAACATACTTGTTTATTATAATAGATAGTACTCAAGGTTAGAGCATATGAATAGATGCTGCATATGCCATCATGTATATGCATCAGTAAAACCCACATCAACATGTATACCTATCCTAGATCGATATTTCCATCCATCTTAAACTCGTAACTATGAAGATGTATACACACACATACAGTTCCAAAATTAATAAATACACCAGGTAGTTTGAAACAGTATTCTACTCGATCTAGAACGAATGAACGACCGCCCAACCACCACATCATCACAACCAAGCGAACAAAAGCATCTCTGTATATGCATCAGTAAAACCCGCATCAACATGTATACCTATCCTAGATCGATATTTCCATCCATCATCTTCAATTCGTAACTATGAATATGTATGGCACACACATACAGATCCAAAATTAATAAATCCACCAGGTAGTTTGAAACAGAATTCTACTCCGATCTAGAACGACCGCCCAACCAGACCACATCATCACAACCAAGACAAAAAAAGCATGAAAAGATGACCCGACAAACAAATTGCACGGCATATATTGAAATAAAGGAAAGGGCAAACCAAACCCTATGCAACGAAACAAAAAAATCATGAAATCGATCCCGTCTGCGGAACGGCTAGAGCCATCCCAGGATTCCCCCAAAGAAACACTGGCAAGTTAGCAATCAGAACGTGTCTGACGTACAGGTCGCATCCGTGTACGAACGCTAGCACGGATCTAACACAAACACGGATCTAACACAAACATGAACAGAAGTAGAACTACCGGGCCTAACCATGGACCGGAACGCCGATCTAGAGAAGGTAGAGGGGGGGACGAGCGGCGTACCTTGAAGCGGAGGTGCCGACGGGTGGATTTGGGGAGATCTGGTTGTGTGTGTGTGCGCTCCGAACAACACGAGGTTGGGGAAGAGGGTGTGGAGGTGTCTATTTATTACGGCGGGCGAGGAAGGCCGAAGGAGCGGTGGGAAAGAATCCCCGTAGCTGCCGGTGCCGTGAGAGGAGGAGGCCGCCTGCCGTGCCGGCTCACGTCTGCCGCTCCGCCACGCAATTTCTGGATGCCGACAGCGGAGCAAGTCCAACAGTGGAGCGGAACTCTCGAGAGGTCAGAGGCAGCGACAGAGATGCCGTGCCGTCTGCTTCGCTTGGCCCGACGCGACGCTGCTGGTTCGCTGGTTGGTGTCCGTTAGACTCGTCGACGGCGTTTAACAGGCTGGCATTATCTACTCGAAACAAGAAAAATGTTTCCTTAGTTTTTAATTTCTTAAAGGGTATTTGTTTAATTTTTAGTCACTTTATTTTATTCTATTTTATATCTAAATTATTAAATAAAAAACTAAAATAGAGTTTTAGTTTTCTTAATTTAGAGGCTAAAATAGAATAAAATAGATGTACTAAAAAATTAGTCTATAAAACCATTAACCTAAACCCTAAATGGATGTACTAATAAAATGGATGAAGTATTATATAGGTGAAGCTATTTGCAAAAAAGGAGAACACATGCACACTAAAAGATAAAACTGTAGAGTCCTGTTGTCAAAATACTCAATTGTCCTTTAGACCATGTCTAACTGTTCATTTATATGATTCTCTAAAACACTGATATTATTGTAGTACTATAGATTATATTATTCGTAGAGTAAAGTTTAAATATATGTATAAAGATAGATAAACTGCACTTCAAACAAGTGTGACAAAAAAATATGTGGTAATTTTTATAACTTAGACATGCAATGCTCATTATCTCAGAGAGGGGCGACCGGGTCACGCTGCACTGTAGGCATGCGTCGAGAGATAGATTTGTGAGAGAGACTGGTGATTTCAGCGTGTCCTCTCCAAATGAAATGAACTTCATATATAGAGGAAGGGTCTTGCGAAGGATAGTGGGATTGTGCGTCATCCTTACGTCAGTGGAGATATCACATCAATCCACTTGCTTTGAAGACGTGGTTGGAACGTCTTCTTTTTCACGATGCTCCTCGTGGGTGGGGGTCCATCTTTGGGACCACTGTCGGCAGAGCATCTTGAACGATAGCCTTTCCTTTATCGCAATGATGGCATTTGTAGGTGCCACCTTCCTTTCTACTGTCCTTTTGATGAAGTGACAGATAGCTGGGCAATGGAATCGAGGAGGTTTCCCGATATTACCCTTTGTTGAAAAGTCTCAATAGCCCTTTGGTCTTCTGAGACTGTATCTTTGATATTCTTGGAGTAGACGAGTGTCGTGCTCCACCATGTTCACATCAATCCACTTGCTTTGAAGACGTGGTTGGAACGTCTTCTTTTTCCACGATGCTCCTCGTGGGTGGGGTCCATCTTTGGGACCACTGTCGGCAGAGGCATCTTGAACGATAGCCTTTCCTTTATCGCAATGATGGCATTTGTAGGTGCCACCTTCCTTTTCTACTGTCCTTTTGATGAAGTGACAGATAGCTGGGCAATGGAATCGAGGAGGTTTCCCGATATTACCCTTTGTTGAAAAGTCTCAATAGCCCTTTGGTCTTCTGAGACTGTATCTTTGATATTCTTGGAGTAGACGAGTGTCGTGCTCCACCATGTTGGCAAGCTGCTCTAGCCAATACGCAAACCGCCTCTCCCGCGCGTTGGCCGATTCATTAATGCAGCTGGCACGACAGGTTTCCCGACTGGAAAGCGGGCAGTGAGCGCAACGCAATTAATGTGAGTTAGCTCACTCATTAGGCACCAGGCTTTACACTTTATGCTTCCGGCTCGTATGTTGTGTGGAATTGTGAGCGGATAACAATTTCACACAGGAAACAGCTATGACATGATTACAATTCGAGCTCAGTACCCTGGATTTTGGTTTTAGGAATTAGAAATTTTATTGATAGAAGTATTTTACAAATACAAATACATACTAAGTTGTACAAAAAACCAGCAACTCACTGCACTGCACTTCACTTCACTTCACTGTATGAATAAAGTCTGGTGTCTGGTTCCTGATCGATGACTGACTTCTCCACTTTGTGCAGAACAGATCTAGAGCTCTTAGTAGAGCGGGGAGATGTTGGTCGGCACGAGCATGATGTTCATGAGGTCGAACTGTGTGCCAGAGTTGAGGTCACGTTGATGTCGAGCGGCACGTCGGAGTTGGAGGAGGCCACCACGTTGCCGATGTTGATGTCGGAGAAGCGAGCACCGTTGTCGTTCACGCCGTCGTTGTTTGTGGTGGTGTTCACGTTTGTGGCGGTGTACACGGCCGTTGATGGTCACGCGGATGGTGGAGTTGCCGATGGAAGACACGCGGAGGTAGAGGTTGTAGGAGTTGCCGTTACCGCGGAGGTGTAACGAGCGGTGGTGTTGTTCTGCTCGAAGCGGAGAGAGTCGCCCTGGTTGCCGAACTTCTCGGAGATGAAGGTGCGGGTCTGGTTGTTCACCTGGGTAGCGTGGATCGGGGAGATGGTGAAGCCGGTGTAGTCGTTTGGGCGAGGTGGGTCATGGAGCCGTTCTCGTGCACAGCGTGGATGTTGTTCTTGCGGTTGTGCACGGACACCATGTAAGCGATGCCTCAGGGTGCCGGACGGGAGGCGATGTTGCGGATCTCGTTGTAGTGGAGTGGGCGACGGAGGTCCTCGTTGCGCACCACGAGCGGCACGCCGGAGATGTTGCGGATGAAGTAGTCCGGGAAGTAGTTGGAGTTGCCGCGTGCGGTGAAGGCACCGGAGCGGAGGCCGAGGTGGTCTCGAAGGACTCGGTCTGCCAGTTGGTCACGGTAGCCACGCCTCGCGGTCGGAGCCGGAGTCGAGCCAGGAGCGCACGAACGGTGTGAGGCGGAGGGAGGAAGGTAGAGCAGTTGAAGTTCTGGTTGAACGGGGAGCGCCGATGTCGCCGGAAGAGATGCCACCGGGTAGTTCACGCGAGCGGCGAGGAGGGCGTGGGTAGTTGTGGAGCCAGGCCCACGATGTTCGGGAAGGTGTTGGAGAGGCGGGCGCCGGAGAAGCCGTTGAGCACGTAGTTGGAGTTCACCTGGAAGGGAGTAGAGGAACAGCCAGTCCTGGGAGGTGAAGGACTGGGTCTGCTGCGGGCGGAGCCGGAGGCGTAGAGGTTGGCGCCGGAGGACACGAGGAGGGACTGGTACTTGAAGAGGGACCAGATGGACACGTACTCGAACACGTTGAGGAACATGTAGGTGCGGAACTCAAGCATCTCGTGGAGAGGGTGTTGAGCTTCTTGAAGGCTGTCTGGTAGGTGTTGATGCAGTAGTTGGAGTACTCCTTGGTGTAGTCCTTGAGGTAGCCACGGTAGGTGCGAGAGTGGCAGCGGAGATACCTCCTCTGCGTTGAGGATCACATCGCGGATGAAGGAGAGGTGGAGGTTGGCGGCCTGAGCGAAGAGTGGAAGGAGAAGGAGTTCATGTACGGAGCTGGAACTGGGTGAGGCGGTTGAGGAAGAGCTGCTGCATGGTGTTCACCGGGGATGTGATGGAAAGGAGAGTCGGGTTACGGTTCGGGTTGAGGAAGTTGTCCACCTGACGGTTGAACAACAACGTTCTTCTGGAGGCCCTCAAGTTCGGCGTTCACGCGGGAGAGGGTGTCAGCGTTGAGGCGCTGGTTGAGGAACTTCTCGGTCTCACGGAGGATGTCCTGCATGAGCTTGGTGTTGTCGTTTGGGAACACGAGGTCCCAGAGGCCATTGAGGATGCGCTTGGCAGCAGAAGCCTCCGAGCTTCTTGAGGAGGAAGATGGCCACAGTACCCACAGGAGCCACGTAGAGTGAGTGGTCGTCGCGCTTCCACTCGGTCCACTCCTTCTGGATGGTGTCGAGGACTTGTACTGGAAGGAGAACGGGTCCTGGGCCATCACGTTGTGGCCGTCGCAGATGGTGGTGCGTCCGGGTTGAGCACGGAAGCTCCTCCACCCTTCCGGGGCCTCGCACCTGATCGATGTGGTAGTCGGTCACGTCGGTCTTCAGGCCGATCTGGTTGCTGCTGGTGAACAGCTCGTTCACGGCCTTCTGAGCCCTCTCAGGTCGTACTCGGCCTCGAAGGTCACCTCGGCGGGCACGAACTCGATGCGGTCGATGTACACCTCGTTGCCGCTGTTGAACGTGGGCGCTCAGGGTGAACACGCTGCTGCGTTGCTGAAGTTGAAGGGGTGGTGAAGCCCGGTGCGGAAGCTGCCGCTCTGCAGGTTGCTGCCGCTGCTCATGGTGGCGCTGAAGTTGCCCTGGTTGATGGGGCGGCCGTCGATGCTGGTGTGGAACTGCAGGTTGGTGGTGCTGGCGTAGCGGATGCGGACGCGGTAGCGCTGGCTCAGGGGGCGGTGATGTTCACGCGCAGGGTGCTGATCTGGCCGGGGCTGGTGCGGCGCAGGATGTCGCCGCCGGTGAAGCCGGGGCCCTTCACCACGCTGGTGCCGCTGCCCAGGTTGGTGCTCTTGGTCAGGGATCTGGGTGATCTGGCTGCTGGGGATGATGTTGTTGAACTCGGCACTGCGGTGAATCCAGCTGAACATAGGTGCACGGATGATGCTCACGCTGCTGTTGCTGAAGCCACTGCGGAACATGCTCACGTGGCTCAGACGGTGGCTGAAGCCCTGTCGAGGTGGCGTTGTTGTTCTGAGGGGATCTCGTCCAGGCTGTCCACGGTGCCGCTCTTGCGGTACACGGCGCTGGGCAGGTTGCTGCTGTGCCGTAGGCGAACTCGGTGCCGTCCAGCACGCTCAGCTGCTGGTTGTTGATGCCGATGTTGAAAGGTCGACGGTACAGGGTGCTGCTCAGGGTGCGGTACACTCCTGGCCCAGCTGTGCCACGATGCGCTGCTGAGGTGCAGCGTTGCCCATGGTGCCGTACAGGGGAAGGTGAACTCGGGGCCGCTGAAGCCGACGGGGCTGGCCGCGATCTGGTGGCCGCTCCAGTAGTACTCGCCGCGGTGGGCGTCGGTGTAGATGGTGATGCTGTTCAGGATGTCCATCAGGTGGGGCTGCGGATGCTGCCCTCGATGCCCTAGGCGCTGCCGCGGAAGCTGCCGTCGAGAGTTCTCCAGCACGGGGTTGGTGTAAATCTCGCGGGTCAGCTGGCTCACGGTGCGGATGGGGTAGGTGCGGCTGTCGTAGTTGGGGAACAGGCTCACGATGTCCAGCACGGTCAGGGTCAGCTCGCGGCGGAACTGGTTGTACCTGATCCAGTCGCGGCTGTCGGGACCCCACGCGCTCCAGGCCGGTGTTGTACCAGCGCACGGCGTGGTCGGTGTAGTTGCCGATCAGGCGGGTCAGGTCGTTGCGGCTGTTGATGGTGGCGGCGTCGAAGCCCCAGCGCTGGCCGAACACGCTGACGTCGCGCAGCACGCTCAGGTGCAGGTTGGCGGCCTGCACGTACACGCTCAGCAGGGGCACCTGGTAGTTCTGCACGGCGAACAGGGGATGGCGGTGGTCAGGGCGCTGTTCATGTCGTTGAACTGGATGCGCATCTCCTCGCGCAGGGCGGGGTTGGTGGGGTCGGCCTCCCACTCGCGGAAGCTCTCGGCGTAGATTTGGTACAGGTTGCTCAGGCCCTCCAGGCGGCTGATGGCCTGGTTGCGGGCGAACTCCTCGATGCGCTGGTTGATCAGCTGCTCGATCTGCACCAGGAAGGCGTCCCACTGGCTGGGGCCGAAGATGCCCCAGATGATGTCCACCAGGCCCAGCACGAAGCCGGCGCCGGGCACGAACTCGCTCAGCAGGAACTGGGTCAGGCTCAGGCTGATGTCGATGGGGTGTAGCCGGTCTCGATGCGCTCGCCGCCCAGCACCTCCACCTCGGGGTTGCTCAGGCAGTTGTAGGATGCACTCGTTGATGTTGGGGTTGTTGTCCATTGTTGGATCCTCTAGAGTCGACCTGCAGAAGTAACACCAAACAACAGGGTGAGCATCGACAAAAGAAACAGTACCAAGCAAATAAATAGCGTATGAAGGCAGGGCTAAAAAAACATATAGCTGCTGCATGTGCCATCATCCAAGTATATCAAGATCAAAATAATTATAAAACATACTTGTTTATTATAATAGATAGGTACTCAAGGTTAGAGCATATGAATAGATGCTGCATATGCCATCATGTATATGCATCAGTAAAACCCATCAACATGTATACCTATCCTAGATCGATATTTCCATCCATCTTAAACTCGTAACTATGAAGATGTATGACACACACATACAGTTCCAAAATTAATAAATACACCAGGTAGTTTGAAACAGTATTCTACTCCGATCTAGAACGAATGAACGACCGCCCAACCACACCACATCATCACAACCAAGCGAACAAAAGCATCTCTGTATATGCATCAGTAAAACCCGCATCAACATGTATACCTATCCTAGATCGATATTTCCATCCATCATCTTCAATTCGTAACTATGAATATGTATGGCACACACATACAGATCCAAAATTAATAAATCCACCAGGTAGTTTGAAACAGAATTCTACTCCGATCTAGAACGACCGCCCAACCAGACCACATCATCACAACCAAGACAAAAAAGCATGAAAAGATGACCTGACAAACAAGTGCACGGCATATATTGAAATAAAGGAAAAGGGCAAACCAAACCCTATGCAACGAAACAAAAAAATCATGAAATCGATCCCGTCTGCGGAACAGCTGAAACCATCCCAGGATTCCCCAAAGAGAACACTGGCAAGTTAGCAATCAGAACGTGTCTGACGTACAGGTCGCATCCGTGTACGAACGCTAGCAGCACGGATCTAACACAAACACGGATCTAACACAAACATGAACAGAAGTAGAACTACCGGGCCTAACCATGGACCGGAACGCCGATCTAGAGAAGGTAGAGAGGGGGGAGGAGCGGCGCCTTGAAGCGGAGGTGCCGACGGGTGGATTTGGGGAGATCTGGTTGTGTGTGTGTGCGCTCGAACAACACGAGGTTGGGGAAGAGGGTGTGGAGGGGTGTCTATTTATTACGGCGGGCGAGGAAGGGAAAGCGAAGGAGCGGTGGGAAGAATCCCCGTAGCTGCCGGTGCCGTGAGGAGGAGGAGGAGGCCGCCTGCCGTGCCGGCTCACGTCTGCCGCTCCGCCACGCAATTTCTGGATGCCGACAGCGGAGCAAGTCCAACGGTGGAGCGGAACTCTCGAGAGGGGTCCAGAGGCAGCGACAGAGATGCCGTGCCGTCTGCTTCGCTTGGCCCGACGCGACGCTGCTGGTTCGCTGGTTGGTGTCCGTTAGACTCGTCGACGGCGTTTAACAGGCTGGCATTATCTACTCGAAACAAGAAAAATGTTTCCTTAGTTTTTAATTTCTTAAAGGGTATTTGTTTAATTTTTAGTCACTTTATTTTATTCTATTTTATATCTAAATTATTAAATAAAAAACTAAAATAGAGTTTTAGTTTTCTTAATTTAGAGTAAAATAGAATAAAATAGATATACTAAAAAATTAGTCTATAAAACCATTAACCCTAAACCCTAAATGGATGTACTAATAAAATGGATGAAGTATTATATAGGTGAAGCTATTTGCAAAAAAAAAGGAGAACACATGCACACTAAAAGATAAAACTGTAGAGTCCTGTTGTCAAAATACTCAATTGTCCTTTAGACCATGTCTTTGTTCATTTATATGATTCTAAAACAAATATATTGTAGTACTATAGATTATATTATTCGTAGAGTAAAGTTTAAATATATGTATAAAGATAGATAAACTGCACTTCAAACAAGTGTGACAAAAAAATATGTGGTAATTTTTATAACTTAGACATGCAATGCTCATTATCTCTAGAGAGGCACGACCGGGTCACGCTGCACTGCAGGCATGCAAGCTTGGCACTGGCCGTCGTTTTACAACGTCGTGACTGGGAAAACCTGGCGTTACCTTAATCGCCTTGCAGCACATCCCCTTTCGCCAGCTGGCGTAATAGCGAAGGCCCGCACCGATCGCCCTTCCCAACAGTTGCGCAGCCTGAATGGCGAATGCTAGAGCAGCGAGCTTGGATCAGATTGTCGTTTCCCTTCGTTTTTAACTATCGGTCGTTGGCGTCGGGCGTGTGGTTGAGCTTCGAGCCGTGAACTTGTCTTCCGAGCACCGACGTCATCGCGATGACCTCCATCTGGGTCGCGGCGGTGGGTTCTTCATGACTTGGTCGATGGCAGTCCCGAGTCACAGCGTCGGCGTCGGACCCCGGCTCGATGGCGTCATGGCGGTTGACGAGCCTCGTGCCCGACCCATTGTTCGGCGGCCGGAAGTGGGCGTAGCACATAGCGTGGAAGTGCTTCGAGGCGAGATGAAGAAGCGGCTGCCTGCGGCTGTCTTCATCGGCGACCGTCGAAGAACATGGTCGAGTTCCGTTGGATCGGGCCGGGGAGGCCAGGTGTCGACCCATTCGATACCAAGTCCGCGAACACAGGACTTGATGGCCTTAGAAGCGAGCGAGATCGCCGCCCATGATTTCCACCGCTGCGATTCTACGGGCCTCGGCACTAGATGATCTCCCGGAAGGATGACACCTGATTCACCGGATGAGACTCGAGTAGTGTCGCAACTTCGCCGCGTCGGGATCACCGCGTCTGGCAGACTTACGAATTTGTGGGTAGCGGATCTTGGTCTCGGACGATACCTGTGATGAAGTAGACTTGCCTCGGGCCGGCAATGCATGCCCCTCTCTCGTCTCTCAACCACGATCGCGGCTTACTTTGAGTGGTCGCGTAGATCAATAGAGCTTCTCCGTGAGGGGCACCAAGATAGGCGTTCGTGAGGCGCCTTGAGTTCGAGGGCTTCCTCGGCCTCGGGGTCCAAGTGAAGCACGGCCTTCCTTAAGAGGCGGTATAGAGCGAGCCTCTTCGGCCCGGCGTGAATGAAGCGGCTCAGGCCGCAAGACATCCGTGACTCTCTGTCGCCCTTCAAGTCCTTGATGGGCCCCATCTTGTGATGGCGCGATCTTCTCGGATTGGCCTCGATGCCACTCGGAGACAATGAACCCCAAGAGCATGCCTCGAGGAACCCCAAAGACACACTTCTCGGATTAAGCTTCCGTCTTTCGCCTTGAGACATCAGAATGTCACTTCAAGGTCGGAAAGGAGGTCTGAGGCTTTCCTCGTCTTGACTCGATGTCATCGGCGTAGGCCTCGGCCGTCGGCCAATGTGTTCGCCGAACACATGGTTCATCTGCACCGGTGCGTCGCACGCATTCCTCAAGCCGAGCGGTATGGTGACATAGCGATACATGCAGAAGGGTGTGATGAAAGAAGTCGCGGTGTCCGGATTCTTTCATCTGATTTGAAGGTACCAGTAGGCATCGAGGAAAGACAAGGTTTCGCACCCAGCGGTGGAATCCACAATTTGATCGATGCGAGGCGGTAGGGAACCCGGACATGCTTTGTTTAGACAGTGTAGTCTACACACATCAGCCCTTTCCCTCCTTTCTTTCTCAAGAAGCGAGGTTGGCAAGCCATTCGGGATGGAATACCTCTTTGATGAACCTGCTGCCATTAGCTTGTGGATCTCCGCCTATGGCTCTGCGCTTTTCTTCATCGATCTTGCCCGAGGTGCGCTTCGCAGTCGGGCTCCAACTCGGATATCGGCGAGTGCTCGGCGACATCCTCGGTATCACGGGCATGTCAGGGACTCCACGTCGGCGTTCGCGCGGAGAAATCGGCGAACCTGCACTTCCTATTTGGGATCGAGCTCGGGCCGATCCGGATCGCTTGGGCGTCGGCTGGCTGGGTCGAGGGCGGACTTAGTCTCGTGGCTCGAGTTGTCGGCGTGGCGCTTCACGTCTGGCACCTCCTTAGAGGCTTTCGGAGTCGGCGATGAGGGCCTTGGATTGGCGAGGGCCTCGGCGTACTCACGCACTCCACGTCGCATTGACGTGTTTGTGCGTGGGTGACGATGACTTGGGTGGGGCGGCATCTTGAGCTTGAGGTAGGTGTAGTTGGGGCGGCCATGAACCGGTAGCATGGCCTCCCGATCTTTGCGTGGTAGGTTCCTCGAACCCGACCACCTCGAGCGTGAGGGTCTCCCCGAAGTTGGAGGGTGTTCGGCGGCGAGGAAGGTCGAGTTGTCAGAAGTGGGGCCAGCTTCGGGGATGATCGTGGAATGGCGCGGCGCTGCGGATAGAGGATAGATCAACAGCGGGGGCAGGGGTCTCGGCATAGATGATGTTGAGGTGTTGCCTCAGTCCGCGAGGACCTTGGTGAGCTGGCGTCACGGATGAGCGGGGTCGACAGCGGCGGGTATTTCGGGCTCGACGCGTGGTCGGGGTGGTCAGCTTAATCGAAGGTGATGGGCTTGTCGGACCAGTCTAGGTAGTGAGTAGCCTAGAGGGGTGGAAAGGCTAATCTGAAAATTTTCACAACAAACTGAAGATTGTTATATACAGTTCGGCCGGTAAGCAGGTTCAGCACTACGTCATAAGTTGAACCACTCAGAACCAATCCATTGTTTATAAACTTTAGACTAAAATCTACTCGAAAAGTATTTACTTGAGTTCTTGAGAGAAGTAAGATATAGCTAAGTGAGGATGAAAAGATGAATATGTAAAGGCTATTTTACTTCTAGATAAACTCTACGGAGAAATCTTTATGTCAATCTTGCAAGTAAAAATATCTCAAGTTGAAACAAGTAAGAACACAAGACACAAGATTTAATCAGAGGTTGGCCACACCACAAGGTGTCCTCTAGTTGAGAGCCCACAAAGGGTAGGGTCTTTTCAGCCTAATCCTCCAAAGCCGACCACAAAGGTCAAGGCAATCTCTTCTTATCTTAGCTCAAGAAGCGGGTGATACCAACTTCTTAGGGTCGTCCACAAATTTGGAGACTCCCAAGTAACCTCGAAGATCTTGAAACCTAGGGTTTCAAGAACACCAAACACACAAAGAGGGGTTTGCACAAGCTCAAGTCTTTGAAAAAGAGATGGGAGAGGAAAACCAAATCGTGAGCACAAGCACAAACCTCACACCCAGAGCTCCTCCAACAAGGTTGAATCTTGAGGAAGATTTAAGTGTGAGAGAGATGGAGAGATGAGTGCTTTGTCTCAAGTTAGGTGAGCAATAAATGAGTGAGTGTTGGTGTCTTGAAGAAGAGAGAGGGGTCTATTTATAGTCACGGCTCAAAACTAGCCGTTGACCAAAACCGCTGGCGGTTGAAGCGCGCCCCTAAGGCGGTTGAACGCCAATAGTCGCCGGTGTCTGCAGTCGGTCCGGGGCCAGGCTATGACCCGACTGCGGCAGTCAGACGAGCGGGATCGGTTGAGCAGCCCCCTAGGCGGTTGAGCAGCCCGACACCCAGCGACACTTGCCCGATCGGTGTGCAGCCAGATCGAGGTCGGAGGGTCGAGAGACGCTTGAGCTACCGAGACGGTTGGTGGTCTAGACCAGAAGTTTGGAGAAAACCCTGCTCACCGCCACAGGGCAAGTTCAATGTTGGTATGGTCGAGAGGTCCACGGTGAAGTTGAAGTTGTTGGAGTTGAAAGTTCAACTCATTTGAAGTCGGCTAAATTTGAAAAGCTGTTCGCAATTTGAGAAGCTCATCTGGCGAAGTTAAGTTAAGTTGGAAAGCTCATCTTGAGTTGAAGAATTGTTTTTTAAGAAGAACACTCTAGGTTTCTCAACCCAAACCATGGTCGACCAAATAATGTTAAAGAGATTTTTGGTTTTCAAAAATAGCTTTTGAATTAGAGGACTTGAGCTATAGCAAACACATGCACATGCGAGGAAAAGAACAAGGAAGAATTACATCATGCAACACAAGATTTTACATAAATTTTATCGTCTGTTGCATGAAGTCCTTGGTGCTTCCTTAAGTTCTGTTTCCTTCTAATCAAACAGAGAACAAAATTGTTAGTACTCTTATTTGTTTTGTCATTAAATCACCAAAACCCTCACTTGGGGTTGATTGCACTTACAATCTCCCCCTTTTTGATTGATGCCAAACAATTAAGGTCAAAAGATATATATTTGCAATAGAAAATTTCTTTTGAATGATTGTATGTAGATGGCTCCCTAAATGTGTATACGATTGTGAATCAACGTCTTGACATAATATGTCATGTGAGCAACACATTTAGAGATAGTGATAACCATACTAAATACAATTATCCGAGGGTGCAAGAGTGTCATGACAGAGTTGTGATGTATGACTATCTCTAAAAACCATTATTTTTACTTCATAGCGAGTAGACATTACAAGCGATAGGACAAAAGATGATGGCGAAAGACCATCATATTACTTTGAATAACAATCCAACAGTTTTATTTCATACAATGGTAAAGGGGTACAAGCCACAACGGTGGCCAAAACAAAAATAATCCAAAAGAAAAGCGAAGAACCACAAACTAGAAGTTGGATTTTCTCCCCTTTGGCAACAAGTACCAAAAGCGGAGAGAGACAAGGATACAGAATCAATATCCTCCAGGAGGAGGATATGACGGAGGATATGAAGTGTAGTCGGGAAAAAGTCCTCATCAGCGTCCTCCTCAAAAGTGGAGTCAGGGTGGCTCATCGTATGAAAAAGCAGATCAAGAAGATGTGATGATCGGGAGGAGGTGGAGGTGGTGGTGGTAACGAGCCGGATGGCCTAGGTCACCAGAATGGGTCCGGACCCGGGTAAGGAGGCGGAGGTGGGAGGATGATTGACCGGATCCCCATGGTAGGGAGGCGGGGGCAAACTCCTGCGATCATCAACATAGACTTCTTCATCTTCATCGTCTACGACATAAAGGCACCAGTGAGGCTGGGTGCCACTCATTGATCTCCGGGGGAGGAGGATGGAGAGGCATCAGGCGAGCGAGGGACAAGGCATGCCCATGGAGGCTTGGCGGCGAATGTCGTCGATCTCCGTGGCGATCGTGCCACCTCATGGACATCGAAATGTTCGTCACATGGAGAAAAATCACGCAAACCCATGGACCAAGCGAGCACCCATCCCACGCGACCTCGACCATGACCACGAGCACGTGGCATGGGAGATCCATGACGAGAGAGGGAGCGAGCGGCGATGAGGAGAGGAGGATGGATGATGGGGAGTGTGGGGTGGCTCAGGTGGAGGAGGCGGGGCCTCGGGCGTGAGGCAGCCGTGCGGCGGCTGGGTTGGAGGAATCAACCCGGTAGAGTGCGTAAGCGGGAGTGTTTGGGAGTTTTTGAAGTGGGTGCAGGTGACCACCGATCATCTTCATGATAAAGGTGCATGAAGACACCCTCGAGAGGAGACGAGAAGCAGGCGATCTCTTCCTGGATCATGTCAAACACATTGATGCGGCGGAACTTTTGGAGCAAGGAGGAGGAGGAAGACTCGACTCTCCCAAGGGCGTTTATTTGATTCCGCCCTTCGGAGCGGTCATCGGGACAAAGAATTAAGGTACCATGGTACCTGCATGTTATTGTTGTCGAACTCACTCGATAGAACATGAATGAATCTGTCTCTTCATGCAAGCGTCGAATGTCATGCACTCGCATGTTCTTTATACGAATATCTCATCGAGAAGCCTGAATATGGGCAAAGCGACGATATGACTTTGTGCGATTACCACGGATGAAGAAATACATAGTACGGGTAATCATACTCATCGGCTTCCTCATCTACTTTCTTTATCGGAGGGTGGCATAGGCGAGCTACCACCTCATCGTTCGGGGTATTCCATGGTCATAATGTTCATGACCCTTTCTCTCTAAGTTTACGGTGGCGACATGTCGGCATCCCCATATTCTCACAACCCTCCATCAATAAATCTATGTCTGGACGGCTGGGTGTTTCTTGGAAAGAATGACAGAATTGTAGAAGTCGGCTGAAAAGATTCCAGGCGAGGTCCCGACACAGGATGACGAGGAGTGAGAGTAGGGTTGGTGAGAACCTCGGTGAAGGTGTCTATTCCCAGTGAAGTCGGCGGGAGATTCATGCGGCCGCGGCGGCGTTCGCCGGATGCATGTGCGATCCGGTGGCGTGAGAGCATCGGCGCGGAAGGTCAGCGAAGTCGTCGAACGCCCATCCTCCTCGACAGGAGCATACGAAGGAGGCGTGCTGGCGAGGATGAGGCGCCAACCTTTTGGCGTCTCCGCGAAGTCGGTGCGAGGGCATGGAAGAGCCTCACCATCCATCAGACGAGGCCACGTCCTCTCTTGAGAGGGGCGGCGAGGCGGAGGAGAGGGGATGAATTCCTCCCGAATCTCGCCTGAGTCATTTGCTAAACTCAATCACAGAGGGTTGCGACGACGCACCATGATTGAAAAAGGTGGTGAAGCTGAAGGTTGGGGTGTGTGAAAGAGTGGCTCTAAGGTTGTGAAGCGACTAAGGCAAGAGTGAATGAAGTGGGAGAAGAAGGGAGAGTCAAGTATATAGCGGTTGGTGGTCTCGAGACCGGTTCAACGCCCCATGGGCTGGATTCATTTGGTTCTAACGGTGTGGTCGAAGAACGAAGCAATGGAGGAAGATGCCAAAATCAAATTTACGGCAGGATTACGAAAAATATCTTGCGAGATTCTATGAAAAAAATCAAAACCAAGTGTTTGCGCGATGAGACCAGGACGGCAAAAGAAAAAACATAAAGAAAGGATTCTGCGGCGCCTGTATCCCTCACGCTGCGCACAGGTGACAAAAAATATTTCCCTTCGCTGCGGGTGACAGGTGATCGGGTCACAAGAAAAGCTAAGGCTACCCAGCGATAGTGTGGGGCTAAAAAAGCTGTCTTGCGCAATTTCTGGAAGCGGCCCAGACGGGGAAAAATTAGGCGCCCACGACATGCATGGTGTGAGCGAGGAGAGAAAGAATCTGCGCAGCATGGTCTTGTGAAATGGTAAGCGATCAAAAAAATGCTGCGCGCCAGATGAGGAAAGTGACCGGACGAACAAATAAAAAATTCACGCATCACGGCAGCTGCTTCTGGGACACCACAAAAAATGCCGCGCGTGGCTAGAACACGTGGATCTGCATGGCGCCACGACATTTGCATGCAGCTAAGACAGGAGCCCTAAAAAAGTTGGCGCTGGCGACAAGCGGCCGGACGAAAAAAATGTGCGTGATTCTTTGGCGTGACAAAAAAGAAAAAAAAATAAATCTGCAGCAGAGGAAGAGGAAGTACCGGACAGGAAAGAGAAAAAAACCACGCCCAGCCAAGAGAAGAGCGGCCGTAAAAATATTATTGCGGCCCGAAAAAAGTCCACGGTAGTGAACTGCGGCCGGTAAAAAAATTCACGCCAATACGGCAAAGTGGAAGCGACCGGTCAATAGAAAAGATCTTTGCCGCGCCAGAGATGTGGCCGGTTAAAAAAATCTCGCGTCGCAACTTGCGGTGGCGACTGCGTGAAACAGAGCACTTGCGGTCGAGGCAGGCGACCGTTCAGCCGGTCCACCATGTCGGTTCAACCGGTTTAACTAGGATGGTCTGGTAAAAAGCGGTTACTTCGAGGTGAAGTTCGGCTCTAAGTTCATTTTCCTCCAAATGAACTAGAAAGTTCAGTGGTCAAGCCAGTGAGCCAAAAAGAATCTTAGCACTAAAATGCATTAATTTTAACATTTTCACCAAGTTTTTGAAAGATCATCACATAGCTTTAAACTATCAATCAAAATAATTGAATCAAACAATTTTGTGACCAGTTTTCAAGTTAAGTTCAAGAATCCAAGATATTTAATTCACTCTTAAGAAAACAAAACTAGTGCGATCGAGGGGTTTTGTGAAGATATCGACTAGTTGATTTTGATTGCTCACATGAAACAATCGATATCTCCTTTGGCCGTGGTCTCTCAAGAAATGGTGTGCGATGTCAATATGTTTAGTTCTAGAGTGTTGCAGGGTTGTTTGCAAGTTTTATGCACTCTCATTGTCACACAAAGTGGAATTTTGTTAAACTCACAACCAAAATCTCTAAGGGTTTACTTCATCCATAACAAGCTGCACAACATGCCCCATAATACTGTCAACCTGCAGTGGAAAGTGCAACACAATTTTGTTTCTTGGAACTCCATAATACTAAGGATCATGAGGTGTAAGTCGAGAGTGCTTTTAGATCTACTTTGCAAGCATAATCTAGTCAGAATAGCCAAGTAAATCGAAAGGGAGCCTTTGGGATACCATAATCCTAGGTTTTGGGTGTGAACTAAGTATCTTAGAATTCTCTTAAGCGCTACAAGATGGCAATCTTTAGGATTTGCTTGCGTGCACACATGCAAACACTCAACATAATATCGGTCTAGATGCACATAAGTAAACTAGTGATCCTATCATTGATCTATATAATGTTTGATCTACTGTTTACCTTCCTCATTTAGTAGAGATGTCCATTTGATGACATTGAGTCTTGGCGTGTTTTGCCTTTTCCATGCCAAATTTCTTGAGCATATCTTGTGTATATTTGGTTTGGCATAGAAAAGTACCTTCCTTGAATTATTTTGACTTGAAATCCCGAGAAGTATTTAAGCTCGCCCATCATAGACATCTCAAACACATTAGTCATTACTTTGCTAAACTCTTCACAAAATTTTTCATTAGTACTACCAAATATAATGTCATCAACATATACTTGCACACAAATAATTCATTGTCAACTTTTCTAGTAAATAAGGTAGAGTCGGCTTTTCCTATTGTAAACCCATTCTTAATTAAAAATTCTTTAAAACAGTCATACCAAGCTCTAGGGGCTTGTTTAAGCCCGTAAGTGCCTTGTGAAGTAGATAAACATGATTTGGCTTCTTTGGATCTTCAAAACCGAGGTTGCTCCACATATCTCTCTCTTGTAGTGGTCCATTTAGAAATGTGCTCTTGACATCCATTTGGTATAGCTTGAAATCATGGTTAGTAGCATATGCAATTAATATTCTAATTGATTCTAACCTTACTCTGCAGCGCATATGTTTAGCCAAATCAAGTCCTTCCCTTGAGTATAGCCTTGGGCAATAGCAGAGTGCCTTGTTTCTTGTAACCGCACCATGTTCATCTTGTTTGTTCCTAAAGACCCATTTAGTCCCAATCACATTTTGTTTGGGTCTTTGGACTAAGGACAGACTTCATTCAGGGTGAAGTTGTTTAACTCTCTTGCATGGCAATTATCCAATCCGGATCACCCAATGCTTCTTCAACCTTAAGTGGCTCAAGAGAGGAAACAAGAGTAAAATTCACAAAAATAGCTAAGCGAGATCAGTCGTTACCCCTCTCTGATGCTACCCGGGATGTTGTCCCTTAGGATGATCCCTTTGAATTGTATGATGGACTCTTGGATGAGGCGCGATGGTTGTCTTTGTATTGCTCCTCCTCATCATTCACTTGATCAAGAGGTACTTCACCATCAATGCTTTCGTGTTCATCTTCTACCATTTGGCATCCTTTGATGATTTTCTTCATGGCTTGGATGACTTGAGGTTGATGGGTTTGCTTGGGTGGAGACTTTGTCCTCACCACCTTTGCACCCACATCAACAACCTCATTGGTCATCCAGTTCCTTCCTCATCATCCTTTTCTTGAGGTCTCACTTCACCTATTGCAGGATTTCTTTGTGGCTACAAGGTAGTTCTTCATTTCCTAGGTGGACATTAGAAACATGCCCTTATGAAACCATTAGACTCATCAAATGTCACGTCTATCGCTATTTCAACAAGGCCGTGGTATTGTTGAAAACACGATATCCATGCGCATTTGATGCATAACCAAGCAAGAAGCCCTCGTCCACTCTAGGAGCAAACTTTGAGCTCTTGACTTTCTTGTTAAGAATAAAACATTTACAACCAAATACTCTAAAATAATCAACTTTAGGTTTGTTACGGTGAGAAGCTCATAAGGTCTTTTTGTAGATCTTATGAAGATAGAGACGGTTGATTGCATGATAGGCGGTGTTGAGCCTACGCTAAAAAGTTGTCGGGTGTCTTGTACTCATCCAACATGGTTCTTGCACTTCAATTAGAGTTCGGTTCTTTCTCCACAACACCATTTTGTTGTGGGTGTAAGGCACCGAGAACTCATGCTTGATTCCCTCTTCTCCTAATTCTTCGACACTGTGTTCTTGAATTCCGTCCCATTATCACTTCTCACTTTCTTGATTTTGAGCTCAAATTCATTTTGAGCTCTCCTCATAATTTCTTCAATATTTCTTGAGTTTCACCTTTATCACTAAAAAGCCGAACCTGTGAATCGAGAAAAATCATCAACAATGACTAAACCATACTTACTACCAATTAATGCTAATGTAGGCCCACGGGTCCAAAAGAGGTCCATGTGAAGAAGCTCAATGGCCTCTTTGTTTGTGACCATTCTTTGATTGATGTGGGACTCATGTTTAACTCTTGCTTGGCATGTACAAACCCTATCTTTCTCAAATACAACATTTGTT

>nano4

AATGTTGTATTTGAGAAAGATAGGGTTTGTAGGCGCATGCCAAGCGGGAAACATGGAGTCCCACATCAATCAAAGTGTGGTCACAACAAAGAGGCCATTGGAGCTTCTTCACATGGACCTCTTTGGACTGTATTCCCATTAGCATTAATTGGTAGTAAGTATGGTTTAGTCATTGTTGATGATTTTTCTCGATTCACACAGGTTTTTCTTTTTAGTGATAAAGGTGAAACTCAAGAAATATTGAAGAAATTCATGAGGAGAGCTCAAAATGGTTTGAGCTCAAAATCAAAAAGTGAGAAGTGATAATGGGACCGAATTCAAGAACACAGTGTCGAAGAATTCTTGGAGAAGAGGGAATCAAGCATGAGTTCTCGGTGCCTTACACTCCACAACAAAATGGTGTTGTGGAAAGAAAGAACGAACTCTAATTGAAGTGCAAGAACCATGTTGGATGAGTACAAGACACGACAACTTTAGGCGGAGGCGGTCTCAACACCGCCTATCATGCAATCAACGTCTCTATCTTCATAAGATCTACAAAAAGATACCTTATGAGCTTCTCACTGGTAACAAACCTAAAGTTGATTATTTTAGAGTATTTGGTTGTAAATGTTTTATTCTTAACAAGAAAGTCAAGAGCTCAAAGTTTGCTCCTAGAGTGGACGAGGGCTTCTTGCTTGGTTATGCATCAAATGCGCATGGATATCGTGTTTTCAACAATACCACCGGTCTTGTTGAAATAGCGATAGACGTGACATTTGATGAGTCTAATGGTCATAAGGGCATGTTTCTAATGTCAACATGGGAAATGAAGAACTACCTTGGAAGCCATAAAGAAACTGCAATAGGTGAAGTGAGACCTCAAGAAAAGGATGATGAGGAAGGAACTTTCTGGATGACCAATGAGGTTGTTGATGTGGGTGCAAAGGTGGTGAGTGACAAAGTCTCCACCCAAGCAAACCCATCAACCTCAAGTCATCCAAGCCATGAAGAAAATCATCAAAGGATGCCAACAGTGGTAGAAGATGAACACGAAAGCATTGATGGTGAAGTACCTCTTGATCAAGTGAATGATGAGGAGGAGCAAATACAAAGACAACCATCGCTGCCTCATCCAAGAGTCCATCATACAATTCAAAGGGATCATCCGTGGACAACATCCCGGGTAGCATCAGGAGGGGTAACGACTCATCTCGTTTAGCTAATTTTTGTGAATTTTACTCGCTTGTTTCCTCTCTTGAGCCACTTAAGGTTGAAGAAGCATTGGGTGATCCGGATTGGATAATTGCCATGCAAGAGAGTTAAACAACTTCAGAATGAAGTGCTGTCCTTAGTCCAAAGACCAAACAAAATGTGATTGGGACTAAATGGGTCTTTAGGAACAAACAAGATGAACATGGTGCGATTACAAGAAACAAGGCACTGTTGTTTGCCCAAGGCTATACTCAAGTGGACTTGATTTTGGCGAAACATATGCGCTGCGCAAGGTTAGAATCAATTAGAATATTAATTGCATATGCTACTAACCATGATTTCAAGCTATACCAAATGGATGTCAAGAGCACATTTCTAAATGGACCACTACAAGAGAGAGTATAATGGAGCAACCTCCGGTTTTGAAGATCCAAAGAAGCCAAATCATGTTTATCTACTTCACAAGGCACTCACGGGCTTAAACAAGCCCAGAGAGCTGGTAATTGTCTTAAAGAATTTTTAATTAAGAATGGGTTTACAATAGGAAAAGCCGACTCTACCTTATTTACTAGAAAAGTTGACAATGAATTATTTGTGTGCCAAGTATATGTTGATGACATTATATTTGGTAGTACTAATGAAAAATTTTGTGAAGAGTTTAGCAAAGTAATGATGAGCGTGTTTGAGATGTCTATGATGATGAGCTTAAATACTTCCGGGATTTCAAGTCAAAACACTTCAAGGAAGGTACTTTTCTATGCCAAACCAAATATACACAAGATATGCTCAAGAAATTTGGCATGGAAAAGGCAAACACGCCAAGACTCAATGTCATCAAATGGACATCTCTACTAAATGAGGAAGGTAAACAGTAGATCAAACATTATATAGATCAATGATAGGATCACTAGTTTACTTATGTGCATCTAGACACGATATTATGTTGAGTGTTTGCATGTGTGCACGTTTTCAAGCAAATCCTAAAGATTGCCATCTTGTAGTGCTTAAGAGAATTCTAAGATACTTAGTTCACACCCAAAACCTAGGATTATGGTATCCCAAAGGCTCCTTTCGATTTACTTGGCTATTCTCCAGTTATGCGGTTGCAAAGTAGATCGAAAAAGCACTCTGGGACACCAATTCCTGGCGATCCTTAGTATTATGGAGTTCCAAGAAACAAAATTGTGTTGCACTTTCCACTGCAAGTACATACTTGGTGGGGCATGTTGTGCAGTTGTTATGGATGAAGTAAACCCTTAGAGATTTTGGTTGTGAGTTTAACAAAATTCCACTTTGTGTGACAATGAGAGTGCCATAAAACTTGCAAACAACCACTGCAACACTCTAGAACTAAACATATTGACATCGGACACCATTTCTTGAGACCACGAAGAAAGGAGATATCGAATTGTTTCATGTGAGCACCGAAAATCAACTGGTCGATATCTTCACAAAACCCTCGATCGCACTAGGTTTGTTTTCTTAGAGTGAATTAAATATCTTGGATTCTCGTAACTTAACTTGAAAACGGTCACAAAATTGTTTGATTCACTATTTTTGATTGATAGTTTAAAGCTATGTGATGATCTTTCAAAAACTTGGTGAAAATGTTAAAATTCAATGCATTTAGTGCTAAGATTCTTTTTGGGCTCAACGGCTTGACCGGTGAACTTTCTAGTTCACCGGAGGAGGCCAAATGAACTTAGAGCCGAACTTCAGCTCGGTGTTTTAACCGATTTTACGGACCATCCTAGTTAAAAGGTTGAACCGACATGGTGGACCCGGTTGAACGGTCTGCCCGGCTCGACCAGTTTGTTCACCAGTCATCAGTCGCAATGATGTCGACGTCGCGAGATTTTTATAGGCCACATCCTGGCAGGCGCTGACATCTTTCTCTATTGACCGTCGCTTCCACTTTTCTTGACTTTGGTGAAATTTTACGGCCGCAGTGCATCAATTTGACCTTTTCGATCGCGCAACAATATTTTTACGGTCGCTTCCTCTCTTTGGTGGCTGGCGTGCAGATTTTTTTCTCTCTTTCCTGTCCGGTCACTTCCCTCTGTCTGGCGCTTGTATTTTTTTTTTCGCTTCTTTGCCGGCTCGTGGTCTGCATTGCCACTTTCTCGTCCGCCGCTTGTGTGGCGCGCGAACTTTTAGGGCTGTACGGTCTTAGCTTTGCATGCATGTGTGGCGCCATGCGATATCCGGCGTGTTCTAGCGTCAATGCGCCGCATGTTTTTGTGGTGTCCTAGCAGTGCTGCGCATCACGAATTTTTTATTTGTTCTGTCCGGTCACTTCTCCTCGGCGCGGCATTTTGGCGTTTAACCATTGCGCACCATCACTGCGAATTCTTTCTCTCCTGCTCACACCATGCATGCGTGTCGAGCGAATTTTTCTTTCTGTCTGGTCGCTTCCAGCGATGCGCGCCAGATTTTAGCCCCACACTATCGCGTGTCGCTAGCCTTTGCTCTTGTGACCCGATCGGTGTCACCAGCGCGACAAAGAAATATTTTTGTCACTTCCGTGCAGCGATGTGAGGATACAGGCGCCGAGAACTTTCTTTTGCGCAGTTCTTTTCTTTTGCCGTCCGGTCTCATCTGCATAAACACTTGGTTTTGATTTTTTTTCATAGAATCTCCGCAGTACTTTTGAATCTGCGTCGAAATTTTGGCATCTTCCTGCTTTGTTGGTTCTCTGACCCACCTTTGGTTAGAACCATGGCCCAGCCATGGGGCGATTGAGTGACCGGCTCAGCTACCTATATATGACTCTCCCTTCTCTTCTCACTTCATTCACTCTTGCCTTAGTCGCTTCACAACCTTTGAGCCACTCTTTCACACACCTCAACCGTTCACGGTTCACCACCTTTTTCAATCATGGTGCGTGTCGCAACCCCTCGTGATTGAGTTTAGCAGCGACTCAGGCGAGATTCGGGAGGTCTCCATCCCTCCTCCTCCGTCGCTCCCTCTCTAAGAGGACGGGCTGGTCTGATGGATGGTGAGGCTCTTGCCCTCGCCGCCGACTTCGCGGAGACGCAAAAGGTTGGCGCCTCATCCTCGTCGTAGCACGCCTTCCTTCGGTATGCTCCTGTCGGAGGAGGATGGGCGTTCGACGACTTCGCTGACCTTCGCGCTGATGCTCTCTACGTCACCGATGCACATGCATCCGGCGAACGTCGGCAGTCCGCATGAATCTCCCGTCGACTTCACTCGAAGGAATAGACACCTTCAGCGCGAGGTTCACCAACCCTACTCTCACTCTCGTCATCTGGGTCCAGGACCCTCCCGGTTCTAATCTTTTCCAGCCGACTTCTACAATCTGTCATTCTTTCCAAGAAACACCCAGCGTCCAGATATAGATTTATTGGAGGGTTGTGAGAATATGGGAGATCTGACATGTCGTCATTCAGAGAGAAAGGGGTTCTGAACATTATGACCATGGAATACCCTGGAACGATGAGGTGGTAGCTCGCCTATGCCACCCTCGGATAAAGAAAGTAGATGAGAAGCCGATGAGTATGATTACCCGATCATGTATTTCTTCATCCGGGTAATCGCACAAAGTCGTTATCGTCGTTTTGCCCATATTCGGCTTCTCGTCTGAATATTGAGCAGCAACATGCAGTGCATGACATTCATTGCCCATGCGGAGAGAGCAGAATTCATTCATGTCTCTTGGTAGGAGTTGGACAACAATAACATGCATAGGTACTACTGGTACCTTAATTCTTTGTCCCGGATGACCGCTTCTCAGGAAGGAATCAAATAAACGCCCTTGGGAGAGTCGAGTCTTCCTCCTCCTCCTTGCTCCAACAGTCTCGCCCGCATCAATGTGTTTGACATCTGAGGAAGAGATCGTCATGCTTCTGGTCTCCTCTCGGGTGTCTTCATGCACATCATGAAGATGATCATGGTCACCTGCACCCACTTCGAAAACCGCCAAACACTCCCGCTTCATGCCCTCTGGGTTGATTCCTCCAACCCACCGCCGCATTCGGCGGGCCCCCACGGCCTGATGCCCAGCCTGCCTCCTCCACCAGCCACCCGGCCTCCCCATCATCCATCCTCCTCTCCCCACTCTTTGCCTCGCGTCCCTCTCGGCCCTGGATCTCCCATGCCACGTGCTCGTGGTCATGGTCGAGGTCGTCCGTGGGATGGGTGCTCGCTTGGTCCATGGGTTTGCGGCATTTTTTCTCCATGTGATAACATTTGCTGATGTCCATGAGGTGTACGGCCCGGCCACGGAGGCTGACGACAATCTTCGCCGTCCAGCTTCCTCCATGGGCATGCCTTTTGTCCCTCGTTCGCGATGTGCCTCTCCATCCTCCCCCGGAGATCAATGAGTGGCACCCAGCCAGTGTCTGGTGCCTTTATGTCGTAGACGATGAAGATGAAGAAGTCTATGTTGATGATCGCAGGAGTTTGCCCCGCCTCCCCATGGGGATCCGGTCAATCATCCTCCCACCTCCGCCTCCTTACCCGGGTCCGGACCCATTCTGGTGACCCTAGGCCATCCGGCTCGTTACCACCACCCTCCACCTCCTCCCGATCATCGTACATCTTGATCTGCTTTTTCTAGGTGATGAGCCACCTCGACTCCACTTTTGAGGAGGACGCGATGAGACTTTTTCCGACTACACTTCATATCCTCCGTCATATCCTCCTCCTGGAGGATATTGATTCTGTATCCTTGTCTCTCTCTCGTTTTGGTACTTGTTGCCAAAGGGGAGAAAAATCCTTCTAGTTTGTGGTTCTTCGTTTTTCTTTTGGATTATTTTTGTTTTTAGCCACCGTTGTGGCTTGTACCCTTACCATTGTATGAAAAAACGTTGGATTGTTATTCAAAGTAATATGATGGTCTTGGCCATCATCTTTTGTCCTATCGCTTTGTAATGTCTACTTATGAAGTAAAAATAATGGTTTTTAGAGATAGTCATGTATCACAACTCTGTCATGACACTCTTGCACCCTCGGATAATTGTATTTAGTATGGTTGTTGTCACTATCTCTAAATGTGTTGCTCACATGACATATTATGTCAAGACGTTGGATTCACAATCGTGCACATTTAGGAGAGCCATCTACATACAATCATTCAAAAGAAATTTTCTATTGCAAATATATATCTTTTGACCTTAATTGTTTGGCATCAATCACCAAAAGGGGGAGATTGTAAGTGCAATCAACCCCAAGTGAGTTTTGGTGATTTAATGACAAAACAAATAAGAGTACTAACAATTTTGTTCTCAGTGTTTGATTAGAAGGAAACAGAGAACTTAAGGAAGCACCAAGGACTTCATGCAACAGACGTATAAAATTTATGTAAAATCTTGTGTTGCATGATGTAATTCTTCCTTGTTCTTTTCCTGCATTGTGCATGTTTGCTATAGCTCAAGTCCTCTAATTCAAAAGCTATTTTTGAAAACCAAAAATCTCTTTAACATTATTTGGTGACCATGGTTTGGGTTGAGAAACCTAGAGTGTTCTTGAAAACAATGAACTTCTTCAGCAACTTGGTGAGCTTTCCAGAACTTAACAGTTGTGATGAGCTTCTTCAATTCGAACAGCTTTTCAGTGTTTGGACTTAACTGAGTTGAACTTTCAACTCGGTGAACTTCAACTTCATTGTGGACCTCGACCATACCAAATGAACTTGCCCTGGGCGGTGAGCTAGGGTTTCTCTCAAAACTCTCGGTCTAGACCACCAACCGGTCTGGGGTAGCTCAACGGTCTCGACCCTCGACCTCCCGATCTGCGATGCCGGTCGGTGGCGATCAGTGCGCCGAGGGTGTCGGGCGTTCAACGCCCTAGGGGCGGTCAACCGATCCGGCTCGTCTGACCGCCTGCAAGTCAGTCGGGTCGATGCCCGGTCGGTGTGACGATCTTACGACTGTCGGCGGTTCAAAGCCTAGGGGCGTTCAACCGGTTTTCAGCGGGTTTTTGGTCAAGCGGCTAGTTTTGAGCCGTGACTATAAATAGACCCCTCTCTCTTCTTCAAGACAATGAACACTCACTCATTTATTGCTCACCTAACTTGAGACAAAGCACTCATCTCTCCATCTCTCTCACACTTAAATCTTCCTCAAGATTCAACCTTGTTGGAGGAGCTCTTGGGTGTGAGGTTTGTGCTTGTGCTCACGATTTGGTTTTCCTCTCCCATCTCTTTTTCAAAGACTTGAGCTTGTGCAAACCCCTCTTGTGCGTTCTTGTGTTCTTGAAACCCTAGGTTTCAAGATCTTCGAGGTTACTGGGAGTCTCCAAATTTGTGGGACGACCCTAAGAAGTTGGTATCACCCGCTCTTTGAGCTAAGATAAGAAGAGATTGCCTTGACCTTTGTGCGGCTTTGGAGGATTAGGGTTGAAAAAGACCCTACCCTTTGTGGGCCTCAACGGGAGTAGGACACCTTGTGGTGTGGCCGAACCTCGGATTAAATCTTGTGTCTTGTGTTCTTACTTGTTTCAACTTGAGATATTTTTACTTGCAAGATTGACATAAAGATTTCTCCGTAGAGTTTATCTAGAAGTAAAATAGCCTTTACATATTCATCTTTCATCCTCACTTAGCTATATCTTACTTCTCTCAAGAACTTGCAGTAAATACTTTCGAGTAGATTTTAGTCTAAAGTTTATAAAGCAATTGGATTGGTTCAGTGGTTCAACTTATGACGCGTAGGCTGTTGAACCGCTGCACCCGAACTGTATATAATCTTCAAGTTTGTTGTGAAAATTTTCAGATTAGCCTATTCACCCCTCTAGGCTACTCACTACCTAGACTGGTCCGACAAGCCCATCACCTTCGACCAAGCGACCACCCCGACCACGTCGAGCCCGGGAAATACACTTCGTTGTCGACCCGATCACCGGTGACGCCGAGGCTCACCAAGGTCCTCGGATGGACGACAGCCTCAACATCATCTATGCCGAGACCCTCGCCTCCTGCGTGTTGATCTATCCTCGGTCCGCAGCGCTGCGCCATTCCACGATCATCCCGAAGCGCGCGTCAACCCCTCGGACAACTCGACCTTCCGTGCGCGAACACCCTCCAACCGAAGGGAGACCCTCACGTTGGGTGGTCGGGTTCGAGAACCTACCACGCATCTTGGGGGAGGCCATGCTACGAGTTCATGGCCGTCCCAACTACACCTACCTCAAGCTCAAGATGCCGCCCCAGCGGGTCATCACCGTCGCCCGCACGCAAACACGTTGAATGCGGCGTGGAGTGCGTGGAGTGCGCCGAGGCCTCGCCGAATCCAAGGCCTCATCGCCACTAAAGCCCTCTGGGAGGTGCCGACGTGAAGCGCCACGCCGACAACCGAGCCACGAGACGGTTAAGTCCGCCCCTCGACCCATGACGGCCTCAAGCGAATCGGATCGGCTCCGAGCTCGATCCCAAATAGAAGCAGTGCTCGCCGACTTTCTCGCGCAAACGCCGACGTTTTGGAGTCCCTGACATGCCCGGTATACCGGGGATGTCGCCGAGCACTCACTGGATATCCGAGTTGGAGCAGGCAAGTGAAGCGGCCTAAATTCGATGAAGAAAAGCGCAGAGCCATAGGCGAGAGATCCACAAGCTAATGGCAGCAGGTTCATCAAAGAGGTATTCCATCCCGAATGGCTTGCCAACCCCGTTCTTGTGAGAAAGAAAGGAGGAAAGGCGGATGTGTGTAGACTACACTGGTCTAAACAAAGCATGTCCGGGGTTCCCTACCCTCCGCCTCGCATCGATCAAATTGTGGATTCCCTCTGGGTGCGAAACCTTGTCTTTCCTCGATGCCTACTGGCTACAAATCGGGATGAAAGAATCCGACGGCTCGCGACTTCTTTCATCACACCCTGGCATGTCTGCTATGTCACCATACCGCTCTTCGAGGAATGCGTGCGACGTACCGGGCGTGCATGAACCATGTGTTCGGCGAACACATTGGCCGGACGGTCGAGGCCTACGCCGATGACATCGTGGTCAAGACGAGGAAAGCCTCAGACCTCCTTTCCGACCTTGAAGTGACATTCCAGTGTCTCAAAGCGGCGGCGTGCTTAATCCAGAAGTGTCTTTGGGGTTCCTCGAGGCATGCTCTTGGGGTTCATTGTCTCAGTGGGGCATCGAGGCCAATCCGAGAAGATCGCGCCATCACGGCATGGGGCCCATCAAGGACTTGAAGGGCGGAGAGTCACGGATGTCTTGCGGCTCGAGCCGCTTCATCTCACGCCTGGCGAAAGAGGCTGCCTCTATCGCCTCTTAAGGAAGGCGAGTGCTTCACTTGGACCCGAGGCCGAGAAGCCTGGAACTCGGCGCTCTCACGAACGCGCCTATCTTGGTGCCCCCCACGGAGAAGCCTATTGATCTACGCCGCGCGACCACTCGGGTGCTAAGCCCGCGATCGTGGTTGAGAGACGAAGAGGCATGCATTCGGAAGGCAAGTCTACTTCATCGTAGTCTTGTCAAAGACCAAGATCCGCTACACAATTCAAAGGCTGCTGACGCGGTGATCCCGACGCGGAAGTTGCGACACTACTTGAGTCTCATCCGTGAATGTGGTGTCATCCTTCCCCGGGAGATCATCTAGTGCCGAGAGGCCTCGTAGAATCGCAGTGGGCGGTGGAAATCATGGGCGGCGATCTCGCTCGCTTCTCGGAAGGCCATCAAGTCCTGTGTCTCGCGGACTTCGTGGTGAATGGGTCGACACCGGCTCGATTCCCGTCTGAACTCTCGACCATGTTCTTCGACGGGTCGATGAAGCAGGAGGCGGCAGAACCTCGCTCTTCATCTCGCCTCGGGAACACCTCGCTATATGTGCTACGCCCCACTTCCTCCGAACAATGTGAGTCGAGGCTCGGTCGCGGGTTCATCGCCATCGGTGGGGTCCGACGCCTCGGCGCCGTGGTGACTCGCTGACTCGTCATCGACCAAGTCGAAGAACTCCCCCGCCGCGACCAAGATGGAGGCTGCTGCGATGACGTTCGGTGCCGGAAGACAAGTTCTACGGCTCGAGCTCAACCACACGCCGACGCTACAACGACCTGATAGTTTAAAGCGGCGAAACGACAATCTGATCAAGCTCAAGCTGCTCTAGCATTCGCCATTCAGGCTGCGCAACTGTTGGGAAGGGCGATCGGTGCGGGCCTCTTCGCTATTACGCCAGCTGGCGAAAGGGGATGTGCTGCAAGGCGATTAAGTTGGGTAACGCCAGGTTTTCCCAGTCACGACGTTGTAAAACGACGGCCAGTGCCAAGCTTGCATGCCTGCAGTGCAGCGTGACCCGGTCGTGCCCCTCTCTAGAGATAATGAGCATTGCATGTCTAAGTTATAAAAATTACCACATATTTTTTTGTCACACTTGTTTGAAGTGCAGTTTATCTATCTTTATACATATATTTAAACTTTACTCTACGAATAATATAATCTATATACTACAATAATATCGTGTTTTAGAGAATCATATAAATGAACAGCTAGACATGGTCTAAAGGACAATTGAGTATTTTGACAACAGGACTCTACATTTTATCTTTTTAGTGTGCATGTGTTCTCCTTTTTTTTTGCAAATAGCTTCACCTATATAATACTTCATCCATTTTATTAGTACATCCATTTAGGGTTTAGGGTTAATGGTTTTATAGACTAATTTTTTAGTACATCTATTTTATTCTATTTTAGCCTCAATTAAGAAAACTAAAACTCTATTTTAGTTTTTTATTTAATAATTTAGATATAAAATAGAATAAAATAAAGTGACTAAAAATTAAACAAATACCCTTTAAGAAATTAAAAACTAAGGAAACATTTTTCTTGTTTCGAGTAGATAATGCCAGCCTGTTAAACGCCGTCGACGAGTCTAACGGACACCAACCAGCGAACCAGCAGCGTCGCGTCGGGCCAAGCGAAGCAGACGGCACGGCATCTCTGTCGCTGCCTCTGGACCCCTCTCGAGAGTTCCGCTCCACCGTTGGACTTGCTCGCTGTCGGCATCCAGAAATTGCGTGGCGGAGCGGCAGACGTGAGCCGGCACGGCAGGCGGCCTCTCCTCCTCCTCACGGCACCGGCAGCTACGGGGATTCCTTCCCACCGCTCCTTCGCTTTCCCTTCCTCGCCCGCCGTAATAAATAGACACCCTCCACACCTCTTTCCCCAACCTCGTGTTGTTCGGAGCGCACACACACAACCAGATCTCCCCAAATCCACCCGTCGGCACCTCCGCTTCAAGGTACGCCGCTCGTCCTCCCCTCTCTACCTTCTCTAGATCGGCGTTCCGGTCCATGGTTAGGCCCGGTAGTTCTACTTCTGTTCATGTTTGTGTTAGATCCGTGTTTGTGTTAGATCCGTGCTGCTAGCGTTCGTACACGGATGCGACCTGTACGTCAGACACGTTCTGATTGCTAACTTGCCAGTGTTTCTCTTTGGGGAATCCTGGATGCTCTAGCCGTTCCGCAGACGGGATCGATTTCATGATTTTTTTTTTCGTTGCATAGGGTTTGGTTTGCCCTTTTCCTTTATTTCAATATATGCCGTGCACTTGTTTGTCGGGTCATCTTTTCATGCTTTTTTGTCTTGGTTGTGATGATGTGGTCTGGTTGGGCGGTCGTTCTAGATCGGAGTAATTCTGTTTCAAACTACCTGGTGGATTTATTAATTTTGGATCTGTATGTGTGTGCCATACATATTCATAGTTACGAATTGAAGATGATGGATGGAAATATCGATCTAGGATAGGTATACATGTTGATGCGGGTTTTACTGATGCATATACAGAGATGCTTTTGTTCGCTTGGTTGTGATGATGTGGTGTGGTTGGGCGGTCGTTCATTCGTTCTAGATCGGAGTAGAATACTGTTTCAAACTACCTGGTGTATTTATTAATTTTGGAACTGTATGTGTGTGTCATACATCTTCATAGTTACGAGTTTAAGATGGATGGAAATATCGATCTAGGATAGGTATACATGTTGATGTGGGTTTTACTGATGCATATACATGATGGCATATGCAGCATCTATTCATATGCTCTAACCTTGAGTACCTATCTATTATAATAAACAAGTATGTTTTATAATTATTTTGATCTTGATATACTTGGATGATGGCATATGCAGCAGCTATATGTGATTTTTAGCCCTGCCTTCATACGCTATTTATTTGCTTGGTACTGTTTCTTTTGTCGATGCTCACCCTGTTGTTTGGTGTTACTTCTGCAGGTCGACTCTAGAGGATCCAACAATGGACAACAACCCCAACATCAACGAGTGCATCCTACAACTGCCTGAGCAACCCCGAGGTGGAGGTGCTGGGCGGCGAGCGCATCGAGACCGGCTACACCCCATCGACATCAGCCTGAGCCTGACCCAGTTCCTGCTGAGCGAGTTCGTGCCCGGCGCCGGCTTCGTGCTGGGCCTGGTGGACATCATCTGGGGCATCTTCGGCCCCAGCCAGTGGGACGCCTTCCTGGTGCAGATCGAGCAGCTGATCAACCAGCGCATCGAGGAGTTCGCCCGCAACCAGGCCATCAGCCGCCTGGAGGGCCTGAGCAACCTGTACCAAATCTACGCCGAGAGCTTCCGCGTGGGAGGCCGACCAACCCCGCCCTGCGCGAGGAGATGCGCATCCAGTTCAACGACATGAACAGCGCCCTGACCACCGCCATCCCCTGTTCGCCGTGCAGAACTACCAGGTGCCCCTGCTGAGCGTGTACGTGCAGGCCGCCAACCTGCACCTGAGCGTGCTGCGCGACGTCAGCGTGTTCGGCCAGCGCTGGGGCTTCGACGCCGCCACCATCAACAGCCGCTACAACGACCTGACCCGCCTGATCGGCAACTACACCGACCACGCCGTGCGCTGGTACAACACCGGCCTGGAGCGCGTGTGGGGTCCCGACAGCCGCGACTGGATCAGGTACAACCAGTTCCGCCGCGAGCTGACCCTGACCGTGCTGGACATCGTGAGCCTGTTCCCCAACTACGACAGCCGCACCTACCCCATCCGCACCGTGAGCCAGCTGACCCGCGAGATTTACACCAACCCCGTGCTGGAGAACTTCGACGGCAGCTTCCGCGGCAGCGCCCAGGGCATCGAGGCAGCATCCGCAGCCCCACCTGATGGACATCCTGAACAGCATCACCATCTACACCGACGCCCACCGCGGCGAGTACTACTGGAGCGGCCACAGATCATGGCCAGCCCCGTCGGCTTCAGCGGCCCCGAGTTCACCTTCCCCTGTACGGCACCATGGGCAACGCTGCACCTCAGCAGCGCATCGTGGCACAGCTGGGCCAGGGAGTGTACCGCACCCTGAGCAGCACCCTGTACCGTCGACCTTTCAACATCGGCATCAACAACCAGCAGCTGAGCGTGCTGGACGGCACCGAGTTCGCCTACGGCACCAGCAGCAACCTGCCCAGCGCCGTGTACCGCAAGAGCGGCACCGTGGACAGCCTGGACGAGATCCCCCTCAGAACAACAACGTGCCACCTCGACAGGGCTTCAGCCACCGTCTGAGCCACGTGAGCATGTTCCGCAGTGGCTTCAGCAACAGCAGCGTGAGCATCATCCGTGCACCTATGTTCAGCTGATTCACCGCAGTGCCGAGTTCAACAACATCATCCCCAGCAGCCAGATCACCCAGATCCCCTGACCAAGAGCACCAACCTGGGCAGCGGCACCAGCGTGGTGAAGGGCCCCGGCTTCACCGGCGGCGACATCCTGCGCCGCACCAGCCCCGGCCAGATCAGCACCCTGCGCGTGAACATCACCGCCCCCTGAGCCAGCGCTACCGCGTCCGCATCCGCTACGCCAGCACCACCAACCTGCAGTTCCACACCAGCATCGACGGCCGCCCCATCAACCAGGGCAACCAGCGCCACCATGAGCAGCGGCAGCAACCTGCAGAGCGGCAGCTTCCGCACCGTGGGCTTCACCCCTTCAACTTCAGCAACGGCAGCAGCGTGTTCACCTGAGCGCCCGTGTTCCAACAGCGGCAACGAGGTGTACATCGACCGCATCGAGTTCGTGCCCGCCGAGGTGACCTTCGAGGCCGAGTACGACCTGGAGAGGGCTCAGAAGGCCGTGAACGAGCTGTTCACCAGCAGCAACCAGATCGGCCTGAAGACCGACGTGACCGACTACCACATCGATCAGGTGCGAGGCCCCGGAAGGGTGGAGGAGCTTCCGTGCTCAACTCCGGACGCACCACCATCTGCGACGGCCACAACGTGATGGCCCAGGACCCGTTCTCCTTCCAGTACAAGTCCTCGACACCATCCAGAAGGAGTGGACCGAGTGGAAGCGCGACGACCACTCACTCTACGTGGCTCCTGTGGGTACTGTGGCCATCTTCCTCCTCAAGAAGCTCGGAGGCTTCGCTGCCAAGCGCATCCTCAATGGCCTCTGGGACCTCGTGTTCCCAAACGACAACACCAAGCTCATGCAGGACATCCTCCGTGAGACCGAGTTCCTCAACCAGCGCCTCAACGCTGACACCCTCTCCCGCGTGAACGCCGAACTTGAGGGCCTCCAGAAGAACGTTGCCGAGTTCAACCGTCAGGTGGACAACTTCCTCAACCCGAACCGTAACCCGACTCTCCTTTCCATCACATCCCCGGTGAACACCATGCAGCAGCTCTTCCTCAACCGCCTCACCCAGTTCCAGCTCCGCGGCTACGAACTCCTTCTCCTTCCTCTTCGCTCAGGCCGCCAACCTCCACCTCTCCTTCATCCGCGATGTGATCCTCAACGCAGAGGAGTGGGTATCTCCGCTGCCACTCTCCACCTACCGTGGCTACCTCAAGGACTACACCAAGGAGTACTCCAACTACTGCATCAACACCTACCAGACAGCCTTCAAGAAGCTCAACACCCTCTCCACGAGATGCTTGAGTTCCGCACCTACATGTTCCTCAACGTGTTCGAGTACGTGTCCATCTGGTCCCTCTTCAAGTACCAGTCCCTCCTCGTGTCCTCCGGCGCCAACCTCTACGCCTCCGGCTCCGGCCCGCAGCAGACCCAGTCCTTCACCTCCCAGGACTGGCTGTTCCTCTACTCCCTCTTCCAGGTGAACTCAACTACGTGCTCAACGGCTTCTCCGGCGCCCGCCTCTCCAACACCTTCCCGAACATCGTGGGCCTCCTGGCTCCACAACTACCCACGCCCTCCTCGCCGCTCGCGTGAACTACTCGGTGGCATCTCTTCCGGCGACATCGGCGCTTCCCGTTCAACCAGAACTTCAACTGCTCTACCTTCCTCCCTCCGCTCTCACACCGTTCGTGCGCTCCTGGCTCGACTCGGCTCCGACCGCGAGGCGTGGCTGTGACCAACTGACCGAGTCCTTCGAGACCACCTCGGCCTCCTCCGGTGCCTTCACCGCACGCGGCAACTCCAACTACTTCCCGGACTACTTCATCCGCAACATCTCCGGCGTGCCGCTCGTGGTGCAACGAGGACCTCCGTCGCCCACTCCTACAACGAGATCCGCAACATCGCCTCCCCGTCCGGCACCCTGGGGTGCACGCGCTTATGGTGTCCGTGCACAACCGCAAGAACAACATCCACGCTGTGCACGAGAACGGCTCCATGACCCACCTCGCCCAAACGACTACACCGGCTTCACCATCTCCCCGATCCACGCTACCCAGGTGAACAACCAGACCCGCACCTTCATCTCCGAGAAGTTCGGCAACCAGGGCGACTCTCTCCGCTTCGAGCAGAACAACACCACCGCTCGTTACACCCTCCGCGGTAACGGCAACTCCTACAACCTCTACCTCCGCGTGTCTTCCATCGGCAACTCCACCATCCGCGTGACCATCAACGGCCGTGTGTACACCGCCACAAACGTGAACACCACCACAAACAACGACGGCGTGAACGACAACGGTGCTCGCTTCTCCGACATCAACATCGGCAACGTGGTGGCCTCCTCCAACTCCGACGTGCCGCTCGACATCAACGTGACCTCAACTCTGGCACAGTTCGACCTCATGAACATCATGCTCGTGCCGACCAACATCTCCCCGCTCTACTAAGAGCTCTAGATCTGTTCTGCACAAAGTGGAGAAGTCAGTCATCGATCAGGAACCAGACACCAGACTTTTATTCATACAGTGAAGTGAAGTGAAGTGCAGTGCAGTGAGTTGCTGGTTTTGTACAACTTAGTATGTATTTGTATTTGTAAAATACTTCTATCAATAAAATTTCTAATTCCTAAAACCAAAATCCAGGGTACTGAGCTCGAATTCGTAATCATGTCATAGCTGTTTCCTGTGTGAAATTGTTATCCGCTCACAATTCCACACAACATACGAGCCGGAAGCATAAAGTGTAAAGCCTGGGTGCCTAATGAGTGAGCTAACTCACATTAATTGCGTTGCGCTCACTGCCCGCTTTCCAGTCGGGAAACCTGTCGTGCCAGCTGCATTAATGAATCGGCCAACGCGCGGGAGAGGCGGTTTGCGTATTGGCTAGAGCAGCTTGCCAACATGGTGGAGCACACACTCGTCTACTCAAGAATATCAAAGATACAGTCTCAGAAGACCAAAGGGCTATTGAGACTTTTCAACAAAGGGTAATATCGGAAACCTCCTCAGATTCCATTGCCCAGCTATCTGTCACTTCATCAAAAGGACAGTAGAAAAGGAAGGTGGCACCTACAAATGCCATCATTGCGATAAAGGAAAGGCTATCGTTCAAGATGCCTCTGCCGACAGTGGTCCCAAAGATGGACCCCACCCACGAGGAGCATCGTGGAAAAGAAGACGTTCCAACCACGTCTTCAAAGCAAGTGGATTGATGTGAACATGGTGGAGCACGACACTCTCGTCCTCCAAGAATATCAAAGATACAGTCTCAGAAGACCAAAGGGCTATTGAGACTTTTCAACAAAGGGTAATATCGGGAAACCTCCTCGATTCCATTGCCCAGCTATCTGTCACTTCATCAAAAGGACAGTAGAAAAGGAAGGTGGCACCTACAAATGCCATCATTGCGATAAAGGAAAGGCTATCGTTCAAGATGCCTCTGCCGACAGTGGTCCCAAAGATGGACCCCCACCCACGAGGAGCATCGTGGAAAGAAGACGTTCCAACCACGTCTTCAAAGCAAGTGGATTGATGTGATATCTCCACTGACGTAAGGGATGACGCACAATCCCACTATCCTTCGCAAGACCCTTCCTCTATATAGGAAGTTCATTTCATTTGGAGAGGACACGCTGAAATCACCAGTCTCTCTCTACAAATCTATCTCTCGACGCATGCCTACAGTGCAGCGTGACCCGGTCGTGCCCCTCTAGAGATAATGAGCATTGCATGTCTAAGTTATAAAAATTACCACATATTTTTTGTCACACTTGTTTGAAGTGCAGTTTATCTATCTTTATACATATATTTAAACTTTACTCTACGAATAATATAATCTATAGTACTACAATAATATCAGTGTTTTAGAGAATCATATAAATGAACAGTTAGACATGGTCTAAAGGACAATTGAGTATTTTGACAACAGGACTCTACAGTTTTATCTTTTAGTGTGCATGTGTTCTCCTTTTTTTTTACAAATAGCTTCACCTATATAATACTTCATCCATTTTATTAGTACATCCATTTAGGGTTTAGGGTTAATGGTTTTATAGACTAATTTTTAGTACATCTATTTTATTCTATTTTAGCCTCTAAATTAAGAAAACTAAAACTCTATTTTAGTTTTTTATTTAATAATTTAGATATAAAATAGAATAAAATAAAGTGACTAAAAATTAAACAAATACCCTTTAAGAAATTAAAAACTAAGGAAACATTTTTCTTGTTTCGAGTAGATAATGCCAGCCTGTTAAACGCCGTCGACGAGTCTGGACACCAACCAGCGAACCAGCAGCGTCGCGTCGGGCCAAGCAAACGGCACGGCATCTCGCGCTGCCTCTGTCCTCTCGAGAGTTCCGCTCCACTGTTGGACTTGCTCCGCTGTCGGCATCCAGAAATTGCGTGGCGGAGCGGCAGACGTGAGCCGGCACGGCAGGCGGCCTCCTCCTCCTCTCGGCACCGGCAGCTACGGGGATTCCTTTCCACCGCTCCTTCGCTTTCCTCGCCCGCCGTAATAAATAGACACCCCTCCACACCTCTTTCCCCAACCTCGTGTTGTTCGAGCGCACACACACAACCAGATCTCCCCAAATCCACCCGTCGGCACCTCCGCTTCAAGGTACGCCGCTCGTCCTCCCCCTCTCTACCTTCTCTAGATCGGCGTTCCGGTCCATGGTTAGGGCCCGGTAGTTCTACTTCTGTTCATGTTTGTGTTAGATCCGTGTTTGTGTTAGATCCGTGCTGCTAGCGTTCGTACACGGATGCGACCGTGTTTCTCTTTGGGAATCCTGGGATGGCTCTAGCCGTTCCGCAGACGGATCGATTTCATGATTTTTTGTTTCGTTGCATAGGGTTTGGTTTGCCCTTTTCCTTTATTTCAATATATGCCGTGCACTTGTTTGTCGGGTCATCTTTTCATGCTTTTTGTCTTGGTTGTGATGATGTGGTCTGGTTGGGCGGTCGTTCTAGATCGGAGTAAATTCTGTTTCAAACTACCTGGTGGATTTATTAATTTTGGATCTGTATGTGTGTGCCATACATATTCATAGTTACAATTGAAGATGATGGATGGAAATATCGATCTAGGATAGGTATACATGTTGATGCGGGTTTACTGATGCATATACAGAGATGCTTTTGTTCGCTTGGTTGTGATGATGTGGTGTGGTTGGGCGGTCGTTCATTCGTTCTAGATCGGATAGAATACTGTTTCAAACTACCTGGTGTATTTATTAATTTTGGAACTGTATGTGTGTGTCATACATCTTCATAGTTACGAGTTTAAGATGGATGGAAATATCGATCTAGGATAGTATACATGTTGATGTGGGTTTTACTGATGCATATACATGATGGCATATGCAGCATCTATTCATATGCTCTAACCTTGAGTACCTATCTATTATAATAAACAAGTATGTTTTATAATTATTTTGATCTTGATATACTTGGATGATGGCATAATGCAGCAGCTATATGTGGTTTTAGCTGCCTTCATACGCTATTTATTTGCTTGGTACTGTTTCTTTTGTCGATGCTCACCCTGTTGTTTGGTGTTACTTCTGGGTCGACTCTAGAGGATCTACCATGGCCACCGCCGCCGCGCGTCTACGCGCTCACTGGCGCCACTACCGCTGCGCCCAAGGCGAGGCGCCGGGCGCACCTCCTGGCCACCCGCCGCGCCCTCGCCGCGCCCATCAGGTGCTCAGCGGCGTCACCCGCCATGCCGATGGCTCCCCGGCCACCCTGCTCCGGCCGTGGGGCCCCACCGATCCCCGCAAGGATCCGACGCCCTGCCCGCCACCTTCGACGTGATCGTGCATCCAGCTCGCGAACTCCGCGGCGAGCTTCGCGCTCAGCCATCCAAGAACTACACCACTCGCTGCCTCCTCGCCGCTGCCCTCGCTGAGGGCGAGACCCGCGTGGTGGGCGTGGCTACCTCTGGACGGAGGCCATGCTCCGCTGCCTCCGCGACTGGGGCGCTGGCGTGGAGCTTGTGGGCGATGACGCCGTGATCCGCGGTTTCGGCGCTCGCCCACAGGCCGGTGTGACCTCAACCAGGCAACGCTGCCGCGGTGGCCCGCCTCCTCATGGGCGTGGCCGCTCACCTCTGGCACCACTTTCGTGACCGACTACCCGGACTCCTCGGCAAGCGCCCTCAGGGCGACCTCCTGAGGCCCTCGAACGCCTCGGTGCCTGGGTGTCCTCCAACGACGGTCGCCCCGATCTCCGTGTCCGGCCCAGTGCGCGGTGGCACCGTGGAGGTGTCCGCCGAGCGCTCCTCCCAGTACGCCTCCGCCTCATGTTCCTCGGCCTCTCCTCCCGGACGGACTCGAACTCCGCCTCACCGGCGACATCAAGTCCGCTCCGCTCCGCCAGACACTCGACACCCTCTGACTTCGGCGTGCGCGCCACTGCCTCCGACGACCTCCGCCGCATCTCCATCCCGGGTGGCCAGAAGTACCGCCCAGGCCGCGTGCTCGTGCCGGCGGCGACTACCCGGCTCCGCTGCCATCCTCACCGCCGCTGCCCTCCTCCCAGGCGAGGTCGCCTCTCTAACCTCCGCGAGCACGACCTCCAGGGCGAGAAGGAGGCCGAACGTGCTCCCGAGATGGGCGCTGACATCGTGCGCGAGGGCGATACCCTCACCGTGCGCGGTGGCGCCGCCTCTCCACGCCGTGACTCGCGACGGCGATTCCTTCACCGACGCCGTGCAAGCCCTCACCGCCGCTGCTGCCCGCCGAGGGCGACACCACCTGGGAGAACGTGGCCACTCTCCGCCTCAAGGAGTGCGACCGCATCTCGACACCAGCGCTGAGCAGAGCGCCTCGGCCTCCGCGCGCGCGAGACCGCCGACTCTCTCCGTGACGGCTCCTGCTCACCTCGGTGGCATCACCGCCGGACCACGGCGACCCGCATGATCATGCTCTCACCCTCCTCGGCCTCAGCGCGGGCCCACTCCATCACCGGCGCACACCACATCCGCAAGTCCTCGGTTCTTCGCTCACCTCGAAGCCCTCGGCGCTGCCGAGTACTTGAGGCCACCGCCTAATAGGGCTCGAGTTTCTCCATAATAATGTGTGAGTAGTTCCCGATAAGGGAATTAGGTTCCTAGGTTTCGCTCATGTGTTGAGCATATAAGAAACCCTAGTATGTATTTGTATTTGTAAAATACTTCTATCAATAAAATTTCTAATTCCTAAAACCAAAATCCAGTACTAAATCCAGATCCCCCGAATTAATTAGCGTAATTCATACATTAAAAACGTCCGCAATGTGTTATTAAGTTGTCTAAGCGCCAATTTGTTTACACCACAAAATAGCCTCGGGGCGAACTTGGTTCGGACGCCTTCTCCCGAGACCAGCATCAACCCTCCGTCAAGATCGACGACACGTCCGAGGCTCGAGGCACCTCGGCCCGGCCGAGCACCCTCGGCTCGATCGAGGCACCTCGGCTCGAGGCACCCTGGCTTCGGCAGGTACCTCGGCCCCGAGATAAGGCCTGCACGTCCGAGGGAGGCTGGCGGGTCACGCTAATCGAACCCGTGCACTGCAATATCTCCACCGAGGAGAGCTACCCTCGACCGAGCCGAAGCTGCGGTTGGTGCGCATGCCAAGTCGTTTGTCTTGGTGGGGGACGGAAGAGTCTACCACCGTAGCCCTCGGGCATCCTCCCGATGCATTTCCATCGCCAAGGCCGAGCTCCTACAGAGATACACTCGGGCTTCAAGCCATCACGCGACACCTCAGGCCTTGTTGGAAACGCCAAACAAGGTTTCTCTTAGCCCAGCAGCGATGGCAACATACACTAAATTGTCCGCACTGCGAAGGGTGTCGATTCTACGCAAGGCCAGACCACCGCTGCCAGGCCCCAGCGGACGATACCCATCACCGGCCTTTTGCGTGGGTCTGATCTCGTCGGCCCCTTGCGAAAGGCACAGGCTACGCACTGTTGGTCACCATCGACAAATTCTCCAAGTGGATCGAGGTCCGACCCTAAAAAAGATCGTCCGAACAAGCGGTGGCGTTCTTCACCAACATCATCCATCGTTTGGTCTGAGAGCACCTAGAGGGGGTGAATAGGTGATCTGTAAAAACTTAAACTTAAGCCACAAAAACTTGTTAAGGTTAGTACAAGTATGGCCAAGTGGCTAGAGAGAACTCAAAACACAATAACCACAAGAAATCACAGAGTTGACACGGTGGTTATCCAGTGGTTCGACCAAGTACAAAACTTGCTCCTTGCCAGGCGTCCCAACTGACGAGTTGCACTCAACTCCTCTCAAGTGATCCAATGATCAACTTGACCACGGTGTTTTCTTTCCTTTGATCTTTCCCGTTATGAATCTCCAACTTAGGTCTCTCCCGCCTACATTGAGTTCACAAAGAAATACGGAGTAAGGTGGGAATGAGCAACGCACACAAGACTCAAAATCGAGCAACAACACGCACACATGTCGCAACAAGAGCTGCAACGCAACACAAAGAGTTCACAACTCCACAAGAGCTCTATATGCTATCACAAAACGAATCGTGAGATTGATGTCTTGGTGCTTAGAAGAGTTGTAGGAATGTTGGTGTACTCTCCATGCGCCTAGGGTCCCTTTTATAGCCCCAAGGCAGCTAGGAGCCGTTAGAACAAATCCGAAGGCCATCTTTGCCTTCATGCGTCGGCGTACCGGAACCCGATCCGGTGCTGCTGACGCGTCGGTGCGATTGTTTCCTTAAATGGCGGCCGAGTTCTTCGGCAAGTGCAGATCTGGCGCCGAGACAATCGGTCACTGGATCGTCCGCCTTTCCGACCGTTGGCTCGCCACGTGTCATGCCGTCATGACCCGGCCGATTAACGGCCGTGTTGGCTCACCGGACATCCGTGCACCGGACGATCCGGTGAATTTTAGCCAATCGCGGAGAAAAACAGAGCGCCTCTTGGCCAGAGGCGATCTGGCGCCGGACATCTTCCCGGTCACCACGAGCGATCCGGTGCCCAGACAGAAGCTGACCCTTGGTGTACACACCAAGTCTTCTCTTCTCTTCTTCTATCTGTTTCTTTTAGACAAAATATATTAGTACACAAAACCAATGTACTAAGGCTTAGAAACATACCTTAACTTGTGATTTGCACTTTGTTCATCCTTGGGCATATTTTCACATTTAAGCACTTGTGTTTGCACTCAATCACCAAAATAAGAAATGGCCCAAGGCACATTTCCTTTCAATCTCCTTTTTGGTGATTTATGCCAACACAACATAAAGTAGATAGCACAAGTGCAAAATCACTTCAAATAAAAACTCAAATTGGTTTGATTCAATTTGGCATATATGGATCATCCTTTGCCACCACTTGGTTTGTTTTGCAAATCAAACTCAAATCTCTATCTCTAAGTCAAACACACATGATGAAGTATAAAGAGTCATTCCAAAAGATTGATCAAAGATTTCAAAACTCCCCTATTTCCCATAATCAACACTTCTCCACAAGAAGCCAACTTTTGACAATAGAGACAATAAGAAATAAAAGCTTTTGACAAAACAAAAACTCTATTCTACTATTAAAATCTCTCAAGTGGTAGTGATCCATTTATCACTTTGGCTTTATTTTCTCCCCTTTGGCATCAAGCACCAAAGCGGGATTAATCTTGGCCTTTTAACCCCATTGCCTCACCAAAGTCTTCAATTAAGAGCAAATGGCAATAAGATTTCATGAGATGAACTTGGAATTAGTTACCCTCTCATCCAGTCAATGGAAGTCTTTCATGGTCCAAGTCCACCTTTTCCCTTTCAATCCTCCTTGAGACTAAATCAAGCAAACTCAAGCAAATGGTTAGTCTCAAAGGGTCAAGTTGTAACACATCTCCCCTAAACGTGTGCATCACTTTGCAACGGACTTGTGAGTCGAGGAGTGTTTGTACAACTTGAGCACCATAATAAGCAACATAATGCAAAAGGAACATGATCAAAAGCATAACTACATGTATGCTACAATTCAATCCAGTTCCTGAATCTAAGACATTTAGCTCACTACGCATTTGCAAAGGTCTTCTCATCTAGAGCTTAGTGAAGATATCGGCTAGTGGTTCTGGTGCCATGCAGAACACTTGATATCTCCCTTTTTCTTCGGTGGTCTCTCAAAAGTGATGCCGGATGTCTATGTGCTTTATCTTGGTGTGTTCATGAGATTCTCCGCCATGGATAGCACTCTCATTGTCACATAGAGTGGGACTTTGCTCGATTGTAGCCAAAGTCCTCGAGGGTTTGCCTCATCCAAGTAGTTGCGCGCAACAACGTCTGCGGCAACATACCGGCCTCGGCGGTGGATAGACAGCGGAAGTTTGTTTCTTAGAGTTCCATGACACCAGGACCTTCCTAAGAATTGTACGTCCCATGTACTCTTCCTCGACCTTACATCCAACATAGTCGATCCGAGTATCCAACTAAGTCAAAGGTAGACCCTTTGGATACGAGGCGGCAGCGTAACAACTAAATATCTAGAATTATAAGTGACATTCCTTAGGATCGGATTGAAATCTAGCACACATGCATACGCTAAGCATAATATCCGGTCTACTAGCACATAAGTAAAGCAAAGAACCTATCATTGGCCGGTATGCTTTTTTGATCAACGGACCTCCTTTGTTGAGGTCGGTGTGTCCGTCGGTCCCCATCGAGTCTTTGCGGGCTTGCGTCCTTCATCCCAAACCGCTACTTGGATCTTGCGTGTGCCGTTTGGGAGGTGAAGTCCTTGAGTTGCTTCACTTGGAACCCAAGGAAGTAGTTCAACTCGCCCATCATTGACATCTCGAATTCTGCGTCATCACCACCTAAACTCTTCACAAGACTTTTGGTTAGTAGAACCAAATATTATGTCATCGACATAAATTTGGCACACAAACAAATCACCATCACATGTCTTTGTAAAAGAGTTGGATCGACCTTCCCAACCTTGAAAGCATTAGCAAGTAAAGTCTCTAAGGCATTCATACCATGCTCTTGGGGCTTGCTTAAGTCCATAGAGCCTTAGAGAGCTTACACACATGGTCGAGTCTGCGTTCATCCTCGAAGCAGGGTTGCTCCGTACACCTCCTCCTTGATTGGCCTGTTGAGGAAGCGCTCTTCATCCATTTGGAACTTTGAAAGAATGGTGGCGGCATATGCTAGCAAAATCGAATGGACTCTAGCCTAGCCACGAGGCAAAGTCTCCTCAAAGTCCAAACCTGCGACTTGGGCATAACCTTTGCCACAAGTCGAGGCCTTGTTCCTCGTCACCACCAGTGCTCGTCTGTTTGTTCTGGAACACCCACTTGGTTCCCACAACATTTTGCTTGGACGAGGCACACAGTGTCCAAACTTCATTGCGCTTGAAGTTGTTTGCTCCTCTTGCATGGCCAACACCTAGTCCGGATCTAGCAAGGCCTCTTCTACTGAAAGGCTCAATAGAAGAGACAAAAGAGTAATGCTCACAAAATTAACTAATCGAGATCGAGTAGTTACTCCCTTGCTAATGTCACCCGAATTTGGTCGTGGGATGATCCCTTTGAATCATCGCTCGAACTTGGGTTGGAGGTCACCCGCTTCTTCTTCTCCATCACTGATCATCTTGGCTCCCCCTTGATCAGCGCCTCCACTTGAGTTGGAAATTTGCACCATTGTTGAGGAAGTGATTGATCTCGTTCATCTTGTTCTGGCGCACTTCTCCAATCGCCATGGTTCGTTATAGCAGTAGTCAACATCTTCTTCATCTACATCATCACAATCAACAACTTGCTCTCTTGGAGAGCCATTAGTCTCATCAAATACGCGTCGCTAGAGACTTCAACCAAACCGATGATTTGTTGAAGACTCTATACGCCTTTATTTGAGTCATAACTAATAAAACCCTTCTACTGCTTTGGGAGCAAACTTAGAATTTCTACCCTTCACTAGAATGTAGCATTTACTCCCAAATACACGAAAACGATACATTGGGTTTGTTCACGGTTAGTAGCTCATACGCGTCTTCTTGAGGCGATGAAGGTAGAACCTGTTGATGGCGTGGCAGCGGTGTTCTGCGGCCAGTCCAAAAGCACTCGGGGTCTTGAACTCCCTAGCATCGTCCTCGCCATGTCGATGGCGTCCTGTTCTTCCTTTCTACCACACCATTTTCTTTGTGGTGTGTGGGAGCGGAGAACTCGTGTTGATCCCTTCATCTTCAAGGAACTCCTCCACTTGAAGGTTCTTGAACTCGGACCCATTGTCGCTCCTTATCTTCTTACCTTGAGCTCAAACTCATTTTGAGCTCTCCAGAGCGCTTGAGGTCCCTTGGGTTTGAACTTATCCAAAAAGAACACCCAAGTGAAGCGGGAAAAGTCATCAACAATAACTAAACCATACTTACTCCCTCCTATGCTCGAATAGGCGGCAGTCCAAGAGATCCATATGCGGACTCGGGGTCTTGAAGTGGTCATCACGTTCTTCTGTGATGTGCTCCTCCCACTTGTTTACACCGCGACAATTTGCACAAGTCTATCTTTTTAAATACACGTTAGTCAATCCTATCACGTGTTCTCCTTTAGAAGCTTGTGAAGGTTCTTCATCCCCACATGTGCTAAGCGGCGTGCCACAATTGATCCATAAAGTCTTAGCCATTAAGCATCTAGAAGCCTCTTCTTTTGCAAAATCAACTAAATAAAGTTTGGTCTAATACACCCTTAAAAGCTAGTGAACCATCACTTCTTCTAAGCGAACACATCTACATTTGTAAATAGACAGTTATACCCCATATGACATAATTGACTAACATAGCAAATTATATCCAAGACTCTCTCTAAAAATACATTGGAGATAGAATGCTCATTAGAAATCGCAATTTTACCTAACTTTTACCTTGCCTTGATTCCCATCATAGAATATAATTGAATCTTGGAATCCTTATTTTTAGGAGGTGAACATCTTCTTCTCCCCATCATATGGTTTTGTGCATCCGTATCGATAATCCGGCTTGAACCTGGATGCATAAACACGCAAGGCAAATTTAGGCTTGGTTTAGGTACCCAACTCATGTTGGGTCCTACAAGGTTAACACAAATATCCTAGGGACCCAAATGCAAGTTTTGTCTCCCTTGCATTTTGCCTCTTCCTAGCAACAATTTTCTTATCCTTTCTACAAATAGCAAAGGAAGCATTTAAAGCATAATAAATGTAAGGTTCATTCACTACTTTCCTAGGAGCATGAATAACATTCTTTCACATGATGAATAGCATTTCTTTTAGGAACAACATTTCTCTAGTAACATTTCTATCATACACATAAGAGGAACTAGGAGCAAACATGGCATGAGAATCATAAACATATGAATCAAAGCATCATGACTTACATTTCTAGTTTGTCTTCTATCATGATACAAAAAGCATGGTTCTTTTGCACATTACTAGCCATAGGGACCTTCCCTTTCTCCTTGGCGGAATGGGGCCTTATGGCTTGTTAAGTTAGCTTCTCTCTTGAAGCCAAGTCCATCCTTAATTGAGGTGTCTACCAATTGTGTAGGCATCCCTTGCAAATTTAGCTTATCGAAATCATTCTTGCTAGTCTTAAGTTGAGCATTAAGACTAGCACTTCATCATTAAGCTTGGAAATGAAACTAGGTGTTCACTACAAGCATCAACGTCAAAATCTTTACACTAGTACAAATTTCAACATGTTCTACACAAGAATTGGATTTATTACTACTTCTAATTTAGCATTTAAATCATTGTTGACACCTTTCAAAGTAGAAATGGTTTCATGACAAGTAGATAGTTAAAAGAAAGCATTTCATTTCTTTTAACTTCTAAAGCATAGGATTTTTGTGCCTCAACAAATTTATCATGCTCTTCATACAACAAATCCTCTTGCTTTTCTAAGTATATTCTTTTCATTCAAGGCATCAATTAATTCATTAATTTTGTCTATCTTAGATCTATCTAAGCCCTTGAACAAACATGAATAATCTACTTCATCCTCATCACTAGATTCGTCCTCACTTGAAGAAGCATAGGTAGAGTTGAGTACATACCTTCTTCTCCTTTGCCATAAGGCATGTGTGGCCTTCGTTGGGAAGAGGGTTGATTTGTTGGCTGTGGCGGCGGAGTCCTTCATTGTCCGGTGGACGAGAGCAATCAGAGTCCCTCCTTGCTAGATCGCCTCACCCTTTGCCTTCTTATAATGCTTCTTCTTTTCCTCTTGTTTCCCTTTTCCTGGTCACTTTCATTATCGGGACAGCTTAGCAATAAAATGACCAAGCTTACATTTGAAACATGATCGCTTCCCCTTGGTCTTAGTCTTGCTCGGTATCCATTGCGTTCACCAGCACCGTCTTGAAGCGCTTAATGATGAGCCATTTCTTCATCATTAAGCCGGCTAGCCTCAATTTGCCACCTTGCTTGGTAGTGCCTCCTTGCTCTCGATGCCTTGAGGCAATGGGTTGAGGCTCGTGGATTGGACGTTCAGCAGCGTCATCCACGTACCTTGCCTCCTTGATCATCATCCGCAGCGCTTACAAATTTTCCAAGAATTTCTTGACATTTTGTGTACAGGATTCGAATATTATTCACCAAATGAGGATCAAACGATAAGACCTTAGCATGAGGCGGACGACGTCGTGGTCCGTCCATCGCGTGCTTCCATAGCTCCTTATCTTGTTGACAAGGGTCTTGGCCCGGTTGTATGTTTGAGTTGTCTCGCCTTATCATGCGAGCCAGTCAAGCTAGCTTCCAACTCCATTTGGTGAGCAAGGTAGCATCATTTCCCTCATGAGAGATCTTGAGGTGTATCAGTTGTAGCGTTATCCAAGCCACTCACTTTATTATATTCATCCTGCACATGAGGCTAGAAAACAGTAGTGCTGTGCATTCTTATGGATTTGCTCATTAATGAATAGGACTATCAGACTATTAAAGTGCATTCCACTATCTACAATCTCCCATGCTAGGATGGAGAGAGAATAGGTGACTCGCATTTTGTGGCTCCAAAATCCGTAGTCCTCCCCATCAAAGTGTGGGCTTCCGAGGTGGAATAAGCAAATGTGAATTTGAACTTTGAATAAGTGTCAAGAAAAGTTAAGTGCTTAGGTTTCCTTTGTTTGTCGTGGTGCGTCGTCCTTTTGGGAAGAAGGGACTCATCATTTGTCGTAGAGGCGATCTCCTTGATCGTCTTGTCTTCTTCTTCCCATCTCTTAAGCATTGGCCCGAAGGTCATTGGACTTGTCATCCCTTGGCTCGTTGAAAGACTCCTTCTCCTTGTCGTTGATCACAATTCCCTTCCCCTTAGGATCCATCTCTTGGGCGGTTAGTCCTTTCTTGAAGAAGAACGGCTCGATACCAATTGAGAGCACCTAGAGGGGGTGAATAGGTGATCTGTAAACAAACTTAAGCCACAAAAACTTGTTAAGGGTTAGTACAAGTATGGCCAAGTGGCTAGAGAGAACTCAAAAACACAATAACCACAAGAAAGCAATCCGCGAGTTGACACGGTGGTTATCCCGTGGTTGACCAAGTACAAAACTTGCCTACTCCACGTTGTGGCGTCCCAGCGGACCGAGAGTTGCACTCAACTCCTCAAGTGATCCAATGATCAACTTGAATACACGGTGTTTTTCTTTCCTTTGATCTTTTCCCGTTTGCGGAATCTCCACAACTTGGGTCTCGCCCTTACAATTGAGTTCTAAAGAAATACGGAGTAAGGTGGGAATGAGCAGCGACACAAGACTCGAAAATCGAGCAACAACACGCACACAAATCGCAACAAGAGCTGTAACAACACAAAGTTCACAACTCCACAAGAGCTCTATATATCACAATGAAGCGAATCGTGAGATTGATGTCTTGGTGCTTAGAAGAGTTGTAGGAATGCTTGGTGCTCCTCCATGCGCCTAGGGGTCCCTTTTATAGCCCAAAGCAGCTAGGAGCCATTGGGAACAAATCTGAAGGCCATCTTGCCTTACATCTGGCCCGCCGGACGATCCGGTGCACCACCGGACTCTGTCCGGTGCCGATTGTTTCTTTAAATGGCGGCCGATTGCCGGCAGATTAGTCTGCGCGCCGGACGATCCGGTCACACCGGACGATCCGTCTTCTTCCGGCCGTTGGCTCGGCCACGTTCGTAGCAGCGCGATGCGTGACGGCCGTTGGCCGCCGGCCGTTGGCTCACGGACGATCGGTACACCGACGATCCGGTGAATTTTGTAGCCGGGCCGAAAAAACCGAGAGCGGCCTCTTGGGGCCGATCTAGCGCCGCGGACCTTGTCCGGTCACCACCGGACTGTCGGTGCACCACCGGTGAATCTGGTGTGATGCGGCGAACCATAAAACACCGATAATCTTCTTTCTCTTCTTCTTCTTTGCTAACAGAGACAAATATATTAGTACACAAAACCAATGTACTAAGGCTTAGAAACATACCTTAACTTGATTTGCACTTGTTCATCCTTGGGCATATTTTCACATTTAAGCACTTGTGTTTGCACTCAATCACCAAAATACTTAGAAATGGCCCAAAGGCACATTTCCCTTTCATCCCGAACTCCATCATCACGACAAGCACCCGTTCCGCAAGCAAAAGTTCCCGACTTACCGCGAGGATCACCACATCCGGGTGGACGGGCCCGTGCTCACCCATAGCCGAATGGGCAGTAGAAGCGTGCCAGCGGCATGATTCTACAAGACTCAAGCCTCGGATCTACAACGACCTCAACAGTTCGCCAAGCGATGGATGAAGGAACTCCTCGGTGGTCTAGAGTCCGAGGACAACGCCCAGCCGAGCCCTGCGGCTTCAGCCGTTCTTTCTAGTCTATGGGGCGAGGCCATCTTGCCCCGGACTTAGAATACGCCCCCAGGGACGAGGGCCTCGGCACCAAAGCAACCAAGTTAGTCGAGAAGACTTCGCTGGACCCAATGGAAGAGGCTCGGACATGGCCTTACTACATTACGACACGGTACCCGGCGACGCTGACACCCGAGAGTGCGGTCAGAGACCTCCGTGGCGACACGGTGCTTGGTGCGGCAGGGCCCGGCGGCCTGGCTCACTCCACTCGGAAGGCCGTTGTCTTCATGCGTGCGAGGCTCGGGCGTACCAAGGCCGCCAGCGATCAAAGCGAGGTCCGCGACGCGCTTGGAACATCCAACACTCGTCCGCTTCTACCCTTAAGATGCTTTCGTTGTTCATATACCTCGCTCCCACGCAAAGTTTAGTCATCAGGAAGGGTCACCTTGCCTCAGCAGCCCGCCCTCCTCGGGGGCTAAAGGGGGAACGTCGAAATTTTCCTCAAAAAGATCCACTGATGTCTTTCGTGCTTTTCGACTACTCAAGTGGATCCACAACGGCGAGTACGTAGCGGTAAGGTGGCCAAGCGAGGACTCCTCGGCCCGGGATACGGATACCTCATCATCACCTTCGTGATAAGTAACTCGCGTCGGATAAGCGATCCTGGGCCGAACAAGTCTTCACACAGCTCCTCTCGGCGATTCTTGGCCCTTCTCGCTGTCGGTGGCGGAAACCCATGGGCGGGCAAGAGTGCACGTGGCGGCAAGGCCGGCCAAGTGAGGATTCTCCGCCTGGGATACGGATACCTCACTCGTCACCTTCCGTGAAAAGCAACTCCACGCTCGCATGACATCCGTTCTAAGCGAAAAGTCCAGATACTCAAAACAAGAGGAAAAGAAGCGCGGCTTTACAACAGCGATGGTGTGTTTGACCTCGCGGCCAGCGAGCGCACACTACAAGATAATCCGATCCTGCGAGCTTGATCTTGGCGAGTTGAAGAGCGGCAGGCTCGGCGCCGACTACACGCCGAGGTCCGACCAGCCTCGGCGGGGCGACGCGGTCGAGGATCACGAAGGACGACACATCATCATCACGCGCCATCGCCGCCGAGGTCTTCTCAAGAATCCGGCTCAGTAGGCGGCACCGGTCCGTCACCCGAGGCCTCGGCCACCGTCCAGAACATCGCCGGCCAGGGCCTCGCAGATCAACTCGGCGTCGGTCCCGCTAATGGGCGGCAGGCGGCTCGACAAGTCTTCTTTCGATAACTCTGCCTGCAATAAAATGCGACACCGCTACCACTGCCGGCTCATCGAGCCGGTAGGGTTCTTTGGCTAAGAAGCCTCGGGCTGCAAAGAAGGCCGTGAAGGACTCCCTCGCCTCCCGGGATACGGATACCTCACTCGTCACCTGTAGGCGACGCTCACTCTTGTGGCGGTTCGAGACAACCAACGGCAGTAGTCTTAGTGCTCGAAAATAAGGAAAAAACGGCTCTGCGCAAAAAACATACGTCGTGCCCGGCTGACAACAATGAACAAAGGCCGACATTCAAGGTGCCATTACAAACGGAACTCGGTTCCACCTCCGTAGTGCGAACAACCCACACGATTGGGGGCTGCGAACAGCGAGAACTAAACGAGCGGCTGCCGTCGCCCGCTCAACGGCGGCGGCGATGGCGACCGCTTTCAGGGCCTAACGGCGGCGACGATGACCTCGGGGTGGATGCTGGCGCCATGAGCCTCGCCATGCCCAAACTCGTGAGGCAGGGCCGGCGAAAGCGGGTAGAGTTGGAGGTCGTCGTGTGGCGGCTATCTTGTCGAAGAACCTCTTCCACCGCCATGGCGGAAGCGGGCGCGGGAGCGGCTCGGCCACTCCGGTCAGAGCGAACGCGCGGCCGCCGGTAGCCAGTGAACGGCGAACGCCCTTCCCTCGATCACGAGGAAGGAGCGGGCCGCCGCGTGAGGCCGACCCCAACTCGACACACTCCTCCCAAGACCGGCGATGAAAATCCTTGAGGGAGGGAGGAGCATGATGCCGGTTGCTTTCCCAGCCATCGAACCGAGGTCACCATCTTGGTGACCGCCGTGAGGGGTGCAGCCGGCCATGTGATGAAATCCTTGAAGCGAACGATGGGTGAAAGGTACCAACCCACGGAGTTGCGTTCCTCAACGACAAGGCGGAGGGATGCGGGTGTCCCATCCGGGGCTGGAAGGTGGAAAGACACGATACATGGGAGCTGCGAAGACATGGTCGCCTTTCAAGGGGTCACCCACCTTTTAAGCGACTCTCCTACTGCGCCCCGGTAGTAAGTGGTGAGTCTTCTCCAACGCTCCAGGTCCTCCCCCGGCGGGCCGGGACCATGGCGTGCATAAGCCTGGTGAGGAAGAAGCCAAACCGCTTGCGCGTGCATACAACGCCAATGTTCGAGCATTCTTCCACTTTCGGACCGGTGAGCGAAGGCGAACAACATGCGAGCGGCATGCAACGCGCCAAGTGGCGCCCGCCGACGCGCACCCGGCGATGGCTGCGCCAGCGTCCGCATAGCCTAGCGCCCGGCTACGATGCAGCTCAGACGCGCCGCCACTCACTGCTACGCGCCTCGGCTGCGAAACCGATCACCGCGACTCGAGGCGACCCTGCGTCACGACCAGCGGTGCGGCCAAGCAGCGATGGTCATCGGCCAAAAATGGGCCGTAATGGCGGTGGCGAGCGGTAGGGCGGCCGGTCGTCGTCGTAGCCCCATCTCCACAGGCGTTGAGAGAACCTCTCCACGGCGTGAAGGCGCGCCAGTGTTCCGTTCCTCGGCGGCGGCGCCGCAGCCGCAGCGGCCCGCCATGACGCTTCGCCGTCGCATTAACTCGGCGGCGGGAAAGCGAGCTCCGGCAGAAGGCGGCGACCGCGCCGCCACAATGACCGCGTCAAAAGTGCGCCACGCCATTCGATTTTGCATCCTTTCCTTTCCTCTTTCTCTCTCTTATAACGAGGGTGGGAAAGAGAGGATACCCCAAGGATCCTTCTCGAAGTGAAGGAAGCAGGCTCCAGAGCCTCCCTCGATAAGAGGTTCAAGGTGGCCCCTGAGGGGTTCAGCAGCGCCAGCCGGCGCGTGGGCTCCACACCCACTTGGTTAGAGGTTGAAGGTCGGCCCCTCGGAAGGTTCAACGGCGCCGCCAGACTCCGCGCCCTGCGATCGGGGTTCAAGGTGCGCCTCGAAGGGTTCACCGCCTCCCGAACCACGAGGATGACCATGGAGTCGCTCGATACATAACCAAGGCTCGGGCACGCTCCCGGAGGTACCTAAGACAGAGACGGCGAAATGATCTTGTATGGAATCCCATCGGAGGAGGTATCGAGCTCGGACCCGTCGACGAGGACCGGTCCTGATCACCGTAGAGTACTTTTGAGCGCGCCCGGACCTCTAGCGACCCCTAACAAATGGGGCCAGGCGTCCACTCGGATTACCCGCCAAGCTCGGAGACACCATGTTTGGCGCCCTCAGGGCAACATGGCGCTTTCCCCCTCCTTGCGGAAAGCGTGCGGCGTATGTTAAAGTCAGTCCATCGCGGCCGTCCTCTCGCCTGTATGGAGGCTCGAGGCGCTCGCAAACCGGCTCCGGCCAAACCGTTGACAACGCGTCAACATACGCCAAGAACTTGGGACGGCCGATGCACCGGGCTCTGGCCACTTCATGAGAGCGACGGTGGCGGCAAGCATTGCGTGAGGTATTAAGACCTGGAGAGTCAAACCACTCTCGAGGCCTCAAAGAGCATACACCGTAGTGCGGCTCGCACCCACCGGGATCGCCTGAGAAAGGCTGGTCCCAAAAAGTCGACAAAAGCCTCCAGCGGTGTTAGCACTCCCTTCGAGGCTCGGGGCTACTGGCCGGTACCATAATTAGGGTACCCTCAAGACTCCTAATTCTCATTGTAACCCCATCGGCACAAGGCTGCAAAGGCACGATGGTGCGACTAAATCGGATCGGTCCATTCGAGGGACTCGATCACGCCTCGCAAGTTAGCCTCGGGCAAGGCGGTTAACCTGGAGGATCTCGCTCGCCTGAGGCCCTCGGCGAACATACTCCAGCTCGCCGAGGCCCGTCTTCGCCAAGAAGCAACCCGCCAATCGCGCGCTGCAAACCAAATCGCGAGGGCATTTAATGCGAGGTGGCTGACACCTTTATCGACGCCACGCCTCCCATCAGAGCCGTGGCCGCGATCGCCGCCCGCTCAACAGTGGCCGACAGGAAGAAGCGGCCCAGCGCTGCCAGCTTCCGTGTGCCACTCAGCGAGTGAGTGACGGCGATCAGGCCGGCCTCAGCACCATAGGAAACTCCGCCGCCCGACCTAGGGCTCGGACTCGAGCCGGCCTAAGATGGCGACCGCTCTGCCGACCTAGGGCTGGACTCGCTCAACCCCGGCTGCGAACTCCGCTCCGCCCAGACCTAGGGTTCGGACTCGCTCGGCCCGAGGCGCGAACTCCGCTCCGCCCAACCCGAGCGCGGACTGCGGGCTCACCGGAAGATGGCGAACTCATCACTTAGGCGGGCTCGGACTGGGCTCGGCCCGAGGCGACGAACTCCGCTCGCTGGGCCCGGGCTCAGACTGTGACACCCTGTCACTCAGGGTTCTCAAATGCCAAACCAAGAACCATCATTTTATGTGAACCAAAGTAAGCATGAGCATCAAAACAACTTAAGTAATAAAGAATTCACCAAGTAAATACTTAAAAGTAACATGATCAAAACAATTGAGTCTTTGAAAAGATAAAAATGTGCAACCTAATTAAGAACCCTAAGTGAGCCCCATGAGCAAAATTCAAGAAAATAAGAAAAGGGAATGAAAGTTTAAAATTATGACTTGAGCCAATTATGAAAATTAAAGAATATTTAATGGGCAACAAGAAATGTTGAGAAAGCTTAGCCAAAATATCAATAAAACCCAAATCAAGCTCCTCATGTGGGACTCATTAGGATTTCTGAATTCTGGATTCAAATTCAGACCTTGAGCCAAAGATCAGGATGTTCACCTTGATCCCTAACTTGAATCCTAATGGCCCATTGACAAAATTGTGTCTAACTAACCCTCCGTCAGTGTGCCGGAAGATGGCATTGGGATGAGCCCTAGACACGACAAAACCTTGATTTGCCTCGGGTTTGGGCGAGGGAGTGAACTGATTTCTGCTCCATATCTCGCAACTTGAGGGCAAAAACCTTTACCCCACAGAATGGTAGCTTGTAGGGAGAGAGAGGTTTTGTGCAGTGACCAAGCGAGGCGAGCTGCGGATGGCGACCACACGCGAGAACTTGGCGAGCGCTTGCGGGCACCGCGTGTTCGACCACGCTCGGCCGCCGAGCTTCGCCTAAGCTCGCTCACCCGGCGTCGGTCAAGTCCTCGCGGCGCCCGCCCCTGTAGTGCCGCCGCGCTTCTCGCCTCCGGCGCACCTCTCTTCGCTCACTCACCCTCTCGCCCGACCACCTTTCCTTGCTCCGTGAGCTTAATTCGCTTCGCCATTGCCACGAGCCCAGCCGCCACATGGCCACCCTCGGTACCCTCAAGCCCACCCGATTGCCGGCTTAGCTCCGCTAGTGCTGTGAAGCTTGCCAAGCCCTGGACGGCAGGACTTCACCGGAGGCAGAGATCGACCTCACCGGACTTGGTCTTCACTTGTGGACCGAGCTATCCGGCTGAGTCTCCGCCCAATTCCTTTCGCTCATATCTTCTACATCCGTGAACCTCCACGTATCTAATTGAACTATCTCGCTGGTGACAAGGCCGGTCTCCTCGCCGTGACGAGCATCCCGCCGCGGCGTGGGTGGACCGACTGGGCCATCTCCGTGGTGTTCGCACACGTTGATCCGAGACCTCCCCTTCGTCCTCGACCACTTCAGGAGCAACCTCGCGCAGAGTGCCTCTTAGCCCTTTTCTCCGCCCGTGACTCTTGTTTAAGGTAGAAGAAGGACCTCTGTTAGGATTGGTAGGGCAGGGTTTTTCTAATGTCGTGACTCATGTGAATAGTAACCTAGGGACGGTCGCGAGAGAGGCAAAATCGAGGACCTCGTGCAAAGTGGATTTCCATTTAATCAATTCTATTATTCTTTTAAATGACCAAAGAACTTAGAAAACCCATAACATGATGAAATCTTAATAAAAGTTGTCAAACCAATTTTGCTAGCTCACGAATATTATGATCTATCATTTAAAAATAGTAAACTCCATGCTTTTCTGTTCAAAATTTTAAAGTTTAAAATTAAAGACGAAACCCACTAAACCTTGTTTAATTAAGGAAAATTAGTTTTTCTTAGGTCTGAACAAGAAAAATTGTAGATGTTTAAACCTTATTTAGACACTATTTAAAAAATAGGAACTGACATTGAAAAATATGATGTAGTTTTTCATTTAAAGCTAATTTGTCCAAAACTTAGAAAAATCAGAAAGGCCTTAGAAATTAATGAGCAGTGATTAATAATATTTTCCTAGTTTACTTATGCAACAGAGAACCTAGGAAAATCTGAGACCATTAATTTGGACCGTTTTAAATTAAGATGATTTGTTTAGCCTCATATAGACTGAAAATCAATTATTAGAGTTACAAACTATAACCAAAATGATTAATAAAAATCTAGTGGACTTATAACCACGAGCCTCACTACAAAAATCTGAGCACCTCAACCCAACTTTTTAAGTAGAAAATAAATACAAGGTGATAATAAGGCATTTTCCAAATAAATCATGAACAACCTTTTAATGTGATAATAGGCAACCAAAATTTGCTAAGCTCATGAGAGGTAAACTACCCAGGAGAAAATGCAAACCCATGAAGAAGAAGTAAACCCATACCTTTGCTAATAATTAAGAAAGACCACTTAATTCAAATAATGCAAACCACCCCTTCCCTTAAGCAAGAAGCCAAACGAATGATTGCTCTTGCACAAAATACTAACTATGAAAAATAAGAACTCATTGTTTGATATTTTCAAATATAGTAGTAGAGCACCCATTTGGCTAGAAACTTGAGAAAACCATAGAAAATTTATTAAGAGGTATTAATGATTAGAAATATCAAGTCATGTTATAACACCTAAAAGCCAGCAAAATAAGTTTTAGAGAATTACCCTTACATTAAATAGTAGTTGTAGTTCAAAGCACATACCTTGAGTTTAATAATTTTGTCGGAGAAAACTATTCACTTTCTGACCCCCAAATTTTGAGACAGGAAACATACACCAGATAACAAGCCACTGTAATTTTTGTGAATTTTAGAATTTTATAAAGCAACTTAGTTCAAACCTACTCAAAACATTAAAGAGAATAAAGAAAAGAGAAGAAGGAATAAACTCTCATCCCAATAAGTCTAATTTGAGAACTTATCATTTATCCCTAAGACTTAAAAGGAATTCATGGAACCCCAAAAAATAAACCTACCACTTACCTTAGCTAAGTCTAACCCAATTTACCAAGTATACCACTAAGGGTTTACATAAGTAAAGTTAACTATTTTAAACTCAAAAGATCCTACACTTTAAAAGTTGTAATTTCTAAAAGCACATATCATATCATGCATATATCTTACATGTGCATTCATTAGATTGTAATCTTGCCGGAGAGTACTTGCTCATCCCGAGCAAGGACTCCCGAAGAGGAGACCAGATGTTTTGGAGGTCTGTATTGAGGATCTCCCCCCTTGCCCCAACAATTGAAGGCAAGCCCGGTTTATGCATAACCATGTTATTATATGCTACTTTACTACACTTAATGCTTGTAGGATTGCAATGTGCCTTAAGTGTAGGAGTTGCTTGAAACCTAGTTGCATGAACTTAGGATTCCTTTTGAGATGAATACTAGTATGCTAGGTCGAGTAGTCGCCATAATCGTGATCTCGGTAGAAGTCGAGTGATTTTTCTAGCACTGCACAGAGGTCGAGAATTGATTGTATTCATCTTGATAACGGATCTATGTTGGTCTATGGATTTGGATCCAGGAGGATGCCTTGTCCATGAGACGGGAAAAATGAATTAAGGATTAATGTGGATACCAGTCAAGCTTTGAGCATTATAAGCATGCCGGAAAATGGTAACCGTAAACCTAGTACGAGTGAAGCCAGGAGCGCGACTTTATCCTCATGCGACTCGAGACTGGTCTCCCATGCTAGCTATGGTGGGTACAAGTGCGGTCAACGCAGCGTGGCCGGGTTAGTGGAGCATTGTATGCCAAGCGATGAGGCTGGGCGAGCGAATCGATGGGGACGGTTGTCATGCGCGGGAGTAATTCCGCGACATCGTGTGTTTAGGTTTACCTTGCAGAGTTTTAAAACTCGATTCAGATAAATGCGCTTCTCGGCTAATGAGATGCTTGATTCCTTATCTTGCATCGAGTAAGAAGTGAAATGGGACTACATGAGATAACTTGTTGATTGAACTAATTGATTGTTACCATGTATGCTTAGAAAGAGCAAATCTAGCTAAGTTAATGATGGTAGAAATTGAAAAGCTAAAAATTGATTTTAGAAACAGACTAGTGCTTTTGGCAAACCAAACCCTCGACCAACAAATGCATAGTCTAGAGGTAGAGGTAGACTCCTCACCAGGTTAAGTCTAGCCGAGTAGTATACTATCAGTACCTGCTTGTGCAGCACAATTTTTGCGGTACCATGGGAATTGGTTGATGGTGTGACTTGGCCTCTCACCACCACTCGGGTTGGGCCCTGAGTGGATGTTGCTCCTAAGGAGAGACATGAGGAGTAGTGGGCTGAGCCTTGCCCATTCCTCATTACCCATTGTCGATTATCGTACACTTTATTTTGTGAACTTTATTTGCTACTCAAAAACTCCGATTTATGTAATAACTCGGTGCAATTTGAGGTTTCTGTTTTGTATTTCTTCTGTGACTCACCTTCGAGTGGGATTGTGGAATTTGATCCTCGTTAAGTGGCTCTATCAGACTAGATATGAGGGATCGAGATTATTCGATTTAAGTGTGTTCACGACTTCTGGCGTGACTTGCACATAAAGCTGAATAATTAGGGTGGTTACACCTGGTGAGCGCCGGAGCAAATTCCACCACCAGGAAGGATAATAAACCATGAATACCAATTTTCAAAATCTAAAACTTGCCTAGAAATCTGGATCGACAGACTAGACCGCTAGACCTGGGACGAAAGGCCTTAGGCATAGGGAGAAATAGGTGGCTAATTAATTAGGCCACAACCAGATATATATTTTACAGGATGCCTAAAAACCCTATCTTTCTTTGAGAGGCGCGCTTTCTTCGGCATGCATGCATTATAAAACCCCAAAGAGGAATTAAAATTTGAGCTAACCCCTTTCTTTAAAACCATCCGGTTCTCTTTTTCTTTTCCTTCCACCATCATCTTTATTCAGTTCCCTATGAATTCACCCACTGCCAAAGGGGAGACTCGCTTGACTCGATTTCTTCGCTCTGTGATGGCTTTCCTTCCATCTTGTGGGAAGTGTAACCGCCGGTTACCCTCGCGCTGTACGATGCGGTTGTATGAGGCATCGGGTACCTTGTTGTCCGTCCGGCGACTTTGGAGGCTCATCCCCGATTTGGCGTTCTCTTGACTCACAGGCGATTGGACCGAGGGCTGGGCGACAGTTGAGGCTGCCCATGAAGACTCGACAACTTTCGTGGCTACCATCCCGGAGATGGTGATGCACCTGGGACTCTTCCTGGCGAGAAGAAGGATGATCCCATGGTGTGTAAAGCGTGAGCCATGAAGGATGTGTGGGCAATGTATCCACGACTTGGTTGGGAGGTCCTTTGTTCGTGCATGAGTGCATTTAGACCGCCTTCGAGCCCTTGGAGCGATGCTATGACACTTCTTGCTAACACCGCTCAGTTTGCCAAGCTCACTCTCGGCGATGAAGATTTTGTGGTCGTATCCACGAGTTGGTGGAGAAGGATCTGCAGTGGAAAGGTGAGCCAGTACGTATACCACCCTGGAGCGACAAGTGGAGATCCGAGACAACACAATTGATGTCTTGGAGAACCACTTCACGATGTGCGAGGAACTCGACCAATGGCCACGGACATGCACCACACGAGATGGAGGCCAATGAAGTAGGAAGCTTAGGAGAAGAGGCCCGAGGAGCTAGGACCACCGTGCCAATGGGACTACGCGATGCCTCCTTCACCGTATCGATCGTCGCTTCTACCGCTCGAGGTTAAGCGGTCGCTTTGACATTTTAGGCGGATAGAAACCTATCGAGCTTAGTAGTATCACATTTTGGACTAGGCTTATGGTACTCCCTGATTGATGTAACCCTATAAACTTTGAAATCTGGGGATCTTTGTCACCATGTTATCTTCATTTCGAACCTAATATTATGATTATGGCATTTTTCCTTCCATATGAGATGATATCTTGTCGCTCAAACGTGAGTTAGGATAACAATGGCAACAATCTCTGCCGAATGGCGACCAGGCGATGGC

>nano5

AAAGATTTCTCCGTAGAGTTTATCTAGAAGTAAAATAGCCTTTACATATTCATCTTTCATCCTCACTTAGCTATATCTTACTTCTCTCAAGAACTTGTGAAATACTTTTCGAGTAGATTTTAGTCTAAAGTTTATAAACAGTTGGATTGGTTGAGTGGTTCAACTTATGACGCGTAGTCCCCCATGGAACCTACACGATCAGCCATGTATCTAACAATCTTTAAAGTTTGCTTGTGAAAATTTTCAGATTAGCCTATTCACCTCTAGGCTACTTTCAATTGGTATCAAAGTCAGCACTGTTTAACCAACCACGTGAGTAAACGTCATGTCTACTCAACGGGACCATGTGGATCCTCTCCTCGAGAAATCCCATCACATCTTCGGTGAGGAAGTGGACCCCAAGTCCTCGACCTCGCCATGAAGATTGCCGAGAGAATGTTCTCAAAATGAAAGAGGAAGATGCTAAGAAAAAGGCCAAGAAGAATCAAGAAGAAAGGCGAAGAAGATAAAGGTAAGGAACATTTGATTATAATGACGATCTAGTGGATCTTTTGGTGTCTAAGGTATTGAGCAAGGTAAGTCTCAACAAAGGATCATCTACCAAAAGCAAAGGTAATGAATTTAGCAAAGTTCAATTTGATTACTCTAGAAATTATATTCCCAACTTCTCTTCCGCCCCACTTGAAAGTTACCAACTCTTAGTGAGTTGAACTATGATGAGTGGTAGAACAAGATGAAGTCGCATTTAATCAGTGTGCATCCTAGTCTTTGGGAGATTGTTAATGTAGGTGTATATAAGTCCGCCCAAGGAAGAGATGACTCGGAAATGATGCAAGAGGTTCATCAAAATGCTCAAGCAGTGAGAGCATAATTAAAGGAAGTCTTTGTCCGTAAGAATACCTGGAAAGTTCAATGAAGAGAAGATGCCCGTGACATTTGGAATATTCTTAAAATGTCACATGAAGGAGATCCCAAAGCTAAAAGACATAGAGTTGAAGCTTTGGAAAGTGAGCTTGTAAGATATGATTGGACAAAGGGTGAGTCGCTTCAATCACTCTTTGACGGCTGATGGTGTTGGTCAACAAAATAAGAGTTCTTGGGAGTGAAGATTGGAGTGACTCCAAGGTCACAAGATTGTTCATGAGAGCTTATAAAGCAAAGGATAAGAGTCTTGCAAGGATGATTAGGATCGTGATGACTATGAGGGGATATAGCCTCATCAATTATTTGCAAAGATTCAACAATCCCAGAAGAAGCCTATCAAGTCAGGACTCTCATGCCTTGATCACTAATGAACAAGACAGAAGAGCAGAGACCACAAAGCAAAGAAAGTGGTCGCGACCTCAAGTGATGAATAGCTCAGTGATGAAGACACAAATATGTTCATCAAAACATTCAAGAAATTTGTAAGGAAAATGACAAGTTTCAAAGGAAAGGAAAGAAGAGGGCATGCTATATGAATGTGGCCAAACAGGGCCATTTCATAGCGGATTGTCCTAACAAGATAGAACAAGAAGCTAGAAAGGAATACAAGAAGGACAAGTTCAAAAGAGGCAAGACCAAGGACACTTCAAGAAGAAGAAATATGGTCAAGCCTATATTGGTGAAGAATGGAACTCAGATGAAGAGTTCTAGCTCAGGAAGAGGAAGTGGTGGCAAGCGTGGCCATCCAATCTACATCAAGCTCATAACTCGTCACAAACTACAAGGCGTCCTACACTCCAACTTACTCATGGCAAAAGGAGATAAGGTAACCTTATTTAGTAATGATTTTCCAAATGATGATGATGATGAACAAATTGCCATGAAAAATAAAATGATTAAAGAATTTGGCTTGAATGGATACAATGTTATCACCAAATTAATGGAGAGCTAGATAAAGAAAAGCAACTCTTGATGCTCAAGAAGACTTGCTTATCCTTGAAAAGGAAAGAAACCTAGAGCTTCAAGATGCTTCACAATAAAGATGAAATGCTAGATATCTTGACTAAGGAAGTATCTTTAGTCAAGATAACTATAGAGAATAAAGATAAAGAAGTAATTAATATGAAAACCTCTAAGCTAATCTTGCAAATGAAAAGAATGCACTTGGCTAAGCATGTTAAGCTTGAATGTTCAAAATCAAGAACTTCAAGTGCAACTTGAAAATTGCAAGAACATCAATGCCTCATCTTAGTGCTTGAATCTAAGTCTAGCTTAAATGATAATTCTTGCAAACATTGTGCCAAATATCATGCTTCTTGTTGTCTAACTAACCATGCAAGGAAGAATAGCCCATGGTGAAGGTCAAAGAAATTTTGAAAAGATGCTCTAGCAATGATGGGTTAAAGTTGAACCCAAGTACAAGTCCTTAAGCCCAACAATGGAAGAAGGGGGCAGGTTCAACTCATCCAAGCCCTAGCCTGAGTGCATAAGGGTGAGATCCCCAAGTTCATAGAGGAACCACCCTATATGATGCCTTGGGGAGGATTCACTCCTCAAATGACAAGTCACCTCAGTAAGGTTAACTTGAGTTCCACAAAGAGTAAGATGAAGGAAGGGATCCTCAAGTGGACAAAAATCTAATGCTCCCATTTCCCACTCATATCTTTGTGATTATATGTTGACTTGGGATTTAGGAAAATTGGTTGTGAAATATGTGGTGCCTACACTAACGAAAAGTCATGAAAAGAAGTGTGTGGGTACCCAAGGCTATAACTAACAGTAGGACCCAATTCAATTGGGTACCTAAAAGCATAGCCTAAACTTGTTTTGCGGTCTACTCCTCACGTGGGTCAAGTTGGGTGCTTGACGGTGGATGTACAAATCACATGACCGGAGAAAGACATAGCTTCATACATTACAACTAACTCAAGAAGCACAAGAAATTGTGTTTGGAGATAGTGGCAAGAGTAAGGTGATTGGTATTGGTAAAATTCCTATCTCGACCACAATCACTTTCAAATGTTTTATTGGTAGATTCTTTAAGCTATAATTTGTTGTCCGTTCACAACTTTGTGGAATGGGTTATAATTGTTTATTTTGGATGTGGATGTGAAGATCCTTAGAAGGGAGGACTCTCGATTGCCTTTACCTTCGCTTGAAGGGCAAGCTTTATCTTGTTGATTTCACAACAAGTAAAGTAGACTTTGAGAGAACTTTTAGTGGCAAAGTCCGACAAGGGTGGCTATGGCATGTACGGCCAGCCCATGTCGGTATGAGAATTTGGCCAAACTTCAAAAGGATAATCATATCATTGGACTAACAAATGTTGTATTTGAGAAAGATAGGGTTTGTAGCGCATGCAAGCGGGAAACAACATGAGTCCCACATCAATCAAAGAATGTGGTCACAACAAAGAGGCCATTGGAGCTTCTTCACATGGACCTCTTTGGACTGTGGCCTACATTAGCATTGGTGGTAGTAAGTATGGTTTAGTCATTGTTGATGATTTTTCTCGATTCACACAGGTTTTCTTTTTAAGTGATAAAGGTGAAACTCAAGAAATATTGAAGAAATTCATGAGGAGAGCTCAAAATGAATTTGAGCTCAAAATCAAGAAAGTGAGAAGTGATAATGGGACCGAATTCAAGAACAAGTGTCGAAGAATTCTTAGGAGAAGAGGGAATCAAGCATGAGTTCTCGGTGCCTTACACTCCACAACAAAATGGTGTTGTGGAAAGAAAGAACCGAACTCTAATTGAAGCCGCAAGAACCATGTTGGATGAGTACAAGACACCGACAACTTTGGCGGCAGTCAACCACCGCCTATCATGCAATCAACCGTCTCTATCTTCATAAGATCTACAAAAAGACCTTAGGCTTCTCACCGTAACAAACCTAAAGTTGATTATTTTAGAGTATTTGGTTGTAAATGTTTTATTCTTAACAAGAAAGTCAAGAGCTCAAAGTTTGCTCCTAGAGTGGACGAGGGCTTCTTGCTTGGTTATGCATCAAATGCGCATGGATATCGTGTTTTCAACAATACCACGGCCTTGTTGAAATAGCGATAGACGTGACATTTGATGAGTCTAATGGTTCATAAGGGCATGTTTCTAATGTCGCTGCGGAAATGAAGAACTACCTTGTGAAGCCATAAAGAAACTTGCAATAGGTGAAGTGAGACCTCAAGAAAGGATGATGAGGAAGGAACTTTCGGATGACCAATGAGGTTGTTGATGTGGGTGCAAAGGTGGTGAGTGACAAAGTCTCCACCCAAGCAAACCCATCAACCTCAAGTCATCAAGCCATGAAGAAAATCATCAAAGGATGCCAAGTGGGCAGAAACAACACAACATGATGGTGAAGTACCTCTTGATCAAGTGAATGATGAGGAGGAGCAATATAAAACAACCATCGTGCCTCATCCAAGAGTCCATCATACAATTCAAAGGAATCATCCGTGGACAACATCCCGGGTAGCATCAGAGATAACGACTCATCCGTTTAGCTAAATTTGTGAATTTTACTCGTTTCCTCTCTTGAGCCACTTAAGGTTGAAGAAGCATTGGGTGATCCGATTGGATAATTGCCATGCAAGAGAGTTAAACAACTTCACCCGGAATGAAGTCTGGTCCTTAGTCCAAAGACCCAAACAAAATGTGATTGGGACTAAATGGGTCTTTAGGAACAAACAAGATGAACATGGTGCGATTACAAGAAACAAGGCACGATTGGTTGCCCAAGGCTATACTCAAGTGGAAGGACTTGATTTTGGCGAAACATATGCGCCGAGCAAGGTTAGAATCAATTAGAATATTAATTGCATATGCTACTAACCATGATTTCAAGCTATACCAAATGGATGTCAGAGCACATTTCTAAATGGACCACTACAAGAGAGAGTATATGGAGCAACCTCCGGGTTTGAAGATCCAAAGAAGCCAAATCATGTTTATCTACTTCACAAGGCACTCTACGGGCTTAAACAAGCCCCTAGAGCTTGGTATGACTGTCTTAAGAATTTTTAATTAAGAATGGGTTTACAATAGGAAAAGCCGACTCTACCTTATTTACTAGAAAAGTTGACAATGAATTATTTGTGTGCCAAGTATATGTTGATGACATTATATTTGGTAGTACTAATGAAAAATTTTGTGAAGAGTTTAGCAAAGTAATGTGAGCGTGTTTGAGATGTCTATGATGGGCGAGCTTAAATACTTCTGGGATTTCAAGTCAAACAACTCAAGGAAGGTACTTTTCTATGCCAAACCAAATATACACAAGATATGCTCAAGAAATTTGGCATGGAAAAGGCAAACACGCCAAGACTCAATGTCATCAAATGGACATCTCTACTAAATGAGGAAGGTAAACAGTAGATCAAACATTATATAGATCAATGATAGGATCACTAGTTTACTTATGTGCATCTAGACACGATATTATGTTGAGTGTTTGCATGTGTGCGTTTTCAAGCAAATCCTAAAGATTGCCATCTTGTATGCGATTAAGAGAATTCTAAGATACTTAGTTCACACCCAAAACCTAGGATTATGGTATCCCAAAGGCTCCTTTCGATTTACTTGGCTATTCGACTAGATTATGCGGTTGCAAAGTAGATCGAAAAGCACTCTCGGGACTTACCAATTCCTTGGGCGATCCTTAGTATTATGGAGTTCCAGAAACAAAATTGTGTTGCACTTTCCCTAGGTAGAAGTACATACTTGTTCAGGGCATGTTGTACAGGATTGTTATGGATGAAGTAAACCCTTAGAGATTTTGGTTGTGAGTTTAACAAAATTCCACTTTTGTGTGACAATGAGAGTGCCATAAAACTTGCAAACAACCGTGCAACACTCTAGAACTAAACATATTGACATCGCAGACACCATTTCTTGAGAGACCACGAAGCCAAAGGAGATATCGAATTGTTTCATGTGAGCAAGAAAATCAACTGGTCGATATCTTCACAAAACCTCGATCGGACTAGGTTTTGTTTTCTTAGAGTGAATTAAATATCTTGGATTCTCGTAACTTAACTTGAAAACGGTCACAAAATTGTTTGATTCACTATTTTTGATTGATAGTTTAAAGCTATGTGATGATCTTTCAAAAACTTGGTGAAAATGTTAAAATCTGAATGCATTTAGTGCTAAGATTCTTTTTGGGCTCAATGGCTTGACCGGCTGAACTTTCTAGTTCATTTGGAGGACGGTGAACAGGAGAAACTTCAACCGGCTGTTTGTTTAACCGTTTTTACGGGACCATCCTAGTTAAACCGGGTTGAACCCAGAGCATGGTGGACCTGGTTGAACTGGTCTCTGCCGGTCTCGGCCAGCAAGAACACATTCACGCAGTCTCACCGCAATGATGTCGACGCATGAGATTTTTAACCGGTCACATCCTGGTAGGTGACATCTTTTCTCTATTGACCGTCGCTTCCACTTTTCTGACACTGGCGTGAAATTTTTTGCAGTCGCAGTCATCAATTTGACTTTTTCGGGTCGCGCAACAATATTTTTACGGTCGCTCTCTCTTCTTTGGTGGCTGGCGTGCAGATTTTTTCTCTCTCTTTCCTGTCCGGTCACTTCCCTCTGTCTGGCGCAGATTTATTTTTTCTTTTAAGCTTGCTCGCTGGCTCGGTGCCGCGTATCGCCACTCTGTCCGATGCTTCTTGTCGTGGCGCCGAACTTTTTAGGGCATCGGTCTTAGCTTTGCATGCAAATGTCGGGCGCCATGCGATATCCGGCGTGGTTCTAGCGTCAATGCGGCATGATTTTGTGGTGTCGTAAGCAGTGCTGCATCGTGAATTTTTTATTTGTTCTGTCGGTCACTTCTCCTCATTGGCGCGACGTTTTTCTTGGCGTTTACCGCTCACCGCGACCATGCCGCAGATTCTTTCTCTCCTGCTCCACCATGCATGCGTGTCGTGGCGCCTAATTTTTCTTTCCTCATCTGGTCCTTCCGTGATGCGCGCACACAGCTTTTTAGCCCCACACTGCAGGTAATGCGTAGCCTTTGCTCTTGTGACGATCCGTGTGTCACCCAGCGGCAAGGAAATATTTTTGTCACTTCCGCACAGCGTGTGAGGATACAGGCGCCGCAGAACTTTTCTTTGCGCAGTTCTTTTCTTTTGCCGTCCGGTCTCATCTGCAAACACTTGGTTTTGATTTTTTTTCATAGAATCTCATGATACACTTTTGTAATCTGCGTGAAATTTGATTTTTGGCATCTTCCTCTGCTTTGTTTCGGTTCTCACCACCTTTGTTAGAACCAAATGAATCCCAGCCCATGGGGCGTTGAACTGTCTGAGGACGGCCAAGCTACTATATATATGACTCTCCTTCTCTTCTCCCACTTCATTCACTCTTGCCTTAGTCGCTTCACAACCAGAGCCACTCTTTCACACACCTCAACCCGGTTCACGGTTCACCACCTTTTTCAATCATGGTGCGTGTCGCAACCCTCAGTGATTGAGTTTAGCAGCGACCATGAGATTCGGAGGAATCTCCCATCCTCTCCCTCCCTCGCCGCTCCCTCTCTAGAGAGGACGTGGCTCGTCCGGATGGATGGTGAGGCTCTTCCATGCCCTCGCAGCCGATGACCGTGAGACGCCAAAAGGTTGGCGCCTCATCCTCGTCGTAGCACGCCTTCCTCTGGTATGCTCCTGTCGGGGAGGATGGGGCGTTCGACGACCGCTGACCTTCGGCGCTGATGCTCTCTACGTCCACGGTCGCACATGCATCCGTGAACGTCGACCGTGATCCGCATGAATCTCCGTCGACTTCACTCACGGAGGAATAGACACCTTCAGCGCTGAGGTTCACCAACCCTACTCTCCTCCTCGTCATCCCGGTGTCCAGGACCCTGGTTCTGGTCTTTTCAGCCGACTTCTACAATGTCATTCTTTCCAAGAAACACCCACCGTCCGACATAGATTTATGGAGGAGGGTTGTGAGAATATGGGAATGCGACATGTCGTCACTCGTCGTAAACAGAAAGTTCACGAACATTATGACCATGGAATACCCCGAACGATGAGGTGGTAGCTCGCCTATGCCACCCTCACGGATAAAGAAAGTAGATGAGAAGCCGATGAGTATGATTACCCGGTCATGTATTTCTTCATCCGGGTAATCACAAAGTCGATTATCGTCGTTTTGCCCATATTCGGCTTCTCGATGCAGATATCAGGCGGCAACATGCGAGTGCATGACATTCGGCGCCCATGCGGAAGAGACAGAATTCATTCATGTCTCTACAGTAGGAGTTGGACAACAACTAACATGCATAGGTACTACAGGTACCTTAATTCTTTGTCCCGGATGACCGTTCTCAGAAGAGCGGAAATCAAATAAACGCCCTTGGGAGAGTCGATCTTCCTCCTCCTCCTTACTCCAACAATCTCGCATCAATGTGTTTGACATGATGCAGGAAGAGATCGTCTGCTTCTGGTCTCCTCTCGGAGGGTGTCTTGCACCCTTTATCATGAAGATGATCAAGTGGTCACCTGCACCCACTTCAAAAACGTCCAAACACTCTCGCTTCGTGCCCTCTACCGGGTTGATTCCTCCAACCCACCGCCGTCTGGCGGGCCCCTCTGATGCGGGCCCTGCCTCCTCCACCAGCTACACCGCCCGGCCTCCCCATCATCCATCCTCCTCCCTCCCTAGCGCCTCGCGTCCTCTCTCGGCATGGATCTCCCATGCCACGTGCTCGTGGTCATGGTCGAGTCGGGCCGTGGGATGGGTGCTCGCTTGGTCCATGGGTTTGCGGCATTTTCTCCATGTGACGCAACATTTTGCCGATGTCCATGAGGTGGCACGACGTCGGCCCGGGAGGCCGACGACAATCTTCGCCGTCAAGCTTCCTCCATGGGCATGCCTTTTGTCCCTCGTTCGCGATGTGCCTCTCCATCCTCCTCCCGGAGATCAATGAGTGGCACCCAGCCAGTCTGGGGTGCCTTTATGTGCAGTAGACGATGAAGATGAAGAAGTCTATGTTGATGATCGCGAGGTTTGCCCCTCCCTACCATGGGGATCCGGTCAATCATCCTCCCACCTCCGCCTCCTTACGGGTCCGGGACCCATTCTGTGACCCTAGGCCATCCGCCTCGTTACCACCACCACCTCCACCTCCTCCCGATCATCGTTACATCTTGATCTGCTTTTAGGGTGATGAGCCACCTCTGACTCCACTTTTGAGGAGGACGCGATGAGACTTTTTGACTACACTTCATATCCTCCGTCATATCCTCCTCCTGGAGGATATTGATTCTGTATCCTTGTCTCTCTCGTTTTGGTACTTGTTGCCAAGGGAGAAATCCAACTTCTAGTTTGTGGTTCTTCGTTTTCTTTTGGATTATTTTTGTTTTTAGCCACCTTTGTGTGGCTTGTACCCTTACCATTGTATGAAATAAAACGTGGATTGTTATTCAAAGTAATATGATGGTCTTGCCATCATCTTTTCCTCCTCGCTTTGTAATGTCTACTCGCTATGAAGTAAAAATAATGGTTTTTAGAGATAGTGTGTACATCACAACTCTGTCATGACACTCTTGCACCCTCGGATAATTGTATTTAGTATGGTTGTTGTCACTATCTCTAAATGTGTTGCTCACATGACATATTATGTCAAGACGTTGGATTCACAATCGTGCACACATTTAGGGGAGCCATCTACATACAATCATTCAAAAGAAATTTTCTATTGCAAATATATATCTTTTGACCTTAATTGTTTTGGCATCAATCACCAAAAAGGAGGTTGTAAGTGCAATCAACCCCAAGTAGGGTTTTGGTGATTTAATGACAAAACAAATAAGAGTACTAACAATTTTGTTCTCATGTTTGATTAGAAGGAAACAGAACTTAAGGAAGCACCAAGGACTTCATGCATGACATTATAAAATTTATGTAAAATCTTGTGTTGCATGATGTAATTCTTCCTTGTTCTTTTCCTCGCAGTGCAGTGTTTGCTATAGCTCAAGTCCTCTAATTCAAAAGCTATTTTTGAAAACCAAAAATCTCTTTAACATTATTTGGTGACCATGGTTTGGGTTGAGAAACCTAGAGTGTTCTTCTTGAAAACAATGAACTTCTTCAATCCAGAGTGAGCTTTCGGTGAACTTAACTTCGGTTGTGATGAGCTTCACCGGTGCGGTGAGCTTTGGTCAAATTTGGACTTGGTGAGTTGAACTTTCAACTCGGTGAACTTCAACTTCACCATGGACCTCTCGACCATACCGGTGAACTTGCCCTGGGCGGTGAGCTGGGTTTCTCTCCAAAACTCTGCGGTGAACCAATCGACCTGTCTGGGGTAGCTCAACTGGTCTCGACCCTCGACCTCGATGCGATGCCGGTCGATCGCGGTCAGTGCGCCGGGGGTGTCGGTAGTTCAACGCCGCCTAGGGGTGCTTCAACCGATCCCGGCTCGTCGACCGCGCCGTCGCTGCGGTCGATCACGCGCCCGGTCAGGTGAGGCGATCTGCGGACACCGGCGGTTCAAAGCCCTAGGGGCTGTTCACTTGTTTTACGGGGTTTTTGGTCAACGGCTGGTTTTGAGCCGTGACTATAAATAGACCCTCTCTCTTCTTCACAGTGAACACTCACTCATTTATTGCTCACCTAACTTGAGACAACATCTGCTCTCCTCATCTCTCACACTCTCTCCTCAACATTAAATCTTCCTCAAGATTCAACCTTGTTGGAGCTCTTGGGTGTGAGGTTTGTGCTTGTGCTCACGATTTGGTTTTCCTCTCCCATCTCTTTTTCAAAAAGACTTGAGCTTGTGCAAACCCCTCTTATGCGTTCTTGGTGTTCTTGAAACCCTAGGTTTCAAGATCTTCGAGGTTACTTGGGAGTCTCCAAATTTGTGGACGACCTAAGAAGTTGGTATCACCCGCTCTTTGAGCTAAGATAAGAAGAGATTGCCTTGACCTTTGTGGTCGGCTTTGGAGGATTAGGGTTGAAAAAGACGACCCTTTGTGGGCTCCTCAACGGGAGTAGGACACCTTGTGGTGTGGCCAGAACCTCGGATTAAATCTTGTGTCTTGTGTTCTTACTTGTTTCAACTTGAGATATTTTTACTTGCAAGATTGACATAAAGATTTCTCGTAGAGTTTATCTAGAAGTAAAATAGCCTTTACATATTCATCTTTCATCCTCACTTAGCTATATCTTACTTCTCTCAAGAACTTGTAAATACTTTTAGTAGATTTTAGTCTAAAGTTTATAAAGCAGATTGGATTGGTTCGAGTGGTTCAACTTATAACGTAGCTGAACCGCTTGCACCCGTCGAACTATGTATATAACAATCTTCAAGTTTGTTGTGAAACAGATTAGCCTATTCACCCCCTCTAGCTACTTTCACCTACCTGGTCCGACAACAAGCCCATCACCTTCGACCAAGCCACACCACCGACCACGTCTGAGCCCGGGAAATACCCTTCGTTGTCGACCGAGTCATCCGGTGACGCCGGGCTCACCAAGGTCCTCATGGACGAGGCGCACCTCAACATCATCTATGCCGAGACCCTCGCCTCTGCGTGTTGATCTATCCTCGATCCGGGCGGCGCGCCATTCCACGAGATCATCCCGGAAGCGCGTCCAACCCTCTGGACAACTCGACCTTCCGTGCCTCGAACACCCTCCAACTTCGAAGGGAGACCCTCACGTTCGAGGTGGTCGGGTTCGAGGAACCTACCACGCAAGATCGGGGGCCATGCTACGCCGTTCATGGCCGTCCCCAACTACACCTACCTCAAGCTCAAGATGGCCCCCCAGCGAATCATCATCACCGTCAACGTACAAACACGCTGCTTGAATGCGGACGGAGTGCGTGGAGTGCGCCGAGGCCTCGGAATCCAAGGCCCTCATCGCCGACTGGAAAGCCTCTCTGAGGAGGTGCCAGCGTGAAGCGCCACGCCAACTAACGAGCCACGAGACGGTTAAGTCCGTCCCCTCGACCCCGTTGGCCAGCGCCTCAGCGGATCCGGATCGGCTCGAGCTCGATCCCAAATAGGAAGTGCTCGCCGACTTTCTCCGCAGCAACGCCGACGCGTGGAGTCCTCTGACATGCCCGGTATACGGGGATGTCGCCGAGCACTATTGGATATCCGAGTTGGAGCAGGCAAGTGAGCGACCTCTGCCGCATTCGATGAAGAAAAGCCTTGAGCCATAGGCGAGAGATCCACAAGCTAATGGCAGAGGTTCATCAAAGAGGTATTCCATCCCGAATGGCTTGCCAACCACTGTTCTTGTGAGAAAGAAAGGAGGGAAAGGCGGATGTGTGTAGACTACACTGTCTAAACAAAGCATGTCCGAAGGTTCCCTACCCTACCTCGCATCGATCAAATTGTGGATTCCACCGCTGGGTGCGAAACCTTGTCTTTCCTCGATGCCTACTCGGGGTACCACCAAATCGGGATGAAAGAATCCGAGCATCGCGACTTCTTTCATCACACCCTTCTGCATGTCCGCTATGTCACCATACCGTTCGGGCTGAGGAATGCGGGTGCGACGTACGGTGCATATGAACCATGTGTTCGGCGAACACATTGGCCGGACGGTCGAGGCCTACGCCGATGACATCGTAGTCAAGACGAGGAAAGCCTCGGACCTCCTTTCCGACCTTGAAGTGACATTCGATGTCTCAAGGCGAAAGACGGAAGCTTAATCCGAGAAGTGTGTCTTTGGGGTTCCTCGAGGCATGCTCTTGGGGTTCATTGTCCTCGAGTGGGGCATCGAGGCCAATCCGAGAAGATCGCGGCCATCACGGCATGGGGCCCATCAAGGACTTGAAGGGCATCGAGAGAGTCACGGATGTCTTGCGGCTCTGAGCCGCTTCATCTCACGCCTCGGCGAAAGGGCTGCCTCTATCGCCTCTTAAGGAAGGCCGAGTGCTTCACTTGGACCCCGAGGCCGAGAAGCCTCGGAACTCAAGGCGCTCTCACGAACGCGCCTATCTTGGTGCCCTGCTGGAGAAGCCCTATTGATCTACGCGCGCGACCACTCGGGTGCTTAGCGCCGCGATCGTGGTTGAGGAGAGGCGAGAGGGCATGCATTGCCCGCCCGAGAGGCAAGTCTACTTCATCGGTAATTGTCAAGACCAAGATCCGCTACCCACAAATTCGTAAGGCTGCAGAGCGGTGATCCGCAGCGGAAGTTGCGACACTACCGAGTCTCATCCGATGCGTGGTGTCATCCTTCCCCTGAGATCATCTAGTCCGAGAGGCCTCGGGTAGAATCGCAAGTGGGCGGTGGAAATCATGGGCGGCGATCTCGCTCGCTTCTGGAAGGCCATCAAGTCCTGTGTCTTGCGGACTGTGGTGAATGGGTCGACACCTGGCTCCGGTTCCGATCCAACCGGACTCGACCATGTTCTTCGACGGTGCCGATGAAGACAGGAGGCCGGCGAGCTCTCTTCATCTCGCCTCGGGAAGCACCTCGCTATGTGCTACGCCTCCACTTCCGGCCGAACAATGTGTAGTCGAGGCTCGTCGCGGTTGCGCATCGCCATCGGTCGAAGTCAGACCGCCTCGGCCGCTCGTGGTGACTCGCGACTCGTCATCGACCAAGTCGAAGAACCTCCCACCGCCGCGACCAAAGATGGAGGCTCATCTGCGATGACGTTCGGTGCGGAAGACAAGTTCTCACGGGCTCGAGAACCACACGCATCGCCGCTACAACGAACGACGATAGTTTAAACTGAAGGCGGGAACGACAATCTGATCAAGCTCAAGCTGCTCTAGCATTCGCCATTCAGGCTGCGCAACTGTTGGGAAGGGCGATCGGTGCGGGCCTCTTCGCTATTACGCCAGCTGGCGAAAGGGATGTGCTGCAAGGCGATTAAGTTGGGTAACGCCAGGTTTTCCCAGTCACGACGTTGTAAAACGACGGCCAGTGCCAAGCTTGCATGCCTGCAGTGCAGCGTGACCCGGTCGTGCCCTCTCTAGAGATAATGAGCATTGCATGTCTAAGTTATAAAAAATTACCACATATTTTTTGTCACACTTGTTTGAAGTGCAGTTTATCTATCTTTATACATATATTTAAACTTTACTCTACGAATAATATAATCTATAATACTACAATAATATCAGTGTTTTAGAATCATATAAATGAACAGCTAGACATGGTCTAAAGGACAATTGAGTATTTTGACAACAGGACTCTACAGTTTTATCTTTTTAGTGTGCATGTGTTCTCCTTTTTTTTTGCAAATAGCTTCACCTATATAATACTTCATCCATTTTATTAGTACATCCATTTAGGGTTTAGGGTTAATGGTTTTATAGACTAATTTTTAGTATATCTATTTTATTCTATTTTAGCCTCAAATTAAGAAAACTAAAACTCTATTTTAGTTTTTTATTTAATAATTTAGATATAAAATAGAATAAAATAAAGTGACTAAAAATTAAACAAATACCCTTTAAGAAATTAAAAACTAAGGAAACATTTTTCTTGTTTCGAGTAGATAATGCCAGCCTGTTAAACGCCGTCGACGAGTCTAACGGACACCAACCAGCGAACCAGCAGCGTCGCGTCGGGCCAAGCGAAGCAGACGGCACGGCATCTCTGTCGCTGCCTCTGGACCCCTCTCGAGAGTTCCGCTCCACCGTTGGACTTGCTCCGCTGTCGGCATCCAGAAATTGCGTGGCGGGCGGCAGACGTGAGCCGGCACGGCAGGCGGCCTCCTCCTCCTCTCACGGCACCGGCAGCTACGGGGATTCCTTTCCCACCGCTCCTTCGCTTTCCCTTCCTCGCCCGCCGTAATAAATAGACACCCCTCCACACCCTCTTTCCCCAACCTCGTGTTGTTCGGCGCACACACACACAACAGATCTCCCCAAATCCACCTGTCGGCACCTCCGCTTCAAGGTACGCCGCTCGTCCTCCCCTCTCTACCTTCTCTAGATCGGCGTTCCGGTCCATGGTTAGGCCCGGTAGTTCTACTTCTGTTCATGTTTGTGTTAGATCCGTGTTTGTGTTAGATCCGTGCTGCTAGCGTTCGTACACGGATGCGACCTGTACGTCAGACACGTTCTGATTGCTAACTTGCCAGTGTTTCTCTTTGGGGAATCCTGGGATGGCTCTAGCTGTTCCGCAGACGGGATCGATTTCATGATTTTTTGTTTCGTTGCATAGGGTTTGGTTTGCCTTTTCCTTTATTTCAATATATGCCGTGCACTTGTTTGTCGGGTCATCTTTTCATGCTTTTTTGTCTTGGTTGTGATGATGTGGTCTGGTTGGGCGGTCGTTCTAGATCGGAGTAAATTCTGTTTCAAACTACCTGGTGGATTTATTAATTTTGGATCTGTATGTGTGTGCCATACATATTCATAGTTACGAATTGAAGATGATGGATGGAAATATCGATCTAGGATAGGTATACATGTTGATGCGGGTTTTACTGATGCATATACAGAGATGCTTTTGTTCGCTTGGTTGTGATGATGTGGTGTGGTTGGGCGGTCGTTCATTCGTTCTAGATCGGAGTAGAATACTGTTTCAAACTACCTGGTGTATTTATTAATTTTGGAACTGTATGTGTGTGTCATACATCTTCATAGTTACGAGTTTAAGATGGATGGAAATATCGATCTAGGATAGGTATACATGTTGATGTGGGTTTTACTGATGCATATACATGATGGCATATGCAGCATCTATTCATATGCTCTAACCTTGAGTACCTATCTATTATAATAAACAAGTATGTTTTATAATTATTTTGATCTTGATATACTTGGATGATGGCATATGCAGCAGCTATATGTGGATTTTTAGCCCTGCCTTCATACGCTATTTATTTGCTTGGTACTGTTTCTTTTGTCGATGCTCACCCTGTTGTTTGGTGTTACTTCTGCAGGTCGACTCTAGAGGATCCAACAATGGACAACAACCCCAACATCAACGAGTGCATCCTACAACTGCCTGAGCAACAACCGAGGTGGAGGTGCTGGGCGGCGAGCGCATCGAGACCGGCTACACCCCCATCGACATCAGCCTGAGCCTGACCCAGTTCCTGCTGAGCGAGTTCGTGCCCGGCGCCGGCTTCGTGCTGGGCCTGGTGGATCATCTGGGGCATCTTCGGCCCCAGCCAGTGGGACGCCTTCCTGGTGCAGATCGAGCAGCTGATCAACCAGCGCATCGAGGAGTTCGCCCGCAACCAGGCCATCAGCCGCCTGGAGGGCCTGAGCAACCTGTACCAAATCTACGCCGAGAGCTTCCGCGAGTGGGAGGCCGACCCCACCAACCCCCCTGCGCGAGGAGATGCGCATCCAGTTCAACGACATGAACAGCGCCCTGACCACCGCCATCCCCTGTTCGCCGTGCAGAACTACCAGGTGCCCCTGCTGAGCGTGTACGTGCAGGCCGCCAACCTGCACCTGAGCGTGCTGCGCGACGTCAGCGTGTTCGGCCAGCGCTGGGGCTTCGACGCCGCCACCATCAACAGCCGCTACAACGACCTGACCCGCCTGATCGGCAACTACACCGACCACGCCGTGCGCTGGTACAACACCGGCCTGGAGCGCGTGTGGGGTCGACAGCCGCGACTGGATCAGGTACAACCAGTTCCGCCGCGAGCTGACCCTGACCGTGCTGGACATCGTGAGCCTGTTCCCCAACTACGACAGCCGCACCTACCCCATCCGCACCGTGAGCCAGCTGACCCGCGAGATTTACACCAACCCCGTGCTGGAGAACCTGACGGCAGCTTCGCGGCAGCGCCCAGGGCATCGAGGGCAGCATCCGCAGCCCCACCTGATGGACATCCTGAACAGCATCACCATCTACACCGACGCCCACCGCGGCGAGTACTACTGGAGCGGCCACAGATCGCGGCCAGCCCCGTCGGCTTCAGCGGCCCCGAGTTCACCTTCCCCTGTACGGCACCATGGGCAACGCTGCACCTCAGCAGCGCATCGTGGCACAGCTGGGCGGGAGTGTACCGCACCCTGAGCAGCACCCTGTACCGTCGACCTTTCAACATCGGCATCAACAACCAGCAGCTGAGCGTGCTGGACGGCACCGAGTTCGCCTACGGCACCAGCAGCAACCTGCCCAGCGCCGTGTACCGCAAGAGCGGCACCGTGGACAGCCTGGACGAGATCCCCTCAGAACAACAACGTGCCACCTCGACAGGGCTTCAGCCACCGTCTGAGCCACGTGAGCATGTTCCGCAGTGGCTTCAGCAACAGCAGCGTGAGCATCATCCGTGCACCTATGTTCAGCTGGATTCACCGCAGTGCCGAGTTCAACAACATCATCCCCAGCAGCCAGATCACCCAGATCCCTGACCAAGAGCACCAACCTGGGCAGCGGCACCAGCGTGGTGAAGGGCCCCGGCTTCACCGGCGGCGACATCCTGCGCCGCACCAGCCCCGGCCAGATCAGCACCCTGCGCGTGAACATCACCGCCCTGAGCCAGCGCTACCGCGTCCGCATCCGCTACGCCAGCACCACCAACCTGCAGTTCCACACCAGCATCGACGGCCGCCCCATCAACCAGGGCAACTTCAGCGCCACCATGAGCAGCGGCAGCAACCTGCAGAGCGGCAGCTTCCGCACCGTGGGCTTCACCACCTTCAACTTCAGCAACGGCAGCAGCGTGTTCACCCTGAGCGCCCGTGTTCCAACAGCGGCAACGAGGTGTACATCGACCGCATCGAGTTCGTGCCCGCCGAGGTGACCTTCGAGGCCGAGTACGACCTGGAGAGGGCTCAGAAGGCCGTGAACGAGCTGTTCACCAGCAGCAACCAGATCGGCCTGAAGACCGACGTGACCGACTACCACATCGATCAGGTGCGAGGCCCCGGAAGGGTGGAGGAGCTTCCGTGCTCAACTCCGGACGCACCACCATCTGCGACGGCCACAACGTGATGGCCCAGGACCCGTTCTCCTTCCAGTACAAGTCCTCGACACCATCCAGAAGGAGTGGACCGAGTGGAAGCGCGACGACCACTCACTCTACGTGGCTCCTGTGGTGGGTACTGTGGCCATCTTCCTCCTCAAGAAGCTCGGAGGCTTCGCTGCCAAGCGCATCCTCAATGGCCTCTGGGACCTCGTGTTCCCAAACGACAACACCAAGCTCATGCAGGACATCCTCCGTGAGACCGAGAAGTTCCTCAACCAGCGCCTCAACGCTGACACCCTCTCCCGCGTGAACGCCGAACTTGAGGGCCTCCAGAAGAACGTTGCCGAGTTCAACCGTCAGGTGGACAACTTCCTCAACCCGAACCGTAACCCGACTCTCCTTTCCATCATCCCCGGTGAACACCATGCAGCAGCTCTTCCTCAACCGCCTCACCCAGTTCCAGCTCGCGGCTACGAACTCCTTCTCCTTCCACTCTTCGCTCAGGCCGCCAACCTCCACCTCTCCTTCATCCGCGATGTGATCCTCAACGCAGAGGAGTGGGTATCTCCGCTGCCACTCTCCGCACCTACCGTGGCTACCTCAAGGACTACACCAAGGAGTACTCCAACTACTGCATCAACACCTACCAGACAGCCTTCAAGAAGCTCAACACCCTCTCCACGAGATGCTTGAGTTCCGCACCTACATGTTCCTCAACGTGTTCGAGTACGTGTCCATCTGGTCCCTCTTCAAGTACCAGTCCTCCTCGTGTCCTCCGGCGCCAACCTCTACGCCTCCGGCTCCGGCCCAGCAGAGACCCAGTCCTTCACCTCCCAGGACTGGCTGTTCCTCTACCCTCTTCCAGGTGAACTCCAACTACGTGCTCAACGGCTTCTCCGGCGCCCGCCTCTCCAACACCTTCCCGAACATCGTGGGCCTCCTGGCTCCACAACTACCCACGCCCTCCTCGCCGCTCGCGTGAACTACTCGGTGGCATCTCTTCCGGCGACATCGGCGCTTCCCGTTCAACCAGAACTTCAACTGCTCTACCTTCCTCCCTCCGCTCTCACACCGTTCGTGCGCTCCTGGCTCGACTCCGGCTCCGACCGCGAGGCGTGGCTACCGTGACCAACTGGCAGACCGAGTCCTTCGAGACCACCTCGGCCTCCGCTCCGGTGCCTTCACCGCACGCGGCAACTCCAACTACTTCCCGGACTACTTCATCCGCAACATCTCCGGCGTGCCGCTCGTGGTGCGCAACGAGGACCTCCGTCGCCCACTCCACTACAACGAGATCCGCAACATCGCCTCCCCGTCCGGCACCCTGGAGGTGCACGCGCCATGGTGTCCGTGCACAACCGCAAGAACAACATCCACGCTGTGCACGAGAACGGCTCCATGACCCACCTCGCCCAAACGACTACACCGGCTTCACCATCTCCCCGATCCACGCTACCCAGGTGAACAACCAGACCCGCACCTTCATCTCCGAGAAGTTCGGCAACCAGGGCGACTCTCTCCGCTTCGAGCAGAACAACACACCGCTCGTTACACCTCCGCGGTAACGGCTCCTACAACCTCTACCTCCGCGTGTCTTCCATCGGCAACTCCACCATCCGCGTGACCATCAACGGCCGTGTGTACACCGCCACAAACGTGAACACCACCACAAACAACGACGGCGTGAACGACAACGGTGCTCGCTTCTCCGACATCAACATCGGCAACGTGGTGGCCTCCTCCAACTCCGACGTGCCGCTCGACATCAACGTGACCTCAACTCTGGCACACAGTTCGACCTCATGAACATCATGCTCGTGCCGACCAACATCTCCCGCTCTACTAAGAGCTCTAGATCTGTTCTGCACAAAGTGGAGTAGTCAGTCATCGATCAGGAACCAGACACCAGACTTTATTCATACAGTGAAGTGAAGTGAAGTGCAGTGCAGTGAGTTGCTGGTTTTTACAACTTAGTATGTATTTGTATTTGTAAAATACTTCTATCAATAAAATTTCTAATTCCTAAAACCAAAATCCAGGGTACTGAGCTCGAATTGTAATCATGTCATAGCTGTTTCCTGTGTGAAATTGTTATCCGCTCACAATTCCACACAACATACGAGCCGGAAGCATAAAGTGTAAAGCCTGGGTGCCTAATGAGTGAGCTAACTCACATTAATTGCGTTGCGCTCACTGCCCGCTTTCCAGTCGGGAAACCTGTCGTGCCAGCTGCATTAATGAATCGGCCAACGCGCGGGAGAGGCGGTTTGCGTATTGGCTAGAGCAGCTTGCCAACATGGTGGAGCACGACACTCGTCTACTCCAAGAATATCAAAGATACAGTCTCAGAAGACCAAAGGCTATTGAGACTTTTCAACAAAGGGTAATATCGGGAAACCTCCTCGGATTCCATTGCCCAGCTATCTGTCACTTCATCAAAAGGACAGTAGAAAAGGAAGGTGGCACCTACAAATGCCATCATTGCGATAAAGGAAAGGCTATCGTTCAAGATGCCTCTGCCGACAGTGGTCCCAAAGATGGACCCCACCCACGAGGAGCATCGTGGAAAAGAAGACGTTCCAACCACGTCTTCAAAGCAAGTGGATTGATGTGAACATGGTGGAGCACGACACTCGTCTACTCCAAGAATATCAAAGATACAGTCTCAGAAGACCAAAGGGCTATTGAGACTTTTCAACAAAGGGTAATATCGGGAAACCTCCTCGATTCCATTGCCCAGCTATCTGTCACTTCATCAAAAGGACAGTAGAAAAGGAAGGTGGCACCTACAAATGCCATCATTGCGATAAAGGAAAGGCTATCGTTCAAGATGCCTCTGCCGACAGTGGTCCCAAAGATGGACCCCCACCCACGAGGAGCATCGTGGAAAAAGAAGACGTTCCAACCACGTCTTCAAAGCAAGTGGATTGATGTGATATCTCCACTGACGTAAGGGATGACGCACAATCCCACTATCCTTCGCAAGACCCTTCCTCTATATATGAAGTTCATTTCATTTGGAGAGGACACGCTGAAATCACCAGTCTCTCTACAAATCTATCTCTCGACGCATGCCTACAGTGCAGCGTGACCCGGTCGTGCCCCTCTAGAGATAATGAGCATTGCATGTCTAAGTTATAAAAATTACCACATATTTTTTTGTCACACTTGTTTGAAGTGCAGTTTATCTATCTTTATACATATATTTAAACTTTTTACGAATAATATAATCTATAGTACTACAATAATATCAGTGTTTTAGAGAATCATATAAATGAACAGTTAGACATGGTCTAAAGGACAATTGAGTATTTTGACAACAGGACTCTACAGTTTTATCTTTTAGTGTGCATGTGTTCTCCTTTTTTTTGCAAATAGCTTCACCTATATAATACTTCATCCATTTTATTAGTACATCCATTTAGGGTTTAGGGTTAATGGTTTTATAGACTAATTTTTAGTACATCTATTTTATTCTATTTTAGCCTCTAAATTAAGAAAACTAAAACTCTATTTTAGTTTTTTATTTAATAATTTAGATATAAAATAGAATAAAATAAAGTGACTAAAAATTAAACAAATACCCTTTAAGAAATTAAAAACTAAGGAAACATTTTTCTTGTTTCGAGTAGATAATGCCAGCCTGTTAAACGCCGTCGACGAGTCTAACGGACACCAACCAGCGAACCAGCAGCGTCGCGTCGGGCCAAGCGAAGCAGACGGCACGGCATCTCTGTCGCTGCCTCTGACCCCTCGAGAGTTCCGCTCCTGCGTTGGACTTGCTCCGCTGTCGGCATCCAGAAATTGCGTGGCGGAGCGGCAGACGTGAGCCGGCACGGCAGGCGGCCTCCTCCTCCTCTCACGGCACCGGCAGCTACGGGGATTCCTTTCCCACCGCTCCTTCTTTCCTTCCTCGCCCGCCGTAATAAATAGACACCCCCTCCACCCTCTTTCCCCAACCTCGTGTTGTTCGGAGCACACACACACAACCAGATCTCCCCAAATCCACCCGTCGGCACCTCCGCTTCAAGGTACGCCGCTCGTCCTCCCCCTCTCTACCTTCTCTAGATCGGCGTTCCGGTCCATGGTTAGGCCCGGTAGTTCTACTTCTGTTCATGTTTGTGTTAGATCCGTGTTTGTGTTAGATCCGTGCTGCTAGCGTTCGTACACGATGCGACCTGTACGTCAGACACGTTCTGATTGCTAACTTGCCAGTGTTTCTCTTTGGGAATCCTGGGATGGCTCTAGCCGTTCCGCAGACGGATCGATTTCATGATTTTTTTGTTTCGTTGCATAGGGTTTGGTTTGCCCTTTTCCTTTATTTCAATATATGCCGTGCACTTGTTTGTCGGGTCATCTTTTCATGCTTTTTGTCTTGGTTGTGATGATGTGGTCTGCTTGGGCGGTCGTTCTAGATCGGAGTAGAATTCTGTTTCAAACTACCTGGTGGATTTATTAATTTTGGATCTGTATGTGTGTGCCATACATATTCATAGTTACGAATTGAAGATGATGGATGGAAATATCGATCTAGGATAGGTATACATGTTGATGCGGGTTTTACTGATGCATATACAGAGATGCTTTTGTTCGCTTGGTTGTGATGATGTGGTGTGGTTGGGCGGTCGTTCATTCGTTCTAGATCGAGTAGAATACTGTTTCAAACTACCTGGTGTATTTATTAATTTTGGAACTGTATGTGTGTGTCATACATCTTCATAGTTACGAGTTTAAGATGGATGGAAATATCGATCTAGGATAGGTATACATGTTGATGTGGGTTTTACTGATGCATATACATGATGGCATATGCATCTATTCATATGCTCTAACCTTGAGTACCTATCTATTATAATAAACAAGTATGTTTTATAATTATTTTGATCTTGATATACTTGGATGATGGCATTATGCAGCAGCTATATGTGGATTTTTTAGCCCTGCCTTCATACGCTATTTATTTGCTTGGTACTGTTTCTTTTGTCGATGCTCACCCTGTTGTTTGCGTTACTTCCAGGTCGACTCTAGAGGATCTACCATGGCCACCGCCGCCGCCGCGTCTACCGCGCTCACTGGCGCCACTACCGCTGCGCCCAAGGCGAGGCGCCGGGCACCTCCTGGCCACCCGCCGCGCCCTCGCCGCGCCCATCAGGTGCTCAGCGGCGTCACCCGCCATGCCGATGGCTCCCGGCCACCCTCCCGGCCGTGGGGCCACCGATCCCCGCAAGGATCCGACGCCCTGCCCGCCACCTTCGACGTGATCGTGCATCCAGCTCGCGAACTCCGCGGCGAGCTTCGCGCTCAGCCATCCAAGAACTACACCACTCGCCTCCTCGCCGCTGCCCTCGCTGAGGGCGAGACCCGCGTGGTGGGCGTGGCTACCTGAGGACGCCGAGGCCATGCTCCGCTGCCTCCGCGACTGGGGCGCTGGCGTGGAGCTTGTGGGCGATGACGCCGTGATCCGCGGTTTCGGCGCTCGCCCACAGGCCGGTGTGACCCACTCCAGCCCAGGCGCGCTGCCGCGGTGGCCCGCCTCCTCATGGGCGTGGCCGCTCTCACCTCTGGCACCACTTTCGTGACCGACTACCCGGACTCCTCGGCAAGCGCCCTCAGGGCGACCTCCTGAGGCCCTCGAACGCCTCGGTGCCTGGGTGTCCTCCAACGACGGTCGCCTCCCGATCTCCGTGTCCGGCCCAGTGCGCGGTGGCACCGTGGAGGTGTCCGCCGAGCGCTCCTCCCAGTACGCCTCCGCCTCATGTTCCTCGGCCCTCTCCTCCCGGACGGACTCGAACTCCGCCTCACCGGCGACATCAAGTCCCACGCTCCGCTCCGCCAGACACTCGACACCTCTCTGACTTCGGCGTGCGCGCCACTGCCTCCGACGACCTCCGCCGCATCTCCATCCCGGGTGGCCAGAAGTACCGCCCAGGCCGCGTGCTCGTGCCGGGCGACTACCCGGCTCGCTGCCATCCTCACCGCCGCTGCCCTCCTCCCAGGCGAGGTGCGCCTCTCTAACCTCTGAGCACGACCTCCAGGGCGAGAAGGAGGCCGTGAACGTGCTCGCGAGATGGGCGCTGACATCGCGCGAGGGCGATACCCTCACGTGCGCGGTACGCCTCTCCACGCCGTGACTCGCGACGGCGATTCCTTCACCGACGCCGTGCAAGCCCTCCGCCGCTGCTGCCTTCGCGGCGGCACCACCTGGGAGAACGTGGCCACTCGCCTCAAGGAGTGCGACCGCATCTCTGACACCAGCGCTGAGCGCCTCGGCCTCCGCGCCGCACGCGAGACCGCCGACTCTCTCCGTGACTGGCTCTGCTCACCTCGCGGTGGCATCACCGCCGACGCCACGGCGACCACCGCATGATCATGCTCTCACCCTCCTCGGCCTCGCAGACGCTCCTCCGCAATCACCGCGCACACCACATCCGCAAGTCCTACCCTCAGTTCTTCGCTCACCTCAAGCCCTCGGCGCCGCCGAGTACGCTGAGGCCACCGCCTAATAGGAGCTCGAGTTTCTCCATAATGTGTGAGTAGTTCCCGATAAGGGAATTAGGTTCCATGGGTTTCGCTCATGTGTTGAGCATATAAGAAACCCTTAGTATGTATTTGTATTTGTAAAATACTTCTATCAATAAAATTTCTAATTCCTAAAACCAAAATCCAGTACTAAAATCCAGATCCCCGAATTAATTGGCGTTAATTCAGTACATTAAAACGTCCGCAATGTGTTATTAAGTTGTCTAAGCGCCAATTTGTTTACACCACAAAATAGCCTCGGGAATGACGGTTCCGACGCCTTCTCCGAGACTGCATCAACCCTCCGTCAAGATCGACGACACGTCCGAAGCTCGAGGCACCCTCGGCCCGAAGGCACCCTCGGCTCGATCGAGGCACCCTCGCTCGGCAGGCACCTCGGCTCGGCAGGACCCTCGCCACGAGGATAAGGCACTGCGCGTCGAGAGGAGCGGGCTGGGTCACGCCTAATCGAAACTGGCAGACCCGTGCAATATCTCCACAGAGAGCTACCCTCACCGAGCAGGCTCGCGGTTGTGCGCATGCCAAGTCGTTTGTCTTGGTGGGGACGGAAAGAGTTCTACCACCCCCTCGAGCATCCTCCGCGATGCATTTCCATCGCCAAGGCCGAGCTCCTACAAGAGATACACTCGGGCTTCACGGCCATCACGCGAAGACCTCGAGCCCTTGTTGAAACGCCAAACAAGGTTTCATCGCAGCCACGGCGATGTGCCCGCCACTAAATTGTCCGCACTGCGAAGGGGTGTCGATTCTCACGGGCAGACCACACTGCTGCTCAGCCCTGCAGACGATACCCATCACACGCCTTTTCGTGTGGTCCGGATCTCGTCGGCCCTTGCGGCACCCCGGCTACCTTTGCTGGTCACCATCGACAAATTCTCCAAGTGGATCGAGCCTAAACTCAAAGGTCGGAACAAGCGGTGGCGTTCTTCACCAACATCATCCATCGCTTCAGGTCCCGAGAGCACCTAGAGGGGGTGAATAGGTGATCTGTAAAAACTTAAACTTAAGCCACAAAAACTTGTTAAGGTTAGTACAAGTATGGCCAAGTGGCTAGAGAGAACTCAAACACAATAACCACAAGAAAGCAATCACGAGTTGACACGGTGGTTATCCCGTGGTTCGACCAGTACAAAACTTGCCTACCACGTTGTAGCGTCCCAACGACGAGAGTTGCACTCAACTCCTCTCAAGTGATCCAATGATCAACTTGAATACCACGGTGTTTCTTTCCTTTGATCTTTTTCCTAGTTCAGAATCTCCACAACTTGGTCTCTCGCCCTTACAATTGAGTTCACAAAGAAATACTGAGTAAGGTGGGAATGAGCAAGCACACAAGACTCAATCAGAGCAACAACACTTACACATGTCGCAACAAGAGCTCGCAGCACAACACAAAGAGTTCACAACTCCACAAGAGCTCTATATGCTATCACAATGAAGCGAATGCGAGATTGATGTCTTGGTGCTTAGAAGAGTTGTAGGAATGCTTGGTGTACTCCTCCATGCGCCTAGGGGTCCCTTTTATAGCCCCAAGGCACTAGGAGCCGTTAATAAATCACGGAAGGCCATCTTGCCTTACGTCTCGTAAGCGCCGCCGGACGGTCCGGTGCTGCTACACCGTGTCGGTGCGATTGTTTCCTTAAATGGCGGCTGAACCGTTGTCGGCAAATTGCAGATCTGGCGCCGCCGGGCCCGATCCGGTGCACCGGCGATCCGTGCTTCCGACAGTTGGCTCGGCCACGTGTCGCGCCGTCGCGGCCGGCCGATCTTGGCCGCCGTTGAGCTCACGACCCGATCGGGTGCTGCGGTGATCCGGTGAATTCGCCGAAATCGCCGGAGAAAAACGAGCGGCCTCTTGGCCAAGGAAGATCTGGCAGCGGGACCTTTGTCGGTGCACCACCGAGCGATCGGTGCCCAGACGAAGCGGCCTTGGTACACACAAAGTCTTCTCTTCTCTTCTTCTATCTGTTCTAACACTTAGACAAATATATTAGTACACAAAACCAATGTACTAAGGCTTAGAAACATACCTTAACTTGTGATTTGCACTTTGTTCATCCTTGGGCATATTTTCACATTTAAGCACTTGTGTTTGCACTCAATCACCAAAATACTTAGAAATGGCCCAAGGCACATTTCCTTTCAATCTCCCCTTTTTGGTGATTTATGCCAACACAACATAAAGTAGATAGAACAAGTGCAAAATCACTTCAAATAAAAACTCAAATTGGTTTTGATTCAATTTTGCATATATGGATCATCCTTTGCCACCACTTGGTTTGTTTTTGCAAATCAAACTCAAATCTCTATCTCTAAGTCAAACACACATGATGAAGTATAAAGAGAGTCATTCCAAAAGAGATTGATCAAAGATTTCAAAACTCCCCTATTTCCCATAATCAACACTTCTCCCACAAGAAGCCAACTTTTGACAATAGAGACAATAAGAGACAATAAGCTTTGACAAAACAAAACTCTATTCTACTATTTTCAAAATCTCAAGTGGTGTATGATCCATTTATCACTTTGGCTTTTTTTCTCCCCTTTATCAAGCACCAAAAGTTAATCTTGGCCTTTTAACCCCATTGCCTCACCAAAGTCTTCAATTAAGAGCAAATGGCAATAAGATTTCATGAGATGAACTTGGAATTAGTTACCCTCTCTCGAGTGCAATGGAAGTCTTTCATGGTCCAAGTCCACCTTTTCCCTTTCAATCCTCCCGAGACTAAATCAAGCAAACTCAAGCAAATGGTTAGTCTCAAAGGGTCAAGTTGTAACACATCTCCCCTAAACATGTGCATCACTGCGGGACTTGTGAGGTCCAGGAGTGTTTGTACAACTTGAGCACCATAATAAGCAACATAATGCAAAAGAACATGATCAAAAGCATAACTACATGTATGCTACAATTCAATCCAGTTCCGAATCTAAGACATTTAGCTCACTACGCAACTTTGCAAAGGTCTTCTCATCTAGAGGCTTAGTGAAGATATCGGCTAGTCTGGTTCTCGGTGCTAACATGAAACACTTCGATATCTCCTTTCTTTGGTGGTCTCTCAAAAGTGATGCCGGATGTCTATGTGCTTTGTGCACGTGTTCAGCAGGATTCTCCGCCATCGGATAGCACTCTCATTGTCACATAGAGTGGGACTTTGCTCGATTGTAGCCAAAGTCCCCGAGGGTTTGCCTCATCCAAAGTAGTTGCGCGCAACACCGCCTGCTGACAACATACTGGCCTCGGCGGTGGGATAGGGCAGCGGAAGTTTGTTTCTTAGAGTTCCATGACACAGGACCTTCCTAAGAATTACGTCCCGATGTACTCTTCCTATCGACCTTACATCGGCATAGTCGGTCCGAGTATCCAACTAAGTCAAAGGTAGACCCTTTGGATACGAGGCAAAGCAAGCGTAGCAACTAAATATCTAAGAATCGCTTCACCACTAAGTGACATTCCTTAGGATCGGATTGAAATCTAGCACACATGCATACGCTAAGCATAATATCCGGTCTACTAGCACATAAGTAAAGCAAAGAACCTATCATTGACGGTATGCTTTTTGATCAACGGACTTACCTCCTTTGTTGAGGTCTTGTGTCCGTGGTCCCCATCGGATCTTTGCGGGCTTGGCGTCCTTCATCCCAAACCGCTTGCAGATCTTGCGTGTGCCGTTTGGGAGATGAAGTGCCGCAGAGTTGCTTCACTTGGAACCCAAGGAAGTAGTTCAACTCGCCCATCATTGACATCTCAATTCTACGCGTCATCACTGCTAAACTCTTCACAAGACTTTTGTTAGTAGAACCAAATATTATGTCATCGACATAAATTTGGCACACAAACAAATCACCATCATGTCTTTGTAAAAGAGTTGGATCGCTTTCCCAACCTTGAAAGCATTAGCAATTAAAAGTCTCTAAGGCATTCATACCATGCTCTTGGGGCTTGCTTAAGTCCATAGAGCGCCTTAGAGCTTACACACAGTCGAGTCTGCTTCATCCTCGAAGCAGGGTTGCTCCGTACACCTCCTCCTTGATTGGCCGTTGAGAAAGCGCTCTTCACATCCATTTGGAACAACCGAAAGAATGGTGAGCGGCATGCTAGCAAAATCGAATGGACTCTAGCCTAGCCCGGGAGCAAAGTCTCCTCAAAGTCCAACCTGCGACTTGGCATAACCTTTGCCACAAGTCGAGCCTTGTTCCTCGTCACCACCGTGCTCGTCTGTTTGTTCTGAACACCCACTGGTTCCCACAACATTTTGCTTCGGACGAGGCACGTGTCCAAACTTCATTGCGCTTGAAGTTGTTTAGCTCCTCTTGCATGGCCAACACCTAGTCGGATCTAGCAAGGCCTCTTCTACTGAAGGCTCAATAGAAGAGACAAAAGAGTAATGCTCACAAAATTAACTAATCGAGATCAGTAGTTACTCCCTTGCTAATGTCACCCGAATTTGGTCGACGGGATGATCCCTTTGAATCATCGCTCGAACTTGGGTTGGAGGTGCCGGTTGCGCTTCTTCCTCCATCACATGATCATCTTGTGCTCCCCCTTGATCAGCGCCTCCACTTGAGTTGGGGCATGCACCATTGTTGAGGAAGAAGATTGATCGTTCATCTTGTTCACGTGGCGCACTTCTCCAATCGCCATGGTTCGTATAGCGTAGTGAACATCTTCTTCATCTACATCATCAATCAACAACTTGCTCTCTTGGAGAGCCATTAGTCTCATCAAATACAAGCGTCGCTAGAGACTTCAACCAAACCCGATGATTTGTTGAAGACTCTATACGCCTTTGTATTTGAGTCATAACCAATAAAAACCCTTCTCTGCTTTGGGAGCAAACTTAGAATTTCTACCTTCTTCACTAGAATGTAGCATTTACTCCCAAATACAGAAGTCGATACATTGGGTTTGTTCTGGTTAGTAGCTCATCGGACGTCTTCTTGAGGAGGCGTGAAGGTAGACCTGTTGATGGCGTGGCAAGCGTGTTGCGGACTTCAGTCCAAAGCACTCGGGTCTTGAACTCCTAACATCGTCCTCGCCATGTCGATGAGCGTCCTGTTTCTTCCTTTCTACCACACCATTTTCTTGTGGTGTGTAGGGAGCGGAGAACTCGTGCAATCCCTTCATCTTCAAGGAACTCCTCCACTTGAAGTTCTTGAACTCTTACATTGTCGCTCTTATCTTCTTCACCTTGAGCTCAAACTCATTTTGAGCTCTCTGAGAAGCGCTGAGGGTCCCTGGTTTGAACTTATCCCGCAAAAAGAACACCCAAGTGAAGCGGAAAAGTCATCAACAATAACTAAACCATACTTCCCTCCTATGCTCGGATAGCGGCGAGTCAGAAGAGATCCATATCGGCAGCTCGGGGTCTTGAAGTGGTCATCACGTTCTTCTGTGATGTGCTCCTCCCACTTGTTTACACGCTTGACAAGCCGCACAAGGTCTATCTTTTTAAATGCACGTTAGTCAAACCTCACGTGTTCTCCTTTAGAAGCTTGTGAAGGTTCTTCATCCCCACATGTGCTAAGCAGCGATGCCACAGCAATCCATGTAAGTCTTAGCTATTAAGCATGCATCTAGACCGCCTCTTCTTTTGCAAAATCAACTAAATAAAGTTTGCCGTCTAATACACCCTTAAAAGCTAGTGAACCATCACTTCTTCTAAAGCAGACATCTACATTTGTAAATAGACAGTTATACCCCATATGACATAATTGACTAACAGATAGCAAATTATATCCAAGACTCTCTACTAAAAATACATTGGAGATAGAATGCTCATTAGAAATCGCAATTTTACCTAACCCTTTTACCTTGCCTTGATTCCCATCACCGAATATAATTGAATCTTGGGAATCCTTATTTTTGACGTAGGAGGTGAACATCTTCTTCTCCCTCGTCATATGGTTTTGTCATCCGTATCCATAATCGAGCTTGAAGGATGCATAAACGCAAACAAATTAAGCTTGGTTTAGGTACCCAACTCATGTTGGGTCCTACAGGTTAGCACAAATATCCTTAGGGACCCAAATGCAAGTTTTGTCTCCTTGCATTTTGCCCCTAACTTCCTAGCAACAATTTTCTTATCCTTTCTACAAATAGCAAAGGAAGCATTTAAAGCATAATAAATTGTAGAAGGTTCATTCACTACTTTCCTAGGAGCATGAATAACATTCTTTCTAGGCACATGATGAATAGCATTTCTTTTAGGAACAACATTTCTCTAGTAACATTTCTATCATACACATAAGAGGAACTAGAGCAAACATGGCATGAGAATCATAAACATATGAATCAAAAGCATCATGACTTACATTTCTAGTTTGTCTTCTATCATGATACAAAAGGCATGGTTCTTTTGCACATTACTAGCCATAGGGACCTTCCCTTTCTCCTTGGCGGGAATGGAGCCTTATGGCTTGTTAAGTTCTTAGCTTCTCTCTTGAAGCCAAGTCCATCCTTAATTGAGGGTGTCTACCAATTGTGTAGGCATCCCTTGCAAATTTAGCTATCGAAATCATTCTTGCTAGTCTTAAGTTGAGCATTAAGACTAGCTACTTCATCATTAAGCTTGAAATTGAAACTAGGTGTTCACTACAAGCATCAACGTCAAAATCTTTACACCTAGTACAAATTTCAACATGTTCTACACAAGAATTGGATTTATTTGCTACTTCTAATTTAGCATTTAAATCATTGTTGACACCTTTCAAAGTAGAAATGGTTTCATGACAAGTAGATAGTTCAAAAGCATTTCATTTCTTTTAACTTCTAAAGCATAGGATTTTTGTGCCTCAACAAATTTATCATGCTCTTCATACAACAAATCCTCTTGCTTTTCTAAGTATATTCTTTTCATTCAAGGCATCAATTAATTCATTAATTTTGTCTATCTTAGATCTATCTAAGCCCTTGAACAAACATGAATAATCTACTTCATCCTCATCACTAGATTCGTCCTCACTTGAAGAAGCATAGGTAGAGTTGCGAGTACATACCTTCTTCTCCCTTGCCATAAGGCATGTGTGCCTTCGTTGGGAAGAGGGTTGATTTGTTGAAGCGGTGGCGAGTCCTTCATTGTCGAGTCGGACGAGAGCAATCAAGTCCCACTCCTGCTAGATGCGCCTCACCCTTTGCCTTATAATGCTTCTTCTTTTCCCTCTTGTTTCCCTTTTCTGGTCACTTTCATTATCGGGGCGAGCTTAGCAATAAATGACCAAGCTGCATTTGAAGCATGATCGCTTCCCTTGGTCTTAGTCTTGCTCGGTATCCATTGCGACCTCTGCAGTCTTGAAGCGCTTAATGATGAGAGCCATTTCTTCATCATTAAGCCGCCGCCTCAATTTGCCCCTTGCTGGTAGTGCCTCCTTATCTCGATGCCTTGAGCAATGGGTTGAGGCTCGTGGATTGGACCGTTCAACGCGTCATCGCGTACCTTGCCTCCTTGATCATCATCCGCGGCTTACAAATTTTCCAAGAATTTCTTCGGGCGACATTTTGGTAATACAGGATTCTCACGAATATTATTCACCAAATGAGGATCAAGAATGAGACCTTAGCATGAGGCTGGACGTCGTGGTCCGTCCATCGCGTGCTTCGTAGCTCCTTATCTTGTTGACAAGGGTCTTGAGCGGTTGTATGTTTGAGTTGGCTCCTCGCCCTTATCATCCTGAACCGTCAAGCTCGCCTCTACCAACTCCATTTTGGTGAGCAAGGTAGCATCATTTCCCTCATGAGAGATCTTGAGGTATCCCAGATTGTGGCGTTATCCAAGCCACTCACTTTATTATATTCATCCTGCACAATGAGGCTAGAAGAAGCGAGTAGTAGCTTGTGCATTCTTATGGATTTGCTCATTAATGAATATAGGACTATCGAACTATTAAAGTGCATTCCACTATCTACAATCTCCCATATGCTAGGATGGAGAGAGAATAGGTGACTCGCATTTTGTGGCTCCAAAATCCGTAGTCCTCCCCATCAAAGTGTGGGGGCTGCCGAGTGGAATAAAAGCAAATGAATTTGAACTTTGCGAATCGAGTAGTCAAAAGAAAAGTTAGAATTAGGTTTCCTTTGTTTGTCGTGGTCGTCGTCCTTTTGGGAAGAAGAGGACTCATCATTAAGTCGAGTAGGCGATCTCCTTGATCGCCTTGTCTTCTTCTTCCCATCTCTTCGCTGTGCCCGAGCCCGAGTCATTGGACTTGTCATCCCTTGGCTCGTTGACGAAGGACTCCTTCTCCTTGTCGTTGATCACAATTCCCTTCCCCTTAGGATCCATCTCTTGGGCGGTTAGTCCTTTCTTGAAGAAACGGCTCGGATACCAATTGAGAGCACCTAGAGGGGGTGAATAGGTGATCTGTAAAACAAACTTAAGCCACAAAAACTTGTTAAGGGTTAGTACAAGTATGGCCAAGTGGCTAGAGAGAACTCAAAACACAATAACCACAAGAAAGCAATCACGGAGTTGACACGGTGGTTATCCCGTGGTTCGGCCAAGTACAAAACTTGCCTACTCCACGTTGTGGCGTCCCAGCGGACGGAGTTGCACTCAACTCCTCTCAAGTGATCCAATGATCAACTTGAATACCACGGTGTTTTTCTTTCCTTTGATCTTTTCCCGTTTGCGAGGAATCTCCACAACTTGGTCTCTCGCCCTTACAATTGAGTTCACAAAGAAATACTGGTAAGGTGGGAATGAGCAGCGCACACAAGACTGAAAATCGGAGCAACAACACGCACACAGTCGCAACAAGAGCTCTAGCACAACACAAAGAGTTCACAACTCCACAAGAGCTCTATATGCTATCACAATGAAACGAATCGTGAGATTGATGTCTTGGTGCTTAGAAGAGTTGTAGGAATGCTTGGTGTACTCCTCCATGCGCCTAGGGGTCCCTTTTATAGCCCCAAAGCAGCTAGGAGCCGTTAGGAACATCACGGAAGGCCATCTTTGCCTTCGTCCGTGGCGCACGGAGACGATCGGTTACACCGGTTCTGTCCGGTGCCGATTTTTCCTTAAATGGCGGCGGCCGCTGCCGATCAGTTGCGAGATCGTGCCGCCGGGCGATCGTGCACACCGGACGATCCGGTGCTTCTTTCCGCCGTTGGCTCGGCCGTGTCGCCAGCCGATCAGCGGCCGGCCGTTGGCCCACGGCCGGCCGTTGGCTCACCGACGATCGGTGCGCCGGCGATCCGGTGAAATAATTTGCCGTCGCCGGGCGAGAAGCGGCCTCTTGGTAAGCGATCTGGCGCCGCGGTTTGTCCGGTGCACCACCGAACCTTTCCGGTGCACCACCGAACGATCGGGTGCCCCCAGATCAAGCGACCCCTTGGTAGTACCGCATAAGTCTTCTCTTCTTCCTTCTATCTGTTTCAACACTTAGACAAATATATTAGTACACAAATGTACTAAGGCTTAGAAACATACCTTAACTTGATTTGCACTTTGTTCATCCTTGGGCATATTTTCACATTTAAGCACTTGTGTTTGCACTCAATCACCAAAATACTTAGAAATGGCCCAAAGGCACATTTCCCTTCATCCCGAACTCCATCATCAAACAACGGCACCCGTTCACCGGCAAAAGTTCCCGGACTTGCGAGGATCACCACATCCGGTGGGCGGGCGCGTGCTCACTCCATACCGAATGGGCAGAGTAGGCGTGCAGCATGCATGATTCTACAAGGACTCAAGCCTCGGATCTACGACCTCAGCGGGTTCGCAGCGATGGATGAAGGAACTCCCTCCCGGTGGTCTAGAGTCCGAGGACAACGCCGAGCCGACCCACGGGCTTCAGCAGCTCTTCTAGTCTATGGGGCCGGGCCATCTTGCAGGGACTTAGAATACGGCTCCCCACCCAGGACGAGGCCTCGGCACCAAAGCAGCCAAGTTAGTCGAGAAGACTCGGGACCCGGTGGAAGAGGCTGGACATGGCCTTACTACATTAACACGGTACCGGCCCGATCCGCTTGCCCACACCCAGGGTGCGGTCCAGAGACCTCCCGCGTGGGCGACACGGTGCTTCGTGCGGCAGGGCGCCGGCGCCTGGCTCACGCGCTCCACGGGAAGGCAGTGCGTCGCCAAAGTTACGAGCCGGCATTACAAGCCGGCCAGCGAGTCAGGAGGTCCGCAGCGCTTGGAACATCCAACAGCTCATCCGCTTCTACCCTTAAGATGCTTTCAAGTTGTTCATATACCTCGCTCCCGCAGTTTAGTCATCAAGGAAGGTCGGCCTTGCCTCAAGGCAGGCCACTCCTCGGGGCTAAAAGGGGAACCCTGCTGTCGAAATTTTCCTCAAAAAGATCCTTCTGCCCGAATGTCTTTCGTGCTTTTCGACTACTGAAAGTGGATCCCGCGGCGGAGTACGTAAGCGGCCCGGTGGCGGCCGAGAGGACTCCCGGCTCCGGGATACGGATACCTCACTCATCACCTGTGATAAGTAACTCGCGTTCGGATAAGCGATTCCGCGGATCGAACAAGTCTTCACACTCGCAGCTCCTCTCCGGCGATTCTTGGCCCTTCTCGCTCGTCGGTGGCGTACCCATGGGCGGGCAAGAGTGCGTGGCGGCAAGGCCGGCCAAGTGAGGATTCCTCGCCTGCGGATACGGATACCTCACTCGTCACCTTCCGTGAAAAGCAACTCCCGCCGCAGACAATTCCGTTCAAGCGAAAAAGTCGGATACTCAAAACAAGAGGAAAAGAAGCGCGGCTTTACAACACAGCGATGGTGTGTTTAGCCTCGCGGTAGCGGCGCACTACAAGATAATCCGATCCTGCGAGCTTGATCTTGAGCGAGTTGAAGAGCGGCCGGCACCTCGGCGCCGACTACACCTTCGGCGGAGTCCGACCTGGCCTCTGGGCGGCGCGGTCCGAGGATCTCCACTGAAGGATCGACATCATCATCACGCCATCGCTCGCGCGAGGTCTTCTCAAGAATCCGGCCAGAGGCGGCTCGGTGGTCACCCGAGGCCCTCGGCCCGTTGTCCCCGACATCGTGGCCGAGCCTCGCAGATCAACTCCGGCGTCGGTCCCGTAATGGACGACCGGCAGAACTCGGCCGACAAGTCTTCTTCCGGTGCTCGCCTGCAATCAAATCGCCACCGCTACCCACAGCCGGCTCATCGACGGCAGTTTCGCTAACTAAGCAGAAGCCTCGGGCAAGCAAGGCCCGGCCGTGAGACTCGCCTCGGGATCCCGGATACGCCACTCCGTCACCTTTATGCGGGCGACTCACTCTTGTGGCGGTTCGAGACAACCCAGCGGCGAGTCTTAGTGCTCAAAATAAAGGAAAAACACGGCTCTGCGTAAAAATACACGCCGCGTTCGGGCCCCGTACAATGAACAAAGGCCGACATTCAAGGTGCCATTACAAACGGAACTCGGTTCCACCTCCGCAGTGCGAACAACCACACGATTGGGGGCTGCGAACAGCGAAGACAACGAGCGGCTCGCCCGCCGCGCGCTCAACGGCCGGCGATGGCGACCACGCTTTCAGGGCCTGGCCGGCGATGACCTCGGGGTGGATGCTGCTGCCGCGAGGCCCTCGCCCATGCCCAAACTCGGAGGCAAGGCGGGCGAAGCGCGTAGAGTTGAGGTGATCGTGGCGGCCGCTATCTTGCGGAAGAGAACTCTTCCACCGCCATGGCGGAAGCGGCGCGGGGCGGCTCGGCCACTCGGCCGAGAGAGCGGTGCACGCGGTGCCGGTAGCCGAATGAGCGGCGGCGCCCTTCCCCCTCGATCGAGGAAAGGAGGGCGCGCCGCCATCGGGGCGACCCCAACTGACACACTCCCTCCCGGCCGATGATGAAAATCCTTGAGGGAGGGAGGCGCATGACGGTTGCTTTCCCAGCCATCGAGCCGGAGGTCACCATCTTGGTGACTGCCGCCGAGGGGTGCAGCCGGCCGCGTGATGAATCCTTGAAGCGAACGATGGTGAAAGGTACCAACTCACTGAGTTGCGTTCCTCAACGACAAAGGCGAGGGATGCGGTGTCCCATCCGGGCTGGAAGGTGGAAACACGATACATAAGGGAGCTGAAGACATGGTCGCCTTTCAAGGTCACCCACCTTTTAAAGCGACTCTCCTACTGCGTCCCGGTAGTCGTGGTGAGTCTTCTCCAACACGCTCAAGGTCCTCCCCCTCGACGCGGTGGGCCCACGCGCGCATACTGGTGACCGGGCGGAAAAGACCGCGCGCGTGGCATGCAACCGCCAGTGATTCACGAGCATTCCTCCACTTTCGCCCGTTCCGGTAGGCGAAGGCGAAGCGACCATGCGGTGGCATACCGCGCCAAGCGCGCGCCTCCGACTTCAACGCACCCGGATGGGGCTTCACGCGTCCGCCGCGGCAGCGCGTCGCTACGATAAGCAAGCTGCGCCGCCACTCGCTGCTTGCGCCTCCTCGATGCGAAACCCGATCACGCGACTGAGGCGACCCTGCGTCAGACGGCCCGTGCGGCCCAAAGCGATGGTCGTCCCGGCAAAAATGGGCGGCAGTAATGGCGGTGGCGAGCGGTAGGCAGTGGTCACGTCGTCGTAGCTTACGCCCATCTCCGGCGGTGAGAGAACCCTCTCCGCGGCGTGAAGACGGCGCCCGTGTTCGCTTCCTCGAGCGGCTCAGCGCCGCAGCGCAGCGGCCGCCGTGACGTTTCGCCGTGCGCATTAACTCCGGCGGGAAAGCGCGCTCCGGCGAGAAGGCGGCGACGGCGCCGCCGCCACAATGGCCAGCGTCAGAAGTACGCCACGTCATTCGATTTTGCATCCTTTTCCTTTTTCCTCTTTCTCTCTCTTACTGTGGACCGGGAAGGATACTAAGGATCCTTCTCCGTGAAGGAAAGCAGGCTCCGAGCCTCCCTCGATAAGAGGTTCAAGGTCGGCCTCGAGAGGTTCAGCAGCCGCCCGAGCGCGTGGGCTCCACACCTCTTGGTTAGAGGTTCAAGGTCGGCCCTCGGAAGGGTTCAACGGCCTCGAGCCACCGGGCTCGCGCCCCTCTGATCGTGGGTTCGGTGGCCCTCGAAGGTTCACGGCCGCCTCGGACGAAGCGAGGGATGACCATGGTGCGCTCGATACATAACCAAGGCTCGGGCTCGCTCCGAGGTACCTAAGACATTTGAGACCAGCAGAAATGATCTTGTAACGGAATCCCATGAGAGGAGGCATCGAGCTCGGACCGTCGACAGGGCCCGGTCCCGTATCACGTAGATACTTTTTTAGCGCGCCTCGGGCCTCTAGCGACAACAAATGGGGCACGGCGTCCACTCGGATTACCCGTGACTCGGAGACACCATGTTTGGCGCCCTCAGGGCAACATGGCGCTTTCCCCCTCCTTGCGGAAAGCGTAGCGGCGTATGTTAAAAAGTCAGTCTGTCTCGAGCCAGTCCTCGCTACTGTCTGGAGCTCGGCCGCCTCGCAAACCCGGCTCGGAAACCGTTGACAACGCCAACATACGACCAAGAACTTGGGACCCGGCCGTGCACCCGGGCTACGGCCACCGCATGAGAGCGACGGTGGCCGTAGGAAGCATTGCGTGGAGGTATTAAGACCTCGAGGAGTCAAACCACTCTCTCCGAGGCCTTGAGAGCTACACACCGGCCAGTCGCGCTCATGCACTCCACCGGGTTCAAAACGCAACAGAAAGGCTGGTCCCTTGCAAAAAGTGCGACAAAGCCTCCAGCGGTGTTAACACTCTCCGAGGCTCGGGGCTCTTGTCGGTACCATAATTAGGGTACCCTCAAGACTCCTAATTCTCATTGTAACCCCATCGGCACAAGGCGCAAAGGCACGATGGTGCGACTAAATCGTGGATCGGTCCATTCGAGGGACTGGATCACGCCTCGCGGCCCAGCCTCGGGCAAGCGGCCGACCACTGGAGGATCTCCGCTCGCAGGCCCCCTCCGGTGACGAACATACTTCCGGCTCGCCGAGGCCCGGTCTTCGCCAAAAGACCACGCCAAATCGCCGCGCTAACTTAAGCCAAATCGCAGGGCATTTAATGCGAGGTGGCTGACACCTTTATCCGACGCCGCCTCGGGCGACAGAGCCGAGTGACCGCGATACTTCGCGCTCAACAATCGGCACAGTGGAAGAACGGCGCCCGCTGCTTCCGATCGGGCTAGTGCCACTCGGCAGTGGTAGACAGCGGCCAGGCACGCCTCGAGCACCATAGGAAACTCCGCCGCCCGACCTAGGGCTCGGACTGGGCTCGGCCGGAAGATGGCGAACTCTGCTCGCCGACCTAGGGCTCGGACTCTGACTCAACCGAGGCGAACTCCGCTCCGAGCCTGACCTGGAGCCGGGACGGCCCGAGCGCCAACTCCGCTCGCCCAACCCGGGCCTGGACCGGGCTCGGCCGGAAGATGGCGAACTCACGCTCACTCGACCAGGGCTCGGACTGGGCTACCGGTAGAGACTGCGAACTCCGCCGTAGGCCTGAGCTCAGACTATGTGACACGATTGTCACTCAGGAGTTTTCTCAAATGCCAAACCAAGAACCATCATTTTATGTGAACCAAAGTAAGCATGAGCATCAAAACAACTTAAGTAATAAAGAATTCACCAAGTAAATACTTAAAAGTAACATGATCAAAACAATTGAGTCTTTGAAAAGATAAAATGTGCAACCTAATTAAGAACCCTAAGTGAGCCCCATGAGCAAAATTCAAGAAAATAAGAAAAGGGAATGAAAGTTTAAAATTATGACTTGAGCCAATTATGAAAATTAAAGAATATTTAATGGGCAACAAGAAATGTTGAGAAAGCTTAGCCAAAATAACTCAAATAAAACCCCAAATCAAGCTCCTCATGTGGGACTCATTAGGATTTCTGATTCGAGATTCTGAAATTCAGACCTTGAGCAAAGATCAGGGATGTTCACCTTGATCCCTAACTTGAATCCTAATGGCAGACAAAATTGTGTCTAACTAACCCTCATGGTGTGCCGGAAAGATGGCATTGGGACGCTGAGCCCTAGACACGACAAAACCTTGGATTTGCCTCGGGTTTGGGCGGAGACGACTAGATTTCTGGCTCCATATCTCTCACCTTGATGGCAAAACCTAAGCCCCCCACACAAGAATGGTAGCTTGTAGGGAGGAGAAGAGGTTTTGTGCACCTGACCAGGCGGAAGCAGGCTCGGATGGCGACCGCACGAGGGCAGGCGAGCGCACGAGCACATCGTGTTCGACCGGTCGTGCCCGCCGAGCTCGCCTAACCCCCGCGCGCTCACCCGTCCGGTCAAGTCCTTCCGCAGCGCCCGCGCCCTCGGCGTTCGCCCGCGCGCCAAAGCCTCGGCACCTCTCGCCCGCACTCACCCTCTCGGCCCGACCCACCTTTCCTTAAGCTCGTGAAGGAATTACTTCGCCATTGCCGCGAGCCGGCCACCATGGCCCCCTCGACCACCCTCAAGCCCACCCGATTGCCGGCTAGCTCGCTAGCGCTGTGAAGCTTGCCAAGCCCTGGACGGCGAGACTTCACCGGAGGCGAGATCGACCTCCACCGGACTTGGTCTTCAACGTGTGGACCGAGCTATCGGCTGAGTCTCCGCCAATTCCTTTCGCTCATATCTTCTCGGCATCCGTGAACCTCCCGACCCATCTAATTGAACTATCTCGCCAGTGACAAGGCCGGTCTCCTCGCCGTGACGAGCATCCCGCTGCGCGCGTGGGTGCCGTGGACTCCGCCATCTCCGTGGTGTTCGCACAGTTGTGATCCTGAGACCTCCCCTTCGTCCTCGACCACTTCCACCGGAGCAACCTCGCCAGCAGTGGCCTCTTAGCCCTTTTCTCCGTGACTCTTCGTTTAAGGTAGAAGAAGGACCTCGGGTTAGGTTGGTAGAACAGGGAGTTTAATAATGTCGATGACTCATGTGAATAGTAACCTAGGCGGTCGCGAGAGCTTAGAAAATCGCCGAGACCCTCGTGCAAAGTGGATTTCCATTTAATCAATTCTGTTATTCTTTTAAAATGACCAAGAACTTAGAAAACCCATAACATGATGAAATCTTAATAAAAGTTGTCAAACCAATTTTGCTAGCTCACGAATATTATGATCTATCATTTAAAAATAGTAAACTCCATGCTTTTCTGTTCAAAATTTTAAAGTTTAAAATTAAAGACGAAACCCATAAACCTTGTTTAATTAAGGAAAATTAGTTTTTCTTACCATCTTGAACAAGAAAAATTGTAGATGTTTAAACCTTATTTAGACACTATTTAAAAATAGTAGGAACTGACATTGAAAAATATGATGTAGTTTTTCATTTAAAGCTAATTTGTCCAAAACTTAGAGAAAATCAGAAAGGCCTGAGAAATTAATGAGCAGTGATTAATAATATTTTCCTAGTTTACTTATGCAGCAGAGAACCTAGGAAAAATGCGAGAGACCATTAATTTGGACCGCTTTAAATTAAGATGATTTGTTTAGCCTCATATAGACTGAAAATCAATTATTAGAGTTACAAACTATAACAAAATGATTAATAAAAATCTAGTGGACTTATAACCACGAGAGCCTCACTACAAAATCTACAGAGCACCTCAACCCAACTTTTAAGTAAAAAATAAATACAAGGTGATAATAAGGCATTTTCCAAATAAATCATGAACAACC

>nano6

TACAGGCGGCTTATGCGGCCAGCGTGGGTCGGCCCAGCGTCGTGGGGAGGACCTTGGGCGTGTTGGAAAGACTTCGGCCACGGCTGGTGGGGGCATAGTAGGAGAATCGCCTTTAAAGGTGGGTGACCCCCTGGCGACCATGTCTTCAGCTCCCTTATGGTGTCTTTCCACCAGGAGCCCGGATGGGACACCGCATGATCCCTCGCCTTGTGCGTTGGAGAGCGCCTCCGTGGGAGTTGGTACCTTTAACCATCGTTCGGCTTCAGGATTTTTCATCAGCGGCGGCTGCACCCCTCTGGCGGTCACCCAAGATGGTGACCTCGGTTGATGGCGGGAAAGCAAGCAGGCTGCGGCCTCTGCCCTCCCTCGGCCTCAGGATTTTCATCGCCGGTCTTTGGGGAGGATGTGTCGGTGGGTCGGCCTGTGCGGCGGCGGCCCGCTCCTTCCCTCGGTGTCGAGGGGCGTTCGCCGTTCAGCGGCCCGCGGTACAGCGTGCAACTCCCGGCCGAGTGGCCGGGCGCTCCGGCGCCCGCCTGCCATACATGGCGGTGGAAGAGTTCTTCGCCGAAACAAGATAGCGGGAGCGCCGCGGGGAGCCTCCAACTCTGACGCTGCCGTCCTTGCCTCCGCGAGTTTGGGCATGGCGAGGCCTCATAGCGGCGACATCCACCGAGTCATCAGCTGCTGTGTTAAACCGGAGCCGAGAAATCGTCATCGTCGCGTATTGTTAGGCGGCGGCGGCAGCGGGTCGTTGGTCTTGTTGCTCAGCAGTCCCAATCGTGTGGGGTTGTTCCGTACCTGCGGAGGTGGGCAGATTCAGTTTGTAATGCACCTTGATATGCGGTCTTTTGTTCATTGTATGTATCGGGGCTGAGCGTGTATGTATTTTTGGCGCGAGCCGTGTTTTCCTTATTTTGAGCACTAAGACTACTTTTGTTGGTTATGCGAACCGCTTCACCAGAGTGAGTCGCCCGTACAAGGTGGCGAGTGAGGTGTCCGTATCCGGGCGTAAATCTCGGCTCACGATCGGCCTTCATTGTGAGCTTCTCTTGTAGTTAAAGAACCCCTCGTGCGCTCTTGATGGGCGGCGGCGAGTAGCGGTGTAAGCTGCCAGCGGCGAGTTGGCTCGCGAAAAGACTTGGTCGGCCGGGCTGGCCGGAGTCGTCGGGCCGGCCAAGAGTTGATCTGCTCCCAGGAGCCTGATGTCTTGATGCCGAGGGCGGTGGCGGGCCTCGGGTGACGGTAGAGCGCCTACTCGGCCGGATTCTTGGAAGACCTGGCGGCGATGGCGAGCGTGATGATGATCGTCTTGAGTGGAGATCCTCGGACCGCGTCGCAGTCAGAGGCTAGGTCGGACCTCGGTGAGGTGTAGTCGGCGCGAGTCTCTTCTGCTCCCTTCAAGCTTATCAAGATCAAACTGCGGTCGGATTATCTTGTAGTGTGCGTTTTACAGCGTGCCAGAGCTAAACACACCATCGTAGTGTTGTAAAGAACTTCTTTTCCTCTTGTTTTGAGTATCGGACACCGTGGTACGGGTTATGCGTGCGGCGGGAGTTGCTTTTCACGGAAGGTGGCCGGTGAGATGTCCAGTATCCAGTAGGAATCCCTCGGCTCGGTCGGCCTTGCTTCGTGCACTCTTGCCGTCCATGAGGTTACGCCGCGTAGCCTTGATCGAGAAGGCGAAGAATCGCAGGAGAGGGCTTCAGTGTGAAGACTTGTTCGGTCTGCAGATAAACATCCCGAGCGAGTTACTTATCAGAAGGTGATGGTGAGGTATCCGTATCCGGGCCGTAGGAGTCCTAAACGGTCGGCCTTGGTTGCGTGTACTCCGTCGTTTTGGATCCACTGAGTAGTCGAAAAGCAGAAAGATACATGAGAAAGGATCTTTTTTGAGAAAATTTCGGCAGAGGGGTTCCCCCTTTTAGCCCAGGAGGTCGGGCTTTGCGAGCAAGGTGACCCTTCATGATGACTAAACTGCGTGGGAGCGAGGTATATGAACAGCGAAGCATCTTAAGGGTAGAGCGGCGTGGTGTTGGATGTTCCAAGCGTTCAACGTGGACCTCGCCTATTGTTGGCGGCTTATGCGTTCAGACGAACTTTGGCGATAATGACGGCCCTTCCGAGGTAGCGTGAGCTTGTGCGCTCGGGCGTCTGTCGCGGCCGGCACCAGTGCGCCACGTGTGGAGGTCGGGACAGGACCCCTCGGGTGTGGTAGCGTCGCGGAGGCTAGCTGTACCGTCTTAGAATGTAGTAAGGCCATGTCGAGCCTCTTCCGGTGAGTCAGTCTTCTCGATAACTTGGTTGCTTTGGTGCGGCGTAGGCCCTCGTCCTTGGGGCCGTATTCTAAGTCTGTGGGCAAGATGGCCTCGGCCCCATAGACTAGAAAGAGCGGCGTGAAGCCGTGGCTCGGCTCGGCGTTGTCCTCGGACTCTAGACCAGGGAGTTCCTTCATCGCCGGCAGAACTTTGTTGGGGTCGTTGTAGATCCGGAGCAGGTCCTTGTAGAATCATGCGGTTACGCTCTACTTGCCCATTCGTCATGGGGTGAGCCACGGCGGCCCAGTCCACCGGATGTGGTGATCTCGCGAAGTCAGGAACTTTGCCGGTGAGCTGGTGCAGATTATTGATGATGGGTTCGGGACACAGAAAGGTAATGCCTTTGGGCCATTTCTAAGTATTTTGGTGATTGAGTGCAAACACAAGTGCTTAAATGTGAAAATATGCCCAGGATGAACAAAATGCAAATCACAAGTTAAGGTATGTTTCTAAGCCTTAGTACATTGGTTTTGTGTACTAATATATTTGTCTAAGTGTTAAAAGCAGATAGAAGAAGAGAGAAGACTTGGTGTGTGCACGGCGAGAACTCCTTGATGCGGAGACGCCGGATGTCGGTGGTGAACCGGTGTCGGTGGTGCCAGGGCGATCCGTGCGCCAGGCTACCTCGGCCGAAGAAGGCGGCTCGGGATTTTCTCCGCGACCGGCTAAAATTCAATTTGTCCGGTGTGCCGGGTATCCGTTGAGCCATGATCGGCCGGGCCAGCAGTCGGCCCGGCGATCGGCGCGCGACGCGTGGCGAGGCAACGGTCGGAAAAACAGCCGGTGTCGGTGTGCGGTGCTTCGGTGCGCGGATCTACAGCGATCGACAGCGGTCGGCCGCCATTTAAGGAGACAAATCGGGCGCCGGACGGTGTCGGTGTGCACCAGTATCCGGTGCGCCGCAGGAGCGAGAAAGGCAAAGATGGCCTTCAGTTTATTCCCAATGGCTCCTTTGCCTTGGGGCTATAAAAGGACCCCTAGATGGAGAGTACACAAGCATTCCTACAACTCTTCTAAGCACCAAGACATCAATCTCACGCGTTCGTTTCATTGTGATAGCATATAGAGCTCTTGTGGAGTTGTGAACTCTTTGTGTTGCGTTGCGAGCTCTTGTTGCGACTTGTGTTGCGTGTTGTTGCTCTGATTTTCGAGTCTTGTGTCGTTGCTCATTCCCTTACTCATATTTCTTTGTGAACTCAATTGTAAGGCGAGAGACTCCAAGTTGTGGAGATTCCCATAAGCGGGAAAAGATCAAAGGAAAGAAAAACACGTGGTATTCAAGTTGATCATTGGATCACTTGAGAGGAGTTGAGTGCAACTCGTCAGTTGGGGCGCCACAGCGTGGAGTAGGCAAGTTTGTGCCAGTAGAACCGGGATAACCAGTGTCAACTCTGTGATTGCTTTCTTGTGGTTATTGTGTTTGAGTTCTCTCTAGCCACTTGGCCATACTTGTACTAACCCTTAACAAGTTTTTGTGGCTTAAGTTTAAGTTTTACAGGATCACCTATTCACCCAGGTGCTCTCAATTGGTATCGGAGCCGTTCTCTTCAAGAAAGGACTGGCGAAGAGATGGATCCTAGGGAGGGAATTGTGATCAGCGACAAGGAAGGAGTCCGTCAGCGACCAAGGGATGACAAGTCCAATGACTCGGGCACCGGCCTAAGAGATGGGAAGAAGACAAGGCGCATCAAGGAGATCGTCTACCTACGACGGCTGATGAGTCCTCTTCTTCCCAAAAGGGCGGCGACCACGACAAACAAAGGAAAGCAGGTTAATTCTAACTTTTCTTTTGACTACTCGTATTCATAAAGTTCAAATTCACATTGCTTTCTATTCCACTAAGCAAGCCCCACACTTTGATGGGAGGACTACGGATTTTGGAGCCACAAAATGCGTGATCACTATCTCTCCATCTAGCATATGGAGATTGTAGATAGTGGAATGCACTTTAATAGTTGGATAGTCTATATTCATTAATGAGCAAATCCATAAGAATGCACAAGCACTACTCACATTCTTCTAGCCTCATTGTGCGAGGATGAATATAATAAAGTGAGTGGCTTGGATGCATGCCAAGCAAATCTGGATACCTCAAGATCTCATGAGAAATGATGCTACCAACTCACCAAAATGGAGTGGTAGAGGCGAGCTTGGGATGATTCGCGATGATAAGGGGCGAGGAGCCAACTCAAACATACAAGCTCAAGACCCTTGTCAACAAGATAAGGAGCTATGGAAGCGCAGATGGACCGGACCGGCGTCGTCGCCTCATGCTTGGAGCTCTCTTCTTGATCCTCATTTGGTGAATAATATTCGCGAAATCCGGTACACCAAAATGTCAGGGAAATTCTTGGAAAATTTGTAGCGGGCGGATGATGATCAAGGAGGCAAAATACGTCGATGACGCGTTGACGGTCAATCCACGAGCCTCAACCCATTGCTCTCAAGGCATCGAGGCAGGAGGCACTACAAGCAAGGTGGTGCAAATTGAGTAGCGGACTTAATGATGAAGAAATGGCTCTCATCATTAAGCGCTTCAAAACAGTGCTAAAGAGTGCAATGGACGGTAGAGCAAGACTAAGACCAGAAAGCGATCATGCTTCAAATGTGCGGTAAGCTTGGTCATTTATTGCTAAGCTGTCGATAATGAAAGTGACCAGAAAAGGGAAACAAGAGGAAAGAAGAAGCATTATAAGAAGCCAAAGGGTGAGGCGCATCTAGGCAGGAGTGGGACTCGGATTGCTCTCGTCAGGCTCGGGCAATGAAGGACTACGCCGCCACAGCCTTCACAAATCAACCCTCTTCCCAAGCAGGCGTCACACATGCCTTATGGCAAGGGAAGAAGGTATGTACTCGCAACTCTACCTATGCTTCTTCAAGTGAGGACGAATCTAGTGATGAGGATGAAGTAGATTATTCATGTTTGTTCAAGGGCTTAGATAGATCTAAGATAGACAAAATTAATGAATTAATTGATGCCTTGAATGAAAGAATATACTGAAAAGCAAGAGGATTTGTTGTATGAAGAGCATGATAAATTTGTTGAGGCACAAAAATCCTATGCTTTAGAAGTTAAAAGAAATGAAATGCTTTCTTTTGACTATCTACTTGTCATGAAACCATTTCTACTTTGAAAGGTGTCAACAATGATTTAAATGCTAAATTAGAAGTAGCAAATAAATCCAATTCTTGTGTAGAACATGTTGAAATTTGTACTAGGTGTAAAGATTTTGACGTTGATGCTTGTAGTGAACACCTAGTTTCAATTTCCAAGCTTAATGATGAAGCTAGTCTTAATGCTCAACTTAAGACTAGCAAGAATATTCGATAAGCTAAATTTGCAAGGGATGCCTACACAATTGGTAGACACCCCTCAATTAAGGGATGGACTTGGCTTCAAGAGAAGCTAAGAACAACAAGCCATAAGGCTCCCATTCGCCAAGGAGAAAGGGAAGGTCCCTATGGCTAATGTGTGCAAGAACCATGCCTTTTGTATCATGATAGAACAAACTAGAAATGTAAGTCATGATGCTTTTGATTCATATGTTTATGATTCTCATGCCATGTTTGCTCCTAGTTCCTCTTATGTGTATGATAGAAATGTTACTAGAGAAATGTTGTTCCTAAAAGAAATGCTATTCATCATGTGCCTAGAAAGAATGTTATTCATGCTCCTAGGAAAGTAGTGAATGAACCTTCCACAATTTATTATGCTTTAAATGCTTCCTTTGCTATTTGTAGAAAGGATAAGAAAATTGTTGCTAGGAAGTTAGGGGCAAAATGCAAGGAGACAAAACTTGCATTTGGGTCCCTAAGATATTGTGCTAACCTTGTAGGACCCAACATGAGTTGGGTACCTAAACCAAGCCTAAATTTGCCTTGCGAGTTTATGCATCCCAGAGTTCAATTTGGATTATCGGCAGCGGATGCACAAACCATATGTGAGGGAGAAGAAGATGTTCACCTCCTCGTAAAAATAAGGATTCAAAGATTCAATTATATTCGGTGATGGGAATCAAGGCAAGGTAAAAAGGTTAGTAAAATTGCGATTTCTAATGAGCATTCTATCTCCAATGTATTTTTAGTAAGAGTCTTGGATATAATTTGCTATCATTAGTCAATTATAATAATATAGAGTATATTGTCTATTTACAAAGATACTTGTCTTTAGAAGAAGTGATGGTTCACTAGCTTTTAAGGGTGTATTAGACGGCAAACTTTATTTAGTTGATTTTGCAAAAGAAGAGGCAGTCTAGATGCATGCTTATGGCTAAGACTTATGTAATTGTTGTGGCATGTGCTTAGCACATGTGGGGATGAAGAACCTTCACAACCTAAAGAGTGCGATAAGTCCAAACTATCTTACGAAAAAGATAGACCTTGTGCGGCTTGTCAGGTAAACAAGTGGGAGGAGCACATCAACAGAGCGTGATGACCACTTCAAGACAAGCTCTGGCATATGGATCTCTTGGACACATGCGCCTATCTCGAGCATAGGAGGGAGTAAGTATGGTTTAGTTATTGTTGATGACTTTTCCTTCACTTGGGTGTTCTTTTTGCGGATAAGTTCAAACCAAAGGACCCTCAAGCGCTTTCTCGGAGAGCTCAAAATGAGTTTGAGCTCAAGGTGAAGAAGATAAGGCGACAATGGGTCGGGAGTTCAAAACCTTCAAGTGGAGGAGTTCCTTGAAGATGAAGGATCAATACGAGTTCTCCGCTCCTACACACCACAGCAAAATGGTGTGGTAGAAAGGAAAAAACAGGGCGCTCATCGACATGGCGGGGCGATGCTAGGGAGTTCAAGACCCAGAGTGCTTTTGGATGAGCGGGTGCCCCCTTGCCACGCCATCATGGGGTCTACCTTCATCGCCTCCTCAAGAAGAGCGTCGTATGAGCTACTAACAGTAACAAACCCAATGTATGCTTCGTGTATTTGGGAGTAAATGCTACATTCTAGTGAAGAAGGGTAGAAATTCTAAGTTTGCTCCCAAAGGCGTAGAAGGGTTTTATTAGGTTATGACTCAAATACAAGGCGTATAGAGTCTTCTCAAATCATCGGGTTTTGGTTGAAGTCTCTAGCGGCGTTATTTGATGAGACTAATGGCTCTCAAGAGAGCAAGTTGTTGATTGTGATGATGTAGATGAAGATGTTAGACTAATTTGCTATATCTGAACCATGGCGATTGGAGAAGTACAGGAACAAGATAGAATCAATCATCTTCCTCAACAATGATGCAAACCCCAACTCAAGTGGGGCGCTTGATCAAGGGGCGCAAGATGATCAGTGATGGAGGAAGAAGCGCAACCAACACCTCAACCAGATTCGGATGATTCAAAGGGATCATCCGTCGACCAAATCACAGGTGACATTAGCAAGGGAGTAACTACTGATCTCGTTAGTTAATTTTGTAGGCATTACTCTTTTGTCTCTTCTATTGAGCCTTTGGGTAGAAGAGGCCTTGCTAGATCCGGACTAGGTGTTGGCCATGCAAGAGGTTAAACAACTTCAAGCAATGAAGTTTGGACACAGTGCCTCGTCAAAAGCAAAATGTTGTAGGAACCAAGTGGGTGTTCCATGACAAGCAGGGCCAGCACAGGGTGGTGACGAGGAACAAGGCTCGACTTGTGGCAAAGGTTATGCCCAAGTCGCGAGTTTGGACTTTAGAGAGACTTTTGCTCCTGTGGCTAGGCTAGAGTCCATTCAGTATTTTGCTAGCATATGTCGCTCACCATTCTTTGAGTTGTTCCAAATGGATGTGAAGCGCTTTCCTCAACGGCCAATCAAGGAGGAGGTGTCGTGGAGCAACCCACTGCCGGGATGAGCAATGAACCGGAAACCATGTGTGTAGCTCTCTAAGGCTCTTGGACTTAAGCAAGCCCCAAGAGCATGGTATGAATGCCTTAGAGACTTTTACTTGCTAATGCTTTCAAGGTTGGGAAGAGTAGATCCAACTCTTTTACAAAGACATGTGATGGTGATTTGTTTGTGTGCCAAATTTATGTCGATGACATAATATTTGGTTCTACTAACAAAAGTCTTGTGAAGAGTTTAGCGAGGTGATAGCGCGGTCGAGATGTCAATGATGGGCGAGTTGAACTACTTCCTTGGGTTCAAGTGAAGCAACTCAGGCGGCACCTTCATCTCCCAAGCGAAGTACCATAAGATCCTGTAAGCGGTTTGGGATGAAGGGCGCCAACCCGCAAAACTCTGATGGGGACCAGCGGACACACGAGCCTCAACAAAGGAGGTAAGTCAGTTGATCAAAAAACATCTTAGTCAATGATAGGTTCTTTGCTTTACTTATGTGCTAGTAGGCCGGATATTATGCTGCGTATGCATGTGTGCTAGATTTCAATCCGATCCTGGGAATGTCACTTAGTAGCGGTGAAGCGAATTCTTAGATATTTAGTTACTCATGTGCTTGGGCTCTGGTATCCAAAGGTCTACCTTTGACAGTTGGATACTCGGACTCAAGCTATCTTTGGATGTAGTCGATAGGAAGAGCACATCGAGGGACGTGCAATTCTTAGGAAGGTCCGAGTGTCATGGAACTCTAAGAAACAAACTTCCAGTTGCCCTATCCACCGTGGTAGTATGTTCTTGCGGGGACGGTGTTCTGGCGCCTACTTTGGATGAGGCAAACCCTCGAGGACTTTGGCTACAATCGAGCAAAGTCCCACTCTATGTGACAATGAGAGTGCTATCGCGCGGCGGAAGATCACAGTGAACACGGTGCACCATTACAGACATCAAGCATCACTTTTGAGAGACCACCGGCAAAGGGAGATATCGAAGTGTTTCATGTTGCACAGAGAACCACTCCTAGATATCTTCACTAAGCCTCTAGATGAGAAGACCTTTTGCGGTTTGTAGTGAGCTAAATGTCTTAGATCGCGGAACTGGATTGAATTGTATACATGTAGTTATGCTTTTGATCATGTTCCTTTTGCATTATGTTGCTTATTATGGTGCTCAAGTTGTACAAACACTCCTGACCTACAAGTCCGTTGCAAAGTGATGCACGTTTAGGGGAGATGTGTTACAACTTGACCCTTTGAGATAACCATTTGCTTGAGTTTGCTTGATTTAGTCTCGAAGAAGAGGATTGAAAGGGAAAAGGTGGACTTGGACCATGAAAGACTTCCTTGCACTCGATGAGAGATGCATAATTCCAAGTTCATCTCATGAAATCTTATTGCCATTTGCTCTTAATTGAAGACTTTGGTGAGCAATGGGGTTAAAAGGCCAAGATTAATCCGTTTGGTGCTTGATGCCAAGGGGAGAAAATAAAGCCAAAGTGATAAATGGATCAACTACCACTTGAGAGATTTTGAAAATAGTAGAATAGAGTTTTGTTTTGTCAAAAGCTTTTATTGTCTCTTATTGTCTCTATTGTCAAAAGTTGGCTTCTTGTGGGAGAGTGTTGATTATGGAAATAGGGGAGTTTTGAAATCTTTGATCAATCTCTTTTGGAATGACTCTCTTTATACTTCATCATGTGTGTTTGACTTAGAGATAGAGATTTGAGTTTGATTTGCAAAAACAAACCAAGTGTTAACAGGGATGATCCATATATGCAAAATTGATCAAAACCAATTTGAGTTTTTATTTGAAGTGATTTTGCACTTGTGCTATCTACTTTATGTTGTGTTGGCATAAATCACCAAAGGGGAGATTGAAAGGAAATGTGCCTTTGGGCCATTTCTAAGTATTTTAGTGATTGAGTGCAAACACAAGTGCTTAAATGTGAAAATATGCCCAAGGATGAACAAAGTGCAAATCACAAGTTAAGGTATGTTTCTAAGCCTTAGTACATTGGTTTTGTGTACTAATATATTTGTCTAAGTGTTAGGGATAGAAGAAGAGAAGAAGACTTGGTGTGTGCGGCCAAAAGTACTCGATGCAGAACGCGGTGTCGGTGGTGCCGAACGGATGTCGGTCTTGCCGACTCCTCGGCCAGAAGGCGCCTCTCAAGTTCTCGGCGACTTGGCTTAAATTCACGGATGCCGGTGTGCCGAGCTATCCGGTGAGCCGCGTCAGCCAAGATACGATCGGCCGTAGCGATCGGCCCATGCGACCGTGGCCGAGCCAGCGGTCGGAAAAACGGGTATCGGGTGTGCCATTGTCGGTCGCGCGGTCTGCGATCGACAGCGGTCGGCCGCCATTTAAGAAACCAATCGAACACGGGCGGTGTCAGTGTGCCGCCGGGCCGTCCGATCTTACGAGGCATGAAGGCAAGATGGCCAGAGTTTGTTAAGCCTAAATGTTGGGGCTATAAAAGGACCCTAGGCGCATGGAGAGTACACCAAGCATTCCTACAACTCTTCTAAGCACCAAGACATCAATCTCGCATTCGTTTCATTGTGATAGCATATAGAGCTCTTGTGGAGTTGTGAACTCTTTGTGTTGCGTTGCGAGCTCTTGTTGCGACATGTGTTAGTGTTGTTGCTCTGATTTTAGTCTTGTGTGCGTTGCTCATTCCCACCTTACTCATATTTCTTTGTGAACTCAATTGTAGGGGAGAGACTCCAAGTTGTGAGATTCTATAAGCAGAAGAATCAAAGAAAGAAAAAACAAGTAGTATTCAAGTTGATCATTGGATCACTTGAGAGGAGTTGAGTGCAACTCTCGTCAGTTGGGCGCCTGGCGTGGGTAGGCAGAGTTTGTACTTGGCGAACACAGGATAACCACCGTGTCAACTCTGTGATTGCTTTCTTGTGGTTGTGTGTTTTGAGTTCTCTCTAGCCACTTGGCCATACTTGTACTAACCCTTAACAAGTTTTGTGTGGCTTAAGTTTAAGTTTACAGATCACCTATTCACCCCCTCTAAGTGCTCTGGGACCTGAAGCGATGGATGATGTTGGTGAAGAACGCCACCGCTTGTTCGGACGATCTTTGTTTGAGGTCGGACCTCGATCCACTTGAGAATTTGTCGATGGTGACCAGCAGTGCGTGTAGCCGGTGCCTGCAAAGGGCGGCGAGATCGGACCACGACAAAAGGCGGTGATGGGTATCGTCTGCGGGCTGGCGGCGGCGGTGGAGTGCGTAGGCCCGACACCCTTCGCGAGTGCGGACAATTCTAGTGGCGTCGGCCACCATGCTTCTTGATGAAACCTGGAAGGCGTTTCCAACAAGGGCTGAGGTCTTCGCGTGATGGGCACCAGCCCAGTGTATCTCTTGTAGGAGCTCGGCCTTGGCGATGGAAATGCATCGCTGGAGGATGCTGAGGGCTACGGTGGTAGAACTCTTTCGCGTCCCCACCAAGACAAGCGACTTGTGCGCGCCAACCGTAGAGCTTCGGCTCGGTCGAGGTAGCTCTCCTCGGTGGAGATATTGCATGCGGGTGCACCAGGTTCGATTGGCGTGACCCAGCCGCTCCTCTCGGCGTCGTGCCTTATCTCGGCGAGGCGCGGGGCGGCTGAGTGTGCCTGGGCGAGCAGTGCCTCGGATGGCTGGCCGAGGTGCCGGTGGTGAAGTGCCTCGGCTTCCGGTGTCGTCGATCTTGGCGGAGGGTTGATGCAGGTCTCGGGAGAAGGCGTCGGAGGAACCGTTATTCGCCGAGGCTATTTTGTGGTGTAAACAAATTGGCGCTTAGACAACAATAACATTGCGGACGTTTTAATGTACTGAATTACTTGAATTAATTCGGGGATCTGGATTTTAGTACTGGATTTTGGTTTTAGGAATTAGAAATTTTATTGATAGAAGTATTTTACAAATACAAATACATACTAAGGGTTTCTTATATGCTCAACACATGAAACCCTATAGAACCTAATTCCCTTATCTGGGAACTACTCACACATTATTATGGAGAACTCGAGCTCCTATTAGGCGGTGGCCTCAAGCGTACTCGGCGGCGCGAGGGCCGAGGTGAGCGGCGAGAGTGAGACTTGCGGATGTGGTGTGCGCGGTGATGCGGATGGGCGTCTGCGTGCGGAGGCCGAGGAGGGTGAGAGCATGATCATGCGGTGAGTCGCCGTGGCCGTCGTGTGATGCCACCGGCGAGGTGAGCGAGAGACCGGTCACGGAGAGAGTCGCGGTCTCGGCGTCGCGAGGCCGAGGCGCTCAAGCTCAGCGTGAGTGTGGATGCGGTCGCCTCCTTGAGGCGGAGAGTGGCGTTCTCCCAGGTGGTGTCGCCCTCGGCCGAAGGCAGCAGCGGCGGTGAGGCTTGCACGGCGTCGGTGAAGGAATCGCCGTCGCGAGTCACGCGGCGTGGAGAGGCGTACCGCGCGCAGTGAGGTATCGCCCTCGCGCGACGATGTCAGCGCCCATCTCGCGGAGCACGTTCACGGCCTCCTTCTCGCCCTGGAGGTCGTGCTCGCGGAGGTTAGAGGCGCCTCCTGGGAGGAGGGCAGCGGCGGTGAGGATAGCAGCGGAGCCGGTGAGTCGCGCCGGCACGAGCACGCGGCCTGGGCGGTACTTCTGGCCACCGGGATGGAGATCGGCGGAGGTCGTCGGAGGCAGTGGCGCGCACGCCGAAGTCAGAGAGGGTGTCGAGTGTCTGGCAGGCGGGCGTGGGACTTGATGTCGCCGGTGAGGCGGAGTTCGAGTCCGTCCGGGAGGAGGGCCGAGGAACATGAGGCGGAGGCGTACTGGGAGGAGCGCTCGGCGGACACCTCCACGGTGCCACCGCGCACTGGGCCGGACACGGAGATCGGGGCGACCGTCGTTGGAGGACACCCAGGCACCGAGGCGTTCGAGGGCCTCAGGAGGTCGCCCTGAGGGCGCTTGCCGAGGAGTCCGGGTAGTCGGTCACGAAAGTGGTGCCAGAGGTGAGGCGGCCACGCCCATGAGGAGGCGGGCCACCGCGGCAGCGTTGCCTGGTTGAGGTCACACCGGCCTGTGGGCGAGCGCCGAAACCGCGGATCACGGCGTCATCGCCACAAGCTCCACGCCAGCGCCCCAGTCGCGGAGGCGGAGCATGGCCTCGGCGTCCTCAGGGTAGCCACGCCCACCACGCGGGTCTCGCCCTCAGCGAGGGCAGCGGCGAGGTAGCGAGTGGTGTGGTTCTTGGATGGCTGAGCGCGAAGCTCGCCGCGGAGTTCGCGAGCTGGATGCACGATCACGTCGAAGGTGGCGGGCAGGGCGTCGGATCCTTGCGGGGATCGGTGGGGCCCCACGGCCGGAGCAGGGTGGCCGGGGAGCCATCGGCATGGCGGGTGACGCCGCTGAGCACCTGATGGGCGCGGCGAGGGCGCGGCGGGTGGCCAGGAGGTGCGCGCCTCGCCTTGGGCGCAGCGGTAGTGGCGCCAGTGAGCGCGGTAGACGCGGCGGCGGCGGTGGCCATGGTAGATCCTCTAGAGTCGACCTGCAAGTAACGCAAACAACAGGGTGAGCATCGACAAAAGAAACAGTACCAAGCAAATAAATAGCGTATGAAGGCAGGGCTAAAAAATCCATATAGCTGCTGCATAATGCCATCATCCAAGTATATCAAGATCAAAATAATTATAAAACATACTTGTTTATTATAATAGATAGGTACTCAAGGTTAGAGCATATGAATAGATGCTGCCATCATGTATATGCATCAGTAAAACCCACATCAACATGTATACCTATCCTAGATCGATATTTCCATCCATCTTAAACTCGTAACTATGAAGATGTATGACACACACATACAGTTCCAAAATTAATAAATACACCAGGTAGTTTGAAACAGTATTCTCTCGATCTAGAACGAATGAACGACCGCCCAACCACACCACATCATCACAACCAAGCGAACAAAAGCATCTCTGTATATGCATCAGTAAAACCCGCATCAACATGTATACCTATCCTAGATCGATATTTCCATCCATCATCTTCAATTCGTAACTATGAATATGTATGGCACACACATACAGATCCAAAATTAATAAATCCACCAGGTAGTTTGAAACAGAATTCTACTCCGATCTAGAACGACCGCCCAGCAGACCACATCATCACAACCAAGACAAAAAGCATGAAAAGATGACCCGACAAACAAATTGCACGGCATATATTGAAATAAAGGAAAAGGGCAAACCAAACCCTATGCAACGAAACAAAAAAATCATGAAATCGATCCCGTCTGCGGAACGGCTAGAGCCATCCCAGGATTCCCAAAGAGAAACACTGGCAAGTTAGCAATCAGAACGTGTCTGACGTACAGGTCGCATCCGTGTACGAACGCTAGCAGCACGGATCTAACACAAACACGGATCTAACACAAACATGAACAGAAGTAGAACTACCGGGCCTAACCATGGACCGGAACGCCGATCTAGAGAGGTAGAGAGGGGGAGGACGAGCGGCGTACCTTGAAGCGGAGGTGCCGACGGGTGGATTTGGGGAGATCTGGTTGTGTGTGTGTGCCTCGAACAACACGAGGTTGGGGAAAGAGGTGTGGAGGTGTCTATTTATTACGGCGGGCGAGGAAGGAAAGCGAAGGCGGTGGGAAAAGTCCCCGTAGCTGCCGGTGCCGTGAGAGGAGGAGGAGGCCGCCTGCCGTGCCGGCTCACGTCTGCCGCTCCGCCACGCAATTTCTGGATGCCGACAGCGGAGCAAGTCCAACAGTGGAGCGGAACTCTCGAGAGGTCAGAGGCAGCGACAGAGATGCCGTGCCGTCTGCTTCGCTTGGCCCGACGCGACGCTGCTGGTTCGCTGGTTGGTGTCCGTTAGACTCGTCGACGGCGTTTAACAGGCTGGCATTATCTACTCGAAACAAGAAAAATGTTTCCTTAGTTTTTAATTTCTTAAAGGTATTTGTTTAATTTTTAGTCACTTTATTTTATTCTATTTTATATCTAAATTATTAAATAAAAAACTAAAATAGAGTTTTAGTTTTCTTAATTTAGAGGCTAAAATAGAATAAAATAGATGTACTAAAAATTAGTCTATAAAACCATTAACCTAAACCCTAAATGGATGTACTAATAAAATGGATGAAGTATTATATAGGTGAAGCTATTTGCAAAAAAAGGAGAACACATGCACACTAAAAGATAAAACTGTAGAGTCCTGTTGTCAAAATACTCAATTGTCCTTTAGACCATGTCTAACTGTTCATTTATATGATTCTCTAAAACACTGATATTATTGTAGTACTATAGATTATATTATTCGTAGAGTAAAGTTTAAATATATGTATAAAGATAGATAAACTACTTCAAACAAGTGTGACAAAAAAATATGTGGTAATTTTTATAACTTAGACATGCAATGCTCATTATCTCTAGAGAGGGGCACGACCGGGTCACGCTGCACTGTAGGCATGCGTCGAGAGATAGATTTGTGAGAGACTGGTGATTTCAGCGTGTCCTCTCCAAATGAAATGAACTTCATATATAGAGGAAGGGTCTTGCGAAGGATAGTGGGATTGTGCGTCATCCCTTACGTCAGTGGAGATATCACATCAATCCACTTGCTTTGAAGACGTGGTTGGAACGTCTTCTTTTTCACGATGCTCCTCGTGGGTGGGGGTCCATCTTTGGGACCACTGTCGGCAGAGCATCTTGAACGATAGCCTTTCCTTTATCGCAATGATGGCATTTGTAGGTGCCACCTTCCTTTTCTACTGTCCTTTTGATGAAGTGACAGATAGCTGGGCAATGGAATCGAGGAGGTTTCCCGATATTACCCTTTGTTGAAAAGTCTCAATAGCCCTTTGGTCTTCTGAGACTGTATCTTTGATATTCTTGGAGTAGACGAGTGTCGTGCTCCACCATGTTCACATCAATCCACTTGCTTTGAAGACGTGGTTGGAACGTCTTCTTTTTCTGATGCTCCTCGTGGGTGGGGTCCATCTTTGGGACCACTGTCAGCAGAGGCATCTTGAACGATAGCCTTTCCTTTATCGCAATGATGGCATTTGTAGGTGCCACCTTCCTTTTCTACTGTCCTTTTGATGAAGTGACAGATAGCTGGGCAATGGATCCGAGGAGGTTCCCGATATTACCCTTTGTTGAAAAGTCTCAATAGCCTTTGGTCTTCTGAGACTGTATCTTTGATATTCTTGGAGTAGACGAGTGTCGTGCTCCACCATGTTGGCAAGCTGCTCTAGCCAATACGCAAACCGCCTCTCCCGCGCGTTGGCCGATTCATTAATGCAGCTGGCACGACAGGTTTCCCGACTGGAAAGCGGGCAGTGAGCGCAACGCAATTAATGTGAGTTAGCTCACTCATTAGGCACCCAGGCTTTACACTTTATGCTTCCGGCTCGTATGTTGTGTGGAATTGTGAGCGGATAACAATTTCACACAGAAACAGCTATGACATGATTACAGATTCGAGCTCAGTACCTGGATTTTGGTTTTAGGAATTAGAAATTTTATTGATAGAAGTATTTTACAAATACAAATACATACTAAGTTGTACAAAACCAGCAACTCACTGCACTGCACTTCACTTCACTTCACTGTATGAATAAAGAGTCTGGTGTCTGGTTCCTGATCGATGACTGACTTCTCCACTTTGTGCAGAACAGATCTAGAGCTCTTAGTAGAGCGGGAGATGTTGGTCGGCACGAGCATGATGTTCATGAGGTCGAACTGTGTGCCAGAGTTGAGGGTCACGTTGATGTCGAGCGGCACGTCGGAGTTGGAGGAGGCCACCACGTTGCCGATGTTGATGTCGGAGAAGCGAGCACCGTTGTCGTTCACGCCGTCGTTGTTTGTGGTGGTGTTCACGTTTGTGGCGGTGTACACGGCCGTTGATGGTCACGCGGATGGTGGAGTTGCCGATGGAAGACACGCGGAGGTAGAGGTTGTAGGGTTGCCGTTACCGCGGAGGTGTAACGAGCGGTGGTGTTGTTCTGCTCGAAGCGGAGAGAGTCGCCCTGGTTGCCGAACTTCTCGGAGATGAAGGTGCGGGTCTGGTTGTTCACCTGGGTAGCGTGGATCGGGAGATGGTGAAGCCGGTGTAGTCGTTTGGGCGAGGTGGGTCATGGAGCCGTTCTCGTGCACAGCGTGGATGTTCTTGCGGTTGTGCACGGACACCATGTAGCGCGATGCACCTCAGGGTGCCGGACGGGGAGGCGATGTTGCGGATCTCGTTGTAGTGGAGTGGGCGACGGAGGTCCTCGTTGCGCACCACGAGCGGCACGCCGGAGATGTTGCGGATGAAGTAGTCCGGGAAGTAGTTGGAGTTGCCGCGTGCGGTGAAGGCACCGGAGCGGAGGCCGAGGTGGTCTCGAAGGACTCGGTCTGCCAGTTGGTCACGGTAGCCACGCCCTCGCGGTCGGGCCGGAGTCGAGCCAGGAGCGCACGAACGGTGTGAGGCGGAGGGAGGAAGGTAGAGCAGTTGAAGTTCTGGTTGAACGGGAAGCGCCGATGTCGCCGGAAGAGATGCCACCGGGTAGTTCACGCGAGCGGCGAGGAGGGCGTGGGTAGTTGTGGAGCCAGGAGGCCCACGATGTTCGGGAAGGTGTTGGAGAGGCGGGCGCCGGAGAAGCCGTTGAGCACGTAGTTGGAGTTCACCTGGAAGAGGAGTAGAGGAACAGCCAGTCCTGGGAGGTGAAGGACTGGGTCTGCTGCGGGCCGGAGCCGGAGGCGTAGAGGTTGGCGCCGGAGGACACGAGGAGGGACTGGTACTTGAAGAGGGACCAGATGGACACGTACTCGAACACGTTGAGGAACATGTAGGTGCGGAACTCAAGCATCTCGTGGAGAGGGTGTTGAGCTTCTTGAAGGCTGTCTGGTAGGTGTTGATGCAGTAGTTGGAGTACTCCTTGGTGTAGTCCTTGAGGTAGCCACGGTAGGTGCGGAGTGGCAGCGGAGATACCCACTCCTCTGCGTTGAGGATCACATCGCGGATGAAGGAGAGGTGGAGGTTGGCGGCCTGAGCGAGTGGAAGGAGAAGGAGTTCGTGTGCGGAGCTGGAACTGGGTGAGGCGGTTGAGGAAGAGCTGCTGCATGGTGTTCACCGGGATGTGATGGAAAGGAGAGTCGGGTTACGGTTCGGGTTGAGGAAGTTGTCCACCTGACGGTTGAACTCGGCAACGTTCTTCTGGAGGCCCTCAAGTTCGGCGTTCACGCGGGAGAGGGTGTCAGCGTTGAGGCGCTGGTTGAGGAACTTCTCGGTCTCACGGAGGATGTCCTGCATGAGCTTGGTGTTGTCGTTTGGGAACACGAGGTCCCAGAGGCCATTGAGGATGCGCTTGGCAGCGAAGCCTCCGAGCTTCTTGAGGAGGAAGATGGCCACAGTACCCACCACAGGAGCCACGTAGAGTGAGTGGTCGTCGCGCTTCCACTCGGTCCACTCCTTCTGGATGGTGTCGAGGACTTGTACTGGAAGAGAACGGGTCCTGGGCCATCACGTTGTGGCCGTCGCAGATGGTGGTGCGTCCGGAGTTGAGCACGGAAGCTCCTCCACCCTTCCGGGCCTCGCACCTGATCGATGTGGTAGTCGGTCACGTCGGTCTTCAGGCCGATCTGGTTGCTGCTGGTGAACAGCTCGTTCACGGCCTTCTGAGCCCTCTCCAGGTCGTACTCGGCCTCGAAGGTCACCTCGGCGGGCACGAACTCGATGCGGTCGATGTACACCTCGTTGCCGCTGTTGAACACGTGGGCTCAGGGTGAACACGCTGCTGCCGTTGCTGAAGTTGAAGGGGTGGTGAAGCCCACGGTGCGGAAGCTGCCGCTCTGCAGGTTGCTGCCGCTGCTCATGGTGGCGCTGAAGTTGCCCTGGTTGATGGGGCGGCCGTCGATGCTGGTGTGGAACTGCAGGTTGGTGGTGCTGGCGTAGCGGATGCGGACGCGGTAGCGCTGGCTCAGGGGGCGGTGATGTTCACGCGCAGGGTGCTGATCTGGCCGGGGCTGGTGCGGCGCAGGATGTCGCCGCCGGTGAAGCCGGGGCCCTTCACCACGCTGGTGCCGCTGCCCAGGTTGGTGCTCTTGGTCAGGGATCTGGGTGATCTGGCTGCTGGGGATGATGTTGTTGAACTCGGCACTGCGGTGATCCAGCTGAACATAGGTGCACGGATGATGCTCACGCTGCTGTTGCTGAAGCCACTGCGGAACATGCTCACGTGGCTCAGACGGTGGCTGAAGCCCTGTCGAGGTGTAACGTTGTTGTTCTGAGGGGATCTCGTCCAGGCTGTCCACGGTGCCGCTCTTGCGGTACACGGCGCTGGGCAGGTTGCTGCTGGTGCCGTAGGCGAACTCGGTGCCGTCCAGCACGCTCAGCTGCTGGTTGTTGATGCCGATGTTGAAAGGTCGACGGTACAGGGTGCTGCTCAGGGTGTACACTCCTGGCCCAGCTGTGCCACGATGCGCTGCTGAGGTGCAGCGTTGCCCATGGTGCCGTACGGGGAAGGTGAACTCGGGGCCGCTGAAGCCGACGGGGCTGGCCGCGATCTGCGTGGCCGCTCCAGTAGTACTCGCCGCGGTGGGCGTCGGTGTAGATGGTGATGCTGTTCAGGATGTCCATCAGGTGGGGCTGCGGATGCTGCCCTCGATGCCCTAGGCGCTGCCGCGGAAGCTGCCGTCAGGGTTCTCCAGCACGGGGTTGGTGTAAATCTCGCGGGTCAGCTGGCTCACGGTGCGGATGGGGTAGGTGCGGCTGTCGTAGTTGGGGAACAGGCTCACGATGTCCAGCACGGTCAGGGTCAGCTCGCGGCGGAACTGGTTGTACCTGATCCAGTCGCGGCTGTCGGGACCCCACACGCGCTCCAGGCCGGTGTTGTACCAGCGCACGGCGTGGTCGGTGTAGTTGCCGATCAGGCGGGTCAGGGTCGTTGCGGCTGTTGATGGTGGCGGCGTCGAAGCCCCAGCGCTGGCCGAACACGCTGACGTCGCGCAGCACGCTCAGGTGCAGGTTGGCGGCCACGTACACGCTCAGCAGGGGCACCTGGTAGTTCTGCACGGCGAACAGGGGATGGCGGTGGTCAGGGCGCTGTTCATGTCGTTGAACTGGATGCGCATCTCCTCGCGCAGGGCGGGGTTGGTGGGGTCGGCCTCCCACTCGCGGAAGCTCTCGGCGTAGATTTGGTACAGGTTGCTCAGGCCCTCCAGGCGGCTGATGGCCTGGTTGCGGGCGAACTCCTCGATGCGCTGGTTGATCAGCTGCTCGATCTGCACCAGGAAGGCGTCCCACTGGCTGGGGCCGAAGATGCCCCAGATGATGTCCACCAGGCCCAGCACGAAGCCGGCGCCGGGCACGAACTCGCTCAGCAGGAACTGGGTCAGGCTCAGGCTGATGTCGATGGGGTGTAGCCGGTCTCGATGCGCTCGCCGCCCAGCACCTCCACCTCGGGTTGCTCAGGCAGTTGTAGGGATGCACTCGTTGATGTTGGGGTTGTTGTCCATTGTTGGATCCTCTAGAGTCGACCTGCAGAAGTAACACCAAACAACAGGGTGAGCATCGACAAAAGAAACAGTACCAAGCAAATAAATAGCGTATGAAGGCAGGGCTAAAAATCCACATATAGCTGCTGCATGTGCCATCATCCAAGTATATCAAGATCAAAATAATTATAAAACATACTTGTTTATTATAATAGATAGGTACTCAAGGTTAGAGCATATGAATAGATGCTGCATATGCCATCATGTATATGCATCAGTAAAACCCACATCAACATGTATACCTATCCTAGATCGATATTTCCATCCATCTTAAACTCGTAACTATGAAGATGTATGACACACATACAGTTCCAAAATTAATAAATACACCAGGTAGTTTGAAACAGTATTCTACTCCGATCTAGAACGAATGAACGACCGCCCAACCACACCACATCATCACAACCAAGCGAACAAAAGCATCTCTGTATATGCATCAGTAAAACCCGCATCAACATGTATACCTATCCTAGATCGATATTTCCATCCATCATCTTCAATTCGTAACTATGAATATGTATGGCACACACATACAGATCCAAAATTAATAAATCCACCAGGTAGTTTGAAACAGAATTCTACTCCGATCTAGAACGACCGCCCAACCAGACCACATCATCACAACCAAGACAAAAAAGCATGAAAAAGATGACCCAAACAAACGTGCACGGCATATATTGAAATAAAGGAAAAGGGCAAACCAAACCCTATGCAACGAAACAAAAAAATCATGAAATCGATCCCGTCTGCGGAACAGCTAAGCCATCCCAGGATTCCCCAAAGAGAAACACTGGCAAGTTAGCAATCAGAACGTGTCTGACGTACAGGTCGCATCCGTGTACGAACGCTAGCAGCACGGATCTAACACAAACACGGATCTAACACAAACATGAACAGAAGTAGAACTACCGGGCCTAACCATGGACCGGAACGCCGATCTAGAGAAGGTAGAGAGGGAGGACGAGCGGCGTACCTTGAAGCGGAGGTGCCGACGGGTGGATTTGGGGAGATCTGGTTGTGTGTGCGCTCGAAACAACACGAGGTTGGGAAAGAGGGTGTGGAGGGGTGTCTATTTATTACGGCGGGCGAGGAAGGGAAAGCGAAGGAGCGGTGGGAAAGGAATCCCCGTAGCTGCCGGTGCCGTGAGGAGGAGGAGGAGGCCGCCTGCCGTGCCGGCTCACGTCTGCCGCTCGCCACGCAATTTCTGGATGCCGACAGCGGAGCAAGTCCAACGGTGGAGCGGAACTCTCGAGAGGGTCCAGAGGCAGCGACAGAGATGCCGTGCCGTCTGCTTCGCTTGGCCCGACGCGACGCTGCTGGTTCGCTGGTTGGTGTCCGTTAGACTCGTCGACGGCGTTTAACAGGCTGGCATTATCTACTCGAAACAAGAAAAATGTTTCCTTAGTTTTTAATTTCTTAAAGGGTATTTGTTTAATTTTTAGTCACTTTATTTTATTCTATTTTATATCTAAATTATTAAATAAAAAACTAAAATAGAGTTTTAGTTTTCTTAATTTAGAGGCTAAAATAGAATAAAATAGATATACTAAAAAATTAGTCTATAAAACCATTAACCCTAAACCCTAAATGGATGTACTAATAAAATGGATGAAGTATTATATAGGTGAAGCTATTTGCAAAAAAAAAGAGAACACATGCACACTAAAAAGATAAAACTGTAGAGTCCTGTTGTCAAAATACTCAATTGTCCTTTAGACCATGTCTAGCGTTCATTTATATGATTCTCAAAACACGATATTATTGTATCATATAGATTATATTATTCGTAGAGTAAAGTTTAAATATATGTATAAAGATAGATAAACTGCACTTCAAACAAGTGTGACAAAAAAATATGTGGTAATTTTTATAACTTAGACATGCAATGCTCATTATCTCTAGAGAGGGCACGACCGGGTCACGCTGCACTGCAGGCATGCAAGCTTGGCACTGGCCGTCGTTTTACAACGTCGTGACTGGGAAAACCTGGCGTTACCCAACTTAATCGCCTTGCAGCACATCCCTTTCGCCAGCTGGCGTAATAGCGAAGAGGCCCGCACCGATCGCCCTTCCCAACAGTTGCGCAGCCTGAATGGCGAATGCTAGAGCAGCTTGAGCTTGGATCAGATTGTCGTTTCGCCTTCAGTTTACTATCGATCTCGTTGTAGCGTGGGCGTGTGGTTGAGGCTCGAGCCGTAGAACTTGTCTTCCGACGCCGACGTCATCGCGATGAGCCTCCATCTTGGTCGCGGCGGTGGGAGTTCTTCATGACTTGTGTGTGACGCAGTCACCACCTGGCGTCGGCGTCGGACCCCGGCTCGATGGCGATCGCGCAGATTGACGAGCCTCGTACCGCGTTGTTCGGCCAGGAAGTGTAGCACATAAGCGTGGAGTGCTTCAGGCGAGATGAAGAAGCGAGCTGCGCGCTCTGTCTTCATCGGCGACCGTCGAAGAACATGGTCGAGTTCGGTTGGATCGGGCCGTTGGGGCCAGTGTCGACCCATTCACCCAAGTCGCGAACACAGGACTTGATGGCCTTAGAAGCGAGCGAGATCGCCGCCCATGATTCCACCGCCCACTGCGATTCTACGGGCCTCGGCGCTAGATGATCTCCCGGAAGGATGACACCTGCGATTCGGATGAGACTCAGTAGTGTGCCCAACCGTGCGTCGGGATCACCGCGTCTGCGGCTTGAATTTGTGGGTAGCGGATCTTGGTCTCGAACGATACCTGTGATGAAGTAGACTTGCCTCGGGCCGGGCAATGCATGCCCCTCTTCGTCTCTCAACCACGATCGCGGCTTAAGCACACGAGTGGTCGGCGGCGTAGATCAATAGGGCTTCTCAGTGAGGGCACCAAGATAGGCGTTCGTGAGGCGCCTTGAGTTCAGAGGCTTCCGGCCTCGAGGTCCAAGTGAAGCACTCGGCCTTCCTTAAGGGCGGTATAGAGCGAGCCTCTTCGCCGAGCGTGTGAAAAAGCGGCTGAGCCGCAAACATCCGTGACTCTCTGCATGCCCTTCAAGTCCTTGATGGGCCCCATGCTTGTGATGGCGCGATCTTCTCGGATTGGCCTCGATGCCCCATCGGAGACAATGAACCCCAAGAGCATGCCTCGAGGAACCCCAAAGCACACTTCTCGGATTAAGCTTCACGTCTTCGCCTTGAGACATCGGAATGTCACTTCAAGGTCGGAAAGGAGGTCGAGGCTTTCCTCGTCTTGACTCGATGTCATCGGCGTAGGCCTCGACCGTCGACCAATGTGTTCGCCGAACACATGGTTCATCACCGGCGGTGCGTGCACGCATTCCTCAAGCCGACGGTATGGTGACATAGCGATACATGCAGAAGGGTGTGATGAAAGAAGTCATGGTGTCGGTTCTTTCATCCCGATTTGAGTGGTACCACAGTAGGCATCGAGGAAAGACAAGGTTCGCACGGTGGAATCCACAATTTGATCGATCGAGGCGAGGGTAGGGAACCCGGACATGCTTTGTTTAGACAGTGTAGTCTACACACATCGCCCCTTTCCCTCCTTTCTTTCTCAAAACGAGGTTGGCAAGCCATTCGGGATGGAATACCTCTTTGATGAACCTGCTGCCATTAGCTTGTGGATCTCACTTTATGGCTCTGCGCTTTTCTTCATCGAATGGCCGCGGAGGCTGCTTCGAGTCGGGCTCAACTCGGATATCGGCGAGTGCTCGGCGACATCCTCGGTATCGGGCATGTCAGGGACTCCGCCAGCGTCGGCGTTCTTGCGGAGAAGTCGGCAACCTTTGCTTCCTATTTGGGATCGAGCTGGGCCGATCGGATCCGCTGGGCGTCGGCTGCTGGGTCGAGGGGCGGACTTAGTCTCGTGGCTCGAGTTGTCGGCGTGGCGCCCACGTCACCTCCTAGAGGCTTTCCGGGTCGGCGATGAGGGCCTTGGATTCGGCGGGCCTCGGCGTACTCCGCCTCCACGTCGCATTCCAAGCAGCGTGTTTGTGCGTGGGGCGCGGTGATGACCCGTTGGGGCGGCATCTTGAGCTTGAGGTAGGTGTAGTTGGGGCGGCCATGAACCGGCGTAGCATGGCCTCCCGATCTTTGCGTGGTAGGTTCCTCGGAACAGCCACACCTCGAGCGTGAGGTCTCCCTTGAAGTTGGAGGGTGTTCGAGCGAGGAAGGTCGAGTTGTCAGGGGTGGGCAGCTTCGGGATGATCGTGTGGAATGGCGCGGCGCTGCGGACGAGGATAGATCAACACGGGGGCAGAGGTCTCGGCATAGATGATGTTGTTTGCGCCTCAGTCCGAGGACCTTGGTGAGCTGGCGTCAGATGGCCAGGGTCGACAGCGGCGGTATTTCCGGGCTCGACGCGTGGTCGGGGTGGTCGCTGGTCGAAGGTGATGGGCTTGTCGGACCGGTCTAGGTAGTGAAAGTAGCCTAGAGGGTGAGAATAGGCTAATCTGAAAATTTTCACAACAAAGAAAGATTATTATACAGTTCGGCTGTGCAAGCAGGTTCATGACTACGTCTAAGTTGAACCACTCGAACCAATCCAGCTGTTTATAAACTTTAGACTAAAATCTACTCGAAAGTATTTACATAAGTTCTTGAGAGAAGTAAGATATAGCTAAGTGAGGATGAAAAGATGAATATGTAAAGGCTATTTTACTTCTAGATAAACTCTGGAGAAATCTTTATGTCAATCTTGCAAGTAAAAATATCTCAAGTTGAAACAAGTAAGAACACAAGACACAAGATTTAATCAGAGGTTCGGCCACACCACAAGGTGTCCTACTCCCGTTGAGGAGCCCACAAAGGGCGGGTCTTTTCAACCTAATCCTCCAAAGCCGAGCCACAAAGGTCAAGGCAATCTCTTCTTATCTTAGCTCAAAAGAGCGGGTGATACCAACTTCTTAGGGTCGTCCACAAATTTGGAGACTCCCAGTAACCTCGAAGATCTTGAAACCTAGGGTTTCAAGAACACCAAGCATACAAGAGGGGTTTGCACAAGCTCAAGTCTTTGAAAAAGAGATGGGAGAGAAAACCAAATCGTGAGCACAAGCACAAACCTCACACCCAGAGCTCCTCCAACAAGGTTGAATCTTGAGGAAGATTTAAGTGTGAGAGAGATGGAGAAGATGAGTGCTTTGTCTCAAGTTAGGTGAGCAATAAATGAGTGAGTGTTGGTTGTCTTGAAGAGAGAGGGAGTCTATTTATAGTCACGGCTCAAAAACTAGCCGTTGACCAAAAACCGTGGCAAGTTGAACAGCCCCTAAGGCGGTTGAACCGCTCCCCAACTGTCGACGGTAATGCGCGGTCGACCGCGGTATGACCACTGCGGCCAGTCAGACGAGCGGATCGGTTGAGCAGCCCCCTAGGGGTTGAGCCGCCCGACCCACCTGGCGACACGCGCCGGTCGGTGGCGAGCTGCCGGATCGGTCGAGTCGAGAGCGCTTGAGCTACCCGAGGACGGTTGGTGGTCTACCGACAAGTTTTGGAGAAACCCTAGCTCGGTGCCACAGGGCAAGTTGTTAAGGTATGGTCGAGAGAAGTCCACGGTGAAGTTGAAGTTCATTTGGAGTTGAAAGTTCAACTCAGTGAAGTCGGCTTGAAAAGCTGTTGCGGTGAAGAAGCTCATCTGGCGAGATTAAGTTGGTGAAGCTCAAAATTGCTTGAAGAAGTTTTGTTTTTTAGAGAACACTCTAGGTTTCTCAACCCAAACCATGGTCGACCAAATAATGTTAAAGAGATTTTTGGTTTTCAAAAATAGCTTTTGAATTAGAGGACTTGAGCTATAGCAAACACTGCACAATGCGAGGAAAAGAACAAGGAAGAATTACATCATGCAACACAAGATTTTACATAAATTTTATCGTCTGTTGCATGAAGTCCTTGGTGCTTCCTTAAGTTCCTGTTTCCTTCTAATCAAACAAGAGAACAAAATTGTTAGTACTCTTATTTGTTTTGTCATTAAATCACCAAAACCCTCACTTGGGGTTGATTGCACTTACAATCTCCTTGGTGATTGATGCCAAAACAATTAAGTCAAAATATATATTTGCAATAGAAAATTTCTTTTGAATGATTGTATGTAGATGGCTCCCTAAATGTGTACGCGATTGTGAATCGCGTCTTGACATAATATGTCATGTGAGCACATTTAGAGATAGTGACAACAACCATACTAAATACAATTATCCGAGGGTAAGAGTGTCATGACGGAGTTGTGATGTACATGACTATCTCTAAAAACCATTATTTTTACTTCATAGCGAGTAGACATTACAAGCGATAGGACAAAGATGATGGCGAAGACCATCATATTACTTTGAATAACAATCCAACGGTTTTATTTCATCAATGGTAAAGGGTACAAGCCAGGTGGCCAAAACAAAAATAATCCAAAGAAAAGCGAAAACCACAAACTAGAAGTTGGATTTTCTCCCCTTTGGCAACAAGTACCAAAGCGGAGAGAGAGACAAGGATACAGAATCAATATCCTCCAGGAGGATGTAACGGGGATAGTGTAGTCGGGGAAAAAAGTCCTCATCGCGTCCTCCTCAAAGTGGAGTCAGAGGTGGCTCATAGTAGAAAACAGATCAAGAATGCGATGATCGGGAGGAGGTGGAGGTGGTGGTGGTAACGAGGCCGGATGGCCTAGGGTCACAGAATGGGTCCGGACCGGGTAAGGAGGCGGAGGTGGGAGGATGATTGACCGGATCCCCATGGTAGGGGCGGGGCAAACTCCTCGATCATCAACATAGACTTCTTCATCTTCATCGTCTCAGACATAAAGGCACCAGTGACCTGGCGGTGCCACTCATTGATCTCCGGGAGAGAGGATGGAGAGGCATCAGTGAGCGAGGGACAAAGGCATGCCCATGGAGGAAGCTTGCGGCGAATGTCGTCGATCTCCGGTGGCGATCGTGCCACCTCATGGACATCAGCCAAATGTTGCGTCACATGGAGAAAATCATGCAAACCCATGGACCAAGCGAGCACATCATCCCACGGCCACGACCTCGACCATGACCACGAGCACGTGGCATGGGAGATCCAGCAGAGAGAGACGCGGCGGCGATGAGAGAGGAGGATGGATGATGGGGAGGCTGGCGGGGTGGCTCAGTGGAGGAGGCAGGGCCTCGAGAATGAGGGCCCGCCGTGCGGCGGCGGGTTGATCCTTGGTAGGAATGCGTGGCGGGAGTGTTTGCGGGTTTTTGAAGTGGGTGCAGGTGACCACTTGATCATCTTCATGATAAAGGGTGCATGAAGACACCCTCGAGAGAGACAGGCCCACGAGCGATCTCTTCGGATCATGTCAAACATTGATGCGGGAGGCCGTTTTGGAGCAAGGAGGAGGAGGAAGACTCGACTCTCCCGAGGGCGTTTATTTGATTTCCGCCCTTGGAGAAGCGGTCATCGGGACAAAGAATTAAGGTACCACTAGTACCTATGCATGTTAGCCGTTGTCGGAACTCAGCTCGAGTAGACATGATGAATGATCTGTCTCTTCGCATGGATAAGAATGTCATGCACGCATGTTCTTTATACCTGATATCTCACATCGAGAAGCCTGAATATGGGCAAACAGCGATAAGCGACTTTGTGCGATTACCACGGATGAAGAAATACATGATGGGTAATCATACTCATCGGCTTCCTCATCTACTTTCTTTATCCGGAGGGTGGCATAGGCGAGCTACCACCTCATCGTTCTAGGTATTCCATGGTCATAATGTTAGTAACCCCTTTCTCTCTAAGTTTAGAGGTGGCGACATGTCGGTTCTCCATATTCTCACAACCCTCCATCAATAAATCTATGTGGACGGTGGGTGTTTCTTGGAAAGAATGACATGTAGAAGTATCGGCTGAAAAGATTCCAGGCGGGTCCTGGACACGGGATGACGAGGAGAGAGAGTAGGGTTGGTGAACCTCAGCGGCGAAGGTGTCTATTCCTTCGAGTGAAGTCGACGGGAGATTCATGCGGGCCGCGGCGGCGTTCGCGGATGCATGCGATCGGTGGCGTAGAGAGCATCGGCGCGGAAGGTCGCGGGTCGTCGAACGCCCATCCTCCTCTGACAGGAGCATACGAAGGAAGGCGTGCTACGGCGAGGATGAGCGCCAACCTTTTGCGTCTCCGCGAATCGGCTGCGAGGGCATGGAAGAGCCTCACCATCCCGACGAGCCACGTCCTCTCTTAGAGAGGAGGCGGCGAGGCGGAGGAGAGAGGATGGGAGATTCCTCTGAATCTCGCCGGAGTATTTGCTAAACTCAATCACAGAGGGGTTGCGATGACGCACCATGATTGAAAAAGGTGGTGGCCGTGAACGGTTGGGTGTGTGAGAGTGGCTCTAAGGTTGTGAAGCGACTAAGGCAAGAGTGAATGAAGTGGGAGAAGAGAAGGGAGAGTCAAGTATATAGCAGTTGGTGGTCCTCAGACCAGTTCAACCTTATGGACGGATTAATTTGGTTCTAACATTGTGGTCGAAGAGCGAAGCAATGGGAGGAAGATGCCAAAAATCAAATTTCTGGCGAGATTACGAAAAAGTATCTGCGGAGATTCTATGAAAAAAAATCAAAACCAAGTGTTTGCGGATGAGACCGGACGGGAAAAGAAGCTGCGCGAAAGTTCTGCGGCGCCTGTATCCTCACCGCTGCTTTGCCGCAGGTGACAAAAATATTTCCTTCTTCTTGCGGGTGACCGGTAGTCACAAGAGCAAAGAGCTGCGCCTGGCGATAGTGTGGGGCTAAAAAAGCGTCTTGCAGTAACTGGAAGCGGCCCAGACGGGAAAGAAAAATTAGGCGCCCACGACACGCATGCATGGTGTGGCGAGAGAGAAGAATCTGCGACATGGTCGCGGTGACGTGGCGTCCAAGAAAAATGCTGCCCAGAGAGAGAAGTAGCCGGACAGAACAAATAAAAAATTGCATCTGTGATGGCGACTCTGGGACACCACAAAAAATGCCAACGCATTGGCGCTAGAACACGGCGGATCTCGCGCCGCACGACATTTGCATAAAGCTAAGACGATGACCCTAAAAAAAGTTACGCGCGACAAGAAAGCGGCCGGACAGAAAGTGTGTGTAGCGGACCTCTTGGCCAGCGACAAGAAAAAAAAAAATAAATCTGCAGCGAGACAGAGGGAAGTAGGACGGGGAAAGAGAGAAAAAAATCTGCACCTTGAGCCACAGAGAAGGAAGCGAGTAAAAATATTATTCTGCGGCCCGAAAAAGGTCGGTATCGACACCTGCAGTAAAAATTTCACGCCCAGTATCGGCCAAAAGTGAAGCGACCGGTCAATAGAAAAAGATCTTTGTACGTTGAGATGTGGCGGTTAAAAAAATCACGCCGCGTCGACATCGCTGCGGTGCGGACTGCGTGAACAGCTTCTTGTGGTCAGGCCAGGCGGCAAGTTCAGGGTCCACCATGTCGGTTCAACGGTTTAACTAGGATGGTCTGGTAAAAATGGTTAGCATTGAGTGAAGTTCGGCTCTAAGTTCATTGGCCTCCTCCGGTGAACTAGAAAGTTCAGTGGTCAAGCAGTTGAGCAAAAAGAATCTTAGCACTAAAATGCATTCAATTTTAACATTTTCACCAAGTTTTTGAAAGATCATCACATAGCTTTAAACTATCAATCAAAAATAGTGAATCAAACAATTTTGTGACCAGTTTTCAAGTTAAGTTCGAATCCAAGATATTTAATTCACTCTTAAGAAAACAAAACCTAGTGCGATCGAGGTTTTGTGAAGATATCGACTGTTGATTTTGATTGCTCACATGAAACAATTCGATATCTCCTTTGGCGCGGTCTCTCAAGAAATGGTGTGCGATGTCAATATGTTTAGTTCTAGAGTGTTGCCTGGGTTGTTTGCAAGTTTTATGGCACTCTCATTGTCACACAAAGTGGAATTTTGTTAAACTCACAACCATCTCTAAGGGTTTACTTCATCCATAACACTGTTACAATACCCCAGGCATATACTGCAACCTGCAGTGGAAAGTGCAACACAATTTTGTTTCTTGGAACTCCATAATACTAAGGATGCCCAGGTGTAAGTCCCGGTAGTGCTTTTTGATCTACTTTGCAAACAATAATCTGTCGAGAATAGCCAAGTAAATCGAAAGGGAGCCTTTGGGATACCATAATCCTAGGTTTTGGGTGTGAACTAAGTATCTTAGAATTCTGCGCTACAAGATGGCAATCTTTAGGATTTGCTTGAAAACACACACATGCAAACACTCAACATAATATCGGTCTAGATGCACATAAGTAAACTAGTGATCCTATCATTGATCTATATAATGTTTGATCTACTGGTTTACCTTCCTCATTTAGTAGAGATGTCCATTTGATGACATTGAGTCTTGGCGTGTTTTGCCTTTTCCATGCCAAATTTCTTGAGCATATCTTGTGTATATTTGGTTTGGCATAGAAAAGTACCTTCCTTAATTGTTTGACTTGAAATCCCGGAAGTATTTAAGCTCGCCCATCATAGACATCTCAAACACATTAGTCATTACTTTGCTAAACTCTTCACAAAATTTTTCATTAGTACTACCAAATATAATGTCATCAACATATACTTGGCACACAAATAATTCATTGTCAACTTTTCTAGTAAATAAGGTAGAGTCGGCTTTTCCTATTGTAAACCCATTCTTAATTAAAAATTCTTTAAAGGCAGTCATACCAAGCTCTAGGGGCTTGTTTAAGCCCGTAGAGTGCCTTGTGAAGTAGATAAACATGATTTGGCTTCTTTGGATCTTCAAAACCGGAGGTTGCTCCACATATATATCTCTCTTGTAGTGGTCCATTTAGAAATGTGCTCTTGACATCCATTTGGTATAGCTTGAAATCATGGTTAGTAGCATATGCAATTAATATTCTAATTGATTCTAACCACTCTAAGCGCATATGTTTATAAAATCAAGTCCTTCCACTTGAGTATAGCCTTGGGCAACCAGAGTGCCTTGTTTCTTGTAACTTCCATGTTCATCTTGTTTGTTCCTAAAGACCCATTTAGTCCCAATCACATTTTGTTTGGTCTTTGGACTAAGGACAGACTTCATTCGGGTGAAGTTGTTTAACTCTCTTGCATGGCAATTATCCAATCCGGATCACCAATGCTTCTTCAACCTTAAGTGGCTCAAGAGAGGAAACAAGCGAGTAAAATTCACAAAAATTAGCTAAAGGATGAGTCGTTACCCCTCTCTGATGCTACCCGGGATGTTGTCACTTAGGATGATCCCTTTGAATTGTATGATGGACTCTTGGATGAGGCATTGATGGTTGTCTTTGTATTTGCTCCTCCTCATCATTCACTTGATCAAGAGGTACTTCACCATCAATGCTTTCGTGTTCATCTTCTACCCTTTGTTGGCATCCTTTGATGATTTTCTTCATGGCTTGGATGACTTGAGGTTGATGGGTTTGCTTGGGTGGAGACTTTGTCCTCACCACCTTTGCACCCACATCAACAACCTCATTGGTCATCGAGAAAGTTCCTTCCTCATCATCCTTTTCTTGAGGTCTCACTTCACCTATTGCAGGATTTCTTTGTGGCTACAAGGTAGTTCTTCATTTCCTACGGTGACATTAGAAACATGCCCTTGACTATTAGACTCATCAAATGTCACGTCTATCGCTATTTCAACAAGACCGTTGGTATTGTTTGAAACCGCGATCCATGCGCATTTGATGCATAACCAAGCAAGAAGCCCTCGTCCACTCTGGGAGCAAACTTTGAGCTCTTGACTTTCTTGTTAAGAATAAAACATTTACAACCAAATACTCTAAAATAATCAACTTTAGGTTTGTTACCCGGTGAGAAGCTCATAGCTGTCTTTTGTAGATCTTATGAAGATAGAGGCGGTTGATTGCATGATAGGCGGTGTTGAGCCTACCTTCAAAGTCGGTGTCTTGTACTCATCCAACATGGTTCTTGCAGCTTCAATTAGAGTTCGGTTCTTTCTTTCCACAACACCATTTTGTTGTGGAGTGTAAGGCAAGAACTCATGCTTGATTCCCTCTTCTCCTAAGAATTCTTCGACACTTGTGTTCTTGAATTCCGTCCCATTATCACTTCTCACTTTCTTGATTTTGAGCTCAAATTCATTTTGAGCTCTCCTCATAATTTCTTCAATATTTCTTGAGTTTCACCTTTATCACTAAAAGAAAACCGAGGTGAATCGAGAAAAATCATCAACAATGACTAAACCATACTTACTACCAATGCTAATGTAGGCCACAGGTCCAAAGAGGTCCATGTGAAGAAGCTCCAATGGCCTCTTTGTTGTGACCACATTCTTTGATTGATGTGGGACTGTGTTGCTTTCTGCGGCATGCGCCACAAACCCTATCTTTCTCAAATACAACATTTGTTAGTCCAATGATATGATTATCCTTTTGAAGTTTGGCCAAATTCCTCATACAGCATGGGCTGGCCGTGATGCCATAGCCACCCTTGTCGGACTTTGCCACTAAACAAAGTCTCGAGCGTCACTTTACTTGTTGTGAAATCAACAAGATAAAGCTTGCCCTTCAAGCCGGTAAAGGCAAGCGAGAGTCCTCCCTTCTAAGGATCTTCATCCACATCCGAAAATAAACAATTATAACCCATTCCACAAAGTTGTGAAGCGGACAACAAATTATAGCTTAAAGAATCTACCAATAAAACATTTGAAAGTGATTGTTGGTCGGAGATAGGAATTTTACCAATACCAATCACCTTACTCTTGCCACTATCTCCAAACACAATTTCTTGTGCTTCTTGAGTTAGTTACAATGTATGAGCTATGTCTTTCTCCCGGTCATGTGATTTGTACATCCACCGTCAAGCACCCAACTTGACCCACGGAGGAGTAGACACGCAAAACAAGTTTAGGCTATGCTTTTAGGTACCCAAATTGAATTGGGTCCTACGGTGTGAATTATAGCCTTGGGTACCCACACACTTCTTTTCATGACTTTTCGTTTAGTGTAGGCACCCATATTTCACAACCAATTTTCCTAAATCCAAGTCAACATATAATGCAAAGATATGAGTGGGAAATGGGAGCATTAGATTTTTGTCCACTTGAGGATCCCTTCCTTCATCTTACTCTTTGTGGAACTCAAGTTAACCTTTACTGAGGTGACTTGTCATTTGAGTGAATCCTCCCCAAGGCATCATATGGGTGGTTCCCTCTATGAACTTGGGGATCTCACCCTTATGCAACGTGCTAGGGTTTTCCTTGGATGAGTTGAACCCAAGCCCCTTCTTCCATTGTTGGGCTTAGGGACTTATACTTGGTTCAACTTTCTTTAACCCATCATTGCTAGAGCATCTTTTCAAAATTTCTTTGACCTTCACATGGGGCTATTCTTCCTTGCATGGTTAGTTAGACAACAAGAAGCATGATATTTGCACAATGTTTGCAAAATTATCATTTAAGCTAGACTTAGATTCAAGCACTAAGATGAGGCATTGATGTTCTTGCAATTTTCAAGTTGCACTTGAAGTTCTTGATTTTGAACATTCAAGCTTAACATGCTTGCTTCAAGTGCATTCTTTTCATTTGCAAGATTAGCTATAGAGGTTTTCATATTAATTACTTCTTTATCTTTATTCTCTATAGTTATCTTGACTAAAGATGCTTCCTTAGTCAAGATATCTAGCATTTCACATCTTTATTGTGAAGCAATTCTTGAAGCTCTAGGTTTCTTTCCTTTCAAGGATAAGCAAGTCTTCTTGAGCATCAAGAGTTGCTTTTCTTTATCCTTCTCCATTAATTTGGTGATAACATTGTATCCATTCAAGCCAAATTCTTTAATCATTTTATTTTTCATGGCAATTTGTTCATCATCATCATCATTTGGAAAATCATTACTAAATAAGGTTACCTTATCTCCTTTTGCCATGAGGTAAGTTGGAGTGTAGGAGTCGTCTTGTAGGTTTGTGACCAGTTGCGAGCTTGATGTAGATTGGATGGCCCACGTTGCCACCACTTCCTCTTCCTCGGAGCTAGAACTCTCTTCATCAGTTCCATTCTTCACCAATATAGGCTTGACCATATTTCTTCTTCTTGAAGTGTCCTTGGTCTTGCCCCCTTTTGAACTTGTCCTTCTTGTATTCCTTCTAGCTTCTTGTTCTATCTTGTTAGGACAATCATTTATGAAATAGCCAGGTTTGGCACATTCATAGCATGCCCTCTTCTTTCCTTTCCTTTGAAACTTGTCATTTTCCTTACAAATTTCTTGAATGTTTTGATGAACATATTTGTGTCTTCATCACTTGAGCTATCTTCATCACTTGAGGTCCCTGACCACTTTCTTTGCTTTGGTCTCTGTTCTTGGGTTGTCTTGTTCATTAGTGATCAAGGCATGAGAGTCCATGACTTGATAGGGCTTCTTGGACTATTGTTGTTGAATCTTTGCAAATAATTGATGAGGCGTCATATCCTCTAGTCATCACGATCCTAATCATCCTTGCAAGACTCTTATCCTTTGCTTTATAAGCTCTCATGAACAATCTTGTGACCTTCACTCCAATCTTCACTCCCAAGAACTCTTATTTTGTTGACCAACACCATCAGCCGATCAAAGAGTGATTGAAGCGACTCACCTTTGTCCAATCATATCTTACAAGCTCACTTTCCAAAGCTTCAACTCTATGTCTTTTAGCTTTGGGATCTCCTTCATGTGACATTTTAAGAATATTCCAAATGTCACGGGCATCTTCTCTTCATTGAACTTTCAGGTATTCTTACGGACAAAGACTTCCTTTAATTATGCTCTGCTTTGAGCATTTGATGAACCTCTTGCATCATTTCGGAGTCATCTCTCTTCCTTGGGCGGACTTATACATACCTACATTAACAATCTCCCAAAGACTAGGATGCACAGGATTAAATCGACTTCATCTTGTCGGCCCTCATCATAGTTCAACTCACTAGAGTTGGTAACTTTCCAAGTGGAGGCGGAAGAGAAGTTGGAATATAATTTCAGAGTAATCAAATTGAACTTTGCTAAATTCATTACCTTTGCTTTTGGTAGATGATCCTTGGTGTTGAGACTTACCTTGCTCAATACCTTAGCACCAAGATCCACTAGATCGTCATTATAATCAAATGTTCCTTACCTTTATCTTCTTGGCCTTTCTTCTTGATTCTTCTTCTTTGGCCTTTTTCTTAGCATCTTCCTCTTTCATTTTGAGAACATTCTCTCCAATGATCTTCATGGAGGTTCGAGGACAACAGTCCACTTCCTCACCGGAAGATGTGATGGGGATTCCTCAGGAGGATCCACATGGTCTATTGAGTGACATGATCGTTTACTCACGTGGTTAAGCGTGCAGTCTGACTTTGATACCAATTGAAAGTAGCCTAGAGGGTGAATAGGCTAATCTGAAAATTTTCACAACAAACAAGATTGTTAGATACATGATTATTAGTGCGGTAGGTTCCATGACTACGTCATAAGTTGAACCACTCTGAACCAATCCAAGCTGTTTATAAACTTTAGACTAAAATCTACTCGAAAAGTATTTCACAAGTTCTTGAGAGAAGTAAGATATAGCTAAGTGAGGATGAAAGATGAATATGTAAAGGCTATTTTACTTCTAGATAAACTCTACGGAGAAATGCTTTATGTCAATCTTGCAGTAAAAATATCTCAAGTTGAAACAAGCAGAACACAAGACACAAGATTTAATCGAGGTTGTCACCCACAAGGTGTCCTACTCCCGTTGAGGAGCCCACAAAGGGCGGTCTTTTCAACCTAATCCTCCAAAAGCGACCACAAGGTCAAGGCAATCTCTTCTTATCTTAGCTCAAGAAGCGGGTGATACCAACTTCTTAGGGTGATCCACAAATTTGGAGCTCCCAAGTAACCTCGAAAGATCTTGAAACCTAGGGTTTCAAGAACACCAGAACTCACAAAGGGTTTGCACAAGCTCAAGTCTTTGAAAAAGATGGGAGAGGAAAACCAAATCGTGAGCACAGGCACAAACCTCACACCCAGGCTCCTCCAACAGGTTGAATCTTGAGAAGATTTAAGTGTGAGAGATGGAGAGATGAGTGCTTTGTCTCAAGTTAGGTGAGCAATAAATGAGGTGCAGTG

>nano7

TAAGTGTGACCAAAATTTGATTTCCTTGACCAAAATCACACTTGGATAACTTGGCATAAGGTGGTGCTCTCATGAGGCGTTGAAGCACTACATGCAAATGCGAAACATGTTCTTCTTCGTTCTTTGAGTACGAGTGTCATCGATGAAAACCACCACAAACTTGTCCAATTCCGGCATGGCGAAATTCATCGGATACATGAAATATCTTTGGTGCATTTGTCGGCCAATGACATCACCAAGAATTCATATAGCCCATATACGGTTGAGAATCAAGTCTTGAATATCACTTGCTCGTATTTTGATCGATGGTAGCGGAGCGAAGGTCTATCTTGGAAAACACCTTGGCCCCGACCAAGCGGTCGAGAACATCAATACGAGGCAAAGGATACTTGTTCTTGATAGTTACGCATTAAGAGGCGTTAATCTATATAGACCTCAAGCTTTCATCCTTCTTCTTCACAAGCAGTCTTTGGACATCCCCAAGGCGAAGTGCTTGGCGAATAAAACCATATCCAGCAACTCTTGCAATTGCCTTCAACTCTGCCAACTCGGTGGGTGGCATTCGGTAGAGCCTCTTGGAAATTGGGTGTTCGGGTTACCTTTCGATGGCGAATTCAATATCGGTCGGTGGCATTCCGGCAATTCATCGGGAAAGACATGCATACTCCTCAGACCGCTGGGATCTTCTTGGGGTAACTCAGTCATGAAGCACATGGTGAGAACCTGACTAAGCGAATCAAAGTGAAATTCCCGAAAGGAGAATCAACTTCAGCGGTCATTGGCTACATCAAGCACAACTTGGTGCAAGGTCATCAATTTGCCCCTAAATAATGTCACATTTTCCAATCCCAACACAAGAGTGGTTTATTAATGTGGCTTCCGATTGAATAGGCACACTTTGGTTTTAATTGATTAGTTGCAATTTTACCCCGTGTGATATCATGAATGAACCTTTTGAGTGAGAGAATGGCGGTTCGTTTTATACTTGAACTTTTAGCTAATGAAACTATGAGATGCACGAGAATCAAGCAATTAAAGCAAGTTGATTATAAAGTGAAAAGATCACAGGTCATGATAGGAGCTCCTTACGCACTTCCTCTAAGCGGTGAGTTGAGCTTCCCTTGCGACTTGCACCTACTGCTTTCTTCCCTTGTTTTGATTTGAGTGGCGGCATCTGCCTCTAGCAGTTCTGGGACAATTCTTGGCATAGTGGCCCACGTTGCCTAGGTGAAACACTTGTTCCCATTGCCACTGGCGGAGGCTACTTCTGGCACTCACAGGCTGATTGTTTCTTAGGCGAAATGGATAAGCGGTTGGAGTGCCCATTTGCTCTTCTTTCTTGCGAGGTAACTGATCACTTATCTGCACACTCTTTGCTGAAAGCCTGCTCTTATTGTGAAAACAATCAAGAACCTCACGAGCACGGCGGATGGTCTTGCCGTGGTGCCTTTCTCTTCTTCTCGCCCGGTGAGCAACAATGCAATCTTCTGGGGAGATGGCCATGTTGACCAACTCGTGAAGCTATCGGCCGGGCGGTGTTGAGTCGTTCGCGACTTGTATTGAGACCCTGCGGGCGATCCTCTTCTTTCATCGAATCGGCGCGATACCTGCATCGGCATAAGTCATTGAAAGCTTGTGCATCTGCGTAAAATGCGGGTTCATGAGCGAGAAAATTCATTCAACTTCCGATCAAGAATGCCGGTGGGATGTGGTGTCCACGAAGGCGATCTTGAATTCCTCCCAAGATACCTCACGATCGCGGTGGAACCTGCAGAAATGGTCCCACCAAGTCCGCGGGCCGCGAAGGCTGTTGTCGTGGCGAGCGACCTTAGTCTCATCGAGCGATCTCTGTGAGGAGAAACTGGACTCGATGGCGCGAGCCACAGCGTCGGCGTCCAAGCGGATCCTACGCCTTGGTGAACAAGAGGCGGTGCGTGCTCGAAACTCTGGTATGTTGCCATAGCGGGAGGTCGAATCTTGGCCTCCACCATGATCTTTGTTGGTGGCTGGCGCTGCAAAGAGCTGTCGCGAGAATCTCGTCTCTTTGGGCCATCGACTCTGCCTTTATGGGGCTGAGGAAATTGGCGAAGGCTTGCTCGTTACGCATGACCAGCTGCTGGTACGCCATCAGGAGAGATTGTTGCCATTGTTATCAACTCACAGTTTGAGCGACAAGATATCATCTCATATGGAAGGAAAAAATGCCATAATCATGTATTAGGTTCGAAATGAAGATAACATGGTGACAAGATCCTGATTTCAAAAGTTTATAGGGTTACATCAATCAAAGTACCTAAGCCTAGTCCAAAATGTGATACTACTAAGCTGCATAGGTTTCACCGCCTAAAAATGTCAAGCTATTGCTTAACCACAGCGGTAGGCGACCGGGATCTGAATTGAAGGAGGCATCGCGAGAGTGAGTCCATTGGCACGAGTAGTCCTGGCTCTCGGGGAGCCTCTTCTCCTAAACTTCCTACTTCATTGGCCTCCATCTCCGGTGGTGCATGTCCAGTGGCCATTGGCTTCCTCGAGTTCCCTACGCACATCGTGAGCTGGTTCTCAAGACATCAATTGTGTTGTCTCGGATCTCCACTTCTTTGCTCCGGGTGGTATCTGTTGGCTCGGCCTTTCCACTGCAGATCCTTCTCCACCAACTCTGTGGATAGGTCGACCACAAAATCTTCCCGGTATCGAGAGTGAGCTTGGCGGCTGGCGGTGTTAGCAAGAAGTGTCATAGCATCGCTCTGAAGGGCTGAGGCGTCTGTTGCACTCATGCTGAGCAGTGACGACCTCCCAACCAAGTCGGGATACATTGCCCACATCCTTCACATGCTCATGCGGTTACACCACATGGGATCATCCTTCTTCTCGTGGGAAGAGTCCCAAGGGTGCATCACCATCTCGGGGATGGTAGCCACGAAAAGTTGTCCGAGTCTTCATGGCGCTGCCTCAACGGTGTCGTCAGTCTGAGTCAATCGTCTGAGTCAAGAGCGTGCTTCATTGAAGGATGAGCCTCAAAGTCGGCGGACAGGCAACAAGGTACCGTGCTCCTCATACAAGCTGCACAGTACAAAGGGGCGTAGGGTAGGCGGAGATTAAGCTTCCACAAGATGGAAGGAAAGCCATCAGTGAGAAAGGAAGTCGAAACTGGCGAGTCTCCTCCCTTTGGCGGGGTGGGTGAATTCATAGGAAGAATCAAAAATAAAGATTATGGTGGAAGGAAAAGAAAAGAGAACCGGATGGTTTTTAAGAAAGGGGTTAGCTCAAATTTAATTCCTCTTTGGGGTTTATAATGCATGCATCTTTGGAAAGCGTTGCCTCTCAAAGAAAGAGATAGGGTGCCTTTTAGGGCATCCTTAAAATATAAATGTGGCCCACGGGGCCTAATTAATCAGCCACTATTTCTCCTCTATACCTAAGGCCTTTCGTCCTAGGTCTAGCGGTCTAGTCTGTCGATCATGTGATTTCTAAGCAAGTTTAGATTTTGAAAATTATATTCATGGTTTTTTATCCTTCGTGGTGGAAATTTGCTCGATGCCAGTGTGGCGAACCACCGGGTATTCGGTTTAAGTGCCTAAGTCACCCGAAGTCGTAACACCGTAAGAATAACCCGTCGATCCCTCATATCTAGTCATAGAGCCATTAACGAGGATCAAATTCCACAATCCCACTCGAAGGTGAGTCACAGGAAGAAATACAAAAAAGAGGAAACCTCAAATTAAATGCTTGAGTTATTACATAAATCAGAGTTTTTGAGTAGCAAATAAAGTTCACAAAATAAAGTCTGTGGATGTCGATGTAAATCGGTAATGAGAAATGGGCAAGGCCTGGCCCACTACTCCTCATGCTCTCTCTGTAGGAGCAACATCCACTCATTGTCCAACCCGATTGGCGGAGGTGAAACCAATCACACCATCAGCAATTCTGTATGGTACACAAAAATTGTGCCACAAGCGAGGTGATATACTAATACTCGGCTAGACTTAGGTGTGAGAGAGTCTACTCCTCTACCTCTGAACTATATAATTTGTTTGGTGAGGGTTTGGTTGCCAAAAGCACTATTGTTTCTAAAATCAATTTTTAGCTTTTCAATTTCTACCATCATTAACTAGCTAGATTTGCTCCTTCTAAGCATACATGGTAACAATCAATTAGTTCAATCAACAAGTTATCTCATGTAGTCCCATTTCACTTCTTACTCGATGCGGTACAAGGAATCAAGCAGTCTCATTAGCTGCACGAAATGAGCGGTCGAATCGAGTTTTAAACGCTAAGGTAAACCTAAACACACGGCATGTCGAGGTACTCGACCCGCGCACATGACAACCGTCCCCATCGATTCCCGTTGCGTCGGGGCCTCATCGCCTTGGCATACAATGCTCCACTAACCCATTGCCGCCAGTGCGGTGGCTTGCCTTGTACCATAGCTAGCATGGGAGACCAGTCTCAGTCGCATGAGGATAAAGTCGCGCCCGGCTTCACTCGAGTACTAGGTTTACGGTTACCATTTTCCGGCATGTGCTTGAATGCGTTCAAAGCTTGACTCGGTATCCACATTAATCCTTAATTCATTTTCCCGTCTCATGGACAGGCATCCTCCTGGATCCAAATCCATAGACCAACATAGATCCGTTATCAAGATGAATACAATCAATTCACGACCCCTGTGGCAGTGCTAGAAAAATCACTCGACTTCTACCGAGATCACGATTATGGCGACTACTCGACCTAGCATACTAGTATTCATCTCAAAGGAATCCTAAGTTCATGCAACTAGAGGTTTCAAGCAACTCCTACATAAGTGCATTGCAATCCTACAAGCATTAAGTGTAGTAAGTAGCATATATAACATGGTTATGCATAAAGCAGGGCTTGCCTTCAATTGTTGAAGTGGGGAGATCCTCAATACTGACCTCAAGGCACTCTGGTCCTCCTCTTGGGCGGAGTCCTTGCTGGGGATGAGCAAGTACTCTCCGTCGACAAGATTACAATCTAATGAATGCAATCAGTAAGATATATGCATGATATGATATGTGCTTTTAGAAATTACAACTTTTAAAGTGTAGGATCTTTTGAGTTTAAAACAGTTAACTTTACTTATGTAAAACCCTTAGTGGTATACTTGGTAAATTGGGTTAGACTTAGCTAAGTAAGTGGTAGGTTTATTTTTTGGGTTCCACAATTCCTTTTAAGTCTTAGGGATAAATGATAAGTTCTCAAATTAGACTTATTGGGATGAGTTTATTCCTTCTTCTCTTTTCTTTATTCTCTTTAATGTTTTTGAGTAGGTTTGAACTACAAGTTGCTTTATAAAATTCTAAAATTCACAAAAATTACAGTGGCTTGTTACTGGTGTATGTTTCCCTGTCTCAAAATTTGGGGTCAGAAAGTGAATAGTTTTCTCTGGACAAAATTATTAAACTGCAGGGTAGAAGGGGTGCTTTGAACTACAACTACTATTTATGATTGGTAATTCTCTAAAACTTATTTTGCTGGCTTTTAGGTGTTATAACATGACTTGATACAAATTTCTAATCATTAATACCTCTTAATAAATTTTCTATGGTTTTCTCAAGTTTCTAGCCAAATGGGTGCTTTCTACTACCACTATATTTGAAAAATATCAAACAATGAGTTCTTATTTTTCATAGTTAGTATTTTGTGCAAAAGATCATGCGAGTTTGGCTTCTTTTTGCAAGGGAAGGGTGGTTTGCATTATTTGAATTAAGTGGTCTTTCTCTTTAATTATTAGCAAAGGTATGGGTTTACTTCTTCTTCATGGGTTTGCATTTTCCTGCAGTAGTTTACCTCCTCATGAGCTTAGCAAATTTTGGTTGCCTATTATCACATTAAAGGGTTGTTCATTGATTTATTTGGAAAATGCCTTATTATCACCTGTATTTATTTTCTTTACTTAAAAAGTTGGGTTGAGGTGCTCTTGTATTTTTGTAGTGAGGCTCTGGTGGTTATAAGTCCACTAGATTTTTATTAATCATTTTGGTTATAGTTTGTAACTCTAATAATTGATTTTGGTCTATATGAGGCTAAACAAATCATCTTAATTTAAAGCGGTCCAAATTAATGGTCTCATTTTTCTAGGTTCTCTGTTGCATAAGTAAACTAGGAAAAATATTATTAATCCTTGTTCATTAATTTCTAAGGCCTTTACGTTTTCTCTAAGTTTTGGACAAATTAGCTTTAAATGAAAAACTACATCATATTTTTTCAATCTTTGAGGTTCCTACTATTTTAAATAGTGTCTAAATAAGGTTTAAACATCTACAATTTTTCTTAAGTTCAGCCTGGAAGAAAACTAATTTTCCTTAATTAAACAAGGTTTAGTGGGTTTGTCTTTAATTTTAAACTTTAAAATTTTGAGCAGAAAGCATGGAGTTCTATTTTTAAATGATAGATCATAATATTAGAGCTAGCAAAATTGGTTTGACAACTTTTTTAAGATTTCATCATGTTATGGGTTTCTAAGTTCTTTGGTCATTTAAAAGAATAAAGAATTGATTAAATGGAAATCCACTTTGCGAGAAGTCACTGGCGATTTTCTAAGTTTCCCTCGCGAATCGGTCCTTAGGTTACTATTCATGAGTCGCGACATTCTGAGAAACCCTCGGGTTCTACCAATCCTAACGAGGTCCTTCTTCTACCTTAAGCGATGGCGCGGCGGAGAAAAGGCGAAAGGCTTACTGCGCGAGGTTGCTCGGTGGTGAGTCGAGGACGAAGGAGGTCGAGGATCAGCGGTGTGCAGTTTGCAAGTCGGAGATGGCCGGTCGGTCGATCCATGGCGGCGAGGATGCTCGTCGCGGCGAGAGGAACGGCCTTGTCCGCAGCGAGATAGTTCAATTAGATGGGTGAGGAGGTTCACGGGATGCGAGAGAAGATATGGCGAAAGGAATTGGCGGAGACTCAGCTGGATAGCTCGGTCCACAGCTTGGAAGACCAAGTCCGGTGGTCGATCTCGAGCCTCCGGTGAAATCTGCGGGTCGAGGCAGCAGCTTCACGAGCTGGCGGGCTAAGCCAGAAACAATGGGTGGGCTTGAGGGTGGTTTGGAGTGGGTGGCCATGGTGGCAGCTCTGTGGCAATGGCAGCGAGGTCCTCGCGGAGCTAGGAAAGGTGGTGGCGGAGAGGGTGAGTCTTGAGCGAAGAGATGCGCCGAGGGAGGCTATCGCGCGGCGGAACACAGTAGGCGTGGTGTATGCGGACTTAATGGACGCCGGTGTGGCGAGGTTAGGCGAGCTCAGGCGTCTGGAGCGATGAGGCACGATGTGCCCGTCGTTACTGCCCAAGTTACGCGTGTGGTCGCTCATCAGAAGCTGCTCTCGCCTTAGTCAGTGCACAAAACCTCTTCTCCTCCCTACAAGCTACCATTCTTGTGTAGGGTCTAGGTTTTGCCTCGGTTGCGAGAGATATGGGCGAAATCTGTCTGTCCTGTGCCCAAAGAGGCAAATCAAGGTTTTGTCGTGTCTAGGGCTCAGCGTCCCAATGCCATCTTACGGCACCTGACGAGAGGGTTAGTTAGACACAATTTTGTCAATGGGGCCATTAGGATTCAAGTTAGGGATCAAGGTGAACATCTGATCTTTGGCTCCAGGTCAATTTGAGAATCTGAAATTCAGAGAAATCCCAATAGTCCCACATGAGGAGCTGATTTGGGGTTTATTTGAGTTATTTTGGCTAAGCTTTCTCAACATTTCTTGTTGCCCATTAAATATTCTTTAATTTTCATAATTGGCTCAAGTCATAATTTTAAACTTTTCATTCCCTTTTCTTATTTTCTTGAATTTTGCTCATGGGGCTCACTTAGGGTTCTTAATTAGGGTTGCACATTTTTATCTTTTCAAAGACTCAATTGTTTTGATCATGTTACTTTTAAGTATTTACTTGGTGAATTCTTTATTACTTAAGTTGTTTGATGCTCATGCTTACTTTGGTTCACATAAAATGATGGTTCTTGGTTTGGCATTTGAAAAACCGGTGACCTTTAGGGTGTCAATCTGAGCACAGGGTCAGGCGACGAGTTCGTCGTCTTAAAGTGGCCGTCAGAACCACCAGGTCGAGTGAATGGGTTCGCCATCTTCAAATTTGAGCAGTCAGAGAATACAGGTTGGCGGGCCGAGTTAGCATCTTCTGAGGCCGAGCAAGTAAAACCTAGGTCAGGGGCCGGAGTTCGCAGTCTTGGGAGTTGAAGCGTCAGGACCCTAGGTCGAGGCGACGGGTCGCCATCTTAGAGTGAGCGAGTCCGAACCCTAGGTCAGGCGGCGGAGTTTCCTATGGTGCTGAGGCGAACTGGCGCCTGTCAGCCTCACTCATCGAGTAACACTGGCCCGATCGGGCCAGCGCGGCGACATCCTCTGTCGGCGGACGGTGGGCGGCGAAATGGCTAGGTCGCCGGCTCTGTCGGTGGAGCGTCGTGTCGGAGATAAAGGTGTCGAGCCACCTGCATTAAATGCCCTGCGATTTGGTCGGTTAGCGCGGCGATTTGGCGGGTTGCTTCTTAGCGGCTGGGCCTCGAGCGGCCGGAGTATATTCGTCGTTGGAGGGCCTCCGGGCGAGCGGAGATCCTCGGTGTTTGCCGCTTGCGAGCTCAAACTGAGCGGGCGTGTCGAGTCCCTCGAATGGACCCGATCCACGACTTAGTCGCACCCATCGACCTTTGCGGCTTTGTCGCGATGGGTTACAGTGAGTGGAGTCTTGAGTACCCTAATTATGGTACCGACGGTAGCCCAGCCTCGAAGAGTGTTAACTCTGCCTCGCTTTAACTGTCACCACAGGGACCAGCCTTTCTCGGTTGCGTTTTGTTCCAGTGGAGTGCGCGGCGGCGCCCGCATGCGAGTCAAGGCCGGGCTCGGAAGTGGTTTGACTCCTCAGGTCTCTACCTCATAATGCTTGGCCGATACAGTCGTTCCCTCATCTGTGCCCATGCCCGCGATGCGCAGTCGGGTCCCAAGTTCTTGGCTGTATGTTGGCTTGTTATCGCAGTTTGGCCGGGCCGGGTTTCAAGCGACGAAACCTCATACAGGCGAAGAGCAGTCGAACGGACTGACTTTTTAACATCGCCCCTAGCGTCGCCTTTCGCAGGAGAAGAGCGCCATGTTGCCCTCGGAGGCGCCAAACATGTGTCTCAGTGTTTCTTTGGCGGGTAATCAGTGTGGGCGCTGGTGCCCCATTTGTTAGGGGTCGGCTAGAGCCGGGGCGCGCTCAAAGTACCTCTGGTGATCTGGGACCGGTCCTGTCGTGAGGTCAGGCTCGATCCTCCTCTGATGGGATTAGTTACAAGATCATTCTCTTTGGTCTCGGAAATGTCTTAGGGTACCTCGGGAGCGTGGCGAGGCCTTGGTTATGTATCGAGCGTACCATGGTCATCCCTCGCCACACCCATATGCAGCGGTGTGAACCCTTCGAGGGCCCGACCGAACCCACGATCGATGAGGTGGCGCGGGCAGGTGGCTGGCCCGTTGAACCCTTAGGGGCCGACCTTGAACCTCTAACTGATGGTGGGTGTGGAGCCCACGCGCTCTGTGGCTGTTGAACCCCTCAGGGGCGACCTTGAACCTCTTATCGGTGGAGGCTCGGAGCTGTTTCCTTCCGCGGAGAAGGATCCTTTTGGGGTATCCCCCTCGGTCCACTGTTATAAGAGAGAAGGAAAGGAAAAAGGATGCAAAATCGAATGGCGTGGCGTACCTCTTTGTGCGGTCATTGTAGAGCGCGGCGTCGCTTCGCTTCCCTGCCGGGCTGCCGCCGCGCGCGCGAGGTGTGCGGCCAGGCGGCGGTTGGCGGCCAGCATTGCGGCTGTGGCCGTTCGAGCGGAACGCAGGCGCGCCGTCTTCACGCCGTGAGAGGTTCTCTCACGCCGGAGATGGGGCGTAAGCAGGTGGCAGGCGGCCATTCTCCTACCTTTGCCACCGCCATTCACTGCGGCCCATTTTGGCCGTATTGACCATCGCGCTGGGCGCCTTCTGGGCGTCTCGCGAGGTCGCCTCAGTCGCGATGCCGGTTCCGCGATCGAGGCGTGGCGGTGGTGCGGTGCGATCGATTGCCTGCGTAGCGGCCGTATGCGTTCTGTCGTGGTCGCGTGACATCGCCGGAGTCGTTGCAAGTCGGACGCGCCCGCCAGCGCGATTGCATCACGCTGCATAGCTGCCACCGCCCATTGATGCAGTGGTGGAAGAATCTCGTAACATTTAGCGGTTGCATGCTGTAGTGACGGTTGGCTAATACGCTGCAGGCGGCTTATGCGGCCAGCGTGGGTCCCGCCCGCGTCGTGGGGAGGACCTTGGGCGTGTTGGAAGAGGCTTCGGCGGGCTGTGGGGCATAAGTAGGAAATCGCCTAAAAGGTGGGTGACCCTTGAAAGGCGACCATGTCTTGCAGCTCCCTTATGTATCGTGTCTTTCCACCTTAGAGCCCGGATGGGAACACCCGCCATCCCTCGCCTTGTCGTTGGAGGAGCGCAACTCAGTGGGAGTTGGTACCTTTAACCATCGTTGGCTTCAAGAGTTTTTCATCACGGCCGGCTGCACCCCTCTGGCGGTCACCCAAGATGGTGACCTCGGTTGATGGCGAGGGAAAGCAAGCCGAGGCTGCGGCCTCTGCCCTCCCTCGGCCTCAGGATTTTCATCGCCGGTCTTGGGGAGGAGTGTCTCAGTTAGAGTCGGCCCATGCGGCGGCCCCGCTCCTTCCTCGGTGATCGAGGGAAGGGCGTTCGCCGTTCGCGGCGGCCCGCGGTACGCGTGCAACTCGGCCGAGTGGCCGGGCGCTCGGCGCCCGCCTTCGCCATGGCGGTGGAAGGTTCTTCGCCCACAAGATAGCGGGGCGCCGCAGGTGAACCTCCAACTCTGACGCCTACGCGATCCTTGCCTCACGAGTTTGGGCATGGGCGAGGAGCCTCATGCGGCACATCCACCGAGTCATCTTCTTTGCTGTTAAACCGGAGCGAATCGTCATCATCGCCCGTATTGGAAGGCGGCGGCAGCGGATCGTTAGTCTTTGCTATCGGTTTGTCGTGTGGGGTTGTTCGTACTGCGGAGGTGGGCGGAGTTAGTTGTAATGGCACCTTGAATGTCGGTCTTTGTTCATTGTGGTTTGTGGGGCACGAGCGTGTATGTATTTTGGCGAGCCGTGTTTTCCTTATTTTCGAGCACTAAGACTACTTTGTTGGTTATGCGAACCGCTTCACCAAGAGTGAGTCGCCCGTACAAGGTGGCGAGTGATGTCCGTATCGGAGGCGTAAGTCCTCGGCTCGATCGGCCTTCTTTGTCGAAACTCTCTCTTTAGTTAAAGAACCCTGTGGCTCTTGATGGCCGCCGGGGTAGCGGTGTGGCTGCAGACGGGCGAGAGTTGGCTCGAAAAGACTTGGTCGGCGGGCTGGCGGGTGCGTCCGATGGCGGGGCCAAGCGCGGAGTTGATCTAGGGCCTCGGCGGTGATGTCTTCGAGACGGTGGCAGGCCGCGGGTGACGGTAGCCGCCTACTGGCCGAGATTCTTGGAAGACCTGGCGGCGATGGCGAGCGTGATGATGATGTCGTCGCCAGTGGAATCCTCGGACCGCGTCATGCGTCAGAGCTGGTCGGACCTCGGCGAAGGTGTAGTCGGCGCGAGGTCTTCTTTGCTCCCTTCAGCTGTCAAGATCCAAGCTGCGAGATCGGATTATCTTGTAGTGTGCGTTTTACTGCGGCATGAAAGCTAAACACACCATCGTGTTGTAAAAAAGCGCTTCTTTTCCTCTTGTTTTGAGTATCTGGACTTTTTCGTCAGTAGCTGGTATGCCATGCGGCGGGAGTTGCTTTTCACGGAAGGTGGCGAGTGAGATATCAGTATCAGGCGTAGAATCCCTCAGTCGGCCCCTTGCCGCTCGTGCCTCTTGCCGATCCATGGGTTACGCTGCCGTAGCTTGATCGAGAAGGGCGAAGAATCGCAGGAGAAGGCTTCAGTGTGAAGACTTGTTCGTGCAGATAAAACATCCGAGCGAGTTACTTATCACAGTAGGTGGGAGGTATCCGTATCCGGGCCGTAGGAGTCCTACGGTCGACCTTGGTACGCTACGTGTACTCCGTCGTTTTCGGGATCCACTTTGATAGTCGAAAAGCACAGACATTACGGCGAAAGGATCTTTTTTGAGAAAATTTCGGCCAGGAGGGTTCCCCCTTTGCCCAGGAGAGGTCGGGCTTTGCAGAGCAAGTGACCCTTCATGATGACTAAACTGCGTGGGATGAGGTATATGAACAACGAAAGCATCTTAAGGGTAGAGCGGCGTGGTGTTGGATGTTCCAAGCGTTCTTTGTGGACCTCGCCTTATTGTTGGCGGCTTATGCGTTCAGACGAGAACTTTGGCGATAATGACGGCCCTTCGAGGTAGCGTGAGCTTGTCGCCCTGGGCGTCTGTCGCGGCCAAGCACGAGTCGCCACGTGGAAGTCTGGGACCAGACCCTCGGGTGTGGTAGCGTCGCGGGCTAACTGTGTCCGTCTTGAATGTAGTAAGGCCATGTCGAGCCTCTTCGGTGATCAGCGAGTCTTCTCGACTAACTTGGTTGCTTTGGTGGCGTAGGCCCTCGTCCTTGGGGGCGTATTCTACAGTGCAATGGGCAAGATGGCCTCGGCCCCATAGACTAGAAGAGCGGCGTGAAGCAGTGGCTCGGCTCGGCGTTGTCCTCGGACTCTGACCCGCGGGGAGTTCCTTCATCCATCGCACGCATGGAACTGTTGAGGTCGTTGTAGATCCGAGCTTGAGTCCTTGTAGAATCATGCAGTTGTACGCTCTACTGCCCATTCGTCATGGGTGAGCCACGGCGGCAGTCCACCGGATGTGGTGATCCTCATGAAGTCGGGAACTTTGCGGTGAGCGGGTGCGTTGTTGCGGTGATGATGGGTTCGGGACTGAGAAATGTGCTTTGGGCCATTTCTAAGTATTTTAGTGATTAGTGCAAACACAAGTGCTTAAATGTGAAAATATGCCCAGGATGACAAAGTGCAAATCACAAGTTAAGGTATGTTTCTAAGCCTTAGTACATTGGTTTTGTGTACTAATATATTTGTCTAAGTGTTAAAGCAGATAGAAGAAGAGAAGAAGACTTGGTGTATGCAACCGAAAAATTCTTCGATGCGAACGCCGGTGTCCGGTGGTGGTGCCGAGGCAGTGTCGATGGTGCCGGGCAGTGTCGATGCGCTTGGTACCTCGGCCGAAAAGCGCGCTCTCGGGATTTTCTCGGCGACCGGCTAAAATTCACCGGGTGTCGGTGTGCCGGCTATCCGTGAGCCGCGATCGGCCGGGCCAGCGGTCGGCCAGCGATCGGCGCGCGCGACGTGGCGGACCATCGGAAAAGAAGCCGGTGCTCCGGTGTGCCGGGGCGTCAAGTGCGCGCGATACAACCGATCGGCAGCGGTCGGCCGCCATTTAAGAAACAATCGGGCGCGGGCGGTGCCGGTGTGCGACCGGGCTTGTCCGGTGCGCCACGAGAGAAAGTAAAGATGGCCTTCAGTTGTTCCCAATGGCTCCTAGCGCCTTGAGGCTATAAAAGGACCTAGCGCATGGAGAGTACACAAGCATTCCTACAACTCTTCTAAGCACCAAGACATCAATCTCACATTCGTTTCATTGTGATAGCATATAGAGCTCTTGTGGGTTGTGAACTCTTTGTGTTGCGTTGCGAGCTCTTGTTGCGACTTGTGTGTGCGTGTTGTTGCTCTGATTTTCGAGTCTTGTGTCGTTGCTCATTCCACCTTACTCAGTATTTCTTTGTGAACTCAATTGTAAGGGCGAGAGACTCCAAGTTGTGGAGATTCCGCAAAGCGGGAAAAGATCAAAGGAAAGAAAAACACGTGGTATTCAAGTTGATCATTGGATCACTTGAGAGGAGTTGAGTGCAACTCTCGTCAGTTGGGACGCCTGCGTGGAGTAGGCAAGTTTTGTGCTTAGTAGAACCGGGATAACCAGTGTCAACTCTGGTGATTGCTTTCTTGTGGTTATTGTGTTTTGAGTTCTCTCTAGCCACTTGGCCATACTTGTACTAACCCTTAACAAGTTTTTGTGGCTTAAGTTTAAGTTTACTGGGATCACCTATTCACCCCCTCTAGGTGCTCTCAATTGGTATCGGAGCCGTTCTCTTCAAGAAAGGACTAACGCTTGAAGAGATGGATCTGAGGGAAGGGTTGTGATCAGCGACAAGGAGAAGAGTCGCCGTCAGGCCAAGGGATGACAAGTCCAATGACTCGGGCTCCGGGCCTGGCGAAGAGATGGGAAGAAGAAGACAAGGCGCATCAAGGAGATCGTCTACTCTGACAGTGATAGTCCTCTTCTTCCCAAGGGCGGCGACCACGACAAACAAAGGAAGCAGGTTAATTCTAACTTTTCTTTTGACTACTCTCGTATTCATAAAGTTCAAATTCACATTGCTTTCTATTCCACTCGCAAGAGCCCCACACTTTGATGGGAGGACTACGGATTTTGGAGCCACAAAATGCGTAGTCACCTATTCTCTCTCCATCTAGCATATGGAGATTGTAGATAGTGGAATGCACTTTAATAGTTGGATAGTCCTATATTCATTAATGAGCAAATCCATAGAATGCACTACTACATTCTTAGCCTCATTGTGCGAGGATGAATATAATAAAGTGAGTGGCTTGGATAGCGCCAAGCAAATGCAGGATACCTCAAGATCTCTCATGAGAAATGATGCTACCTTACTCACCAAAATGGAGTTGGTAGAAGCGAGCTTGGGCGGTTCGCGATGATAAGGGCGAGGAGCCAACTCAAACATACTGCCGGCTCAAGACCCTTGTCAACAAGATAAGGAGCTGGAAGCGCGCGATGGGCCGGACCACGGCGTCGTCGCCTCATGCTAAGTCGCTCATTCTTCTTGATCCTCATTTGGTGAATAATATTAGCGTGAAATCCCGGTACACAAAATGTGCTTGAAGAAATTCTTGGAAAATTTGTAGCGGCGGATGATGATCAAGGAGGCAAAATGCGTCGATGGCCAGCGTTGAGCAGTCGTCCACGAGCCTCAACCCATTGCTCTCAAGGCATCGAGGAAGGCACTACCAAAATTGGTGCAAATTGGTAGCCGAGCTTAATGATGAAAAATGGCTCTCATCATTAAAGCGCTTCAAAACAGTGCTAAAGGTGCAATGGACGGTAGAGCAAGACTAAGACCAAGGGGCGATCATGCTTCAAATGCGGTAAGCTTGGTCATTTATTGCTAAGCTGTCGATAATGAAAGTGACAGGGAAGGAAACAAGAGGAAAGAAGAAGCATTATAAGAAGGCAAAGGGTGAGGCGCATCTAGGCAAGGAGTGGGACTCGGATTGCTCTCGTCCCAGCTCAGACAATGAAGGACTACGCAGCCACACGCCTTCAACAAATCAACCCTCTTCCCAAGCAGCGTCACACATGCCTTATGGCAAGGGAGAAGAAGGTATGTACTGCAACTCTACCTATGCTTCTTCAAGTGAGGACGAATCTAGTGATGAGGATGAAGTAGATTATTCATGTTTGTTCAAGGGCTTAGATAGATCTAAGATAGACAAAATTAATGAATTAATTGATGCCTTGAATGAAAGAATATACTTTTAAACAAGAGGATTTGTTGTATGAAGAGCATGATAAATTTGTTGAGGCACAAAATCCTATGCTTTAGAAGTTAAAAGAAATGAAATGCTTTCTTTTGAACTATCTACTTGTCATGAAACCATTTCTACTTTGAAAGGTGTCAACAATGATTTAAATGCTAAATTAGAAGTAGCAAATAAATCCAATTCTTGTGTAGAACATGTTGAAATTTGTACTAGGTGTAAAGATTTTGGCGTTGATGCTTGTAGTGAACACCTAGTTTCAATTTCCAAGCTTAATGATGGTAGCTAGTCTTAATGCTCAACTTAAGACTAGCAGAATATTCGATAAGCTAAAATTTGCAGGGATGCCTACACAATTGGTAGACACCCACCTCAATTGGGATGGACTTGGCTTCAAGAGAAGCTAAGAACTTAACAAGCCATAAGGCTCCATTCATAAGGAGAAAGGAAGGTCCCTATGGCTAGTAATGTGCAAAAGAACCATGCCTTTTGTATCATGATAGAAGACAAACTAGAAATGTAAGTCATGATGCTTTTGATTCATATGTTTATGATTCTCATGCCATGTTTGCTCCTAGTTCCTCTTATGTGTATGATAGAAATGTTACTAGGAGAAATGTTGTTCCTAAAAGAACTATTCATCATGTGCCAGAAAGAATGTTATTCATGCTCCTAGGAAAGTAGTGAATGAACCTTCCACAATTTATTATGCTTTAAATGCTTCCTTTGCTATTTGTAGAAAGGATAAGAAAAATTGTTGCTAGGAAGTTAGGGCAAAATGCAAGGGAGACAAAACTTGCATTTGGGTCCCTAAGGATATTGTGCTAACCTTGTAGGACCCAACATGAGTTGGGTACCTAAAGCCAAGCCTAAATTTGCCTTGCGGTTTATGCATCCAGAGTTCATTTGGATTATCGGCAGCGGATGCACAAACCATATGTGGGGAGAAGAAGATGTTCTCCTACGTCAAAAATAAGGATTCAAGATTCAATTATATTGGTGATGGGGATCAAGGCAAGTAAAGGGTTAGGTAAAATTGCGATTTCTAATGAGCATTCTATCTCAATGTATTTTTAGTAGAGAGTCTTGGATATAATTTGCTATCATTAGTCAATTATGTCATATAGAGTATCTTGTCTATTTACAAATGTAGATGTTGCATCTTTAGAAGAAGTGATGGTTCACTAGCTTTAAAGGGTGTATTAGACGGCAAACTTTATTTAGTTGATTTTGCAAAAGAAGAGGTAGGTCTAGATGCATGTAATGGCTAAGACTTACGTAATTTGTTGTGGCATGCTAGCTTAGCACATGTGGGGATGAAGAACCTTCACAAGCTTCTAAAGGAAACACGTGATAGGATGACTAACGTGCATTTCGAAAAAGATAGACCTTGTGCGGCTTGTCAGCGGTAAACAAGTGGGAGGAGCACATCACGGCAAACGTGACCACTTCAAGACCTGAGGCTGGCGCATATGGATCTCTTGGACACATGCGCCTATCCGAGCATAGGAGGAATAAGTATGGTTTAGTTATTGTTGATGACTTTTCCCTTCACTTGGGTGTTCTTTTGCGAGATAAGTTGCAGGCAAGGGACCCTCAAAGCGCTTTCTCGGAGCTCAAAATGAGTTTGAGCTCAGGTGAAGATAGGCGACAATGGGTCAGAGTTCAAACCTTCAAGTGGAGGAGTTCCTTGAAGATGAAGGATCAAGCACGAGTTCTCCGCTCCTACACCTGGCAAAATGGTGTGGTAGAAAGGAAAAAACAGGGCGGCTCATCGACATGGCGAGGCGATGCTAGGGAGTTCAAGACCCAGAGTGCTTTTGGATGAGCGGGTGCAACAACTTGCCACGCCATCAACGGTCTACCTTCATCGCCTCCTCAAGAAGAGCGTCATGATGCTACTACTAGGTAACAAACCCAATGTATCGTGCTTTACGTGTATTTGGGAGTAAATGCTACATTCTAGTGAAGAAGGGTAGAAATTCTAAGTTTGCTCCCAAAATTTGTAGAAGGGTTTTATTAGGTTATGACTCAAATACAAAAGCGTATAGAGTCTTCAACAAATCATCGGGTTTGGTTGAAGTCTCTAGCGACGTTGTATTTGATGAGACTATGGCTCTCAAGAGAGCAAGTTGTTGATTGTGATGATGTAGATGAAGAAGATGTTAGGCGGTTATATGTCGAACCATGGCGATTGGAGAAGTGCGGCCAGGAACAAGATGAGCGAATCAATCATCTTCCTCAACAATGGTGCGACCCCAACTCAAGTGGAGCGCTTGATCAAGAAGAGGGCGCAAGATGATCAGTGATGGAGAAGCGCAGCCCGGCACCTCCAACAGGATTCGGCGATGATTCAAAGGGATCATCCCGTCGACCAAATTACAGGTGACATTAGCAAGGGAGTAACTACTCGATCTCGATTAGTTAATTTTGTGAGCATTACTCTTTTGTCTCTTCTATTGAGCCTTTCGGGTGAAGAGGCCTTGCTAGATCCAGGACTAGGTGTTGGCCATGCAAGAGGTTAAAACAACTTCAAGCGCAATGAAGTTTGGACACAGGTGCCTCGTCAGAAGCAAAATGTTGTAGGAACCAAGTGGGTGTTCCATAACAAGCAGAACAAACACGGGGTGGTGACGAGGAACAAGGCTCGACTTGTGGCAAAGTTATGCCCAAGTCGCGAGTTTGGACTTTGAGAGACTTTTGCTCTGTGGCTTAGGCTAGAAGTCCATTAAATATTTTGCTAGCATATGTCGCTCACCATTCTTTGAGTTGTTCCAAATGGATGTGAGAAGCGCTTTCCTCAGCAGGCCAATCAAGGAGGAGGTGTCGTGAGCAACCCTGGCCGAGGATGAGCGGTACCAAACCATGTGTGTAGCTCTCTAAGCGCTCTGGACTTAAGCAAGCCCAAGAGCATGGTATGAATGCCTTAGAGACTTTTTGCTAATGCTTTCAAGGTTGGGAAGGCGATCCAACTCTTTTACAAAGACATGTGATGGTGATTTGTTTGTGTGCCAAATTTATGTCGATGACATAATATTTGGTTCTACTAACAAAAGTCTTGTGAAGAGTTTAGCGAGGTGATGTGTGAGAAATCGAGATGTCAATGATGGCGAGAGTTGAACTACTTCCTTGGGTTCCAAGTGAAGCAACTCAGGGCGGCACCTTCATCTCCCAAGCGAAGTACACGCAAGATCTGCTAAGCGGTTTGGGATGAAGGGCGCCAACTTAAAGACTCGATGGGGGCCAGACGGACACATGAGCCTCAACAAAGGAGGTAAGTCAGTTGATCAAAAAAGCATCTTAGGTCAATGATAGGTTCTTTGCTTTACTTATGTGCTAGTAGGCAGGATATTATGCCGCGTATGCATGTGTGCTAGATTTCAATCCGATCCTAAGGAATGTCACTTGAGTAGCGGTGAAGCGAATTCTTAGATATTTAGTTGCTGCGCTTGGAGACTCTGGTATCCAAAGGGGTCTACCTTTGACTTAGTTGGATACTCGGACTCGACTATCTTTGGATGTAGGTCGATAGGAAGAGCACATCGGGACGTGCAATTCTTAGGAAGGTCCTGGTGTCATGGAACTCTAGAAACAAACTTCCGTTGCCCTATCCACCCGTGGGTAGAGTATGTTCCGCGGGACGGTGTTGCAGCGCAACTACTGGATGAGCAAACCCTCGAGGACTTTGGCTGTCGGCAAAGTCCTCACATGTTGATGAGAGTGCTATCGCATGGCGGAAGAATCACAGTGAACACGTGCCAAATACATGACATCCGGCATCACTTTTGAGACCACGAAGCAAAGGAGATATCGAAGTGTTTCATGTTGCAGAGAAGCCAAGCTAGCCGATATCTTCACTAAGCCTCTAGATGAGACCTTTGGAGTTCGTAGTGAGCTAAATGTCTTAGATTCGCGGAACTGGATTGAATTGTAGCATACATGTAGTTATGCTTTTGATCATGTTCCTTTTGCATTATGTTGCTTATTATGGTGCTCAAGTTGTACAAACACTCCTGGACCTCACAAGTCCGTTGCAAAGTGATGCACACGTTTAGGGAGATGTGTTACAACTTGACCCTTTGAGACTAACCATTTGCTTGAGTTTGCTTGATTTAGTCTCAGAAGGAAGAAAGGGAAAAGGTGGACTTGGACCATGAAAGACTTCATTTTTTCGATGAGAGGAATGCTAATTCAAAGTTCATCTCATGAAATCTTATTGCCATTTGCTCTTAATTGAAGACTTTGGTGAGGCAATGGGGTTAAAAGGCCAAATTAATCCGTTTTGGTGCTTGATGCCAAGGGGAGAAAATAAAGACAAAGTGATAAATGGATCAGACTACCACTTGAGAGATTTTGAAAATAGTAGAATAGAGTTTTGTTTTGTCAAAAGCTTTTATGTCTCTTATTGTCTCTATTGTCAAAAGTTGGCTTCTTGTGGGGAGAAGTGTTGATTATGGGAAATAGGGGAGTTTTGAAATCTTTGATCAATCTCTTTTGGAATGACTCTCTTTATACTTCATCATGTGTGTTTGACTTAGAGATAGAATTTGAGTTTGATTTGCAAAAAACAAACCAAGTGTTGAGGATGATCCATATATAAAATTGAATCAAAAATTTGAGTTTTTATTTGAAGTGATTTTGCACTTGTGCTATCTACTTTATGTTGTGTTGGCATAAATCACCAAAAAGGGGAGATTGAAAGAAATGTGCCTTTGGGCCATTTCTAAGTATTTTAGTGATTGAGTGCAAACACAAGTGCTTAAATGTGAAAATATGCCCAAGGATGAACAAAGTGCAAATCACAAGTTAAGGTATGTTTCTAAGCCTTAGTACATTGGTTTTGTGTACTAATATATTTGTCTAAGTGTTAGAAGCAGATAGAAGAAGAAGAAGACTTGGTGTGTAATACGGACAATTTCTCCAGTCTGAGACCGCGGTGTCGGTGGTGCCGAACTGATGTCGGTCTTGACCTTTCGGCCAGAAGCTCTCGGAATTTTCTCGCGACTTGGCTAAAATTCACCGGATGTCCGGTGTGCCGAGTGTCCGGTGAGCCAGCGATCAGCCAAGACCAGCGATCGGCCGCGCGTCAGCCGCGACACGTGGCCGAGCCAGCGGTCGGAAAAAGAAGCCGCCGGTTATCCGGTGTGCCGATTTGTCGGTCGCAATCTGCGATCGACAGCGGTCAGCCGCCATTTAAAGAAACATCGGGCACGGGCGGTGTCGATGTGCCGGATTTGTCCGGTCTTGCCGAAAGGCAGATGGCCTTCATTTGTTCAGCGGCTCTAGTGCCTTGGGGCTATAAAGGGACCCTAGGCATGGAGAGTACACCAAGCATTCCTACAACTCTTCTAAGCACCAAGACATCAATCTCATTCGTTTCATTGTGATAGCATATAGAGCTCTTGTGGAGTTGTGACTCTTTGTGTTGCGTTGCGAGCTCTTGTTGCGACATATGTGCGTGTTGTTGCTCTGATTTTGAGTCTTGTGTGCGTTGCTCATTCCCACCTTACTCAGTATTTCTTTGTGAACTCAATTGTAGGGGCGAGGACTCAAGTTGTGGAGATTCTGCATAAGCAGGAAAAAGATCAAAGGAAAGAAACAAGTGGTATTCAAGTTGATCATTGGATCACTTGAGAGGAGTTGAGTCTGCTCGTCAGTTGGGGCGCGCTGGAGTAGGCAAGTTTTTGTCTTGCGAACCAGGGATAACCACCGTGTCAACTCTGTGATTGCTTTCTTGTGGTTGTGTGTTTGAGTTCTCTCTAGCCACTTGGCCATACTTGTACTAACCCTTAACAAGTTTTGTGGCTTAAGTTTAAGTTTTCACAGGATCACCTATTCACCCCCTCTAAAGTGCTCTCGGGACCGAAGCGATGGATGATGTTGGTGAAGAACGCCGCTTGTTCGGACGATCTTTGTTTAGAGTCGGACCTCGATCCACTTGGAAATTTATCGATGGTGACCAGCAGTGCGTGTAGCCGGGGTGCCTACGCAAGGGCCGCGAGATCGGACCCACGACGGCAAGGCGGGTGATGGGTATCGTCTGCGGGGCTGGCGAGCGGTGGTCTGCCTGCGTGGCCCGACACCCTTCGCGAGTGCGGACAATTCTAGTGGCGTCGGCCACCGTGGGTGCGATGAAACCTTGTCGGGCGTTTCCAACAAGGGCTGAGGTCTTCGCGTGATGGGTTGCCAAGCCCAGTGTATCTCTTGTAGCTCGGCCTTGGCGATGGAAATGCATCGGCTGGAGGATGCTGAGGGCTACGGTGGTAAACTCTTTCCGTCCCCCACCAAGACAAAGCGACTTGGCATGCGCGCCAACCGCCGCTTGGCTCGGTCGGGTAGCTCTCCTCGGTGGAGATATTGCAGTCACGAGTGCCGGTTCGATGGCGTGACCCAGCCGCTCCCCTCTCGGCGTGCGTGCCTTATCTCGAGGCGGTACCTCGGGCCGGCTGAGTGCCTGCGGGCGAGCAGTGCCTCGGGCGAGCGGGAGGTGCCTCGTGGCCGAGGTGCCTCGAGCTTCCGGACGTGTCGTCGATCTTGGCGGAGGGTTGATGCTGGTCTCGGGAGAAGGCGTCAGGAGGCAGTTGTTCGCCGAGGCTATTTTGTGGTGTAAACAAATTGGCGCTTAGACAACTTAATAACACATTGCGGACGTTTTTAATGTACTGGATGCGCTTGAATTAATGGGGATCTGGATTTTAGTACTGGATTTTGGTTTTAGGAATTAGAAATTTTATTGATAGAAGTATTTTACAAATACAAATACATACTAAGGGTTTCTTATATGCTCAACACATGAGAAACCCTGGAACCTAATTCCCTATCTGGGAACTACTCACACATTATTATGGAGCTCGAGCTCCTATTAGGCGGTGGCCTCAGCGTACTCGGCGGCGGAGGGCTTCGGTGAGCGAAGAACTGGTGGGACTTGCGGATGTGGTGTGCGCCGGTGATGCGGAGTGGGCGTCTGTGCGGAGGCCGAGGAGGGTGAGAGCATGATCATGCGGTGATCGCCGTGGCCCGTCGTGATGATACCACCGGTGAGCAGAGCCAGTCACGGAGAGAGTCGGCGGTCTCGGCGTCGCGCGAGAGGCCGAGGCGCTCAAGCTCAGCAGTGTCGAGATCGGTCGCACTCCTTGAGGCGGAGAGTGGCCACGTTCTCCCAGGTGGTGTCGCCCTCGGCGAAGGCAGCAGCGGCGGTGAGGGCTTGCACGGCGTCGGTGAAGGAATCGCCGTCGCGAGTCCGCAGCGTGGAGAGGCGGCCGCGCGGTGAGGTATCGCCCTCGCGCCGATGTCAGCGCCCATCTCGCGAACACGTTCGGCCTCCTTCTCGCCCTGGAGGTCGTGCTCGCGGGAGGTTAGAAGGCTCGCCTGGGAGGAGGGCAGCGGCGGTGAGGATGGCAGCGGGCCGGGTAGTCGCCCGCACGACACGCGGCCTGGGCGGTACTTCTGGCCACCCGGATGGAGATGCGGCGGAGGTCGTCGGAGGCAGTGGCGCGCACGCCGAAGTCAGAGAGGGTGTCGAGTGTCTGGCAGGCGGAGCGTGGGACTTGATGTCGCCGGTGAGGCGGGTTCGAGTCCGTCCGGGAGGAGAGGCCGAGGAACATGAGGGCGGAGGCGTACTGGGAGGAGCGCTCGGCGGACACCTCCACGGTGCCACCGCGCACTGGGCCAGACACGGAGATCGGAGGCGACCGTCGTTGGAGGACACCCAGGCACCGAGGCGTTCGAGGGCCTCAGGAGGTCGCCCTGAGGGCGCTTGCCGAGGAGTCCGGGTAGTCGGTCACGAAAGTGGTGCCAGAGGTGAGCGGCCACGCCCATGAGGAGGCGGGCCACCGCGGCAGCGTTGCCTGGGTTGAGGTCACACCGGCCTGTGGGCGAGCGAAACCGCGGATCACGGCGTCATCGCCCAAAGCTCCACGCCAGCGCCCCAGTCGCGGAGGCAGCGGAGCATGGCCTCGGCGTCCTCAGGTAGCCACGCCCACCACGCGGGTCTCGCCCTCGAGGGCAGCGGCGAGGATGGCGAGTGGTGTAGTTCTTGGATGGCTGAGCGCGAAGCTCGCCGCGGAGTTCGCAGGCTGGATGCACGATCACGTCGAAGGTGGCGGGCAGGGCGTCGGATCCTTGCGGGGATCGGTGGGGCCCCACGGCCGGGCAGGGTGGCCGGGGAGCCATCGGCATGGCGGTGACGCCGCTGGGCACCTGATGGGCGCGGCGAGGCGCGGCGGGTGGCCAGGAGGTGCGCCAGCGCCTCGCCTTGGGCGCAGCGGTAGTGGCGCCAGTGAGCGCGGTAGACGCGGCGGCGGCGGTGGCCATGGTAGATCCTCTAGAGTCGACCTGCAGAGTAACGCAAACAACAGGGTGAGCATCGACAAAAGAAACAGTACCAAGCAAATAAATAGCGTATGAAGGCAGGGCTAAAAAATCACATATAGCTGCTGCATAATGCCATCATCCAAGTATATCAAGATCAAAATAATTATAAAACATACTTGTTTATTATAATAGATAGGTACTCAAGGTTAGAGCATATGAATAGATGCTGCATATGCCATCATGTATATGCATCAGTAAAACCCATCAACATGTATACCTATCCTAGATCGATATTTCCATCCATCTTAAACTCGTAACTATGAAGATGTATGACACACACATACAGTTCCAAAATTAATAAATACACCAGGTAGTTTGAAACAGTATTCTCTCCGATCTAGAACGAATGAACGACCGCCCAACCACACCACATCATCACAACCAAGCGAACAAAAGCATCTCTGTATATGCATCAGTAAAACCCGCATCAACATGTATACCTATCCTAGATCGATATTTCCATCCATCATCTTCAATTCGTAACTATGAATATGTATGGCACACACATACAGATCCAAAATTAATAAATCCACCAGGTAGTTTGAAACAGAATTCTACTCCGATCTAGAACGACCGCCCAGCAGACCACATCATCACAACCAAGACAAAAAAAGCATGAAAAGATGACCCGACAAACAGAGTGCACGGCATATATTGAAATAAAGGAAAAGGCAAACCAAACCCTATGCAACGAAACAAAAAAATCATGAAATCGATCCCGTCTGCGGAACGGCTAGAGCCATCCCAGGATTCCCCAAAGAGAAACACTGGCAAGTTAGCAATCAGAACGTGTCTGACGTACAGGTCGCATCCGTGTACGAACGCTAGCAACACGGATCTAACACAAACACGGATCTAACACAAACATGAACAGAAGTAGAACTACCGGGCCCTAACCATGGACCGGAACGCCGATCTAGAGAAGGTAGAGAGGGGGAGGACGAGCGGCGTACCTTGAAGCGGAGGTGCCGACGGGTGGATTTGGGGAGATCTGGTTGTGTGTGTGTGCGCTCGAACAACACGAGGTTGGGGAAAGGGTGTGGAGGGGTGTCTATTTATTACGGCGGGCGAGGAAGGAAAGCGAAGGAGGCGGTGGGAAGAATCCCCGTAGCTGCCGGTGCCGTGAGAGGAGGAGGAGGCCGCCTGCCGTGCCGGCTCACGTCTGCCGCTCCGCCACGCAATTTCTGGATGCCGACAGCGGAGCAAGTCCAACAGTGGAGCGGAACTCTCGAGGGGTCAGAGGCAGCGACAGAGATGCCGTGCCGTCTGCTTCGCTTGGCCCGACGCGACGCTGCTGGTTCGCTGGTTGGTGTCCGTTAGACTCGTCGACGGCGTTTAACAGGCTGGCATTATCTACTCGAAACAAGAAAAATGTTTCCTTAGTTTTTAATTTCTTAAAGGGTATTTGTTTAATTTTTAGTCACTTTATTTTATTCTATTTTATATCTAAATTATTAAATAAAAAACTAAAATAGAGTTTTAGTTTTCTTAATTTAGAGGCTAAAATAGAATAAAATAGATGTACTAAAAATTAGTAGTCTATAAAAAATTAACCCTAAACCCTAAATGGATGTACTAATAAAATGGATGAAGTATTATATAGGTGAAGCTATTTGCAAAAAAGGAGAACACATACACTAAAAGATAAAACTGTAGAGTCCTGTTGTCAAAATACTCAATTGTCCTTTAGACCATGTCTAACTGTTCATTTATATGATTCTCTAAAACACTGATATTATTGTAGTACTATAGATTATATTATTCGTAGAGTAGAAGTTTAAATATATGTATAAAGATAGATAACTGCTTCACAAGTGTGTGAAAAAAATATGTGGTAATTTTTTATAACTTAGACATGCAATGCTCATTATCTCTAGAGAGGGGCACGACCGGGTCACTGCACTGTAGGCATGCGTCGAGAGATAGATTTGTAGAGAGAGACTGGTGATTTCAGCGTGTCCTCTCCAAATGAAATGAACTTCATATATAGAGAAGGGTCTTGCGAAGGATAGTGGGATTGTGCGTCATCCCTACCGTCAGTGGAGATATCACATCAATCCACTTGCTTTGAAGACGTGGTTGGAACGTCTTCTTTTTCACGATGCTCTCGTGGGTGGGGGTCCATCTTTGGGACCACTGTCGGCAGAGGCATCTTGAACGATAGCCTTTCCTTTATCGCAATGATGGCATTTGTAGGTGCCACCTTCCTTTTCTACTGTCCTTTTGATGAAGTGACAGATAGCTGGGCAATGGAATCGAGGAGGTTTCCCGATATTACCCTTTGTTGAAAAGTCTCAATAGCCCTTTGGTCTTCTGAGACTGTATCTTTGATATTCTTGGAGTAGACGAGTGTCGTGCTCCACCATGTTCACATCAATCCACTTGCTTTGAAGACGTGGTTGGAACGTCTTCTTTTCCACGATGCTCCTCGTGGGTGGGGTCCATCTTTGGGACCACTGTCAGCAGAGGCATCTTGAACGATAGCCTTTCCTTTATCGCAATGATGGCATTTGTAGTGCCACCTTCCTTTTCTACTGTCCTTTTGATGAAGTGACAGATAGCTGGGCAATGGAATCCGAGGAGGTTTCCCGATATTACCCTTTGTTGAAAAGTCTCAATAGCCCTTTGGTCTTCTGAGACTGTATCTTTGATATTCTTGGAGTAGACGAGTGTCGTGCTCCACCATGTTGGCAAGCTGCTCTAGCCAATACGCAAACCGCCTCTCCCGCGCGTTGGCCGATTCATTAATGCAGCTGGCACGACAGGTTTCCCGACTGGAAAGCGGGCAGTGAGCGCAACGCAATTAATGTGAGTTAGCTCACTCATTAGGCACCCAGGCTTTACACTTTATGCTTCCGGCTCGTATGTTGTGTGGAATTGTGAGGCGGATAACAATTTCACACAGAGAAACAGCTATGACATGATTACAATTCGAGCTCAGTACCCTGGATTTTGGTTTTAGGAATTAGAAATTTTATTGATAGAAGTATTTTACAAATACAAATACATACTAAGTTGTACAAAACCAGCAACTCACTGCACTGCACTACTTCACTTCACTGTATGAATAAAGTCTGGTGTCTGGTTCCTGATCGATGACTGACTTCTCCACTTTGTGCAGAACAGATCTAGAGCTCTTAGTAGAGCGGGGAGATGTTGGTCGGCACGAGCATGATGTTCATGAGGTCGAACTGTGTGCCAGAGTTGAGGTCACGTTGATGTCGAGCGGCACGTCGGAGTTGGAGGAGGCCACCACGTTGCCGATGTTGATGTCGGAGAAGCGAGCACCGTTGTCGTTCACGCCGTCGTTGTTTGTGGTGGTGTTCACGTTTGTGGCGGTGTACACACGGCCGTTGATGGTCACGCGGATGGTGGAGTTGCCGATGGAAGACACGCGGAGGTAGAGGTTGTAGGAGTTGCCGTTACCGCGGAGGTGTAACGAGCGGTGGTGTTATTCTGCTCGAAGCGGAGAGAGTCGCCCTGGTTGCCGAACTTCTCGGAGATGAAGGTGCGGGTCTGGTTGTTCACCTGGGTAGCGTGGATCGGGAGATGGTGAAGCCGGTGTAGTCGTTTGGGCGAGGTGGGTCATGGAGCCGTTCTCGTGCACAGCGTGGATGTTGTTCTTGCGGTTGTGCACGGACACCATGTAGCGCGATGCACCTCAGGGTGCCGGACGGGGAGGCGATGTTGCGGATCTCGTTGTAGTGGAGTGGGCGACGGAGGTCCTCGTTGCGCACCACAGCGGCACGCCGGAGATGTTGCGGATGAAGTAGTCCGGGAAGTAGTTGGAGTTGCCGCGTGCGGTGAAGGCACCGGAGCGGAGGCCGAGGGTGGTCTCGAAGGACTCAGTCTGCCAGTTGGTCACAGTAGCCACGCCCTCGCGGTCGGGCCGGAGTCGAGCCAGGAGCGCACGAACGGTGTGTGAGGCGGAGGGAGGAAGGTAGAGCAGTTGAAGTTCTGGTTGAACGGGGCGCCGATGTCGCCGGAAGAGATGCCACCGGGTAGTTCACGCGAGCGGCGAGGAGGGCGTGGGTAGTTGTGGAGCCAGGAGGCCCACGATGTTCGGGAAGGTGTTGGAGGGCGGGCGCCGGAGAAGCCGTTGAGCACGTAGTTGGAGTTCACCTGGAAGAGGAGTAGAGAACAGCCAGTCCTGGGAGGTGAAGGACTGGGTCTGCTGCGGGCGGGCCGGAGGCGTAGAGGTTGGCGCCGGAGGACACGAGGAGGGACTGGTACTTGAAGAGGGACCAGATGGACACGTACTCGAACACGTTGAGAACATGTAGGTGCGGAACTCAAGCATCTCGTGGAGAGGGTGTTGAGCTTCTTGAAGGCTGTCTGGTAGGTGTTGATGCAGTAGTTGGAGTACTCCTTGGTGTAGTCCTTGAGGTAGCCACGGTAGGTGCGGAGTGGCAGCGGAGATACCCACTCCTCTGCGTTGAGGATCACATCGCGGATGAAGGAGAGGTGGAGGTTGGCGGCCTGAGCGAGGAAGTGGAAGGAGAGGTTCAGCCGCGGAGCTGGAACTGGGTGAGGCGGTTGAGGAAGAGCTGCTGCATGGTGTTCACCGGGGATGTGATGGAAAGGAGGTCAGGTTACGGTTCGGGTTGAGAAGTTGTCCACCTGACGGTTGAACTCGGCAACGTTCTTCTGGAGGCCCTCAAGTTCGGCGTTCACGCGGGAGAGGGTGTCAGCGTTGAGGCGCTGGTTGAGGAACTTCTCGGTCTCACGGAGGATGTCCTGCATGAGCTTGGTGTTGTCGTTTGGGAACACGAGGTCCCAGAGGCCATTGAGGATGCGCTTGGCAGGCCTCGAGCTTCTTGAGGAGGAAGATGGCCACAGTACCCACCACAGGAGCCACGTAGAGTGAGTGGTCGTCGCGCTTCCACTCGGTCCACTCCTTCTGGATGGTGTCGAGGACTTGTACTGGAAGAGAACGGGTCCTGGGCCATCACGTTGTGGCCGTCGCAGATGGTGGTGCGTCCGGAGTTGAGCACGGAAGCTCCTCCACCCTTCCGGGGCCTCGCACCTGATCGATGTGGTAGTCGGTCACGTCGGTCTTCAGGCCGATCTGGTTGCTGCTGGTGAACAGCTCGTTCACGGCCTTCTGAGCCCTCTCCAGGTCGTACTCGGCCTCGAAGGTCACCTCGGCGGGCACGAACTCGATGCGGTCGATGTACACCTCGTTGCCGCTGTTGAACACGTGGGCGCTCAGGGTGAACACGCTGCTGCCGTTGCTGAAGTTGAAGGGGTGGTGAAGCCCACGGTGCGGAAGCTGCCGCTCTGCAGGTTGCTGCCGCTGCTCATGGTGGCGCTGAAGTTGCCCTGGTTGATGGGGCGGCCGTCGATGCTGGTGTGGAACTGCAGGTTGGTGGTGCTGGCGTAGCGGATGCGGACGCGGTAGCGCTGGCTCAGGGGCGGTGATGTTCACGCGCAGGGTGCTGATCTGGCCGGGGCTGGTGCGGCGCAGGATGTCGCCGCCGGTGAAGCCGGGGCCCTTCACCACGCTGGTGCCGCTGCCCAGGTTGGTGCTCTTGGTCAGGGGATCTGGGTGATCTGGCTGCTGGGGATGATGTTGTTGAACTCGGCACTGCGGTGATCAGCTGCTGAACATAGGTGCACGGATGATGCTCACGCTGCTGTTGCTGAAGCCACTGCGGAACATGCTCACGTGGCTCAGACGGTGGCTGAAGCCCTGTCGAGGTGGCACGTTGTTGTTCTGAGGGGATCTCGTCCAGGCTGTCCACGGTGCCGCTCTTGCGGTACGGCTGGGCAGGTTGCTGCTGGTGCCGTAGGCGAACTCGGTGCCGTCCAGCACGCTCAGCTGCTGGTTGTTGATGCCGATGTTGAAGGTCGACGGTACAGGGTGCTGCTCAGGGTGCGGTACACTCCTGGCCAGCTGTGCCACGATGCGCTGCTGAGGTGCAGCGTTGCCCATGGTGCCGTACGCAGGGAAGGTGAACTCGGGGCCGCTGAAGCCGACAGGGCTGGCCGCGATCTGAGTGGCCGCTCAGTAGTACTCGCCGCGGTGGGCGTCGGTGTAGATGGTGATGCTGTTCAGGATGTCCATCAGGTGGGGCTGCGGATGCTGCCCTCGATGCCCTAGGCGCGCTGCCGCGGAAGCTGCCGTCGAAGTTCTCCAGCACGGGGTTGGTGTAAATCTCGCGGGTCAGCTGGCTCACGGTGCGGATGGGGTAGGTGCGGCTGTCGTAGTTGGGGAACAGGCTCACGATGTCCAGCACGGTCAGGGTCAGCTCGCGGCGGAACTGGTTGTACCTGATCCAGTCGCGGCTGTCAGGACCACGCGCTCCAGGGGTGTTGTACCAGCGCACGGCGTGGTCGGTGTAGTTGCCGATCAGGCGGGTCAGGTCGTTAGCGGCTGTTGATGGTGGCGGCGTCGAAGCCCCAGCGCTGGCCGAACACGCTGACGTCGCGCAGCACGCTCAGGTGCAGGTTGGCGGCCTGCACGTACACGCTCAGCAGGGGCACCTGGTAGTTCTGCACGGCGAACAGGGGATGGCGGTGGTCAGGGCGCTGTTCATGTCGTTGAACTGGATGCGCATCTCCTCGCGCAGGGCGGGGTTGGTGGGGTCGGCCTCCCACTCGCGGAAGCTCTCGGCGTAGATTTGGTACAGGTTGCTCCAGGCCCTCCAGGCGGCTGATGGCCTGGTTGCGGCGAACTCCTCGATGCGCTGGTTGATCAGCTGCTCGATCTGCACCAGGAAGGCGTCCCACTGGCTGGGGCCGAAGATGCCCCAGATGATGTCCCGGGCCCAGCACGAAGCCGGCGCCGGGCACGAACTCGCTCAGCAGGAACTGGGTCAGGCTCAGGCTGATGTCGATGGGGTGTAGCCGGTCTCGATGCGCTCGCCGCCCAGCACCCTCGGGGTTGCTCAGGCAGTTGGGGATGCACTCGTTGATGTTGGGGTTGTTGTCCATTGTTGGATCCTCTAGAGTCGACCTGCAGAAGTAACACCAAACAACAGGGTGAGCATCGACAAAAGAAACAGTACCAAGCAAATAAATAGCGTATGAAGGCAGGGCTAAAAATCCACATATAGCTGCTGCATGTGCCATCATCCAAGTATATCAAGATCAAAATAATTATAAAACATACTTGTTTATTATAATAGATAGGTACTCAAGGTTAGAGCATATGAATAGATGCTGCATATGCCATCATGTATATGCATCAGTAAAACCCACATCAACATGTATACCTATCCTAGATCGATATTTCCATCCATCTTAAACTCGTAACTATGAAGATGTATGACACACATACAGTTCCAAAATTAATAAATACACCAGGTAGTTTGAAACAGTATTCTACTCCGATCTAGAACGAATGAACGACCGCCCAACCACACCACATCATCACAACCAAGCGAACAAAAGCATCTCTGTATATGCATCAGTAAAACCCGCATCAACATGTATACCTATCCTAGATCGATATTTCCATCCATCATCTTCAATTCGTAACTATGAATATGTATGGCACACACATACAGATCCAAAATTAATAAATCCACCAGGTAGTTTGAAACAGAATTCTACTCCGATCTAGAACGACCGCCCAACCAGACCACATCATCACAACCAAGACAAAAAAGCATGAAAAAGATGACCCGACAAACAAGTGCACGGCATATATTGAAATAAAGGAAAAGGGCAAACCAAACCCTATGCAACGAAACAAAAAATCATGAAATCGATCCCGTCTGCGGAACAGCTAGAAGCCATCCCAGGATTCCCCAAAGAGAAACACTGGCAAGTTAGCAATCAGAACGTGTCTGACGTACAGGTCGCATCCGTGTACGAACGCTAGCAGCACGGATCTAACACAAACACGGATCTAACACAAACATGAACAGAAGTAGAACTACCGGGCCTAACCATGGACCGGAACGCCGATCTAGAGAAGGTAGAGAGGGGAGGACGAGCGGCGTACCTTGAAGCGGAGGTGCCGACGGGTGGATTTGGGAGATCTGGTTGTGTGTGCGCTCGAACAACACGAGGTTGGGGAAGAGGGTGTGGAGGGGTGTCTATTTATTACGGCGGGCGAGGAAGGGAAAGCGAAGGAGCGGTGGGAAAGAATCCCCGTAGCTGCCGGTGCCGTGAGGAGGAGGAGGAGGCCGCCTGCCGTGCCGGCTCACGTCTGCCGCTCCGCCACGCAATTTCTGGATGCCGACAGCGGAGCAAGTCCAACGGTGGAGCGGAACTCTCGAGAGGGTCCAGAGGCAGCGACAGAGATGCCGTGCCGTCTGCTTCGCTTGGCCCGACGCGACGCTGCTGGTTCGCTGGTTGGTGTCCGTTAGACTCGTCGACGGCGTTTAACAGGCTGGCATTATCTACTCGAAACAAGAAAATGTTTCCTTAGTTTTTAATTTCTTAAAGGGTATTTGTTTAATTTTTAGTCACTTTATTTTATTCTATTTTATATCTAAATTATTAAATAAAAAACTAAAATAGAGTTTTAGTTTTCTTAATTTAGAGGCTAAAATAGAATAAAATAGATATCTAAAAAATTAGTCTATAAAACCATTAACCTAAACCCTAAATGGATGTACTAATAAAATGGATGAAGTATTATATAGGTGAAGCTATTTGCAAAAAAAAAGGAGAAACAGAACACACTAAAAGATAAAACTGTAGAGTCCTGTTGTCAAAATACTCAATTGTCCTTTGACCATGTCTACTATTGTTCATTTATATGATTCTCAAAACACTGATATTATTGTAGTCTTATAGATTATATTATTCGGTAGAGTAAAGTTTAAATATATGTATAAAGATAGATAAACTGCACTTCAAACAAGTGTGACAAAAAAATATGTGGTAATTTTTTATAACTTAGACATGCAATGCTCATTATCTCTAGAGAGGGCGACCGGGTCACGCTGCACTGCAGGCATGCAAACTTGCACTGGCCGTCGTTTTACAACGTCGTGACTGGGAAAACCTGGCGTTACCCAACAATCGCCGCAGCACATCCCTTTCGCCAGCTGGCGTAATGCGAAGAGGCCCGCACCGATCGCCCTTCCCAACAGTTGCGCAGCCTGAATGGCGAATGCTAGAGCAGCTTGAGCTTGATCGATTATGCGTTTCCCGCCGGTTTAAACTATCGGTCGTTGTAGCGTCGAGGCGGTTGGCTGGCCGTGAAACTTGTCTTCCGAACGCCGACGTCGTCGCGGTGAGCCTCCATCTTGGGTCGGCGGCGGTGGGAGTTCTTCATGACTTCGTGGGTGTGTTCAGGTACCCACGGCGTCGGCGTCGGACCCCGGCTCGATGGCGATCGCGGCAGTTGACGAGCCTCGTACCCGACCATTGTTCGGCCAGCGGAAGTGGGCGTAGCACATAAGCGTGGGTGCTTCAGGCGAGATGAAGAAGCGAGCTGCAGCGCGGCTCCTGTCTTCATCGACCGTCGAAGAACATGGTCGAGTTCGGTTGGATCGGGCCGTTGGGCCCAGGTGTCGACCCATTCGACCACAAGTCCGCGAACACAGGACTTGATGGCCTTCCGAAGCGGCGGAGATCGCCGCCCATGATTTCCACAGCCCACTGCGATTCTACGGGCCTCTCGGCACTGATGATCTCCCGGGAAGGATGACACCGATTCACCGGATGAGACTCAGTAGTGTACGCCCAACCGCGTCGGGATCACCGCGTCTGGCCCGAACCACGAATTTGTGGGTAGCGGATCTTGGTCTCGAACGATACCTCGTGATGAAGTAGACTTGCCTCGGGCGGACAATGTGCCCCTCTTTGTCTCTCTCAACCACGATCGCGGCGTCTTTGAGTGGTCGCGGCGGCGTAGGTCAATAAGGCTTCTCGTGAGGGGGCACCAAGATAAGCGCGTTCGTGAGGCGCCTTGAGTTCAGGAGCTTCCTCGGCCTCGAGTCAAGTAAACACTCGGCCTTCCTTAAGCGGTATAGGCGAGCCTCTTCGGCAGAAGCGCGTGAATGAAGCGGCTCGAGCGCAAGACATCCGTGACTCTGTGCGCCCTTCAAGTCCTTGATGGGCCCCATCTTTGATGGCGCGATCTTCTCGGATTGGCCTCGATGCCACCGGGAGACAATGAACCCCAAGAGCATGCCTCGAGAACCCCAGACACACTCGGATTAAGCTTCGTCTTCGCCTTGAGACATCGGAATGTCACTTCAAAGGTCGGAAAGGAGGTCTGAGGCTTTCGTCTTGACGTACGATGTCATCGCGTAGGCCTCGGCCGTCCGGCCAATGTGTTCGCGAACACATGGTTCATCACACATGGGATGCGTCGCACCCGCATTCCTCAAATGAGCGGTATGGTGACATAGCGGTACATGCAGAAAAGTGTGATGAAAGAAGTCGTGGCTGGCCGGATTCTTTCATCTGATTTACAGTGGTACCACTGAGTAGGCATCGAGGAAAGACAGGTTTCGCACCCAGCGATGGAATCCAATTTGATCGATGCGAGGCGGTGGGAACAACCGGACATGCTTTGTTTAGACAGTGTAGTCTACACACATCGCCCTTCCCTCCTTTCTTTCTCCACAAAGCGAGGTTGGCAAGCCATTGGGATGGAATACCTCTTTGATGAACCTGC

>nano8

AACCTCGGGTTCTACCAATCCTAACGAGGGTCCTTCTTCTACCTTAAGCGATGGCGCGGCGGAGAAAAGAGGCGAAAGGCTGCTGGCGAGGTTGCTCGGTGAAGTGAGTCGAGGAGAAGGGAGGTCTCGAGGATCTAGCGGTGTGCGAGTTTGCGTCGGAGATGGCGGGTCGGTCGATCCGCAGCGCGGCGAGGATGCTCGTCGCGGCGAGGGACGGCCTTGTCAGCGAGATAGTTCAATTAGATGGGTCGGGAGGTTCCGGGATGCCGAAGAAGATATGGCGAAAAAGGTGGCGGAGACTCAGCTGATAGCTCGGTCCACCTTGTGGGGACCAAGTCCGGTGGTCGATCTCGAGCCTCCGGTGAAATCTGGCGGTCGAGGGCAGCAGCTTCACAGGCTACTGCGAGCTAAGCCAGAAACAGGTGGGCTTGAGGGTGGTGGTGGGTGCAGCCATGGTGGCCAGCTCTGTGTGTAGCCAGGTCCTCGCGGAGCTAGGAAAGGTGGGTGGCGGAGAGAGGTGAGTCTTGAGCGAAAGAAGGTCTTGCAGGGGAGGCTTTATAAAGCGGCGCGGGTACAGCAGGGCGTGGCTGTGGCCGGACAATGGGCAGCCGGGTGGCGAGGTTAGGCGAGCTCAGGCGTCACGAGACAGTCGAGGCACGATGTGCCGTGCGTTCTGCCCAGTTACGGCGTGTGGTCGCTCCGACTGCTCTCGCCTTAGTTGGTGCACAAAACCTCTTCTCCTCCCCAAGCTACCATTCTTGTGTAGGGTCTTAGGTTTTGCCTCGGTTGCGAGAGATATGGAGCGAAATCTGGTCTGTCTGCTGAAACCGAAGCAAATCCAAGGTTTTGTCGTGTCTAGGGCTCACGTCCCAATGCCATCTTACGGCACCAGCGGAGGGTTAGTTAGACACAATTTTGTCAATGGGCCATTAGGATTCAAGTTAGGGATCAAGGTGAACATCCTGATCTTTGGCTCAAGGTCTGAATTTGAGAATCTACAGGTGAAATCTAATGAGTCCCACATGAGGAGCTTGATTTGGGGTTTATTTGAGTTATTTTGGCTAAGCTTTCTCAACATTTCTTGTTGCCCATTAAATATTCTTTAATTTTCATAATTGGCTCAAGTCATAATTTTAAACTTCATTCCCTTTTCTTATTTTCTTGAATTTTGCTCATGGGGCTCACTTAGGGTTCTTAATTAGGTTGCACATTTTTATCTTTTCAAAGACTCAATTGTTTTGATCATGTTACTTTTAAGTATTTACTTGGTGAATTCTTTATTACTTAAGTTGTTTGATGCTCATGCTTACTTTGGTTCACATAAAATGATGGTTCTTGGTTTGGCATTTGAGAAAACGAGTGACCCAGGTGTCCTGATCTGAGCCACAGGGTCAGGCGAGCGAGTTAAATCGTCTTACAAGTGAGCAGTCAGAGCCCCAGGTCGGTGGGCGAGTTCGCCATCTTCCGAATTTGAGCAGTCAGAATACTGGGTTAGGCGGGCGGAGTTGCAGTCTCGGCGAGCCGTCGAGGCTTCCTAGGGCTGGGGCGGAGTTCGCCGTCTTCGGAGTTGAGCAGAGTCCGAGCCTAGGTCGAGCGAGCGAGTCGCCATCTTAGTGAGCCCGTCCGAGCCCTAGGTCGGGCGGCGGAGTTTCCTATGGTGCAGCGGGCTGGCCGCTGTCACCTCACTCATCGAGTGCACTGGCCCGATCGGAAGCGCCAGCGGACGCATCCTTCTGTCGGCCGTTGAGTGGGCGGCGATATTGCGATCACCGGCTCTGTCGGCGGAGCGTCGTCGGAGATAAAGTGTGAGCCACCTCGCATTAAATGCCCTGCGATTTGGTCGGTTAGCGCGGCGATTTGGCGGGGTTGCTTCTTAGCGAGAGCGGGCCTCGGCGAGCCGGAAGTATGTTCGTCGTTGGAGGGGGCTCGGCGAGCGGAGATCCTCCAGGTGTTGCCGCCCGAGGCTAAACAGCGGGCGTGTCGAGTCCCTCGAATGGACCGATCCAACTTAGTCGCACCATCGGGCCTTTGCGGCTTTGTCGCGATGGGTTACAGTTGAAATTAGGAGTCTTGAGGGTACCCTAATTATGGTACCGGCGGTAGCCCCTCGAAGAGTGTTAACTGCGGGAGGCTTTATGCAAAGGGACCCGGCCTTTCTCGGTTGCGTTTTGTTCCCGGTGGTGCGCGCGGCGCCACTGGCGAGTGTAACCAAGGCCTCGGAAGGTGGTTTGACTCCTCGAGGTCTTAATACCTCACGCAATGCTTGGCCGTCTAATCGTTCCCTCATCTGGTGGCCGTAGCCGGAGTCGCAGTCGGGTCCCAAGTTCTTGAGCGTATGTTGGCGTTGTCAGTTTGGCCGGGCCGGGTTCAGGCAGCCGAACCTCCATACAAGCGAAGAGGGTCGAGACGGACTGACTTTTTAACATCGCCCCTAGCGTTCGCCTTTCGCAGGAAGAAAAGCGCCATGTTGCCCTCGGGCGCCAAACATGGTGTCTCGTCATTCTTTGGCGGGTAATCAAGTGGGCGCTTCGTGCCCCATTTGTTGAAGTCGGCTAGAGGCGGGCGCGCTCAAAAAGTACCCTCGGGTGATCTGCGGACCGGTCCTGTCGGCCCGAGTCAGGCTCGGCCTCCTCGATGGGATTAGTTTACAAGATCATTCTGCTGGTCTCGGAAATGTCTTAGGTACCTCGGGGCGTGGCCCGAGCCTTGGTTATGTAGAGCGCGTACCCATGGTCATCCCTCGCTCACTGTGCGAGGCGGTGTGAACCCTTCGAGGGCCCGACCTTGAACCACGATCGATGAGTGGGCGCGGGCCCGTGGCTGGCGGCCGTTGAACCCTCAGGGGCCGACCTTGAACCTCTAACGGTAGTGGGTGTGGAGCCCAGCGCTCTGCGGCTGTTGAACCCCTCAGGGCGACCTTGAACCTCTTATCGGTAGGCTCGGAGCACTGTTTCCTTCACGGAGAAGGATCCTTTTGGGGTATCCCCTTTCCCGGTCCACAGTTATAAGAGAGAGAAAGAGGAAAAAGAAAAGGATGCAAAATCGAATGGCGTGGCGTACCTCTGACCGGTCATTGTGGAAAGCGGCGTCGCTGCGCCCCTGCCGGAGGCTGCCTTTCCCGCGCGGAGTTAATGCGGCGAGGCGGCGAAGTTCAGCGAAGCGGCGCGTGCGGCGTCGCGAGCGGTCGAGAGCGGAACCAGGCCGTCTTCGCCGTGAGAGGGTTCTCTCACGGAGATGGGGCGTAAGCAGGCAGCAGCGTGACCGGCTGCTCCGCTGCCACCGCCATTCACTGCGGCCCATTTTTGGCGTATTGACCATCGCGCTTTGGCGGCGCCTTCTTGGCCGTCGTCTGCGAGGTCGCCTCAGTCGCGATCGCCGGTTCCGCGATCGAGGCGTGGCGGTGGTGCGGTGGCGTCGATTGCTTGCGTAGCGGCCGTGCGCCCGATGCGTGTGCGATGGCGTGAGCCCCATCTTAGTGCGTTGGAAGTCGGAGGCGCCCGCAGCGCGGTTGCGCATCGCTGCATACGCCATGCTTCGCGCGGCCAGATGCAGGCGAAAGTGGAAGAATCTCGTAACATTGGCGGTTGCATGCCACGGTTTGGCTTCTAATACGCTCACAGGCGGCTTATGCGGCCAGCGTGGGTCCCGGCCCAGCGTCGTGGGGAGGACCTTAGGCGTGTTGGAGAAGACTTCGCCCACGGCAGTGGGGGCATAGTAGGAGAGTCGCCTTTAAGGTGGGTGACCCCTTGAAAGGCGACCATGTCTTCAGCTCCCTTATGTCGTGTCTCCACCTTAGAGCCCGGATGGGACACCCATGATCCCTCGCCTTGTCGTTTGGAGAGCAAACTCCGTGGGAGTTGGTACCTTCGCCATCGTTCGGCTTCAAGGATTTTCATCGCGGCCGGCTGCACCCCTCTACCGGCGGTCACCCAGATGGTGACCTCGGTTGATGGCGAGGGAAAGCAAGCCGGCTGCGGCCTCCCTCTCGGCCTCAGGATTTTCATCGCCGGTCTTTGGGGAAGTGTGTCGAGTTAAGGTCGGCCACATGCGCGTGGCCCGCTCCTTCCCTGATGTCGAGGAAGCGTTCGCCCGTTCATGGTGGCGGCTACCGCGTGCAGCTCTCGGCCGAGTGGCCGACGCTCGGCGCCGCTTCCGCCATGGCGGTGGAAGAGGTTCTTCGCCGAAAGATAGCGGGGCCGCCGCGGGCCAAACCTCAACTCTCCACGCCTGCGATCCTTGCCTCGGTTTGGGCATGGGCGAGGCCTCATGCGCGACATCCACCACGGGTCATCGCTGCTAACATTAGGCCCGGGCGAAGTCGTCATCATCGCCCGTGTTGGAAGCGGCGGCAGGCCGTTCGTTGATGTCATTGCTCACGCGAATCCCCAATCGTGTAGGGTTGTTCATTACGCGGAGGTGGAGCAGAGTTCCGTTTGTAATGGCACCTTGAATGTCGGTCTTTTGTTCATTGTTTGTGGGGCACGAGCGTGTATATATTTTTGCGCGAGCCGTGTTTTTCCTTATTTTGAGCACTAAGACTCACTTTGTTGGTTGTGCGAAGCTTCACAAGAGTGAGTCGCCCGTACAAGGTGACGAGTGAGGTATCCGTATCCGGGGCGTAAGAGTCTCCATTCGGTCGGCCTGGCCGTCAAGGCTTCTCTTGTTAGTTAAAGAACCCTCGGCGCTCTTGATGGCCGGCCCAGATGTCGGTGCAGCAGCAGGGTGGTGCAGGGAAAGAGTTCAGTCGCCGGGCTGGCGGGCGTCGTCGATCGGGCCTGGACGCGCAGTTGATCTGCAGGCTTCGGTACAGTGTTCCGAGGGCAATGGGCAGAGCCTCGGGTGACGGTGAGCCGCCTACTCGGCCGGATTCTTGGAAGACCTGGCGGCGATGGCGAGCGTGATGATGATGTCGTCGCCAGTGGAGATCCTCGGACCGCGTCGCCGTCAGAGCCTGGTCGGACCTCGGTGAAGGTGTAGTCGGCAGCGAGGTCTTTCACCCCTCCTTCAGCATCAAGATCAAAGCTGCGGATCGGATTATCTTGTAGTGTGCGTTTTACGCGTAAAGCTAAAACACACCATCGCCCGTGTTGTAAAGCTGCTTTTCCTCTTGTTTTGAGTATCCGACTTTTTGATGCGGAATTATGCTGCGGCGGGAGTTGCTTTTCACGGAGGTGGCGAGTGAGGTATCCGTATCCCGGTAGGAATCCCTCGGCTCGGTCGGCCTTGCCGCTACGTGCCTCTTGCCCGTCCCAGGGTTACGCCGCCGCCTTGATCGAGAGGCGAAGAATCGGCGGAGGAGCTTCGAGTGTGAAGACTTGTTCGGTGCAGATAAACTTATCGAGCGAGTTACTTATCACGAAGGTGATGAGTGAGGTATCCGTATCCGGGCCGTAGGAGTCCTCGGCTCGATGGGCCTTAACGCTTGCGTGTACTCCGTCGTTTGGGATCCACTTTGAAGTAATAGCGAAGAACGATGGCCCGAAAGGATCTTTTTGAGGAAAATTTCGGCTGAGAGGGTTCCCTTTTAGCCCAGGGAGGGTCGGGCTTTAGAGCAAGTGACCCTTCCTTGATACTACGTGGGAGCGAGGTATATGAACAACGAAAGCATCTTAAGGGTAGAGCGGCGTGGTGTTGGATGTTCCAAGCGTTAACGTGGACCTCGCCTTATTTGTTGGCGGCATGCGTTAGGGCGAACTTTGGCGATAATGACGGCCCTTCGAGGAGCGCGTGAGCTTAATGCGCCCTCGGCGTCTGTCGCGGCGAAGCAGGTCGCCCACGTGGAAGTCTCGGGACGAGACCCTCCGGGTGTGGTAGCGTCGCGAGGCTGGCTGTACCGTGCCGGAATGTAGTAAGGCCATGTCGAGCCTCTTCCGGTGGTCAGCGAGTCTTCGACTAACTTGGTTGCTTTGGTGCGTGAGGCCCTCGTCCTTGGGGGGCCGTATTCTAAGTGCATGGGCAAGATGGCCTCGGCCCCATAGACTAGAAAGAGCGGCGTGAAGCCCATGGCTCGGCTCGGCGTTGTCCTCGGACTCTAGACCAGAGGAGTTCCTTCATCCCGCTTGCGAACACTGTTGAGTCGTTGTAGATCCGAGCTTGGTCCTTGTAGAATCATGCAGTTGACGCTTCTCTGCCCATTCGGTCATGGGGTGAACCACGCGGCCGTCCACACCGGATGTGGTGATCTCATGAGAAGTCGGGAACTTTTGCGGTGAGCTGGGTCAGTTGTCGGTGATGATGGGTTCGGGACTGAGGAAATGTGCCTTTGGGCCATTTCTAAGTATTTTGGTGATTGAGTGCAAACACAAGTGCTTAAATGTGAAAATATGCCCAAGGATGAACAAAGTGCAAATCACAAGTTAAGGTATGTTTCTAAGCCTTAGTACATTGGTTTTGTGTACTAATATATTTGTCTAAGTGTTGAAGCAGATAGAAGAGAAGAAGACTTGGTGTATGCGTGAGAACGCCGATGCGAAACACCGAGGTGTCGGTGGTGCCGGGCGTGTCGGTGGTGCCGGACATTGATGTCGTCTGTATTCTCGGCCGGCCAGCTCTCGGGTTTTTCTCCGGCGACGGCTAAAATTCACCGGTTGTCCGGTGTGCCGGCTATCCGGTGAGCCAGCGATCGGCGGGCCAGCGGTCGGCGCCGCGATCGGCGTGCGACACGTGGCGAGCCAACGGTCGAAAAACACGGGTATCGGTGTGCCGGTGTCCGGTCGCGCGATACTGCGATCGGCAGCGGTAACCGCCATTTAAGAAACAAATCGGACGCGGACGGTGTCCGGTGTGCACCGGGCCCATCTCGGTGCGCCGCGGGAGCGAAGGCAAGATGGCCTTCAGTTGTTCCCAATGGCTCCTAGCGCCTTGGGGCTATAAAAGGGACCCTGGCGCATGGAGAGTACCAAGCATTCCTACAACTCTTCTAAGCACCAAGACATCAATCTCACGCATTCGTTTCATTGTGATAGCATATAGAGCTCTTGTGGAGTTGTGAACTCTTTGTGTTGCGTTGCGAGCTCTTGTTGCGACTTGTGTATGCGTGTTGTTGCTCTGATTTTCGAGTCTTGTGTCGTTGCTCATTCCCACCTTACTCAGTATTTCTTTGTGAACTCAATTGTAAGGCGAGAGACTCCAAGTTGTGGAGATTCCGCAAGCGGGAAAAGATCAAAGGAAAGAAAAACACCGTGGTATTCAAGTTGATCATTGGATCACTTGAGAGGTTGAGTGCAACTCTCGTCGGATTGGGACGCCACAGCGTGGAGTAGGCAAGTTTGTGCTTAGTAGAACCACGGATAACCAGTGTCAACTCGTGATTGCTTTCTTGTGGTTATTGTGTTTGAGTTCTCTCTAGCCACTTGGCCATACTTGTACTAACCCTTAACAAGTTTTGTGGCTTAAGTTTAAGTTTTCACGGGATCACCTATTCACCCCCTCTAGGTGCTCTCAATTGGTATCGGGCCGTTCTCTTCAAGAAAGGACTAACGCTTGAAGAGATGGATCCTAAGGGAAGGAATTGTGATCAGCGACAGGAGAAGGTCGCCGTCAGGCCAGGGATGACAAGTCCAATGACTCGGGCTCGGGCCTGGCGAAGAGATGGGAAGAAGAAGACAAGGCGCATCAAGGAGATCGTCCTACTACGGCTGATGAGTCCTCTTCTTCCCAAGGGCAGCGACCACGACAAACAAAGGAAAGCGGTTAATTCTAACTTTTCTTTTGACTACTCTCGTATTCATAAAGTTCAAATTCACATTTGCTTTCTATTCCACTCGGCAAGCCCCACTTTGATGGGGAGGACTACGGATTTTGGAGCCACAAAATGCGTAGTCACTATTCTCTCTCCATCTAGCATATGGGAGATTGTAGATAGTGGAATGCACTTTAATAGTTCGGATAGTCCTATATTCATTAATGAGCAAATCCATAAGAATACAAGCTACTCTGTTCTTCTAGCCTCATTGTGCGAGGATGAATATAATAAAGTGAGTGGCTTGGATAGCATGCAAATGCAGAGACCTCAAGATCTCTCATGAGGAAATGATGCTGCTCACCAAAATGGAGTTGGTAGAGGCGAGCTTGGGCGGTTCGCGATGATAAGGCGAGAGCCAACTCAAACATACAGCCGGCTCAAGACCCTTGTCAACAAGATAAGGAGCTATGGAAGCCGCGATGGGCCGGACCACGGCGTCGTCAGCCTCATGCTAAGGTCGCTATCATTCTTGATCCTCATTTGGTGAATAATATTCGTGAGAATCGGGTACACAAAATGTGCTTGAAGAAATTCTTGGAAAATTTGTAGCGGGCGGATGATGATCAAGGAGGCAAGGTGCGTCGATGGCGCGTTGAGCAGTCCAATCCACGAGCCTCAACCCATTGCTCTCAAGGCATCGAGAGCAGGAGGCACTACCAAGCAGGTGGTGCAAATTGGTAATAAGGCTTAATGATGAAGAAATGGCTCTCATCATTAAAGCGCTTCAAACAGTGCTAAAGAGATGCAATGGACGGTAGAGCAAGACTAAGACCAAGGGAAGCGATCATGCTTCAAATGCGGTAAGCTTGGTCATTTATTGCTAAGCTGTCGATAATGAAGTGACCGGGAAAGGGACAAGAGGGAAAAGAAGAAGCATTATAAGAAGGCAAAGGGTAGGCGCATCTAGGCAAGGAGTGGGACTCGGATTGCTCCTCGTCAGGCTCTAGACAATGAAGGACTACCGCCACCGCCTTCAACAAATCAACCCTCTTCCCAAGCGGCGTCACACATGCCTTATGGCAAAGGGAGAAGAAGGTATGTACGCAACTCTACCTATGCTTCTTCAAGTGAGGACGAATCTAGTGATGAGGATGAAGTAGATTATTCATGTTTGTTCAAGGGCTTAGATAGATCTAAGATAGACAAAATTAATGAATTAATTGATGCCTTGAATGAAAGAATATACTTTTAGAAAAGCAAGAGGATTTGTTGTATGAAGAGCATGATAAATTTGTTGAGCACAAAAAATCCTATGCTTTAGAAGTTAAAAGAAATGAAATGCTTTCTTTTGAACTATCTACTTGTCATGAAACCATTTCTACTTTGAAAGGTGTCAACAATGATTTAAATGCTAAATTAAAGTAGCAAATAAATCCAATTCTTGTGTAGAACATGTTGAAATTTGTACTAGGTGTAAAGATTTTGACGTTGATGCTTGTAGTGAACACCTAGTTTCAATTTCCAAGCTTAATGATGAGTGGCTAGTCTTAATGCTCAACTTAAGACTAGCAGAATATTCGATAAGCTAAAATTTGCAGGGATGCCTACAATTGGTAGACACCTCAATTAAGGATGGACTTGGCTTCAAGAGAAGCTAAGAACAACAAGCCATAAGGCTCCCATTCCCCAAAGGAGAAAGGGAAGGTCCTATAGCTAATGTGTGCAAAAGAACCATGCCTTTTTATCATGATAGAAGACAAACTAGAAATGTAAGTCATGATGCTTTTGATTCATATGTTTATGATTCTCATGCCATGTTTGCTCCTAGTTCCTCTTATGTGTATGATAGAAATGTTACTAGGAGAAATGTTGTTCCTAAAAGAAATGCTATTCATCATGTGCCTAGAAAGAATGTTATTCATGCTCCTAGGAAAGTAGTGAATGAACCTTCCACAATTTATTATGCTTTAAATGCTTCCTTTGCTATTTGTAGAAAGGATAAGAAAATTGTTGCTAGGAAGTTAGGGCAAAATGCAAGGGAGACAAAACTTGCATTTGGGTCCCTAAGGATATTGTACTAACCTTGTAGGACCCAACATGAGTTGGGTACCTAAACCCAAGCCTAAATTTGCCTTGCGGTTTATGCATCAGGAGTTCAAATTTGGATTATCGGCAGCGGATGCACAAACCATATGTGAGGAGAAGAAGATGTTCCTCCTACGTCAAAAATAAGGATTCCCAAGATTCAATTATATTCGGTGATGGGAATCAAGGCAAGGTAAAAAAGGTTAGATAAAATGCGATTTCTAATGAGCATTCTATCTCAATGTATTTTTAGTAGAGAGTCTTGGATATAATTTGCTATCTGTAGTCAATTATGTCATATGGAGTATCTTGTCTATTTACAAATGTAGATGTACATCTTTAGAAGAAGTGATGGTTCACTAGCTTTTAAGGGTGTATTAGACGGCAAACTTTATTTAGTTGATTTTGCAAAAGAAGAGGCGGTCTAGATGCATGCAATGGCTAAGACTTACATGTTTGGTGTGGCATCGCTAGCTTAGCATGTGGGGATGAAGAACCTTCACAAGCTTCTAAAGGGAGAACACGTGATAGGATGACTAACGTGCATTTGAAAAAGATAGACCTTGTGCGGCTTGTCAAGCGGGTAAACAAGTGGGAGGAGCACATCACGACAGAGCGTGATGACCACTTCAAGACCCAGGCTGGCGCATATGGATCTCTTGGACACATCGCCTATCGAGCATAGGAGGAGTAAGTATGGTTTAGTTATTGTTGATGACTTTTCCCTTCACTTGGGTGTTCTTTTTGAGGATAAGTGCAAACAAAGGACCCTCAGCGCTTTCTCGGGAGAGCTCAAAATGAGTTTGAGCTCAGGTGAAGAAGATAAGGAGGCGACAATGGGTCGAGTTCAAAACCTTCAAGTGGAGGTTCCTTGAAGATGAAGGATCAAGCACGAGTTCTCCGCTCCTACACACCACACAAAATGGTGTGGTAGAAAGGAAAAACGGGGCTCATCGACATGGCGGGGCGATGCTAGGGAGTTCAAGACCCCAGAGTGCTTTTGGGCGGAGCCGTTAACGCTACTTGCCACGCCATCAACGGGGTCTACCTTCATCGCCTCCTCAAGAAGGCGTCGTATGAGCTACTAACAGTAACCCAATGTATCGTGCTTTACGTGTATTTGGGAGTAAATGCTACATTCTAGTGAAGAAGGGTAGAAATTCTAAGTTTGCTCCCAAAATTTGTAGAAGGTTTTATTAGGTTATGACTCAAATACAAAAGCGTATAGAGTCTTCAACAAATCATCGGGTTTGGTTGAAGTCTCTAGCGACGTTGTATTTGATGAGACTAATGGCTCTCAAGAGAGCAAGTTGTTGATTGTGATGATGTAGATGAAGAAGATGTTGGCGTTGTGCTATACCGAACCATGGCGATTGGAGAAATGCGGCCCGGGAACAAGATAACGAGATCAATCATCTTCCTCAACAATGGTGCAACCCCAACTCAAGTGGAGCGCTTGATCAAGAAGGGCGCAAGATGATCAAGTGATGGAGAAGAAGCGCCTTGGCACCTCCAACCCAAGTTCGGCGATGATTCAAAGGGATCATCCCGTCGACCAAATTCAGGTGACATTAGCAAGGGAGTAACTACTCGATCTCGATTAGTTAATTTTGTGACATTACTCTTTTGTCTCTTCTATTGAGCCTTTCGGGTAGAAGAGGCCTTGCTAGATCCGGACTAGGTGTTGGCCATGCAGAGGTTAAACAACTTCGCGCAATGAAGTTTGGACAGTGCCTCGTCAGAAGCAAAATGTTGTAGGAACCAAGTGGGTGTTCATGACAAGCAGAACAAGCACAGGGTGGTGACGAGGAACAAGGCTCGACTTGTGGCAAGGTTATGCCCAAGTCGCGAGTTTGGACTTTGAGAGACTTGCTCCTGCATGGCTAGGCTAGAGTCCATTCAGTATTTGCTAGCATATCGCTCACCATTCTTTCGAGTTGTTCCAAATGGATGTGAAGAGCGCTTTCCTCAGCGGGCCAATCAAGGAGGAGGTGTCGTGGAGCAACCCACTGCCGAGGATGAGCAGTACCCGACCATGTGTAAGCTCTCTAAGCTCTGGACTTAAGCAAGCCCCAAGAGCATGGTATGAATGCCTTAGAGACTTTTACTTGCTAATGCTTTCAAGGTTGGGAAGGCGATCCAACTCTTTTACAAAGACATGTGATGGTGATTTGTTTGTGTGCCAAATTTATGTCGATGACATAATATTTAGTTCTACTAACAAAAGTCTTGTGAAGAGTTTAGCGAGGTGATGCGCGAAGTCGAGATGTCAATGATGGGCGAGTTGAACTACTTCCTTGGGTTCCAAGTGAAGCAACTCAAGGCGGCACCTTCATCTCCCAAGCGAAGCACATGAATCTGCTAAGCGGTTTGGGATGAAGGGCGCCAAGCACGCAAAGACTCGATGGGGACGACGGACACCAGAGCCTCAACAAAGGAGGTAAGTCAGTTGATCAAAAAACATACCGTCAATGATAGGTTCTTTGCTTTACTTATGTGCTAGTAGGCCGGATATTATGCTTAGCGTATGCATGTGTGCTAGATTTCAATCCGATCCTAAGGAATGTCACTTGAGTGGCGGTGAAGCGAATTCTTAGATATTTAGTTGCTACGTGCTTGAGACTCTGGTATCCAAGGGGTCTACTTTGACTTCAGTTGGATACTCGGACTCGACTATCTTGGATGTAAGGTCGATAGGAAGAGCACATCGGGGCGTGCAATTCTTAGGAAGGTCCACGGTGTCATGGAACTCTAAGAAACAAACTTCCGTTGCCCTATCCACCCCGTGGTAGGAGTATGTTCCGCGGGAGACGGTGTTGCAGCAGCTACTTTGGATGAGGCAAACCCTCGGGGACTTTGGCTACAAGCAAAGTCCCACTCCTATGTGACAATGAGAGTGCTATCACGCATGGCGGAAGAATCTGTTGAACACGGCCGCACAAAGCACATAGACATCGGCATCACTTTTGAGACCACCGGCAAAGGGAGATATCGAAGTGTTTCATGTTAGCAGAGAACCATTAGCCGATATCTTCACTAAGCCTCTAGATGAGAAGACCTTTTGAGTTGCGTAGTGAGCTAAATGTCTTAGATTCGCGGAACTGGATTGAATTGTAGCATACATGTAGTTATGCTTTTGATCATGTTCCTTTTGCATTATGTTGCTTATTATGGTGCTCAAGTTGTACAAACACTCCTGGACCTCAAGTCAGTTGCAAAGTGATGCACACGTTTGGGGAGATGTGTTACAACTTGACCCTTTGAGACTAACCATTTGCTTGAGGTTTGCTTGATTTAGTCTCGAAGGAGGATTGAAAGGAAAAGGTGGACTTGGACCATGAAGACTTCCTTGCACTCAGATGAGAGGTAACTAATTCCAAGTTCATCTCATGAAATCTTATTGCCATTTGCTCTTAATTGAAGACTTTGGTGAGGCAATGGGGTTAAAAGGCCAAGATTAATCCCGTTTGGTGCTTGATGCCAAGGGGAGAAAATAAGGCCAAAGTGATAAATGGATCACTACCACTTGAGAGATTTTGAAAATAGTAGAATAGAGTTTTGTTTTGTCAAAGCTTTTATTGTCTCTTATTGTCTCTATTGTCAAAAGTTGGCTTCTTGTGGGAGAAGTGTTGATTATGGGAAATAGGGGAGTTTTGAAATCTTTGATCAATCTCTTTTGGAATGACTCTCTTTATACTTCATCATGTGTGTTTGACTTAGAGATAGAGATTTGAGTTTGATTTGCAAAACAAACCAAGTGGTGGCAAAGGATGATCCATATATAAAATTGAATCAAAACCAATTTGAGTTTTTATTTGAAGTGATTTTGCACTTGTGCTATCTACTTTATGTTGTGTTGGCATAAATCACCAAGGGAGATTGAAAGGAAATGTGCCTTTGGGCCATTTCTAAGTATTTTAGTGATTGAGTGCAAACACAAGTGCTTAAATGTGAAAATATGCCCAAGGATGAACAAAGTGCAAATCACAAGTTAAGGTATGTTTCTAAGCCTTAGTACATTGGTTTTGTGTACTAATATATTTGTCTAAGTGTTAAACAGATAGAAGAAGAAAGAAGACTTGGTGTGTCGGACGAATTTCTCGATGCAGGGCGCGGTGTCGGTGGTGCCGAACGATGTCGGTCTTGATTCTCGGCCAGAAAAGAGGCGCTCTCGGTTTTCTCCGCGATTTAAAATTCACCGGATGTCCGGTGTGCCGGCTGTCCGGTGAGCCACGTCAGCCAAGACTAGCGGTCGGCCGCGCGTCGGCGCGCGACACGTGGCGAGCCAGCGGTCGGAAAAGCCGGGTGTCCGGTGTGCCGGGCTTGTCGGTGCGCCCGGTCTGCGATCGACAGCGGTCAGCCGCCATTTAAGGAAACCGTCGGACCGAGGGCGGTGTCCGTGTGCCGGTTGTCGGTCGCGCCCACGAGCGAAGGCAAAGATGGCCTTCAGTTGTTCTAGCGGCTCTAGCTGCCTTGGGGCTATAAAGGGACCCTAGAGCATGGAGAGTACACCAAGCATTCCTACAACTCTTCTAAGCACCAAGACATCAATCTCATTCGTTTCATTGTGATAGCATATAGAGCTCTTGTGGAGTTGTGAACTCTTTGTGTTGCGTTGCGAGCTCTTGTTGCGACATGTGTGCGTGTTGTTGCTCTGATTTTGAGTCTTGTGTGCGTTGCTCATTCCCACCTTACTCAGTATTTCTTTGTGAACTCAATTGTAAGGCGAGAGACTCCAAGTTGTGGAGATTCTCATAAGAGGAAAAAGATCAAAGGAAAGAAAAACACCGTGGTATTCAAGTTGATCATTGGATCACTTGAGAGGAGTTGAGTGCAACTCTCGTCAGTTGGGGCCACAACGTGGAGTAGGCAAGTTTTGTACTTGGCGAACCACGGGATAACCACCGTGTCAACTCTGTGATTGCTTTCTTGTGGTTGTGTGTTTTGAGTTCTCTCTAGCCACTTGGCCATACTTGTACTAACCTTAACAAGTTTTGTGGCTTAAGTTTAAGTTTTTCTGAGATCACCTATTCACCCCCTCTAGGTGCTCTCGGGACCACGAAGCGATGGATGATGTTGGTGGCGCCACCGCTTGTTCGGACGATCTTTGTTTAGAGTCGGACCTCGATCCACTTGGAGAATTTATCGATGGTGACCAACAGTCGTGGCCGGGTGCCTTACGCAGGGTAGCGAGATCGGACCCACGACAAAAGGCGGTGATGGGTATCGTCTGCGGGGCTGAAGCGGTAGTGGAGTTTGCGTCGCCCGCACCCTTCGCGAGTGCGGACAATTCTAGTGGCGTCGGCCACCGCGCGTTGGCGATGAAACCTTGCCGGGCGTTTCCAACAAGGGCTGAGGTCTTGCGTGATGGGCGCAAGCCCCGGTGTATCTCTTGTGGAGCTCGGCCTTGTGGAAATGCATCATTGGAGGATGCTGAGGGCTACGGTGGTAGAACTCTTTCGAGTCCCCACCAAGACAAGCGACTTGGCATGCGCGCCAACGCGAGCTTCGGCTCGGTCGGGTAGCTCTCCTCGGTGGAGATATTGCAGTGCAAGTGCCCCGTTCGATTAAGTGACCCAGCCGCTCCTCCTCGGCGTCGTGCCTTATCTCGGGGGCGAGGTACCTCGGGCGGTGAGGTGCCTGCGGGCGAGCAGTGCCTCGGGGTGAGCGGGACCGGTGCGGGCCGAGGTGCCTCGGCTTCCGGACGTGTCGTCGATCTTGGCGGAGGGTTGATGCAGGTCTCGGGAGAAGGCGTCGGAGAACCGTTGTTCGCCGAGGCTATTTTGTGGTGTAAACAAATTGGCGCTTAGACAACTTAATAACATTGCGGACGTTTTTAATGTACTGAATTAGCCTGAATTAATTCGGGATCTGGATTTTAGTACTGGATTTTGGTTTTAGGAATTAGAAATTTTATTGATAGAAGTATTTTACAAATACAAATACATACTAAGGGTTTCTTATATGCTCAACACATGAAAGAAACCTATAGAACCTAATTCCCTATCGGGAACTACTCACACATTATTATGGAGAAACTCGAGCTCTATTAGGCGGTGGCCTCAGCGTACTCGGCGGCGCCGAGGGCCGAGGTGAGCGAAGAACGAGAGGTAGGACTTGCGGATGTGGTGCGCCGGTGATGCGGAGTGGGCGTCTGCGTGAGGCCGAGGAGGGTGAGAGCATCATGCGGTGGTCGCCGTGGCGTCGTGGTGATGCCACCGAGGTGAGCAGCCGATCCGCGAGGAGAGTCGGCGGTCTCGCGTGCGGAGGCCGAGGCGCTCAAGCTCAGTGGCGGTGTCAGGAGATGCGGTCGCCTCCTTGAGGCGGAGAGTGGCCACGTTCTCCCAGGTGGTGTCGCCCTCGCCGAAGGCAGCAGCGGTGAGGGCTTGCACGGCGTCGGTGAAGGAATCGCCGTCGCGAGTCCGCAGCGTGGAGAGGCGGCCACGCGCACGGTGAGGTATCGCCCTCGCGCCGCGATGTCAGCGCCCATCTCGCGGAGCACGTTCGGCCTCCTTCTCGCCCTGGAGGTCGTGCTCGCGGAGGTTAGAGCGCACCTCGCGCTGGGAGGAGGGCAGCGGCGGTGAGGATAGCAGGCGGGTAGTCGCCGGCACGAGCACGCGGCCTGGGCGGTACCTGGCCACCCGGGATGGAGATGCGGCGGAGGTCGTCGGAGGCAGTGGCGCGCACGCCGAAGTCAGAGAGGGTGTCGAGTGTCTGGCGGAGCGGAGCGTGGGACTTGATGTCGCGGTGAGGCGGAGTTCGAGTCCGTCCGGGAGGAGAGGCCGAGAACATGAGGCGGAGGCGTACTGGGAGGAGCGCTCGGCGGACACCTCCACGGTGCCACCGCGCTGGGCCGGACACGGAGATCGGGAGGCGACCGTCGTTGGAGGACACCCAGGCACCGAGGCGTTCGAGGGCCTCAAGGAGGTCGCCCTGAGGGCGCTTGCCGAGGAGTCCGGGTAGTCGGTCACGAAAGTGGTGCCAGAGGTGAGCGGCCACGCCCATGAGGAGGCGGGCCACCGCGGCAGCGTTGCCTGGGTTGAGGTCACACCGGCCTGTGGGCGAGCGCCGAAACCGCGGATCACGGCGTCATCGCCCACAAGCTCCACGCCAGCGCCCCAGTCGCGGAGGCAGCGGAGCATGGCCTCGGCGTCCTCAAGTAGCCACGCCCACCACGCGGGTCTCGCCCTCAGCGAGGGCAGCGGCGAGGAGGTGGCGAGTGGTGTAGTTCTTGGATGGCTGAGCGCGAAGCTCGCCGCGGAGTTCGCAGCTGGATGCACGATCACGTCGAAGGTGGCGGGCAGGGCGTCGGATCCTTGCGGGGATCGGTGGGCCCCACGGCCGGAGCAGGGTGGCCGGGAGCCATCGGCATGGCGGGTGACGCCGCTGAGCACCTGATGGGCGCGGCGAGGCGCGGCGGTGTGTGGGGTGCGCCCGGCGCCTCGCCTTGGGCGCAGCGGTAGTGGCGCCAGTGAGCGCGGTAGACGCGGCGGCGGCGGTGGCCATGGTAGATCCTCTAGAGTCGACCTGCAAGTAACACAAACAACAGGGTGAGCATCGACAAAAGAAACAGTACCAAGCAAATAAATAGCGTATGAAGGCAGGGCTAAAAAATCCACATATAGCTGCTGCATATGCCATCATCCAAGTATATCAAGATCAAAATAATTATAAAACATACTTGTTTATTATAATAGATAGGTACTCAAGGTTAGAGCATATGAATAGATGCTGCATATGCCATCATGTATATGCATCAGTAAAACCCACATCAACATGTATACCTATCCTAGATCGATATTTCCATCCATCTTAAACTCGTAACTATGAAGATGTATGACACACACATACAGTTCCAAAATTAATAAATACACCAGGTAGTTTGAAACAGTATTCTCGATCTAGAACGAATGAACGACCGCCCAACCACACCACATCATCACAACCAAGCGAACAAAAGCATCTCTGTATATGCATCAGTAAAACCCGCATCAACATGTATACCTATCCTAGATCGATATTTCCATCCATCATCTTCAATTCGTAACTATGAATATGTATGGCACACACATACAGATCCAAAATTAATAAATCCACCAGGTAGTTTGAAACAGAATTCTACTCCGATCTAGAACGACCGCCCACCAGACCACATCATCACAACCAAGACAAAAAGCATGAAAAGATGACCCGACAAACAAATGCACGGCATATATTGAAATAAAAAGGAAAGGGCAAACCAAACCCTATGCAACGAAACAAAAAAATCATGAAATCGATCCCGTCTGCGGAACGGCTAGAGCCATCCCAGGATTCCCAAAGAGAAACACTGGCAAGTTAGCAATCAGAACATGTCTGACGTACAGGTCGCATCCGTGTACGAACGCTAGCAGCACGGATCTAACACAAACACGGATCTAACACAAACATGAACAGAAGTAGAACTACCGGGCCTAACCATGGACCGGAACGCCGATCTAGAGAGGTAGAGAGGGGGGAGGACGAGCGGCGTACCTTGAAGCGGAGGTGCCGACGGGTGGATTTGGGGAGATCTGGTTGTGTGTGTGTGCGCTCGAACAACACGAGGTTGGGGAAAGAGGTGTGGAGGGGTGTCTATTTATTACGGCGGGCGAGGAAGGGAAAGCGAAGGCGGTGAAGGAATCCCCGTAGCTGCCGGTGCCGTGAGAGGAGGAGGCCGCCTGCCGTGCCGGCTCACGTCTGCCGCTCCGCCACGCAATTTCTGGATGCCGACAGCGGAGCAAGTCCAACAGTGGAGCGGAACTCTCGAGAGGTCAGAGGCAGCGACAGAGATGCCGTGCCGTCTGCTTCGCTTGGCCCGACGCGACGCTGCTGGTTCGCTGGTTGGTGTCCGTTAGACTCGTCGACGGCGTTTAACAGGCTGGCATTATCTACTCGAAACAAGAAAAATGTTTCCTTAGTTTTTAATTTCTTAAAGGGTATTTGTTTAATTTTTAGTCACTTTATTTTATTCTATTTTATATCTAAATTATTAAATAAAAAACTAAAATAGAGTTTTAGTTTTCTTAATTTAGAGGCTAAAATAGAATAAAATAGATGTACTAAAAATTAGTCTATAAAACCATTAACCCTAAACCCTAAATGGATGTACTAATAAAATGGATGAAGTATTATATAGGTGAAGCTATTTGCAAAAAAAAGGAGAACACATGCACACTAAAAAGATAAAACTGTAGAGTCCTGTTGTCAAAATACTCAATTGTCCTTTAGACCATGTCTAACTGTTCATTTATATGATTCTCTAAAACACTGATATTATTGTAGTACTATAGATTATATTATTCGTAGAAGTAAAGTTTAAATATATGTATAAAGATAGATAAACTGCACTTCAAACAAGTGTGACAAAAAAATATGTGGTAATTTTATAACTTAGACATGCAATGCTCATTATCTCTAGAGGGGCACGACCGGGTCACGCTGCACTGTAGGCATGCGTCGAGAGATAGATTTGTGAGAGAGACTGGTGATTTCAGCGTGTCCTCTCCAAATGAAATGAACTTCATATAGAGGAAGGGTCTTGCGAAGGATAGTGGGATTGTGCGTCATCCCTTACGTCAGTGGAGATATCACATCAATCCACTTGCTTTGAAGACGTGGTTGGAACGTCTTCTTTCCACGATGCTCCTCGTGGGTGGGGGTCCATCTTTGGGACCACTGTCGGCAGAGGCATCTTGAACGATAGCCTTTCCTTTATCGCAATGATGGCATTTGTAGGTGCCACCTTCCTTTTCTGTCCTTTTGATGAAGTGACAGATAGCTGGGCAATGGAATCGAGGAGGTTTCCCGATATTACCCTTTGTTGAAAAGTCTCAATAGCCCTTTGGTCTTCTGAGACTGTATCTTTGATATTCTTGAGTAGACGAGTGTCGTGCTCCACCATGTTCACATCAATCCACTTGCTTTGAAGACGTGGTTGGAACGTCTTCTTTTTCTGATGCTCCTCGTGGGTGGGGTCCATCTTTGGGACCACTGTCGGCAGAGGCATCTTGAACGATAGCCTTTCCTTTATCGCAATGATGGCATTTGTAGGTGCCACCTTCCTTTTCTACTGTCCTTTTGATGAAGTGACAGATAGCTGGGCAATGGAATCCGAGGAGGTTTCCCGATATTACCCTTTGTTGAAAAGTCTCAATAGCCCTTTGGTCTTCTGAGACTGTATCTTTGATATTCTTGGAGTAGACGAGTGTCGTGCTCCACCATGTTGGCAAGCTGCTCTAGCCAATACGCAAACCGCCTCTCCCGCGCGTTGGCCGATTCATTAATGCAGCTGGCACGACAGGTTTCCCGACTGGAAAGCGGGCAGTGAGCGCAACGCAATTAATGTGAGTTAGCTCACTCATTAGGCACCCAGGCTTTACACTTTATGCTTCCGGCTCGTATGTTGTGTGGAATTGTGAGCGGATAACAATTTCACACAGAAACAGCTATGACATGATTACAATTCGAGCTCAGTACCCTGGATTTTGGTTTTAGGAATTAGAAATTTTATTGATAGAAGTATTTTACAAATACAAATACATACTAAGTTGTACAAAACCAGCAACTCACTGCACTGCACTTCACTTCACTTCACTGTATGAATAAAGAGTCTGGTGTCTGGTTCCTGATCGATGACTGACTTCTCCACTTTGTGCAGAACAGATCTAGAGCTCTTAGTAGAGCGGGGAGATGTTGGTCGGCACGAGCATGATGTTCATGAGGTCGAACTGTGTGCCAGAGTTGAGGTCACGTTGATGTCGAGCGGCACGTCAGCGGAGGAGGCCACCACGTTGCCGATGTTGATGTCGGAGAAGCGAGCACCGTTGTCGTTCACGCCGTCGTTGTTTGTGGTGGTGTTCACGTTTGTGGCGGTGTACACCGGCCGTTGATGGTCACGCGGATGGTGGAGTTGCCGATGGAAGACACGCGGAGGTAGAGGTTGTAGGAGTTGCCGTTACCGCGGAGGGTGTAACGAGCGGTGGTGTTGTTCTGCTCGAAGCGGAGAGAGTCGCCCTGGTTGCCGAACTTCTCGGAGATGAAGGTGCGGGTCTGGTTGTTCACCTGGGTAGCGTGGATCGGGAGATGGTGAAGCCGGTGTAGTCGTTTGGGCGAGGTGGGTCATGGAGCCGTTCTCGTGCACAGCGTGGATGTTCTTGCGGTTGTGCACGGACACCATGTAGCGCGATGCACCTCAGGGTGCCGGACGGGAGGCGATGTTGCGGATCTCGTTGTAGTGGAGTGGGCGACGGAGGTCCTCGTTGCGCACCACGAGCGGCACGCCGGAGATGTTGCGGATGAAGTAGTCCGGGAAGTAGTTGGAGTTGCCGCGTGCGGTGAAGGCACCAGGCAGGCCGAGGTGGTCTCGAAGGACTCGGTCTGCCAGTTGGTCACGGTAGCCACGCCTCGCGGTCGGAGCCGGAGTCGAGCCAGGAGCGCACGAACGGTGTGAGGCGGAGGGAGGAAGGTAGAGCAGTTGAAGTTCTGGTTGAACGGGAGCGCCGATGTCGCCGGAAGAGATGCCACCGGGTAGTTCACGCGAGCGGCGAGGAGGGCGTGGGTAGTTGTGGAGCCAGGGGCCCACGATGTTCGGGAAGGTGTTGGAGAGGCGGGCGCCGGAGAAGCCGTTGAGCACGTAGTTGGAGTTCACCTGGAAGAGGAGTAGAGGAACAGCCAGTCCTGGGAGGTGAAGGACTGGGTCTGCTGCGGGCCGGAGCCGGAGGCGTAGAGGTTGGCGCCGGAGGACACGAGGAGGGACTGGTACTTGAAGAGGGACCAGATGGACACGTACTCGAACACGTTGAGGAACATGTAGGTGCGGAACTCAAGCATCTCGTGGAGAGGGTGTTGAGCTTCTTGAAGGCTGTCTGGTAGGTGTTGATGCAGTAGTTGGAGTACTCCTTGGTGTAGTCCTTGAGGTAGCCACGGTAGGTGCGAGAGTGGCAGCGGTACCCACTCCTCTGCGTTGAGGATCACATCGCGGATGAAGGAGAGGTGGAGGTTGGCGGCCTGAGCGAAGAGTGGAAGGAGAAGGAGTTCGTGCCGCGGAGCTGGAACTGGGTGAGGCGGTTGAGGAAGAGCTGCTGCATGGTGTTCACCGGGGATGTGATGGAAAGGAGTCGGGTTACGGTTCGGGTTGAGGAAGTTGTCCACCTGACGGTTGAACTCGGCAACGTTCTTCTGGAGGCCCTCAAGTTCGGCGTTCACGCGGGAGAGGGTGTCAGCGTTGAGGCGCTGGTTGAGGAACTTCTCGGTCTCACGGAGGATGTCCTGCATGAGCTTGGTGTTGTCGTTTGGGAACACGAGGTCCCAGAGGCCATTGAGGATGCGCTTGGCAGCAAGCCTCGAGCTTCTTGAGGAGGAAGATGGCCACAGTACCCACAGGAGCCACGTAGAGTGAGTGGTCGTCGCGCTTCCAGGTCCACTCCTTCTGGATGGTGTCGAGGACTTGTACTGGAAGAGAACGGGTCCTGGGCCATCACGTTGTGGCCGTCGCAGATGGTGGTGCGTCCGGAGTTGAGCACGGAGCTCCTCCACCCTTCCGGGGCCTCGCACCTGATCGATGTGGTAGTCGGTCACGTCGGTCTTCAGGCCGATCTGGTTGCTGCTGGTGAACAGCTCGTTCACGGCCTTCTGAGCCCTCTCCAGGTCGTACTCGGCCTCGAAGGTCACCTCGGCGGGCACGAACTCGATGCGGTCGATGTACCCTCGTTGCCGCTGTTGAACACGTGGGCGCTCAGGTGAACACGCTGCTGCCGTTGCTGAAGTTGAAGGGGTGGTGAAGCCCACGGTGCGGAAGCTGCCGCTCTGCAGGTTGCTGCCGCTGCTCATGGTGGCGCTGAAGTTGCCCTGGTTGATGGGGCGGCCGTCGATGCTGGTGTGGAACTGCAGGTTGGTGGTGCTGGCGTAGCGGATGCGGACGCGGTAGCGCTGGCTCAGGGGGCGGTGATGTTCACGCGCAGGGTGCTGATCTGGCCGGGGCTGGTGCGGCGCAGGATGTCGCCGCCGGTGAAGCCGGGGCCCTTCACCACGCTGGTGCCGCTGCCCAGGTTGGTGCTCTTGGTCAGGGGATCTGGGTGATCTGGCTGCTGGGGATGATGTTGTTGAACTCGGCACTGCGGTGAATCCAGCTGAACATAGGTGCACGGATGATGCTCACGCTGCTGTTGCTGAAGCCACTGCGGAACATGCTCACGTGGCTCAGACGGTGGCTGAAGCCCTGTCGAGGTGGCGTTGTTGTTCTGAGGGGATCTCGTCCAGGCTGTCCACGGTGCCGCTCTTGCGGTACACGGCGCTGGGCAGGTTGCTGCTGGTGCCGTAGGCGAACTCGGTGCCGTCCAGCACGCTCAGCTGCTGGTTGTTGATGCCGATGTTGAAAGGTCGACGGTACAGGGTGCTGCTCAGGGTGCGGTACACTCCTGGCCCAGCTGTGCCACGATGCGCTGCTGAGGTGCAGCGTTGCCCATGGTGCCGTACAGGGAAGGTGAACTCGGGGCCGCTGAAGCCGACGGGGCTGGCCATGATCTGCGCCGCTCCAGTAGTACTCGCCGCGGTGGGCGTCGGTGTAGATGGTGATGCTGTTCAGGATGTCCATCAGGTGGGGCTGCGGATGCTGCCCTCGATGCCCTGGGCGCTGCCGCGGAAGCTGCCGTCGAAGTTCTCCAGCACGGGGTTGGTGTAAATCTCGCGGGTCAGCTGGCTCACGGTGCGGATGGGGTAGGTGCGGCTGTCGTAGTTGGGGAACAGGCTCACGATGTCCAGCACGGTCAGGGTCAGCTCGCGGCGGAACTGGTTGTACCTGATCCAGTCGCGGCTGTCGGGACCCCACACGCGCTCCAGGCCGGTGTTGTACCAGCGCACGGCGTGGTCGGTGTAGTGCCGATCAGGCGGGTCAGGTCGTTGTAGCGGCTGTTGATGGTGGCGGCGTCGAAGCCCCAGCGCTGGCCGAACACGCTGACGTCGCGCAGCACGCTCAGGTGCAGGTTGGCGGCCTGCACGTACACGCTCAGCAGGGGCACCTGGTAGTTCTGCACGGCGAACAGGGGATGGCGGTGGTCAGGGCGCTGTTCATGTCGTTGAACTGGATGCGCATCTCCTCGCGCAGGGCGGGGTTGGTGGGGTCGGCCTCCACTCGCGGGCTCTCGGCGTAGATTTGGTACAGGTTGCTCAGGCCCTCCAGGCGGCTGATGGCCTGGTTGCGGGCGAACTCCTCGATGCGCTGGTTGATCAGCTGCTCGATCTGCACCAGGAAGGCGTCCCACTGGCTGGGGCCGAAGATGCCCCAGATGATGTCCACCAGGCCCAGCACGAAGCCGGCGCCGGGCACGAACTCGCTCAGCAGGAACTGGGTCAGGCTCAGGCTGATGTCGATGGGGTGTAGCCGGTCTCGATGCGCTCGCCGCCCAGCACCTCCACCTCGGGGTTGCTCAGGCAGTTGTAGGATGCACTCGTTGATGTTGGGGTTGTTGTCCATTGTTGGATCCTCTAGAGTCGACCTGCAGAAGTAACACCAAACAACAGGGTGAGCATCGACAAAAGAAACAGTACCAAGCAAATAAATAGCGTATGAAGGCAGGGCTAAAAAATCCACATATAGCTGCTGCATGTGCCATCATCCAAGTATATCAAGATCAAAATAATTATAAAACATACTTGTTTATTATAATAGATAGGTACTCAAGGTTAGAGCATATGAATAGATGCTGCATATGCCATCATGTATATGCATCAGTAAAACCCACATCAACATGTATACCTATCCTAGATCGATATTTCCATCCATCTTAAACTCGTAACTATGAAGATGTGACACACACATACAGTTCCAAAATTAATAAATACACCAGGTAGTTTGAAACAGTATTCTACTCCGATCTAGAACGAATGAACGACCGCCCAACCACACCATCATCACAACCAAGCGAACAAAAGCATCTCTGTATATGCATCAGTAAAACCCGCATCAACATGTATACCTATCCTAGATCGATATTTCCATCCATCATCTTCAATTCGTAACTATGAATATGTATGGCACACACATACAGATCCAAAATTAATAAATCCACCAGGTAGTTTGAAACAGAATTCTACTCCGATCTAGAACGACCGCCCAACCAGACCACATCATCACAACCAAGACAAAAAAGCATGAAAATGACCCGACAAACAAGTGCACGGCATATATTGAAATAAAGGAAAAGGGCAAACCAAACCTATGCAACGAAACAAAAAAATCATGAAATCGATCCCGTCTGCGGAACAGCTAGAGCCATCCCAGGATTCCCCAAAGAGAAACACTGGCAAGTTAGCAATCAGAACGTGTCTGACGTACAGGTCGCATCCGTGTACGAACGCTAGCAGCACGGATCTAACACAAACACGGATCTAACACAAACATGAACAGAAGTAGAACTACCGGGCCTAACCATGGACCGGAACGCCGATCTAGAGAAGGTAGAGAGGGGAGGACGAGCGGCGTACCTTGAAGCGGAGGTGCCGACGGGTGGATTTGGGGAGATCTGGTTGTGTGTGTGTGCTCGAACAACACGAGGTTGGGGAAAGAGGGTGTGGAGGGGTGTCTATTTATTACGGCGGGCGAGGAAGGGAAAGCGAAGGAGCGGTGGGAAAGGAATCCCCGTAGCTGCCGGTGCCGTGAGGAGGAGGAGGCCGCCTGCCGTGCCGGCTCACGTCTGCCGCTCCGCCACGCAATTTCTGGATGCCGACAGCGGAGCAAGTCCAACGGTGGAGCGGAACTCTCGAGAGGGGTCCAGAGGCAGCGACAGAGATGCCGTGCCGTCTGCTTCGCTTGGCCCGACGCGACGCTGCTGGTTCGCTGGTTGGTGTCCGTTAGACTCGTCGACGGCGTTTAACAGGCTGGCATTATCTACTCGAAACAAGAAAAATGTTTCCTTAGTTTTTAATTTCTTAAAGGGTATTTGTTTAATTTTTAGTCACTTTATTTTATTCTATTTTATATCTAAATTATTAAATAAAAAACTAAAATAGAGTTTTAGTTTTCTTAATTTAGAGGCTAAAATAGAATAAAATAGATGTACTAAAAAATTAGTCTATAAAACCATTAACCCTAAACCCTAAATGGATGTACTAATAAAATGGATGAAGTATTATATAGGTGAAGCTATTTGCAAAAAAAAAGAGAACACATGCACACTAAAAGATAAAACTGTAGAGTCCTGTTGTCAAAATACTCAATTGTCCTTTAGACCATGTCTAACTGTTCATTTATATGATTCTCTAAAACACTGATATTATTGTAGTACTATAGATTATATTATTCGTAGAGTAAAGTTTAAATATATGTATAAAGATAGATAAACTGCACTTCAAACAAGTGTGACAAAAAAATATGTGGTAATTTTTATAACTTAGACATGCAATGCTCATTATCTCTAGAGAGGGGCGACCGGGTCACGCTGCACTGCAGGCATGCAAGCGGCACTGGCCGTCGTTTTACAACGTCGTGACTGGGAAAACCTGGCGTTACCCAACAATCGCCTTGCAGCACATCCCCTTTCGCCAGCTGGCGTAATAGCGAAGAGGCCCGCACCGATCGCCCTTCCCAACAGTTGCGCAGCCTGAATGGCGAATGCTAGAGCAGCTTGAGCTTGATCAGATTGTCGTTTCCCGCCTTCAGTTTAAACTATCGGTCGTTGTAGCGTCGAGGCGTGTGGTTGAGCTCGAGCCGTAGAACTTGTCTTCCGAGCGCCGACGTCGTCGCGATGAGCCTCCATCTTGGGTCGCGGCGGTGGGAGTTCTTCATGACTTGGTCGATGGCAGTGCGAGTCACCCGAGCGTCGGCGTCGGACCCCGGCTCGATGGCGATCGCGGCAGATTGACGAGCCTGTGCCCGACTCCACATTCGGCGGCGGGAAGTGGAGCGTAGCACATAGCGTGGAGTGCTTCAGGCGAGATGAAGAAGCGAGCCTGCAGGCTCCTCTTCATCGACAGTCGAAGAACATGGTCGAGTTCGGTTGGATCGGGCCGTTGGGCCGGTGTCGACCCATTCGACCACGAAGTCCGCGAACACAGGACTTGATGGCCTTCAGAAGCGAGCGAGATCGCCGCCCATGATTTCCGCCCACTTTGCGATTCTACAGGGCCTCGGCACTAGATGATCTCCCCAGAAGGATGACACCGATTCACCGGATGAGACTCGAAGTAGTGTCGCAACCGCCGCGTCGGGATCACCGCGTCAACCGAATTTGTGGGTAGCGGATCTTGGTCTCGAGACGATTACCTCGTGAAGTAGACTTGCCTCACGGGCGGGCAATGCATGCCCCTCTTCTCTCTCTCAACCACGATCGCGGCGCTAAGCACACGAGTGGTCGCGGCGTAGATCAATAAGGCTTCTCCGTGGCAGGGGCACCAAGATAGGCGCGTTCGTGAGGCGCCTTGAGTTCAGAGGCTTCTCGGCCTCGAGTCCAAGTAAACACTCGGCCTTCCTTAAGAGGCGGTATAAGCGAGCCTCTTCGGCGAGGCGTGAGAATGAAGCGGCTCGAGCGCAAGACATCCGTGACTCTCATGCGCCCTTCAAGTCCTTGATGGGCCCCCTTTGATGATGGCGATCTTCTCGGATTGGCCTCGATGCCCACTCGGAGACAATGAACCCCAAGAGCATGCCTCGAGGAACCCCAAAGACACACTTCTCGGATTAAGCTTCCGTCTTTCGCCTTGAGACATCGGAATGTCACTTCAAGGTCGGAAAGGAGGTCTGAGGCTTTCCTCGTCTTGACTCGATGTCATCGGCGTAGGCCTCGACCGTCCGACCAAATGTGTTCGCCGAACACATGGTTCATCTGCCATTGGTACGTCGCACCCGCATTCCTCAGCCCGAACGGTATGGTGACATAGCGATACATGCAGAAGGGTGTGATGAAAGAAGTCATGGTGTCGGATTCTTTCATCTGATTTGAGTGGTACACAGTAGGCATCGAGGAAAGACAAGGTTCGCACCCGGTGGAATCCACAATTTGATCGATCGAGGCGGAGGGTAGGGAACCGGACATGCTTTGTTTAGACAGTGTAGTCTACACACATCCGCCTTTCCTCCTTTCTTTCTCAAAAGCGGGGTTGGCAAGCCATTCGGGATGGAATACCTCTTTGATGAACCTGCTGCCATTAGCTTGTGGATCTCTCGCCTATGGCTCTGCGCTTTTCTTCATCGAATCATTGCGGCGCTTCATAGTCGGGCTCCAACTCGGATATCGGTGAGTGCTCGGCGACATCCCTCGGTATCACGGGCATGTCAGAGGACTCCGCAGCGTCGGCGTTTGCGCGGAGAAAGTCGGCGAACCGCACTTCCCTATTGGGATCGAGCTCGGGCCGATCCGGATCGCTGGGCGTCGGCTGGCTGGGGTCGAGGGGCGGACTTAACCGTCTCGTGGCTCGAGTTGTCGGCGTGGCGCTTCACGTCGGCACCTCCTTAGAGGCTTTCCGGGTCGCGATGAGGGCCTTGGATGGCGAGGGCCTCGGCGCCTCCACGCACCTCCGTCGCGTCCGAGCAGCGTGTTTGTGCGTGGGGCGGCGGTGATGACCCGTTGGGCGGCATCTTGAGCTTGAGGTAGGTGTAGTTGGGGGCGGCCATGAACCCGTAGCATGGCCTCCCGATCTTTGCGTGGTAGGTTCTCGAACCCGACCACCTCGACGTGAGGGTCTCCCTTGAAGTTGGAGGGTGTTCGGCGGGCGGAAGGTCGAGTTGTCCAGAAATTTGGGCCAGCTTCGGGATGATCCGTGGAATGGCGCGGCGCTCCGGACGAGGATAGATCAACACGCGAGGCAGAGGTCTCGGCATAGATGATGTTGAGGTGCGCCTCAGTCATGAGGACCTTGGTGAGCTGGCGTCACGATGGCAGGGTCGACAGCGGCGAGTATTTCCCGGGCTCGACGCGTGGTCGGGGTGGTCAGCTGGTCGAAGGTGATGGGCTTGTCGGACCAGTCTAGGTAGTGAAAGTAGCCAGGGGGTGAATAGGCTAATCTGAAAATTTTCACAACAAACTTGAAAGATTATTATATACAGATTCATTGGTGCAAGCAGTTCAGCAGCTACGTCATAAGTTGAACCACTCGAACCAATCAAGCTGTTTATAAACTTTAGACTAAAATCTACTCGAGAAAGTATTTATAAGTTCTTGAGAGAAGTAAGATATAGCTAAGTGAGGATGAAAAGATGAATATGTAAAGGCTATTTTACTTCTAGATAAACTCTACTGGAGAAATCTTTATGTCAATCTTGCAAGTAAAAATATCTCAAGTTGAAACAAGTAAGAACACAAGACACAAGATTTAATCAGAGGTTGGCCACACCACAAGGTGTCCTACTTGAGAGCCCACAAAGGGTAGGGTCTTTTTCAACCTAATCCTCCAAAGCCGACCACAAGGTCAAGGCAATCTCTTCTTATCTTAGCTCAAGAGCGGGTGATACCAACTTCTTAGGTCGTCCACAAATTTGGAGACTCCCAAGTAACCTCGAAGATCTTGAAACCTAGGGTTTCAAGAACACCAAAGCACAAGAGGGGTTTGCACAAGCTCAAGTCTTTGAAAAAGAGATGGGAGAGAAAACCAAATCGTGAGCACAAGCACAAACCTCACACCCAGAGCTCCTCCAACAAGGTTGAATCTTGAGGAAGATTTAAGTGTGAGAGAGATGGAGAGATGAGTGCTTTGTCTCAAGTTAGGTGAGCAATAAATGAGTGAGTGTTGGTGTCTTGAAGAAGAGAGAGGGTCTATTTATAGTCACGGCTCAAAAACTAGCCGTTGACCAAAACCCCGGCGAAAACAGTTGACAGCCCCTAAGGCGGTTGAACCATGGTCGTGACTGTCTGCCCGGTCACGGCTGGCCGGTGTAACGACCCGACTGCGTTAGTCAGACGAGCGGATCGGTTGAGCAGCCCCTAGAGCGGTTGAGCAGCCACGCCTACAGCACATTTGCCGGTCGGTGGCGGCTGCAGTCAGGTCGAAGGTCGAGACGGTTGAGCTACCCCGGGACGGTTGGTGGTCTAGGCCGAGTTTTGGAGAAAACCTAGCTCGGTACTGCAGGGCAAGTTCATTGGGTATGGTCGAGAGAAGGTCCACAATGAAGTTGAAGTTCATTTGGGTTGGTTCAACTTCAACTTGAAGTCCACCAATTTGAAAAGCTCATTGCGGTGAAGAAGCTCATCTGGCGAAGTTAAGTTCAGTGGAAAGCTCATTCTTGATTGAAGAAATTCAGTTTTTTAAGCAAGAACACTCTAGGTTTCTCAACCCAAACCATGGTCGACCAAATAATGTTAAAGAGATTTTTGGTTTTCAAAAATAGCTTTTGAATTAGAGGACTTGAGCTATAGCAAACACTGTGCACATGCGAGGGAAAAGAACAAGGAAGAATTACATCATGCAACACAAGATTTTACATAAATTTTATCGTCTGTTGCATGAAGTCCTTATGCTTCCTTAAGTTCCTGTTTCCTTCTAATCAAACACTGAGAACAAAAATTGTTAGTACTCTTATTTGTTTTGTCATTAAATCACCAAAACCCTCACTTGGGGTTGATTGCACTTACAATCTCCCCCTTTTTGGTGATTGATGCCAAAACAATTAAAGTCAAAAGATATATATTTGCAATAGAAAATTTCTTTTGAATGATTGTATGTAGATGGCTCCCTAAATGTGTACGATTGTGAATCCAACGTCTTGACATAATATGTCATGTAGCAACACATTTAGAGATAGTGACTTAACCATACTAAATACAATTATCCGAGGGTGCAAGAGTGTCATGACAGGAGTTGTGATGTACATGACTATCTCTAAAAACCATTATTTTTACTTCATAGCAGAGTAGACATTACAAACGATAGGACAAAAGATGATGGCCAAAGACCATCATATTACTTTGAATAACAATCCAACAGTTTTATTTCATACAATGGTAAAAGGGTACAAGCTGGCAAAAAGCAAAAACAAAAATAATCCAAAAGAAAAACAGAAAGAACCACAAACTAAGTTGGATTTTCTCCCCGCTTGGCAACAAGTACCAAAAAAACGGAGAGAGACAGGATACAAATCCAATATCCTCCAGGAGGAGGGGTGGATATGACGGAGGATAA

>nano9

CCATGTGTAAGCTCTCTAAAAAGCTCTGGACTTAAGCAAGCCCCAAGAGCATGGTATGAATGCCTTAGAGACTTTTTACTTGCTAATGCTTTCAAGGTTGGGAAAGGCGATCCAACTCTTTTACAAAAAGACATGTGATGGTGATTTGTTTGTGTGCCAAATTTATGTCGATGACATAATATTTGGTTCTACTAACAAAAGTCTTGTGAAGAGTTTAGCAGGGAGTGATGGCAAGTCGAGATGTCAATGATGGGCGAGTTGAACTACTTCCTTGGGTTCCAAGTGAAGCAACTCAGGGACGGCACCTTCATCTCCCAAGCAGTACAACAAGATCTGCTAAGCGGTTTGGGATGAAGGGCGTAAACGCAAAGACTCGGATGGGGGCCAGACGGACACATGAGCCTCAACAAAGGAGGTAAGTCAGATTGATCAAAAAAGCATACCGGTCAATGATAGGTTCTTTGCTTTACTTATGTGCTAATGAGCCGGATATTATGCCGCGTATGCATGCTGAGATTTCAATCCGATCCTAAGGAATGTCACTTGAGTAGCGGTGAAGCGAATTCTTAGATATTTAGTTGCTCATGTGCTTGGGCTCTGGTATCAAAGGGTCTACCTTTGACAGTTGGATACTCAGACTCGGCTATCTTTGGATGTAAGGTCGATAGGAAGAGCACATCGGGGCGTGCAATTCTTAGGAAGGTCCACTGGTGTCATGGAACTCTAAAACAAACTTCCGTTGCCCTATCCCACCCCGTGGTAGGTATGTTCTGCGGGGACCGGTGTTGCGCGCAACTACTTTGGATGAGGCAAACCCTCGAGGACTTTGGCTACAATCGAGCAAAGTCCCACTCTATGTGACAATGAGAGTGCTATCGCATGGCGGAAGAATCACATGAACCATGGCCGCACCAGCCTATAGACATCGGCATCACTTTTGAGAGACCACCGGCAAAGGGAGATATCGAAGTGTTTCATGTTACAGAGAACCCGACTAGCCGATATCTTCACTAAACCTCTAGATGAGAAGCCTTTTTGGGTTGCGTAGTGAGCTAAATGTCTTAGATTCCTGGAACACAGATTGAATTGTAGCATACATGTAGTTATGCTTTTGATCATGTTCCTTTTGCATTATGTTGCTTATTATGGTGCTCAAGTTGTACAAACACTCCTGGACCTCACATCCGTTGCAAAGTGATGCACACGTTTAGGGAGATGTGTTACAACTTGACCCTTTAGACTAACCATTTGCTTGAGTTTGCTTGATTTAGTCTCGAAGGAGGATTGAAAGGAAAAGGTGGACTTGGACCATGAAAGACTTCTTTACTCAGTGAGAGGTAACTAATTCCAAGTTCATCTCATGAAATCTTATTGCCATTTGCTCTTAATTGAAGACTTTGGTGAGGCAATAGAGGGTTAAAAGGCCAAGATTAATCCGTTTTGGTGCTTGATGCCAAGGGGAGAAAATAAAGCCAAAGTGATAAATGGATCAACTACCACTTGAGAGATTTTGAAAATAGTAGAATAGAGTTTTGTTTTGTCAAAAGCTTTGTGTCTCTTATTGTCTCTATTGTCAAAAGTTGGCTTCTTGTGGGGAGAAGTGTTGATTATGGGAAATAGGGGAGTTTTGAAATCTTTGATCAATCTCTTTTGGAATGACTCTCTTTATACTTCATCATGTGTGTTTGACTTAGAGATAGAGTTTGAGTTTGATTTGCAAAAAACAAACCAAGTGACAGGATGATCCATATATGCCAAAATTGAATCAAAAATTTGAGTTTTTATTTGAAGTGATTTTGCACTTGTGCTATCTACTTTATGTTGTGTTGGCATAAATCACCAAAGGAGAATTGAAAGAAATGTGCCTTTGGCCATTTCTAAGTATTTTAGTGATTGAGTGCAAACACAAGTGCTTAAATGTGAAAATATGCCCAAGGATGAACAAAGTGCAAATCACAAGTTAAGGTATGTTTCTAAGCCTTAGTACATTGGTTTTGTGTACTAATATATTTGTCTAAGTGTTAGAGCAGATAGAAGAAGAAGAAGACTTGGTGTGTCGGCAGGCTACTCCGATCAGAACGCGGATGTCGGTGGTGCCGGACTGATGTCGGTCTGCCTGACGCCTCGGCCAGAAAGCGCGCTCTCAATTTCTCGCGACTTGGCTAAATTCACCGGATCAGGTGTGCCGGCTGTCCGGTGAGCCAACGATCGGCCAAGACCAGCGGGTGTAGCGATCGGCCAGCGCGACACGTGGCCGAGCCAGCGGTCGGAAAAAGCCGGTTATCCGGTGTGCCGAATTGTCGGTGCGCCAGATCTGCGATCGGCGGTCAGCCGCCATTTAAGGACATCGAACCGCCGGGCGATGTCCAGTGTGCCGGTGTCGGTGCGCCGAAGACAGTAGGTGGCCTTCGGTTGTTTCATGCGCTCTAAAGCTGCCTTGGGGCTATAAAGGGACCCTAGGCATGGAGAGTACACCAAGCATTCCTACAACTCTTCTAAGCACCAAGACATCAATCTCGCATTCGTTTCATTGTGATAGCATATAGAGCTCTTGTGGAGTTGTGAACTCTTTGTGTTGCGTTGCGAGCTCTTGTTGCGACATGTGTAGATGTTGTTGCTCTGATTTTGAGTCTTGTGTGCGTTGCTCATTCCCACCTTACTCAGTATTTCTTTGTGAACTCAATTGTAAGGGCGAGAGACTCCAAGTTGTGGAGATTCTATATGAAGAGGAAAAAAGATCAAAGGAAAGAAAAACACCGTAGTATTCAAGTTGATCATTGGATCACTTGAGAGGAGTTGAGTCAACTCGTCAGTTGGGGCGCCTGGCGTGGAGTAGGCAAGTTTTGTACTTGTAGAACCAGGATAACCACCGTGTCAACTCTGTGATTGCTTTCTTGTGGTTGTGTGTTTTGAGTTCTCTCTAGCCACTTGGCCATACTTGTACTAACCCTTAACAAGTTTTGTGGCTTAAGTTTAAGTTTTCTACAGGATCACCTATTCACCCCCTCTAGGTGCTCTCGGGACCTGAAGCGATGGATGATGTTGGTGAGAACGCGCTTGTTCGGACGATCTTTGTTTTAGAGTCGGACCTCGATCCACTTGAGAATTTCGATGGTGACCAGCAGTGCGTGTAGCCGGGGTGCCTTATACGCGAAGCCGCGAGATCGGACCCACGGCAAAAGGCGGTGATGGGTATCGTCTGCGGGGCTGGCGGCGAGTGGAGTGGCCTGCGTAGAATCCCGACACCCTTCGCGAGTGCGGACAATTCAGTGGCGTCGGCCACTGGTGCCCGATGAAACGCAGCCGGGCGTTTCCAACAAGGGCTGAGGTCTGCGTGATGGGACCAAGCCCAGTGTATCTCTTGTAGAGACTCGGCCTTGGCGATGGAAATGCATCGCTGGAGGATGCTGAGGGCTACGGTGGTAGGCTCTTTCCGTCCCCACCAAGACAAAGCGACTTGGCATGCGCGCCAACGCGAGCTTCGGCTCGGTCGAGTGTAGCTCTCTCGGTGGAGATATGCGGTGCGAGATACTGCCCGGTTCGATTAAGCGTGACCAGCCCGCTCCTCCTCGGCGTCGCGTGCCTTATCCTCGAGGGCGGTACCTCGGGCGGTGAGTGCCTCGGGCGAGCAGTGCCTCGGATTTGAGCGAGGGTGCTCGGGCTGGCCGAGTGCCTCGGCTTCCGACGTGTCGTCGATCTTGGCGGAGGGTTGATGCTGGTCTCGGGAGAAGGCGTCGGAGGAACCGTTATTCGCCGAGGCTATTTTGTGGTGTAAACAAATTGGCGCTTAGACAACTTAATAACACATGCGGGCGTTTTAATGTACTGAATGCCTTGAATTAATTCGGGGATCTGGATTTAGTACTGGATTTTGGTTTTAGGAATTAGAAATTTTATTGATAGAAGTATTTTACAAATACAAATACATACTAAGGGTTTCTTATATGCTCAACACATGAGCGAAACCCTATAGAACCTAATTCCCTTATCAGGAACTACTCACACATTATTATGGAGAAACTCGAGCTCTATTAGGCGGTGGCCTCAGCGTACTCGAGCGGCGCGAGGGCCGAGGTGAGCGAAGAACGAGGGGAGTAGGACTTGCGGATGTGGTGTGCGCGGTGATCGGAGTGGGCGTCTGCGGGCCGAGGAGTGAGAGCATGATCATCACGGTGGTCGCCGTGGGCCGTCGTGGTGATGCCACCAGCGAGTGAGCAGAGACCGTCACGGAGAGAGTCGGCGGTCGGCGTGCGCGAGGCCGAGGCGCTCAAGCTCAGCAGTGTCGAGATGCGGTCGCTCCTTGAGGCGGAGAGCCACGTTCTCCCAGGTGGTGTCGCCCTCGGCGAAGGCAGCAGCGGCGGTGAGGGCTGCACGGCGTCGGTGAAGGAATCGCCGTCGCGAGTCCGCAGCGTGGAGAGGCGTACCGCGCACGGTGAGGTATCGCCCTCGCGCCGATGTCAGCGCCCATCTCGCGGAACACGTTCGGCCTCCTTCTCGCCCTGGAGGTCGTGCTCGCGAGGTTAGGCGCACCTCGCCTGGGAGGAGGGCAGCGGCGGTGAGGATGGCAGCGGGCCGGGTAGTCGCCCGGCACGAGCACGCGGCCTGGGCGGTACTTCTGCCACCCGGGATGGAGATCGGCGGAGGTCGTCGGAGGCAGTGGCGCGCACGCCGAAGTCAGAGAGGGTGTCGAGTGTCTGGCGGAGCGGAGCGTGGGACTTGATGTCGCCGGTGAGGCGGAGTTCGAGATCCGTCCGGGAGAGGGCCGAGGAACATGAGGCGGAGGCGTACTGGGAGGAGCGCTCGGCGGACACCTCCACGGTGCCACCGCGCACTGGGCCAGACACGGAGATCGGGAGGCGACCGTCGTTGGAGGACACCCAGGCACCGAGGCGTTCGAGGGCCTCAGGAGGTCGCCCTGAGGGCGCTTGCCGAGGAGTCCGGGTAGTCGGTCACGAAAGTGGTGCCAGAGGTGAGCGGCCACGCCCATGAGGAGGCGGGCCACCGCGGCAGCGTTGCCTGGAGTTGAGGTCACACCGGCCTGTGGGCGAGCGCCGAAACCGCGGATCACGGCGTCATCGCCCAAGCTCCACGCCAGCGCCCCAGTCGCGGAGGCAGCGGAGCATGGCCTCGGCGTCCTCAGAGTAGCCACGCCCACCACGCGGGTCTCGCCCTCAGCGAGGGCAGCGGCGAGGAGGCAGCGAGTGGTGTAGTTCTTGGATGGCTGAGCGCGAAGCTCGCCGCGGAGTTCGCGAGCTGGATGCACGATCACGTCGAAGGTGGCGGGCAGGGCGTCGGATCCTTGCGGGGATCGGTGGGGCCCGGCCGGGCAGGGTGGCCGGGGCCATCGGCATGGCGGGTGACGCCGCTGAGCACCTGATGGGCGCGGCGAGGGCGCGGCGGGTGGCCAGGAGGTGCGCCCGGCGCCTCGCCTTGGGCGCAGTAGTGGCGCCAGTGAGCGCGGTAGACGCGGCGGCGGCGGTGGCCATGGTAGATCCTCTAGAGTCGACCTGCAGAGTAACGCAAACAACAGGGTGAGCATCGACAAAAGAAACAGTACCAAGCAAATAAATAGCGTATGAAGGCAGGGCTAAAAAATCACATATAGCTGCTGCATAATGCCATCATCCAAGTATATCAAGATCAAAATAATTATAAAACATACTTGTTTATTATTATAATAGATAGGTACTCAAGGTTAGAGCATATGAATAGATGCTGCATATGCCATCATGTATATGCATCAGTAAAACCCACATCAACATGTATACCTATCCTAGATCGATATTTCCATCCATCTTAAACTCGTAACTATGAAGATGTATGACACACATACAGTTCCAAAATTAATAAATACACCAGGTAGTTTGAAACAGTATTCTACTCCGATCTAGAACGAATGAACGACCGCCCAACCACACCACATCATCACAACCAAGCGAACAAAAGCATCTCTGTATATGCATCAGTAAAACCCGCATCAACATGTATACCTATCCTAGATCGATATTTCCATCCATCATCTTCAATTCGTAACTATGAATATGTATGGCACACACATACAGATCCAAAATTAATAAATCCACCAGGTAGTTTGAAACAGAATTCTACTCCGATCTAGAACGACCGCCCAGCAGACCACATCATCACAACCAAGACAAAAAAAAGCATGAAAAGATGACCCGACAAACAAATTGCACGGCATATATTGAAATAAAGGAAAAGAACAAACCAAACCCTATGCAACGAAACAAAAATCATGAAAATCGATCCCGTCTGCGGAACGGCTAGAGCCATCCCAGGATTCCCCAAAGAGAAACACTGGCAAGTTAGCAATCAGAGTGTCTGACGTACAGGTCGCATCCGTGTACGAACGCTAGCAACACGGATCTAACACAAACACGGATCTAACACAAACATGAACAGAAGTAGAACTACCGGGCCTAACCATGGACCGGAACGCCGATCTAGAGAGGTAGAGAGGGGGAGGGACGAGCGGCGTACCTTGAAGCGGAGGTGCCGACGGGTGGATTTGGGGAGATCTGGTTGTGTGTGCGCTCGAACAACACGAGGTTGGGGAAAGAGGGTGTGGAGGTGGTGTCTATTTATTACGGCGGGCGAGGAAAGCGAAGGAGCGGTGAAAGAATCCCCGTAGCTGCCGGTGCCGTGAGGAGGAGGAGGCCGCCTGCCGTGCCGGCTCACGTCTGCCGCTCCGCCACGCAATTTCTGGATGCCGACAGCGGAGCAAGTCCAACAGTGAGCGGAACTCTCGAGAGGTCAGAGGCAGCGACAGAGATGCCGTGCCGTCTGCTTCGCTTGGCCCGACGCGACGCTGCTGGTTCGCTGGTTGGTGTCCGTTAGACTCGTCGACGGCGTTTAACAGGCTGGCATTATCTACTCGAAACAAGAAAAATGTTTCCTTAGTTTTTTAATTTCTTAAAGGGTATTTGTTTAATTTTTAGTCACTTTATTTTATTCTATTTTATATCTAAATTATTAAATAAAAAACTAAAATAGAGTTTTAGTTTTCTTAATTTAGAGGCTAAAATAGAATAAAATAGATGTACTAAAAATTAGTCTATAAAACCATTAACCTAAACCCTAAATGGATGTACTAATAAAATGGATGAAGTATTATATAGGTGAAGCTATTTGCAAAAAAAAGGAGAACACATGCACTAAAAAGATAAAACTGTAGAGTCCTGTTGTCAAAATACTCAATTGTCCTTTAGACCATGTCTAACTGTTCATTTATATGATTCTCTAAAACACTGATATTATTGTAGTACTATAGATTATATTATTCGTAGAGTAAAGTTTAAATATATGTATAAGATAGATAAACTGCACTTCAAACAAGTGTGACAAAAAATATGTAGTAATTTTTATACTTAGACATGCAATGCTCATTATCTCTAGAGAGGGGCACGACCGGGTCACGCTGCACTGTAGGCATGCGTCGAGAGATAGATTTGTAGAAGAGACTGGTGATTTCAGCGTGTCCTCTCCAAATGAAATGAACTTCATATATAGAGGAAGGGTCTTGCGAAGGTGGGATTGTGCGTCATCCCCGTCAGTGGAGATATCACATCAATCCACTTGCTTTGAAGACGTGGTTGGAACGTCTTCTTTTCCACGATGCTCCTCGTGGGTGGGGGTCCATCTTTGGGACCACTGTCGGCAGAGCATCTTGAACGATAGCCTTTCCTTTATCGCAATGATGGCATTTGTAGGTGCCACCTTCCTTTTCTGTCCTTTTGATGAAGTGACAGATAGCTGGGCAATGGAATCGAGGAGGTTTCCCGATATTACCCTTTGTTGAAAAGTCTCAATAGCCCTTTGGTCTTCTGAGACTGTATCTTTGATATTCTTGAGTAGACAGAGTGTCGTGCTCCACCATGTTCACATCAATCCACTTGCTTTGAAGACGTGGTTGGAACGTCTTCTTTTTCCACGATGCTCCTCGTGGGTGGGGTCCATCTTTGGGACCACTGTCAGCAGAGAGCATCTTGAACGATAGCCTTTCCTTTATCGCAATGATGGCATTTGTAGGTGCCACCTTCCTTTTCTACTGTCCTTTTGATGAAGTGACAGATAGCTGGGCAATGGAATCCGAGGAGGTTTCCCGATATTACCCTTTGTTGAAAAGTCTCAATAGCCTTTGGTCTTCTGAGACTGTATCTTTGATATTCTTGGAGTAGACGAGTGTCGTGCTCCACCATGTTGGCAAGCTGCTCTAGCCAATACGCAAACCGCCTCTCCGCGCGTTGGCCGATTCATTAATGCAGCTGGCACGACAGGTTTCCCGACTGGAAAGCGGGCAGTGAGCGCAACGCAATTAATGTGAGTTAGCTCCTCATTAGGCACCCAGGCTTTACACTTTATGCTTCCGGCTCGTATGTTGTGTGGAATTGTGAGCGGATAACAATTTCACACAGGAAACATATGACATGATTACAATTCGAGCTCAGTACCCCTGGATTTTGGTTTTAGGAATTAGAAATTTTATTGATAGAAGTATTTTACAAATACAAATACATACTAAGTTGTACAAAAAACCAGCAACTCACTGCACTGCACTTCACTTCACTTCACTGTATGAATAAAAGTCTGGTGTCTGGTTCCTGATCGATGACTTCTCCACTTTGTGCAGAACAGATCTAGAGCTCTTAGTAGAGCGGGAGATGTTGGTCGGCACGAGCATGATGTTCATGAGGTCGAACTGTGTGCCAGAGTTGAGGGTCACGTTGATGTCGAGCGGCACGTCGGAGTTGGAGGAGGCCACCACGTTGCCGATGTTGATGTCGGAGAAGCGAGCACCGTTGTCGTTCACGCCGTCGTTGTTTGTGGTGGTGTTCACGTTTGTGGCGGTGTACACACGGCCGTTGATGGTCACGCGGATGGTGGAGTTGCCGATGGAAGACACGCGGAGGTAGAGGTTGTAGGAGTTGCCGTTACCGCGGAGGTGTAACGAGCGGTGGTGTTCTGCTCGAAGCGGAGAGAGTCGCCCTGGTTGCCGAACTTCTCGGAGATGAAGGTGCGGGTCTGGTTGTTCACCTGGGTAGCGTGGATCGGGGAGATGGTGAAGCCGGTGTAGTCGTTTGGGCGAGGTGGGTCATGGAGCCGTTCTCGTGCACAGCGTGGATGTTGTTCTTGCGGTTGTGCACGGACACCGTAAGGCGCATGCACCAGGGTGCCGGACGGGAGGCGATGTTGCGGATCTCGTTGTAGTGGAGTGGGCGACGGAGGTCCTCGTTGCGCACCACGAGCGGCACGCCGGAGATGTTGCGGATGAAGTAGTCCGGGAAGTAGTTGGAGTTGCCGCGTGCGGTGAAGGCACCAGGAGGGCCGAGGGTGGTCTCGAAGGACTCGGTCTGCCAGTTGGTCACGGTAGCCACGCCTCGCGGTCGGGCCGAGTCGAGCCAGGAGCGCACGAACGGTGTGAGGAGCGGAGGGAGGAAGGTAGAGCAGTTGAAGTTCTGGTTGAACGGGAAGCGCCGATGTCGCCGGAAGAGATGCCACCGGGTAGTTCACGCGAGCGGCGAGGAGGGCGTGGGTAGTTGTGGAGCCAGGAGGCCCACGATGTTCGGGAAGGTGTTGGAGAGGCGGGCGCCGGAGAAGCCGTTGAGCACGTAGTTGGAGTTCACCTGGAAGAGGGTAGAGGAACAGCCAGTCCTGGGAGGTGAAGGACTGGGTCTGCTGCGGGCGGAGCCGGAGGCGTAGAGGTTGGCGCCGGAGGACACGAGGAGGGACTGGGCGCGAAGAGGGACCAGATGGACACGTACTCGAACACGTTGAGGAACATGTAGGTGCGGAACTCAAGCATCTCGTGGAGAGGGTGTTGAGCTTCTTGAAGGCTGTCTGGTAGGTGTTGATGCAGTAGTTGGAGTACTCCTTGGTGTAGTCCTTGAGGTAGCCACGGTAGGTGCGGAGAGTGGCAGCGGAGTCACCACTCTGCGTTGAGGATCACATCGCGGATGAAGGAGAGGTGGAGGTTGGCGGCCTGAGCGAAGAGTGGAAGAAGGAGTTCGTAGCCGCGGAGCTGGAACTGGGTGAGGCGGTTGAGGAAGAGCTGCTGCATGGTGTTCACCGGGGATGTGATGGAAAGGAGAGTCGGGTTACGGTTCGGGTTGAGGAAGTTGTCCACCTGACGGTTGAACTCGGCAACGTTCTTCTGGAGGCCCTCAAGTTCGGCGTTCACGCGGGAGAGGGTGTCAGCGTTGAGGCGCTGGTTGAGGAACTTCGGTCTCACGGAGGATGTCCTGCATGAGCTTGGTGTTGTCGTTTGGGAACACGAGGTCCCAGAGGCCATTGAGGATGCGCTTGGCAGCGAAGCCTCCGAGCTTCTTGAGGAGGAAGATGGCCACAGTACCCACAGGAGCCACGTAGAGTGAGTGGTCGTCGCGCTTCCACTCGGTCCACTCCTTCTGGATGGTGTCGAGGACTTGTACTGGAGGAGAACGGGTCCTGGGCCATCACGTTGTGGCCGTCGCAGATGGTGGTGCGTCCGGAGTTGAGCACGGGCTCCTCCACCCTTCGGGGCCTCGCACCTGATCGATGTGGTAGTCGGTCACGTCGGTCTTCAGGCCGATCTGGTTGCTGCTGGTGAACAGCTCGTTCACGGCCTTCTGAGCCCTCTCCAGGTCGTACTCGGCCTCGAAGGTCACCTCGGCGGGCACGAACTCGATGCGGTCGATGTACACCTCGTTGCCGCTGTTGAACACGTGGGCGCTCAGGTGAACACGCTGCTGCCGTTGCTGAAGTTGAAGGGGTGGTGAAGCCCACGGTGCGGAAGCTGCCGCTCTGCAGGTTGCTGCCGCTGCTCATGGTGGCGCTGAAGTTGCCCTGGTTGATGGGGCGGCCGTCGATGCTGGTGTGGAACTGCAGGTTGGTGGTGCTGGCGTAGCGGATGCGGACGCGGTAGCGCTGGCTCAGGGGGCGGTGATGTTCACGCGCAGGGTGCTGATCTGGCCGGGGCTGGTGCGGCGCAGGATGTCGCCGCCGGTGAAGCCGGGGCCCTTCACCACGCTGGTGCCGCTGCCCAGGTTGGTGCTCTTGGTCAGGGGATCTGGGTGATCTGGCTGCTGGGGATGATGTTGTTGAACTCGGCACTGCGGTGAATCCAGCTGAACATAGGTGCACGGATGATGCTCACGCTGCTGTTGCTGAAGCCACTGCGGAACATGCTCACGTGGCTCAGACGGTGGCTGAAGCCCTGTCGAGGTGGCACGTTGTTGTTCTGAGGGGATCTCGTCCAGGCTGTCCACGGTGCCGCTCTTGCGGTACACGGCGCTGGGCAGGTTGCTGCTGGTGCCGTAGGCGAACTCGGTGCCGTCCAGCACGCTCAGCTGCTGGTTGTTGATGCCGATGTTGAAAGGTCGACGGTACAGGGTGCTGCTCAGGGTGCGGTACACTCCCTGGCCCATGTGCCACGATGCGCTGCTGAGGTGCAGCGTTGCCCATGGTGCCGTACACAGGAAGGTGAACTCGGGGCCGCTGAAGCCGACGGGGCTGGCCATGATCTGGTGGCCGCTCAGTAGTACTCGCCGCGGTGGGCGTCGGTGTAGATGGTGATGCTGTTCAGGATGTCCATCAGGTGGGGCTGCGGATGCTGCCCTCGATGCCCTGGGCGCTGCCGCGGAAGCTGCCGTCGAAGTTCTCCAGCACGGGGTTGGTGTAAATCTCGCGGGTCAGCTGGCTCACGGTGCGGATGGGGTAGGTGCGGCTGTCGTAGTTGGGGAACAGGCTCACGATGTCCAGCACGGTCAGGGTCAGCTCGCGGCGGAACTGGTTGTACCTGATCCAGTCGCGGCTGTCGGGACCACACGCGCTCCAGGCCGGTGTTGTACCAGCGCACGGCGTGGTCGGTGTAGTTGCCGATCAGGCGGGTCAGGTCGTTGTAGCGGCTGTTGATGGTGGCGGCGTCGAAGCCCCAGCGCTGGCCGAACACGCTGACGTCGCGCAGCACGCTCAGGTGCAGGTTGGCGGCCTGCACGTACACGCTCAGCAGGGGCACCTGGTAGTTCTGCACGGCGAACAGGGGATGGCGGTGGTCAGGGCGCTGTTCATGTCGTTGAACTGGATGCGCATCTCCTCGCGCAGGGCGGGGTTGGTGGGGTCGGCCTCCACTCGCGGAAGCTCTCGGCGTAGATTTGGTACAGGTTGCTCAGGCCCTCCAGGCGGCTGATGGCCTGGTTGCGGGCGAACTCCTCGATGCGCTGGTTGATCAGCTGCTCGATCTGCACCAGGAAGGCGTCCCACTGGCTGGGGCCGAAGATGCCCCAGATGATGTCCACCAGGCCCAGCACGAAGCCGGCGCCGGGCACGAACTCGCTCAGCAGGAACTGGGTCAGGCTCAGGCTGATGTCGATGGGGTGTAGCCGGTCTCGATGCGCTCGCCGCCCAGCACCTCCACCTCGGGGTTGCTCAGGCAGTTGTAGGATGCACTCGTTGATGTTGGGGTTGTTGTCCATTGTTGGATCCTCTAGAGTCGACCTGCAGAAGTAACACCAAACAACAGGGTGAGCATCGACAAAAGAAACAGTACCAAGCAAATAAATAGCGTATGAAGGCAGGGCTAAAAAATCCACATATAGCTGCTGCATGTGCCATCATCCAAGTATATCAAGATCAAAATAATTATAAAACATACTTGTTTATTATAATAGATAGGTACTCAAGGTTAGAGCATATGAATAGATGCTGCATATGCCATCATGTATATGCATCAGTAAAACCCACATCAACATGTATACTATCCTAGATCGATATTTCCATCCATCTTAAACTCGTAACTATGAAGATGTATGACACACACATACAGTTCCAAAATTAATAAATACACCAGGTAGTTTGAAACAGTATTCTACTCCGATCTAGAACGAATGAACGACCGCCCAACCACACCACATCATCACAACCAAGCGAACAAAAGCATCTCTGTATATGCATCAGTAAAACCCGCATCAACATGTATACCTATCCTAGATCGATATTTCCATCCATCATCTTCAATTCGTAACTATGAATATGTATGGCACACACATACAGATCCAAAATTAATAAATCCACCAGGTAGTTTGAAACAGAATTCTCTCCGATCTAGAACGACCGCCCAAGACCACATCATCACAACCAAGACAAAAAAGCATGAAAAAGATGACCCGACAAACAAGTGCACGGCATATATTGAAATAAAGGAAAAGGGCAAACCAAACCCTATGCAACGAAACAAAAAATCATGAAATCGATCCCGTCTGCGGAACAGCTAGAAACATCCCAGGATTCCCCAAAGAGAAACACTGGCAAGTTAGCAATCAGAACGTGTCTGACGTACAGGTCGCATCCGTGTACGAACGCTAGCAGCACGGATCTAACACAAACACGGATCTAACACAAACATGAACAGAAGTAGAACTACCGGGCCTAACCATGGACCGGAACGCCGATCTAGAGAAGGTAGAGAGGGGAGGACGAGCGGCGTACCTTGAAGCGGAGGTGCCGACGGGTGGATTTGGGGAGATCTGGTTGTGTGTGTGTGCGCTCGAACAACACGAGGTTGGGGAAAGAGGGTGTGGAGGGGTGTCTATTTATTACGGCGGGCGAGGAAGGGAAAGCGAAGGAGCGGTGGGAAGGAATCCCCGTAGCTGCCGGTGCCGTGAGGAGAGAGGAGGGCGCCTGCCGTGCCGGCTCACGTCTGCCGCTCCGCCACGCAATTTCTGGATGCCGACAGCGGAGCAAGTCCAACGGTGGAGCGGAACTCTCGAGAGGGAGAGGCAGCGACAGAGATGCCGTGCCGTCTGCTTCGCTTGGCCCGACGCGACGCTGCTGGTTCGCTGGTTGGTGTCCGTTAGACTCGTCGACGGCGTTTAACAGGCTGGCATTATCTACTCGAAACAAGAAAATGTTTCCTTAGTTTTTAATTTCTTAAAGGGTATTTGTTTAATTTTTAGTCACTTTATTTTATTCTATTTTATATCTAAATTATTAAATAAAAAACTAAAATAGAGTTTTAGTTTTCTTAATTTAGAGGCTAAAATAGAATAAAATAGATATACTAAAAAATTAGTCTATAAAACCATTAACCCTAAACCCTAAATGGATGTACTAATAAAATGGATGAAGTATTATATAGGTGAAGCTATTTGCAAAAAAAAGGAGAACACATGCACACTAAAAGATAAAACTGTAGAGTCCTGTTGTCAAAATACTCAATTGTCCTTTAGACCATGTCTATTGTTCATTTATATGATTCTAAAAAACTTGATATTATTGTAGTACTATAGATTATATTATTCGTAGAGTAAAGTTTAAATATATGTATAAAGATAGATAAACTGCACTTCAAACAAGTGTGACAAAAAAATATGTGGTAATTTTTTATAACTTAGACATGCAATGCTCATTATCTCTAGAGAGGGCACGACCGGGTCACGCTGCACTGCAGGCATGCAAGCTTGGCACTGGCCGTCGTTTTACAACGTCGTGACTGGGAAAACCTGGCGTTACCCTTAATCGCCTTGCAGCACATCCCCTTTCGCCAGCTGGCGTAATAGCGAAGAGGCCCGCACCGATCGCCCTTCCCAACAGTTGCGCAGCCTGAATGGCGAATGCTAGAGCAGCGAGCTTGATCAGATTGTCGTTTCCCGCCGGTTTAAACTATCGGTCGTTGTAGCGTCGAGCGTGTGGTTGAGCTCGAGCCGTAGAACTTGTCTTCGCGAAGCGTCATCGCGATGAGCCTCCATCTTGGGTGGCGGTGGGAGTTCTTCATGACTTGGTCGATGGCGAGTGCGAGTCACCCTGGCGTCGGCGTCGGACCCCGGCTCGATGGCGTCGCGGCGATTGACGAGCCGTGCCGGCATTGTTCGCGGCCGGAAGTGGGCGTAGCACATAAGCGTAGGTGCTTCAGGCGAGATGAAGAGCGAGCTGCAGCCGCTCCTGTCTTCATCGACCGTCGAAGAACATGGTCGAGTTCGGTTGGATCGGAACGTTGGGGCCCAGGTGTCGACCCATTCGACCACAAGTCGCGAGACACAGGACTTGATGGCGCCGAAGCGAGCGAGATCGCCGCCCATGATTTCCACCGCCCACTGCGATTCTACAGGGCCTCTCGGCTAGATGATCTCGGGGAAGGATGACACCGATTCACGGATGAGACTCAGTAGTGTGCAACCCGCCGCGTCGGGATCACCGCGTCTGGCCCAACCGAATTTGTGGGTAGCGGATCTTGGTCTCGGACGATACCTCGTTGATGAAGTAGACTTGCCTCGGGCGGGCAATGCATGCCCCTCTTCTCGTCTCTCAACCACGATCGCGGCTTTAAGCAGTTGCGGCGGCGTAGATCAATAAGGCTTCTCCGTGAGGCACCAAGATAGGCGCGTTCGTGAGGCGCCTTGAGTTCCAGGGCTTCCTCGGCCTCGGTCCAAGTGAAGCACTCGGCCTTCCTTAAGAGCGTATAAGCGAGCCTCTTTCGCCAGCGTGAGATGAACGGCTCAGGCCGCAAGACATCCGTGACTCTCGTCGCCCTTCAAGTCCTTGATGGGCCCCATCTTTGTGATGGCGCGATCTTCTCGGATTGGCCTCGATGCCCCACTCGGAGACAATGAACCCCAAGAGCATGCCTCGAGGAACCCCAAAGACACTTCTCGGATTAAGCTTCCGCGTCTTCGCCTTGAGACATCGAATGTCACTTCAAGGTCGGAAAGGAGGTCGAGGCTTTCCTCGTCTTGACTACGATGTCATCGGCGTAGGCCTCGACCGTCCGCCAATGTGTTCGCCGAACACATGGTTCATACACCGTGATGCGTCGCACCGCATTCCTCAGCCGTGAGCGGTATGGTGACATAGCGATACATGCAGAAGGGTGTGATGAAAGAAGTCATGGTGTGGTTCTTTCATCTGATTTGATGGCCACAGTAGGCATCGAGGAAAGACAAGGTTCGCACCCAGCGGTGGAATCCACAATTTGATCGATGCGAGGCGGAGGGTAGGGAACCTTCGGACATGCTTTGTTTAGACAGTGTAGTCTACACACATCCGCCTTTCCCTCCTTTCTTTCTCACGAAGCGAGGTTGGCAAGCCATTCGGGATGGAATACCTCTTTGATGAACCTGCTGCTTGCCATTAGCTTGTGGATCTCTCGCCTATGGCTCTGCGCTTTTCTTCATCGAATCAGGCGAGAGGCCCTTCACAGTCGGGCTCCAACTCGGATATCCAGTGGTGCTCGGCGACATCCCTCGGTATCACGGGCATGTCAGGGACTCCAGGCGTCGGCGTTCTGCGGAGAAGTCGGCGACACACTTCCTATTTGGGATCGAGCTCGGGCCGATCCGGATCCGCAGGCGTCGGCTGGCGGGGTCGAGGGGCGGACTTAACCGTCTCGTGGCTCGAGTTGTCGGCGTGGCGCTTCACGTCTGCACCTCCTTAGAGAGGCTTTCGGTCGTGATAGGGGCCTTGGATTGGCGAGGGCCTCGGCGTACTCCGCCTCCGTCGCATTGAACGCGTGTTTGTACGTGGGGCGACGGTGACCCGTTGGGGCGGCATCTTGAGCTTGAGGTAGGTGTAGTTGGGGCGGCCATGAACCGGCGTAGCATGGCCTCCCGATCTTTGCGTGGTAGGTTCTCGGAACGAGCCACCTCGAGCGTGAGGTCTCCCTTGAAGTTGGAGGGTGTTCGAAGCGACGGAAGGTCGAGTTGTCAAAATTTGGGGCAGCTTCGGGGATGATCGTGGAATGGCGCGGCGCTGCGGAGAGGATAGATCAACAGCGAGGCGAGGGTCGGCATAGATGATGTTGAGGCTCTTGCCTCGATCCGCGAGACCTTGGTGAGCTGGCGTCCACCGATGGCCAAGTCGACAGCGGCGGTATTTCCCGGGCTCAAACGCGTGGTCGGGGGTGGTCAGCTGATCGAAGGTGATGGGCTTGTCGGACCAGTCTAGGTAGTGAAAGTAGCCTAGAGGGGGTGAATAGGCTAATCTGAAAAATTTCACAACAAACTTGAAGATTGTTATATACAGTTCATTGGTGCAAGCGGTTCAACAGCTACGTCATAAGTTGAACCACTCGAACCAATCCAGCTGTTTTATAAACTTTAGACTAAAATCTACTCGAAAGTATTTACATAAGTTCTTGAGAAAGTAAGATATAGCTAAGTGAGGATGAAAAGATGAATATGTAAAGGCTATTTTACTTCTAGATAAACTCTAGGAGAAATCTTTATGTCAATCTTGCAAGTAAAAATATCTCAAGTTGAAACAAGTAAGAACACAAGACACAAGATTTAATCAGGGTTCTACCACAAGGTGTCCTACTCCCGTTGAGGCCCACAAAGGGGGGTCTTTTCAACCCTAATCCTCCAGCGAGCCACAAAGGTCAAGGCAATCTCTTCTTATCTTAGCTCAAAGAGCGGGTGATACCAACTTCTTAGGGTCGTCCACAAATTTGGAGACTCCCAAGTAACCTCGAAGATCTTGAAACCTAGGGTTTCAAGAACAAAACACAAGAGAGGTTTGCACAAGCTCAAGTCTTTGAAAAAGAGATGGGAGAGGAAAACCAAATCGTGAGCACAAGCACAAACCTCACACCCAGAGCTCCTCCAACAAGGTTGAATCTTGAGGAAGATTTAAGTGTGAGAGAGATGGAGAAGATGAGTGCTTTGTCTCAAGTTAGGTGAGCAATAAATGAGTGAGTGTTGGTGTCTTGAAGAAGAGAGAGAGGGTCTATTTATAGTCACGGCTCAAACTAGCCGTTTGACCAAAACCCGTGGCAAGTTGAACAGCCCCTAGGCGGTTGAACCGCCAACAGTCGTGGGGTGTCTGCCGTCGGTGCGGCCATTGACGGAGCTCTTGCGGTCAGACGAGCGGATCGGTTGAGCAGCCCTAGGGCGGTTGCCGCCCGCACACCACAGCGACACTTTGCCCGGTCGGTAGCGGGAGCTGCGATCGAGGTCGAGGGTCGAGAGACCAGTTGAGCTACCCAGGACGGTTGGTGGTCTAGACCGAGTTTTGGAGAAACCCTAGCTCGGTACGCCACAGGGCAAGTTCATTTGGGTATGGTCGAGGGTCCACAATTGAAGTTGAAGTTCATTTGGAGTTGAAAGTTCAACTCATTTGAAGTCGGCTCAATGAAAAGCTCATTGCGGTGAAGAAGCTCATCTGGCGAAGTTAAGTTAATGGAAAGCTCAAGTGTTGAAGAAGTTATTTGTTTTCAACAAGAACACTCTAGGTTTCTCAACCCAAACCATGGTCGACCAAATAATGTTAAAGAGATTTTTGGTTTTCAAAAATAGCTTTTGAATTAGAGGACTTGAGCTATAGCAAACACTGTGCACAATGCGGAAAAGAACAAGGAAGAATTACATCATGCAACACAAGATTTTACATAAATTTTATCGTCATTTGCATGAAGTCCTTGGTGCTTCCTTAAGTTCTGTTTCCTTCTAATCAAACAAAGAACAAAAATTGTTAGTACTCTTATTTGTTTTGTCATTAAATCACCAAAACCCTCACTTGGGGTTGATTGCACTTACAATCTCCCCCTTTTTGGTGATTGATGCCAAACAATTAAGATCAAAAGATATATATTTGCAATAGAAATTTCTTTTGAATGATTGTATGTAGATGGCTCCCTAAATGTGTGCACGATTGTGAATCAACGTCTTGACATAATATGTCATGTGAGCAACACATTTAGAGATAGTGACAACAACCATACTAAATACAATTATCAGGGGTGCAAGAGTGTCATGACAGAGTTGTGATGCATGACTATCTCTAAAAACCATTATTTTTACTTCATAGCAGTAGACATTACAAGCGATAGGACAAAGATGATGGCGAAGACCATCATATTACTTTGAATAACAATCAACGGTTTATTTCATACAATGGTAAAGGTACAAAGCCACAACGGTGGCCAAAACAAAAATAATCCAAAAGAAAAGCGAAGAACCACAAACTAGAAGTTGGATTTTCTCCCCTTTGGCAACAAGTACCGCGGAGAGAGACAAAGGATACAGAATCAATATCCTCCGGGAGGAGGAGGATATGGCGGAGGATATGAAGTGTAGTCGGGAAAAAAGTCCTCATCACGTCCTCCTCAAAGTGGAGTCAGAGGTGGCTCATCAGTATAGAAAAAGCAGATCGAAGATGTGCGATGATCGGAGGAGGAGGTGGAGGGTGGTGGTGGTAACAGCCGGATGGCCTAGGGTCACAGAGAATGGGTCCGGACCGGGTAAGGGAGGCGGAGGTGGGAGGATGATTGACCGGATCCCCATGGTAGGGGCGGGGCAAACTCCTGCGATCATCAACATAGACTTCTTCATCTTCATCGTCTCTGAGCATAAAGGCACCAGTAGGCCTGCGGTGCCACTCATTGATCTCCGGGGAGAGGAGGATGGAGAGGCACATCAGGCGAGCGAGGACAAAAGGCATGCCCATGGAGGAAGCTTGGCGGCGAAGATTGTCGTCGATCCCAGTGACATCGTGCCACCTCATGGACATCAGAAAATGTTGCGTCACATGGAAAAAATGCATAAACCCATGGACCAAGCGAGCACCCATCCCACGGCCCGACCTCGACCATGACCACGAGCACGTGGCATGGGAGATCCATGGCCAGAGAGACGCGGCGCGATGAGAGGAGGATGGATGATGGGAGAGTGGCGGGGTGGCTCGGTGGAGGAGGCGGGGCCTCAGGCGTGGGGCCCAAATGCGGCGGTGGGTTGGAGGAATCAACGGTAAGTGCATGGCGGGAGTGTTTGGCGGGTTTTGAAGTGGGTGCAGGTGACCACTTGATCATCTTCATGATAAAGGGTGCATGAAGACACCTCGAGAGGAGACCGAAGCCTGACGATCTCTTCCCGATCATGTCAAACACATTGATGCGGCGAGGCTGTTTGGAGCAAGGAGGAGGAGGAAGACTCGACTCTCCCAGGGCGTTTATTTGATTTCCGCCCGGAGAACGGTCATCCGGGACAAAATTAAGGTACTGTAGTACCTATGCATGTTAGTTTGTTGTCCGAACTCCAGCTCGGTAGAACATGAATGAATTACGTCTCTTCGCATAAACGGCCGAATGTCATGCACTCGCATGTTGCTATACGAATATCGCATCGAGAAGCCGAATATGGGCAAAGCAGCGATAGCGACTTTGTGCGATTACCGGATGAAGAAATACATGATCGGGTAATCATACTCATCGGCTTCCTCATCTACTTTCTTTATCGGAGGGTGGCATAGGCGAGCTACCACCTCATCGTTCGGGGTATTCCATGGTCATAATGTTCGTAACCCTTTCTCTCTAAGTTTACGAGTGGCGACATGTCGGCATCTCCATATTCTCACAACCCTCCAGTCAATAAATCTATGTGGACGTGGGTGTTTCTTGGAAAGAATGCAGAATTGTAGAAATCGGCTGAAAAGATTCCGAACGAGTCTGGACACACGGATGAGGTGAAAGTAGGGTTGGTGAACCTCCAGCTGAAGGTGTCTATTCCTTGAGTGAAGTCGGCAGGAGATTCATGCGGACCGCGGTGGCGTTCGCGGATGCATGTGCGATCCGGTGGCGTAGAGCATCGGCCGGAAGGTCAAGTCGTCGAGCGCCCATCCTCCTCTGACAGAGCATACCGAAGGAAAGTGCTCTGGCGAGGATGAGGCGCCAACCTTTTGGCGTCTCCGCGGTAGTCTGCGAGCATGGAAGAGCCCTCACCATCCATCCGACGAGCCACGTCCTCTCTAGAGAGGGGCGGCGAGGAGGAGAGATGGGAATTCCTCTGAATCTCATGGTCGCTGCTAAACTCAATCACAGAGGGTTGCGACGGACGCACCATGATTGAAAAAGGTGGTGGCGTGAACGGTTGAGGTGTGTGAAAGAGTGGCTCTAAGGTTGTGAAGCGACTAAGGCAAGAGTGAATGAAGTGGGAGAAGAGAAGGGAGAGTCAAGTATATAGCGAGTTGGTGGTCTCAGGACAGTTCAAGCCCCATGGGCGGTTCAATTTGGTTCTAACGATTGTGGTCGAGAGAACGAAGCAGCAGAGGAAGATGCCAAAAATCAAATTTGGCGAGATTACGAAAAAGTGTATCTGCGGAGATTCTATGAAAAAAATCAAAACCAAGTGTTTGCTTGAGATGAGGCCGGACGGCAAAGAAAAAAAGCTGCATAAAGAAAGTTCTGCGGCGCCTGTATCCTCACCTTCTTTGCGGAAGTGACAAAAAATATTTCCTTTGCTTGCTGGGTGACACGTGATCACGAGCAAGGCTGCGCCTACAAGCGATAGTGTGGGGCTAAAAAAGCGTCTTGTTTAATTGTGGACGGCCCAGACGGAGGAAAGAAAAATTAGGCGCCCACGACATGCATGGTGTAGCGAGGAGAGAAAGAATCTGCGACATGGTCGCGAGGTGGCGGCGATCCGAAAAAATGCTGCCGCGCCCGGTGAAGAAAGTGGCAGACAGAACAAATAAAAAATTCTGCATCACCTGTGACACTGCTCTACGGACACAAAATACATGCGTAGCATTGCGCTAGAACCACGGTGGATCACGCATGGCGTACGACATTTGCATGCAAAGCTAAGACGATGACCCTAAAAAGAATTACAGCGGCCAACAAAGCGGCCGGACTGAAAAAGTGGCCTAGCGTGATTCCTTGGCTGGCGACAAAGAAAAAAATAAATCTGCAGCGAACCAGAGGAAGTGGCGGGACAGAAGAAAAAAAAATCTGCCTTGAGCCACATGAGAGGAAGCGACCGTAAAAATATTGTTGCGCCCCGAAAAAGGTCACATTATGGAACTGCGGCCGGTAAAAAAATTTCACCTGTTTGTCACAAAAAGTGAAGCGGCGGTCAATAGAAAAAGATCTTTGCGCTTGAGATGTGGCCGTTAAAAAAATCCTGCCGTCGACATCGCTGCGGTGGCGACCTGCGTGAAACAGGCTTCTTGTGGTCAGGCAGAGCGGCAAGTTCAACCGTCCCCACCATGTCGGTTCAACGGTTTTACTAGGATGGTCTGGTAAAATGGTTAAGCATTGTTGAAGTTCGGCTCCTAAGTTCAATTTGGCCTCCTCCGGTGAACTAGAAAGTTCAGTGGTCAAGCAGTTGAGCCAAAAAGAATCTTAGCACTAAAATGCATTGATTTTAACATTTTCACCAGTTTTTGAAAGATCATCACATAGCTTTAAAACTATCAATCAAAAATAGTGAATCAAACAATTTTGTGACCGTTTTCAAGTTAAGTTCAGAATCCAAGATATTTAATTCACTCTTAAGAAAACAAACCTAGTCGATCGAGTTTTTGTGAAGTATAACTAGTTGATTTTCGATTGCTCACATGAAACAATTCGATATCTTTGGCATTGGTCTCTCAAGAAATGGTGTGCGATGTCAATATGTTTAGTTCTAGAGTGTTGCAGGGTTGTTTGCAAGTTTTATGGCACTCTCATTGTCACACAAAGTGGAATTTTGTTAAACTCACAACCAAAATCTCTAAGGGTTTACTTCATCCATAACAATGTGCACAACATGCCCCAATGCTATGTGCTCGGCTTAAGTGGAAAGTGCAACACAATTTTGTTTCTTGGAACTCCATAATACTAAGGATCGCCCAAGGTGTAAGTCGATAGTGCTTTTGATCTACTTTTGCAAGCATAATCTAGTCAGAATAGCCAAGTAAATCGAAAGGAGCCTTTGGGATACCATAATCCTAGGTTTTGGGTGTGAACTAAGTATCTTAGAATTCTCTTAGCACTACAAGATGGCAATCTTTAGGATTTGCTAAAACGTGCACACATGCAAACACTCAACATAATATCGGTCTAGATGCACATAAGTAAACTAGTGATCCTATCATTGATCTATATAATGTTTGATCTCTGGGTTTACCTTCCTCATTTAGTAGAGATGTCCATTTGATGACATTGGAGTCTTGGCGTGTTTTGCCTTTTCCATGCCAAATTTCTTGAGCATATCTTGTGTATATTTGGTTTGGCATAGAAAAGTACCTTCCTTGAATTTGTTTGACTTGAAATCCCGAGAAGTATTTAAGCTCGCCCATCATAGACATCTCAAACACATTAGTCATTACTTTGCTAAACTCTTCACAAAATTTTCATTAGTACTACCAAATATAATGTCATCAACATATACTTGGCACACAAATAATTCATTGTCAACTTTTCTAGTAAATAAGGTAGAGTCGGCTTTTCCTATTGTAAACCCATTCTTAATTAAAAATTCTTTAGGCAGTCATACCAAGCTCTAGGGGCTTGTTTAAGCCCGTAGAGTGCCTTGTGAAGTAGATAAACATGATTTGGCTTCTTTGGATCTTCAAAACCCGGAGGTTGCTCCATATACTCTCTCTTGTAGTGGTCCATTTAGAAATGTGCTCTTGACATCCATTTGGTATAGCTTGAAATCATGGTTAGTAGCATATGCAATTAATATTCTAATTGATTCTAACCTTCTCTGAGCGCATATGTTTAAAATCAAGTCCTTCCACTTGAGTATAGCCTTGGGCAACCAGGTGCCTTGTTTCTTAGCACCATGTTCATCTTGTTTGTTCCTAAAGACCCATTTAGTCCCAATCACATTTTGTTTGGGTCTTTGGACTAAGGACAGACTTCATTAGGGTGAAGTTGTTTAACTCTCTTGCATGGCAATTATCCAATCCGGATCACCCAATGCTTCTTCAACCTTAAGTGGCTCAGAGGAAACAAGCGAGTAAAATTCACAAAAATTAGCTAAGCGAGATAGAGTCGTTATCTCTGATGCTACCCGGGATGTTGTCACTTAGGATGATCCCTTTGAATTGTATGATGGACTCTTGGATGAGGCAGCGATGGTTGTCTTTGTATTTGCTCCTCCTCATCATTCACTTGATCAAGAGGTACTTCACCATCAATGCTTTCGTGTTCATCTTCTACCACTTGTTGGCATCCTTTGATGATTTTCTTCATGGCTTGGATGACTTGAGGTTGATGGGTTTGCTTGGGTGGAGACTTTGTCACTCACCACCTTTGCACCCACATCAACAACCTCATTGGTCATCCAGAAAGTTCCTTCCTCATCATCCTTTCTTGAGGTCTCACTTCACCTATTGCAAGTTTCTTTGTATTCACAAGGTTTCTTCATTTCTGCTGACATTAGAAACATGCCCTTATGAAACCATTAGACTCATCAAATGTCACGTCTATCGCTATTTCAACAAGACGGTGGTATTGTTGAAAACACGATATCCATGCGCATTTGATGCATAACCAACAAGAAGCCCTCGTCCACTCTAGGAGCAAACTTTGAGCTCTTGACTTTCTTGTTAAGAATAAAACATTTACAACCAAATACTCTAAAATAATCAACTTTAGGTTTGTTACCCGTGAGAAGCTCATAAGCAGTCTTTTTGTAGATCTTATGAAGATAGAGGCGGTTGATTGCATGATAGCGGTGTTGACGCCTCCTAAAGTTGTCGGGTGTCTGTACTCATCCAACATGGTTCTTGCGGCTTCAATTAGAGTTCGGTTCTTTCTTTCCACAACACCATTTTGTTGTGGAGTGTAAGGTACAGAACTCATGCTTGATTCCCTCTTCTCCTAAGAATTCTTGACACCAGTGTTCTTGAATTCCGTCCCATTATCACTTCTCACTTTCTTGATTTTGAGCTCAAATTCATTTTGAGCTCTCCTCATAATTTCTTCAATATTTCTTGAGTTTCACCTTTATCACTAAAAGAAAACCGAGGTGAATCGAGAAAAATCATCAACAATGACTAAACCATACTTACTACCAATGCTAATGTAGGCCCACAGGTCCAAAGAGGTCCATGTGAAGAAGCTCCAATGGCCTCTTTGTTGTGACCATTCTTTGATTGATGTGGGACTCCATGTTGCTTTCGCTTGGCATGCGCCACAAACCCTATCTTTCTCAAATACAACATTTGTTAGTCCAATGATATGATTATCCTTTTGAAGTTTGGCCAAATTCCTCATCTGCAGCATGGGCTGGCAGCGATGCCAGCCACCGTCGGACTTTGCCACTAAACAAGTCTCGTCGTCACTTTACTTGTTGTGAAATCAACAAGATAAAGCTTGCCCTTCAAGCGTAGGTAAAGGCAGAGTCCTCCCTTCTAAGGATCTTCATCCACATCCGAAAATAAACAATTATAACCCATTCCACAAAGTTGTAAACGGACAACAAATTATAGCTTAAAGAATCTACCAATAAAACATTTGAAAGTGATTGTTGGTCGAGATAGGAATTTTACCAATACCAATCACCTTACTCTTGCCACTATCTCCAAACACAATTTCTTGTGCTTCTTGAGTTAGTTGCAATGTATGAAACTATGTCTTTCTCCCGGTCATGTGATTTGTACATCCCTTTGTCAAGCACCCAACTTGACCCACGGAGGAGTAGACACCCAAAACAAGTTTAGGCTATGCTTTTAGGTACCCAAATTGAATTGGGTCACTGTTAGTTATAGCCTTGGGTACCCACACTTCTTTTCATGACTTCGTTTAGTGTAGGCACCACATATTTCACAACCAATTTTCCTAAATCCCAAGTCAACATATAATCACAAAGATATGAGTGGGAAATGGGAGCATTAGATTTTGTCCACTTGAGGATCACTTCCTTCATCTTACTCTTTGTGGAACTCAAGTTAACTTTACTGAGGTGACTTGTCATTTGAGTGAATCCTCCCCAAGGCATCATATGGTGGTTCCTCTATGAACTTGGGGATCTCCACCCTTATGCAACGTGCTAGGGTTTTCCTTGGATGAGTTGAACCCACCCCTTCTTCCATTGTTGGGCTTAAGGGACTTATACTTGGGTTCAACTTTCTTTAACCCATCATTGCTAGAGCATCTTTTCAAAATTTCTTTGACCTTCACCATGGGCTATTCTTCCTTGCATGGTTAGTTAGACAACAAGAAGCATGATATTTGGCACAATGTTTGCAAGAATTATCATTTAATAGACTTAGATTCAAGCACTAAAGATGAGGCATTGATGTTCTTGCAATTTTCAAGTTGCACTTGAAGTTCTTGATTTTGAACATTCAAGCAACATGCTTGCTAAAGTGCATTCTTTTCATTTGCAAGATTAGCTATAGAGTTTTCATATTAATTACTTCTTTATCTTTATTCTCTATAGTTATCTTGACTAAAGATGCTTCCTTAGTCAAGATATCTAGCATTTCATCTTTATTGTGAAACAATCTTGAAGCTCTAGGTTTCTTTCCTTTTCAAGGATAAGCAAGTCTTCTTGAGCATCAGAGTTGCTTTTCTTTATCTAGCTTCTCCATTAATTTGGTGATAACATTGTATCCATTCAAGCCAAATTCTTTAATCATTTATTTTTTCATGGCAATTTGTTCATCATCATCATCATTTGGAAAATCATTACTAAATAAGGTTACCTTATCTCCTTTTGCCATGAGGTAAGTTGGAGTGTAGGAGTAGTCTTGTAGGTTTGTGAAGAGTTGCGAGCTTGATGTAGATTGGATGGCCACGTTGCCACCACTTCCTCTTCCTCGAGCTAGAACTCTCTTCATCGGGTTCCATTCTTCACCAATATAGGCTTGACCATATTTCTTCTTCTTGAAGTGTCCTTGGTCTTGCCTTTTGAACTTGTCCTTCTTGTATTCCTTCTTAGCTTCTTGTTCTATCTTGTTAGGACAATTGCTAGAAATAGGCCAGGTTTGCCACATTCATAGCATGCCCTCTTCTTTCCTTTCCTTTGAAACTTGTCATTTTCCTTACAAATTTCTTGAATGTTTTGATGAACATATTTGTGTCTTCATCACTTGAGCTATCTTCATCACTTGAGGTCGCGACCACTTTCTTTGCTTTGGTCTCTGCTCTTCTTGGGTTGTCTTGTTCATTAGTGATCAAGGCATGAGAGTCCCTGACTTGATAGGGGCTTCTTGGACTCGTGTTGTTGAATCTTTGCAAATAATTGATGAGGCGTCATATCCTCATAGTCATCACGATCCTAATCATCCTTGCAAGACTCTTATCCTTTGCTTTATAAGCTCTCATGAACAATCTTGTGACCTTGAGTCACTCTCAATCTTACTCCCAAGAACTCTTATTTTGTTGACCAACACCATCAGCCGTCAAAGAGTGATTAGGCGACTCACCTTTGTCCAATCATATCTTGCAAGCTCACTTTCCAAAGCTTCAACTCTATGTCTTTTAGCTTTGGGATCTCCTTCATGTGACATTTTAAGAATATTCCAAATGTCACGGGCATCTTCTCTTCATTGAACTTTCCAGTATTCTTACGGACAAGACTTCTTTAATTATGCTCACTGCTTGAGCATTTGATGAACCTCTTGCATCATTTCGGAGTCATCTCTCTTCTCCTTGGGCGGACTTATACATACCTACATTAACAATCTCCCAAAGACTAGGATGCACAGGTTAAATCGACTTCATCTTGTCGGCCCACTCATCATAGTTCAACTCACTAGAGTTGGTAACTTTCCAAGTGGAGCGGAAGAGAAGTTGGAATATAATTCTAGAGTAATCAAATTGAACTTTGCTAAATTCATTACCTTTGCTTTTGGTAGATGATCCTTGGTGTTGAGACTTACCTTGCTCAATACCTTAGACACCAAAAGATCCACTAGATCGTCATTATAATCAAATGTTCCCTTACCTTTATCTTCTTGGCCTTTCTTCTTGATTCTTCTTCTTTGGCCTTTTTCTTAGCATCTTCCTCTTTCATTTTGAGAACATTCTCTCGGCAATCTTCATGGCGGGTAGGACAACAGGGTCCACTTCCTCACCGGAAGATGTGATGGGGATTTCCTCGGAGAGGATCCACATGGTCCCGTTAGTAGACATGATCGTTTACTCACGTGGTTGGCGTAAAGCAGTCTGACTTTGATACCAATTGAAAGTAGCCTAGAGGGTGAATAGGCTAATCTGAAAATTTTCACAACAAACTTGAAGATTGTTAGATATGATTCATTGATGCGGTAGGTTCCATGACGCTACGTCGCAAGTTGAACCACTCAACCAATCCAAGCTGTTTATAAACTTTAGCTAAAATCTACTCGAAAAGTATTTCACAAGTTCTTGAGAGTAAGATATAGCTAAGTGAGGATGAAAGATGAATATGTAAAGGCTATTTTACTTCTAGATAAACTCTCACGGAGAAATCTTTATGTCAATCTTGCAAGTAAAAATATCTCAAGTTGAAACAAGCAAGAACACAAGACACAAGATTTAATCCGAGTTCGCCACACCACAAGGTGTCCTACTCCCGTTGAGGAGCCCACAAAGGGCGGGTCTTTTCAACCTAATCCTCCAAGCCGACCACAAAGGTCAAGGCAATCTCTTCTTATCTTAGCTCAAGAAGCGGGTGATACCAACTTCTTAGGGTTCGTCCACAAATTTGGAGACTCCCAAGTAACCTCGAAAGATCTTGAAACCTAGGGTTTCAAGAACACCAGAAAACTCACAAAGGGTTTGCACAAGCTCAAGTCTTTGAAAAAGAGATGGGAGAAAACCAAATCGTGAGCACAAGCACAAACCTCACACCCAGGCTCCTCCAACAAGGTTGAATCTTGAGGAAGATTTAAGTGTGAGAGATGGAGAGATGAGTGCTTTGTCTCAAGTTAGGTGAGCAATAAATGAGGTAGTGTTCAGCCGTCTTGAAGAAGAGAGAGGAGTCTATTTTATAGTCGGCTCAAAAACTAGCTGTTTGACCAAGCCATTTGGCCGGTTGAACCGCCACAGCGAGTCAGGTATGCTGCCGATCATGGCGGTGGCTGGTGACCCGACTGCGGCGGGTCGAGCCCGAGACAGTTGAACGCCCCTAGAGGCGGTTGGGCCCTGACACCTGGCGACTGGTGCGGTCCCGGTAGCGGTACCCGAATCGAGAGGTCGAGTCGAGGGCAGTTGAGCTACCCCGAGGACGGTTCGGTGGTCTAGACGAAGTTTTGGAGAAAACTGACTCAGTGCCACAGGGCAAGTTCAGCTAGGTATGGTCGAGAAGGTCACGTACAGTTGAAAGTTCAACTGATTGAAGTCGGCTCCCAGTGAAAAGCTCATTGCGGTGAAGCTCATCACAGTGAAGTTAGATTGGTGACTCAGCTACTGTG

>nano10

CTATTTGCAAAAAAAAGGAGAACACATTTTGCACACTAAGATAAAACTGTAGAGTCCTGTTTAATAAAATACTCAATTGTCCCTTTGGACCATATCTAGCTTGTTCATTTATATGATTCTCTAAAACACTTGATATTATTGTAAAATATTATAGATTATATTATTAAATTAGAGTAAAAAGTTTAAATATATGTATAGATAGATAAACTGCACTTCAAACAAGTGTGACAAAAAAATATGTGGTAATTTTATAGCTTAGACATGCAATGCTCATTATCTCTAGAGGGGCACGACAAGTTGCTGCACTGTAGGCATGCGTCGAGAGATAGATTTGTAGAGAGACTGGTGATTTTCAGCGTGTCCTCTCCAAATGAAATGAACTTCATATATGAGGAAGGGTCTTGCGAAGGATAGTGGGATTGTGCGTCATCCCTACGTCAGTGGAGATATCACATCAATCCACTTGCTTTGAAGACGTGGTTGGAACGTCTTCTTTTTCACGATGCTCCTCGTGGGTGGGGGTCCATCTTTGGGACCACTGTCGGCAGAGGCATCTTGAACGATAGCCTTTCCTTTATCGCAATGATGGCATTTGTAGGTGCCACCTTCCTTTTCTACTGTCCTTTTGATGAAGTGATGACAGATAGCTGGGCAATGGAATCGAGGAGGTTTCCCGATATTACCCTTTGTTGAAAAGTCTCAATAGCCCTTTGGTCTTCTGAGACTGTATCTTTGATATTCTTGGAGTAGACGAGAGTGTCGTGCTCCACCATGTTCACATCAATCCACCTTTGAAGACGTGGTTGGAACGTCTTCTTTTTCCACGATGCTCCTCGTGGGTGGGGTCCATCTTTGGGACCACTGTCGGCAGAGGCATCTTGAACGATAGCCTTTCCTTTATCGCAATGATGGCATTTGTAGGTGCCACCTTCCTTTTCTACTGTCCTTTTGATGAAGTGACAGATAGCTGGGCAATATAATCGAGAGAGTTTCCCGATATTACCCTTTGTTGAAAAGTCTCAATAGCCCTTTGGTCTTCTGAGACTGTATCTTTGATATTCTTAGGTAGACGAGTGTCGTGCTCCACCATGTTAACAAGCTGCTCTAGCCAATACGCAAACCGCCTCTCCCCGCGTTGGCCGATTCATTAATGCAGCTGGCACGACAGGTTTCCCGACTGGAAAGCGGGCAGTGAGCGCAACGCAATTAATGTGAGTTAGCTCTCATTAGGCACCCAGGCTTTACACTTTATGCTTCCGGCTCGTATGTTGTGTGGAATTGTGAGCGGATAACAATTTCACAGGAAACAGCTATGACATGATTACGAATTCGAGCTCAGTACCCCTGGATTTTGGTTTTAGGAATTAGAAATTTTATTGATAGAAGTATTTTACAAATACAAATACATACTAAGTTGTACAAAAACCAGCAACTCACTGCACTGCACTTCACTTCACTTCACTGTATGAATAAAGTCTGGTGTCTGGTTCCTGATCGATGACTGACTACTCCACTTTGTGCAGAACAGATCTAGAGCTCTTAGTAGAGCGGGAGATGTTGGTCGGCACGAGCATGATGTTCATGAGGTCGAACTGTGTGCCAGAGTTGAGGGTCACGTTGATGTCGAGCGGCACGTCGGAGTTGGAGGAGGCCACCACGTTGCCGATGTTGATGTCGGAGAAGCGAGCACCGTTGTCGTTCACGCCGTCGTTGTTTGTGGTGGTGTTCACGTTTGTGCGGTGTACACACGGCCGTTGATGGTCACGCGGATGGTGGAGTTGCGATGGAAGACACGCGGAGGTAGAGGTTGTAGGAGTTGCCGTTACCGCGGAGGTGTAACGAGCGGTGGTGTTGTTCTGCTCGAAGCGGAGAGAGTCGCCCTGGTTGCCGAACTTCTCGGAGATGAAGGTGCGGGTCTGGTTGTTCACCTGGGTAGCGTGGATCGGGGAGATGGTGAAGCCGGTGTAGTCGTTTGGGCGAGGTGGGTCATGGAGCCGTTCTCGTGCACAGCGTGGATGTTGTTCTTGCGGTTGTGCACGGACACCATGTAAGCGCGTGCACCTCCAGGGTGCCGGACGGGAGGCGATGTTGCGGATCTCGTTGTAGTGGAGTGGGCGACGGAGGTCCTCGTTGCGCACCACGAGCGGCACGCCGGAGATGTTGCGGATGAAGTAGTCCGGGAAGTAGTTGGAGTTGCCGCGTGCGGTGAAGGCACCGGAGCGGAGGCCGAGGGTGGTCTCGAAGGACTCGGTCTGCCAGTTGGTCACGGTAGCCACGCCTCGCGGTCGGAGCCGAGTCGAGCCAGGAGCGCACGAACGGTGTGAGCGGAGGGAGGAAGGTAGAGCAGTTGAAGTTCTGGTTGAACGGGAAGCGCCGATGTCGCCGGAAGAGATGCCACCGAGTAGTTCACGCGAGCGGCGAGGAGGGCGTGGGTAGTTGTGGAGCCAGGGGCCCGATGTTCGGGAAGGTGTTGGAGAGGCGGGCGCCGGAGAAGCCGTTGAGCACGTAGTTGGGTTCACCTGGAAGAGGAGTAGAGGAACAGCCAGTCCTGGGAGGTGAAGGACTGGGTCTGCTGCGGGCCGGAGCCGGAGGCGTAGAGGTTGGCGCCGGAGGACACGAGGAGGGACTGGTACTTGAAGAGGGACCAGATGGACACGTACTCGAACACGTTGAGGAACATGTAGGTGCGGAACTCAAGCATCTCGTGGAGAGGGTGTTGAGCTTCTTGAAGGCTGTCTGGTAGGTGTTGATGCAGTAGTTGGAGTACTCCTTGGTGTAGTCCTTGAGGTAGCCACGGTAGGTGCGGAGAGTGGCAGCGGAGATACCCCTCACCTCTGCGTTGAGGATCACATCGCGGATGAAGGAGAGGTGGAGGTTGGCGGCCTGAGCGAAGAGTGGAAGGAGAAGGAGTTCGTGTACGGAGCTGGAACTGGGTGAGGCGGTTGAGGAAGAGCTGCTGCATGGTGTTCACCGGGGATGTGATGGAAAGGAGAGTCGGGTTACGGTTCGGGTTGAGGAAGTTGTCCACCTGACGGTTGAACTCGGCAACGTTCTTCTGGAGGCCCTCAAGTTCGGCGTTCACGCGGGAGAGGGTGTCAGCGTTGAGGCGCTGGTTGAGGAACTTCTCGGTCTCACGGAGGATGTCCTGCATGAGCTTGGTGTTGTCGTTTGGGAACACGAGGTCCCAGAGGCCATTGAGGATGCGCTTGGCAGCGAAGCCTCCGAGCTTCTTGAGGAGGAAGATGGCCACAGTACCCACCACAGGAGCCACGTAGAGTGAGTGGTCGTCGCGCTTCCACTCGGTCCACTCCTTCTGGATGGTGTCGAGGACTTGTACTGGAAGGAGAACGGGTCCTGGGCCATCACGTTGTGGCCGTCGCAGATGGTGGTGCGTCCGGAGTTGAGCACGGAGCTCCTCCACCCTTCGGGGCCTCGCACCTGATCGATGTGGTAGTCGGTCACGTCGGTCTTCAGGCCGATCTGGTTGCTGCTGGTGAACAGCTCGTTCACGGCCTTCTGAGCCCTCTCCAGGTCGTACTCGGCCTCGAAGGTCACCTCGGCGGGCACGAACTCGATGCGGTCGATGTACACCTCGTTGCCGCTGTTGGAACGTGGGCGCTCAGGTGAACACGCTGCTGCCGTTGCTGAAGTTGAAGGGGTGGTGAAGCCCACGGTGCGGAAGCTGCCGCTCTGCAGGTTGCTGCCGCTGCTCATGGTGGCGCTGAAGTTGCCCTGGTTGATGGGGCGGCCGTCGATGCTGGTGTGAACTGCAGGTTGGTGGTGCTGGCGTAGCGGATGCGGACGCGGTAGCGCTGGCTCAGGGGGCGGTGATGTTCACGCGCAGGGTGCTGATCTGGCCGGGGCTGGTGCGGCGCAGGATGTCGCCGCCGGTGAAGCCGGGGCCCTTCACCACGCTGGTGCCGCTGCCCAGGTTGGTGCTCTTGGTCAGGGATCTGGGTGATCTGGCTGCTGGGGATGATGTTGTTGAACTCGGCACTGCGGTGAATCCAGCTGAACATAGGTGCACGGATGATGCTCACGCTGCTGTTGCTGAAGCCACTGCGGAACATGCTCACGTGGCTCAGACGGTGGCTGAAGCCCTGTCGAGGTGGCGTTGTTGTTCTGAGGGGATCTCGTCCAGGCTGTCCACGGTGCCGCTCTTGCGGTACACGGCGCTGGGCAGGTTGCTGCTGGTGCCGTAGGCGAACTCGGTGCCGTCCAGCACGCTCAGCTGCTGGTTGTTGATGCCGATGTTGAAGGTCGACGGTACAGGGTGCTGCTCAGGGTGCGGTACACTCCTGGCCCAGCTGTGCCACGATGCGCTGCTGAGGTGCAGCGTTGCCCATGGTGCCGTACAGGGGAAGGTGAACTCGGGGCCGCTGAAGCCGACGGGGCTGGCCGCGATCTGGTGGCCGCTCCAGTAGTACTCGCCGCGGTGGGCGTCGGTGTAGATGGTGATGCTGTTCAGGATGTCCATCAGGTGGGGCTGCGGATGCTGCCTCGATGCCCTGGGCGCTGCCGCGGAAGCTGCCGTCGGGTTCTCCAGCACGGGGTTGGTGTAAATCTCGCGGGTCAGCTGGCTCACGGTGCGGATGGGGTAGGTGCGGCTGTCGTAGTTGGGGAACAGGCTCACGATGTCCAGCACGGTCAGGGTCAGCTCGCGGCGGAACTGGTTGTACCTGATCCAGTCGCGGCTGTCGGGACCCGCGCTCCAGGCCGGTGTTGTACCAGCGCACGGCGTGGTCGGTGTAGTTGCCGATCAGGCGGGTCAGGTCGTTGTAGCGGCTGTTGATGGTGGCGGCGTCGAAGCCCCAGCGCTGGCCGAACACGCTGACGTCGCGCAGCACGCTCAGGTGCAGGTTGGCGGCCTGCACGTACACGCTCAGCAGGGGCACCTGGTAGTTCTGCACGGCGAACAGGGGATGGCGGTGGTCAGGGCGCTGTTCATGTCGTTGAACTGGATGCGCATCTCCTCGCGCAGGGCGGGGTTGGTGGGGTCGGCCTCCCACTCGCGGAAGCTCTCGGCGTAGATTTGGTACAGGTTGCTCAGGCCCTCCAGGCGGCTGATGGCCTGTTGCGGGCGAACTCCTCGATGCGCTGGTTGATCAGCTGCTCGATCTGCACCAGGAAGGCGTCCCACTGGCTGGGGCCGAAGATGCCCCAGATGATGTCCACCAGGCCCAGCACGAAGCCGGCGCCGGGCACGAACTCGCTCAGCAGGAACTGGGTCAGGCTCAGGCTGATGTCGATGGGTGTAGCCGGTCTCGATGCGCTCGCCGCCCAGCACCTCCACCTCGGGGTTGCTCAGGCAGTTGTAGGGATGCACTCGTTGATGTTGGGTTGTTGTCCATTGTTGGATCCTCTAGAGTCGACCTGCAGAAGTAACACCAAACAACAGGGTGAGCATCGACAAAAGAAACAGTACCAAGCAAATAAATAGCGTATGAAGGCAGGGCTAAAAAATCCACATATAGCTGCTGCATGTGCCATCATCCAAGTATATCAAGATCAAAATAATTATAAAACATACTTGTTTATTATAATAGATAGGTACTCAAGGTTAGAGCATATGAATAGATGCTGCATATGCCATCATGTATATGCATCAGTAAAACCCACATCAACATGTATACCTATCCTAGATCGATATTTCCATCCATCTTAAACTCGTAACTATGAAGATGTATGACACACACATACAGTTCCAAAATTAATAAATACACCAGGTAGTTTGAAACAGTATTCTACTCCGATCTAGAACGAATGAACGACCGCCCAACCACACCACATCATCACAACCAAGCGAACAAAAGCATCTCTGTATATGCATCAGTAAAACCCGCATCAACATGTATACCTATCCTAGATCGATATTTCCATCCATCATCTTCAATTCGTAACTATGAATATGTATGGCACACACATACAGATCCAAAATTAATAAATCCACCAGGTAGTTTGAAACAGAATTCTACTCCGATCTAGAACGACCGCCCAACCAGACCACATCATCACAACCAAGACAAAAAAGCATGAAAAGATGACCCGACAAACAAGTGCACGGCATATATTGAAATAAAGGAAAAGGGCAAACCAAACCCTATGCAACGAAACAAAAAAATCATGAAATCGATCCCGTCTGGAACGCTAAACCATCCCAGGATTCCCCAAGAGAAACACTGGCAAGTTAGCAATCAGAACGTGTCTGACGTACAGGTCGCATCCGTGTACGAACGCTAGCAGCACGGATCTAACACAAACACGGATCTAACACAAACATGAACAGAAGTAGAACTACCGGGCCTAACCATGGACCGGAACGCCGATCTAGAGAAGGTAGAGGGGAGAGGACGAGCGGCGTACCTTGAAGCGGAGGTGCCGACGGGTGGATTTGGGGAGATCTGGTTGTGTGTGTGCTCCGAACAACACGAGGTTGGGGAAGAGGGTGTGGAGGGGTGTCTATTTATTACGGCGGGCGAGGAAGGGAAAGCGAAGGAGCGGTGGAAAGGAATCCCCGTAGCTGCCGGTGCCGTGAGGAGAGGAGGAGGCCGCCTGCCGTGCCGGCTCACGTCTGCCGCTCCGCCACGCAATTTCTGGATGCCGACAGCGGGCAAGTCCAACGGTGGAGCGGAACTCTCGAGAGGTCCAGAGGCAGCGACAGAGATGCCGTGCCGTCTGCTTCGCTTGGCCCGACGCGACGCTGCTGGTTCGCTGGTTGGTGTCCGTTAGACTCGTCGACGGCGTTTAACAGGCTGGCATTATCTACTCGAAACAAGAAAAATGTTTCCTTAGTTTTTAATTTCTTAAAGGGTATTTGTTTAATTTTTAGTCACTTTATTTTATTCTATTTTATATCTAAATTATTAAATAAAAAACTAAAATAGAGTTTTAGTTTTCTTAATTTAGAGGCTAAAATAGAATAAAATAGATGTACTAAAAAATTAGTCTATAAAACCATTAACCTAAACCCTAAATGGATGTACTAATAAAATGGATGAAGTATTATATAGGTGAAGCTATTTGCAAAAAAAAAGGAGAACACATGCACACTAAAAGATAAAAGCTGTAGAGTCCTGTTGTCAAAATACTCAATTGTCCTTTAGACCATGTCTAGCGTTCATTTATATGATTCTCTAAAACAACGATATTATTGTAGTATATAGATTATATTATTCGTAGAGTAAAGTTTAAATATATGTATAAAGATAGATAAACTGCACTTCAAACAAGTGTGACAAAAAAATATGTGGTAATTTTTTATAACTTAGACATGCAATGCTCATTATCTCTAGAGAGGGGCACGACCGGGTCACGCTGCACTGCAGGCATGCAAGCTTGGCACTGGCCGTCGTTTTACAACGTCGTGACTGGGAAAACCTGGCGTTACCCAACAATCGCCTTGCAGCACCCCCCTTTCGCCAGCTGGCGTAATAGCGAAGAGGCCCGCACCGATCGCCCTTCCCAACAGTTGCGCAGCCTGAATGGCGAATGCTAGAGCAGCTTGAGCTTGGATCAGATTGTCGTTTCCGCCTTCAGTTTAAACTATCGGTCCGTTGGCGTCGGGCGTGTGGTTGAGCTCGAGCCGTAGAACTTGTCTTCCGGGCACCGAGCGTCATCGCGATGAGCCTCCATCTTGGTCGCGGCGGTGGGAGTTCTTCATGCAGTCGATGTGGCTGCAGTCACCACTGGCGTCGGCGTCGGACCCCGGCTCGATGGCGATCGCAACAGTTGACGAGCCTCGTACTGGCACATTGTTCGGCCGGAAGTGGGGCGTAGCACATAGCGTGGAGTGCTTCAGGCGAGATGAAGAAGCGAGCCTGCGGCCGCTCTGTCTTCATCGACCGTCGAAGAACATGGTCGAGTTCGGTTGGATCGAGGCCGGGGTGAGTCGACCCATTCACCACGAATCGCGAGACACAGGACTTGATGGCCTTAGAAGCGACGAGATCGCCGCCCATGATTTCCGCCACTTTGCGATTCTACAGAGGCCTCTCGGCACTAGATGATCTCCCGGGGAAGGATGACACCACGATTCACGGATGAGACTCGGTAGTGTACCGCAACCTGCCTGTCGGATCACCGCGTCTGGACCGAATTTGTGGGTAGCGGATCTTGGTCTCGAACGATACCTCGTGATGAAGTAGACTTGCCTCTGGGCGGGCAATGCATGCCTCTTCGTCTCTCAACCACGATCGCGGCTAAGCACACGAGTGGTCGGCGGCGTAGATCAATAGGGCTTCTCCGTGGCGGGGCACCAAGATAGGCGTTCGTGAGGCGCCTTGGTTCAGGGGCTTCCACGGCCTCGAAGTCCAAGAAGCACTCGGCCTTCCTTAAGAGGCGGTATAGGCGAGCCTCTTCGTAGGCGTGAGATGAAGCGGCTCGGCCGCAAGACATCCGTGACTCTCGTCGCCCTTCAAGTCCTTGATGGGCCCCATGCCGTGATGGCCGCGATCTTCTCGGATTGGCCTCGATGCCCCTCGGAGACAATGAACCCCAAGAGCATGCCTCGAGGAACCCCAAAGACACTTCTGGGATTAAGCTTCCGCGTCTTTCGCCTTGAGACATCGAATGTCACTTCAAGGTCGGAAAGGAGGTCTGAGGCTTTCGTCTTGACTACGATGTCATCGGCGTAGGCCTCGCCGTCCGCCCAATGTGTTCGCCGAACACATGGTTCATCACACCATTGATGCGTCGCACCGCATTCCTCAAGCGAGCGGTATGGTGACATAGCGATACATGCAGAAGGGTGTGATGAAAGAAGTCGCGGTGTCGGTTCTTTCATCTGATTTGATGGTACCACAGTAGGCATCGAGGAAAGACAAGTTTCGCACCCAGTGATGGAATCCACAATTTGATCGATGCGAGCGGAGGGTAGGAACCCGGACATGCTTTGTTTAGACAGTGTAGTCTACACACATCCGCCTTTCCCTCCTTTCTTTCTCACAAGAGCGAGGGTTGGCAAGCCATTCGGGATGGAATACCTCTTTGATGAACCTGCTGCCATTAGCTTGGATCTCTCGCCTATGGCTCTGCGCTTTTCTTCATCGAATCGCGTGAGGCTCGCTTCAGTGGGCTCCAACTCGGATATCGGTGGTGCTCGGCGACATCCCTCGGTATCACGGGCATGTCAGAGGACTCCACGCGTCGGCGTTCTTGCGGAGAAAGTCGGCAACCTTTGCTTCCTATTGGGATCGAGCGGGCTTGCGTCCGGATCGCTAGGCGTCGGCTTTGGGTCGAGGGACGGACTTAGTCTCGCGGCTCGAGTTGTCGGCGTGGCGCTTCACGTCGGCACCTCCTTAGAGAGGCTTTCGAGTCGTGATAGGGGCCTTGGATTGGCGAGGGCCTCGGCGTACTCACGCCTCCACGTCGCATTCGAGCAGCGTGTTTGTACGTGGGTGCGTGGTGATGACCGTTGGGGCGGCATCTTGAGCTTGAGGTAGGTGTAGTTGGGGCGGCCATGAACCGGCGTAGCATGGCCTCCCGATCTTGCGTGGTAGGTTCCTCGAACGAGCCACCTCGAGCGTGAGTCTCCCTTGAGGTTGGAGGTGTTCGCGAGCGGGAAGGTCGAGTTGTCAGAAATGGGCGCTTCGGGATGATCGTGGAATGGCGCGCGCTGCGGGCGAGGATAGATCAACCGCGGAGGGCAGGGGTCTCGGCATAGATGATGTTGAGGCTGCTGCCTCAGATCCATGAGGACCTTGGTGAGCTGGCGTCACGGGTGGCGAAATTCGACAGCGGCGGGTATTTCCCGGGCTCGACGCGTGGTCGGGGTGGTCGGCTTTCGAAGGTGATGGGCTTGTCGGACCCGTCTAGGTAGTGAAAGTAGCCTAGAGGGGGTGAATAGGCTAATCTGAGAAAATTTTCACAACAAACTTGAAGATTGTTATATACAGATTCGGCTGGTGCAAGCGGTTCAACAGCTACGTCATAAGTTGAACCACTCAGAACCAATCAAGCTGTTTTATAAACTTTAGACTAAAATCTACTCGAAAGTATTTACATGAAGTTCTTGAGAGAAGTAAGATATAGCTAAGTGAGGATGAAAAGATGAATATGTAAAGGCTATTTTACTTCTAGATAAACTCTCGGAGAAATCTTTATGTCAATCTTGCAAGTAAAAATATCTCAAGTTGAAACAAGTAAGAACACAAGACACAAGATTTAATCAGAGGTTCGCCACACCACAAGGTGTCCTACTCCCGTTGAGGAGCCCACAAAGGGTAGGGTCTTTTCAACCCTAATCCTCCAAAGCCAGCCACAAGGTCAAGGCAATCTCTTCTTATCTTAGCTCAAAGAGCGGGTGATACCAACTTCTTAATTAGTCCACAAATTTGGAGACTCCCAAGTAACCTCGAAGATCTTGAAACCTAGGGTTTCAAGAACACCAAGCACGCAAGAGGGGTTTGCACAAGCTCAAGTCTTTGAAAAAGAGATGGGAGAGAAAACCAAATCGTGAGCACAAGCACAAACCTCACACCCAGAGCTCCTCCAACAAGGTTGAATCTTGAGGAAGATTTAAGTGTGAGAGAGATGGAGAAGATGAGTGCTTTGTCTCAAGTTAGGTGAGCAATAAATGAGTGAGTGTTCACCTGTCTTGAAGAAGAGAGAGAGTCTATTATAGTCACGGCTCAAAACTAGCCGTTTGACCAAAAACCCGTGGCAAGTTGAGCCCCCTAGGCGGTTGAACCGCTCTGACAGTCGGTGTCTGCAGTCATGTGAGGCCAGGCTGTGACGACCTAAGGCGAGTCAGACGAGCGGGATCGGTTGAACCGCCCCCTAGGGGTTGAGCAGCCCGCGACACCCTGGCGACACGACGCGGTCGGTAGCGAGCTGCAGATCAGGTCGAGGGTGAGACCAGTTGAGCTCCCGGACGGTTGGTGGTCTAGACCGAGTTTTGGAGAAAACCCTAGCTCGGTACTGACAGGGCAAGTTGTTGGGGTATGGTCGAGAGAGGTCCACGGTGAAGTTGAAGTTGGAGGGTTGAAAGTTCAACTCAGTTAAGTCGGCAAATTTGAAAAGCTGTTCTTGGTGAAGCTCATCTGGCGAAGTTAAGTTAATTGGAAAGCTCAACTCTTGGTTGAAGAAGTTCAATTGTTTTTAAATAAGAACACTCTAGGTTTCTCAACCCAAACCATGGTCGACCAAATAATGTTAAAGATTTTTGGTTTTCAAAAATAGCTTTTGAATTAGAGGACTTGAGCTATAGCAAACACATGCACAATGCGAGGAAAGAACAAGGAAGAATTACATCATGCAACACAAGATTTTACATAAATTTTATCGTGTTGCATGAAGTCCTTGGTGCTTCCTTAAGTTCTGTTTCCTTCTAATCAAACACTGAGAACAAAAATTGTTAGTACTCTTATTTGTTTTGTCATTAAATCACCAAAACCCTCACTTGGGGTTGATTGCACTTACAATCTCCCCCTTTTTGGTGATTGATGCCAAACAATTAAATCAAAAGATATATATTGCAATAGAAATTTCTTTTGAATGATTGTATGTAGATGGCTCCCTAAATGTGTGCACGATTGTGAATCCAACGTCTTGACATAATATGTCATGTGAGCAACACATTTAGAGATAGTGACAACAACCATACTAAATACAATTATCAGGGGGTGCAAGTGTCATGACAGAGTTGTGATGTACATGACTATCTCTAAAAACCATTATTTTTACTTCATAGCAGTAGACATTACAAGCGATAGGACAAAGATGATGGCGAAGACCATCATATTACTTTGAATAACAATCCAGCTTTATTTCATACAATGGTAAAGGGTACAAGCCACAAGTGGCCAAAACAAAAATAATCCAAAAGAAAAGCGAAGAACCACAAACTAGAAGTTGGATTTTCTCCCCTTTGGCAACAAGTACCAAAAGCGGAGAGAGAGACAGGATACAGAATCAATATCCTCCAGGAGGAGGATATGGCGGGATATGAAGTGTAGTCGGGGAAAAAGTCCTCATCAGCGTCCTCCTCAAAAGTGGAGTCAAGAGTGGCTCATCGTAGAGAAAAAGCAGATCACGAAGATGTCACGATGATCGGAGGAGGAGGTGGTGGTGGTGGTAACGAGCCGGATGGCCTAGGGTCACAGAATGGGTCCGGACCGGGTAAGGAGGAGAGGTGGGAGGATGATTGACCGGATCCCCATGGTAGGGCGGGGGCAAACTCCTGCGATCATCAACATAGACTTCTTCATCTTCATCGTCTACTGACATAAAGGCACCAGTGAGGTCTGCGGTGCCACTCATTGATCTCGGGGAGGAGGATGGAGAGGCACATCAGGCGAGCGAGGGACAAAGGCATGCCCATGGAGGAAGCTTGCGGCGAATTGTCGTCGATCTCCGTGGCGTCGTGCCACCTCATGGACATCAGCCGAAATGTTGCGTCACATGGAGAAAAAATGCGCAAACCCATGGACCAAGCAGCATCCCACGGCCACGACCTCGACCATGACCACGAGCACGTGGCATGGGAGATCCATGACGAGAGAGGACGCGGCGATGAGAGAGGAGGATGGATGATGGGGAGGCTGGCGGGGTGGCTCAGTGGGAAGGCGGGGCCTCCAGAGGCGTGAGGGCCCGCAAATGCGGCGGTGGGTTGGAGGAATCAACCCGGTAGGTATGCGTGGAGTGTTTGCGGGTTTTTGAAGTGGGTGCAGGTGACCATTGATCATCTTCATGATAAAGGGTGCATGAAGACACCTCGAGAGGAGACCAGAAGCACGGACGATCTCTTCGGATCATGTCAAACACATTGATGCGCGAGACCGTTTGGAGCAAGGAGGAGGAGGAAGCTCGACTCTCCCGGGGCGTTTATTTGATTCCGCCCTGGAAGCGGTCATCGGGACAAAGAATTAAGGTACCATGTAGTACCTATGCATGTTAGTGTTGTCGAACTCAGCTCGGTAGAGACATGAATGAATTTGTCTCTTCGCATGGGCGGCCGAATGTCATGCACTCGCATGTTCTTTATACTGAATCTACATCGAAGAAGCCGAATATGGGCAAAGCAGCGATAAGCGACTTTGTGCGATTACCACGGATGAAGAAATACATATCGGGTAATCATACTCATCGGCTTCCTCATCTACTTTCTTTATCGAGAGGGTGGCATAGGCGAGCTACCACCTCATCGTTCGGGGGTATTCCATGGTCATAATGTTCGTAACCCCTTTCTCTCTAAGTTTACGAGTGACGACATGTCGGCATCTCCCATATTCTCACAACCCTCCCGGTCAATAAATCTATGTGGACGGTGGGTGTTTCTTGGAAAGAATGAGAATTGTAGAAATCGGCTGAAAAGATTCAGAACCGAGGGTCTGGACACGGGATGACGAAAGTGAGTAGGGTTGGTGAACCTCGGCAGCTGAAAGTGTCTATTCCTTGAGTGAAGTCGACAGGGAGATTCATGCGGGCCGGGCGACGTTCCCGGATGCATGTGCGATCCGGTGGCGTAGAGAGCATCGGCCGGAAGGTCAGCGAAGTCGTCGAAGCGCCCATCCTCCTCTGACAGGAGCATACGAAGGAAGGCGTGCTCTGACGAGGATGAGGCGCCAACCTTTTGGCGTCTCCGCGAGTCGGCTGCGAGGGCATGGAAGAGCCTCACCATCCATCGGACGAGCCACGTCCTCTCTTAGAGAGGCGGCGAGCGGAGGAGAGGGATGGAAATTCCTCTGAATCTCATAGTCGGCGCTAAACTCAATCACAGGAGGTTGCGACGGCGCACCATGATTGAAAAAGGTGGTGAAGCGTGAACCGGTTGAGGTGTGTGAAAGAGTGGCTCTAAGGTTGTGAAGCGACTAAGGCAAGAGTGAATGAAGTGGGAGAAGAAGGGAGAGTCAAGTATATAGGCGTTGAGCCGGTCCTCGGGACAGTTCAACCCTTATGGACGATTCATGGTTCTAACGATTAATGGTGAAGAACGAAGCAATGAGAGGAAGATGCCAAAAATCAAATTTACAAGCGGGATTACGAAAAAGTGTCACTATGGAGATTCTATGAAAAAAAATCAAAACCAAGTGTTTGCAGGATGAGGCCGGACGGCAAAAAAAAAAAAGAACTGCAAAAGAAAGTTCTGGCGCCTGTATCCTCACCTTCTTTGCTGCGGAAGTGACAAAAAATATTTCTGCTGCCTTGGGTGACAGCGGATCGTCACAAGAGCAAAGGCTACTTTGTGATAGTGTGGGGCTAAAAAGCGTCTTGTACTAATTTCTGGAAGCGGCCCGACGAGGAAAGAAAATTAGCGCCACGACATGCATGGTGTGAGGGGAGAGAAAGAATCTGCGACATGGTCTTGTGAGCTGAAGCGGCGATCAAGAAAAATGCTGCCTTGGAGGGAGAAGTGACCGGACGAGAACAAATAAAAAATTCACGCATCTGCTTGCGCACCTTTCTGGGACACCACAAAAACATGCAGCGCGTGGCGCTAGAACACGGCGGATCTGCATGGCGTACGACATTTCATAAGCTAAGACGAGCGACCCTAAAAAAAAGTTACGGCGTACGACAAAGCGGCCGGACGAGAGAGAAAGAAAAGTGGCCTATTGCGTGACCCTAGCTGTGAAAAGCGAAAAAAATAAATCTGCACGAGACAGAGGGAAGTGACGGACAGGAAAGAGAGAGAAAAAATCTGCACGCCAGCCACAGAGAAGAGCGACCGTAAAAATATTATTCGCGACCCGAAAAAGTCACGGTGTGGACACTGCGGCCGGTAAAAAAATTTCGCCCGGTCATCGGCAAAGTGAAGCGCCGGTCAATAGAAAAAGATCTTTGCGCGCTGAGGATGTGGCCGTTAAAAATCCTGCGTCGACGCTGCTGCGGTGGAGACTGCGTGAACAGACTGCTTGTCGGTCGAGCCGGCGACAAGTTCAGCCGGTCCACCATGTCGGTTCAAGGTTTTAACTAGGATGGTCTGGTAAGCGGTTAAAAGCAGTGGTGAAGTTGGCTCTAAGTTGTTGGCCTCCTCGGTGAACTAGAAAGTTCAGTGGTCAAGCCATTGAGCCCAAAAAGAATCTTAGCACTAAATGCATTGAATTTTAACATTTTCACCTTTTTTTTGAAAGATCATCACATAGCTTTAAACTATCAATCAAAAATAGTGAATCAAACAATTTTGTGACCGTTTTCAAGTTAAGTTCACGAATCCAAGATATTTAATTCACTCTTGAAAACAAAACTAGTGCGATCGAGGTTTTGTGAAGATATCGCCTAGTTGATTTTCGATTGCTCACATGAAACAATTCGATATCTCCTTTGGCTTCGTGGTCTCTCAAGAAATGGTGTCGATGTCAATATGTTTAGTTCTAGAGTGTTGCATGGTTGTTTGCAAGTTTTATGGCACTCTCATTGTCACACAAAGTGGAATTTTGTTAAACTCACAACCAAAATCTCTAAGGGTTTACTTCATCCATAACAAGCTGTGCAACATGCCCTGCTATGTACTCGGCTTCTGCAGTGGAAAGTGCAACACAATTTTGTTTCTTGGAACTCCATAATACTAAGGATCGCCCGAGGAATTGTAAGTCCCGATAGTGCTTTTGATCTACTTTGCAGCCAAATAATCTGGTCGGAATAGCCAAGTAAATCGAAGGGAGCCTTTGGGATACCATAATCCTAGGTTTTGGGTGTGAACTAAGTATCTTAGAATTCTCTTAAGCACTACAAGATGGCAATCTTTAGGATTTGCTTGAAAATGCACACATGCAAACACTCAACATAATATCGGTCTAGATGCACATAAGTAAACTAGTGATCCTATCATTGATCTATATAATGTTTGATCTACTGGGTTTACCTTCCTCATTTAGCAGATGTCCATTTGATGACATTGGAGTCTTGGCGTGTTTTGCCTTTTCCATGCCAAATTTCTTGAGCATATCTTGTATATTTGGTTTGGCATAGAAAAGTACCTTCCTTGGTTTGTTTGACTTGAAATCCCGGAAGTATTTAAGCTCGCCCATCATAGACATCTCAAACCTGTTAGTCATTACTTTGCTAAACTCTTCACAAAATTTTTCATTAGTACTACCAAATATAATGTCATCAACATATACTTGGCACACAAATAATTCATTGTCAACTTTTCTAGTAAATAAGGTAGATCGGCTTTTCCTATTGTAAACCCATTCTTAATTAAAAATTCTTTAGGCAGTCATACCAAGCTCTAGGGGCTTGTTTAAGCCCGTAGAGTGCCTTGTGAAGTAGATAAACATGATTTGGCTTCTTTGGATCTTCAAAACCGGAGGTTGCTCCACATATACTCTCTCTTGTAGTGGTCCATTTAGAAATGTGCTCTTGACATCCATTTGGTATAGCTTGAAATCATGGTTAGTAGCATATGCAATTAATATTCTAATTGATTCTAACCTCTGGCGCATATGTTTAGTAAAATCAAGTCCTTCCACTTGAGTATAGCCTTGGGCAACCAGCGTGCCTTGTTTCTTGTAACCGCACCATGTTCATCTTGTTTGTTCCTAAAGACCCATTTAGTCCCAATCACATTTTGTTTGGGTCTTTGGACTAAGGACAGGACTTCATTGGTGATTGTTTAACTCTCTTGCATGGCAATTATCCAATCGGATCACCCAATGCTTCTTCAACCTTAAGTGGCTCAAGAGAGAAACAAGCGAGTAAAATTCACAAAATTAGCTAAGCGAGATCAGTCGTTACCCCTCCTGATGCTACCGGGATGTTGTCCCTTGGATGATCCCTTTGAATTGTATGATGGACTCTTGGATGAGGCCAGCGATGGTTGTCTTTGTATTTGCTCCTCCTCATCATTCACTTGATCAAGAGGTACTTCACCATCAATGCTTTCGTGTTCATCTTCTACCACTTTGTTGGCATCCTTTGATGATTTTCTTCATGGCTTGGATGACTTGAGGTTGATGGGTTTGCTTGGGTGGAGACTTTGTCCTCACCACCTTTGCACCCACATCAACAACCTCATTGGTCATCGAAAGTTCCTTCCTCATCATCCTTTTCTTGAGGTCTCACTTCACCTATTGCAAGTTTCTTTATGGCTTCAGGTAGTTCTTCATTTCCTGCGGTGACATTAGAAACATGCCCTTGCAACCATTAGACTCATCAAATGTCACGTCTATCGCTATTTCAACAAGACCGTGGTATTGTTGAAAACACGATATCCATGCGCATTTGATGCATAACCAAGCAAGCCCTCGTCCACTCTAGGAGCAAACTTTGAGCTCTTGACTTTCTTGTTAAGAATAAAACATTTACAACCAAATACTCTAAAATAATCAACTTTAGGTTTGTTACCCGTGAGAAGCTCATAAGCGTTCTTTTTGTAGATCTTATGAAGATAGAGGCGGTTGATTGCATGATAGGCGGTGTTGACCGCCTACGCCCAAAGTTGTCGAGTGTCTTGTACTCATCCAACATGGTTCTTGCGGCTTCAATTAGAGTTCGGTTCTTTCTTTCCACAACACCATTTTGTTGTGGAGTGTAAGGCAGAGAACTCATGCTTGATTCCCTCTTCTCCTAAGATTCTTACACTGTGTTCTTGAATTCGTCCCATTATCACTTCTCACTTTCTTGATTTTGAGCTCAAATTCATTTTGAGCTCTCCTCATAATTTCTTCAATATTTCTTGAGTTTCACCTTTATCAAAAAAAAAGCCGGGTGAATCGAGAAAAATCATCAACAATGACTAAACCATACTTACTACCAATGCTAATGTAGGCCACAGTCCAAAGAGGTCCATGTGAAGAAGCTCCAATGGCCTCTTTGTTGTGACCATTCTTTGATTGATGTGGGACTCCATGTTGCTTTCTGCTTGGCATGCGCCACAAACCCTATCTTTCTCAAATACAACATTTGTTAGTCCAATGATATGATTATCCTTTTGAAGTTTGGCCAAATTCCTCATGCAGACATGGGCTGGCAGCGATGCCATGCCACCCTTGTCGGACTTTGCCTAAACAAGTCTCAAGTCACTTTACTTGTTGTGAAATCAACAAGATAAAGCTTGCCCTTCAAGCGCCGGTAAAGGCAAGCGAGAGTCCTCCCTTCTAAGGATCTTCATCCACATCAGAAAATAAACAATTATAACCCATTCCACAAAGTTGTGAACGGACAACAAATTATAGCTTAAAGAATCTACCAATAAAACATTTGAAAGTGATTGTTGGTCGAGATAGGAATTTTACCAATACAATCACCTTACTCTTGCCACTATCTCCAAACACAATTTCTTGTGCTTCTTGAGTTAGTTGCAATGTATGGCTATGTCTTTCTCCCAGTCATGTGATTTGTACATCCACATGGCACCCAACTTGACCCACAGAGAGAGTAGACACCAAAACAAGTTTAGGCTATGCTTTTAGGTACCCAAATTGAATTGGGTCCTGTTAGAATTATAGCCTTGGGTACCCACACACTTCTTTTCATGACTTTTAGTTTAGTGTAGGCACCACATATTTCACAACCAATTTTCCTAAATCCCAAGTCAACATATAATCAAAAGATATGAGTGGAAATGGGAGCATTAGATTTTTGTCCACTTGAGGATCCCTTCCTTCATCTTACTCTTTGTGGAACTCAAGTTAACCTTTACTAAGGTGACTTGTCATTTGAGTGAATCCTCCCCAAGGCATCATATAGTGGTTCCCTCTATGAACTTGGGGATCTCCACCCTTATGCCTTGTCTAGGGTTTTCCTTGGATGAGTTGAACTGGCCCCTTCTTCCATTGTTGGGCTTAGGGACTTGTACTTGGGTTCAACTTTCTTTAACCCATCATTGCTAGAGCATCTTTTCAAAATTTCTTTGACCTTCACATGGGCTATTCTTCCTTGCATGGTTAGTTAGACAACAAGAAGCATGATATTTGGCACAATGTTTGCAAGAATTATCATTTAAGCTAGACTTAGATTCAAGCACTAAGATGAGGCATTGATGTTCTTGCAATTTTCAAGTTGCACTTGAAGTTCTTGATTTTGAACATTCAAGCAACATGCTTGCTTCAAGTGCATTCTTTTCATTTGCAAGATTAGCTATAGAGGTTTTCATATTAATTACTTCTTTATCTTTATTCTCTATAGTTATCTTGACTAAAGATGCTTCCTTAGTCAAGATATCTAGCATTTCATCTTTATTGTGAAGCAATTCTTGAAGCTCTAGGTTTCTTTCCTTTTCAAGGATAAGCAAGTCTTCTTGAGCATCAGAGTTGCTTTTCTTTATCTAGCTTCTCCATTAATTTGGTGATAACATTGTATCCATTCAAGCCAAATTCTTTAATCATTTTATTTTCATGGCAATTTGTTCATCATCATCATCATTTGGAAAATCATTACTAAATAAGGTTACCTTATCTCCTTTTGCCATGAGGTAAGTTGGAGTGTAGGAGTAGTCTTGTAGGTTTGTGACGAGTTGCAGCTTGATGTAGATTGGATGGCCACGTTGCCACCACCTCCTCGGAGCTAGAACTCTCTTCATCAGGGTTCCATTCTTCACCAATATAGGCTTGACCATATTTCTTCTTCTTGAAGTGTCCTTGGTCTTGCCCCCTTTTGAACTTGTCCTTCTTGTATTCCTTCTTAGCTTCTTGTTCTATCTTGTTAGGACAATCCATTTATGAAATAGCATTTGGCACATTCATAGCATGCCCTCTTCTTTCCTTTCCTTTGAAACTTGTCATTTTCCTTACAAATTTCTTGAATGTTTTGATGAACATAGTTTGTGTCTTCATCACTTGAGCTATCTTCATCACTTGAGGTCGCGACCACTTTCTTTGCTTTGTGGTCTCTGTTCTTCTTGGGTTGTCTTGTTCATTAGTGATCAAGGCATGAGAGTCCTTGACTTGATAGGGGCTTCTTGGACTCGTGTTGTTGAATCTTGCAAATAATTGATGATCCCCTCATAGTCATCACGATCCTAATCATCCTTGCAAGACTCTTATCCTTTGCTTTATAAGCTCTCATGAACAATCTTGTGACCTTGAGTCCTCAATCTTACTCCCAAGAACTCTTATTTTGTTGACCAACACCATCAGCCGTCAAAGAGTGATTAGGCGACTCACCTTTGTCCAATCATATCTTGCAGCTCACTTTCCAAAGCTTCAACTCTATGTCTTTTAGCTTTGGGATCTCCTTCATGTGACATTTTAAGAATATTCCAAATGTCACGGGCATCTTCTCTCATTGAACTTTCCAGTATTCTTACGGACAGACTTCCTTTAATTATGCTCTGCTTGAGCATTTGATGAACCTCTTGCATCATTTCGGAGTCATCTCTCTTCTCCTGGCGGACTTATACATACCTACATTAACAATCTCCCAAAGACTAGGATGCACTGGAGTTAAATCGACTTCATCTTGTCGGCCCACTCATCATAGTTCAACTCACTAGAGTTGGTAACTTTCCAAGTGGAGGCGGAAGAGAAGTTGGAATATAATTTCTAGAGTAATCAAATTGAACTTTGCTAAATTCATTACCTTTGCTTTTGGTAGATGATCCTTGGTGTTGAGACTTACCTTGCTCAATACCTTAGACACCAAGATCCACTAGATCGTCATTATAATCAAATGTTCCCTTACCTTTATCTTCTTGGCCTTTCTTCTTGATTCTTCTTCTTTGGCCTTTTTCTTAGCATCTTCCTCTTTCATTTTGAGAACATTCTCTCGGCAATCTTCATGGCGAGTCGAGGACCTTAGGGTCCACTTCCTCAGGAAGATGTGATGGGGATTTCCTCGGGAGAGGATCCACATGGTCTAGTTGGTAGACATGATCGTTTACTCACGTGGTTAGCGTAAAGCAGTCTGACTTTGATACCAATTGAAAGTAGCCTAGAGGTGAATAGGCTAATCTGAAAATTTTCACAACAAACTTGAAGATTGTTAGATACATTCAGCCAGTGTGGTAGGTTCCACATGACTACGTCATAAGTTGAACCTCTGAACAATCCAAGTATTTATAAACTTTAGACTAAAATCTACTCGAAAAGTATTTCACAAGTTCTTGAGAGAAGTAAGATATAGCTAAGTGAGGATGAAAGATGAATATGTAAAGGCTATTTTACTTCTAGATAAACTCTCACGGAGAAATCTTTATGTCAATCTTGCAAGTAAAAATATCTCAAGTTGAAACAAGCAAGAACACAAGACACAAGATTTAATCCGAGGTTCAGCCACACCACAAGGTGTCCTACTCCCGTTGAGGAGCCCACAAAGGGCGGGTCTTTTCAACCCTAATCCTCCAAAAGCCGACCACAAAGGTCAAGGCAATCTCTTCTTATCTTAGCTCAAAGCGGGTGATACCAACTTCTTAGGGTCGTCCACAAATTTGAGACTCCCAAGTAACCTCGAAAGATCTTGAAACCTAGGGTTTCAAGAACACCAGAACTCACAAAGGGTTTGCACAAGCTCAAGTCTTTGAAAAAGAGATGGGAGAGAAAACCAAATCGTGAGCACAAGCACAAACCTCACGAAGAGCTCCTCCAACAGGTTGAATCTTGAGGAAGATTTAAGTGTGAGAGATGGAGAGATGAGTGCTTTGTCTCAAGTTAGGTGAGCAATAAATAGGTAGTGTTCAGCCGTCTTGAAGAAGAGAGAGGGTCTATTTATAGTCACGGCTCAAAAACTAAACTTTTGACCAAAAACCGTTTGGCAGGTTGAGCAGCCCCCCAGCCAGTGTGGTGTCGCCGATCGGTGGCGGTATTGCTGACCCGACCTGCGGCGGGTCGAGCAGGCGAGACAGTTGAACGGCCCCGGGCGGTTGGCCGCCCTGACACCTGGCGACTGGGCGCGGTCGGCCCGCCGATGCGGTCGAGGTCGAGAAAGTCAGGAACCAGTTGAGCTACCCGAGGACGGTCGGTGGTCTAGACAAGTTTGGAGAGAAAACTGACTCAGTGCCTGGGGGCAAGTTCATAGGTATGGTCGAGAGAGGTCCGGTACAGGTTGAAAGTTCCTTTCGATTGAAGTCGGCTCGGTGAAAATTCTTGGTGAAGAAGCTCATCACCTGAAGTTGAAGTTAAATTGGTGACTCAGTACGATTGAAGAAGTTATTGTTTTCAGCAAGAACACTCTAGGTTTCTCAACCCAAACCATAGTCGACCAAATAATGTTAAAGAGTTTTTGGTTTTAAAAATAGCTTTTGAATTAGAGGACTTGAGCTATAGCAAACACTGTGCACAATGCGAGGAAAAGAACAAGGGAAGAATTACATCATGCAACACAGATTTTACATAAATTTTATCGTCAGTTGCGCGAAGTCCTTGTGCTTCCTTAAGTTCCTGTTTCCTTCTAATCAAACGAGAACAAAGCTGTAGTACTCTTATTTGTTTTGTCATTAAATCACCAAAACCCTCACTTGGGGTTGATTGCACTTCTGGTGAAGTGATACCTTCACCAGCAGACCTCCCGGCGCTCTTGCTTGCGGTCTGCGTAGAGGCGTTGCCGGCTTGCAGTAGATCATGGGCGATCAGCGGACCTCGAGAACTCTGCTGCTGGTGATCTTCCTTCTTGTCGTTGTCGCGAGCCTGCCACCCTCACTGGGTGGCGAGCCACCTGTGGAAGTGGCGCCGAAGCATGGCGCCTCCTCAGGGTATTGCTTGGCGGGCCTGATGATAGGGGCACGGCTCCTTATATCTTGTCGAAGGGTTGGCACCTCCGGGGTTTCAGGGTTCTTGTACTGTGGCCCGGCAGGTCACGCGTCGCGGCGTCGCGTTCGCTTAAACTTCTTCTTGCCTTTCTTCTTGGCGCCTTGCTGAGTTGGCCTTCGGCGTCTTCGGTGGGTAGCCACAGGGCTTGTCCTTGGGAAGATAGCCTCAGCCTCACGGCGGAGGTGAACTTGAGTGGCGATGTCCATCATTTCGCTACTACAGTGGGGTTTTGCGACCCGACTTGCCTGACAGGTCGCGGCAAGTATGTGGCGAGGCGGCGCCGATGACATCTGAATCGGTGATGTTGGGCGGCTCGGTCGCGTTGCCGAGTGCCGGATGTAGTCGGAGGACTCTCCATTTGCTGTCGGCGACTTTGGAGGTCCCGAGGAGTTTCACAGGCGCGCGTCGTGCCAGAAATGCCAGCGAAGGCTTGGACGAGTCATCGATTAGAGATCTGCCGGGCGGGTGCTCCAACCGAACCGCGGCGGTGTCGGTGGAGAGGCGAGGAGATTGCGGATGATGGGTGTCATCGTCAGTTCCACCTAGTTAGCGAGGCGGTACCGGTGATGCGAGCCACGGTTCGATCTCGTCTCCCAAGTACTTTGTTGATAGTAGTCGGGGTTGGGCGGGTCGAGAGCAGCCCCGTCGTATGGCGTGGTGAAAACTGCGACCGGGTGGTTCGGGCGAGGACTCCGTCCTCCCGTTGCCGTGGCGTCCCCACGCTGGGGTGATAGCCTCGGCGCACCCTCTCGTCG
